# The *Cis*-regulatory Logic of the Mammalian Photoreceptor Transcriptional Network

**DOI:** 10.1371/journal.pone.0000643

**Published:** 2007-07-25

**Authors:** Timothy H.-C. Hsiau, Claudiu Diaconu, Connie A. Myers, Jongwoo Lee, Constance L. Cepko, Joseph C. Corbo

**Affiliations:** 1 Department of Pathology and Immunology, Washington University School of Medicine, St. Louis, Missouri, United States of America; 2 Howard Hughes Medical Institute, Department of Genetics, Harvard Medical School, Boston, Massachusetts, United States of America; University of Washington, United States of America

## Abstract

The photoreceptor cells of the retina are subject to a greater number of genetic diseases than any other cell type in the human body. The majority of more than 120 cloned human blindness genes are highly expressed in photoreceptors. In order to establish an integrative framework in which to understand these diseases, we have undertaken an experimental and computational analysis of the network controlled by the mammalian photoreceptor transcription factors, *Crx*, *Nrl*, and *Nr2e3*. Using microarray and *in situ* hybridization datasets we have produced a model of this network which contains over 600 genes, including numerous retinal disease loci as well as previously uncharacterized photoreceptor transcription factors. To elucidate the connectivity of this network, we devised a computational algorithm to identify the photoreceptor-specific *cis*-regulatory elements (CREs) mediating the interactions between these transcription factors and their target genes. *In vivo* validation of our computational predictions resulted in the discovery of 19 novel photoreceptor-specific CREs near retinal disease genes. Examination of these CREs permitted the definition of a simple *cis*-regulatory grammar rule associated with high-level expression. To test the generality of this rule, we used an expanded form of it as a selection filter to evolve photoreceptor CREs from random DNA sequences *in silico*. When fused to fluorescent reporters, these evolved CREs drove strong, photoreceptor-specific expression *in vivo*. This study represents the first systematic identification and *in vivo* validation of CREs in a mammalian neuronal cell type and lays the groundwork for a systems biology of photoreceptor transcriptional regulation.

## Introduction

Transcriptional regulatory networks (TRNs) lie at the center of organismal development and physiology [1], [2]. Transcription factors (TFs) within these networks control the spatiotemporal pattern and levels of expression of their target genes by binding to CREs, short (∼300–600 bp) stretches of genomic DNA which can lie upstream, downstream, or within the introns of the genes they control. Significant progress has been made in the computational identification of putative CREs in a variety of species [3]–[7]. One recent study demonstrated the effectiveness of using deep phylogenetic conservation of non-coding DNA to identify developmentally active CREs in the mouse [4]. However, given the importance of *cis*-regulatory change in evolution [8] and the relatively limited number of deeply conserved non-coding regions in the mouse (∼3000 were identified in [4]), it is likely that there are many tissue-specific CREs which do not fall within such regions.

Despite advances in the design of computational algorithms to identify CREs in mammalian genomes, the development of cheap, high-throughput assay systems for validating these computational predictions in vivo has lagged far behind. Most studies of mammalian *cis*-regulation to date have relied on mouse transgenesis as a means of assaying the enhancer function of CREs [4]. This technique is time-consuming, costly and subject to insertion site effects. On the other hand, rapid assays for mammalian CRE function have been developed in tissue culture systems, but it is not clear how such results translate into the *in vivo* behavior of CREs. We aim to demonstrate in this paper that rapid, inexpensive, high throughput analysis of mammalian CREs can be achieved by exploiting electroporation to introduce CRE-reporter fusion constructs either into living tissue *in vivo* or in *ex vivo* explant culture. This approach retains many of the desirable features of *in vivo* transgenic approaches to CRE analysis but is much more rapid and inexpensive.

Photoreceptor cells are sensory neurons that elaborate a highly specialized, membrane-rich organelle, the outer segment, which is exquisitely sensitive to light. These cells are particularly susceptible to degeneration. There are currently over 180 mapped disease loci which cause blindness in humans (http://www.sph.uth.tmc.edu/RetNet/). Of these, more than 120 have been cloned, and the majority of these genes have been shown to be specifically expressed, or highly enriched, in photoreceptors [9]. Unfortunately, there is currently no systems-level understanding of how transcriptional regulation of these disease genes is globally coordinated.

We aim to provide such understanding via analysis of the mouse photoreceptor transcriptional network. Numerous prior studies have demonstrated a central role in this network for the TFs *Crx*, *Nrl*, and *Nr2e3* [10]–[15]. *Crx* is expressed in both rods and cones and activates numerous genes in both [9]–[11], [16]. *Nrl* and *Nr2e3*, in contrast, are rod-specific and are required for activation of rod genes and repression of cone genes [13], [15], [17]–[20]. *Nrl* appears to be a molecular switch between cone and rod cell fate: if a photoreceptor precursor expresses *Nrl* it becomes a rod, otherwise it becomes a cone [21]. All three genes have been implicated in a variety of blinding diseases in humans [14], [22], [23]. Previous studies of mice with mutations in these TFs identified a range of potential target genes [9], [18], [20], [24], [25].

Here, we present a more complete analysis of the genes affected by these mutations in order to define the nodes of the photoreceptor TRN. To understand how gene expression in this network is orchestrated, we identified and characterized many of the CREs linking these nodes via a combination of computational prediction and *in vivo* validation using electroporation of CRE-reporter fusion constructs. This analysis resulted in the identification of a *cis*-regulatory motif associated with high-level expression in photoreceptors. To test the importance of this motif for photoreceptor expression, a selection filter based on this motif was used to evolve photoreceptor-specific CREs *in silico,* and their functional activity was then demonstrated in photoreceptors. This study demonstrates the feasibility of a high throughput, *in vivo*, non-transgenic approach to mammalian CRE analysis which can easily be applied to a wide range of different tissues.

## Results

### The transcriptional network controlled by *Crx*, *Nrl*, and *Nr2e3*

**Figure 1:**
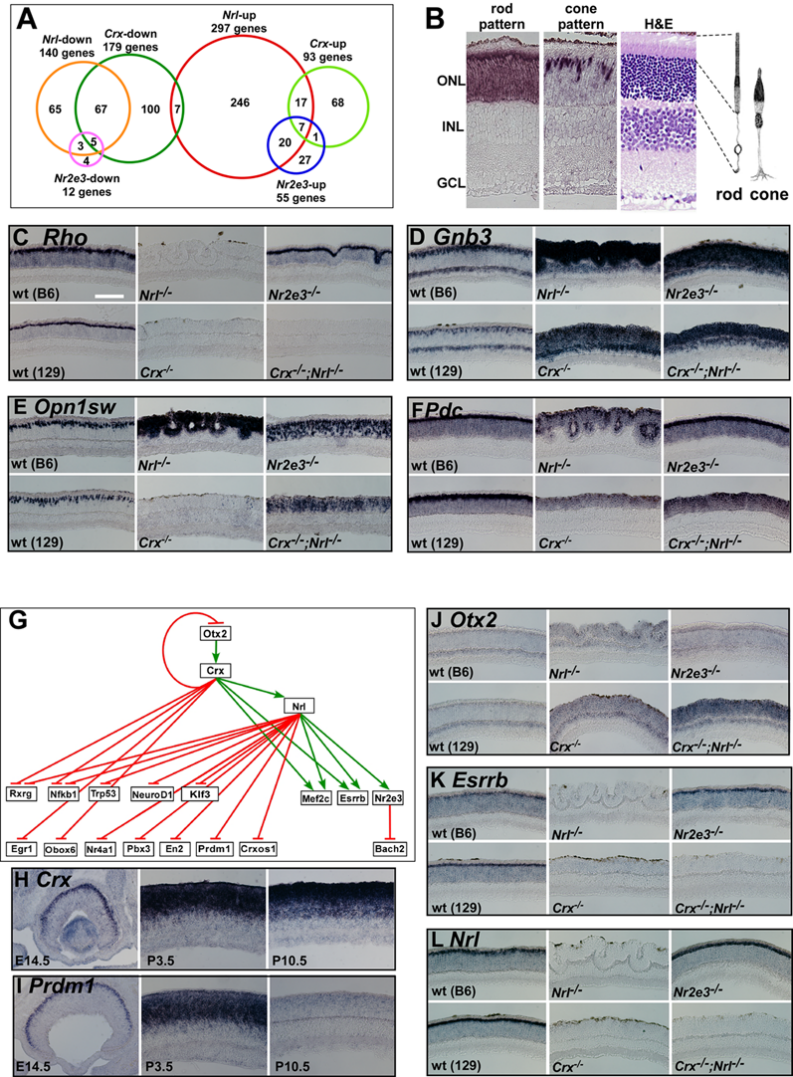
The transcription network controlled by *Crx*, *Nrl*, and *Nr2e3.* A, Venn diagram summarizing all genes dysregulated under high stringency criteria. A complete list of dysregulated genes is given in Table S2. The identity of all genes at points of intersection (including additional intersections not depicted here) are given in Table S7. B, Summary of rod-specific and cone-specific ISH patterns relative to a hematoxylin-eosin (H&E) stained section of retina. The scleral side of the retina is oriented up in all subsequent images. ONL = outer nuclear layer; INL = inner nuclear layer; GCL = ganglion cell layer. C-F, ISH with the indicated probes on four mutant and two wild-type backgrounds. Size bar (in C) = 100 µm G, Summary of transcription factors dysregulated in *Crx^-/-^*, *Nrl^-/-^*, and/or *Nr2e3^-/-^* under high stringency criteria. Green arrows indicate activation and red indicate repression. H and J, ISH with *Crx* and *Prdm1* probes, respectively, on wild-type (CD-1) retinas at the indicated timepoints. J-L, ISH with the indicated probes on four mutant and two wild-type backgrounds. Note that all ISHs were performed on retinas from 6-9 week old animals with the exception of those on *Crx^-/-^* which derive from 4-5 week old animals.

In order to elucidate the global architecture of transcriptional regulation in mouse photoreceptors, analyses of genes expressed in *Crx^-/-^* retinas at P21 were carried out on Affymetrix microarrays. These data were integrated with those of previous studies of *Nrl^-/-^* and *Nr2e3^-/-^* retinas [20], [25]. Using stringent criteria to define up- and downregulation, a total of 628 genes were identified as dysregulated in at least one of the three mutants (Fig. 1A; Tables S1, S2, S3, S4, S5 and S6). 179 genes were downregulated in *Crx^-/-^* (compared to 140 in *Nrl^-/-^* and 12 in *Nr2e3^-/-^*) whereas 93 genes were upregulated (compared to 297 in *Nrl^-/-^* and 55 in *Nr2e3^-/-^*). Our results accord well with two previous gene expression studies of the *Crx* mutant using cDNA microarrays and SAGE [9], [16]. The dysregulated genes comprise many known photoreceptor genes including numerous components of both rod and cone phototransduction cascades.

A remarkable degree of overlap between genes downregulated in *Crx^-/-^* and *Nrl^-/-^* was discovered. 51% (72/140) of *Nrl*-downregulated genes were also downregulated in *Crx^-/-^* using stringent criteria (Fig. 1A). These results suggest that many photoreceptor genes are co-regulated by Crx and Nrl and are consistent with previous studies of individual photoreceptor genes showing co-regulation by these two TFs [26], [27]. To analyze the effects of removing *Crx*, *Nrl*, and *Nr2e3* activity from the retina simultaneously, *Crx^-/-^;Nrl^-/-^* double mutant mice were generated and their retinas were subjected to microarray analysis at P21. Since *Nr2e3* is not expressed in *Nrl^-/-^* [13], these mice are effectively *Crx^-/-^;Nrl^-/-^;Nr2e3^-/-^*. We found an even greater number of downregulated genes in the double mutant (298) than in either of the single mutants using similar criteria (Table S2). This finding further confirms the status of these TFs as major hubs within the photoreceptor TRN.

In order to validate the microarray results, we carried out ISH with probes against 76 photoreceptor genes (∼10% of the genes in the proposed network) on four mutant (*Nr2e3^-/-^*, *Crx^-/-^*, *Nrl^-/-^* , and *Crx^-/-^;Nrl^-/-^*) and two wild-type (C57BL/6 and 129S6/SvEv) backgrounds. The results for *Nrl^-/-^* and a subset of the results for *Nr2e3^-/-^* were reported previously [20], [25] and are reproduced here for comparison. The ISH results directly reflected the changes seen by microarray for nearly all of the genes and genotypes examined. An exception was that five of the probes examined showed a greater degree of downregulation in *Crx^-/-^* than was predicted by microarray (see Table S1). This discrepancy may relate to the fact that the ISH was performed on retinas from a somewhat later timepoint (4–5 weeks postnatal) than when the microarray was performed.

Several examples of ISHs for genes residing at points of intersection in the Venn diagram in Fig.1A are shown in Fig. 1C-F (the full intersection dataset is given in Table S7). *Rho*, which is typical of genes co-regulated by *Crx* and *Nrl*, shows marked downregulation in both of these mutants as well as in *Crx^-/-^;Nrl^-/-^* (Fig. 1C). Many cone genes such as *Gnb3* were upregulated in both *Nrl^-/-^* and *Crx^-/-^* (Fig. 1D). In contrast, some cone genes such as *Opn1sw* were upregulated in *Nrl^-/-^* and downregulated in *Crx^-/-^* (Fig. 1E). The reason for this difference is not known. A minority of photoreceptor-specific genes such as *Pdc* were not changed in any of the mutants (Fig. 1F), suggesting the possibility of regulation by other TFs. ISH results for all 76 genes are presented in Table S1 and Fig. S1. Overall, these results show that the microarray data are a powerful predictor of photoreceptor gene expression patterns.

### Downstream transcription factors and retinal disease genes in the photoreceptor network

Given the importance of TFs as regulatory nodes within TRNs we subjected all 628 genes in our network to Gene Ontology (GO) analysis to identify those downstream TFs which were dysregulated in *Crx^-/-^*, *Nrl^-/-^* or *Nr2e3^-/-^* retinas. Out of 27 genes with the GO classification “transcription factor activity”, we identified 17 sequence-specific TFs (Table S8). Aside from *Crx*, *Nrl*, and *Nr2e3*, this set included two known photoreceptor TFs: *Otx2* and *Rxrg*. *Otx2* was previously shown to be required for photoreceptor formation in the mouse and to activate *Crx* transcription [28]. Derepression of *Otx2* in both *Crx^-/-^* and *Crx^-/-^;Nrl^-/-^* suggests that it is under negative feedback regulation by Crx (Fig. 1J). *Rxrg* is required for regulation of S-cone opsin in mice [29] and is also upregulated in *Nrl^-/-^*. *Neurod1* mutant photoreceptors undergo degeneration suggesting a role for this TF in photoreceptor gene regulation [30]. Although not in the GO list, we also found *Neurod1* to be upregulated in *Nrl^-/-^* (Table S1). Derepression of *Neurod1* and *Rxrg* in *Nrl^-/-^* suggests that both genes are cone-enriched in the adult. Surprisingly, *Nrl* itself was downregulated in *Crx^-/-^* both by microarray and ISH (Fig. 1 and Table S1), suggesting a requirement for *Crx* in maintenance of *Nrl* expression.

Aside from *Nrl*, *Nr2e3* is the only known rod-specific TF. Yet, the microarray data suggest that *Esrrb* and *Mef2c* are also rod-enriched (Fig. 1G, K). *Esrrb* is an orphan nuclear receptor required for placental development and maintenance of “stemness” in embryonic stem cells and germ cells [31]-[33]. Although previously shown to be photoreceptor-enriched [34], complete loss of expression in *Nrl^-/-^* strongly suggests that *Esrrb* is rod-specific in the adult. *Mef2c* is required for heart development [35]. It was markedly downregulated in both *Crx^-/-^* and *Nrl^-/-^* by microarray suggesting that it too might be rod-enriched in the adult. *Prdm1*, also known as Blimp-1, is a well known regulator of B-cell and germ cell differentiation [36]-[38]. We found that *Prdm1* is expressed at high levels in developing photoreceptors in a pattern indistinguishable from *Crx* itself (Fig. 1H, I). In contrast to *Crx*, *Prdm1* expression is undetectable in the adult wild-type retina. Intriguingly, homeodomain-containing TFs on either side of the *Crx* locus were also dysregulated: *Obox6* (upregulated in *Crx^-/-^*) and *Crxos1* (upregulated in *Nrl^-/-^*). The latter result suggests that *Crxos1*, previously reported as a possible antisense regulator of *Crx* [39], is cone-enriched. Overall, these findings implicate a range of TFs in regulation of photoreceptor gene expression downstream of *Crx* and *Nrl*.

**Figure 2:**
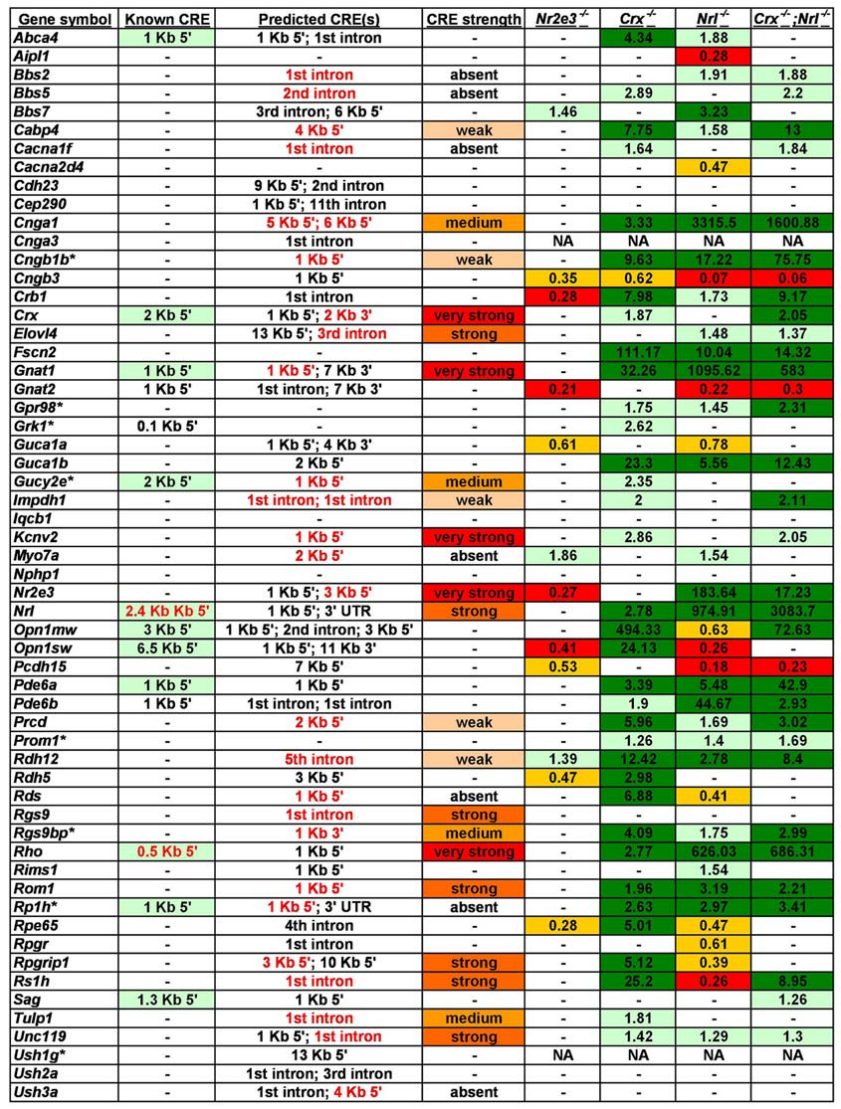
Retinal disease genes in the photoreceptor network. This figure is a manually curated list of all retinal disease genes which are known or are likely to have a photoreceptor-enriched expression pattern. Those gene symbols which differ between mouse and human are marked with a single asterisk, and the human equivalents are given here: *Cngb1* = *CNGB1*; *Gpr98* = *USH2C*; *Grk1* = *RHOK*; *Gucy2e* = *GUCY2D*; *Prom1* = *PROML1*; *Rgs9bp* = *R9AP*; *Rp1h* = *RP1*; *Ush1g* = *SANS*. ‘Known CRE’ indicates the location of published photoreceptor CREs which have experimental support (references are given in MATERIALS AND METHODS). Annotations in this column such as ‘6.5 Kb 5’’ means the tested CRE contained the first 6.5 Kb upstream of the TSS. Known CREs predicted by our algorithm are highlighted in light green. ‘Predicted CRE(s)’ lists candidate CREs predicted by our algorithm which lie within +/− 15 Kb of the TSS. In some cases, two locations are given which represent that CRE prediction closest to the TSS and that with the highest score. Only one location is given when they are the same. Experimentally tested CREs are highlighted in red. The CREs tested for *Crx* and *Elovl4* lie more than 15 Kb downstream of the TSS. The CRE tested for *Tulp1* scores below the cutoff threshold of 200. This CRE was selected for testing during the early phases of this project using an earlier version of PhastCons with which it scored above threshold. A third predicted CRE location is given for *Opn1mw* which corresponds to the locus control region. ‘CRE strength’ indicates the estimated strength of the indicated CRE as tested by electroporation (see Fig. S2). The last four columns of the table show the wild-type-to-mutant ratios of the averaged microarray scores for the given gene. Dark green = downregulated under high stringency (as described in MATERIALS AND METHODS); light green = downregulated under low stringency; red = upregulated under high stringency; orange = upregulated under low stringency. A dash indicates that the gene was not significantly altered in the given mutant. ‘NA’ indicates that this gene is not represented on the Affymetrix Mouse 430 2.0 microarray.

Given the remarkable genetic heterogeneity of human retinal disease, we wished to determine the extent to which the causative genes are regulated by *Crx*, *Nrl*, and *Nr2e3*. A set of 58 mouse orthologs of human retinal disease genes from the Retnet database were manually curated (Fig. 2). This set is meant to encompass all disease genes with known or probable photoreceptor-enriched patterns of expression based on analysis of the literature. 89% (50/56) of those disease genes for which microarray data were available were dysregulated to some extent in at least one of the four mutant backgrounds. This finding suggests that the majority of known human retinal disease genes expressed in photoreceptors is under the transcriptional control of *Crx*, *Nrl*, and/or *Nr2e3*.

### Computational and experimental analysis of photoreceptor *cis*-regulatory elements

These microarray and ISH studies identified many of the important nodes within the photoreceptor TRN. Next we sought to elucidate the connectivity between these nodes by testing the hypothesis that the majority of *Crx*, *Nrl*, and *Nr2e3* target genes are direct transcriptional targets (i.e., these TFs bind directly to the CREs of the genes they regulate). Accordingly, a computational algorithm was developed to identify putative CREs around genes within the network. The algorithm analyzes the genomic region 15 Kb upstream and downstream of the transcriptional start site (TSS) of a gene, assigning a score to successive 500 bp stretches of genomic DNA based on the number, affinity, and clustering of phylogenetically conserved Crx, Nrl, and Nr2e3 binding sites. Those 500 bp blocks with a score above a threshold value of 200 are considered likely photoreceptor-specific CREs. This algorithm was used to make CRE predictions around all genes in the photoreceptor network (Table S1).

**Figure 3:**
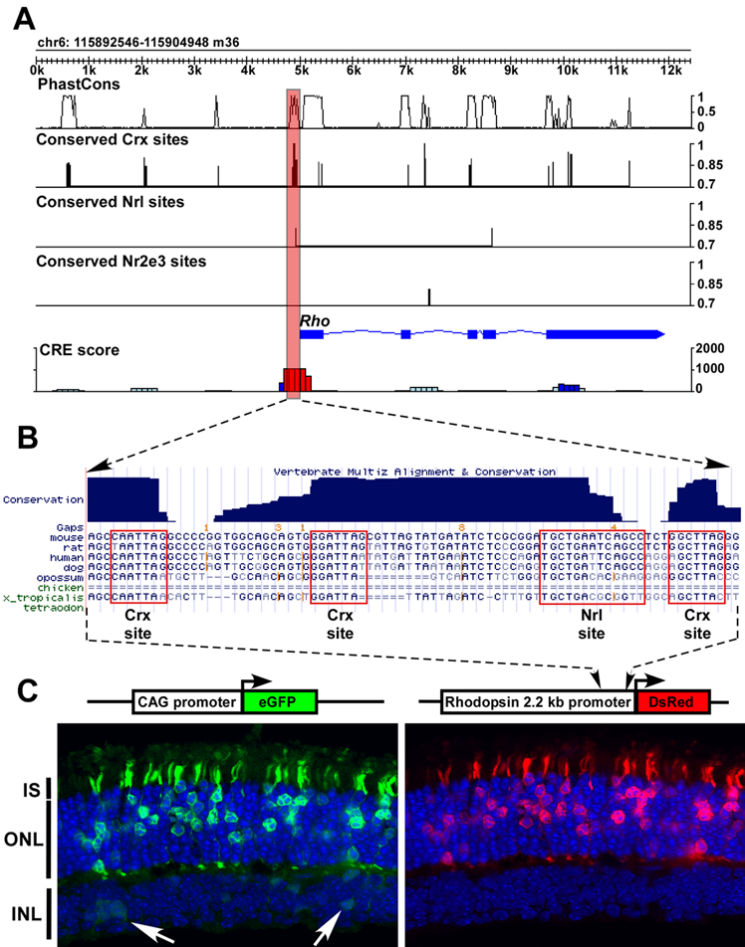
Computational algorithm to identify photoreceptor *cis*-regulatory elements. A, Output of computational algorithm for 12 Kb of genomic DNA around the *Rho* locus. ‘PhastCons’ indicates the degree of phlyogenetic conservation of a given stretch of DNA as determined by the PhastCons algorithm. ‘Conserved Crx sites’ indicates the occurrence of a phylogenetically conserved Crx site. The height of the bar representing a site indicates the ‘affinity’ of the site as measured as the percentile rank of that site's log odds score relative to the consensus site for that transcription factor = 1 (i.e., 100%ile). ‘CRE score’ indicates the likelihood that a given 500 bp stretch (on which the peak is centered) harbors a photoreceptor CRE (details about scoring are given in MATERIALS AND METHODS). Color code: red if score ≥500, dark blue if 500>score ≥200, light blue if score <200. B, Pattern of phylogenetic conservation around *Rho* CRE as displayed by UCSC genome browser. C, Sections of mouse retina electroporated *in vivo* at P0 with the indicated constructs and harvested at P14. The *Rhodopsin* promoter used here derives from cow. The white arrows highlight CAG-eGFP-positive cells in the INL in which DsRed is not expressed. IS = inner segment.

Fig. 3A depicts the output of this algorithm for the *Rhodopsin* (*Rho*) locus. Note that a strong photoreceptor-specific CRE is predicted within the first 500 bp upstream of the TSS. This peak corresponds to a previously well characterized photoreceptor-specific CRE [40], [41]. Fig. 3C depicts a retina harvested two weeks after being electroporated *in vivo* at P0 with a construct containing the proximal 2.2 Kb upstream of the bovine *Rho* gene fused to DsRed. This promoter, which contains the regulatory peak predicted by the algorithm, drives very strong, photoreceptor-specific expression, as shown previously [41]. A literature search resulted in the identification of 14 genes (out of 58 in the disease gene list) for which corresponding CREs had been previously reported (Fig. 2). The algorithm predicts a potential regulatory peak corresponding to these validated CREs in 79% (11/14) of the cases (Fig. 2). In addition to the *Rho* regulatory peak, the successful predictions include a strong regulatory peak ∼3 Kb upstream of the TSS of *Opn1mw*, the location of the well characterized locus control region which drives expression of the X-linked cone opsins in humans [42]. Furthermore, a prior computational and experimental analysis of the photoreceptor TRN validated three novel CREs *in vitro* (*Abca4*, *Gucy2e*, and *Rp1h*) [43], all of which were predicted by the algorithm. These findings suggest that the algorithm has a high sensitivity for identifying active photoreceptor-specific CREs around known photoreceptor-enriched genes.

**Figure 4:**
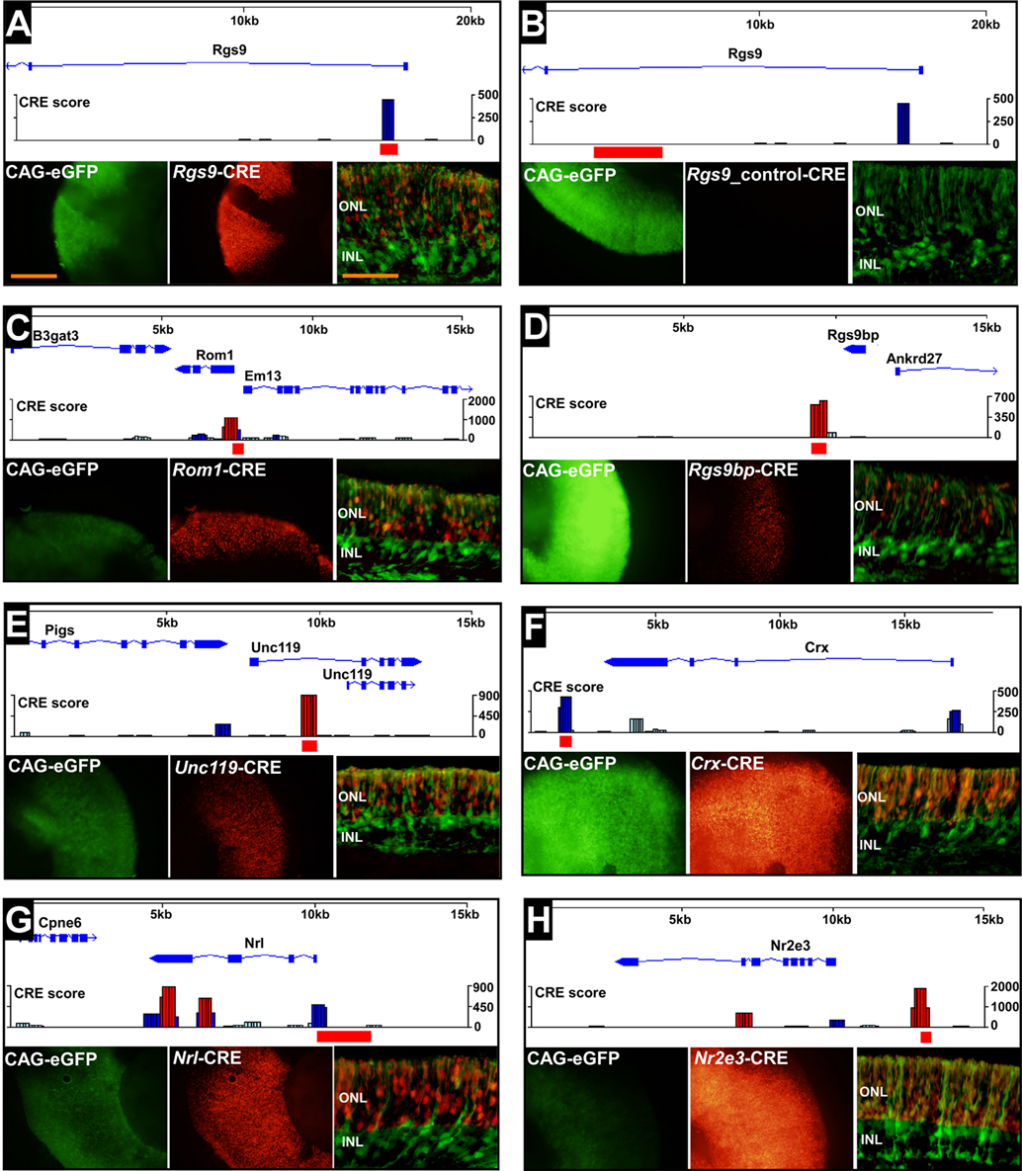
Novel photoreceptor-specific *cis*-regulatory elements. A, The top panel shows the output of our computational algorithm for the first intron of *Rgs9*. The red bar indicates the region of genomic DNA assayed for CRE activity. The bottom panels show flatmount and cross-sectional images of a mouse retina electroporated at P0 with the indicated constructs, cultured as an explant, and harvested at P8. The CAG-eGFP is a ‘loading’ control expressed in all retinal cell types. ‘*Rgs9*-CRE’ consists of the *Rgs9* regulatory peak (indicated by the red bar in top panel) fused to DsRed. The cross-sectional image is a composite of images taken in the green and red channels. Size bar = 500 µm for flatmount images and 100 µm for cross-sections B, A control construct containing the indicated region of DNA from the first intron of *Rgs9* which does not correspond to a predicted regulatory peak. C–E, Additional examples of novel photoreceptor-specific CREs. Note that some annotations of the *Rgs9bp* locus suggest that the indicated regulatory peak falls within the 3′ UTR of this gene. F–H, CREs around the *Crx*, *Nrl*, and *Nr2e3* loci. The regulatory peak upstream of the TSS in F indicates a previously characterized *Crx* CRE [44]. The *Crx*-CRE depicted in the bottom panels corresponds to the downstream regulatory peak identified in the present study (indicated by the red bar). The *Nrl*-CRE shown in G was previously characterized [24] and is included here as a control.

In order to test how reliably the algorithm can predict novel functional photoreceptor CREs, we assayed the activity of predicted regulatory peaks around 26 retinal disease genes that had not been previously studied (Fig. 2). Short PCR products (range: 0.35 to 1.7 Kb) containing the individual predicted peaks were tested for enhancer activity by electroporation as CRE-reporter fusions into retina (Table S9). 73% (19/26) of the computationally predicted CREs tested drove detectable expression in photoreceptors (Fig. S2; Table S1). Novel CREs residing upstream, downstream, and within introns of the genes they control were identified (Fig. 4). For example, Fig. 4A depicts strong expression driven by a 1^st^ intronic CRE from the *Rgs9* gene. When a portion of this same intron not corresponding to a computationally predicted CRE was tested by electroporation, it failed to drive any expression in the retina (Fig. 4B), thus demonstrating the specificity of the prediction. The algorithm predicts a regulatory peak immediately upstream of the TSS of *Crx* and *Nrl*, both regions shown previously to harbor functional CREs [24], [44] (Fig. 4F, G). Furthermore, the algorithm predicts additional peaks downstream of *Crx* and within the 3^rd^ intron and 3′ UTR of *Nrl*. In order to test whether these peaks might also contribute to regulation of these genes, we assayed the peak downstream of *Crx*, which drove very strong expression in photoreceptors (Fig. 4F). The fact that active CREs around the *Crx* locus contain clustered Crx sites suggests that they may represent autoregulatory elements, as noted previously [44]. Alternatively, since Otx2 has a nearly identical binding preference to that of Crx, some of these sites may actually be bound by Otx2 *in vivo*. We also validated a very strong CRE ∼3 Kb upstream of the TSS of *Nr2e3* (Fig. 4H). This CRE is the strongest we have characterized to date. Our analysis has more than doubled the number of known, experimentally validated photoreceptor-specific CREs.

In order to classify the novel CREs according to expression strength, flatmount images of the retinas electroporated with the novel CRE-reporter constructs were photographed at a defined exposure time. The images were compared with each other and with retinas electroporated with two previously characterized CREs, mouse *Rho* and *Nrl* [24], [40]. The novel CREs were categorized into five expression groups (Fig. S2): very strong (4 CREs), strong (6), medium (4), weak (5) and absent (7). Over half of the CREs which showed activity were classified as either ‘very strong’ or ‘strong’. By comparison, *Rho*-CRE was classified as ‘very strong’ and *Nrl*-CRE as ‘strong’. Next we examined the CREs within the five expression classes to determine whether any rules could be derived to explain their differences in expression level. A very simple *cis*-regulatory motif associated with high level expression in photoreceptors was found. The motif consists of a single Nrl site less than 40 bp from a Crx site, with individual and combined affinity scores above defined cutoffs (Fig. S3). When we assessed all 26 tested photoreceptor CREs for the presence of this motif, we found that it was present in the following percentage of CREs: 100% (4/4) of ‘very strong’ CREs, 17% (1/6) of ‘strong’ CREs, 25% (1/4) ‘medium’ CREs, and 0% of ‘weak’ (0/5) and ‘absent’ (0/7) CREs (Fig. S3). This motif is also present within the first 100 bp of the *Rho* promoter. These findings suggest that synergistic activation of photoreceptor gene transcription by Crx and Nrl as first reported for *Rho*-CRE [26], is a common principle of photoreceptor CRE design, at least for CREs which drive high level expression in rods. The absence of this motif from many photoreceptor CREs, some with ‘strong’ expression, demonstrates that clusters of Crx sites (perhaps in combination with sites for other unidentified TFs) can also support strong photoreceptor-specific expression.

If the novel photoreceptor CREs identified here represent the principal regulatory regions controlling expression of the genes in question, then one might expect there to be a correlation between the expression strength of a given CRE and the corresponding gene's transcript levels *in vivo*. In order to test this idea, we compared the expression strength classification of 21 photoreceptor CREs (19 novel CREs along with *Rho*-CRE and *Nrl*-CRE as controls) with their mRNA levels (using the average array value of the gene in question as a surrogate for mRNA levels). We found that the average wild-type (C57BL/6) array values for genes in each of the four expression strength categories were as follow: ‘very strong’ = 19,607; ‘strong’ = 15,308; ‘medium’ = 11,016; and ‘weak’ = 7,927. This result demonstrates that the average transcript levels of genes within a given CRE expression category correlate with the strength of that category. Despite this trend, the genes within individual expression strength categories show a wide range of array values. For example, Rgs9-CRE was rated as ‘strong’, but the array value of the corresponding gene was quite low (i.e., <300). There are a couple possible explanations for this type of discrepancy. First, although array values roughly correlate with endogenous transcript levels, they are not a strictly quantitative measure of mRNA levels. Non-representative array values could therefore result in a lack of correlation between CRE strength and endogenous transcript levels. In addition, for a number of genes (e.g., Crx) there appears to be more than one functional CRE. In such cases, the endogenous transcript levels would reflect the concerted activity of all associated CREs and so would not be expected to correlate perfectly with the activity of any one CRE tested in isolation. Despite a number of individual exceptions, the overall correlation between CRE strength and transcript levels suggests that we have successfully identified the principal regulatory regions of many of the genes under consideration.

### *In silico* evolution of functional photoreceptor *cis*-regulatory elements

**Figure 5:**
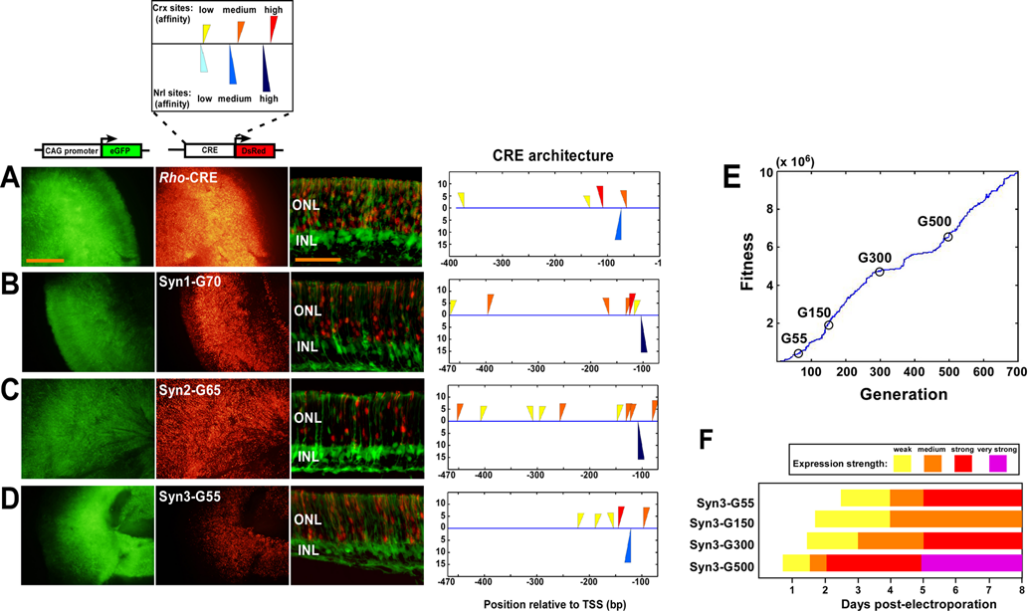
In silico evolution of functional photoreceptor *cis*-regulatory elements. A, Flatmount and cross-sectional images of mouse *Rho*-CRE fused to DsRed co-electroporated with CAG-eGFP. All constructs were electroporated at P0, cultured as explants, and harvested at P8. The column labeled ‘CRE architecture’ shows the distribution of Crx (top half) and Nrl (bottom half) sites with the indicated ‘affinity’ as described in the legend at the top of the figure. The orientation of a given site is indicated by the direction of the triangle representing that site. The score on the y-axis is the log odds score for that site which reflects its closeness to consensus. Size bar = 500 µm for flatmount images and 100 µm for cross-sections B-D, Images of retinas electroporated with the indicated synthetic CREs. For example, ‘Syn1-G70’ indicates a sequence corresponding to the genome of the ‘fittest’ organism of generation 70 in evolutionary run 1. The CREs in B-D derive from three separate evolutionary runs. E, Graph of first 700 generations of evolutionary run 3. The four ‘organisms’ from this run whose genomes were tested for CRE activity are circled and images of their expression patterns are given in Fig. S4. F, Graph of expression strength of four synthetic CREs from evolutionary run 3.

Comparative analysis of the CREs identified in this study demonstrated a correlation between a closely linked pair of Crx and Nrl sites and strong expression in photoreceptors. In order to test the generality of this association, we created a genetic algorithm to evolve photoreceptor CREs from random DNA sequences *in silico* using a selection filter based on clustering and affinity of *Crx* and *Nrl* binding sites (see MATERIALS AND METHODS for details). The purpose of this experiment was to produce sequences that retain the essential features of photoreceptor-specific CREs (i.e., closely clustered Crx and Nrl binding sites), while randomizing all intervening sequences. We found that in less than 100 generations it was possible to evolve CREs with features highly reminiscent of naturally occurring photoreceptor-specific CREs, including some with a strong resemblance to the well characterized *Rho*-CRE (compare Fig. 5A with B-D; Table S10). In order to test whether these *in silico* evolved CREs could drive photoreceptor-specific transcription *in vivo*, we selected three ‘organisms’ from three independent evolutionary runs which had a distribution of Crx and Nrl binding sites resembling that of *Rho*-CRE and which contained the *cis*-regulatory motif described above. None of these evolved sequences had significant linear sequence homology with each other or with *Rho*-CRE. We then synthesized these three 400 bp sequences and cloned them upstream of a minimal basal promoter driving DsRed. This basal promoter alone does not drive any expression in photoreceptors (Fig. S2). When electroporated into explanted P0 retinas, these synthetic CREs drove photoreceptor-specific expression after several days in culture. All three synthetic CREs were scored as ‘strong’ with syn1-G70 and syn2-G65 being somewhat stronger than syn3-G55. These results strongly suggest that closely clustered Crx and Nrl sites are a critical determinant of strong photoreceptor-specific expression and that there is significant flexibility in the architecture of photoreceptor-specific CREs.

We wished to establish with certainty that clustering of Crx and Nrl sites alone is sufficient to support high-level photoreceptor-specific expression in these synthetic constructs. However, the basal promoter used in these experiments contains the proximal 36 bp upstream of the ‘TATA’ box from the bovine *Rho* locus. This stretch of DNA includes the so-called ‘Ret4’ element which consists of a single low-affinity Crx site as well as a binding site for an unidentified ubiquitous factor [45]. To rule out the possibility that this ubiquitous factor is playing a significant role in driving expression in these synthetic constructs, we repeated these experiments using a basal promoter carrying a mutation known to eliminate binding of this ubiquitous factor. The expression level of two constructs (Syn2-G65 and Syn3-G500) were basically unchanged and the levels of two others (Syn1-G70 and Syn3-G55) were only modestly reduced (data not shown). These findings suggest that clusters of Crx and Nrl sites alone are sufficient to drive high-level expression in photoreceptors in this assay system.

In order to determine whether further evolution could be used to generate CREs with progressively stronger expression, CRE-reporter constructs corresponding to the fittest organism from the 150^th^, 300^th^, and 500^th^ generations of run number 3 (the same run from which syn3-G55 was derived) were synthesized. The 500^th^ generation construct drove very strong expression in photoreceptors, comparable to some of the strongest naturally occurring CREs we examined (Fig. S4D). In addition, we found that expression was detectable at progressively earlier timepoints the more highly evolved the element (See Fig. 5E, F; Fig. S4). However, there was not a clear linear progression in the ultimate strength of expression. In fact, the CRE from generation 150 was significantly weaker than that from generation 55 (Fig. S4). The reason for this decrease is unclear but could relate to the acquisition of a binding site for an unidentified repressor.

## DISCUSSION

We have employed experimental and computational analyses of *Crx*, *Nrl*, and *Nr2e3* mutant retinas to define a comprehensive model of the mouse photoreceptor TRN which contains over 600 genes. This model will serve as a template for understanding retinal diseases at a systems level. Using computational prediction and *in vivo* validation we were able to identify 19 new photoreceptor-specific CREs, thus doubling the number of known photoreceptor-specific CREs. Given recent progress in using viral vectors for intraretinal gene therapy in animal models of blindness [46], [47], these novel CREs could prove useful as gene-specific drivers for rescue constructs in human gene therapy. This study also demonstrates the usefulness of electroporation into living tissue as a means of assaying mammalian CREs in a rapid manner which circumvents costly mouse transgenesis. We believe that this approach can be extended to other cell types and organs and can easily be made quantitative.

Our microarray and *in situ* hybridization results showed that many photoreceptor genes appear to be co-regulated by Crx and Nrl, a finding which is corroborated by prior analyses of individual photoreceptor-specific CREs [26], [27]. At the same time, we also showed that maintenance of normal Nrl transcript levels depends on Crx. This latter result suggests that the similarities between the sets of target genes regulated by Crx and Nrl could, in part, be attributable to the decrease in Nrl expression in *Crx^-/-^*. Although we cannot completely rule out this possibility, multiple lines of evidence suggest that, for many co-regulated genes, Crx's transcriptional control is independent of Nrl. First, several cone-enriched genes (e.g., *Opn1sw*, *Pde6h*, and *Arr3*) are downregulated in *Crx^-/-^* and upregulated in *Nrl^-/-^* (see Table S1). This pattern is the opposite of what one would expect if Crx's regulation of these genes was exclusively mediated via maintenance of Nrl transcription. Secondly, we showed that Crx binding sites are abundant within many functional CREs around co-regulated photoreceptor genes. Again, this finding would not be expected if Crx's control of these genes was mediated solely via downstream activation of Nrl. Despite these points, the extent to which the transcriptional changes seen in *Crx^-/-^* are due to a decrease in the levels of Nrl is an important problem for future studies.

Using synthetic CREs, we found that clusters of Crx and Nrl binding sites alone are sufficient to drive strong photoreceptor-specific expression in the mouse retina. This finding was somewhat surprising given the proposed role of other photoreceptor TFs in the regulation of rod photoreceptor gene expression. For example, recent studies have shown that mice carrying mutations in the nuclear receptor TF, *Nr2e3*, show a delay in the onset of *Rho* expression [20] and modest decreases in the levels of expression of several other rod genes [19]. In addition, *Neurod1* is thought to regulate gene expression in rods since mice carrying mutations in this TF show rod degeneration [30]. Furthermore, we demonstrated in this study that there is a small subset of photoreceptor genes whose expression is unchanged in *Crx^-/-^;Nrl^-/-^* (see Fig. S1), suggesting that there must be alternative activators of rod gene expression. In general, however, the effects of *Nr2e3* and *Neurod1* mutations on rod gene expression are modest [18]-[20] (and data not shown). We therefore propose that Crx and Nrl are the primary activators of gene expression for the majority of rod genes in the mouse, and that other TFs expressed in rods such as *Nr2e3* and *Neurod1* are involved in fine-tuning expression.

In analyzing the novel photoreceptor CREs identified in the present study, we were able to define a simple *cis*-regulatory motif associated with high-level expression in photoreceptors which consisted of a pair of Crx and Nrl binding sites. This motif is reminiscent of a *cis*-regulatory motif recently identified in the regulatory regions of *Drosophila* immune response genes which consists of a heterotypic pair of REL and GATA binding sites with a defined orientation relative to each other [48]. The mammalian eye offers other intriguing examples of cell types in which two different TFs play the predominant role in regulation of gene expression. For instance, Otx2 and MITF are known to be the principal TFs involved in regulation of retinal pigment epithelium (RPE) gene expression and are thought to act at the same hierarchical level in the RPE gene network [49]. In addition, gene expression in the mammalian lens is known to depend crucially on binding sites for two types of TF, Maf and Sox [50]. In this case, multiple members of the Sox family are known to participate in different stages of lens differentiation [51], but they all share a very similar binding site. These findings suggest that such ‘two-site’ motifs may be a common feature of metazoan *cis*-regulatory regions. We propose that despite the apparent diversity of TFs expressed in a given cell type (e.g., Fig. 1G) in many cases only two or three TFs may play the major role in control of gene expression. The host of other TFs in that cell type may either be involved in fine-tuning gene expression or in regulating the expression of a selected subset of genes.

Another example of this general principle is the control of cone photoreceptor gene expression in the mouse. Many cone genes are activated by *Crx* and many are repressed (in rods) by *Nrl* and *Nr2e3* [12], [13], [18]–[20], [25]. These three TFs therefore appear to be the primary regulators of cone gene expression. However, several other cone TFs have been shown to regulate a small subset of cone genes. For example, Thrb2 is known to be required for activation of *Opn1mw* and dorsal repression of *Opn1sw* in the mouse [52]. The TF, Rxrg, appears to have an even more specialized role in cone photoreceptors in that it is involved in the dorsal repression of *Opn1sw*, but does not appear to regulate *Opn1mw* [29]. Lastly, a recent paper showed that the orphan nuclear receptor TF, RORbeta, is required for the activation of *Opn1sw* [53]. Although it is possible that these three TFs have other transcriptional targets in cone photoreceptors, it may also be the case that they their transcriptional regulatory role is restricted to only a subset of genes in this cell type.

A recent study identified functional CREs in the human genome by searching for contiguous blocks of extreme phylogenetic conservation and then testing them in transgenic mice [4]. We compared the location of the 19 novel photoreceptor CREs identified here with a set of over 3000 elements conserved between human and *Fugu* identified in that study. We found that none of the 19 CREs were included in that set (data not shown). This result suggests that approaches which rely solely on identifying extended blocks of extreme phylogenetic conservation may fail to detect many cell-type specific CREs. The algorithm used in the present study only requires that the identified binding site itself be phylogenetically conserved. This approach was employed because examination of known photoreceptor CREs demonstrated that, in many cases, blocks of conservation were found to encompass only a single binding site (e.g., the rightmost Crx site in Fig. 3B).

Given the reliance of the CRE-finding algorithm used in the present study on clustering of phylogenetically conserved Crx binding sites which contain the canonical ‘TAAT’ sequence bound by many homeodomain TFs, putative regulatory peaks predicted around non-photoreceptor genes must be interpreted with caution. A preliminary analysis of the genomic regions around 20,000 mouse genes using this algorithm detected a marked enrichment of predicted regulatory peaks around TFs and other developmental regulators (data not shown). Nearly half of the 100 top-ranked genes in this analysis were homeodomain TFs, most of which are unlikely to be involved in photoreceptor development and therefore represent false positives. We believe there are two possible explanations for this result. First, the scoring algorithm is not normalized according to the extent of regional phylogenetic conservation of non-coding DNA. Since developmental regulators including TFs are known to exhibit a greater degree of phylogenetic conservation in their surrounding non-coding regions [54], the algorithm would be expected to predict more regulatory peaks in such regions by chance alone. Secondly, the abundance of homeodomain TFs among the top-ranked genes suggests that the algorithm may be identifying bona fide CREs containing clusters of binding sites for homeodomain TFs other than Crx. Given these caveats, the algorithm presented here identifies functional CREs with considerable reliability when applied to the genomic region around photoreceptor-enriched genes.

## MATERIALS AND METHODS

### Oligonucleotide microarray analysis

Microarray analysis of P21 *Crx^-/-^* and *Crx^-/-^;Nrl^-/-^* retinas was performed on Affymetrix mouse genome 430 2.0 GeneChip arrays (Affymetrix, Santa Clara, California, United States). The full microarray datasets for the *Crx^-/-^* and *Crx^-/-^;Nrl^-/-^* analyses are given in Tables S5 and S6. A comparison of these microarray datasets alongside the results of our prior microarray studies of *Nr2e3^-/-^* and *Nrl^-/-^* are presented in Table S4. A total of three microarray hybridizations were performed for each mutant. Since the *Crx* mutant allele is on a 129 background, we also performed three control microarray hybridizations with retinas derived from 129S6/SvEv mice at P21. For each microarray, RNA was prepared from 4-6 freshly dissected retinas derived from 2-3 animals at P21. Probes were synthesized starting with 10 µg of total RNA for each sample according to manufacturer's instructions (Affymetrix). Hybridization, washing, and scanning of the microarrays were all performed at the Bauer Center for Genomics Research at Harvard University according to manufacturer's instructions (Affymetrix).

Initial data analysis was carried out using the GeneChip Operating System (GCOS) software from Affymetrix. Pairwise comparisons were made between individual mutant microarray results and controls. For our ‘low stringency’ analysis, up- and downregulated genes were determined by using Wilcoxon's signed rank test to compare mutant and control microarrays in a pairwise fashion with a p-value = 0.002. Only those array features which were significantly ‘increased’ or ‘decreased’ in three out of three microarray comparisons were considered to have met our criteria for inclusion in the low stringency datasets.

To create a ‘high stringency’ dataset, we used the same approach used previously for the *Nrl* mutant [25]. First, we separated the features in the low stringency datasets into three expression level categories: (for analysis of downregulated features) (1) features whose average microarray score in the wild-type retina (score_129_) is ≥5000; (2) 1000≤score_129_<5000; and (3) 100≤score_129_<1000. For analysis of upregulated features, the same categories were created using the average microarray score for the mutant. Next, we sorted each of these three sub-lists according to the ratio of their average wild-type to average mutant scores and retained only those features that passed the following thresholds for the three expression categories: category (1) at least a 2-fold change; category (2) at least a three-fold change and category (3) at least a 5-fold change. In this manner, we applied progressively more stringent fold-change requirements for the features showing lower levels of expression. Next, in order to create a non-redundant list of up- and downregulated genes (since our initial feature lists contained multiple Affymetrix features which correspond to a single gene) we used the DAVID functional annotation tool (http://david.abcc.ncifcrf.gov/) to annotate the low and high stringency lists. We then wrote a Perl script to sort and format the DAVID outputs (script available on request). Data from our previously published microarray study of the *Nr2e3* mutant [20] were processed in the same way to generate high and low stringency datasets. High stringency datasets for the *Nrl* mutant were reported previously [25], and low stringency datasets for the *Nrl* mutant were obtained as described above.

Since the *Crx* and *Nrl* mutant alleles are on different backgrounds (129 and B6, respectively), the *Crx;Nrl* double mutant created in the present study was on a mixed 129 X B6 background. Since microarray data from a mixed wild-type background were not available as a control, it was necessary to use the pure 129 and B6 wild-type datasets as controls for the *Crx;Nrl* double mutant. Accordingly, pairwise comparisons were made between individual *Crx^-/-^;Nrl^-/-^* microarray results and both 129 and B6 controls using the GeneChip Operating System (GCOS) software from Affymetrix (i.e., a total of six pairwise comparisons). For our ‘low stringency’ analysis, up- and downregulated genes were determined as described above for the single mutants with the exception that we required that a given feature be up- or downregulated in all six pairwise comparisons in order to be included in our low stringency dataset. High stringency datasets for *Crx^-/-^;Nrl^-/-^* were then obtained in the same manner as described for the single mutants.

### RNA *in situ* hybridization

ISH on tissue sections was performed essentially as described previously [55]. Most of the ISH images of *Nrl^-/-^* and B6 were published previously [25] with the exception of the images for *Esrrb*, *Otx2* and *Neurod1*. ISH images of *Nr2e3^-/-^* using a subset of the probes employed in the present study were also published previously [20] but on sections from earlier developmental timepoints (P14 and P28). Briefly, retinas from 6-9 week old *Nr2e3^-/-^*, *Nrl^-/-^* , *Crx^-/-^;Nrl^-/-^*, C57BL/6 and 129S6/SvEv mice and from 4-5 week old *Crx^-/-^* mice were harvested in PBS and immediately put in 4% paraformaldehyde fixative at 4°C overnight. The earlier timepoint was used for *Crx^-/-^* because of more rapid onset of degeneration in this mutant. Tissue was equilibrated in 30% sucrose/PBS and then embedded in OCT (Tissue Tek). Sectioned tissue from all four mutants and both wild-type controls were placed on a single glass slide which was then hybridized with RNA riboprobes synthesized from PCR products derived from the templates described in our prior study [25] with the exception of probes for *Esrrb*, *Otx2* and *Neurod1*. The latter probes were derived from the following templates: BC044858 (*Esrrb*), AI836617 (*Otx2*), and an in-house clone corresponding to nucleotides 966-1845 of NM_010894 (*Neurod1*).

### Photoreceptor *cis*-regulatory element scoring algorithm

Position frequency matrices (PFMs) were constructed for *Crx*, *Nrl*, and *Nr2e3* based on previous literature [10], [18], [56]. Given that all members of the *maf* subfamily of leucine zipper TFs (which includes *Nrl*) have very similar DNA binding preferences [57], a well characterized PFM for v-maf [56] was used as the ‘Nrl’ PFM. Nrl and other maf subfamily members can bind to 13-bp and 14-bp variants of their binding site which differ by one nucleotide in the middle of the motif. In preliminary computational analyses we found very few instances of the 14-bp motif around photoreceptor genes (data not shown) and we therefore only used the 13-bp motif in all subsequent analyses. PFMs were converted to position weight matrices (PWMs) using the following formula:
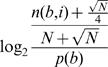
where *n*(*b*, *i*) is the number of occurrences of base *b* at position *i*, *N* is the number of sequences used to create the PFM, and *p*(*b*) is the background genomic frequency of base *b* (given a GC content of 0.43 in the mouse). Site scoring was then performed on the NCBI Mouse Build 36 using the TFBS Perl module [58]. PhastCons release mm8 was used to estimate the degree of conservation for each binding site [59]. Average PhastCons scores for each binding site were calculated and then the binding site scores were weighted according to the formula:
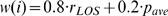
Where *w(i)* is the weighted binding site score, *r_LOS_* is the raw log odds score for the site and *p_ave_* is the average PhastCons score for the site. This weighting ensures that binding sites that lie within phylogenetically conserved stretches of the genome contribute a higher score to the overall score of that 500 bp window. CREs typically consist of closely linked clusters of binding sites for multiple TFs [1], [2]. Accordingly, closely clustered phylogenetically conserved binding sites were given more weight than sites that were less closely clustered. Our scoring scheme computed module scores for 500 bp windows according to the following formula:
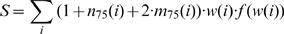
where *S* is the score for the 500 bp window. The summation is done for all binding sites in the window with *i* being the iteration index, *n*_75_ is the number of homotypic neighbors (i.e., Crx-Crx, Nrl-Nrl, or Nr2e3-Nr2e3 pairs) within ±75 bp, *m*_75_ is the number of heterotypic neighbors (i.e., Crx-Nrl pairs etc.) within ±75 bp, *w*(*i*) is the weighted binding score, and *f*(*w*(*i*)) is defined to be 4 if *w*(*i*) is greater than 90% of the maximum log odds score for that particular PWM. This scoring scheme gives greater weight to high affinity sites (i.e., sites whose log odds scores are in the top 10% of all sites for that TF) and closely clustered (i.e., within 75 bp of each other) heterotypic pairs of sites. Once a given 500 bp window is scored, the window is shifted by 100 bp and the process is repeated. CRE predictions were made for a ±15 Kb interval around genes in our dataset. Gene structure information was accessed using the EnsEMBL Perl modules (version 40) [60]. The BioPerl Graphics package [61] was used to visualize predictions superimposed on gene structures. The script for this algorithm is available from the authors on request.

### Literature analysis of previously characterized photoreceptor *cis*-regulatory elements

Through a literature search we identified a total of 14 retinal disease genes for which mammalian CREs had been previously experimentally analyzed: *Abca4*, *Crx*, *Gnat1*, *Gnat2*, *Grk1*, *Gucy2e*, *Nrl*, *Opn1mw*, *Opn1sw*, *Pde6a*, *Pde6b*, *Rho*, *Rp1h*, and *Sag* [24], [27], [41]–[44], [62]–[67].

### Construction of *cis*-regulatory element fluorescent reporters

PCR primers were designed around computationally predicted CREs using Primer3 [68]. The sequences of all PCR primers used (along with restriction sites added for purposes of subcloning) are given in Table S9. Two different starting vectors were used for the construction of the CRE-DsRed reporters: *Rho*-basal and no-basal. The *Rho*-basal vector was used for all CRE that resided at some distance for the gene's endogenous basal promoter region. The no-basal vector was used for those CRE that occurred immediately upstream of the TSS. In the latter case, the gene's own endogenous basal promoter could be cloned directly upstream of the DsRed coding sequence. The *Rho*-basal vector was created by replacing the 2.2 Kb bovine *Rho* promoter region in pRho-2.2K-DsRed [69] with a minimal basal promoter which contains nucleotides -36 to +79 around the ‘TATA’ box of bovine *Rho*. This Rho minimal basal promoter includes the so-called ‘Ret4’ element which contains a single low affinity Crx site (log odds score = 5.1) and a binding site for an unidentified ubiquitous factor [45]. The following PCR primers were used to create this minimal basal promoter: 5′- taccgCTCGAGatatctctagaggtaccgaattcgattcagccgggagcttag and 5′- tccgaaCCCGGGgatgcttct. The 5′ primer includes an *Xho*I site for cloning (in upper case) and the following additional enzyme sites for subsequent subcloning of CREs into the *Rho*-basal vector (from 5′ to 3′): *Eco*RV, *Xba*I, *Kpn*I, and *Eco*RI. The downstream primer contains a *Sma*I site (in upper case) for cloning. This *Rho* basal promoter PCR product was digested with *Xho*I and *Sma*I and ligated into the pRho-2.2K-DsRed vector that had been previously digested with *Sal*I and *Sma*I, treated with calf intestinal alkaline phosphatase (AP), and separated from the 2.2 Kb bovine *Rho* promoter fragment by gel electrophoresis. The *Rho*-basal vector was the product of this ligation. The no-basal vector was created by digesting pRho-2.2K-DsRed with *Sal*I, treating the cut vector with AP, and ligating in a short linker created by kinasing and annealing the following two oligonucleotides: 5′-tcgacgatatctctagagaattcc and 5′-tcgaggaattctctagagatatcg. The CAG-eGFP vector used as a loading control for all electroporations was described previously (referred to in that paper as pCAG-GFP) [69].

A total of six 400 bp synthetic CREs (Syn1-G70, Syn2-G65, Syn3-G55, Syn4-G150, Syn3-G300, and Syn3-G500) were synthesized (Integrated DNA Technologies Inc.) with appropriate restriction enzyme sites added to the ends for purposes of subcloning into *Rho*-basal.

### Retinal electroporation and explant culture

*In vivo* and *in vitro* electroporation and explant culture were performed as described previously [69] with the following modifications. Retinas from newborn (P0) CD-1 mice were dissected in serum free medium (SFM; 1:1 DMEM:Ham's F12 (Gibco), 100 units/ml penicillin, 100 µg/ml streptomycin, 2 mM L-glutamine (Gibco) and 2 µg/ml insulin (Sigma)) from surrounding sclera and soft tissue leaving the lens in place. Retinas were then transferred with forceps to an electroporation chamber containing a 1 µg/µl solution of supercoiled DNA in PBS (model BTX453 Microslide chamber, Genetronics Inc.). The chamber had been previously divided with silicon cement into four sub-chambers each with a volume of ∼60 µl. Next, five square pulses (30 V) of 50-ms duration with 950-ms intervals were applied using a pulse generator (model ECM 830, Genetronics Inc.). Electroporated retinas were removed from the electroporation chamber and allowed to recover in SFM for several minutes before being transferred to the same medium supplemented with 5% fetal calf serum (Gibco) for several minutes. The retinas were then placed (lens side down) on polycarbonate filters (Whatman, 0.2 µm pore size) and cultured at 37°C in SFM supplemented with 5% fetal calf serum for 8 days. Upon harvesting, retinas were fixed in 4% paraformadehyde in PBS for 30 minutes washed two times with PBS. Next, the retinas were flat-mounted (photoreceptor side up) and examined using a BX51 compound microscope (Olympus). Digital images were captured using a DP70 camera (Olympus). After imaging of flatmounts, retinas were equilibrated in 30% sucrose/PBS overnight at 4°C and embedded in OCT. 16 µm cryosections were collected, and confocal micrographs were captured using a BX61WI microscope (Olympus) equipped with a DSU spinning disc and an ORCA-ER CCD camera (Hamamatsu).

The expression strength of the novel CREs were categorized in the following manner. Images of the best electroporated retina for each CRE-reporter construct (defined as that retina with the greatest area and intensity of green fluorescence of the retinas examined for a given construct) were captured in both green and red channels at the same exposure time (1/300 second) for all constructs. These images were then compared side-by-side with images for all other constructs as well as images of retinas electroporated with *Rho*-CRE and *Nrl*-CRE controls. ‘very strong’ expression was defined as red fluorescence (i.e., expression levels of DsRed driven by the CRE in question) that showed extensive pixel saturation (i.e., yellow areas on the image) at 1/300 second exposure. ‘strong’ expression was defined as widespread robust red fluorescence without significant saturation. ‘medium’ expression was defined as red flourescence that was visible at 1/300 exposure but that was considerably weaker or more patchy than that seen in ‘strong’ constructs. ‘weak’ expression was defined as any definite expression in photoreceptors that was not easily visible in a 1/300 second exposure. ‘absent’ expression was defined as absolutely no detectable expression in the retina.

### Algorithm for *in silico* evolution of photoreceptor *cis*-regulatory elements

A genetic algorithm was developed in Matlab to evolve photoreceptor-specific CREs *in silico*. The original inspiration for this algorithm came from a paper by Thomas Schneider [70]. The algorithm consists of four distinct steps: initiation, mutation, selection, and reproduction. In the initiation step, random 400 bp DNA sequences were generated in accordance with the GC content of the mouse genome (0.43), and a single such sequence lacking Crx and Nrl sites (i.e., containing no Crx or Nrl sites with log odds scores greater than 4 and 6, respectively) was selected. This sequence was then assigned to 100 ‘organisms’, each consisting of a single 400 bp genome. In the mutation step, random point mutations were assigned to individual organisms according to a Poisson distribution such that, on average, each organism received one point mutation per generation.

In the selection step, the ‘fitness’ of each organism was determined by assessing the affinity of any Crx or Nrl sites in the organism's genome (scored using the PWMs and TFBS Perl module described above), the spacing between pairs of sites, whether the pairs of sites were homotypic (i.e., Crx-Crx or Nrl-Nrl) or heterotypic (i.e., Crx-Nrl) and the distance of sites from the TSS. Specifically, the fitness score, *S*, for each 400 bp organism was calculated using the following formula:
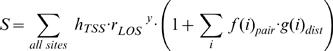
where *r_LOS_* is the raw log odds score of the site being scored; *y* is a normalizing factor to even the contribution made by Crx and Nrl sites, where *y* = 3 for Crx sites and 2.5 for Nrl sites (the raw log odds score for a high affinity Nrl site is greater than that for a high affinity Crx site since the Nrl binding site is almost twice as long); *i* represents all other Crx or Nrl binding sites within the 400 bp genome; *f_pair_* is a weighting term which equals 2 for homotypic neighboring sites (i.e., Crx neighbors if site being scored is Crx; Nrl neighbors if site being scored is Nrl) and 8 for heterotypic neighboring sites (i.e., greater weight is attributed to Crx-Nrl pairs to reflect the *cis*-regulatory motif defined in this study); *g_dist_* is a second weighting term which reflects the importance of site clustering: *g_dist_* = 0 if *i*>100 bp from the site being scored; *g_dist_* = 1 if 40 bp<*i*≤100 bp; and *g_dist_* = 2 if *i*≤40 bp (this weighting term also reflects the important contribution of sites within 40 bp of each other as demonstrated in our *cis*-regulatory motif); lastly, *h_TSS_* is a weighting term which increases from 1 to 4 the closer the location of the site being scored is to the 3′ end of the 400 bp genome (this term is intended to reflect the importance of proximity of binding sites to the TSS; several of the most strongly expressing endogenous CREs examined (e.g., *Rho*, *Kcnv2*, and *Nr2e3*) have key TF binding sites less than 200 bp from the TSS. For purposes of scoring, Crx and Nrl sites with log odds scores less than 6 and 9, respectively, were ignored.

After assignment of a fitness score, the organisms are ranked from highest to lowest fitness, the bottom 50 organisms are culled, and the top 50 are permitted to differentially reproduce to restore the original population of 100. In this reproduction step, the top ten scoring organisms each contribute three identical offspring to the next generation, the 21^st^ to 40^th^ highest scoring organisms contribute two identical offspring to the next generation, and the 41^st^ to 50^th^ highest scoring organisms each contribute one offspring to the next generation. The new population of 100 organisms reenters the algorithm at the mutation step and the cycle is repeated. The script for this algorithm is available from the authors on request.

The full datasets for the three evolutionary runs used in this study are given in Table S10.

### Graphing of *cis*-regulatory element architecture

A Matlab graphic user interface (GUI) was developed to visualize the spatial arrangement of Crx and Nrl sites on a segment of DNA. For color-coding of Crx and Nrl binding sites the following log odds score thresholds were used: 5<Crx_LOS_<7 (yellow); 7≤Crx_LOS_<9 (orange); Crx_LOS_≤9 (red); 9<Nrl_LOS_<12 (light blue); 12≤Nrl_LOS_<15 (blue); Nrl_LOS_≤15 (dark blue). The term ‘affinity’ used in this paper to describe individual Crx and Nrl sites is not actually the true affinity of the binding sites in question but rather a measure of closeness of a given binding site's sequence to that of the consensus site for that TF as derived from the PFMs.

## Supporting Information

Figure S1 *In situ* hybridizations for genes not in Table S1. ISH images on four mutant and two wild-type backgrounds for nine photoreceptor-enriched genes which were not dysregulated in *Crx^-/-^*, *Nrl^-/-^*, or *Nr2e3^-/-^* and therefore not included in Table S1. Size bar = 100 µm.
(7.10 MB TIF)

Figure S2 Expression strength categorization of novel photoreceptor *cis*-regulatory elements. Images of retinas electroporated with the indicated constructs at P0, cultured as explants, and harvested at P8. All 26 predicted CREs tested in the present study are shown in flatmount along with previously characterized mouse *Rho*-CRE and *Nrl*-CRE as controls. Also shown are retinas electroporated with the ‘vector only’ controls: ‘Rho-basal’ and ‘No-basal’ (described in METHODS). For comparison purposes, all images were taken at the same exposure time (1/300 second) except *Nr2e3*-CRE (1/600 second) and all CREs categorized as ‘absent’ (1/150 second). The ‘vector only’ controls were also taken at (1/150 second). Images taken with longer exposures to highlight expression of ‘weak’ CREs are given in Table S1. Some of the retinas electroporated with constructs with ‘absent’ expression show faint, diffuse red fluorescence which represents autofluorescence in the lens. Size bar = 100 µm.
(9.61 MB TIF)

Figure S3 *Cis*-regulatory motif associated with high-level expression in photoreceptors. A-D, Images showing the distribution of Crx and Nrl sites (as described in Legend at the top of the figure) in four ‘very strong’ CREs. All four CREs show a simple cis-regulatory motif (summarized in E) which is boxed. E, Summary of novel *cis*-regulatory motif associated with high-level expression in photoreceptors. ‘Crx^ LOS^’ indicates the log odds score for the Crx site, a measure of its closeness to consensus. In addition to minimal log odds scores for the individual Crx and Nrl sites, this grammar rule requires that their combined log odds scores be ≥ 16.0 as indicated and that the two sites be <40 bp apart. F, Summary of percentage of novel CREs from each expression strength category which contain the cis-regulatory motif described in E.
(7.33 MB TIF)

Figure S4 Expression of synthetic constructs from evolutionary run 3. A-D, Images of retinas electroporated with the indicated constructs at P0, cultured as explants, and harvested at P8. Syn3-G55 is shown in Fig. 5 and is reproduced here for comparison. All four constructs derive from the ‘organisms’ indicated in Fig. 5E. The expression strengths of these four constructs over time are summarized in Fig. 5F. Details about ‘CRE architecture’ notation are given in Fig. 5. Size bar = 500 µm for flatmount images and 100 µm for cross-sections.
(6.18 MB TIF)

Table S1 Genes in the mouse photoreceptor transcription network. This table is a database which includes all genes dysregulated in *Crx^-/-^*, *Nrl^-/-^*, and/or *Nr2e3^-/-^* under high stringency criteria. Each row in the database represents a gene in the network and includes multiple links to additional types of information about that gene. ‘Large image gene +/− 15 Kb’ links to an image of our computational prediction of photoreceptor CREs in the genomic region 15 Kb upstream and downstream of the gene in question. ‘Closeup image TSS +/− 15 Kb’ links to an image of our computational prediction in the region 15 Kb on either side of the gene's TSS. Black bars within this image highlight the following regulatory peaks (if any are present): the peak closest to the TSS and the peak with the highest CRE score within this 30 Kb window. For those genes whose CRE predictions were tested experimentally, a red bar in this image indicates the location of the CRE that was tested. For *Crx* and *Elovl4* the tested CRE is outside of this 30 Kb window and is therefore indicated in the ‘Large image’. ‘Links’ contains links to the UCSC genome browser, ENSEMBL, and NCBI database entries for the indicated gene, if available. ‘Max score (threshold of 200)’ contains the score of the highest predicted regulatory peak within the 30 Kb window around the gene's TSS. If this window does not contain any predicted peaks ≥ 200 (our cutoff threshold) no value is given (indicated by a dash). The next four columns of the table (‘Nr2e3-/-’ etc.) show the wild-type-to-mutant ratios of the averaged microarray scores for the given gene. For those genes to which more than one Affy tag correspond, the data for the first one listed under ‘Affy Mouse 430 2.0’ is given. Dark green = downregulated under high stringency (as described in METHODS); light green = downregulated under low stringency; red = upregulated under high stringency; orange = upregulated under low stringency. A dash indicates that the gene was not significantly altered in the given mutant. ‘NA’ indicates that the gene is not represented on the Affymetrix Mouse 430 2.0 microarray. ‘In situ hybridization’ links to ISH images of the gene, if available. Alterations in expression level ≤ 2-fold on microarray are generally not detectable by ISH. ‘Electroporation’ links to images of retinas electroporated with the CRE indicated by the red bar in ‘Closeup image TSS +/− 15 Kb’, if available. All images were taken at the same exposure time (1/300 second) unless indicated in the image. ‘Averaged Affy scores’ includes the average score for the first Affy tag listed under ‘Affy Mouse 430 2.0’ from three independent microarray experiments in the given mutant or wild-type background. Each mutant is shown in association with the score for its respective wild-type background. The score for the ‘B6;129’ control for ‘*Crx^-/-^*, *Nrl^-/-^*’ is the average of the averaged B6 and 129 scores (as described in more detail in METHODS). ‘Locus’ indicates the chromosomal locus of the gene. ‘Affy Mouse 430 2.0’ contains all Affy tags corresponding to the gene in question which were dysregulated under high stringency criteria in any of the single mutants. An interactive version of this table is available at: http://www.plosone.org/suppinfo/pone.0000643/
(27.90 MB ZIP)

Table S2 Genes dysregulated in *Nr2e3^-/-^*, *Crx^-/-^*, *Nrl^-/-^*, and *Crx^-/-^*; *Nrl^-/-^* under high stringency criteria. This list contains eight sub-lists of genes which met high stringency criteria for up- or downregulation in *Nr2e3^-/-^*, *Crx^-/-^*, *Nrl^-/-^*, and *Crx^-/-^*; *Nrl^-/-^* retinas (described in METHODS). The data for *Nrl^-/-^* were published previously [25] and are reproduced here for convenience. The microarray datasets for *Nr2e3^-/-^* were also published previously [20], but this analysis is new. Each sub-list is subdivided into three parts by color coding (taking Nrl-downregulated genes as an example): light green: genes whose average raw microarray score in the given mutant retina (score^ Nrl^) is ≥5000; brown : 1000≤score^ Nrl^<5000; and pink: 100≤score^Nrl^<1000. "Nrl_ave" and "B6_ave" indicate the average scores for *Nrl* mutant and wild-type (C57BL/6) obtained for the indicated feature from three separate microarray experiments. Within each of the three sub-lists, genes are listed in order of their degree of change in the *Nrl* mutant retina (relative to wild-type) as indicated by the ratio of their average wt: *Nrl* ratios ("B6_ave/Nrl_ave"). The columns labeled "Additional Affy_IDs" contain additional Affymetrix microarray feature identifiers which correspond to the gene in question and which were also met our stringent criteria for upregulation in the *Nrl* mutant.
(0.43 MB XLS)

Table S3 Genes dysregulated in *Nr2e3^-/-^*, *Crx^-/-^*, *Nrl^-/-^*, and *Crx^-/-^*; *Nrl^-/-^* under low stringency criteria. This list contains eight sub-lists of genes which met low stringency criteria for up- or downregulation in *Nr2e3^-/-^*, *Crx^-/-^*, *Nrl^-/-^*, and *Crx^-/-^*; *Nrl^-/-^* retinas (described in METHODS). The data for *Nrl^-/-^* and *Nr2e3^-/-^* were published previously [20], [25], but this analysis is new and they are therefore included here for completeness. Each row corresponds to a single gene which is dysregulated in the given background. ‘Affy_ID’ is an Affymetrix feature identifier representative of the given gene. Taking ‘Nr2e3-downregulated genes as an example, ‘Nr2e3_ave’ and ‘B6_ave’ columns show average Affymetrix scores for the feature in the first column from three independent microarray experiments. ‘B6_ave/Nr2e3_ave’ is the ratio of the B6 to *Nr2e3^-/-^* ratios. Within each sub-list, genes are listed in order of their degree of change in the mutant retina (relative to wild-type) as indicated by the ratio of their average wild type-to-mutant ratios. ‘Additional Affy_IDs’ show additional Affymetrix tags which correspond to the gene question in those cases where there is more than one.
(2.19 MB XLS)

Table S4 Complete averaged microarray datasets for *Nr2e3^-/-^*, *Crx^-/-^*, *Nrl^-/-^*, and *Crx^-/-^*; *Nrl^-/-^*. This table contains average scores for all four mutants compared to their respective wild-types for all 45,037 Affymetrix feature identifiers. The data for *Nrl^-/-^* and *Nr2e3^-/-^* were presented previously [20], [25] and are reproduced here for comparison. The descriptors for the columns are as described in Table S3.
(18.01 MB XLS)

Table S5 Complete microarray datasets for wild-type vs. *Crx^-/-^* comparison at P21. This table contains the full microarray datasets from six microarray hybridizations (three biological replicates each from wild-type (129S6/SvEv) and *Crx^-/-^* retinas at P21). The data from each of the individual microarray experiments are labeled with the following prefixes: ‘Crx-mut_#1’ etc. and ‘129-wt-P21-#1’ etc.
(26.17 MB XLS)

Table S6 Complete microarray datasets for wild-type vs. *Crx^-/-^*; *Nrl^-/-^* comparison at P21. This table contains the full microarray datasets from nine microarray hybridizations (three biological replicates each from wild-type (C57BL/6), wild-type (129S6/SvEv) and *Crx^-/-^*; Nrl^-/-^ retinas at P21). Since *Crx^-/-^*; *Nrl^-/-^* is on a mixed 129 X B6 background, we present comparisons between both of the individual wild-type mutant backgrounds (as data from the mixed control background were not available). The data from each of the individual microarray experiments are labeled with the following prefixes: ‘Crx-mut_#1’ etc., ‘B6-wt-P21-#1’ etc. and ‘129-wt-P21-#1’ etc.
(39.54 MB XLS)

Table S7 Intersection sets from Venn diagram analysis of photoreceptor network. This table includes all possible intersections of sets of genes dysregulated in more than one of the three single mutants: *Crx^-/-^*, *Nrl^-/-^*, and *Nr2e3^-/-^*. For example, ‘Crx-down ∩ Nrl-down’ includes all genes which were downregulated in both *Crx^-/-^* and *Nrl^-/-^* under high stringency criteria. The set ‘Nrl-down ∩ Nrl-up’ includes one gene, *Rs1h*, which has two corresponding Affy tags. One of these tags in downregulated in *Nrl^-/-^*, the other upregulated. These two tags possibly represent variant transcriptional isoforms which are controlled differently by Nrl.
(0.03 MB XLS)

Table S8 Transcription factors downstream of *Crx*, *Nrl*, and *Nr2e3*. List of genes with Gene Ontology classification ‘transcription factor activity’ derived from an analysis of the 628 genes shown to be dysregulated in *Crx^-/-^*, *Nrl^-/-^*, and/or *Nr2e3^-/-^* under high stringency criteria. Those genes highlighted in light green are thought to be sequence-specific TFs.
(0.03 MB XLS)

Table S9 Sequences of novel photoreceptor *cis*-regulatory elements. This table includes the full DNA sequences of the 26 novel photoreceptor CREs characterized in this study along with the sequences of the PCR primers used to obtain them. The sequences of the mouse *Rho*-CRE as well as the control_ *Rgs9*-CRE are also included. Restriction enzyme sites included in the PCR primers for purposes of cloning are in upper case. Details about the starting vectors and subcloning procedure are given in Supplementary Methods.
(0.04 MB XLS)

Table S10 Complete datasets for three *in silico* evolutionary runs. This spreadsheet includes three separate tabs corresponding to the three independent evolutionary runs: Syn1, Syn2, and Syn3. Each tab includes 1000 rows corresponding to the generations of the experiment. For each generation the ‘fitness’ score and genome sequence of the fittest ‘organism’ from that generation are given. The organisms corresponding to the six synthetic CREs assayed (i.e. Syn1-G70 etc.) are highlighted in light green.
(0.97 MB XLS)
