# Supplementary figures and images for: The Cis-regulatory Logic of the Mammalian Photoreceptor Transcriptional Network (part 1 of 3)
Source: PLoS One. 2007 Jul 25;2(7):e643. doi: 10.1371/journal.pone.0000643 (PMC1916400; doi:10.1371/journal.pone.0000643)

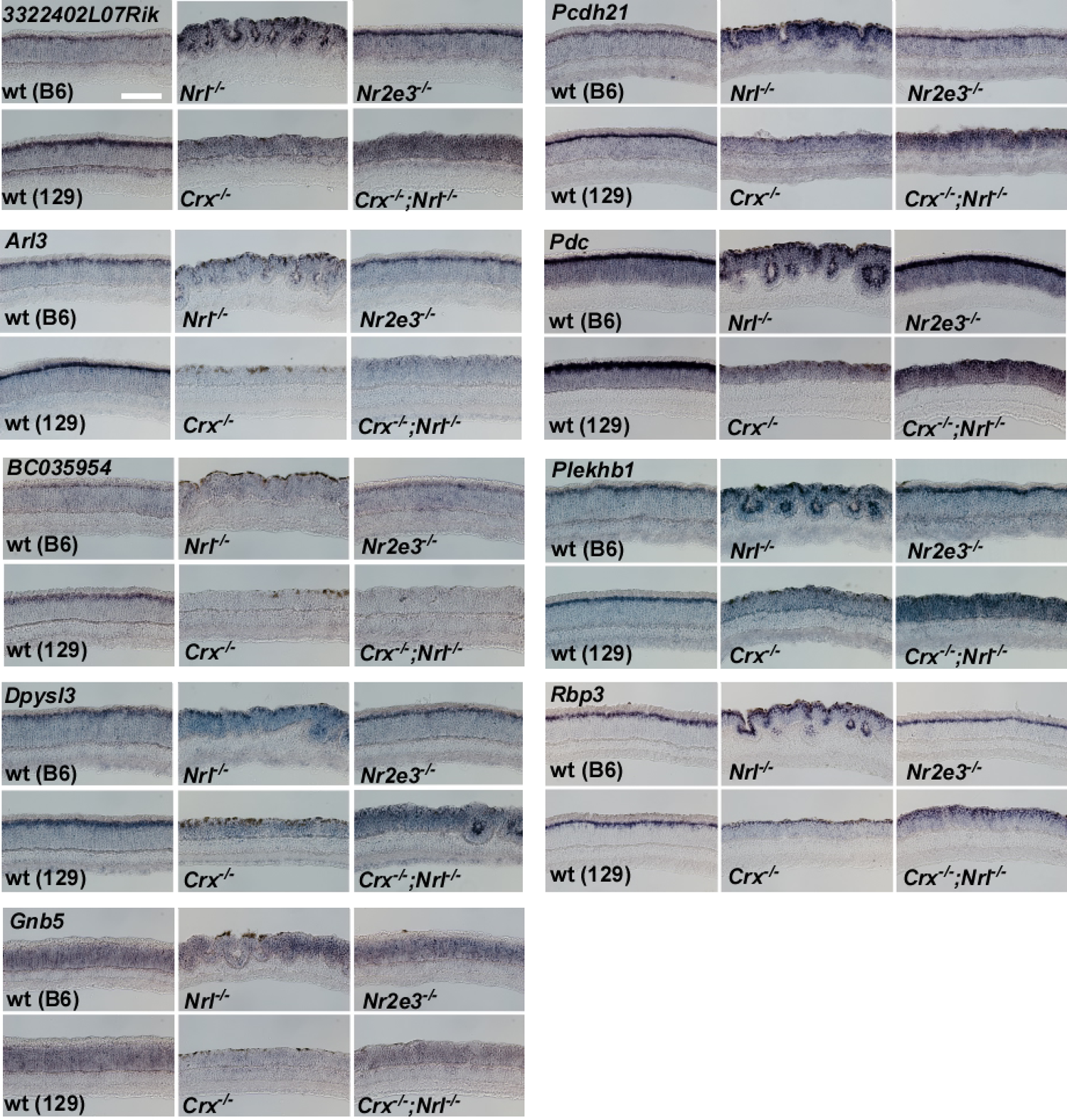

Supplement: Figure S1 — In situ hybridizations for genes not in Table S1. ISH images on four mutant and two wild-type backgrounds for nine photoreceptor-enriched genes which were not dysregulated in Crx-/-, Nrl-/-, or Nr2e3-/- and therefore not included in Table S1. Size bar = 100 µm. (7.10 MB TIF) [file pone.0000643.s001.tif]

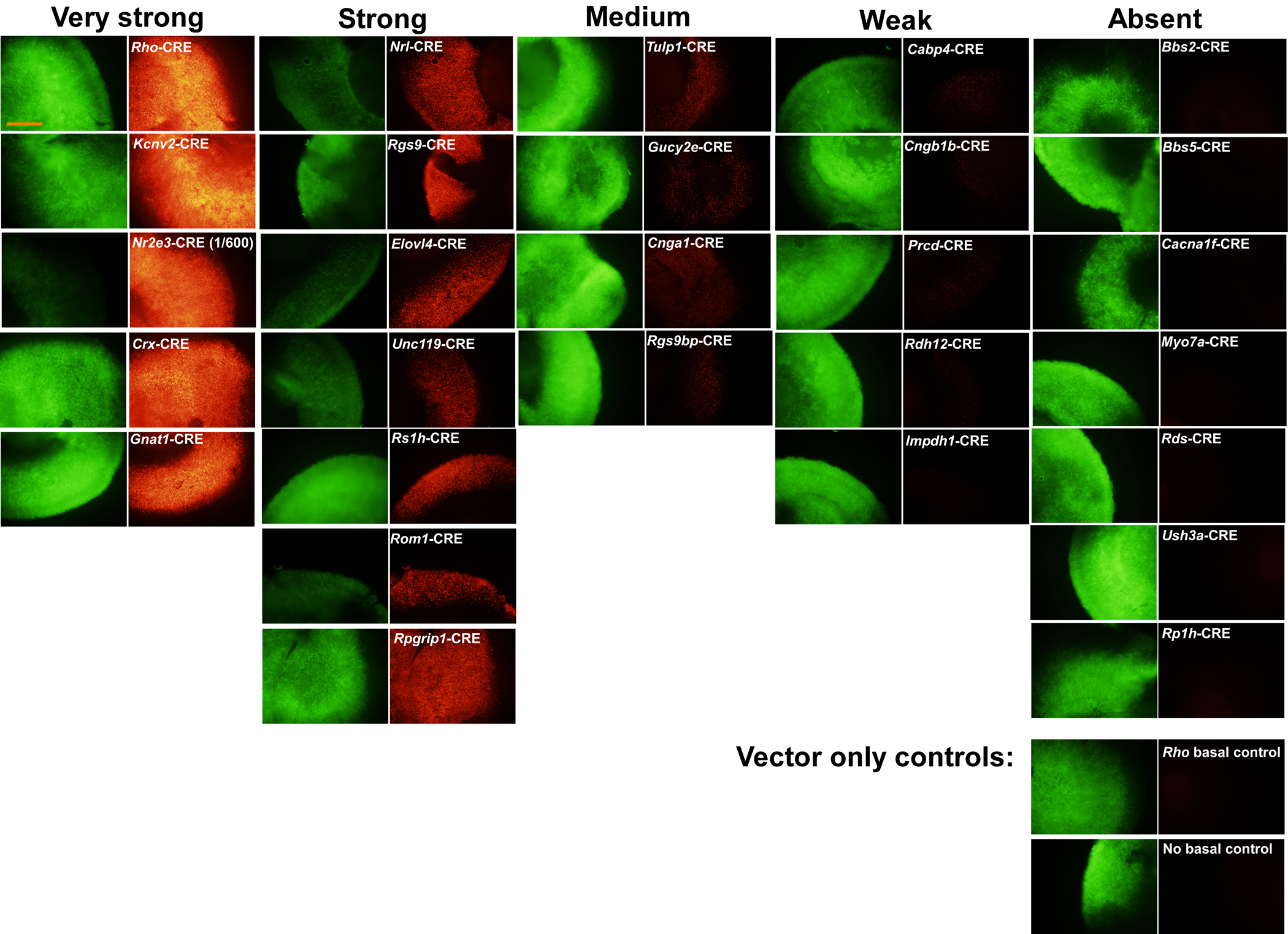

Supplement: Figure S2 — Expression strength categorization of novel photoreceptor cis-regulatory elements. Images of retinas electroporated with the indicated constructs at P0, cultured as explants, and harvested at P8. All 26 predicted CREs tested in the present study are shown in flatmount along with previously characterized mouse Rho-CRE and Nrl-CRE as controls. Also shown are retinas electroporated with the ‘vector only’ controls: ‘Rho-basal’ and ‘No-basal’ (described in METHODS). For comparison purposes, all images were taken at the same exposure time (1/300 second) except Nr2e3-CRE (1/600 second) and all CREs categorized as ‘absent’ (1/150 second). The ‘vector only’ controls were also taken at (1/150 second). Images taken with longer exposures to highlight expression of ‘weak’ CREs are given in Table S1. Some of the retinas electroporated with constructs with ‘absent’ expression show faint, diffuse red fluorescence which represents autofluorescence in the lens. Size bar = 100 µm. (9.61 MB TIF) [file pone.0000643.s002.tif]

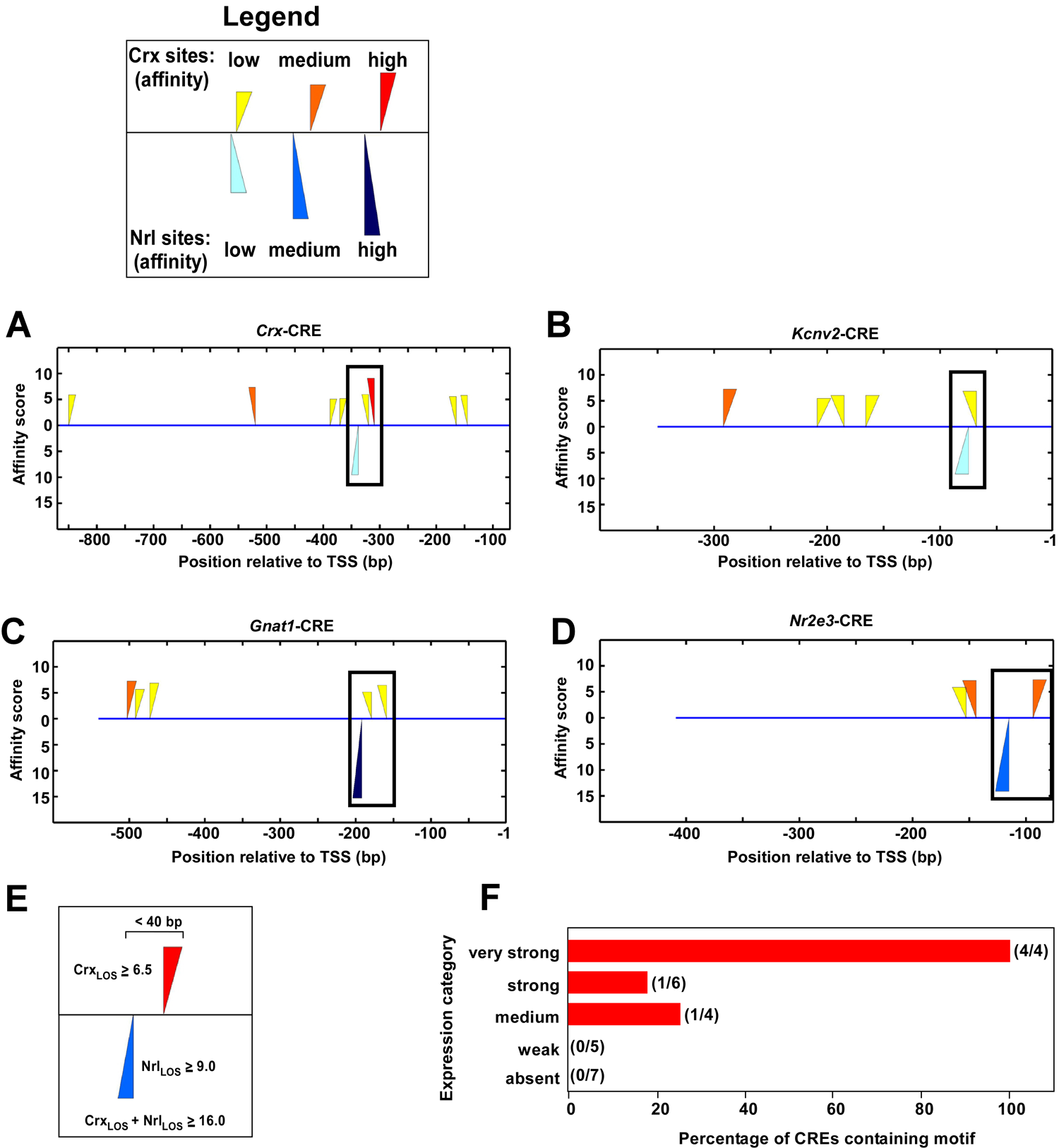

Supplement: Figure S3 — Cis-regulatory motif associated with high-level expression in photoreceptors. A-D, Images showing the distribution of Crx and Nrl sites (as described in Legend at the top of the figure) in four ‘very strong’ CREs. All four CREs show a simple cis-regulatory motif (summarized in E) which is boxed. E, Summary of novel cis-regulatory motif associated with high-level expression in photoreceptors. ‘Crx LOS’ indicates the log odds score for the Crx site, a measure of its closeness to consensus. In addition to minimal log odds scores for the individual Crx and Nrl sites, this grammar rule requires that their combined log odds scores be ≥ 16.0 as indicated and that the two sites be <40 bp apart. F, Summary of percentage of novel CREs from each expression strength category which contain the cis-regulatory motif described in E. (7.33 MB TIF) [file pone.0000643.s003.tif]

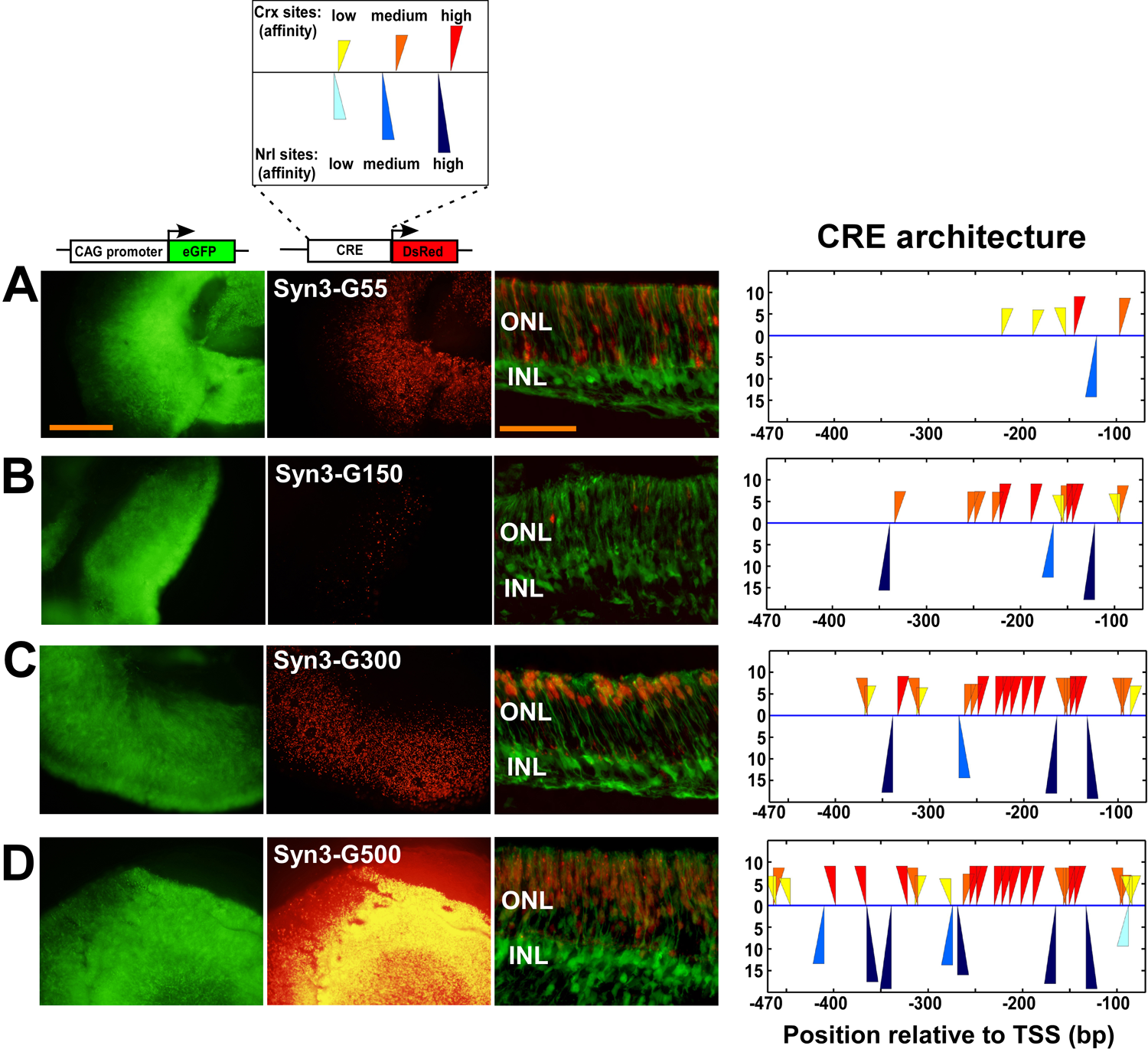

Supplement: Figure S4 — Expression of synthetic constructs from evolutionary run 3. A-D, Images of retinas electroporated with the indicated constructs at P0, cultured as explants, and harvested at P8. Syn3-G55 is shown in Fig. 5 and is reproduced here for comparison. All four constructs derive from the ‘organisms’ indicated in Fig. 5E. The expression strengths of these four constructs over time are summarized in Fig. 5F. Details about ‘CRE architecture’ notation are given in Fig. 5. Size bar = 500 µm for flatmount images and 100 µm for cross-sections. (6.18 MB TIF) [file pone.0000643.s004.tif]

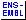

Supplement: Table S1 — Genes in the mouse photoreceptor transcription network. This table is a database which includes all genes dysregulated in Crx-/-, Nrl-/-, and/or Nr2e3-/- under high stringency criteria. Each row in the database represents a gene in the network and includes multiple links to additional types of information about that gene. ‘Large image gene +/− 15 Kb’ links to an image of our computational prediction of photoreceptor CREs in the genomic region 15 Kb upstream and downstream of the gene in question. ‘Closeup image TSS +/− 15 Kb’ links to an image of our computational prediction in the region 15 Kb on either side of the gene's TSS. Black bars within this image highlight the following regulatory peaks (if any are present): the peak closest to the TSS and the peak with the highest CRE score within this 30 Kb window. For those genes whose CRE predictions were tested experimentally, a red bar in this image indicates the location of the CRE that was tested. For Crx and Elovl4 the tested CRE is outside of this 30 Kb window and is therefore indicated in the ‘Large image’. ‘Links’ contains links to the UCSC genome browser, ENSEMBL, and NCBI database entries for the indicated gene, if available. ‘Max score (threshold of 200)’ contains the score of the highest predicted regulatory peak within the 30 Kb window around the gene's TSS. If this window does not contain any predicted peaks ≥ 200 (our cutoff threshold) no value is given (indicated by a dash). The next four columns of the table (‘Nr2e3-/-’ etc.) show the wild-type-to-mutant ratios of the averaged microarray scores for the given gene. For those genes to which more than one Affy tag correspond, the data for the first one listed under ‘Affy Mouse 430 2.0’ is given. Dark green = downregulated under high stringency (as described in METHODS); light green = downregulated under low stringency; red = upregulated under high stringency; orange = upregulated under low stringency. A dash indicates that the gene was not significantly a [file pone.0000643.s005.zip › Corbo_TableS1/ensembl.GIF]

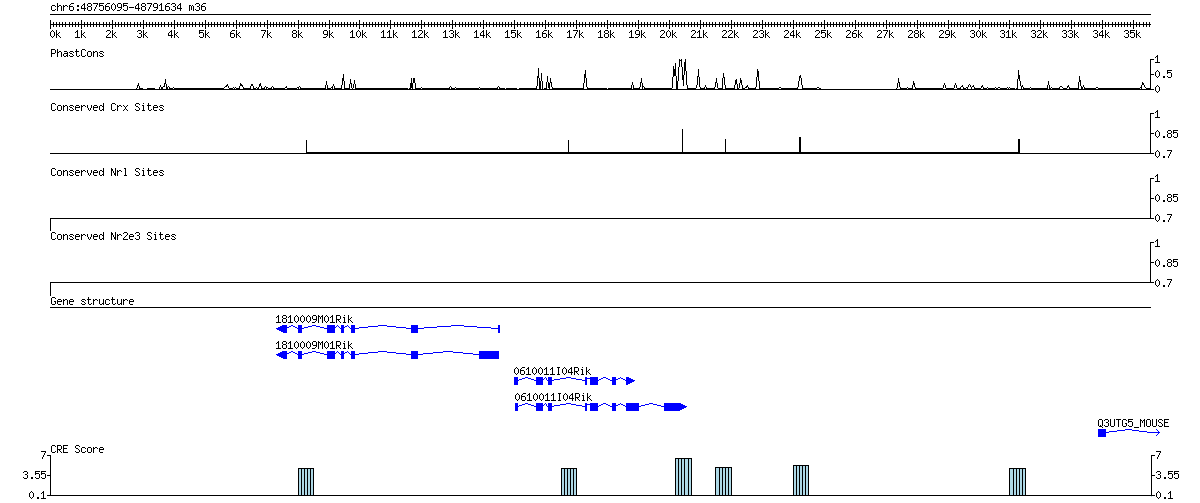

Supplement: Table S1 — Genes in the mouse photoreceptor transcription network. This table is a database which includes all genes dysregulated in Crx-/-, Nrl-/-, and/or Nr2e3-/- under high stringency criteria. Each row in the database represents a gene in the network and includes multiple links to additional types of information about that gene. ‘Large image gene +/− 15 Kb’ links to an image of our computational prediction of photoreceptor CREs in the genomic region 15 Kb upstream and downstream of the gene in question. ‘Closeup image TSS +/− 15 Kb’ links to an image of our computational prediction in the region 15 Kb on either side of the gene's TSS. Black bars within this image highlight the following regulatory peaks (if any are present): the peak closest to the TSS and the peak with the highest CRE score within this 30 Kb window. For those genes whose CRE predictions were tested experimentally, a red bar in this image indicates the location of the CRE that was tested. For Crx and Elovl4 the tested CRE is outside of this 30 Kb window and is therefore indicated in the ‘Large image’. ‘Links’ contains links to the UCSC genome browser, ENSEMBL, and NCBI database entries for the indicated gene, if available. ‘Max score (threshold of 200)’ contains the score of the highest predicted regulatory peak within the 30 Kb window around the gene's TSS. If this window does not contain any predicted peaks ≥ 200 (our cutoff threshold) no value is given (indicated by a dash). The next four columns of the table (‘Nr2e3-/-’ etc.) show the wild-type-to-mutant ratios of the averaged microarray scores for the given gene. For those genes to which more than one Affy tag correspond, the data for the first one listed under ‘Affy Mouse 430 2.0’ is given. Dark green = downregulated under high stringency (as described in METHODS); light green = downregulated under low stringency; red = upregulated under high stringency; orange = upregulated under low stringency. A dash indicates that the gene was not significantly a [file pone.0000643.s005.zip › Corbo_TableS1/images/0610011I04Rik.gif]

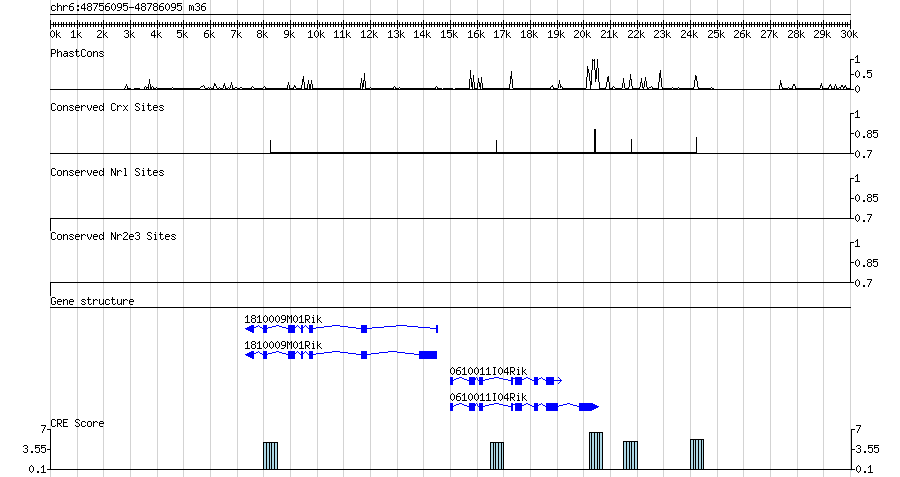

Supplement: Table S1 — Genes in the mouse photoreceptor transcription network. This table is a database which includes all genes dysregulated in Crx-/-, Nrl-/-, and/or Nr2e3-/- under high stringency criteria. Each row in the database represents a gene in the network and includes multiple links to additional types of information about that gene. ‘Large image gene +/− 15 Kb’ links to an image of our computational prediction of photoreceptor CREs in the genomic region 15 Kb upstream and downstream of the gene in question. ‘Closeup image TSS +/− 15 Kb’ links to an image of our computational prediction in the region 15 Kb on either side of the gene's TSS. Black bars within this image highlight the following regulatory peaks (if any are present): the peak closest to the TSS and the peak with the highest CRE score within this 30 Kb window. For those genes whose CRE predictions were tested experimentally, a red bar in this image indicates the location of the CRE that was tested. For Crx and Elovl4 the tested CRE is outside of this 30 Kb window and is therefore indicated in the ‘Large image’. ‘Links’ contains links to the UCSC genome browser, ENSEMBL, and NCBI database entries for the indicated gene, if available. ‘Max score (threshold of 200)’ contains the score of the highest predicted regulatory peak within the 30 Kb window around the gene's TSS. If this window does not contain any predicted peaks ≥ 200 (our cutoff threshold) no value is given (indicated by a dash). The next four columns of the table (‘Nr2e3-/-’ etc.) show the wild-type-to-mutant ratios of the averaged microarray scores for the given gene. For those genes to which more than one Affy tag correspond, the data for the first one listed under ‘Affy Mouse 430 2.0’ is given. Dark green = downregulated under high stringency (as described in METHODS); light green = downregulated under low stringency; red = upregulated under high stringency; orange = upregulated under low stringency. A dash indicates that the gene was not significantly a [file pone.0000643.s005.zip › Corbo_TableS1/images/0610011I04Rik_TSS.gif]

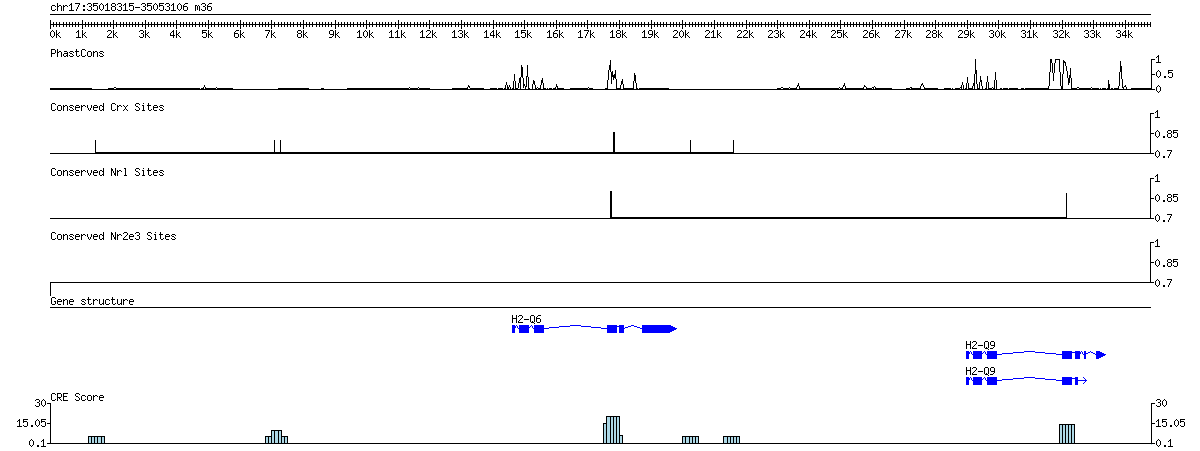

Supplement: Table S1 — Genes in the mouse photoreceptor transcription network. This table is a database which includes all genes dysregulated in Crx-/-, Nrl-/-, and/or Nr2e3-/- under high stringency criteria. Each row in the database represents a gene in the network and includes multiple links to additional types of information about that gene. ‘Large image gene +/− 15 Kb’ links to an image of our computational prediction of photoreceptor CREs in the genomic region 15 Kb upstream and downstream of the gene in question. ‘Closeup image TSS +/− 15 Kb’ links to an image of our computational prediction in the region 15 Kb on either side of the gene's TSS. Black bars within this image highlight the following regulatory peaks (if any are present): the peak closest to the TSS and the peak with the highest CRE score within this 30 Kb window. For those genes whose CRE predictions were tested experimentally, a red bar in this image indicates the location of the CRE that was tested. For Crx and Elovl4 the tested CRE is outside of this 30 Kb window and is therefore indicated in the ‘Large image’. ‘Links’ contains links to the UCSC genome browser, ENSEMBL, and NCBI database entries for the indicated gene, if available. ‘Max score (threshold of 200)’ contains the score of the highest predicted regulatory peak within the 30 Kb window around the gene's TSS. If this window does not contain any predicted peaks ≥ 200 (our cutoff threshold) no value is given (indicated by a dash). The next four columns of the table (‘Nr2e3-/-’ etc.) show the wild-type-to-mutant ratios of the averaged microarray scores for the given gene. For those genes to which more than one Affy tag correspond, the data for the first one listed under ‘Affy Mouse 430 2.0’ is given. Dark green = downregulated under high stringency (as described in METHODS); light green = downregulated under low stringency; red = upregulated under high stringency; orange = upregulated under low stringency. A dash indicates that the gene was not significantly a [file pone.0000643.s005.zip › Corbo_TableS1/images/0610037M15Rik.gif]

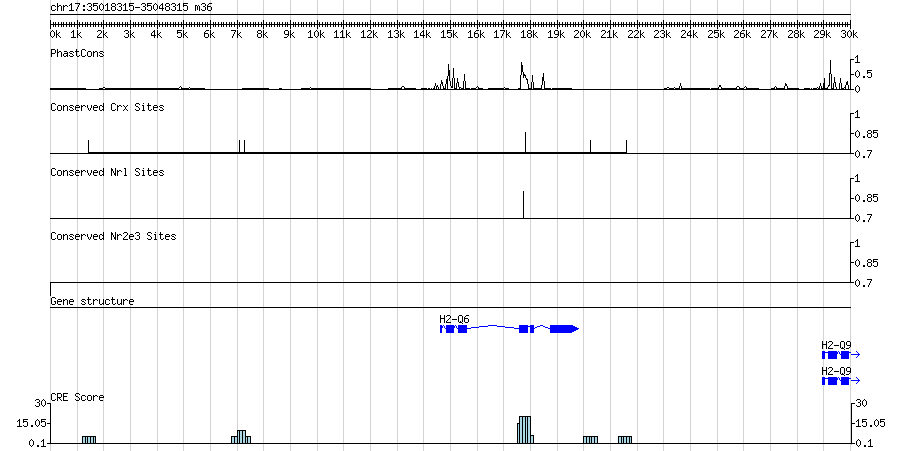

Supplement: Table S1 — Genes in the mouse photoreceptor transcription network. This table is a database which includes all genes dysregulated in Crx-/-, Nrl-/-, and/or Nr2e3-/- under high stringency criteria. Each row in the database represents a gene in the network and includes multiple links to additional types of information about that gene. ‘Large image gene +/− 15 Kb’ links to an image of our computational prediction of photoreceptor CREs in the genomic region 15 Kb upstream and downstream of the gene in question. ‘Closeup image TSS +/− 15 Kb’ links to an image of our computational prediction in the region 15 Kb on either side of the gene's TSS. Black bars within this image highlight the following regulatory peaks (if any are present): the peak closest to the TSS and the peak with the highest CRE score within this 30 Kb window. For those genes whose CRE predictions were tested experimentally, a red bar in this image indicates the location of the CRE that was tested. For Crx and Elovl4 the tested CRE is outside of this 30 Kb window and is therefore indicated in the ‘Large image’. ‘Links’ contains links to the UCSC genome browser, ENSEMBL, and NCBI database entries for the indicated gene, if available. ‘Max score (threshold of 200)’ contains the score of the highest predicted regulatory peak within the 30 Kb window around the gene's TSS. If this window does not contain any predicted peaks ≥ 200 (our cutoff threshold) no value is given (indicated by a dash). The next four columns of the table (‘Nr2e3-/-’ etc.) show the wild-type-to-mutant ratios of the averaged microarray scores for the given gene. For those genes to which more than one Affy tag correspond, the data for the first one listed under ‘Affy Mouse 430 2.0’ is given. Dark green = downregulated under high stringency (as described in METHODS); light green = downregulated under low stringency; red = upregulated under high stringency; orange = upregulated under low stringency. A dash indicates that the gene was not significantly a [file pone.0000643.s005.zip › Corbo_TableS1/images/0610037M15Rik_TSS.gif]

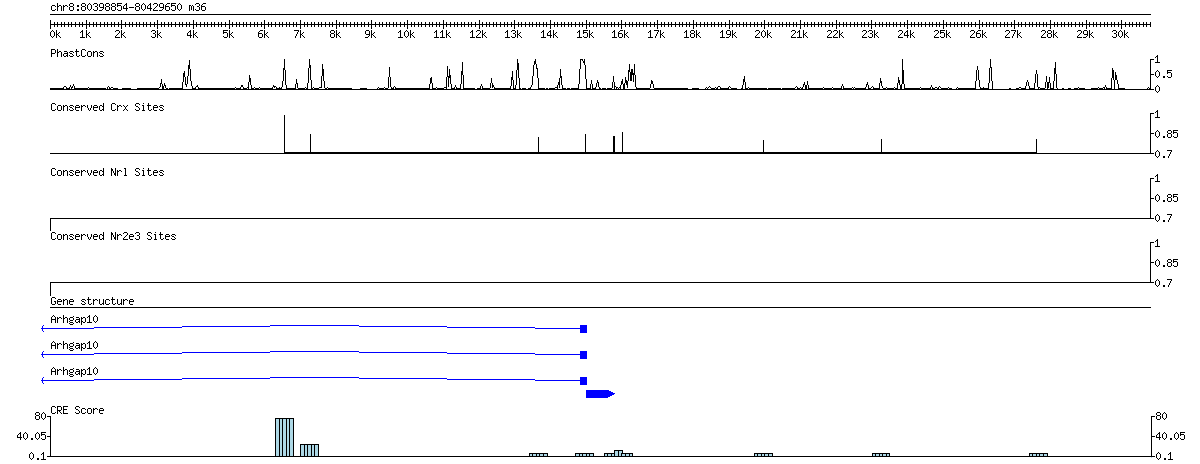

Supplement: Table S1 — Genes in the mouse photoreceptor transcription network. This table is a database which includes all genes dysregulated in Crx-/-, Nrl-/-, and/or Nr2e3-/- under high stringency criteria. Each row in the database represents a gene in the network and includes multiple links to additional types of information about that gene. ‘Large image gene +/− 15 Kb’ links to an image of our computational prediction of photoreceptor CREs in the genomic region 15 Kb upstream and downstream of the gene in question. ‘Closeup image TSS +/− 15 Kb’ links to an image of our computational prediction in the region 15 Kb on either side of the gene's TSS. Black bars within this image highlight the following regulatory peaks (if any are present): the peak closest to the TSS and the peak with the highest CRE score within this 30 Kb window. For those genes whose CRE predictions were tested experimentally, a red bar in this image indicates the location of the CRE that was tested. For Crx and Elovl4 the tested CRE is outside of this 30 Kb window and is therefore indicated in the ‘Large image’. ‘Links’ contains links to the UCSC genome browser, ENSEMBL, and NCBI database entries for the indicated gene, if available. ‘Max score (threshold of 200)’ contains the score of the highest predicted regulatory peak within the 30 Kb window around the gene's TSS. If this window does not contain any predicted peaks ≥ 200 (our cutoff threshold) no value is given (indicated by a dash). The next four columns of the table (‘Nr2e3-/-’ etc.) show the wild-type-to-mutant ratios of the averaged microarray scores for the given gene. For those genes to which more than one Affy tag correspond, the data for the first one listed under ‘Affy Mouse 430 2.0’ is given. Dark green = downregulated under high stringency (as described in METHODS); light green = downregulated under low stringency; red = upregulated under high stringency; orange = upregulated under low stringency. A dash indicates that the gene was not significantly a [file pone.0000643.s005.zip › Corbo_TableS1/images/0610038B21Rik.gif]

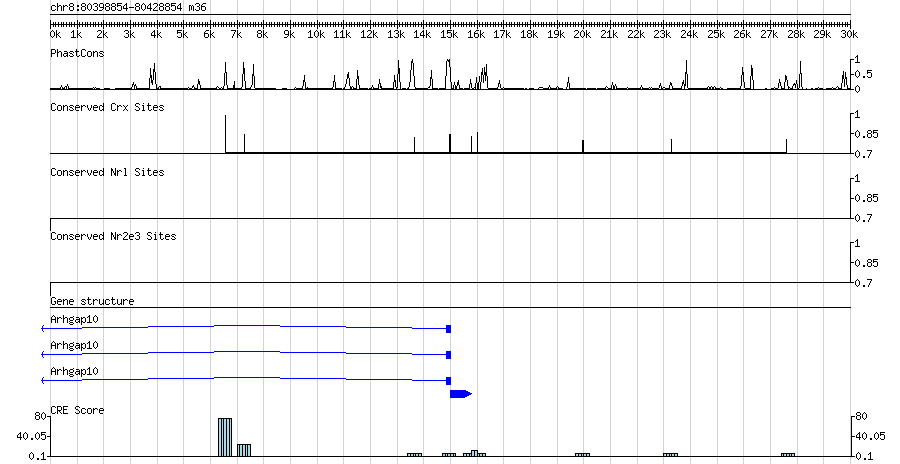

Supplement: Table S1 — Genes in the mouse photoreceptor transcription network. This table is a database which includes all genes dysregulated in Crx-/-, Nrl-/-, and/or Nr2e3-/- under high stringency criteria. Each row in the database represents a gene in the network and includes multiple links to additional types of information about that gene. ‘Large image gene +/− 15 Kb’ links to an image of our computational prediction of photoreceptor CREs in the genomic region 15 Kb upstream and downstream of the gene in question. ‘Closeup image TSS +/− 15 Kb’ links to an image of our computational prediction in the region 15 Kb on either side of the gene's TSS. Black bars within this image highlight the following regulatory peaks (if any are present): the peak closest to the TSS and the peak with the highest CRE score within this 30 Kb window. For those genes whose CRE predictions were tested experimentally, a red bar in this image indicates the location of the CRE that was tested. For Crx and Elovl4 the tested CRE is outside of this 30 Kb window and is therefore indicated in the ‘Large image’. ‘Links’ contains links to the UCSC genome browser, ENSEMBL, and NCBI database entries for the indicated gene, if available. ‘Max score (threshold of 200)’ contains the score of the highest predicted regulatory peak within the 30 Kb window around the gene's TSS. If this window does not contain any predicted peaks ≥ 200 (our cutoff threshold) no value is given (indicated by a dash). The next four columns of the table (‘Nr2e3-/-’ etc.) show the wild-type-to-mutant ratios of the averaged microarray scores for the given gene. For those genes to which more than one Affy tag correspond, the data for the first one listed under ‘Affy Mouse 430 2.0’ is given. Dark green = downregulated under high stringency (as described in METHODS); light green = downregulated under low stringency; red = upregulated under high stringency; orange = upregulated under low stringency. A dash indicates that the gene was not significantly a [file pone.0000643.s005.zip › Corbo_TableS1/images/0610038B21Rik_TSS.gif]

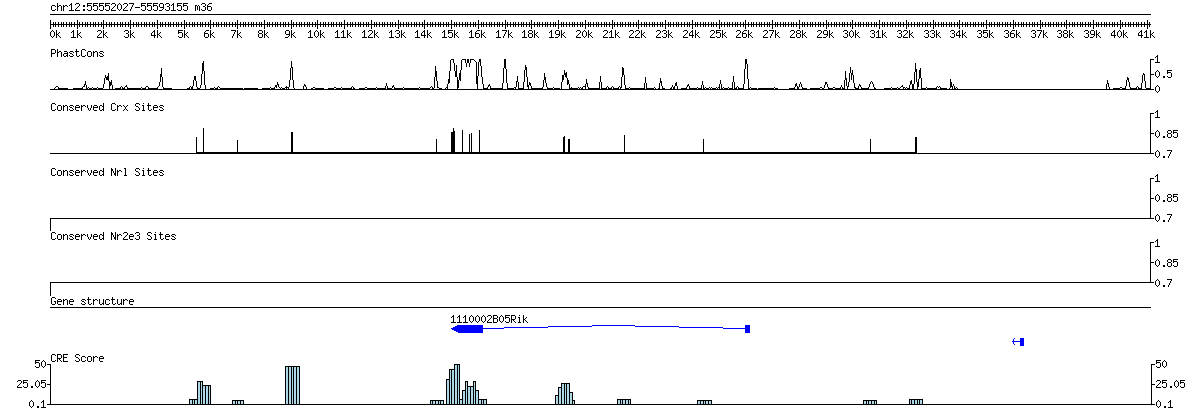

Supplement: Table S1 — Genes in the mouse photoreceptor transcription network. This table is a database which includes all genes dysregulated in Crx-/-, Nrl-/-, and/or Nr2e3-/- under high stringency criteria. Each row in the database represents a gene in the network and includes multiple links to additional types of information about that gene. ‘Large image gene +/− 15 Kb’ links to an image of our computational prediction of photoreceptor CREs in the genomic region 15 Kb upstream and downstream of the gene in question. ‘Closeup image TSS +/− 15 Kb’ links to an image of our computational prediction in the region 15 Kb on either side of the gene's TSS. Black bars within this image highlight the following regulatory peaks (if any are present): the peak closest to the TSS and the peak with the highest CRE score within this 30 Kb window. For those genes whose CRE predictions were tested experimentally, a red bar in this image indicates the location of the CRE that was tested. For Crx and Elovl4 the tested CRE is outside of this 30 Kb window and is therefore indicated in the ‘Large image’. ‘Links’ contains links to the UCSC genome browser, ENSEMBL, and NCBI database entries for the indicated gene, if available. ‘Max score (threshold of 200)’ contains the score of the highest predicted regulatory peak within the 30 Kb window around the gene's TSS. If this window does not contain any predicted peaks ≥ 200 (our cutoff threshold) no value is given (indicated by a dash). The next four columns of the table (‘Nr2e3-/-’ etc.) show the wild-type-to-mutant ratios of the averaged microarray scores for the given gene. For those genes to which more than one Affy tag correspond, the data for the first one listed under ‘Affy Mouse 430 2.0’ is given. Dark green = downregulated under high stringency (as described in METHODS); light green = downregulated under low stringency; red = upregulated under high stringency; orange = upregulated under low stringency. A dash indicates that the gene was not significantly a [file pone.0000643.s005.zip › Corbo_TableS1/images/1110002B05Rik.gif]

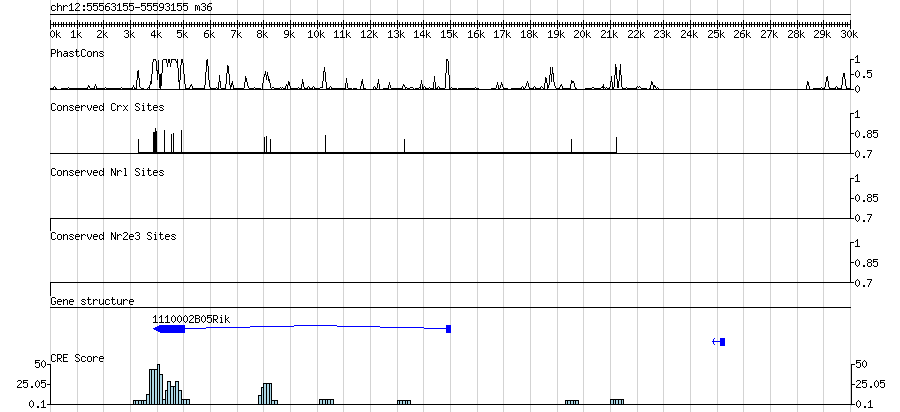

Supplement: Table S1 — Genes in the mouse photoreceptor transcription network. This table is a database which includes all genes dysregulated in Crx-/-, Nrl-/-, and/or Nr2e3-/- under high stringency criteria. Each row in the database represents a gene in the network and includes multiple links to additional types of information about that gene. ‘Large image gene +/− 15 Kb’ links to an image of our computational prediction of photoreceptor CREs in the genomic region 15 Kb upstream and downstream of the gene in question. ‘Closeup image TSS +/− 15 Kb’ links to an image of our computational prediction in the region 15 Kb on either side of the gene's TSS. Black bars within this image highlight the following regulatory peaks (if any are present): the peak closest to the TSS and the peak with the highest CRE score within this 30 Kb window. For those genes whose CRE predictions were tested experimentally, a red bar in this image indicates the location of the CRE that was tested. For Crx and Elovl4 the tested CRE is outside of this 30 Kb window and is therefore indicated in the ‘Large image’. ‘Links’ contains links to the UCSC genome browser, ENSEMBL, and NCBI database entries for the indicated gene, if available. ‘Max score (threshold of 200)’ contains the score of the highest predicted regulatory peak within the 30 Kb window around the gene's TSS. If this window does not contain any predicted peaks ≥ 200 (our cutoff threshold) no value is given (indicated by a dash). The next four columns of the table (‘Nr2e3-/-’ etc.) show the wild-type-to-mutant ratios of the averaged microarray scores for the given gene. For those genes to which more than one Affy tag correspond, the data for the first one listed under ‘Affy Mouse 430 2.0’ is given. Dark green = downregulated under high stringency (as described in METHODS); light green = downregulated under low stringency; red = upregulated under high stringency; orange = upregulated under low stringency. A dash indicates that the gene was not significantly a [file pone.0000643.s005.zip › Corbo_TableS1/images/1110002B05Rik_TSS.gif]

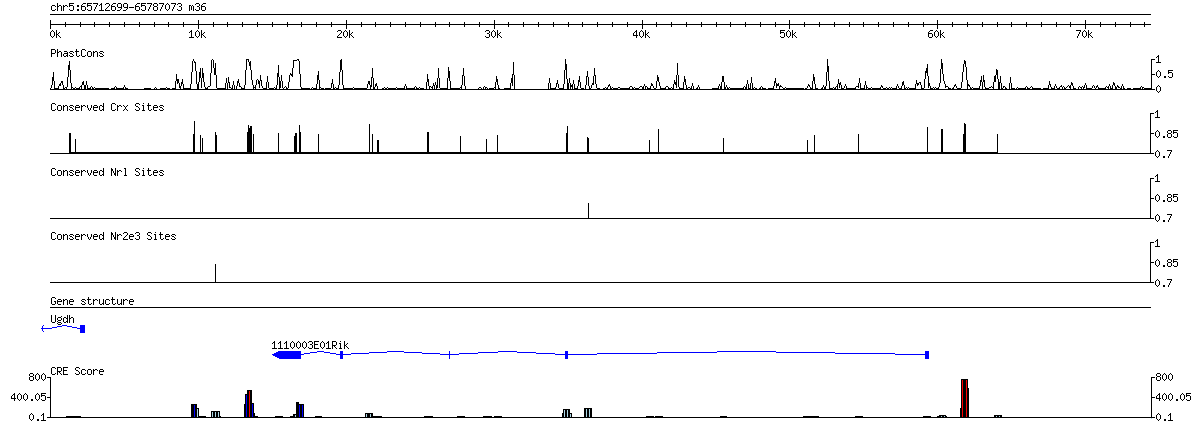

Supplement: Table S1 — Genes in the mouse photoreceptor transcription network. This table is a database which includes all genes dysregulated in Crx-/-, Nrl-/-, and/or Nr2e3-/- under high stringency criteria. Each row in the database represents a gene in the network and includes multiple links to additional types of information about that gene. ‘Large image gene +/− 15 Kb’ links to an image of our computational prediction of photoreceptor CREs in the genomic region 15 Kb upstream and downstream of the gene in question. ‘Closeup image TSS +/− 15 Kb’ links to an image of our computational prediction in the region 15 Kb on either side of the gene's TSS. Black bars within this image highlight the following regulatory peaks (if any are present): the peak closest to the TSS and the peak with the highest CRE score within this 30 Kb window. For those genes whose CRE predictions were tested experimentally, a red bar in this image indicates the location of the CRE that was tested. For Crx and Elovl4 the tested CRE is outside of this 30 Kb window and is therefore indicated in the ‘Large image’. ‘Links’ contains links to the UCSC genome browser, ENSEMBL, and NCBI database entries for the indicated gene, if available. ‘Max score (threshold of 200)’ contains the score of the highest predicted regulatory peak within the 30 Kb window around the gene's TSS. If this window does not contain any predicted peaks ≥ 200 (our cutoff threshold) no value is given (indicated by a dash). The next four columns of the table (‘Nr2e3-/-’ etc.) show the wild-type-to-mutant ratios of the averaged microarray scores for the given gene. For those genes to which more than one Affy tag correspond, the data for the first one listed under ‘Affy Mouse 430 2.0’ is given. Dark green = downregulated under high stringency (as described in METHODS); light green = downregulated under low stringency; red = upregulated under high stringency; orange = upregulated under low stringency. A dash indicates that the gene was not significantly a [file pone.0000643.s005.zip › Corbo_TableS1/images/1110003E01Rik.gif]

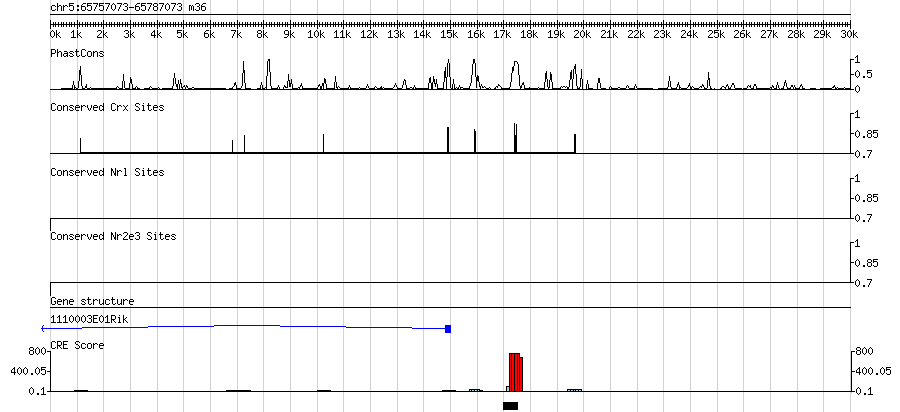

Supplement: Table S1 — Genes in the mouse photoreceptor transcription network. This table is a database which includes all genes dysregulated in Crx-/-, Nrl-/-, and/or Nr2e3-/- under high stringency criteria. Each row in the database represents a gene in the network and includes multiple links to additional types of information about that gene. ‘Large image gene +/− 15 Kb’ links to an image of our computational prediction of photoreceptor CREs in the genomic region 15 Kb upstream and downstream of the gene in question. ‘Closeup image TSS +/− 15 Kb’ links to an image of our computational prediction in the region 15 Kb on either side of the gene's TSS. Black bars within this image highlight the following regulatory peaks (if any are present): the peak closest to the TSS and the peak with the highest CRE score within this 30 Kb window. For those genes whose CRE predictions were tested experimentally, a red bar in this image indicates the location of the CRE that was tested. For Crx and Elovl4 the tested CRE is outside of this 30 Kb window and is therefore indicated in the ‘Large image’. ‘Links’ contains links to the UCSC genome browser, ENSEMBL, and NCBI database entries for the indicated gene, if available. ‘Max score (threshold of 200)’ contains the score of the highest predicted regulatory peak within the 30 Kb window around the gene's TSS. If this window does not contain any predicted peaks ≥ 200 (our cutoff threshold) no value is given (indicated by a dash). The next four columns of the table (‘Nr2e3-/-’ etc.) show the wild-type-to-mutant ratios of the averaged microarray scores for the given gene. For those genes to which more than one Affy tag correspond, the data for the first one listed under ‘Affy Mouse 430 2.0’ is given. Dark green = downregulated under high stringency (as described in METHODS); light green = downregulated under low stringency; red = upregulated under high stringency; orange = upregulated under low stringency. A dash indicates that the gene was not significantly a [file pone.0000643.s005.zip › Corbo_TableS1/images/1110003E01Rik_TSS.gif]

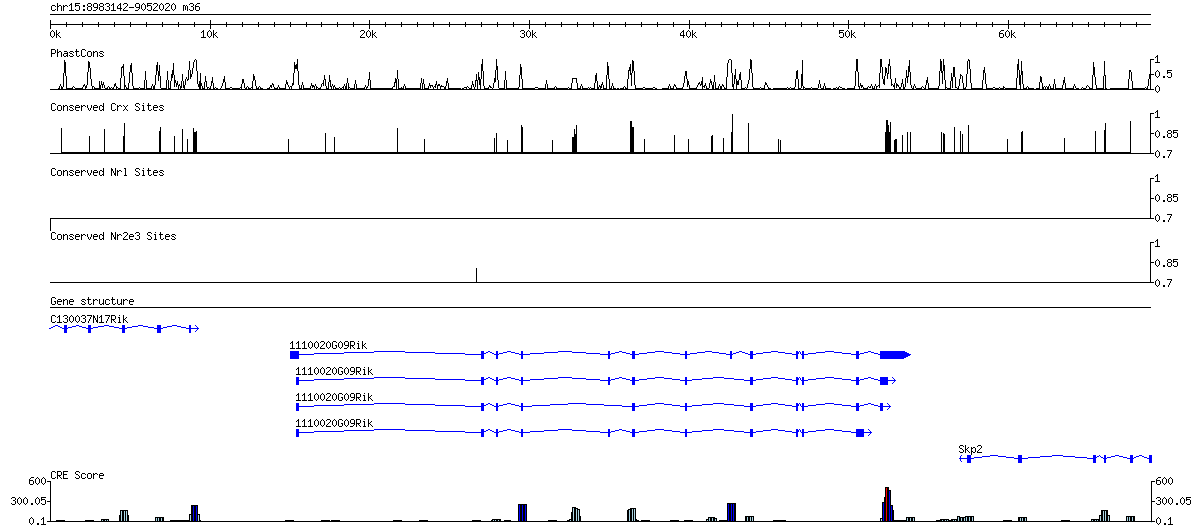

Supplement: Table S1 — Genes in the mouse photoreceptor transcription network. This table is a database which includes all genes dysregulated in Crx-/-, Nrl-/-, and/or Nr2e3-/- under high stringency criteria. Each row in the database represents a gene in the network and includes multiple links to additional types of information about that gene. ‘Large image gene +/− 15 Kb’ links to an image of our computational prediction of photoreceptor CREs in the genomic region 15 Kb upstream and downstream of the gene in question. ‘Closeup image TSS +/− 15 Kb’ links to an image of our computational prediction in the region 15 Kb on either side of the gene's TSS. Black bars within this image highlight the following regulatory peaks (if any are present): the peak closest to the TSS and the peak with the highest CRE score within this 30 Kb window. For those genes whose CRE predictions were tested experimentally, a red bar in this image indicates the location of the CRE that was tested. For Crx and Elovl4 the tested CRE is outside of this 30 Kb window and is therefore indicated in the ‘Large image’. ‘Links’ contains links to the UCSC genome browser, ENSEMBL, and NCBI database entries for the indicated gene, if available. ‘Max score (threshold of 200)’ contains the score of the highest predicted regulatory peak within the 30 Kb window around the gene's TSS. If this window does not contain any predicted peaks ≥ 200 (our cutoff threshold) no value is given (indicated by a dash). The next four columns of the table (‘Nr2e3-/-’ etc.) show the wild-type-to-mutant ratios of the averaged microarray scores for the given gene. For those genes to which more than one Affy tag correspond, the data for the first one listed under ‘Affy Mouse 430 2.0’ is given. Dark green = downregulated under high stringency (as described in METHODS); light green = downregulated under low stringency; red = upregulated under high stringency; orange = upregulated under low stringency. A dash indicates that the gene was not significantly a [file pone.0000643.s005.zip › Corbo_TableS1/images/1110020G09Rik.gif]

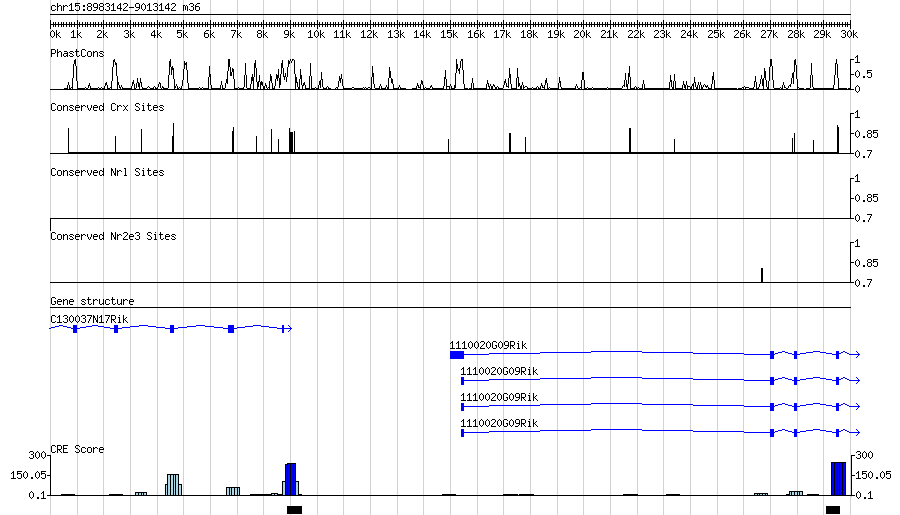

Supplement: Table S1 — Genes in the mouse photoreceptor transcription network. This table is a database which includes all genes dysregulated in Crx-/-, Nrl-/-, and/or Nr2e3-/- under high stringency criteria. Each row in the database represents a gene in the network and includes multiple links to additional types of information about that gene. ‘Large image gene +/− 15 Kb’ links to an image of our computational prediction of photoreceptor CREs in the genomic region 15 Kb upstream and downstream of the gene in question. ‘Closeup image TSS +/− 15 Kb’ links to an image of our computational prediction in the region 15 Kb on either side of the gene's TSS. Black bars within this image highlight the following regulatory peaks (if any are present): the peak closest to the TSS and the peak with the highest CRE score within this 30 Kb window. For those genes whose CRE predictions were tested experimentally, a red bar in this image indicates the location of the CRE that was tested. For Crx and Elovl4 the tested CRE is outside of this 30 Kb window and is therefore indicated in the ‘Large image’. ‘Links’ contains links to the UCSC genome browser, ENSEMBL, and NCBI database entries for the indicated gene, if available. ‘Max score (threshold of 200)’ contains the score of the highest predicted regulatory peak within the 30 Kb window around the gene's TSS. If this window does not contain any predicted peaks ≥ 200 (our cutoff threshold) no value is given (indicated by a dash). The next four columns of the table (‘Nr2e3-/-’ etc.) show the wild-type-to-mutant ratios of the averaged microarray scores for the given gene. For those genes to which more than one Affy tag correspond, the data for the first one listed under ‘Affy Mouse 430 2.0’ is given. Dark green = downregulated under high stringency (as described in METHODS); light green = downregulated under low stringency; red = upregulated under high stringency; orange = upregulated under low stringency. A dash indicates that the gene was not significantly a [file pone.0000643.s005.zip › Corbo_TableS1/images/1110020G09Rik_TSS.gif]

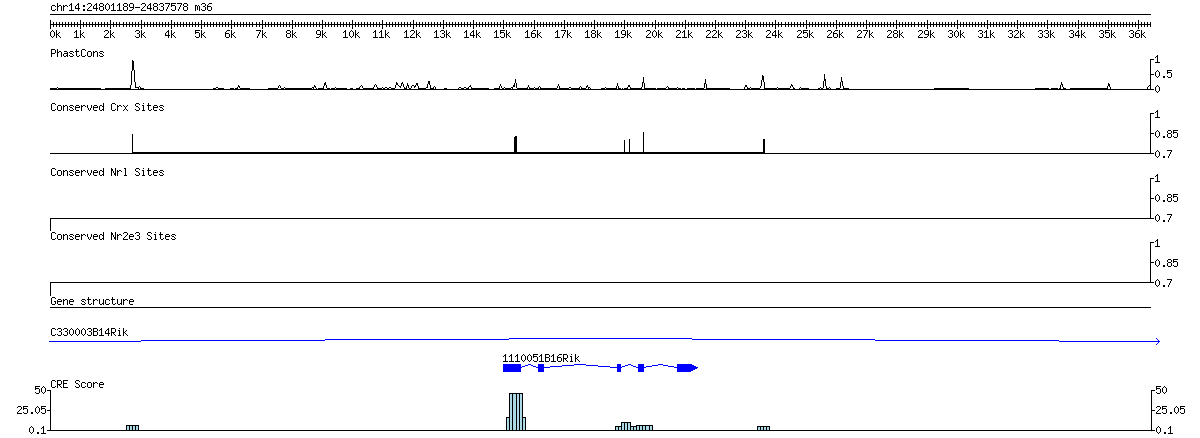

Supplement: Table S1 — Genes in the mouse photoreceptor transcription network. This table is a database which includes all genes dysregulated in Crx-/-, Nrl-/-, and/or Nr2e3-/- under high stringency criteria. Each row in the database represents a gene in the network and includes multiple links to additional types of information about that gene. ‘Large image gene +/− 15 Kb’ links to an image of our computational prediction of photoreceptor CREs in the genomic region 15 Kb upstream and downstream of the gene in question. ‘Closeup image TSS +/− 15 Kb’ links to an image of our computational prediction in the region 15 Kb on either side of the gene's TSS. Black bars within this image highlight the following regulatory peaks (if any are present): the peak closest to the TSS and the peak with the highest CRE score within this 30 Kb window. For those genes whose CRE predictions were tested experimentally, a red bar in this image indicates the location of the CRE that was tested. For Crx and Elovl4 the tested CRE is outside of this 30 Kb window and is therefore indicated in the ‘Large image’. ‘Links’ contains links to the UCSC genome browser, ENSEMBL, and NCBI database entries for the indicated gene, if available. ‘Max score (threshold of 200)’ contains the score of the highest predicted regulatory peak within the 30 Kb window around the gene's TSS. If this window does not contain any predicted peaks ≥ 200 (our cutoff threshold) no value is given (indicated by a dash). The next four columns of the table (‘Nr2e3-/-’ etc.) show the wild-type-to-mutant ratios of the averaged microarray scores for the given gene. For those genes to which more than one Affy tag correspond, the data for the first one listed under ‘Affy Mouse 430 2.0’ is given. Dark green = downregulated under high stringency (as described in METHODS); light green = downregulated under low stringency; red = upregulated under high stringency; orange = upregulated under low stringency. A dash indicates that the gene was not significantly a [file pone.0000643.s005.zip › Corbo_TableS1/images/1110051B16Rik.gif]

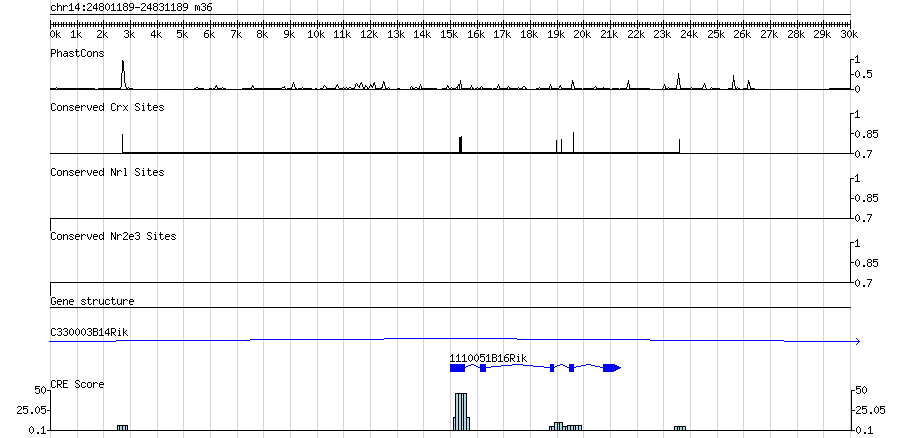

Supplement: Table S1 — Genes in the mouse photoreceptor transcription network. This table is a database which includes all genes dysregulated in Crx-/-, Nrl-/-, and/or Nr2e3-/- under high stringency criteria. Each row in the database represents a gene in the network and includes multiple links to additional types of information about that gene. ‘Large image gene +/− 15 Kb’ links to an image of our computational prediction of photoreceptor CREs in the genomic region 15 Kb upstream and downstream of the gene in question. ‘Closeup image TSS +/− 15 Kb’ links to an image of our computational prediction in the region 15 Kb on either side of the gene's TSS. Black bars within this image highlight the following regulatory peaks (if any are present): the peak closest to the TSS and the peak with the highest CRE score within this 30 Kb window. For those genes whose CRE predictions were tested experimentally, a red bar in this image indicates the location of the CRE that was tested. For Crx and Elovl4 the tested CRE is outside of this 30 Kb window and is therefore indicated in the ‘Large image’. ‘Links’ contains links to the UCSC genome browser, ENSEMBL, and NCBI database entries for the indicated gene, if available. ‘Max score (threshold of 200)’ contains the score of the highest predicted regulatory peak within the 30 Kb window around the gene's TSS. If this window does not contain any predicted peaks ≥ 200 (our cutoff threshold) no value is given (indicated by a dash). The next four columns of the table (‘Nr2e3-/-’ etc.) show the wild-type-to-mutant ratios of the averaged microarray scores for the given gene. For those genes to which more than one Affy tag correspond, the data for the first one listed under ‘Affy Mouse 430 2.0’ is given. Dark green = downregulated under high stringency (as described in METHODS); light green = downregulated under low stringency; red = upregulated under high stringency; orange = upregulated under low stringency. A dash indicates that the gene was not significantly a [file pone.0000643.s005.zip › Corbo_TableS1/images/1110051B16Rik_TSS.gif]

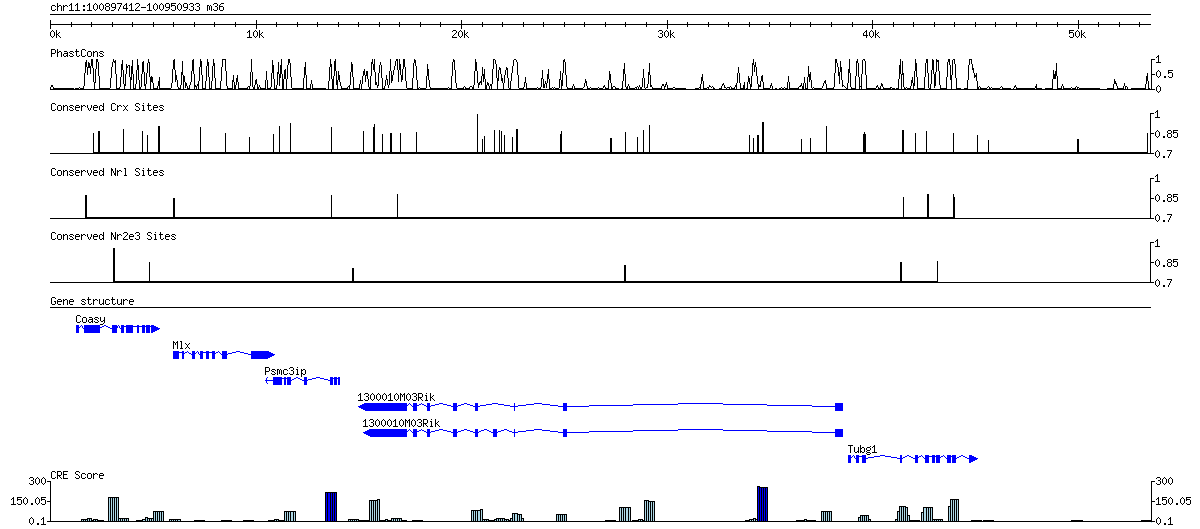

Supplement: Table S1 — Genes in the mouse photoreceptor transcription network. This table is a database which includes all genes dysregulated in Crx-/-, Nrl-/-, and/or Nr2e3-/- under high stringency criteria. Each row in the database represents a gene in the network and includes multiple links to additional types of information about that gene. ‘Large image gene +/− 15 Kb’ links to an image of our computational prediction of photoreceptor CREs in the genomic region 15 Kb upstream and downstream of the gene in question. ‘Closeup image TSS +/− 15 Kb’ links to an image of our computational prediction in the region 15 Kb on either side of the gene's TSS. Black bars within this image highlight the following regulatory peaks (if any are present): the peak closest to the TSS and the peak with the highest CRE score within this 30 Kb window. For those genes whose CRE predictions were tested experimentally, a red bar in this image indicates the location of the CRE that was tested. For Crx and Elovl4 the tested CRE is outside of this 30 Kb window and is therefore indicated in the ‘Large image’. ‘Links’ contains links to the UCSC genome browser, ENSEMBL, and NCBI database entries for the indicated gene, if available. ‘Max score (threshold of 200)’ contains the score of the highest predicted regulatory peak within the 30 Kb window around the gene's TSS. If this window does not contain any predicted peaks ≥ 200 (our cutoff threshold) no value is given (indicated by a dash). The next four columns of the table (‘Nr2e3-/-’ etc.) show the wild-type-to-mutant ratios of the averaged microarray scores for the given gene. For those genes to which more than one Affy tag correspond, the data for the first one listed under ‘Affy Mouse 430 2.0’ is given. Dark green = downregulated under high stringency (as described in METHODS); light green = downregulated under low stringency; red = upregulated under high stringency; orange = upregulated under low stringency. A dash indicates that the gene was not significantly a [file pone.0000643.s005.zip › Corbo_TableS1/images/1300010M03Rik.gif]

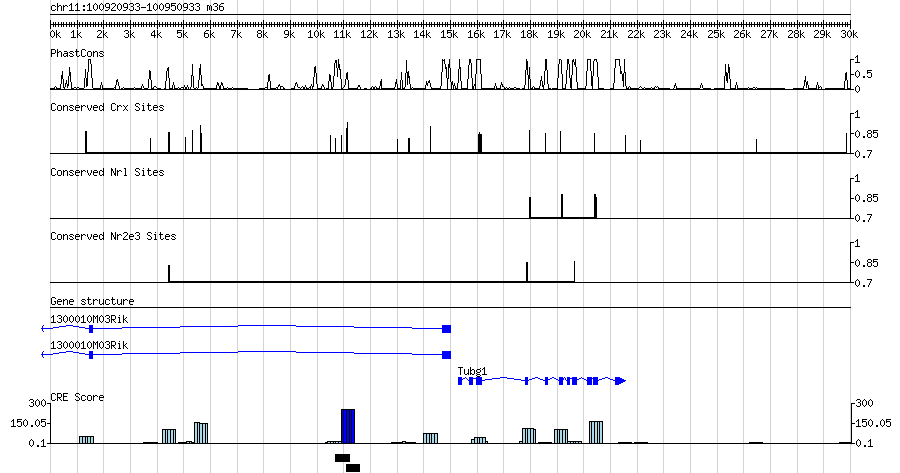

Supplement: Table S1 — Genes in the mouse photoreceptor transcription network. This table is a database which includes all genes dysregulated in Crx-/-, Nrl-/-, and/or Nr2e3-/- under high stringency criteria. Each row in the database represents a gene in the network and includes multiple links to additional types of information about that gene. ‘Large image gene +/− 15 Kb’ links to an image of our computational prediction of photoreceptor CREs in the genomic region 15 Kb upstream and downstream of the gene in question. ‘Closeup image TSS +/− 15 Kb’ links to an image of our computational prediction in the region 15 Kb on either side of the gene's TSS. Black bars within this image highlight the following regulatory peaks (if any are present): the peak closest to the TSS and the peak with the highest CRE score within this 30 Kb window. For those genes whose CRE predictions were tested experimentally, a red bar in this image indicates the location of the CRE that was tested. For Crx and Elovl4 the tested CRE is outside of this 30 Kb window and is therefore indicated in the ‘Large image’. ‘Links’ contains links to the UCSC genome browser, ENSEMBL, and NCBI database entries for the indicated gene, if available. ‘Max score (threshold of 200)’ contains the score of the highest predicted regulatory peak within the 30 Kb window around the gene's TSS. If this window does not contain any predicted peaks ≥ 200 (our cutoff threshold) no value is given (indicated by a dash). The next four columns of the table (‘Nr2e3-/-’ etc.) show the wild-type-to-mutant ratios of the averaged microarray scores for the given gene. For those genes to which more than one Affy tag correspond, the data for the first one listed under ‘Affy Mouse 430 2.0’ is given. Dark green = downregulated under high stringency (as described in METHODS); light green = downregulated under low stringency; red = upregulated under high stringency; orange = upregulated under low stringency. A dash indicates that the gene was not significantly a [file pone.0000643.s005.zip › Corbo_TableS1/images/1300010M03Rik_TSS.gif]

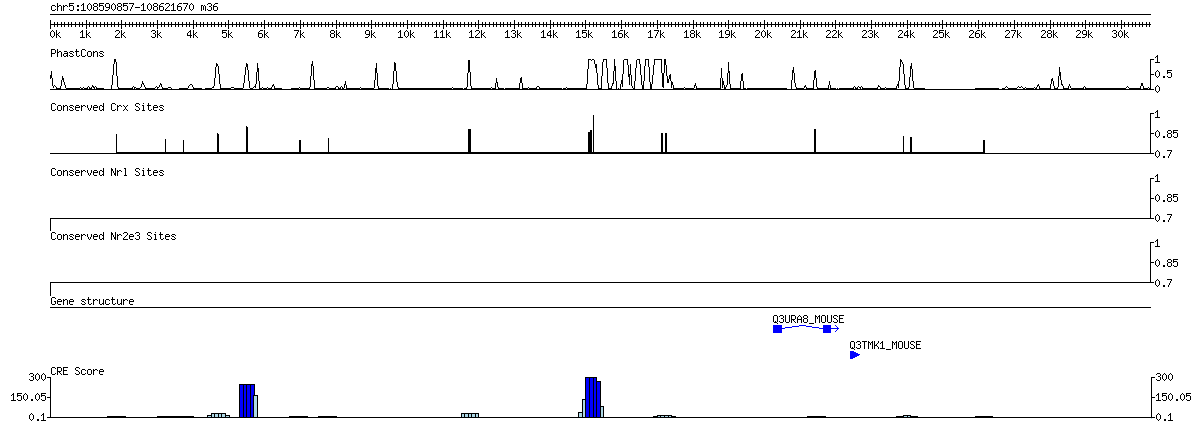

Supplement: Table S1 — Genes in the mouse photoreceptor transcription network. This table is a database which includes all genes dysregulated in Crx-/-, Nrl-/-, and/or Nr2e3-/- under high stringency criteria. Each row in the database represents a gene in the network and includes multiple links to additional types of information about that gene. ‘Large image gene +/− 15 Kb’ links to an image of our computational prediction of photoreceptor CREs in the genomic region 15 Kb upstream and downstream of the gene in question. ‘Closeup image TSS +/− 15 Kb’ links to an image of our computational prediction in the region 15 Kb on either side of the gene's TSS. Black bars within this image highlight the following regulatory peaks (if any are present): the peak closest to the TSS and the peak with the highest CRE score within this 30 Kb window. For those genes whose CRE predictions were tested experimentally, a red bar in this image indicates the location of the CRE that was tested. For Crx and Elovl4 the tested CRE is outside of this 30 Kb window and is therefore indicated in the ‘Large image’. ‘Links’ contains links to the UCSC genome browser, ENSEMBL, and NCBI database entries for the indicated gene, if available. ‘Max score (threshold of 200)’ contains the score of the highest predicted regulatory peak within the 30 Kb window around the gene's TSS. If this window does not contain any predicted peaks ≥ 200 (our cutoff threshold) no value is given (indicated by a dash). The next four columns of the table (‘Nr2e3-/-’ etc.) show the wild-type-to-mutant ratios of the averaged microarray scores for the given gene. For those genes to which more than one Affy tag correspond, the data for the first one listed under ‘Affy Mouse 430 2.0’ is given. Dark green = downregulated under high stringency (as described in METHODS); light green = downregulated under low stringency; red = upregulated under high stringency; orange = upregulated under low stringency. A dash indicates that the gene was not significantly a [file pone.0000643.s005.zip › Corbo_TableS1/images/1438068_at.gif]

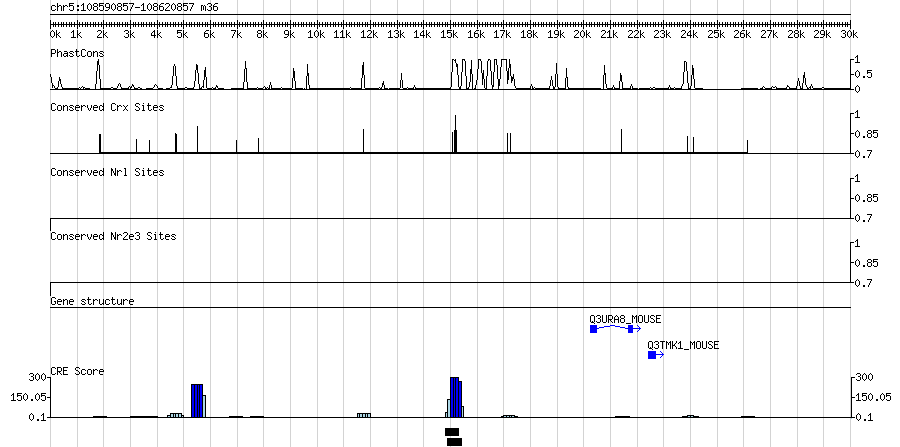

Supplement: Table S1 — Genes in the mouse photoreceptor transcription network. This table is a database which includes all genes dysregulated in Crx-/-, Nrl-/-, and/or Nr2e3-/- under high stringency criteria. Each row in the database represents a gene in the network and includes multiple links to additional types of information about that gene. ‘Large image gene +/− 15 Kb’ links to an image of our computational prediction of photoreceptor CREs in the genomic region 15 Kb upstream and downstream of the gene in question. ‘Closeup image TSS +/− 15 Kb’ links to an image of our computational prediction in the region 15 Kb on either side of the gene's TSS. Black bars within this image highlight the following regulatory peaks (if any are present): the peak closest to the TSS and the peak with the highest CRE score within this 30 Kb window. For those genes whose CRE predictions were tested experimentally, a red bar in this image indicates the location of the CRE that was tested. For Crx and Elovl4 the tested CRE is outside of this 30 Kb window and is therefore indicated in the ‘Large image’. ‘Links’ contains links to the UCSC genome browser, ENSEMBL, and NCBI database entries for the indicated gene, if available. ‘Max score (threshold of 200)’ contains the score of the highest predicted regulatory peak within the 30 Kb window around the gene's TSS. If this window does not contain any predicted peaks ≥ 200 (our cutoff threshold) no value is given (indicated by a dash). The next four columns of the table (‘Nr2e3-/-’ etc.) show the wild-type-to-mutant ratios of the averaged microarray scores for the given gene. For those genes to which more than one Affy tag correspond, the data for the first one listed under ‘Affy Mouse 430 2.0’ is given. Dark green = downregulated under high stringency (as described in METHODS); light green = downregulated under low stringency; red = upregulated under high stringency; orange = upregulated under low stringency. A dash indicates that the gene was not significantly a [file pone.0000643.s005.zip › Corbo_TableS1/images/1438068_at_TSS.gif]

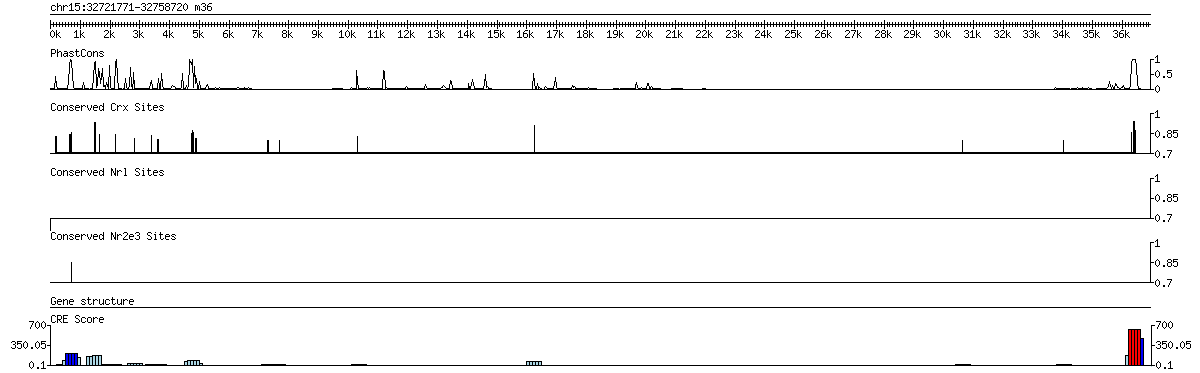

Supplement: Table S1 — Genes in the mouse photoreceptor transcription network. This table is a database which includes all genes dysregulated in Crx-/-, Nrl-/-, and/or Nr2e3-/- under high stringency criteria. Each row in the database represents a gene in the network and includes multiple links to additional types of information about that gene. ‘Large image gene +/− 15 Kb’ links to an image of our computational prediction of photoreceptor CREs in the genomic region 15 Kb upstream and downstream of the gene in question. ‘Closeup image TSS +/− 15 Kb’ links to an image of our computational prediction in the region 15 Kb on either side of the gene's TSS. Black bars within this image highlight the following regulatory peaks (if any are present): the peak closest to the TSS and the peak with the highest CRE score within this 30 Kb window. For those genes whose CRE predictions were tested experimentally, a red bar in this image indicates the location of the CRE that was tested. For Crx and Elovl4 the tested CRE is outside of this 30 Kb window and is therefore indicated in the ‘Large image’. ‘Links’ contains links to the UCSC genome browser, ENSEMBL, and NCBI database entries for the indicated gene, if available. ‘Max score (threshold of 200)’ contains the score of the highest predicted regulatory peak within the 30 Kb window around the gene's TSS. If this window does not contain any predicted peaks ≥ 200 (our cutoff threshold) no value is given (indicated by a dash). The next four columns of the table (‘Nr2e3-/-’ etc.) show the wild-type-to-mutant ratios of the averaged microarray scores for the given gene. For those genes to which more than one Affy tag correspond, the data for the first one listed under ‘Affy Mouse 430 2.0’ is given. Dark green = downregulated under high stringency (as described in METHODS); light green = downregulated under low stringency; red = upregulated under high stringency; orange = upregulated under low stringency. A dash indicates that the gene was not significantly a [file pone.0000643.s005.zip › Corbo_TableS1/images/1439911_at.gif]

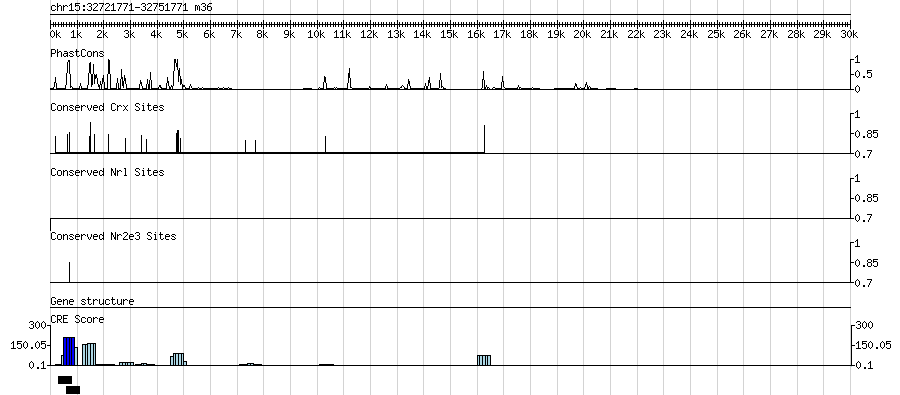

Supplement: Table S1 — Genes in the mouse photoreceptor transcription network. This table is a database which includes all genes dysregulated in Crx-/-, Nrl-/-, and/or Nr2e3-/- under high stringency criteria. Each row in the database represents a gene in the network and includes multiple links to additional types of information about that gene. ‘Large image gene +/− 15 Kb’ links to an image of our computational prediction of photoreceptor CREs in the genomic region 15 Kb upstream and downstream of the gene in question. ‘Closeup image TSS +/− 15 Kb’ links to an image of our computational prediction in the region 15 Kb on either side of the gene's TSS. Black bars within this image highlight the following regulatory peaks (if any are present): the peak closest to the TSS and the peak with the highest CRE score within this 30 Kb window. For those genes whose CRE predictions were tested experimentally, a red bar in this image indicates the location of the CRE that was tested. For Crx and Elovl4 the tested CRE is outside of this 30 Kb window and is therefore indicated in the ‘Large image’. ‘Links’ contains links to the UCSC genome browser, ENSEMBL, and NCBI database entries for the indicated gene, if available. ‘Max score (threshold of 200)’ contains the score of the highest predicted regulatory peak within the 30 Kb window around the gene's TSS. If this window does not contain any predicted peaks ≥ 200 (our cutoff threshold) no value is given (indicated by a dash). The next four columns of the table (‘Nr2e3-/-’ etc.) show the wild-type-to-mutant ratios of the averaged microarray scores for the given gene. For those genes to which more than one Affy tag correspond, the data for the first one listed under ‘Affy Mouse 430 2.0’ is given. Dark green = downregulated under high stringency (as described in METHODS); light green = downregulated under low stringency; red = upregulated under high stringency; orange = upregulated under low stringency. A dash indicates that the gene was not significantly a [file pone.0000643.s005.zip › Corbo_TableS1/images/1439911_at_TSS.gif]

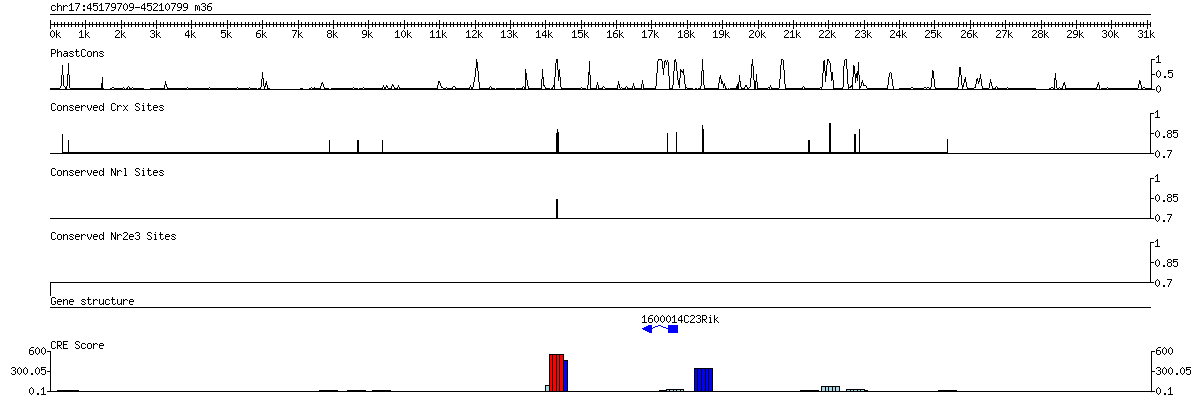

Supplement: Table S1 — Genes in the mouse photoreceptor transcription network. This table is a database which includes all genes dysregulated in Crx-/-, Nrl-/-, and/or Nr2e3-/- under high stringency criteria. Each row in the database represents a gene in the network and includes multiple links to additional types of information about that gene. ‘Large image gene +/− 15 Kb’ links to an image of our computational prediction of photoreceptor CREs in the genomic region 15 Kb upstream and downstream of the gene in question. ‘Closeup image TSS +/− 15 Kb’ links to an image of our computational prediction in the region 15 Kb on either side of the gene's TSS. Black bars within this image highlight the following regulatory peaks (if any are present): the peak closest to the TSS and the peak with the highest CRE score within this 30 Kb window. For those genes whose CRE predictions were tested experimentally, a red bar in this image indicates the location of the CRE that was tested. For Crx and Elovl4 the tested CRE is outside of this 30 Kb window and is therefore indicated in the ‘Large image’. ‘Links’ contains links to the UCSC genome browser, ENSEMBL, and NCBI database entries for the indicated gene, if available. ‘Max score (threshold of 200)’ contains the score of the highest predicted regulatory peak within the 30 Kb window around the gene's TSS. If this window does not contain any predicted peaks ≥ 200 (our cutoff threshold) no value is given (indicated by a dash). The next four columns of the table (‘Nr2e3-/-’ etc.) show the wild-type-to-mutant ratios of the averaged microarray scores for the given gene. For those genes to which more than one Affy tag correspond, the data for the first one listed under ‘Affy Mouse 430 2.0’ is given. Dark green = downregulated under high stringency (as described in METHODS); light green = downregulated under low stringency; red = upregulated under high stringency; orange = upregulated under low stringency. A dash indicates that the gene was not significantly a [file pone.0000643.s005.zip › Corbo_TableS1/images/1439936_at.gif]

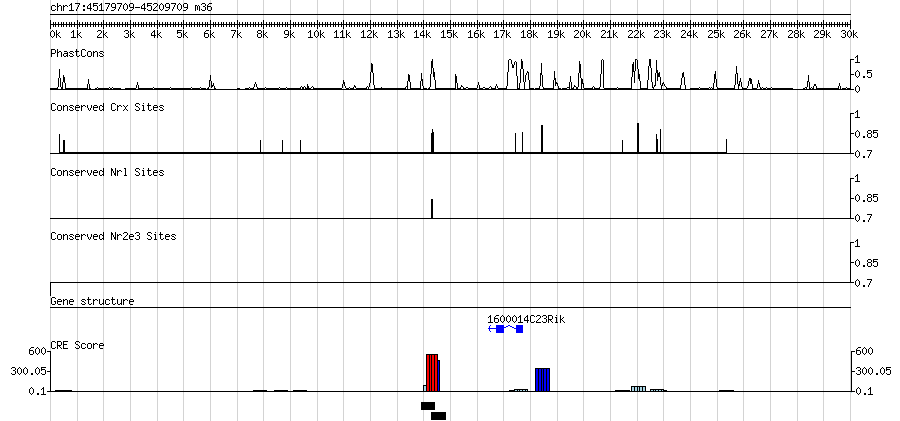

Supplement: Table S1 — Genes in the mouse photoreceptor transcription network. This table is a database which includes all genes dysregulated in Crx-/-, Nrl-/-, and/or Nr2e3-/- under high stringency criteria. Each row in the database represents a gene in the network and includes multiple links to additional types of information about that gene. ‘Large image gene +/− 15 Kb’ links to an image of our computational prediction of photoreceptor CREs in the genomic region 15 Kb upstream and downstream of the gene in question. ‘Closeup image TSS +/− 15 Kb’ links to an image of our computational prediction in the region 15 Kb on either side of the gene's TSS. Black bars within this image highlight the following regulatory peaks (if any are present): the peak closest to the TSS and the peak with the highest CRE score within this 30 Kb window. For those genes whose CRE predictions were tested experimentally, a red bar in this image indicates the location of the CRE that was tested. For Crx and Elovl4 the tested CRE is outside of this 30 Kb window and is therefore indicated in the ‘Large image’. ‘Links’ contains links to the UCSC genome browser, ENSEMBL, and NCBI database entries for the indicated gene, if available. ‘Max score (threshold of 200)’ contains the score of the highest predicted regulatory peak within the 30 Kb window around the gene's TSS. If this window does not contain any predicted peaks ≥ 200 (our cutoff threshold) no value is given (indicated by a dash). The next four columns of the table (‘Nr2e3-/-’ etc.) show the wild-type-to-mutant ratios of the averaged microarray scores for the given gene. For those genes to which more than one Affy tag correspond, the data for the first one listed under ‘Affy Mouse 430 2.0’ is given. Dark green = downregulated under high stringency (as described in METHODS); light green = downregulated under low stringency; red = upregulated under high stringency; orange = upregulated under low stringency. A dash indicates that the gene was not significantly a [file pone.0000643.s005.zip › Corbo_TableS1/images/1439936_at_TSS.gif]

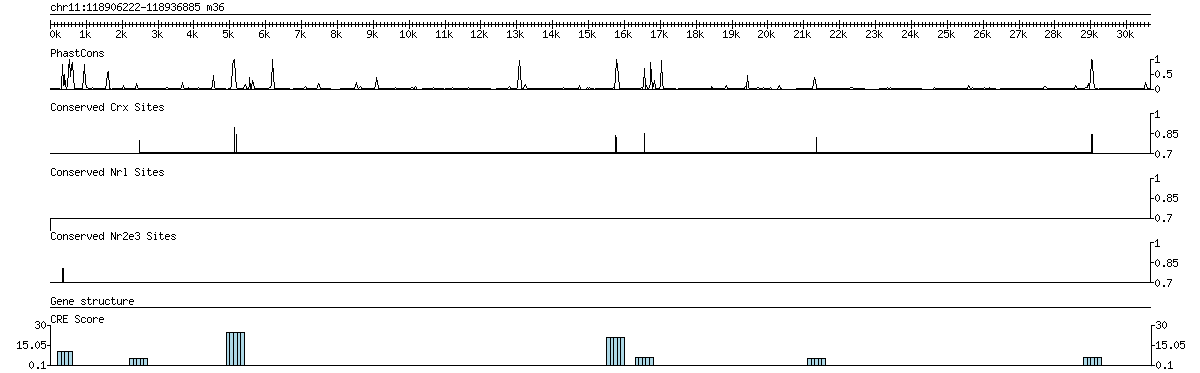

Supplement: Table S1 — Genes in the mouse photoreceptor transcription network. This table is a database which includes all genes dysregulated in Crx-/-, Nrl-/-, and/or Nr2e3-/- under high stringency criteria. Each row in the database represents a gene in the network and includes multiple links to additional types of information about that gene. ‘Large image gene +/− 15 Kb’ links to an image of our computational prediction of photoreceptor CREs in the genomic region 15 Kb upstream and downstream of the gene in question. ‘Closeup image TSS +/− 15 Kb’ links to an image of our computational prediction in the region 15 Kb on either side of the gene's TSS. Black bars within this image highlight the following regulatory peaks (if any are present): the peak closest to the TSS and the peak with the highest CRE score within this 30 Kb window. For those genes whose CRE predictions were tested experimentally, a red bar in this image indicates the location of the CRE that was tested. For Crx and Elovl4 the tested CRE is outside of this 30 Kb window and is therefore indicated in the ‘Large image’. ‘Links’ contains links to the UCSC genome browser, ENSEMBL, and NCBI database entries for the indicated gene, if available. ‘Max score (threshold of 200)’ contains the score of the highest predicted regulatory peak within the 30 Kb window around the gene's TSS. If this window does not contain any predicted peaks ≥ 200 (our cutoff threshold) no value is given (indicated by a dash). The next four columns of the table (‘Nr2e3-/-’ etc.) show the wild-type-to-mutant ratios of the averaged microarray scores for the given gene. For those genes to which more than one Affy tag correspond, the data for the first one listed under ‘Affy Mouse 430 2.0’ is given. Dark green = downregulated under high stringency (as described in METHODS); light green = downregulated under low stringency; red = upregulated under high stringency; orange = upregulated under low stringency. A dash indicates that the gene was not significantly a [file pone.0000643.s005.zip › Corbo_TableS1/images/1441518_at.gif]

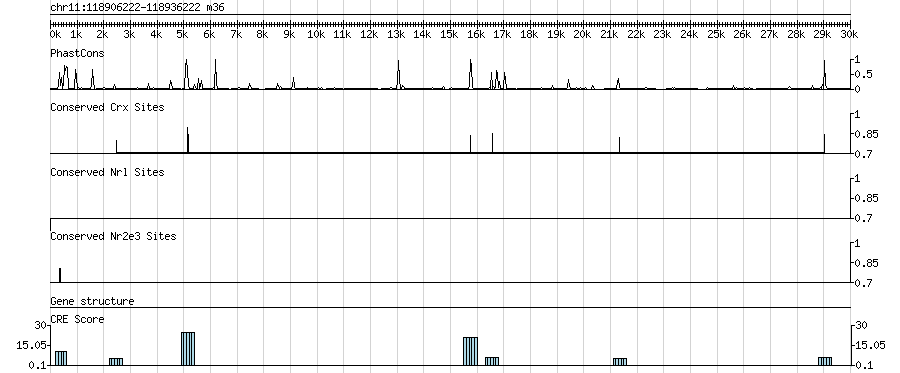

Supplement: Table S1 — Genes in the mouse photoreceptor transcription network. This table is a database which includes all genes dysregulated in Crx-/-, Nrl-/-, and/or Nr2e3-/- under high stringency criteria. Each row in the database represents a gene in the network and includes multiple links to additional types of information about that gene. ‘Large image gene +/− 15 Kb’ links to an image of our computational prediction of photoreceptor CREs in the genomic region 15 Kb upstream and downstream of the gene in question. ‘Closeup image TSS +/− 15 Kb’ links to an image of our computational prediction in the region 15 Kb on either side of the gene's TSS. Black bars within this image highlight the following regulatory peaks (if any are present): the peak closest to the TSS and the peak with the highest CRE score within this 30 Kb window. For those genes whose CRE predictions were tested experimentally, a red bar in this image indicates the location of the CRE that was tested. For Crx and Elovl4 the tested CRE is outside of this 30 Kb window and is therefore indicated in the ‘Large image’. ‘Links’ contains links to the UCSC genome browser, ENSEMBL, and NCBI database entries for the indicated gene, if available. ‘Max score (threshold of 200)’ contains the score of the highest predicted regulatory peak within the 30 Kb window around the gene's TSS. If this window does not contain any predicted peaks ≥ 200 (our cutoff threshold) no value is given (indicated by a dash). The next four columns of the table (‘Nr2e3-/-’ etc.) show the wild-type-to-mutant ratios of the averaged microarray scores for the given gene. For those genes to which more than one Affy tag correspond, the data for the first one listed under ‘Affy Mouse 430 2.0’ is given. Dark green = downregulated under high stringency (as described in METHODS); light green = downregulated under low stringency; red = upregulated under high stringency; orange = upregulated under low stringency. A dash indicates that the gene was not significantly a [file pone.0000643.s005.zip › Corbo_TableS1/images/1441518_at_TSS.gif]

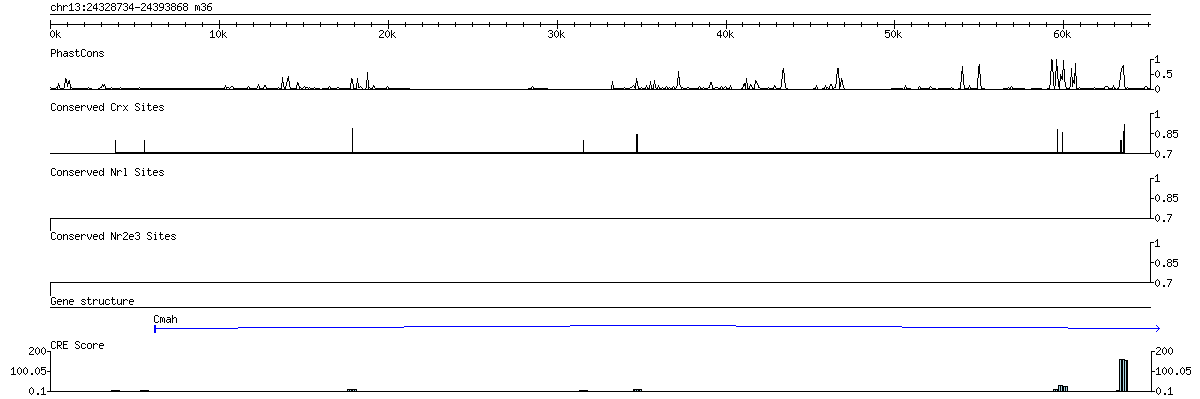

Supplement: Table S1 — Genes in the mouse photoreceptor transcription network. This table is a database which includes all genes dysregulated in Crx-/-, Nrl-/-, and/or Nr2e3-/- under high stringency criteria. Each row in the database represents a gene in the network and includes multiple links to additional types of information about that gene. ‘Large image gene +/− 15 Kb’ links to an image of our computational prediction of photoreceptor CREs in the genomic region 15 Kb upstream and downstream of the gene in question. ‘Closeup image TSS +/− 15 Kb’ links to an image of our computational prediction in the region 15 Kb on either side of the gene's TSS. Black bars within this image highlight the following regulatory peaks (if any are present): the peak closest to the TSS and the peak with the highest CRE score within this 30 Kb window. For those genes whose CRE predictions were tested experimentally, a red bar in this image indicates the location of the CRE that was tested. For Crx and Elovl4 the tested CRE is outside of this 30 Kb window and is therefore indicated in the ‘Large image’. ‘Links’ contains links to the UCSC genome browser, ENSEMBL, and NCBI database entries for the indicated gene, if available. ‘Max score (threshold of 200)’ contains the score of the highest predicted regulatory peak within the 30 Kb window around the gene's TSS. If this window does not contain any predicted peaks ≥ 200 (our cutoff threshold) no value is given (indicated by a dash). The next four columns of the table (‘Nr2e3-/-’ etc.) show the wild-type-to-mutant ratios of the averaged microarray scores for the given gene. For those genes to which more than one Affy tag correspond, the data for the first one listed under ‘Affy Mouse 430 2.0’ is given. Dark green = downregulated under high stringency (as described in METHODS); light green = downregulated under low stringency; red = upregulated under high stringency; orange = upregulated under low stringency. A dash indicates that the gene was not significantly a [file pone.0000643.s005.zip › Corbo_TableS1/images/1442190_at.gif]

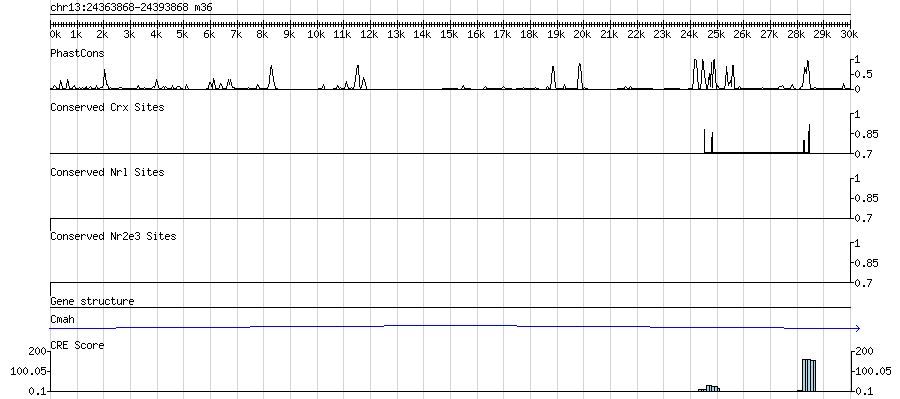

Supplement: Table S1 — Genes in the mouse photoreceptor transcription network. This table is a database which includes all genes dysregulated in Crx-/-, Nrl-/-, and/or Nr2e3-/- under high stringency criteria. Each row in the database represents a gene in the network and includes multiple links to additional types of information about that gene. ‘Large image gene +/− 15 Kb’ links to an image of our computational prediction of photoreceptor CREs in the genomic region 15 Kb upstream and downstream of the gene in question. ‘Closeup image TSS +/− 15 Kb’ links to an image of our computational prediction in the region 15 Kb on either side of the gene's TSS. Black bars within this image highlight the following regulatory peaks (if any are present): the peak closest to the TSS and the peak with the highest CRE score within this 30 Kb window. For those genes whose CRE predictions were tested experimentally, a red bar in this image indicates the location of the CRE that was tested. For Crx and Elovl4 the tested CRE is outside of this 30 Kb window and is therefore indicated in the ‘Large image’. ‘Links’ contains links to the UCSC genome browser, ENSEMBL, and NCBI database entries for the indicated gene, if available. ‘Max score (threshold of 200)’ contains the score of the highest predicted regulatory peak within the 30 Kb window around the gene's TSS. If this window does not contain any predicted peaks ≥ 200 (our cutoff threshold) no value is given (indicated by a dash). The next four columns of the table (‘Nr2e3-/-’ etc.) show the wild-type-to-mutant ratios of the averaged microarray scores for the given gene. For those genes to which more than one Affy tag correspond, the data for the first one listed under ‘Affy Mouse 430 2.0’ is given. Dark green = downregulated under high stringency (as described in METHODS); light green = downregulated under low stringency; red = upregulated under high stringency; orange = upregulated under low stringency. A dash indicates that the gene was not significantly a [file pone.0000643.s005.zip › Corbo_TableS1/images/1442190_at_TSS.gif]

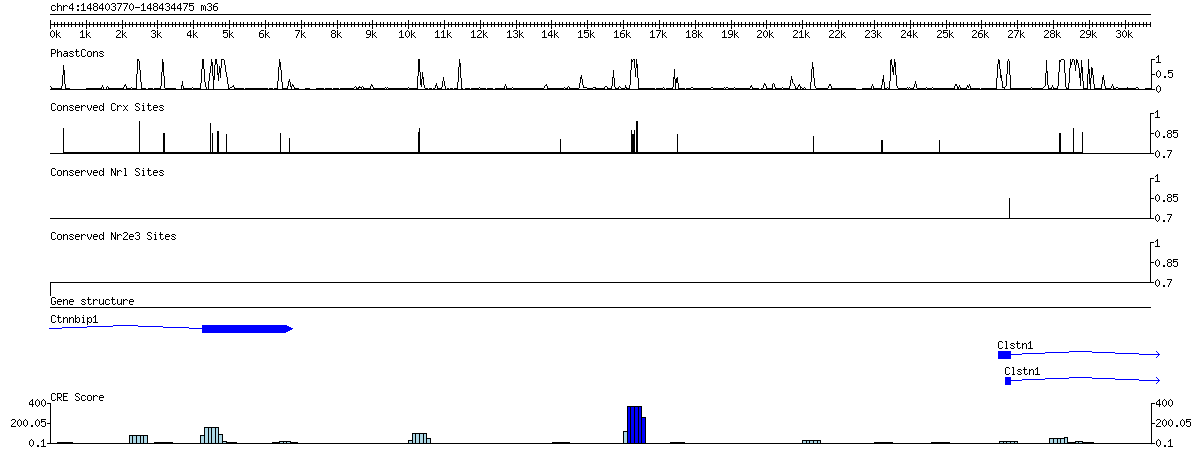

Supplement: Table S1 — Genes in the mouse photoreceptor transcription network. This table is a database which includes all genes dysregulated in Crx-/-, Nrl-/-, and/or Nr2e3-/- under high stringency criteria. Each row in the database represents a gene in the network and includes multiple links to additional types of information about that gene. ‘Large image gene +/− 15 Kb’ links to an image of our computational prediction of photoreceptor CREs in the genomic region 15 Kb upstream and downstream of the gene in question. ‘Closeup image TSS +/− 15 Kb’ links to an image of our computational prediction in the region 15 Kb on either side of the gene's TSS. Black bars within this image highlight the following regulatory peaks (if any are present): the peak closest to the TSS and the peak with the highest CRE score within this 30 Kb window. For those genes whose CRE predictions were tested experimentally, a red bar in this image indicates the location of the CRE that was tested. For Crx and Elovl4 the tested CRE is outside of this 30 Kb window and is therefore indicated in the ‘Large image’. ‘Links’ contains links to the UCSC genome browser, ENSEMBL, and NCBI database entries for the indicated gene, if available. ‘Max score (threshold of 200)’ contains the score of the highest predicted regulatory peak within the 30 Kb window around the gene's TSS. If this window does not contain any predicted peaks ≥ 200 (our cutoff threshold) no value is given (indicated by a dash). The next four columns of the table (‘Nr2e3-/-’ etc.) show the wild-type-to-mutant ratios of the averaged microarray scores for the given gene. For those genes to which more than one Affy tag correspond, the data for the first one listed under ‘Affy Mouse 430 2.0’ is given. Dark green = downregulated under high stringency (as described in METHODS); light green = downregulated under low stringency; red = upregulated under high stringency; orange = upregulated under low stringency. A dash indicates that the gene was not significantly a [file pone.0000643.s005.zip › Corbo_TableS1/images/1442249_at.gif]

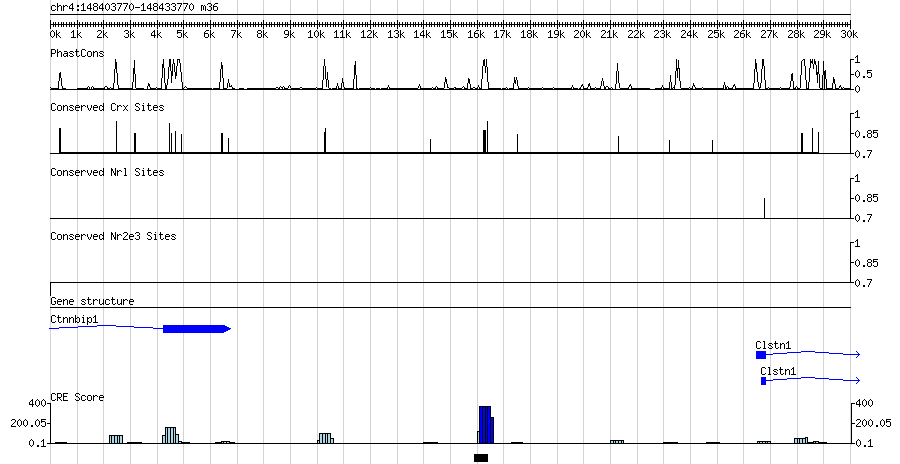

Supplement: Table S1 — Genes in the mouse photoreceptor transcription network. This table is a database which includes all genes dysregulated in Crx-/-, Nrl-/-, and/or Nr2e3-/- under high stringency criteria. Each row in the database represents a gene in the network and includes multiple links to additional types of information about that gene. ‘Large image gene +/− 15 Kb’ links to an image of our computational prediction of photoreceptor CREs in the genomic region 15 Kb upstream and downstream of the gene in question. ‘Closeup image TSS +/− 15 Kb’ links to an image of our computational prediction in the region 15 Kb on either side of the gene's TSS. Black bars within this image highlight the following regulatory peaks (if any are present): the peak closest to the TSS and the peak with the highest CRE score within this 30 Kb window. For those genes whose CRE predictions were tested experimentally, a red bar in this image indicates the location of the CRE that was tested. For Crx and Elovl4 the tested CRE is outside of this 30 Kb window and is therefore indicated in the ‘Large image’. ‘Links’ contains links to the UCSC genome browser, ENSEMBL, and NCBI database entries for the indicated gene, if available. ‘Max score (threshold of 200)’ contains the score of the highest predicted regulatory peak within the 30 Kb window around the gene's TSS. If this window does not contain any predicted peaks ≥ 200 (our cutoff threshold) no value is given (indicated by a dash). The next four columns of the table (‘Nr2e3-/-’ etc.) show the wild-type-to-mutant ratios of the averaged microarray scores for the given gene. For those genes to which more than one Affy tag correspond, the data for the first one listed under ‘Affy Mouse 430 2.0’ is given. Dark green = downregulated under high stringency (as described in METHODS); light green = downregulated under low stringency; red = upregulated under high stringency; orange = upregulated under low stringency. A dash indicates that the gene was not significantly a [file pone.0000643.s005.zip › Corbo_TableS1/images/1442249_at_TSS.gif]

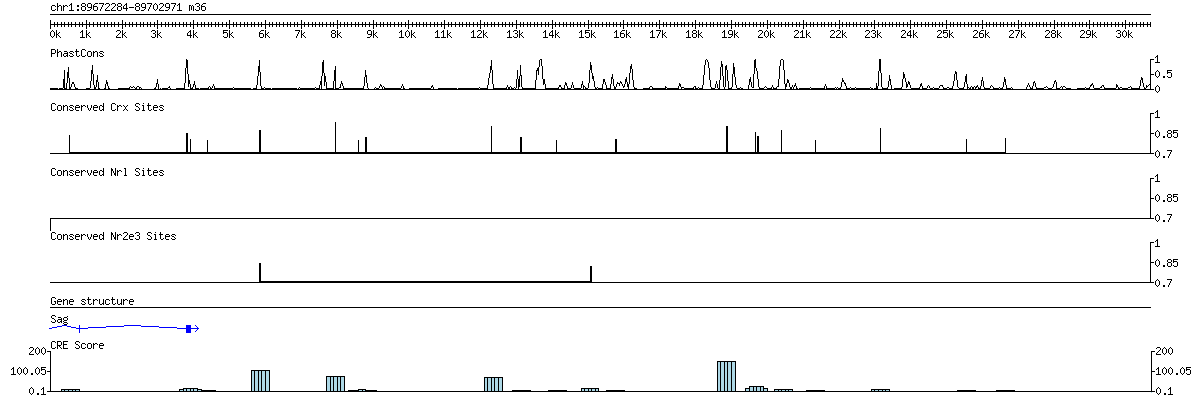

Supplement: Table S1 — Genes in the mouse photoreceptor transcription network. This table is a database which includes all genes dysregulated in Crx-/-, Nrl-/-, and/or Nr2e3-/- under high stringency criteria. Each row in the database represents a gene in the network and includes multiple links to additional types of information about that gene. ‘Large image gene +/− 15 Kb’ links to an image of our computational prediction of photoreceptor CREs in the genomic region 15 Kb upstream and downstream of the gene in question. ‘Closeup image TSS +/− 15 Kb’ links to an image of our computational prediction in the region 15 Kb on either side of the gene's TSS. Black bars within this image highlight the following regulatory peaks (if any are present): the peak closest to the TSS and the peak with the highest CRE score within this 30 Kb window. For those genes whose CRE predictions were tested experimentally, a red bar in this image indicates the location of the CRE that was tested. For Crx and Elovl4 the tested CRE is outside of this 30 Kb window and is therefore indicated in the ‘Large image’. ‘Links’ contains links to the UCSC genome browser, ENSEMBL, and NCBI database entries for the indicated gene, if available. ‘Max score (threshold of 200)’ contains the score of the highest predicted regulatory peak within the 30 Kb window around the gene's TSS. If this window does not contain any predicted peaks ≥ 200 (our cutoff threshold) no value is given (indicated by a dash). The next four columns of the table (‘Nr2e3-/-’ etc.) show the wild-type-to-mutant ratios of the averaged microarray scores for the given gene. For those genes to which more than one Affy tag correspond, the data for the first one listed under ‘Affy Mouse 430 2.0’ is given. Dark green = downregulated under high stringency (as described in METHODS); light green = downregulated under low stringency; red = upregulated under high stringency; orange = upregulated under low stringency. A dash indicates that the gene was not significantly a [file pone.0000643.s005.zip › Corbo_TableS1/images/1442254_at.gif]

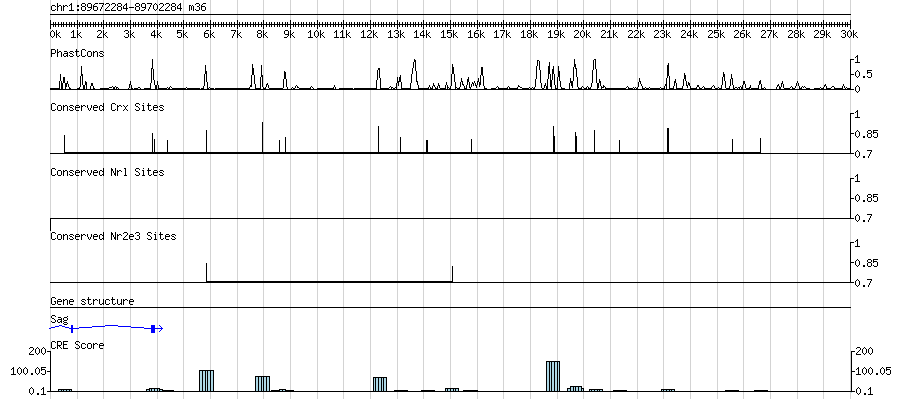

Supplement: Table S1 — Genes in the mouse photoreceptor transcription network. This table is a database which includes all genes dysregulated in Crx-/-, Nrl-/-, and/or Nr2e3-/- under high stringency criteria. Each row in the database represents a gene in the network and includes multiple links to additional types of information about that gene. ‘Large image gene +/− 15 Kb’ links to an image of our computational prediction of photoreceptor CREs in the genomic region 15 Kb upstream and downstream of the gene in question. ‘Closeup image TSS +/− 15 Kb’ links to an image of our computational prediction in the region 15 Kb on either side of the gene's TSS. Black bars within this image highlight the following regulatory peaks (if any are present): the peak closest to the TSS and the peak with the highest CRE score within this 30 Kb window. For those genes whose CRE predictions were tested experimentally, a red bar in this image indicates the location of the CRE that was tested. For Crx and Elovl4 the tested CRE is outside of this 30 Kb window and is therefore indicated in the ‘Large image’. ‘Links’ contains links to the UCSC genome browser, ENSEMBL, and NCBI database entries for the indicated gene, if available. ‘Max score (threshold of 200)’ contains the score of the highest predicted regulatory peak within the 30 Kb window around the gene's TSS. If this window does not contain any predicted peaks ≥ 200 (our cutoff threshold) no value is given (indicated by a dash). The next four columns of the table (‘Nr2e3-/-’ etc.) show the wild-type-to-mutant ratios of the averaged microarray scores for the given gene. For those genes to which more than one Affy tag correspond, the data for the first one listed under ‘Affy Mouse 430 2.0’ is given. Dark green = downregulated under high stringency (as described in METHODS); light green = downregulated under low stringency; red = upregulated under high stringency; orange = upregulated under low stringency. A dash indicates that the gene was not significantly a [file pone.0000643.s005.zip › Corbo_TableS1/images/1442254_at_TSS.gif]

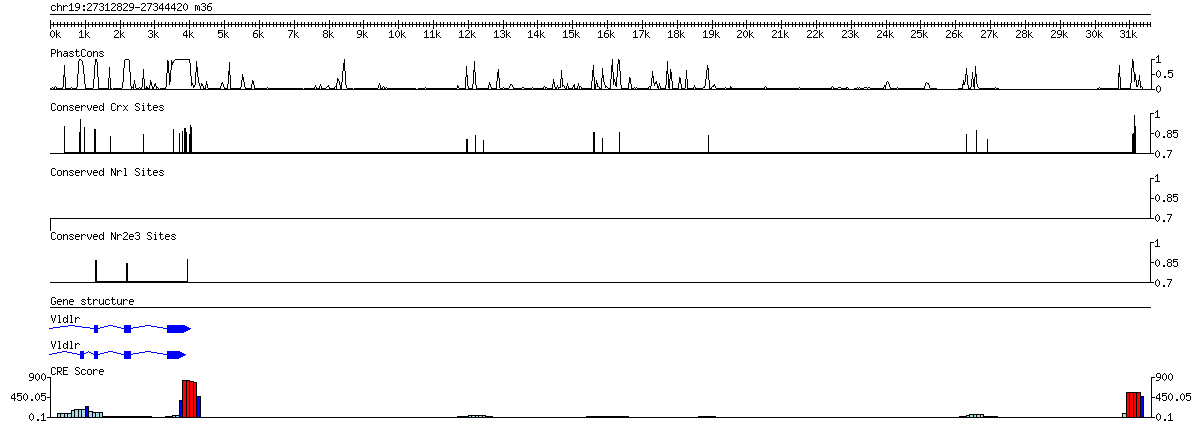

Supplement: Table S1 — Genes in the mouse photoreceptor transcription network. This table is a database which includes all genes dysregulated in Crx-/-, Nrl-/-, and/or Nr2e3-/- under high stringency criteria. Each row in the database represents a gene in the network and includes multiple links to additional types of information about that gene. ‘Large image gene +/− 15 Kb’ links to an image of our computational prediction of photoreceptor CREs in the genomic region 15 Kb upstream and downstream of the gene in question. ‘Closeup image TSS +/− 15 Kb’ links to an image of our computational prediction in the region 15 Kb on either side of the gene's TSS. Black bars within this image highlight the following regulatory peaks (if any are present): the peak closest to the TSS and the peak with the highest CRE score within this 30 Kb window. For those genes whose CRE predictions were tested experimentally, a red bar in this image indicates the location of the CRE that was tested. For Crx and Elovl4 the tested CRE is outside of this 30 Kb window and is therefore indicated in the ‘Large image’. ‘Links’ contains links to the UCSC genome browser, ENSEMBL, and NCBI database entries for the indicated gene, if available. ‘Max score (threshold of 200)’ contains the score of the highest predicted regulatory peak within the 30 Kb window around the gene's TSS. If this window does not contain any predicted peaks ≥ 200 (our cutoff threshold) no value is given (indicated by a dash). The next four columns of the table (‘Nr2e3-/-’ etc.) show the wild-type-to-mutant ratios of the averaged microarray scores for the given gene. For those genes to which more than one Affy tag correspond, the data for the first one listed under ‘Affy Mouse 430 2.0’ is given. Dark green = downregulated under high stringency (as described in METHODS); light green = downregulated under low stringency; red = upregulated under high stringency; orange = upregulated under low stringency. A dash indicates that the gene was not significantly a [file pone.0000643.s005.zip › Corbo_TableS1/images/1442304_at.gif]

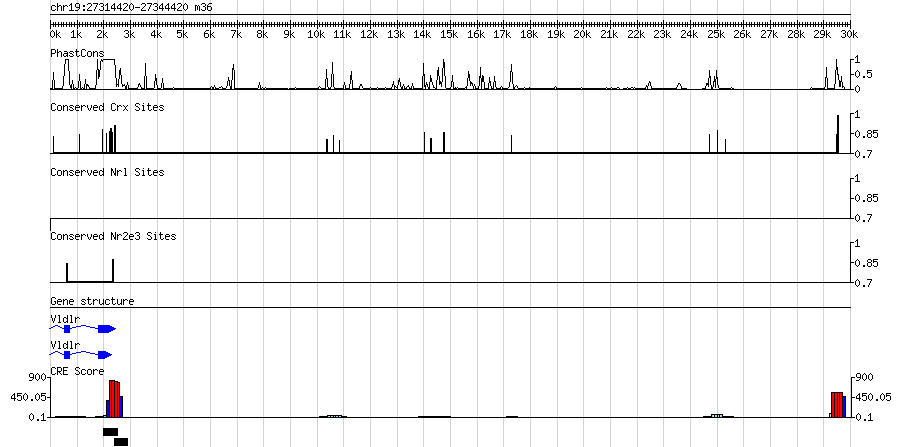

Supplement: Table S1 — Genes in the mouse photoreceptor transcription network. This table is a database which includes all genes dysregulated in Crx-/-, Nrl-/-, and/or Nr2e3-/- under high stringency criteria. Each row in the database represents a gene in the network and includes multiple links to additional types of information about that gene. ‘Large image gene +/− 15 Kb’ links to an image of our computational prediction of photoreceptor CREs in the genomic region 15 Kb upstream and downstream of the gene in question. ‘Closeup image TSS +/− 15 Kb’ links to an image of our computational prediction in the region 15 Kb on either side of the gene's TSS. Black bars within this image highlight the following regulatory peaks (if any are present): the peak closest to the TSS and the peak with the highest CRE score within this 30 Kb window. For those genes whose CRE predictions were tested experimentally, a red bar in this image indicates the location of the CRE that was tested. For Crx and Elovl4 the tested CRE is outside of this 30 Kb window and is therefore indicated in the ‘Large image’. ‘Links’ contains links to the UCSC genome browser, ENSEMBL, and NCBI database entries for the indicated gene, if available. ‘Max score (threshold of 200)’ contains the score of the highest predicted regulatory peak within the 30 Kb window around the gene's TSS. If this window does not contain any predicted peaks ≥ 200 (our cutoff threshold) no value is given (indicated by a dash). The next four columns of the table (‘Nr2e3-/-’ etc.) show the wild-type-to-mutant ratios of the averaged microarray scores for the given gene. For those genes to which more than one Affy tag correspond, the data for the first one listed under ‘Affy Mouse 430 2.0’ is given. Dark green = downregulated under high stringency (as described in METHODS); light green = downregulated under low stringency; red = upregulated under high stringency; orange = upregulated under low stringency. A dash indicates that the gene was not significantly a [file pone.0000643.s005.zip › Corbo_TableS1/images/1442304_at_TSS.gif]

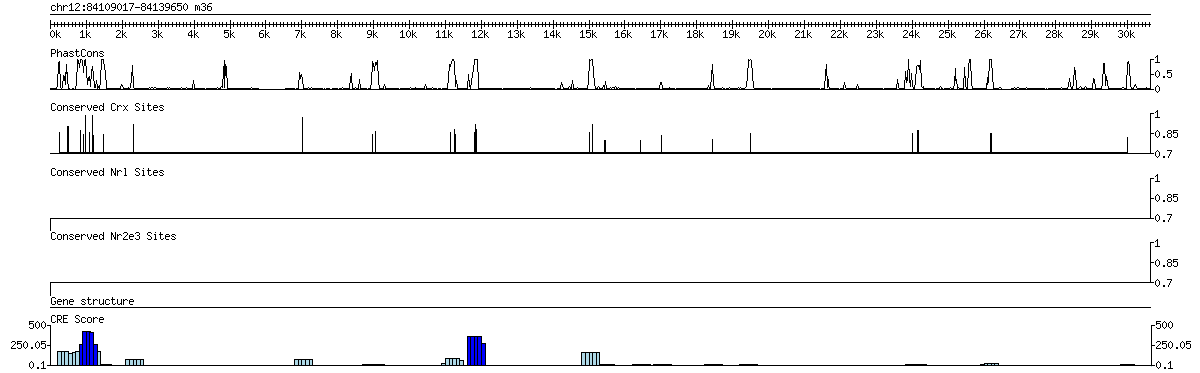

Supplement: Table S1 — Genes in the mouse photoreceptor transcription network. This table is a database which includes all genes dysregulated in Crx-/-, Nrl-/-, and/or Nr2e3-/- under high stringency criteria. Each row in the database represents a gene in the network and includes multiple links to additional types of information about that gene. ‘Large image gene +/− 15 Kb’ links to an image of our computational prediction of photoreceptor CREs in the genomic region 15 Kb upstream and downstream of the gene in question. ‘Closeup image TSS +/− 15 Kb’ links to an image of our computational prediction in the region 15 Kb on either side of the gene's TSS. Black bars within this image highlight the following regulatory peaks (if any are present): the peak closest to the TSS and the peak with the highest CRE score within this 30 Kb window. For those genes whose CRE predictions were tested experimentally, a red bar in this image indicates the location of the CRE that was tested. For Crx and Elovl4 the tested CRE is outside of this 30 Kb window and is therefore indicated in the ‘Large image’. ‘Links’ contains links to the UCSC genome browser, ENSEMBL, and NCBI database entries for the indicated gene, if available. ‘Max score (threshold of 200)’ contains the score of the highest predicted regulatory peak within the 30 Kb window around the gene's TSS. If this window does not contain any predicted peaks ≥ 200 (our cutoff threshold) no value is given (indicated by a dash). The next four columns of the table (‘Nr2e3-/-’ etc.) show the wild-type-to-mutant ratios of the averaged microarray scores for the given gene. For those genes to which more than one Affy tag correspond, the data for the first one listed under ‘Affy Mouse 430 2.0’ is given. Dark green = downregulated under high stringency (as described in METHODS); light green = downregulated under low stringency; red = upregulated under high stringency; orange = upregulated under low stringency. A dash indicates that the gene was not significantly a [file pone.0000643.s005.zip › Corbo_TableS1/images/1443306_at.gif]

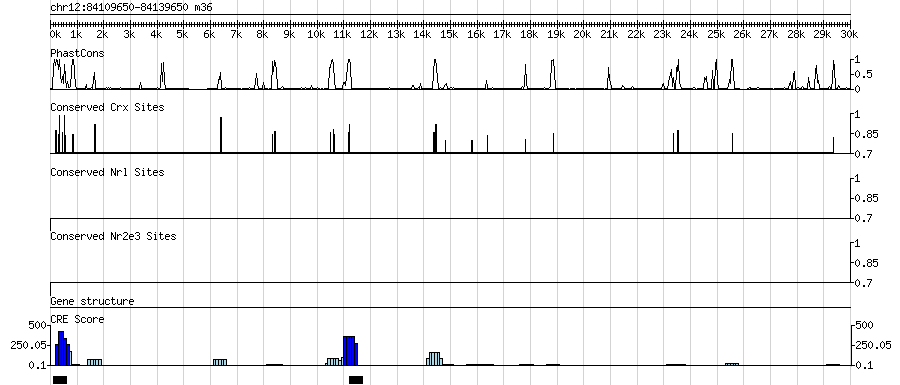

Supplement: Table S1 — Genes in the mouse photoreceptor transcription network. This table is a database which includes all genes dysregulated in Crx-/-, Nrl-/-, and/or Nr2e3-/- under high stringency criteria. Each row in the database represents a gene in the network and includes multiple links to additional types of information about that gene. ‘Large image gene +/− 15 Kb’ links to an image of our computational prediction of photoreceptor CREs in the genomic region 15 Kb upstream and downstream of the gene in question. ‘Closeup image TSS +/− 15 Kb’ links to an image of our computational prediction in the region 15 Kb on either side of the gene's TSS. Black bars within this image highlight the following regulatory peaks (if any are present): the peak closest to the TSS and the peak with the highest CRE score within this 30 Kb window. For those genes whose CRE predictions were tested experimentally, a red bar in this image indicates the location of the CRE that was tested. For Crx and Elovl4 the tested CRE is outside of this 30 Kb window and is therefore indicated in the ‘Large image’. ‘Links’ contains links to the UCSC genome browser, ENSEMBL, and NCBI database entries for the indicated gene, if available. ‘Max score (threshold of 200)’ contains the score of the highest predicted regulatory peak within the 30 Kb window around the gene's TSS. If this window does not contain any predicted peaks ≥ 200 (our cutoff threshold) no value is given (indicated by a dash). The next four columns of the table (‘Nr2e3-/-’ etc.) show the wild-type-to-mutant ratios of the averaged microarray scores for the given gene. For those genes to which more than one Affy tag correspond, the data for the first one listed under ‘Affy Mouse 430 2.0’ is given. Dark green = downregulated under high stringency (as described in METHODS); light green = downregulated under low stringency; red = upregulated under high stringency; orange = upregulated under low stringency. A dash indicates that the gene was not significantly a [file pone.0000643.s005.zip › Corbo_TableS1/images/1443306_at_TSS.gif]

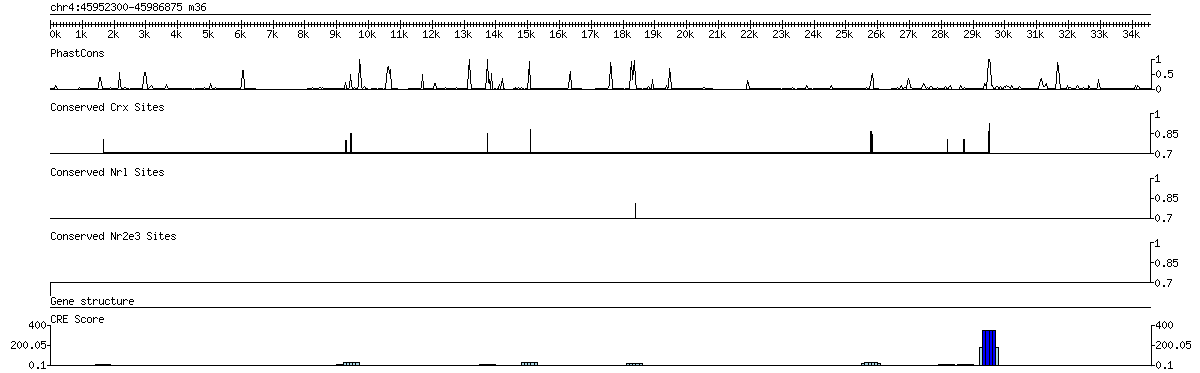

Supplement: Table S1 — Genes in the mouse photoreceptor transcription network. This table is a database which includes all genes dysregulated in Crx-/-, Nrl-/-, and/or Nr2e3-/- under high stringency criteria. Each row in the database represents a gene in the network and includes multiple links to additional types of information about that gene. ‘Large image gene +/− 15 Kb’ links to an image of our computational prediction of photoreceptor CREs in the genomic region 15 Kb upstream and downstream of the gene in question. ‘Closeup image TSS +/− 15 Kb’ links to an image of our computational prediction in the region 15 Kb on either side of the gene's TSS. Black bars within this image highlight the following regulatory peaks (if any are present): the peak closest to the TSS and the peak with the highest CRE score within this 30 Kb window. For those genes whose CRE predictions were tested experimentally, a red bar in this image indicates the location of the CRE that was tested. For Crx and Elovl4 the tested CRE is outside of this 30 Kb window and is therefore indicated in the ‘Large image’. ‘Links’ contains links to the UCSC genome browser, ENSEMBL, and NCBI database entries for the indicated gene, if available. ‘Max score (threshold of 200)’ contains the score of the highest predicted regulatory peak within the 30 Kb window around the gene's TSS. If this window does not contain any predicted peaks ≥ 200 (our cutoff threshold) no value is given (indicated by a dash). The next four columns of the table (‘Nr2e3-/-’ etc.) show the wild-type-to-mutant ratios of the averaged microarray scores for the given gene. For those genes to which more than one Affy tag correspond, the data for the first one listed under ‘Affy Mouse 430 2.0’ is given. Dark green = downregulated under high stringency (as described in METHODS); light green = downregulated under low stringency; red = upregulated under high stringency; orange = upregulated under low stringency. A dash indicates that the gene was not significantly a [file pone.0000643.s005.zip › Corbo_TableS1/images/1443552_at.gif]

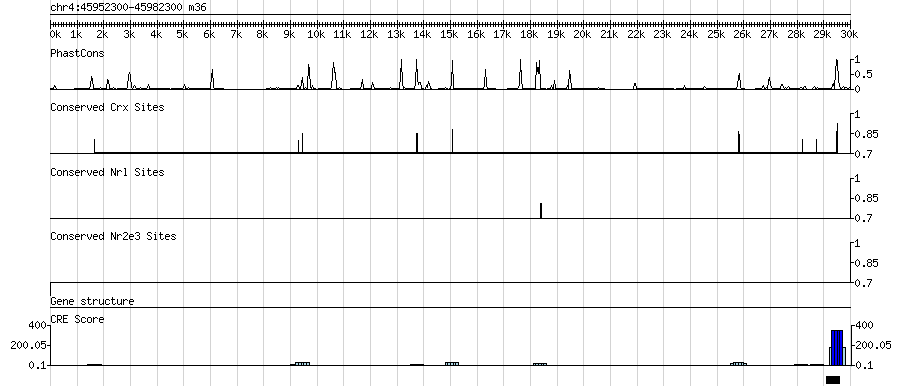

Supplement: Table S1 — Genes in the mouse photoreceptor transcription network. This table is a database which includes all genes dysregulated in Crx-/-, Nrl-/-, and/or Nr2e3-/- under high stringency criteria. Each row in the database represents a gene in the network and includes multiple links to additional types of information about that gene. ‘Large image gene +/− 15 Kb’ links to an image of our computational prediction of photoreceptor CREs in the genomic region 15 Kb upstream and downstream of the gene in question. ‘Closeup image TSS +/− 15 Kb’ links to an image of our computational prediction in the region 15 Kb on either side of the gene's TSS. Black bars within this image highlight the following regulatory peaks (if any are present): the peak closest to the TSS and the peak with the highest CRE score within this 30 Kb window. For those genes whose CRE predictions were tested experimentally, a red bar in this image indicates the location of the CRE that was tested. For Crx and Elovl4 the tested CRE is outside of this 30 Kb window and is therefore indicated in the ‘Large image’. ‘Links’ contains links to the UCSC genome browser, ENSEMBL, and NCBI database entries for the indicated gene, if available. ‘Max score (threshold of 200)’ contains the score of the highest predicted regulatory peak within the 30 Kb window around the gene's TSS. If this window does not contain any predicted peaks ≥ 200 (our cutoff threshold) no value is given (indicated by a dash). The next four columns of the table (‘Nr2e3-/-’ etc.) show the wild-type-to-mutant ratios of the averaged microarray scores for the given gene. For those genes to which more than one Affy tag correspond, the data for the first one listed under ‘Affy Mouse 430 2.0’ is given. Dark green = downregulated under high stringency (as described in METHODS); light green = downregulated under low stringency; red = upregulated under high stringency; orange = upregulated under low stringency. A dash indicates that the gene was not significantly a [file pone.0000643.s005.zip › Corbo_TableS1/images/1443552_at_TSS.gif]

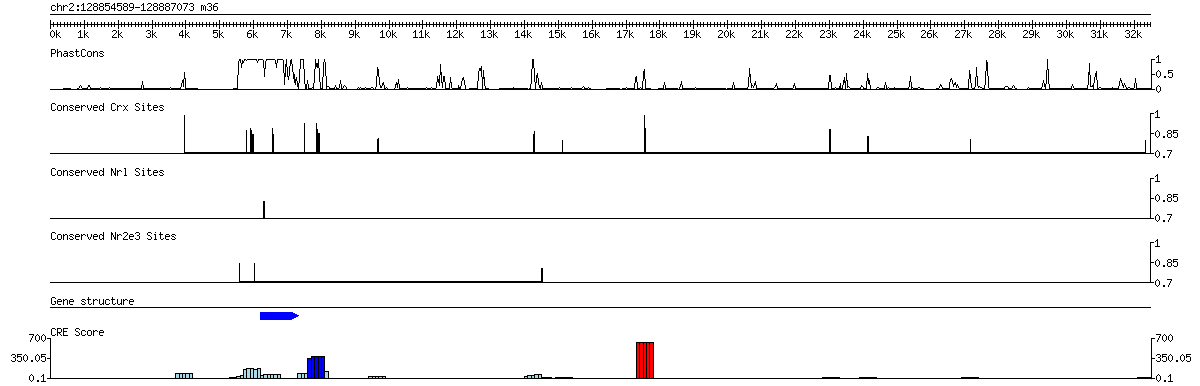

Supplement: Table S1 — Genes in the mouse photoreceptor transcription network. This table is a database which includes all genes dysregulated in Crx-/-, Nrl-/-, and/or Nr2e3-/- under high stringency criteria. Each row in the database represents a gene in the network and includes multiple links to additional types of information about that gene. ‘Large image gene +/− 15 Kb’ links to an image of our computational prediction of photoreceptor CREs in the genomic region 15 Kb upstream and downstream of the gene in question. ‘Closeup image TSS +/− 15 Kb’ links to an image of our computational prediction in the region 15 Kb on either side of the gene's TSS. Black bars within this image highlight the following regulatory peaks (if any are present): the peak closest to the TSS and the peak with the highest CRE score within this 30 Kb window. For those genes whose CRE predictions were tested experimentally, a red bar in this image indicates the location of the CRE that was tested. For Crx and Elovl4 the tested CRE is outside of this 30 Kb window and is therefore indicated in the ‘Large image’. ‘Links’ contains links to the UCSC genome browser, ENSEMBL, and NCBI database entries for the indicated gene, if available. ‘Max score (threshold of 200)’ contains the score of the highest predicted regulatory peak within the 30 Kb window around the gene's TSS. If this window does not contain any predicted peaks ≥ 200 (our cutoff threshold) no value is given (indicated by a dash). The next four columns of the table (‘Nr2e3-/-’ etc.) show the wild-type-to-mutant ratios of the averaged microarray scores for the given gene. For those genes to which more than one Affy tag correspond, the data for the first one listed under ‘Affy Mouse 430 2.0’ is given. Dark green = downregulated under high stringency (as described in METHODS); light green = downregulated under low stringency; red = upregulated under high stringency; orange = upregulated under low stringency. A dash indicates that the gene was not significantly a [file pone.0000643.s005.zip › Corbo_TableS1/images/1444095_a_at.gif]

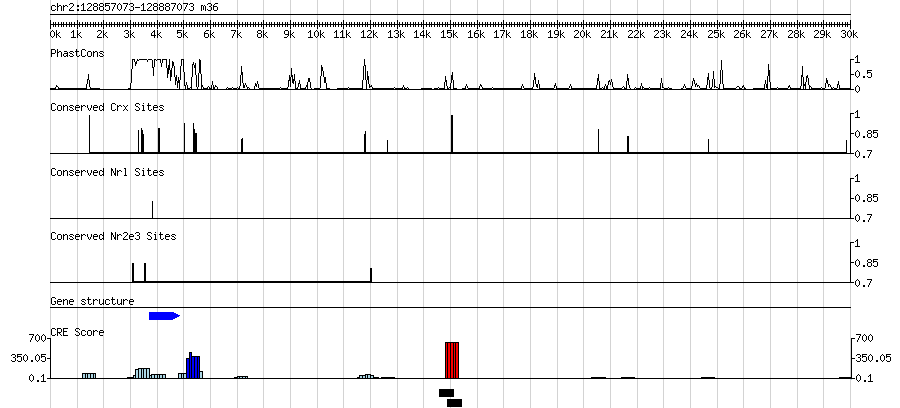

Supplement: Table S1 — Genes in the mouse photoreceptor transcription network. This table is a database which includes all genes dysregulated in Crx-/-, Nrl-/-, and/or Nr2e3-/- under high stringency criteria. Each row in the database represents a gene in the network and includes multiple links to additional types of information about that gene. ‘Large image gene +/− 15 Kb’ links to an image of our computational prediction of photoreceptor CREs in the genomic region 15 Kb upstream and downstream of the gene in question. ‘Closeup image TSS +/− 15 Kb’ links to an image of our computational prediction in the region 15 Kb on either side of the gene's TSS. Black bars within this image highlight the following regulatory peaks (if any are present): the peak closest to the TSS and the peak with the highest CRE score within this 30 Kb window. For those genes whose CRE predictions were tested experimentally, a red bar in this image indicates the location of the CRE that was tested. For Crx and Elovl4 the tested CRE is outside of this 30 Kb window and is therefore indicated in the ‘Large image’. ‘Links’ contains links to the UCSC genome browser, ENSEMBL, and NCBI database entries for the indicated gene, if available. ‘Max score (threshold of 200)’ contains the score of the highest predicted regulatory peak within the 30 Kb window around the gene's TSS. If this window does not contain any predicted peaks ≥ 200 (our cutoff threshold) no value is given (indicated by a dash). The next four columns of the table (‘Nr2e3-/-’ etc.) show the wild-type-to-mutant ratios of the averaged microarray scores for the given gene. For those genes to which more than one Affy tag correspond, the data for the first one listed under ‘Affy Mouse 430 2.0’ is given. Dark green = downregulated under high stringency (as described in METHODS); light green = downregulated under low stringency; red = upregulated under high stringency; orange = upregulated under low stringency. A dash indicates that the gene was not significantly a [file pone.0000643.s005.zip › Corbo_TableS1/images/1444095_a_at_TSS.gif]

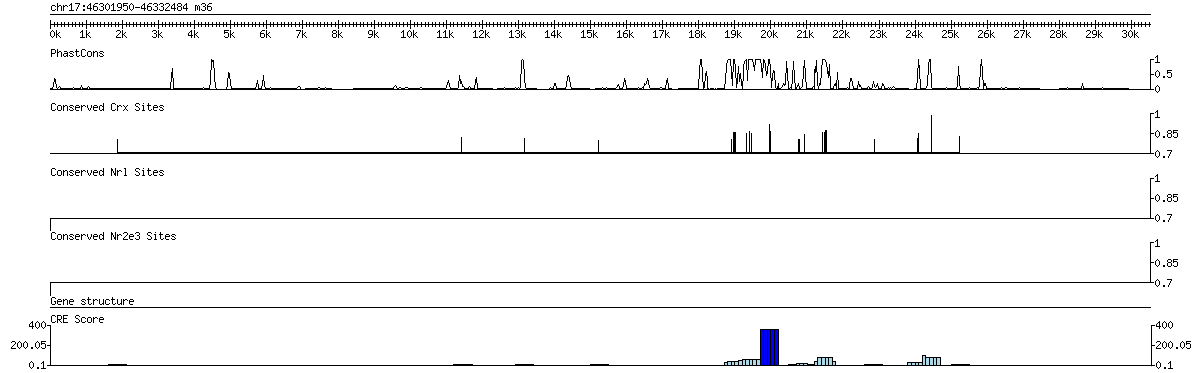

Supplement: Table S1 — Genes in the mouse photoreceptor transcription network. This table is a database which includes all genes dysregulated in Crx-/-, Nrl-/-, and/or Nr2e3-/- under high stringency criteria. Each row in the database represents a gene in the network and includes multiple links to additional types of information about that gene. ‘Large image gene +/− 15 Kb’ links to an image of our computational prediction of photoreceptor CREs in the genomic region 15 Kb upstream and downstream of the gene in question. ‘Closeup image TSS +/− 15 Kb’ links to an image of our computational prediction in the region 15 Kb on either side of the gene's TSS. Black bars within this image highlight the following regulatory peaks (if any are present): the peak closest to the TSS and the peak with the highest CRE score within this 30 Kb window. For those genes whose CRE predictions were tested experimentally, a red bar in this image indicates the location of the CRE that was tested. For Crx and Elovl4 the tested CRE is outside of this 30 Kb window and is therefore indicated in the ‘Large image’. ‘Links’ contains links to the UCSC genome browser, ENSEMBL, and NCBI database entries for the indicated gene, if available. ‘Max score (threshold of 200)’ contains the score of the highest predicted regulatory peak within the 30 Kb window around the gene's TSS. If this window does not contain any predicted peaks ≥ 200 (our cutoff threshold) no value is given (indicated by a dash). The next four columns of the table (‘Nr2e3-/-’ etc.) show the wild-type-to-mutant ratios of the averaged microarray scores for the given gene. For those genes to which more than one Affy tag correspond, the data for the first one listed under ‘Affy Mouse 430 2.0’ is given. Dark green = downregulated under high stringency (as described in METHODS); light green = downregulated under low stringency; red = upregulated under high stringency; orange = upregulated under low stringency. A dash indicates that the gene was not significantly a [file pone.0000643.s005.zip › Corbo_TableS1/images/1444552_at.gif]

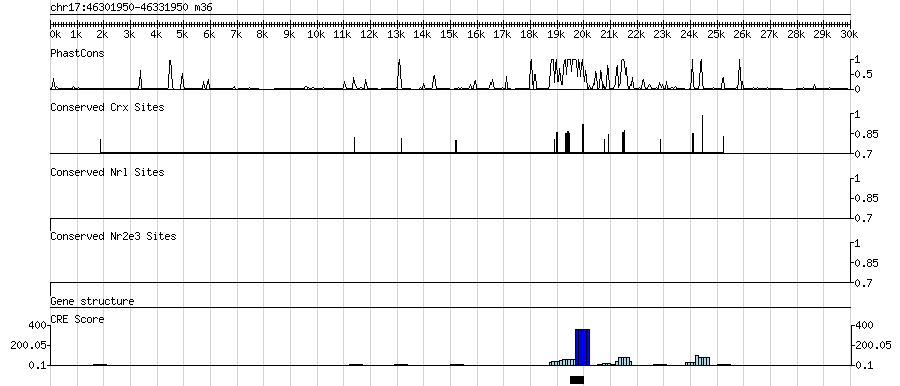

Supplement: Table S1 — Genes in the mouse photoreceptor transcription network. This table is a database which includes all genes dysregulated in Crx-/-, Nrl-/-, and/or Nr2e3-/- under high stringency criteria. Each row in the database represents a gene in the network and includes multiple links to additional types of information about that gene. ‘Large image gene +/− 15 Kb’ links to an image of our computational prediction of photoreceptor CREs in the genomic region 15 Kb upstream and downstream of the gene in question. ‘Closeup image TSS +/− 15 Kb’ links to an image of our computational prediction in the region 15 Kb on either side of the gene's TSS. Black bars within this image highlight the following regulatory peaks (if any are present): the peak closest to the TSS and the peak with the highest CRE score within this 30 Kb window. For those genes whose CRE predictions were tested experimentally, a red bar in this image indicates the location of the CRE that was tested. For Crx and Elovl4 the tested CRE is outside of this 30 Kb window and is therefore indicated in the ‘Large image’. ‘Links’ contains links to the UCSC genome browser, ENSEMBL, and NCBI database entries for the indicated gene, if available. ‘Max score (threshold of 200)’ contains the score of the highest predicted regulatory peak within the 30 Kb window around the gene's TSS. If this window does not contain any predicted peaks ≥ 200 (our cutoff threshold) no value is given (indicated by a dash). The next four columns of the table (‘Nr2e3-/-’ etc.) show the wild-type-to-mutant ratios of the averaged microarray scores for the given gene. For those genes to which more than one Affy tag correspond, the data for the first one listed under ‘Affy Mouse 430 2.0’ is given. Dark green = downregulated under high stringency (as described in METHODS); light green = downregulated under low stringency; red = upregulated under high stringency; orange = upregulated under low stringency. A dash indicates that the gene was not significantly a [file pone.0000643.s005.zip › Corbo_TableS1/images/1444552_at_TSS.gif]

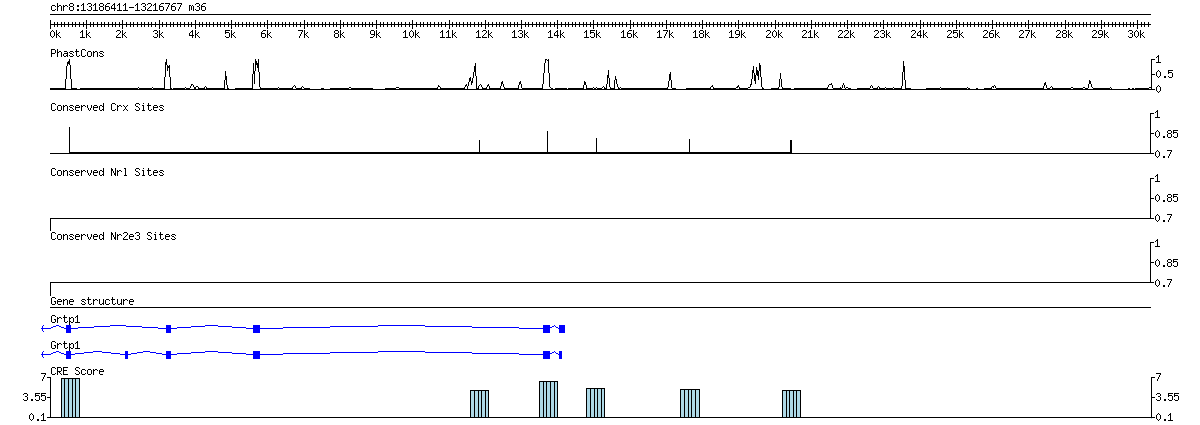

Supplement: Table S1 — Genes in the mouse photoreceptor transcription network. This table is a database which includes all genes dysregulated in Crx-/-, Nrl-/-, and/or Nr2e3-/- under high stringency criteria. Each row in the database represents a gene in the network and includes multiple links to additional types of information about that gene. ‘Large image gene +/− 15 Kb’ links to an image of our computational prediction of photoreceptor CREs in the genomic region 15 Kb upstream and downstream of the gene in question. ‘Closeup image TSS +/− 15 Kb’ links to an image of our computational prediction in the region 15 Kb on either side of the gene's TSS. Black bars within this image highlight the following regulatory peaks (if any are present): the peak closest to the TSS and the peak with the highest CRE score within this 30 Kb window. For those genes whose CRE predictions were tested experimentally, a red bar in this image indicates the location of the CRE that was tested. For Crx and Elovl4 the tested CRE is outside of this 30 Kb window and is therefore indicated in the ‘Large image’. ‘Links’ contains links to the UCSC genome browser, ENSEMBL, and NCBI database entries for the indicated gene, if available. ‘Max score (threshold of 200)’ contains the score of the highest predicted regulatory peak within the 30 Kb window around the gene's TSS. If this window does not contain any predicted peaks ≥ 200 (our cutoff threshold) no value is given (indicated by a dash). The next four columns of the table (‘Nr2e3-/-’ etc.) show the wild-type-to-mutant ratios of the averaged microarray scores for the given gene. For those genes to which more than one Affy tag correspond, the data for the first one listed under ‘Affy Mouse 430 2.0’ is given. Dark green = downregulated under high stringency (as described in METHODS); light green = downregulated under low stringency; red = upregulated under high stringency; orange = upregulated under low stringency. A dash indicates that the gene was not significantly a [file pone.0000643.s005.zip › Corbo_TableS1/images/1444603_at.gif]

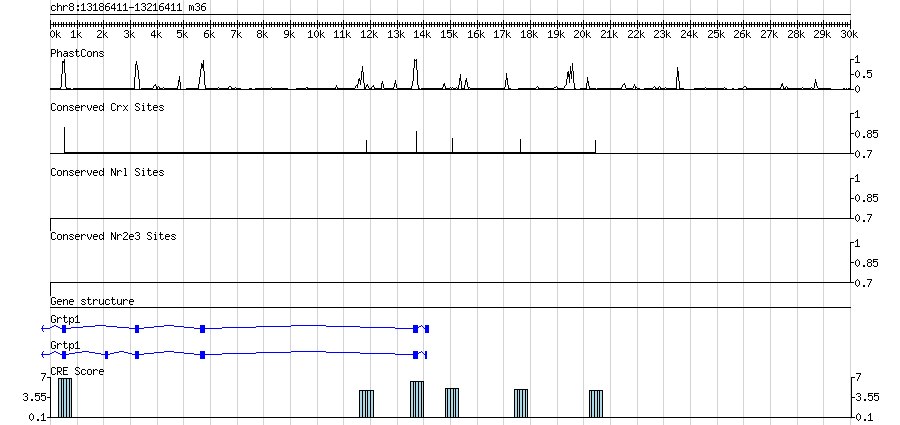

Supplement: Table S1 — Genes in the mouse photoreceptor transcription network. This table is a database which includes all genes dysregulated in Crx-/-, Nrl-/-, and/or Nr2e3-/- under high stringency criteria. Each row in the database represents a gene in the network and includes multiple links to additional types of information about that gene. ‘Large image gene +/− 15 Kb’ links to an image of our computational prediction of photoreceptor CREs in the genomic region 15 Kb upstream and downstream of the gene in question. ‘Closeup image TSS +/− 15 Kb’ links to an image of our computational prediction in the region 15 Kb on either side of the gene's TSS. Black bars within this image highlight the following regulatory peaks (if any are present): the peak closest to the TSS and the peak with the highest CRE score within this 30 Kb window. For those genes whose CRE predictions were tested experimentally, a red bar in this image indicates the location of the CRE that was tested. For Crx and Elovl4 the tested CRE is outside of this 30 Kb window and is therefore indicated in the ‘Large image’. ‘Links’ contains links to the UCSC genome browser, ENSEMBL, and NCBI database entries for the indicated gene, if available. ‘Max score (threshold of 200)’ contains the score of the highest predicted regulatory peak within the 30 Kb window around the gene's TSS. If this window does not contain any predicted peaks ≥ 200 (our cutoff threshold) no value is given (indicated by a dash). The next four columns of the table (‘Nr2e3-/-’ etc.) show the wild-type-to-mutant ratios of the averaged microarray scores for the given gene. For those genes to which more than one Affy tag correspond, the data for the first one listed under ‘Affy Mouse 430 2.0’ is given. Dark green = downregulated under high stringency (as described in METHODS); light green = downregulated under low stringency; red = upregulated under high stringency; orange = upregulated under low stringency. A dash indicates that the gene was not significantly a [file pone.0000643.s005.zip › Corbo_TableS1/images/1444603_at_TSS.gif]

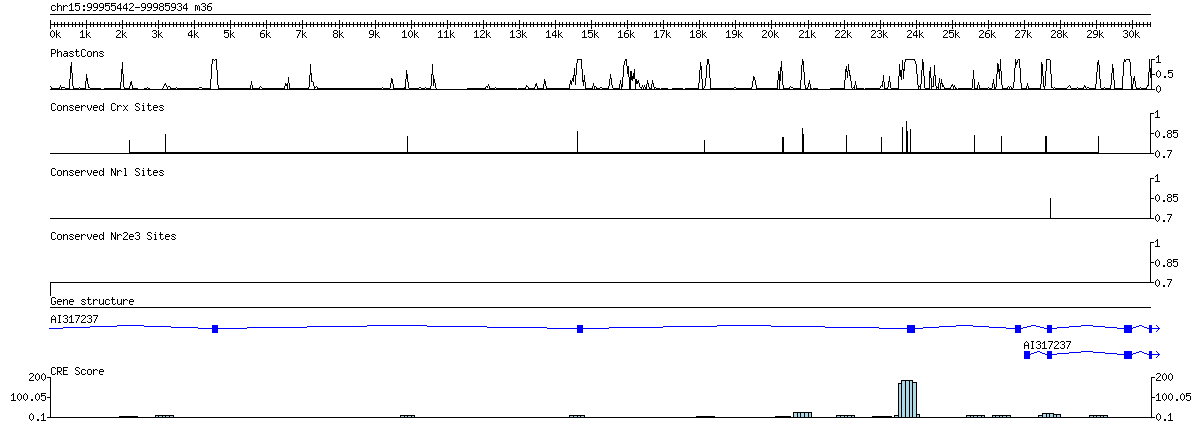

Supplement: Table S1 — Genes in the mouse photoreceptor transcription network. This table is a database which includes all genes dysregulated in Crx-/-, Nrl-/-, and/or Nr2e3-/- under high stringency criteria. Each row in the database represents a gene in the network and includes multiple links to additional types of information about that gene. ‘Large image gene +/− 15 Kb’ links to an image of our computational prediction of photoreceptor CREs in the genomic region 15 Kb upstream and downstream of the gene in question. ‘Closeup image TSS +/− 15 Kb’ links to an image of our computational prediction in the region 15 Kb on either side of the gene's TSS. Black bars within this image highlight the following regulatory peaks (if any are present): the peak closest to the TSS and the peak with the highest CRE score within this 30 Kb window. For those genes whose CRE predictions were tested experimentally, a red bar in this image indicates the location of the CRE that was tested. For Crx and Elovl4 the tested CRE is outside of this 30 Kb window and is therefore indicated in the ‘Large image’. ‘Links’ contains links to the UCSC genome browser, ENSEMBL, and NCBI database entries for the indicated gene, if available. ‘Max score (threshold of 200)’ contains the score of the highest predicted regulatory peak within the 30 Kb window around the gene's TSS. If this window does not contain any predicted peaks ≥ 200 (our cutoff threshold) no value is given (indicated by a dash). The next four columns of the table (‘Nr2e3-/-’ etc.) show the wild-type-to-mutant ratios of the averaged microarray scores for the given gene. For those genes to which more than one Affy tag correspond, the data for the first one listed under ‘Affy Mouse 430 2.0’ is given. Dark green = downregulated under high stringency (as described in METHODS); light green = downregulated under low stringency; red = upregulated under high stringency; orange = upregulated under low stringency. A dash indicates that the gene was not significantly a [file pone.0000643.s005.zip › Corbo_TableS1/images/1447191_at.gif]

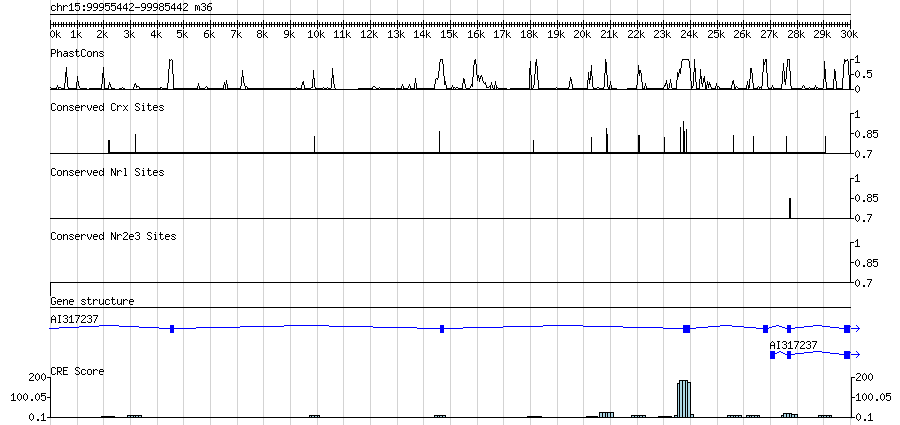

Supplement: Table S1 — Genes in the mouse photoreceptor transcription network. This table is a database which includes all genes dysregulated in Crx-/-, Nrl-/-, and/or Nr2e3-/- under high stringency criteria. Each row in the database represents a gene in the network and includes multiple links to additional types of information about that gene. ‘Large image gene +/− 15 Kb’ links to an image of our computational prediction of photoreceptor CREs in the genomic region 15 Kb upstream and downstream of the gene in question. ‘Closeup image TSS +/− 15 Kb’ links to an image of our computational prediction in the region 15 Kb on either side of the gene's TSS. Black bars within this image highlight the following regulatory peaks (if any are present): the peak closest to the TSS and the peak with the highest CRE score within this 30 Kb window. For those genes whose CRE predictions were tested experimentally, a red bar in this image indicates the location of the CRE that was tested. For Crx and Elovl4 the tested CRE is outside of this 30 Kb window and is therefore indicated in the ‘Large image’. ‘Links’ contains links to the UCSC genome browser, ENSEMBL, and NCBI database entries for the indicated gene, if available. ‘Max score (threshold of 200)’ contains the score of the highest predicted regulatory peak within the 30 Kb window around the gene's TSS. If this window does not contain any predicted peaks ≥ 200 (our cutoff threshold) no value is given (indicated by a dash). The next four columns of the table (‘Nr2e3-/-’ etc.) show the wild-type-to-mutant ratios of the averaged microarray scores for the given gene. For those genes to which more than one Affy tag correspond, the data for the first one listed under ‘Affy Mouse 430 2.0’ is given. Dark green = downregulated under high stringency (as described in METHODS); light green = downregulated under low stringency; red = upregulated under high stringency; orange = upregulated under low stringency. A dash indicates that the gene was not significantly a [file pone.0000643.s005.zip › Corbo_TableS1/images/1447191_at_TSS.gif]

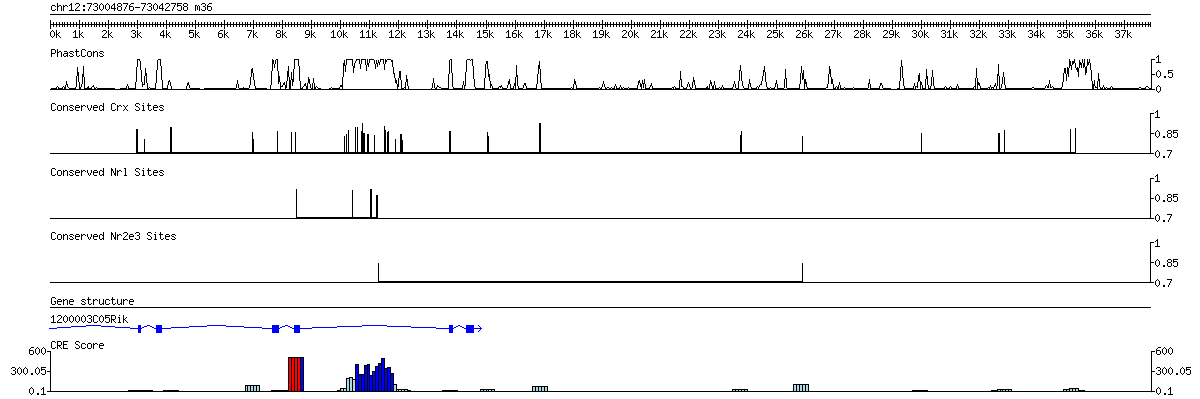

Supplement: Table S1 — Genes in the mouse photoreceptor transcription network. This table is a database which includes all genes dysregulated in Crx-/-, Nrl-/-, and/or Nr2e3-/- under high stringency criteria. Each row in the database represents a gene in the network and includes multiple links to additional types of information about that gene. ‘Large image gene +/− 15 Kb’ links to an image of our computational prediction of photoreceptor CREs in the genomic region 15 Kb upstream and downstream of the gene in question. ‘Closeup image TSS +/− 15 Kb’ links to an image of our computational prediction in the region 15 Kb on either side of the gene's TSS. Black bars within this image highlight the following regulatory peaks (if any are present): the peak closest to the TSS and the peak with the highest CRE score within this 30 Kb window. For those genes whose CRE predictions were tested experimentally, a red bar in this image indicates the location of the CRE that was tested. For Crx and Elovl4 the tested CRE is outside of this 30 Kb window and is therefore indicated in the ‘Large image’. ‘Links’ contains links to the UCSC genome browser, ENSEMBL, and NCBI database entries for the indicated gene, if available. ‘Max score (threshold of 200)’ contains the score of the highest predicted regulatory peak within the 30 Kb window around the gene's TSS. If this window does not contain any predicted peaks ≥ 200 (our cutoff threshold) no value is given (indicated by a dash). The next four columns of the table (‘Nr2e3-/-’ etc.) show the wild-type-to-mutant ratios of the averaged microarray scores for the given gene. For those genes to which more than one Affy tag correspond, the data for the first one listed under ‘Affy Mouse 430 2.0’ is given. Dark green = downregulated under high stringency (as described in METHODS); light green = downregulated under low stringency; red = upregulated under high stringency; orange = upregulated under low stringency. A dash indicates that the gene was not significantly a [file pone.0000643.s005.zip › Corbo_TableS1/images/1456101_at.gif]

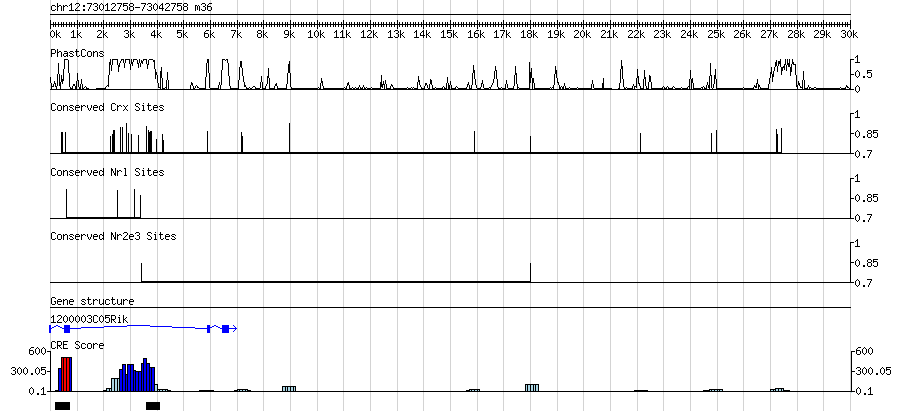

Supplement: Table S1 — Genes in the mouse photoreceptor transcription network. This table is a database which includes all genes dysregulated in Crx-/-, Nrl-/-, and/or Nr2e3-/- under high stringency criteria. Each row in the database represents a gene in the network and includes multiple links to additional types of information about that gene. ‘Large image gene +/− 15 Kb’ links to an image of our computational prediction of photoreceptor CREs in the genomic region 15 Kb upstream and downstream of the gene in question. ‘Closeup image TSS +/− 15 Kb’ links to an image of our computational prediction in the region 15 Kb on either side of the gene's TSS. Black bars within this image highlight the following regulatory peaks (if any are present): the peak closest to the TSS and the peak with the highest CRE score within this 30 Kb window. For those genes whose CRE predictions were tested experimentally, a red bar in this image indicates the location of the CRE that was tested. For Crx and Elovl4 the tested CRE is outside of this 30 Kb window and is therefore indicated in the ‘Large image’. ‘Links’ contains links to the UCSC genome browser, ENSEMBL, and NCBI database entries for the indicated gene, if available. ‘Max score (threshold of 200)’ contains the score of the highest predicted regulatory peak within the 30 Kb window around the gene's TSS. If this window does not contain any predicted peaks ≥ 200 (our cutoff threshold) no value is given (indicated by a dash). The next four columns of the table (‘Nr2e3-/-’ etc.) show the wild-type-to-mutant ratios of the averaged microarray scores for the given gene. For those genes to which more than one Affy tag correspond, the data for the first one listed under ‘Affy Mouse 430 2.0’ is given. Dark green = downregulated under high stringency (as described in METHODS); light green = downregulated under low stringency; red = upregulated under high stringency; orange = upregulated under low stringency. A dash indicates that the gene was not significantly a [file pone.0000643.s005.zip › Corbo_TableS1/images/1456101_at_TSS.gif]

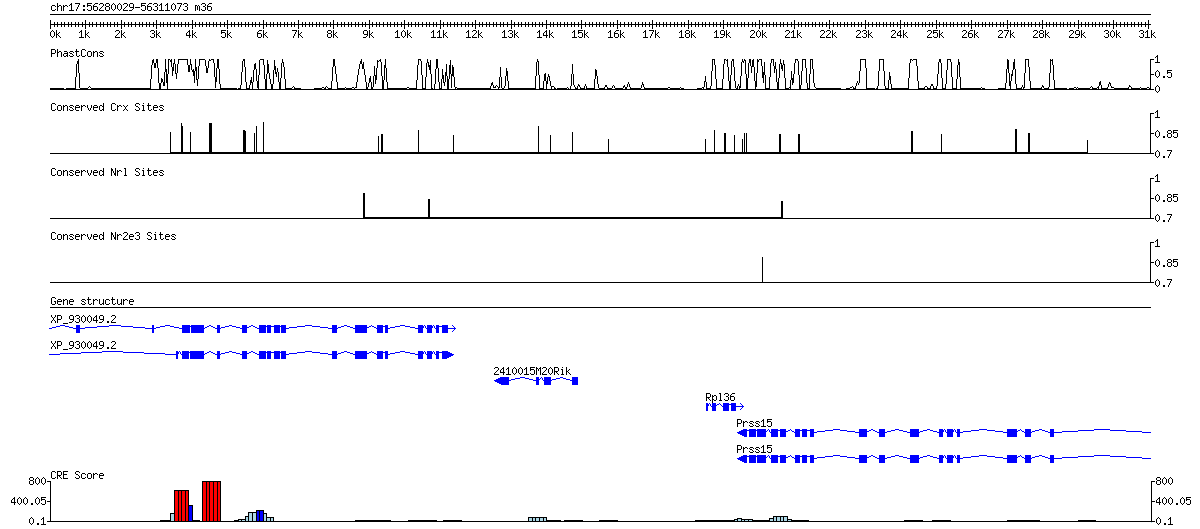

Supplement: Table S1 — Genes in the mouse photoreceptor transcription network. This table is a database which includes all genes dysregulated in Crx-/-, Nrl-/-, and/or Nr2e3-/- under high stringency criteria. Each row in the database represents a gene in the network and includes multiple links to additional types of information about that gene. ‘Large image gene +/− 15 Kb’ links to an image of our computational prediction of photoreceptor CREs in the genomic region 15 Kb upstream and downstream of the gene in question. ‘Closeup image TSS +/− 15 Kb’ links to an image of our computational prediction in the region 15 Kb on either side of the gene's TSS. Black bars within this image highlight the following regulatory peaks (if any are present): the peak closest to the TSS and the peak with the highest CRE score within this 30 Kb window. For those genes whose CRE predictions were tested experimentally, a red bar in this image indicates the location of the CRE that was tested. For Crx and Elovl4 the tested CRE is outside of this 30 Kb window and is therefore indicated in the ‘Large image’. ‘Links’ contains links to the UCSC genome browser, ENSEMBL, and NCBI database entries for the indicated gene, if available. ‘Max score (threshold of 200)’ contains the score of the highest predicted regulatory peak within the 30 Kb window around the gene's TSS. If this window does not contain any predicted peaks ≥ 200 (our cutoff threshold) no value is given (indicated by a dash). The next four columns of the table (‘Nr2e3-/-’ etc.) show the wild-type-to-mutant ratios of the averaged microarray scores for the given gene. For those genes to which more than one Affy tag correspond, the data for the first one listed under ‘Affy Mouse 430 2.0’ is given. Dark green = downregulated under high stringency (as described in METHODS); light green = downregulated under low stringency; red = upregulated under high stringency; orange = upregulated under low stringency. A dash indicates that the gene was not significantly a [file pone.0000643.s005.zip › Corbo_TableS1/images/1457284_at.gif]

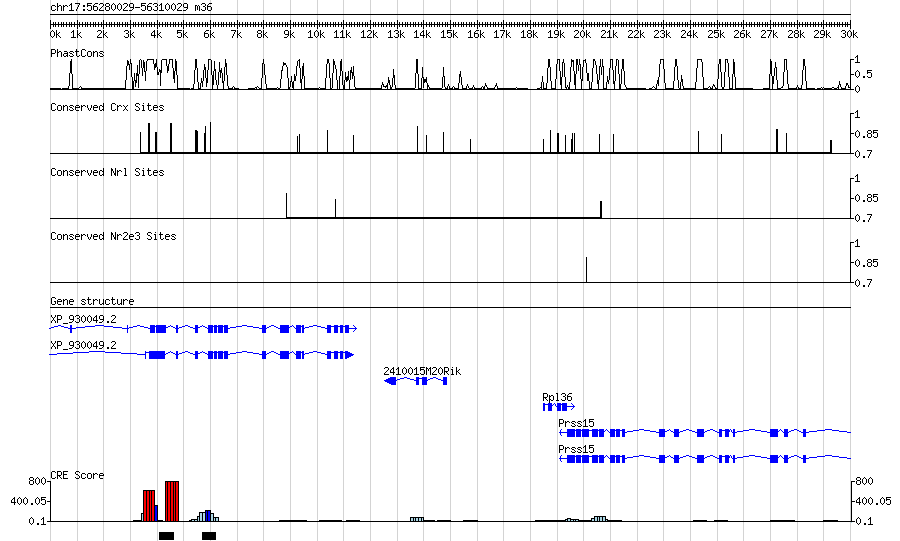

Supplement: Table S1 — Genes in the mouse photoreceptor transcription network. This table is a database which includes all genes dysregulated in Crx-/-, Nrl-/-, and/or Nr2e3-/- under high stringency criteria. Each row in the database represents a gene in the network and includes multiple links to additional types of information about that gene. ‘Large image gene +/− 15 Kb’ links to an image of our computational prediction of photoreceptor CREs in the genomic region 15 Kb upstream and downstream of the gene in question. ‘Closeup image TSS +/− 15 Kb’ links to an image of our computational prediction in the region 15 Kb on either side of the gene's TSS. Black bars within this image highlight the following regulatory peaks (if any are present): the peak closest to the TSS and the peak with the highest CRE score within this 30 Kb window. For those genes whose CRE predictions were tested experimentally, a red bar in this image indicates the location of the CRE that was tested. For Crx and Elovl4 the tested CRE is outside of this 30 Kb window and is therefore indicated in the ‘Large image’. ‘Links’ contains links to the UCSC genome browser, ENSEMBL, and NCBI database entries for the indicated gene, if available. ‘Max score (threshold of 200)’ contains the score of the highest predicted regulatory peak within the 30 Kb window around the gene's TSS. If this window does not contain any predicted peaks ≥ 200 (our cutoff threshold) no value is given (indicated by a dash). The next four columns of the table (‘Nr2e3-/-’ etc.) show the wild-type-to-mutant ratios of the averaged microarray scores for the given gene. For those genes to which more than one Affy tag correspond, the data for the first one listed under ‘Affy Mouse 430 2.0’ is given. Dark green = downregulated under high stringency (as described in METHODS); light green = downregulated under low stringency; red = upregulated under high stringency; orange = upregulated under low stringency. A dash indicates that the gene was not significantly a [file pone.0000643.s005.zip › Corbo_TableS1/images/1457284_at_TSS.gif]

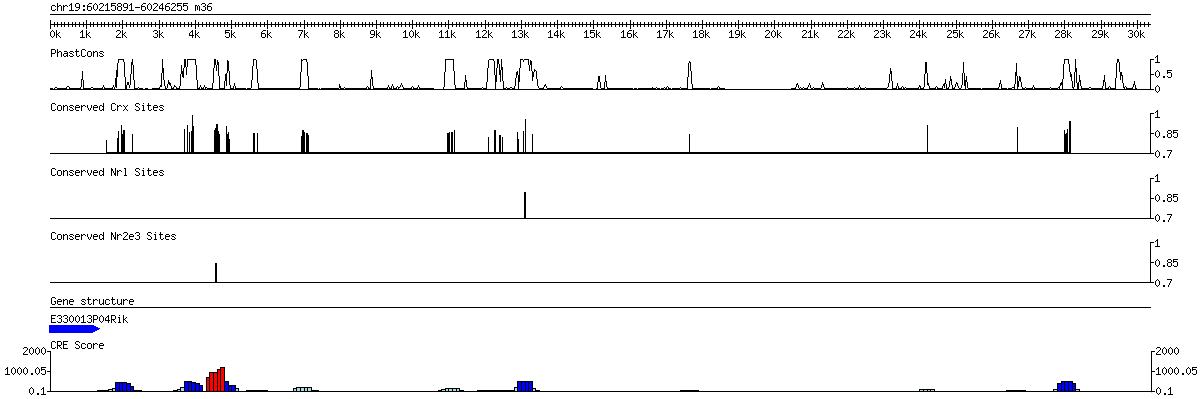

Supplement: Table S1 — Genes in the mouse photoreceptor transcription network. This table is a database which includes all genes dysregulated in Crx-/-, Nrl-/-, and/or Nr2e3-/- under high stringency criteria. Each row in the database represents a gene in the network and includes multiple links to additional types of information about that gene. ‘Large image gene +/− 15 Kb’ links to an image of our computational prediction of photoreceptor CREs in the genomic region 15 Kb upstream and downstream of the gene in question. ‘Closeup image TSS +/− 15 Kb’ links to an image of our computational prediction in the region 15 Kb on either side of the gene's TSS. Black bars within this image highlight the following regulatory peaks (if any are present): the peak closest to the TSS and the peak with the highest CRE score within this 30 Kb window. For those genes whose CRE predictions were tested experimentally, a red bar in this image indicates the location of the CRE that was tested. For Crx and Elovl4 the tested CRE is outside of this 30 Kb window and is therefore indicated in the ‘Large image’. ‘Links’ contains links to the UCSC genome browser, ENSEMBL, and NCBI database entries for the indicated gene, if available. ‘Max score (threshold of 200)’ contains the score of the highest predicted regulatory peak within the 30 Kb window around the gene's TSS. If this window does not contain any predicted peaks ≥ 200 (our cutoff threshold) no value is given (indicated by a dash). The next four columns of the table (‘Nr2e3-/-’ etc.) show the wild-type-to-mutant ratios of the averaged microarray scores for the given gene. For those genes to which more than one Affy tag correspond, the data for the first one listed under ‘Affy Mouse 430 2.0’ is given. Dark green = downregulated under high stringency (as described in METHODS); light green = downregulated under low stringency; red = upregulated under high stringency; orange = upregulated under low stringency. A dash indicates that the gene was not significantly a [file pone.0000643.s005.zip › Corbo_TableS1/images/1459721_at.gif]

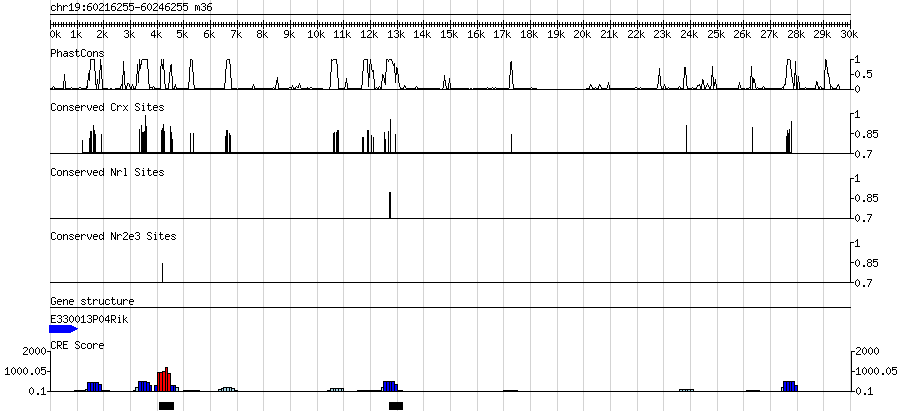

Supplement: Table S1 — Genes in the mouse photoreceptor transcription network. This table is a database which includes all genes dysregulated in Crx-/-, Nrl-/-, and/or Nr2e3-/- under high stringency criteria. Each row in the database represents a gene in the network and includes multiple links to additional types of information about that gene. ‘Large image gene +/− 15 Kb’ links to an image of our computational prediction of photoreceptor CREs in the genomic region 15 Kb upstream and downstream of the gene in question. ‘Closeup image TSS +/− 15 Kb’ links to an image of our computational prediction in the region 15 Kb on either side of the gene's TSS. Black bars within this image highlight the following regulatory peaks (if any are present): the peak closest to the TSS and the peak with the highest CRE score within this 30 Kb window. For those genes whose CRE predictions were tested experimentally, a red bar in this image indicates the location of the CRE that was tested. For Crx and Elovl4 the tested CRE is outside of this 30 Kb window and is therefore indicated in the ‘Large image’. ‘Links’ contains links to the UCSC genome browser, ENSEMBL, and NCBI database entries for the indicated gene, if available. ‘Max score (threshold of 200)’ contains the score of the highest predicted regulatory peak within the 30 Kb window around the gene's TSS. If this window does not contain any predicted peaks ≥ 200 (our cutoff threshold) no value is given (indicated by a dash). The next four columns of the table (‘Nr2e3-/-’ etc.) show the wild-type-to-mutant ratios of the averaged microarray scores for the given gene. For those genes to which more than one Affy tag correspond, the data for the first one listed under ‘Affy Mouse 430 2.0’ is given. Dark green = downregulated under high stringency (as described in METHODS); light green = downregulated under low stringency; red = upregulated under high stringency; orange = upregulated under low stringency. A dash indicates that the gene was not significantly a [file pone.0000643.s005.zip › Corbo_TableS1/images/1459721_at_TSS.gif]

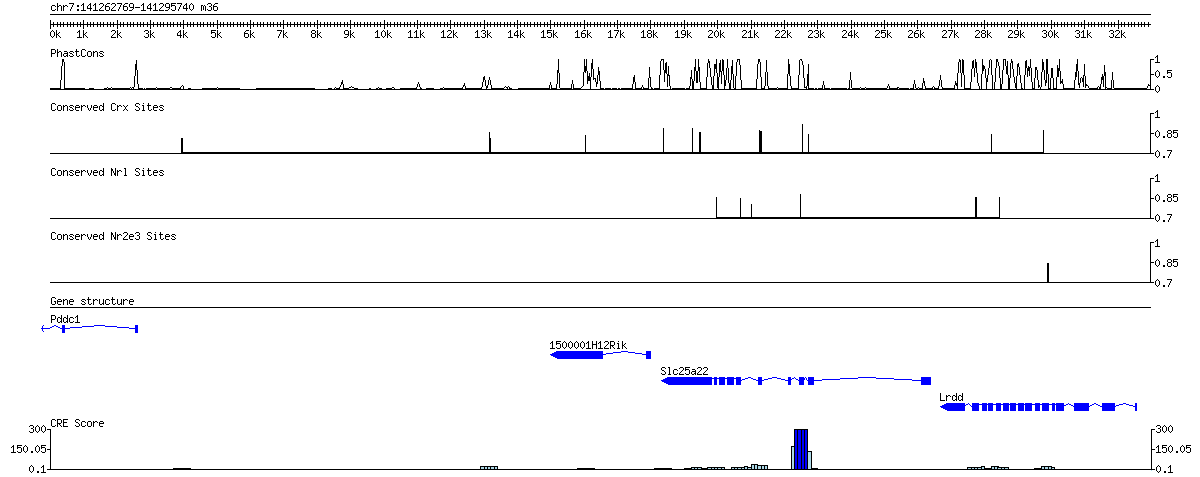

Supplement: Table S1 — Genes in the mouse photoreceptor transcription network. This table is a database which includes all genes dysregulated in Crx-/-, Nrl-/-, and/or Nr2e3-/- under high stringency criteria. Each row in the database represents a gene in the network and includes multiple links to additional types of information about that gene. ‘Large image gene +/− 15 Kb’ links to an image of our computational prediction of photoreceptor CREs in the genomic region 15 Kb upstream and downstream of the gene in question. ‘Closeup image TSS +/− 15 Kb’ links to an image of our computational prediction in the region 15 Kb on either side of the gene's TSS. Black bars within this image highlight the following regulatory peaks (if any are present): the peak closest to the TSS and the peak with the highest CRE score within this 30 Kb window. For those genes whose CRE predictions were tested experimentally, a red bar in this image indicates the location of the CRE that was tested. For Crx and Elovl4 the tested CRE is outside of this 30 Kb window and is therefore indicated in the ‘Large image’. ‘Links’ contains links to the UCSC genome browser, ENSEMBL, and NCBI database entries for the indicated gene, if available. ‘Max score (threshold of 200)’ contains the score of the highest predicted regulatory peak within the 30 Kb window around the gene's TSS. If this window does not contain any predicted peaks ≥ 200 (our cutoff threshold) no value is given (indicated by a dash). The next four columns of the table (‘Nr2e3-/-’ etc.) show the wild-type-to-mutant ratios of the averaged microarray scores for the given gene. For those genes to which more than one Affy tag correspond, the data for the first one listed under ‘Affy Mouse 430 2.0’ is given. Dark green = downregulated under high stringency (as described in METHODS); light green = downregulated under low stringency; red = upregulated under high stringency; orange = upregulated under low stringency. A dash indicates that the gene was not significantly a [file pone.0000643.s005.zip › Corbo_TableS1/images/1500001H12Rik.gif]

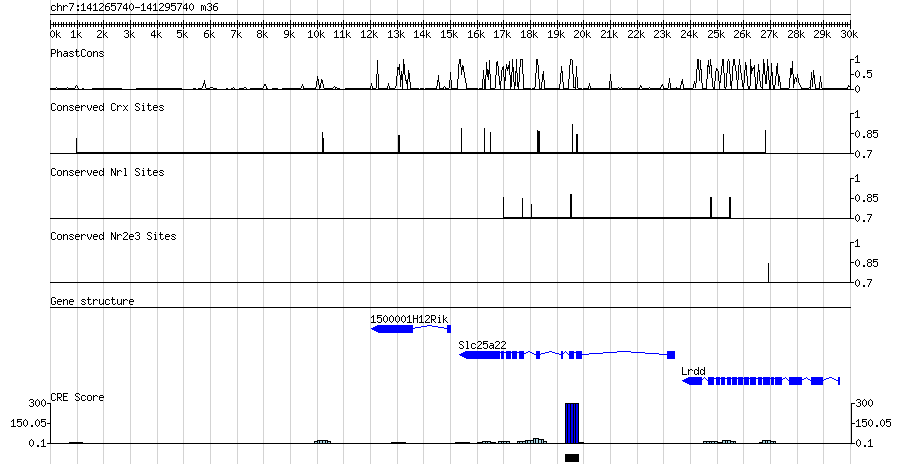

Supplement: Table S1 — Genes in the mouse photoreceptor transcription network. This table is a database which includes all genes dysregulated in Crx-/-, Nrl-/-, and/or Nr2e3-/- under high stringency criteria. Each row in the database represents a gene in the network and includes multiple links to additional types of information about that gene. ‘Large image gene +/− 15 Kb’ links to an image of our computational prediction of photoreceptor CREs in the genomic region 15 Kb upstream and downstream of the gene in question. ‘Closeup image TSS +/− 15 Kb’ links to an image of our computational prediction in the region 15 Kb on either side of the gene's TSS. Black bars within this image highlight the following regulatory peaks (if any are present): the peak closest to the TSS and the peak with the highest CRE score within this 30 Kb window. For those genes whose CRE predictions were tested experimentally, a red bar in this image indicates the location of the CRE that was tested. For Crx and Elovl4 the tested CRE is outside of this 30 Kb window and is therefore indicated in the ‘Large image’. ‘Links’ contains links to the UCSC genome browser, ENSEMBL, and NCBI database entries for the indicated gene, if available. ‘Max score (threshold of 200)’ contains the score of the highest predicted regulatory peak within the 30 Kb window around the gene's TSS. If this window does not contain any predicted peaks ≥ 200 (our cutoff threshold) no value is given (indicated by a dash). The next four columns of the table (‘Nr2e3-/-’ etc.) show the wild-type-to-mutant ratios of the averaged microarray scores for the given gene. For those genes to which more than one Affy tag correspond, the data for the first one listed under ‘Affy Mouse 430 2.0’ is given. Dark green = downregulated under high stringency (as described in METHODS); light green = downregulated under low stringency; red = upregulated under high stringency; orange = upregulated under low stringency. A dash indicates that the gene was not significantly a [file pone.0000643.s005.zip › Corbo_TableS1/images/1500001H12Rik_TSS.gif]

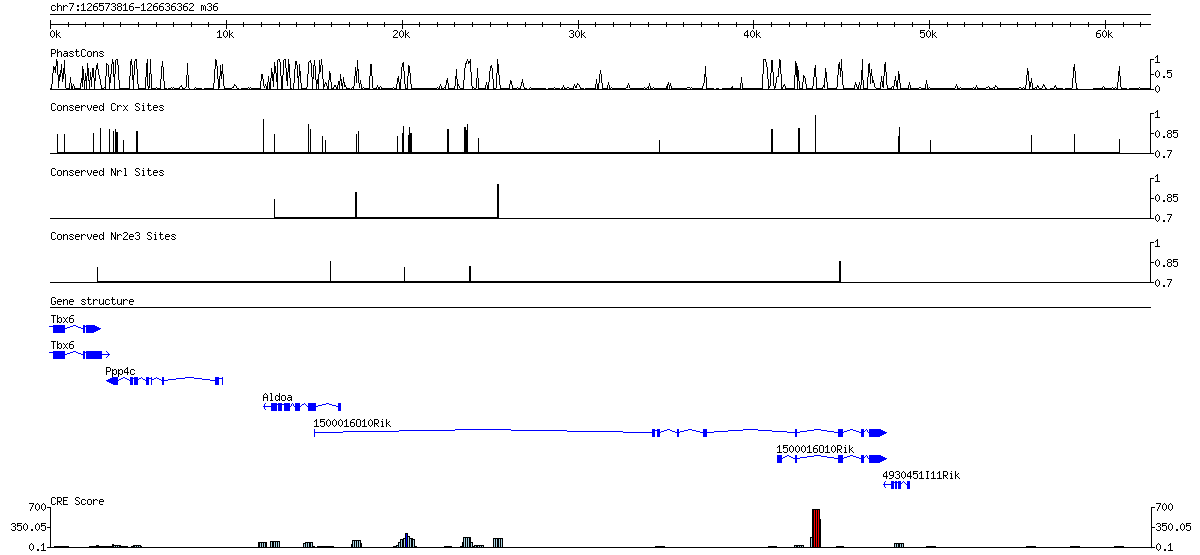

Supplement: Table S1 — Genes in the mouse photoreceptor transcription network. This table is a database which includes all genes dysregulated in Crx-/-, Nrl-/-, and/or Nr2e3-/- under high stringency criteria. Each row in the database represents a gene in the network and includes multiple links to additional types of information about that gene. ‘Large image gene +/− 15 Kb’ links to an image of our computational prediction of photoreceptor CREs in the genomic region 15 Kb upstream and downstream of the gene in question. ‘Closeup image TSS +/− 15 Kb’ links to an image of our computational prediction in the region 15 Kb on either side of the gene's TSS. Black bars within this image highlight the following regulatory peaks (if any are present): the peak closest to the TSS and the peak with the highest CRE score within this 30 Kb window. For those genes whose CRE predictions were tested experimentally, a red bar in this image indicates the location of the CRE that was tested. For Crx and Elovl4 the tested CRE is outside of this 30 Kb window and is therefore indicated in the ‘Large image’. ‘Links’ contains links to the UCSC genome browser, ENSEMBL, and NCBI database entries for the indicated gene, if available. ‘Max score (threshold of 200)’ contains the score of the highest predicted regulatory peak within the 30 Kb window around the gene's TSS. If this window does not contain any predicted peaks ≥ 200 (our cutoff threshold) no value is given (indicated by a dash). The next four columns of the table (‘Nr2e3-/-’ etc.) show the wild-type-to-mutant ratios of the averaged microarray scores for the given gene. For those genes to which more than one Affy tag correspond, the data for the first one listed under ‘Affy Mouse 430 2.0’ is given. Dark green = downregulated under high stringency (as described in METHODS); light green = downregulated under low stringency; red = upregulated under high stringency; orange = upregulated under low stringency. A dash indicates that the gene was not significantly a [file pone.0000643.s005.zip › Corbo_TableS1/images/1500016O10Rik.gif]

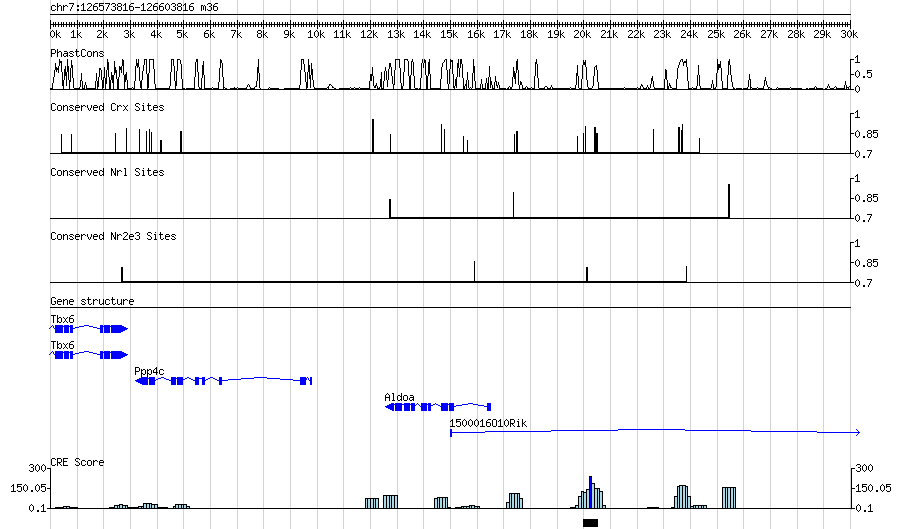

Supplement: Table S1 — Genes in the mouse photoreceptor transcription network. This table is a database which includes all genes dysregulated in Crx-/-, Nrl-/-, and/or Nr2e3-/- under high stringency criteria. Each row in the database represents a gene in the network and includes multiple links to additional types of information about that gene. ‘Large image gene +/− 15 Kb’ links to an image of our computational prediction of photoreceptor CREs in the genomic region 15 Kb upstream and downstream of the gene in question. ‘Closeup image TSS +/− 15 Kb’ links to an image of our computational prediction in the region 15 Kb on either side of the gene's TSS. Black bars within this image highlight the following regulatory peaks (if any are present): the peak closest to the TSS and the peak with the highest CRE score within this 30 Kb window. For those genes whose CRE predictions were tested experimentally, a red bar in this image indicates the location of the CRE that was tested. For Crx and Elovl4 the tested CRE is outside of this 30 Kb window and is therefore indicated in the ‘Large image’. ‘Links’ contains links to the UCSC genome browser, ENSEMBL, and NCBI database entries for the indicated gene, if available. ‘Max score (threshold of 200)’ contains the score of the highest predicted regulatory peak within the 30 Kb window around the gene's TSS. If this window does not contain any predicted peaks ≥ 200 (our cutoff threshold) no value is given (indicated by a dash). The next four columns of the table (‘Nr2e3-/-’ etc.) show the wild-type-to-mutant ratios of the averaged microarray scores for the given gene. For those genes to which more than one Affy tag correspond, the data for the first one listed under ‘Affy Mouse 430 2.0’ is given. Dark green = downregulated under high stringency (as described in METHODS); light green = downregulated under low stringency; red = upregulated under high stringency; orange = upregulated under low stringency. A dash indicates that the gene was not significantly a [file pone.0000643.s005.zip › Corbo_TableS1/images/1500016O10Rik_TSS.gif]

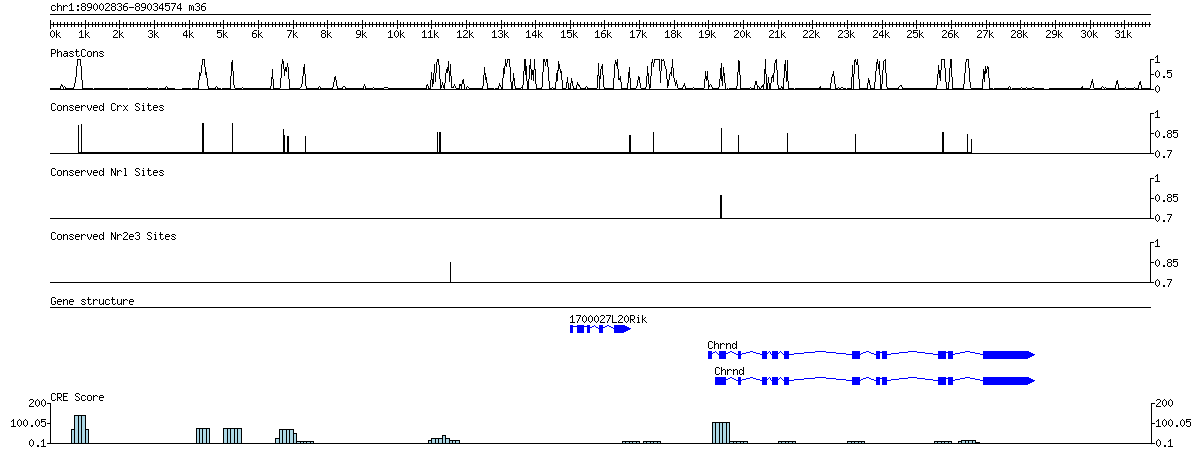

Supplement: Table S1 — Genes in the mouse photoreceptor transcription network. This table is a database which includes all genes dysregulated in Crx-/-, Nrl-/-, and/or Nr2e3-/- under high stringency criteria. Each row in the database represents a gene in the network and includes multiple links to additional types of information about that gene. ‘Large image gene +/− 15 Kb’ links to an image of our computational prediction of photoreceptor CREs in the genomic region 15 Kb upstream and downstream of the gene in question. ‘Closeup image TSS +/− 15 Kb’ links to an image of our computational prediction in the region 15 Kb on either side of the gene's TSS. Black bars within this image highlight the following regulatory peaks (if any are present): the peak closest to the TSS and the peak with the highest CRE score within this 30 Kb window. For those genes whose CRE predictions were tested experimentally, a red bar in this image indicates the location of the CRE that was tested. For Crx and Elovl4 the tested CRE is outside of this 30 Kb window and is therefore indicated in the ‘Large image’. ‘Links’ contains links to the UCSC genome browser, ENSEMBL, and NCBI database entries for the indicated gene, if available. ‘Max score (threshold of 200)’ contains the score of the highest predicted regulatory peak within the 30 Kb window around the gene's TSS. If this window does not contain any predicted peaks ≥ 200 (our cutoff threshold) no value is given (indicated by a dash). The next four columns of the table (‘Nr2e3-/-’ etc.) show the wild-type-to-mutant ratios of the averaged microarray scores for the given gene. For those genes to which more than one Affy tag correspond, the data for the first one listed under ‘Affy Mouse 430 2.0’ is given. Dark green = downregulated under high stringency (as described in METHODS); light green = downregulated under low stringency; red = upregulated under high stringency; orange = upregulated under low stringency. A dash indicates that the gene was not significantly a [file pone.0000643.s005.zip › Corbo_TableS1/images/1700027L20Rik.gif]

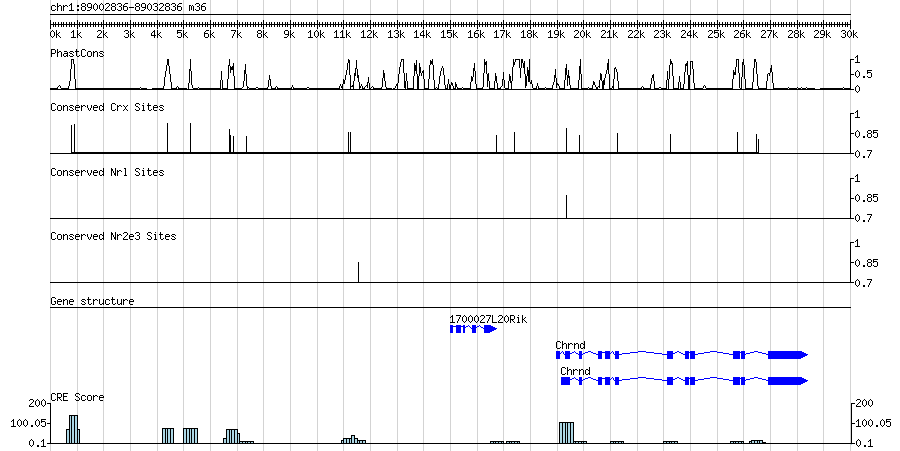

Supplement: Table S1 — Genes in the mouse photoreceptor transcription network. This table is a database which includes all genes dysregulated in Crx-/-, Nrl-/-, and/or Nr2e3-/- under high stringency criteria. Each row in the database represents a gene in the network and includes multiple links to additional types of information about that gene. ‘Large image gene +/− 15 Kb’ links to an image of our computational prediction of photoreceptor CREs in the genomic region 15 Kb upstream and downstream of the gene in question. ‘Closeup image TSS +/− 15 Kb’ links to an image of our computational prediction in the region 15 Kb on either side of the gene's TSS. Black bars within this image highlight the following regulatory peaks (if any are present): the peak closest to the TSS and the peak with the highest CRE score within this 30 Kb window. For those genes whose CRE predictions were tested experimentally, a red bar in this image indicates the location of the CRE that was tested. For Crx and Elovl4 the tested CRE is outside of this 30 Kb window and is therefore indicated in the ‘Large image’. ‘Links’ contains links to the UCSC genome browser, ENSEMBL, and NCBI database entries for the indicated gene, if available. ‘Max score (threshold of 200)’ contains the score of the highest predicted regulatory peak within the 30 Kb window around the gene's TSS. If this window does not contain any predicted peaks ≥ 200 (our cutoff threshold) no value is given (indicated by a dash). The next four columns of the table (‘Nr2e3-/-’ etc.) show the wild-type-to-mutant ratios of the averaged microarray scores for the given gene. For those genes to which more than one Affy tag correspond, the data for the first one listed under ‘Affy Mouse 430 2.0’ is given. Dark green = downregulated under high stringency (as described in METHODS); light green = downregulated under low stringency; red = upregulated under high stringency; orange = upregulated under low stringency. A dash indicates that the gene was not significantly a [file pone.0000643.s005.zip › Corbo_TableS1/images/1700027L20Rik_TSS.gif]

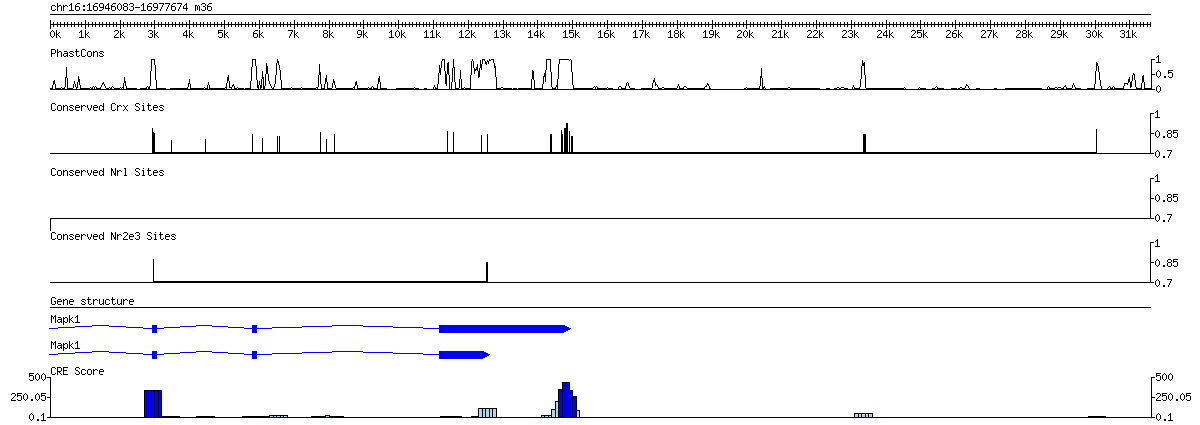

Supplement: Table S1 — Genes in the mouse photoreceptor transcription network. This table is a database which includes all genes dysregulated in Crx-/-, Nrl-/-, and/or Nr2e3-/- under high stringency criteria. Each row in the database represents a gene in the network and includes multiple links to additional types of information about that gene. ‘Large image gene +/− 15 Kb’ links to an image of our computational prediction of photoreceptor CREs in the genomic region 15 Kb upstream and downstream of the gene in question. ‘Closeup image TSS +/− 15 Kb’ links to an image of our computational prediction in the region 15 Kb on either side of the gene's TSS. Black bars within this image highlight the following regulatory peaks (if any are present): the peak closest to the TSS and the peak with the highest CRE score within this 30 Kb window. For those genes whose CRE predictions were tested experimentally, a red bar in this image indicates the location of the CRE that was tested. For Crx and Elovl4 the tested CRE is outside of this 30 Kb window and is therefore indicated in the ‘Large image’. ‘Links’ contains links to the UCSC genome browser, ENSEMBL, and NCBI database entries for the indicated gene, if available. ‘Max score (threshold of 200)’ contains the score of the highest predicted regulatory peak within the 30 Kb window around the gene's TSS. If this window does not contain any predicted peaks ≥ 200 (our cutoff threshold) no value is given (indicated by a dash). The next four columns of the table (‘Nr2e3-/-’ etc.) show the wild-type-to-mutant ratios of the averaged microarray scores for the given gene. For those genes to which more than one Affy tag correspond, the data for the first one listed under ‘Affy Mouse 430 2.0’ is given. Dark green = downregulated under high stringency (as described in METHODS); light green = downregulated under low stringency; red = upregulated under high stringency; orange = upregulated under low stringency. A dash indicates that the gene was not significantly a [file pone.0000643.s005.zip › Corbo_TableS1/images/1700056N10Rik.gif]

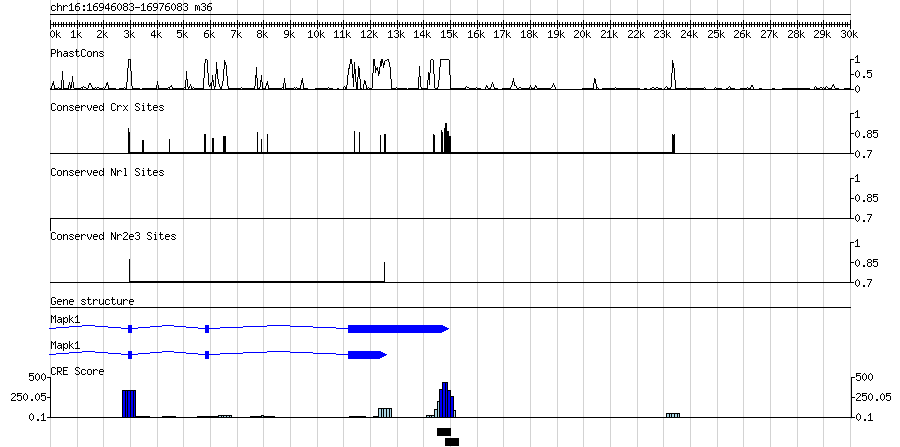

Supplement: Table S1 — Genes in the mouse photoreceptor transcription network. This table is a database which includes all genes dysregulated in Crx-/-, Nrl-/-, and/or Nr2e3-/- under high stringency criteria. Each row in the database represents a gene in the network and includes multiple links to additional types of information about that gene. ‘Large image gene +/− 15 Kb’ links to an image of our computational prediction of photoreceptor CREs in the genomic region 15 Kb upstream and downstream of the gene in question. ‘Closeup image TSS +/− 15 Kb’ links to an image of our computational prediction in the region 15 Kb on either side of the gene's TSS. Black bars within this image highlight the following regulatory peaks (if any are present): the peak closest to the TSS and the peak with the highest CRE score within this 30 Kb window. For those genes whose CRE predictions were tested experimentally, a red bar in this image indicates the location of the CRE that was tested. For Crx and Elovl4 the tested CRE is outside of this 30 Kb window and is therefore indicated in the ‘Large image’. ‘Links’ contains links to the UCSC genome browser, ENSEMBL, and NCBI database entries for the indicated gene, if available. ‘Max score (threshold of 200)’ contains the score of the highest predicted regulatory peak within the 30 Kb window around the gene's TSS. If this window does not contain any predicted peaks ≥ 200 (our cutoff threshold) no value is given (indicated by a dash). The next four columns of the table (‘Nr2e3-/-’ etc.) show the wild-type-to-mutant ratios of the averaged microarray scores for the given gene. For those genes to which more than one Affy tag correspond, the data for the first one listed under ‘Affy Mouse 430 2.0’ is given. Dark green = downregulated under high stringency (as described in METHODS); light green = downregulated under low stringency; red = upregulated under high stringency; orange = upregulated under low stringency. A dash indicates that the gene was not significantly a [file pone.0000643.s005.zip › Corbo_TableS1/images/1700056N10Rik_TSS.gif]

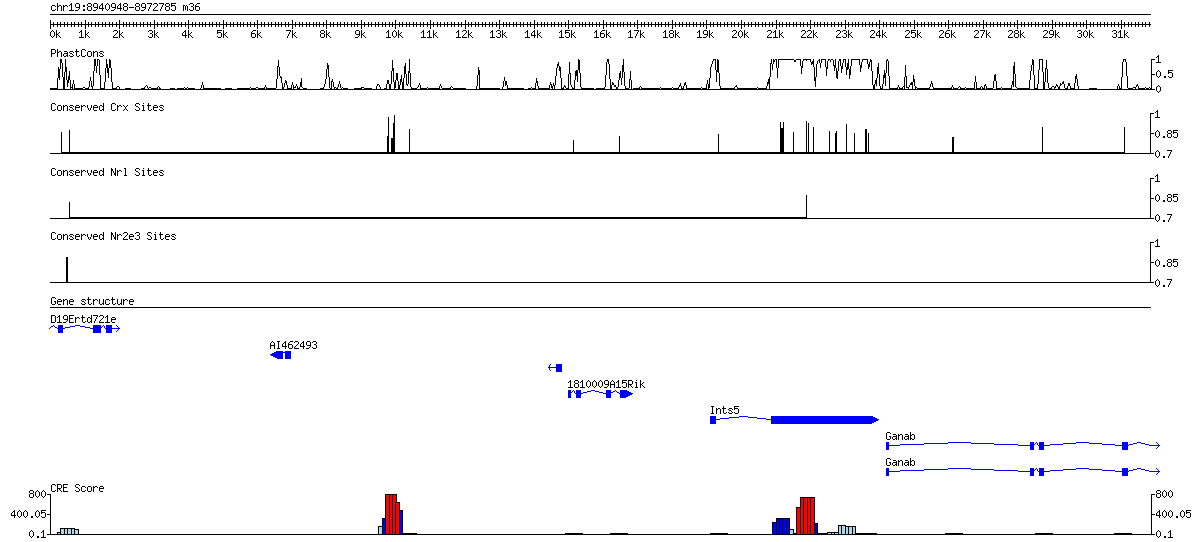

Supplement: Table S1 — Genes in the mouse photoreceptor transcription network. This table is a database which includes all genes dysregulated in Crx-/-, Nrl-/-, and/or Nr2e3-/- under high stringency criteria. Each row in the database represents a gene in the network and includes multiple links to additional types of information about that gene. ‘Large image gene +/− 15 Kb’ links to an image of our computational prediction of photoreceptor CREs in the genomic region 15 Kb upstream and downstream of the gene in question. ‘Closeup image TSS +/− 15 Kb’ links to an image of our computational prediction in the region 15 Kb on either side of the gene's TSS. Black bars within this image highlight the following regulatory peaks (if any are present): the peak closest to the TSS and the peak with the highest CRE score within this 30 Kb window. For those genes whose CRE predictions were tested experimentally, a red bar in this image indicates the location of the CRE that was tested. For Crx and Elovl4 the tested CRE is outside of this 30 Kb window and is therefore indicated in the ‘Large image’. ‘Links’ contains links to the UCSC genome browser, ENSEMBL, and NCBI database entries for the indicated gene, if available. ‘Max score (threshold of 200)’ contains the score of the highest predicted regulatory peak within the 30 Kb window around the gene's TSS. If this window does not contain any predicted peaks ≥ 200 (our cutoff threshold) no value is given (indicated by a dash). The next four columns of the table (‘Nr2e3-/-’ etc.) show the wild-type-to-mutant ratios of the averaged microarray scores for the given gene. For those genes to which more than one Affy tag correspond, the data for the first one listed under ‘Affy Mouse 430 2.0’ is given. Dark green = downregulated under high stringency (as described in METHODS); light green = downregulated under low stringency; red = upregulated under high stringency; orange = upregulated under low stringency. A dash indicates that the gene was not significantly a [file pone.0000643.s005.zip › Corbo_TableS1/images/1810009A15Rik.gif]

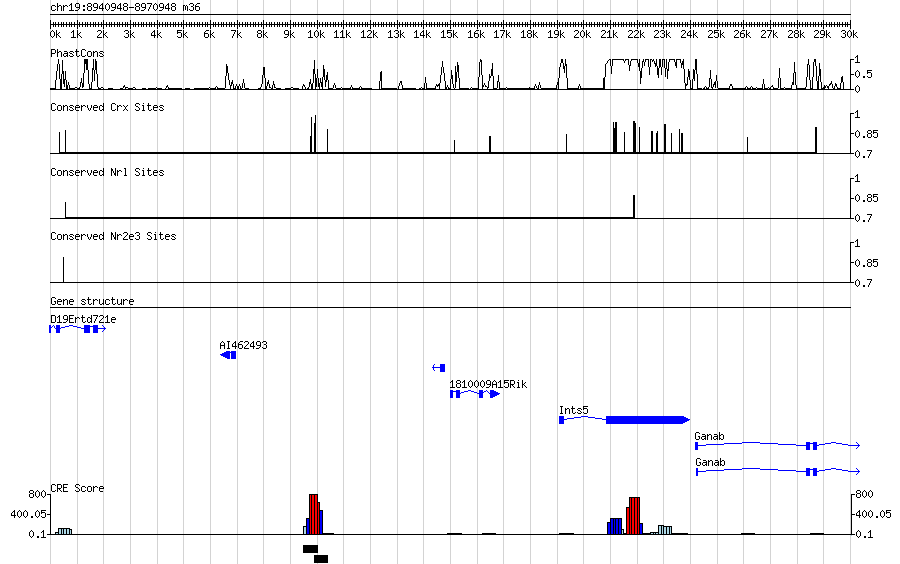

Supplement: Table S1 — Genes in the mouse photoreceptor transcription network. This table is a database which includes all genes dysregulated in Crx-/-, Nrl-/-, and/or Nr2e3-/- under high stringency criteria. Each row in the database represents a gene in the network and includes multiple links to additional types of information about that gene. ‘Large image gene +/− 15 Kb’ links to an image of our computational prediction of photoreceptor CREs in the genomic region 15 Kb upstream and downstream of the gene in question. ‘Closeup image TSS +/− 15 Kb’ links to an image of our computational prediction in the region 15 Kb on either side of the gene's TSS. Black bars within this image highlight the following regulatory peaks (if any are present): the peak closest to the TSS and the peak with the highest CRE score within this 30 Kb window. For those genes whose CRE predictions were tested experimentally, a red bar in this image indicates the location of the CRE that was tested. For Crx and Elovl4 the tested CRE is outside of this 30 Kb window and is therefore indicated in the ‘Large image’. ‘Links’ contains links to the UCSC genome browser, ENSEMBL, and NCBI database entries for the indicated gene, if available. ‘Max score (threshold of 200)’ contains the score of the highest predicted regulatory peak within the 30 Kb window around the gene's TSS. If this window does not contain any predicted peaks ≥ 200 (our cutoff threshold) no value is given (indicated by a dash). The next four columns of the table (‘Nr2e3-/-’ etc.) show the wild-type-to-mutant ratios of the averaged microarray scores for the given gene. For those genes to which more than one Affy tag correspond, the data for the first one listed under ‘Affy Mouse 430 2.0’ is given. Dark green = downregulated under high stringency (as described in METHODS); light green = downregulated under low stringency; red = upregulated under high stringency; orange = upregulated under low stringency. A dash indicates that the gene was not significantly a [file pone.0000643.s005.zip › Corbo_TableS1/images/1810009A15Rik_TSS.gif]

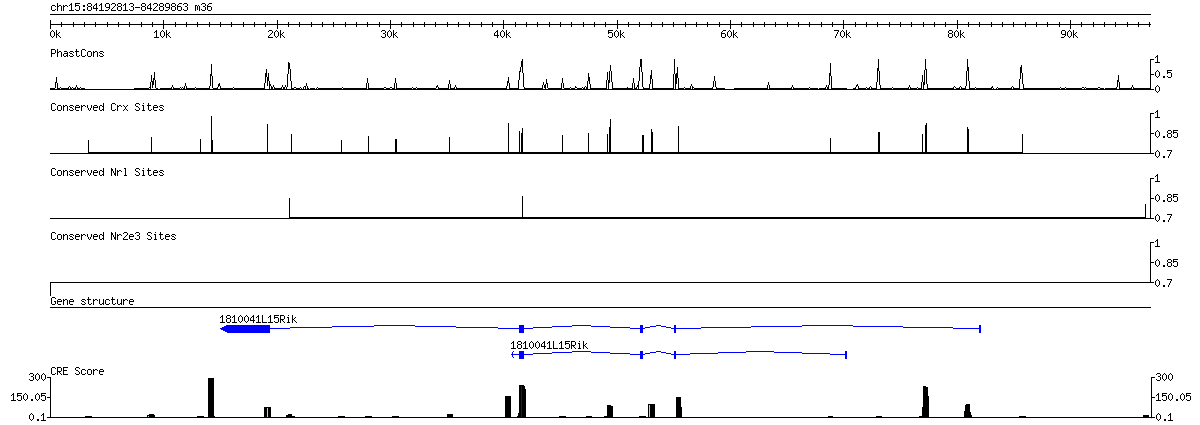

Supplement: Table S1 — Genes in the mouse photoreceptor transcription network. This table is a database which includes all genes dysregulated in Crx-/-, Nrl-/-, and/or Nr2e3-/- under high stringency criteria. Each row in the database represents a gene in the network and includes multiple links to additional types of information about that gene. ‘Large image gene +/− 15 Kb’ links to an image of our computational prediction of photoreceptor CREs in the genomic region 15 Kb upstream and downstream of the gene in question. ‘Closeup image TSS +/− 15 Kb’ links to an image of our computational prediction in the region 15 Kb on either side of the gene's TSS. Black bars within this image highlight the following regulatory peaks (if any are present): the peak closest to the TSS and the peak with the highest CRE score within this 30 Kb window. For those genes whose CRE predictions were tested experimentally, a red bar in this image indicates the location of the CRE that was tested. For Crx and Elovl4 the tested CRE is outside of this 30 Kb window and is therefore indicated in the ‘Large image’. ‘Links’ contains links to the UCSC genome browser, ENSEMBL, and NCBI database entries for the indicated gene, if available. ‘Max score (threshold of 200)’ contains the score of the highest predicted regulatory peak within the 30 Kb window around the gene's TSS. If this window does not contain any predicted peaks ≥ 200 (our cutoff threshold) no value is given (indicated by a dash). The next four columns of the table (‘Nr2e3-/-’ etc.) show the wild-type-to-mutant ratios of the averaged microarray scores for the given gene. For those genes to which more than one Affy tag correspond, the data for the first one listed under ‘Affy Mouse 430 2.0’ is given. Dark green = downregulated under high stringency (as described in METHODS); light green = downregulated under low stringency; red = upregulated under high stringency; orange = upregulated under low stringency. A dash indicates that the gene was not significantly a [file pone.0000643.s005.zip › Corbo_TableS1/images/1810041L15Rik.gif]

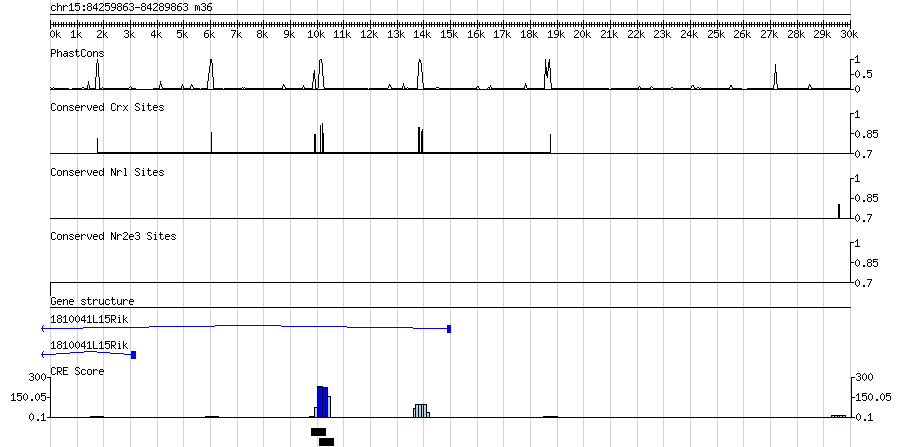

Supplement: Table S1 — Genes in the mouse photoreceptor transcription network. This table is a database which includes all genes dysregulated in Crx-/-, Nrl-/-, and/or Nr2e3-/- under high stringency criteria. Each row in the database represents a gene in the network and includes multiple links to additional types of information about that gene. ‘Large image gene +/− 15 Kb’ links to an image of our computational prediction of photoreceptor CREs in the genomic region 15 Kb upstream and downstream of the gene in question. ‘Closeup image TSS +/− 15 Kb’ links to an image of our computational prediction in the region 15 Kb on either side of the gene's TSS. Black bars within this image highlight the following regulatory peaks (if any are present): the peak closest to the TSS and the peak with the highest CRE score within this 30 Kb window. For those genes whose CRE predictions were tested experimentally, a red bar in this image indicates the location of the CRE that was tested. For Crx and Elovl4 the tested CRE is outside of this 30 Kb window and is therefore indicated in the ‘Large image’. ‘Links’ contains links to the UCSC genome browser, ENSEMBL, and NCBI database entries for the indicated gene, if available. ‘Max score (threshold of 200)’ contains the score of the highest predicted regulatory peak within the 30 Kb window around the gene's TSS. If this window does not contain any predicted peaks ≥ 200 (our cutoff threshold) no value is given (indicated by a dash). The next four columns of the table (‘Nr2e3-/-’ etc.) show the wild-type-to-mutant ratios of the averaged microarray scores for the given gene. For those genes to which more than one Affy tag correspond, the data for the first one listed under ‘Affy Mouse 430 2.0’ is given. Dark green = downregulated under high stringency (as described in METHODS); light green = downregulated under low stringency; red = upregulated under high stringency; orange = upregulated under low stringency. A dash indicates that the gene was not significantly a [file pone.0000643.s005.zip › Corbo_TableS1/images/1810041L15Rik_TSS.gif]

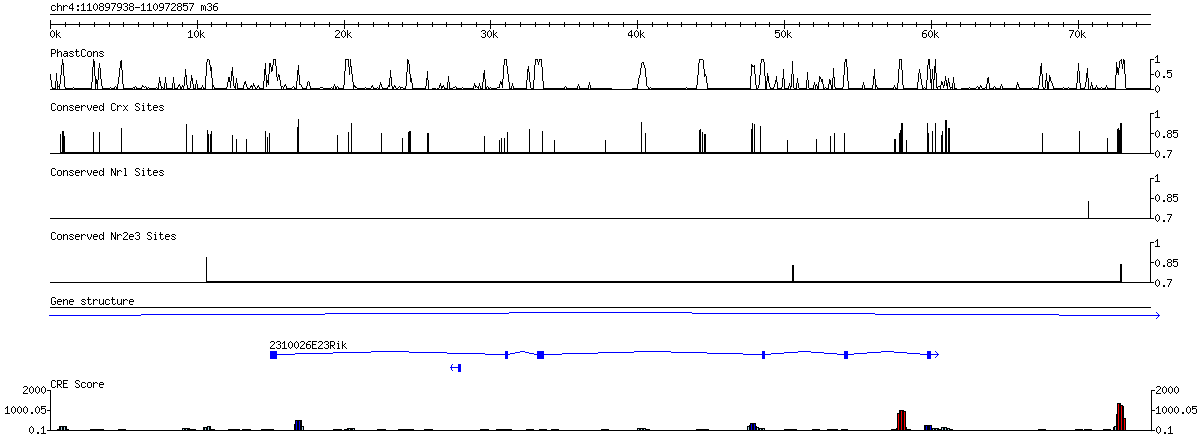

Supplement: Table S1 — Genes in the mouse photoreceptor transcription network. This table is a database which includes all genes dysregulated in Crx-/-, Nrl-/-, and/or Nr2e3-/- under high stringency criteria. Each row in the database represents a gene in the network and includes multiple links to additional types of information about that gene. ‘Large image gene +/− 15 Kb’ links to an image of our computational prediction of photoreceptor CREs in the genomic region 15 Kb upstream and downstream of the gene in question. ‘Closeup image TSS +/− 15 Kb’ links to an image of our computational prediction in the region 15 Kb on either side of the gene's TSS. Black bars within this image highlight the following regulatory peaks (if any are present): the peak closest to the TSS and the peak with the highest CRE score within this 30 Kb window. For those genes whose CRE predictions were tested experimentally, a red bar in this image indicates the location of the CRE that was tested. For Crx and Elovl4 the tested CRE is outside of this 30 Kb window and is therefore indicated in the ‘Large image’. ‘Links’ contains links to the UCSC genome browser, ENSEMBL, and NCBI database entries for the indicated gene, if available. ‘Max score (threshold of 200)’ contains the score of the highest predicted regulatory peak within the 30 Kb window around the gene's TSS. If this window does not contain any predicted peaks ≥ 200 (our cutoff threshold) no value is given (indicated by a dash). The next four columns of the table (‘Nr2e3-/-’ etc.) show the wild-type-to-mutant ratios of the averaged microarray scores for the given gene. For those genes to which more than one Affy tag correspond, the data for the first one listed under ‘Affy Mouse 430 2.0’ is given. Dark green = downregulated under high stringency (as described in METHODS); light green = downregulated under low stringency; red = upregulated under high stringency; orange = upregulated under low stringency. A dash indicates that the gene was not significantly a [file pone.0000643.s005.zip › Corbo_TableS1/images/2310026E23Rik.gif]

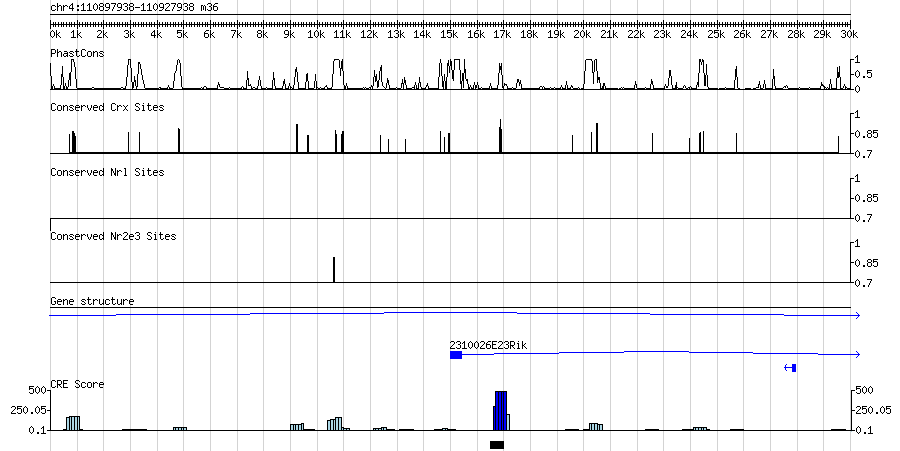

Supplement: Table S1 — Genes in the mouse photoreceptor transcription network. This table is a database which includes all genes dysregulated in Crx-/-, Nrl-/-, and/or Nr2e3-/- under high stringency criteria. Each row in the database represents a gene in the network and includes multiple links to additional types of information about that gene. ‘Large image gene +/− 15 Kb’ links to an image of our computational prediction of photoreceptor CREs in the genomic region 15 Kb upstream and downstream of the gene in question. ‘Closeup image TSS +/− 15 Kb’ links to an image of our computational prediction in the region 15 Kb on either side of the gene's TSS. Black bars within this image highlight the following regulatory peaks (if any are present): the peak closest to the TSS and the peak with the highest CRE score within this 30 Kb window. For those genes whose CRE predictions were tested experimentally, a red bar in this image indicates the location of the CRE that was tested. For Crx and Elovl4 the tested CRE is outside of this 30 Kb window and is therefore indicated in the ‘Large image’. ‘Links’ contains links to the UCSC genome browser, ENSEMBL, and NCBI database entries for the indicated gene, if available. ‘Max score (threshold of 200)’ contains the score of the highest predicted regulatory peak within the 30 Kb window around the gene's TSS. If this window does not contain any predicted peaks ≥ 200 (our cutoff threshold) no value is given (indicated by a dash). The next four columns of the table (‘Nr2e3-/-’ etc.) show the wild-type-to-mutant ratios of the averaged microarray scores for the given gene. For those genes to which more than one Affy tag correspond, the data for the first one listed under ‘Affy Mouse 430 2.0’ is given. Dark green = downregulated under high stringency (as described in METHODS); light green = downregulated under low stringency; red = upregulated under high stringency; orange = upregulated under low stringency. A dash indicates that the gene was not significantly a [file pone.0000643.s005.zip › Corbo_TableS1/images/2310026E23Rik_TSS.gif]

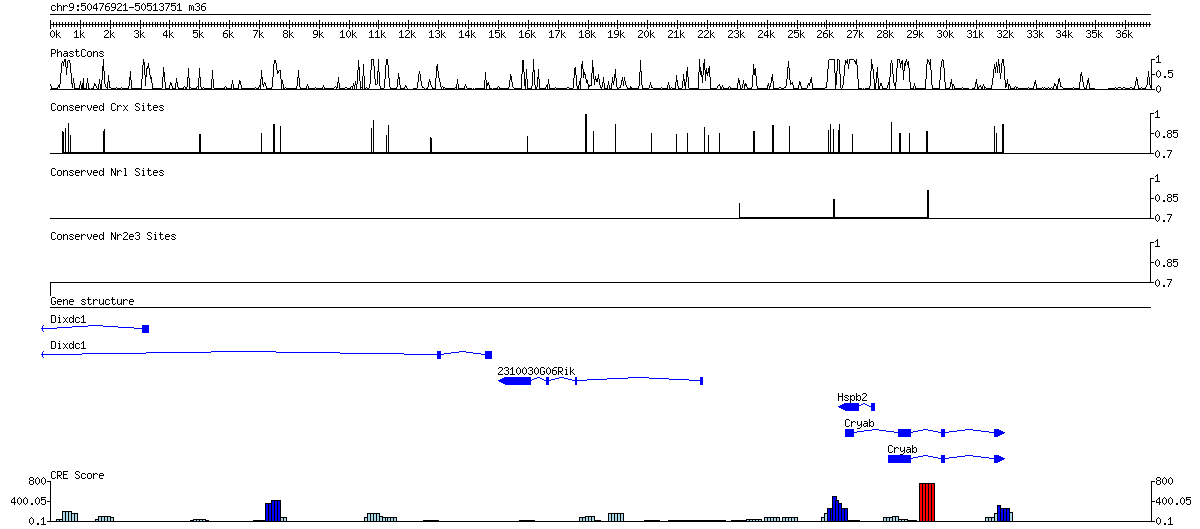

Supplement: Table S1 — Genes in the mouse photoreceptor transcription network. This table is a database which includes all genes dysregulated in Crx-/-, Nrl-/-, and/or Nr2e3-/- under high stringency criteria. Each row in the database represents a gene in the network and includes multiple links to additional types of information about that gene. ‘Large image gene +/− 15 Kb’ links to an image of our computational prediction of photoreceptor CREs in the genomic region 15 Kb upstream and downstream of the gene in question. ‘Closeup image TSS +/− 15 Kb’ links to an image of our computational prediction in the region 15 Kb on either side of the gene's TSS. Black bars within this image highlight the following regulatory peaks (if any are present): the peak closest to the TSS and the peak with the highest CRE score within this 30 Kb window. For those genes whose CRE predictions were tested experimentally, a red bar in this image indicates the location of the CRE that was tested. For Crx and Elovl4 the tested CRE is outside of this 30 Kb window and is therefore indicated in the ‘Large image’. ‘Links’ contains links to the UCSC genome browser, ENSEMBL, and NCBI database entries for the indicated gene, if available. ‘Max score (threshold of 200)’ contains the score of the highest predicted regulatory peak within the 30 Kb window around the gene's TSS. If this window does not contain any predicted peaks ≥ 200 (our cutoff threshold) no value is given (indicated by a dash). The next four columns of the table (‘Nr2e3-/-’ etc.) show the wild-type-to-mutant ratios of the averaged microarray scores for the given gene. For those genes to which more than one Affy tag correspond, the data for the first one listed under ‘Affy Mouse 430 2.0’ is given. Dark green = downregulated under high stringency (as described in METHODS); light green = downregulated under low stringency; red = upregulated under high stringency; orange = upregulated under low stringency. A dash indicates that the gene was not significantly a [file pone.0000643.s005.zip › Corbo_TableS1/images/2310030G06Rik.gif]

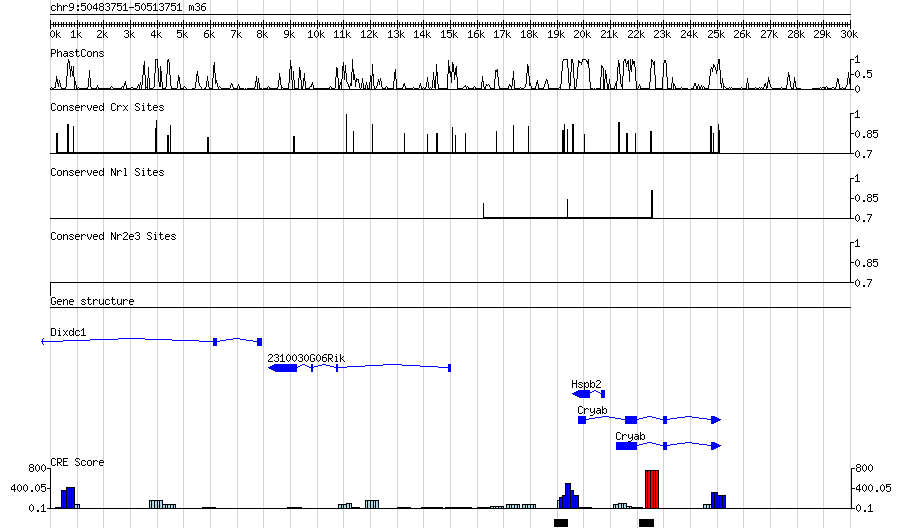

Supplement: Table S1 — Genes in the mouse photoreceptor transcription network. This table is a database which includes all genes dysregulated in Crx-/-, Nrl-/-, and/or Nr2e3-/- under high stringency criteria. Each row in the database represents a gene in the network and includes multiple links to additional types of information about that gene. ‘Large image gene +/− 15 Kb’ links to an image of our computational prediction of photoreceptor CREs in the genomic region 15 Kb upstream and downstream of the gene in question. ‘Closeup image TSS +/− 15 Kb’ links to an image of our computational prediction in the region 15 Kb on either side of the gene's TSS. Black bars within this image highlight the following regulatory peaks (if any are present): the peak closest to the TSS and the peak with the highest CRE score within this 30 Kb window. For those genes whose CRE predictions were tested experimentally, a red bar in this image indicates the location of the CRE that was tested. For Crx and Elovl4 the tested CRE is outside of this 30 Kb window and is therefore indicated in the ‘Large image’. ‘Links’ contains links to the UCSC genome browser, ENSEMBL, and NCBI database entries for the indicated gene, if available. ‘Max score (threshold of 200)’ contains the score of the highest predicted regulatory peak within the 30 Kb window around the gene's TSS. If this window does not contain any predicted peaks ≥ 200 (our cutoff threshold) no value is given (indicated by a dash). The next four columns of the table (‘Nr2e3-/-’ etc.) show the wild-type-to-mutant ratios of the averaged microarray scores for the given gene. For those genes to which more than one Affy tag correspond, the data for the first one listed under ‘Affy Mouse 430 2.0’ is given. Dark green = downregulated under high stringency (as described in METHODS); light green = downregulated under low stringency; red = upregulated under high stringency; orange = upregulated under low stringency. A dash indicates that the gene was not significantly a [file pone.0000643.s005.zip › Corbo_TableS1/images/2310030G06Rik_TSS.gif]

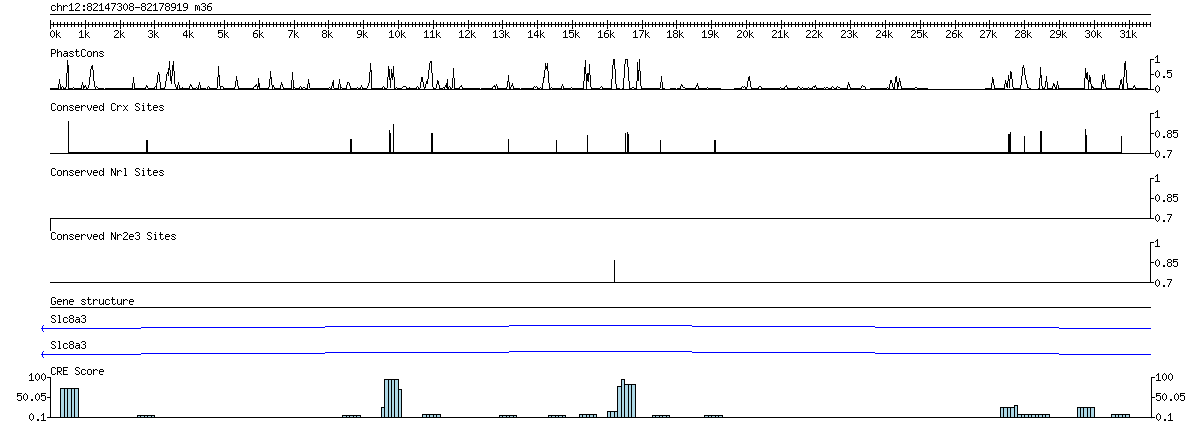

Supplement: Table S1 — Genes in the mouse photoreceptor transcription network. This table is a database which includes all genes dysregulated in Crx-/-, Nrl-/-, and/or Nr2e3-/- under high stringency criteria. Each row in the database represents a gene in the network and includes multiple links to additional types of information about that gene. ‘Large image gene +/− 15 Kb’ links to an image of our computational prediction of photoreceptor CREs in the genomic region 15 Kb upstream and downstream of the gene in question. ‘Closeup image TSS +/− 15 Kb’ links to an image of our computational prediction in the region 15 Kb on either side of the gene's TSS. Black bars within this image highlight the following regulatory peaks (if any are present): the peak closest to the TSS and the peak with the highest CRE score within this 30 Kb window. For those genes whose CRE predictions were tested experimentally, a red bar in this image indicates the location of the CRE that was tested. For Crx and Elovl4 the tested CRE is outside of this 30 Kb window and is therefore indicated in the ‘Large image’. ‘Links’ contains links to the UCSC genome browser, ENSEMBL, and NCBI database entries for the indicated gene, if available. ‘Max score (threshold of 200)’ contains the score of the highest predicted regulatory peak within the 30 Kb window around the gene's TSS. If this window does not contain any predicted peaks ≥ 200 (our cutoff threshold) no value is given (indicated by a dash). The next four columns of the table (‘Nr2e3-/-’ etc.) show the wild-type-to-mutant ratios of the averaged microarray scores for the given gene. For those genes to which more than one Affy tag correspond, the data for the first one listed under ‘Affy Mouse 430 2.0’ is given. Dark green = downregulated under high stringency (as described in METHODS); light green = downregulated under low stringency; red = upregulated under high stringency; orange = upregulated under low stringency. A dash indicates that the gene was not significantly a [file pone.0000643.s005.zip › Corbo_TableS1/images/2310068G24Rik.gif]

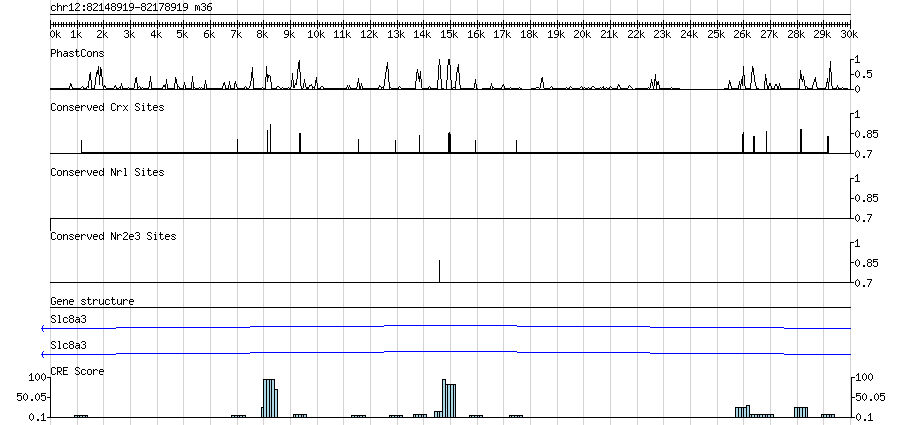

Supplement: Table S1 — Genes in the mouse photoreceptor transcription network. This table is a database which includes all genes dysregulated in Crx-/-, Nrl-/-, and/or Nr2e3-/- under high stringency criteria. Each row in the database represents a gene in the network and includes multiple links to additional types of information about that gene. ‘Large image gene +/− 15 Kb’ links to an image of our computational prediction of photoreceptor CREs in the genomic region 15 Kb upstream and downstream of the gene in question. ‘Closeup image TSS +/− 15 Kb’ links to an image of our computational prediction in the region 15 Kb on either side of the gene's TSS. Black bars within this image highlight the following regulatory peaks (if any are present): the peak closest to the TSS and the peak with the highest CRE score within this 30 Kb window. For those genes whose CRE predictions were tested experimentally, a red bar in this image indicates the location of the CRE that was tested. For Crx and Elovl4 the tested CRE is outside of this 30 Kb window and is therefore indicated in the ‘Large image’. ‘Links’ contains links to the UCSC genome browser, ENSEMBL, and NCBI database entries for the indicated gene, if available. ‘Max score (threshold of 200)’ contains the score of the highest predicted regulatory peak within the 30 Kb window around the gene's TSS. If this window does not contain any predicted peaks ≥ 200 (our cutoff threshold) no value is given (indicated by a dash). The next four columns of the table (‘Nr2e3-/-’ etc.) show the wild-type-to-mutant ratios of the averaged microarray scores for the given gene. For those genes to which more than one Affy tag correspond, the data for the first one listed under ‘Affy Mouse 430 2.0’ is given. Dark green = downregulated under high stringency (as described in METHODS); light green = downregulated under low stringency; red = upregulated under high stringency; orange = upregulated under low stringency. A dash indicates that the gene was not significantly a [file pone.0000643.s005.zip › Corbo_TableS1/images/2310068G24Rik_TSS.gif]

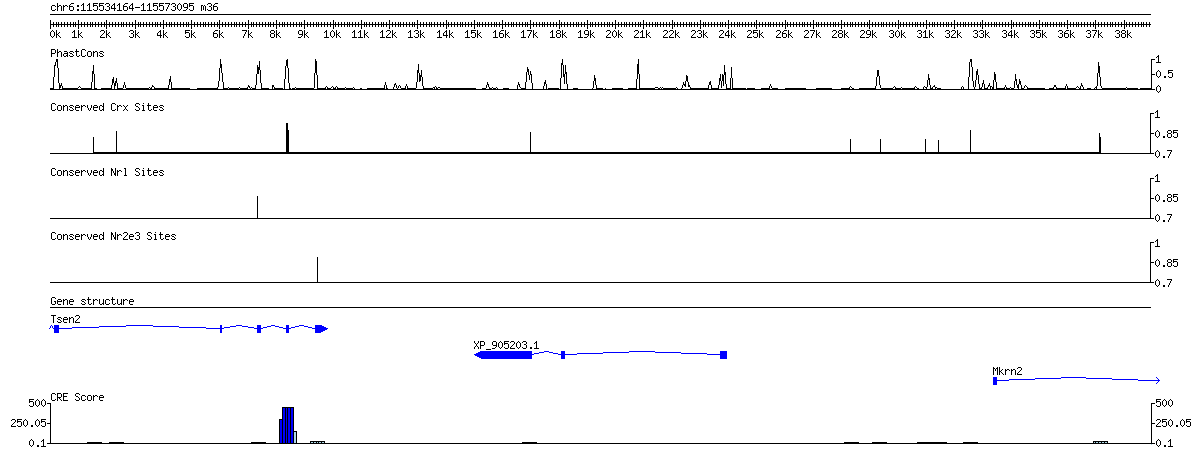

Supplement: Table S1 — Genes in the mouse photoreceptor transcription network. This table is a database which includes all genes dysregulated in Crx-/-, Nrl-/-, and/or Nr2e3-/- under high stringency criteria. Each row in the database represents a gene in the network and includes multiple links to additional types of information about that gene. ‘Large image gene +/− 15 Kb’ links to an image of our computational prediction of photoreceptor CREs in the genomic region 15 Kb upstream and downstream of the gene in question. ‘Closeup image TSS +/− 15 Kb’ links to an image of our computational prediction in the region 15 Kb on either side of the gene's TSS. Black bars within this image highlight the following regulatory peaks (if any are present): the peak closest to the TSS and the peak with the highest CRE score within this 30 Kb window. For those genes whose CRE predictions were tested experimentally, a red bar in this image indicates the location of the CRE that was tested. For Crx and Elovl4 the tested CRE is outside of this 30 Kb window and is therefore indicated in the ‘Large image’. ‘Links’ contains links to the UCSC genome browser, ENSEMBL, and NCBI database entries for the indicated gene, if available. ‘Max score (threshold of 200)’ contains the score of the highest predicted regulatory peak within the 30 Kb window around the gene's TSS. If this window does not contain any predicted peaks ≥ 200 (our cutoff threshold) no value is given (indicated by a dash). The next four columns of the table (‘Nr2e3-/-’ etc.) show the wild-type-to-mutant ratios of the averaged microarray scores for the given gene. For those genes to which more than one Affy tag correspond, the data for the first one listed under ‘Affy Mouse 430 2.0’ is given. Dark green = downregulated under high stringency (as described in METHODS); light green = downregulated under low stringency; red = upregulated under high stringency; orange = upregulated under low stringency. A dash indicates that the gene was not significantly a [file pone.0000643.s005.zip › Corbo_TableS1/images/2510049J12Rik.gif]

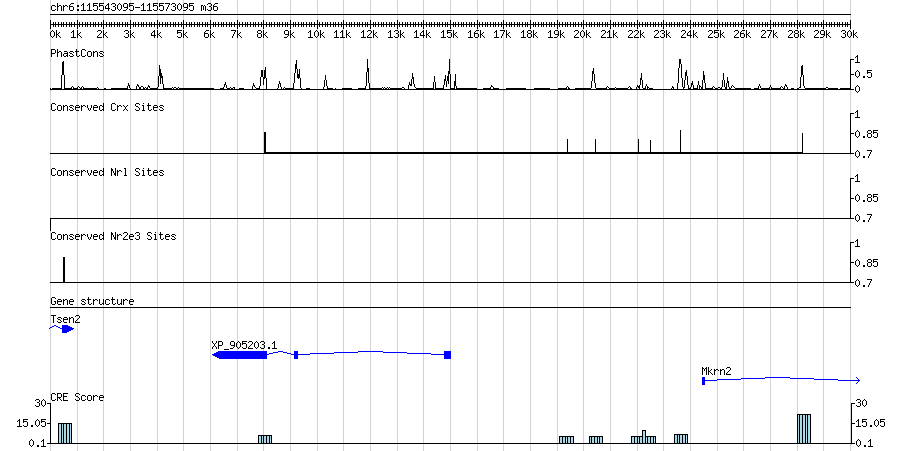

Supplement: Table S1 — Genes in the mouse photoreceptor transcription network. This table is a database which includes all genes dysregulated in Crx-/-, Nrl-/-, and/or Nr2e3-/- under high stringency criteria. Each row in the database represents a gene in the network and includes multiple links to additional types of information about that gene. ‘Large image gene +/− 15 Kb’ links to an image of our computational prediction of photoreceptor CREs in the genomic region 15 Kb upstream and downstream of the gene in question. ‘Closeup image TSS +/− 15 Kb’ links to an image of our computational prediction in the region 15 Kb on either side of the gene's TSS. Black bars within this image highlight the following regulatory peaks (if any are present): the peak closest to the TSS and the peak with the highest CRE score within this 30 Kb window. For those genes whose CRE predictions were tested experimentally, a red bar in this image indicates the location of the CRE that was tested. For Crx and Elovl4 the tested CRE is outside of this 30 Kb window and is therefore indicated in the ‘Large image’. ‘Links’ contains links to the UCSC genome browser, ENSEMBL, and NCBI database entries for the indicated gene, if available. ‘Max score (threshold of 200)’ contains the score of the highest predicted regulatory peak within the 30 Kb window around the gene's TSS. If this window does not contain any predicted peaks ≥ 200 (our cutoff threshold) no value is given (indicated by a dash). The next four columns of the table (‘Nr2e3-/-’ etc.) show the wild-type-to-mutant ratios of the averaged microarray scores for the given gene. For those genes to which more than one Affy tag correspond, the data for the first one listed under ‘Affy Mouse 430 2.0’ is given. Dark green = downregulated under high stringency (as described in METHODS); light green = downregulated under low stringency; red = upregulated under high stringency; orange = upregulated under low stringency. A dash indicates that the gene was not significantly a [file pone.0000643.s005.zip › Corbo_TableS1/images/2510049J12Rik_TSS.gif]

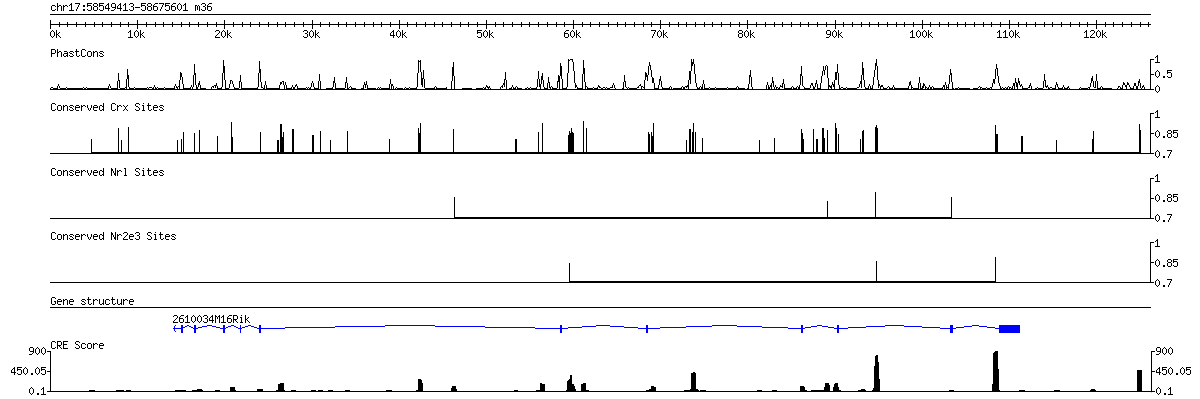

Supplement: Table S1 — Genes in the mouse photoreceptor transcription network. This table is a database which includes all genes dysregulated in Crx-/-, Nrl-/-, and/or Nr2e3-/- under high stringency criteria. Each row in the database represents a gene in the network and includes multiple links to additional types of information about that gene. ‘Large image gene +/− 15 Kb’ links to an image of our computational prediction of photoreceptor CREs in the genomic region 15 Kb upstream and downstream of the gene in question. ‘Closeup image TSS +/− 15 Kb’ links to an image of our computational prediction in the region 15 Kb on either side of the gene's TSS. Black bars within this image highlight the following regulatory peaks (if any are present): the peak closest to the TSS and the peak with the highest CRE score within this 30 Kb window. For those genes whose CRE predictions were tested experimentally, a red bar in this image indicates the location of the CRE that was tested. For Crx and Elovl4 the tested CRE is outside of this 30 Kb window and is therefore indicated in the ‘Large image’. ‘Links’ contains links to the UCSC genome browser, ENSEMBL, and NCBI database entries for the indicated gene, if available. ‘Max score (threshold of 200)’ contains the score of the highest predicted regulatory peak within the 30 Kb window around the gene's TSS. If this window does not contain any predicted peaks ≥ 200 (our cutoff threshold) no value is given (indicated by a dash). The next four columns of the table (‘Nr2e3-/-’ etc.) show the wild-type-to-mutant ratios of the averaged microarray scores for the given gene. For those genes to which more than one Affy tag correspond, the data for the first one listed under ‘Affy Mouse 430 2.0’ is given. Dark green = downregulated under high stringency (as described in METHODS); light green = downregulated under low stringency; red = upregulated under high stringency; orange = upregulated under low stringency. A dash indicates that the gene was not significantly a [file pone.0000643.s005.zip › Corbo_TableS1/images/2610034M16Rik.gif]

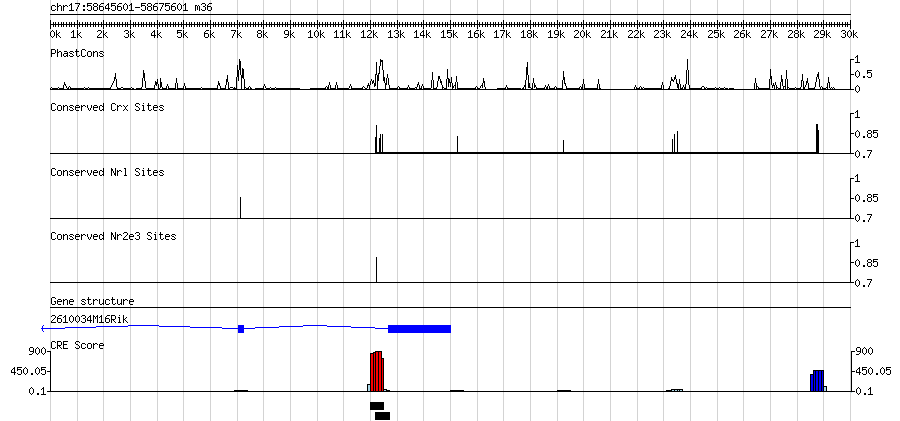

Supplement: Table S1 — Genes in the mouse photoreceptor transcription network. This table is a database which includes all genes dysregulated in Crx-/-, Nrl-/-, and/or Nr2e3-/- under high stringency criteria. Each row in the database represents a gene in the network and includes multiple links to additional types of information about that gene. ‘Large image gene +/− 15 Kb’ links to an image of our computational prediction of photoreceptor CREs in the genomic region 15 Kb upstream and downstream of the gene in question. ‘Closeup image TSS +/− 15 Kb’ links to an image of our computational prediction in the region 15 Kb on either side of the gene's TSS. Black bars within this image highlight the following regulatory peaks (if any are present): the peak closest to the TSS and the peak with the highest CRE score within this 30 Kb window. For those genes whose CRE predictions were tested experimentally, a red bar in this image indicates the location of the CRE that was tested. For Crx and Elovl4 the tested CRE is outside of this 30 Kb window and is therefore indicated in the ‘Large image’. ‘Links’ contains links to the UCSC genome browser, ENSEMBL, and NCBI database entries for the indicated gene, if available. ‘Max score (threshold of 200)’ contains the score of the highest predicted regulatory peak within the 30 Kb window around the gene's TSS. If this window does not contain any predicted peaks ≥ 200 (our cutoff threshold) no value is given (indicated by a dash). The next four columns of the table (‘Nr2e3-/-’ etc.) show the wild-type-to-mutant ratios of the averaged microarray scores for the given gene. For those genes to which more than one Affy tag correspond, the data for the first one listed under ‘Affy Mouse 430 2.0’ is given. Dark green = downregulated under high stringency (as described in METHODS); light green = downregulated under low stringency; red = upregulated under high stringency; orange = upregulated under low stringency. A dash indicates that the gene was not significantly a [file pone.0000643.s005.zip › Corbo_TableS1/images/2610034M16Rik_TSS.gif]

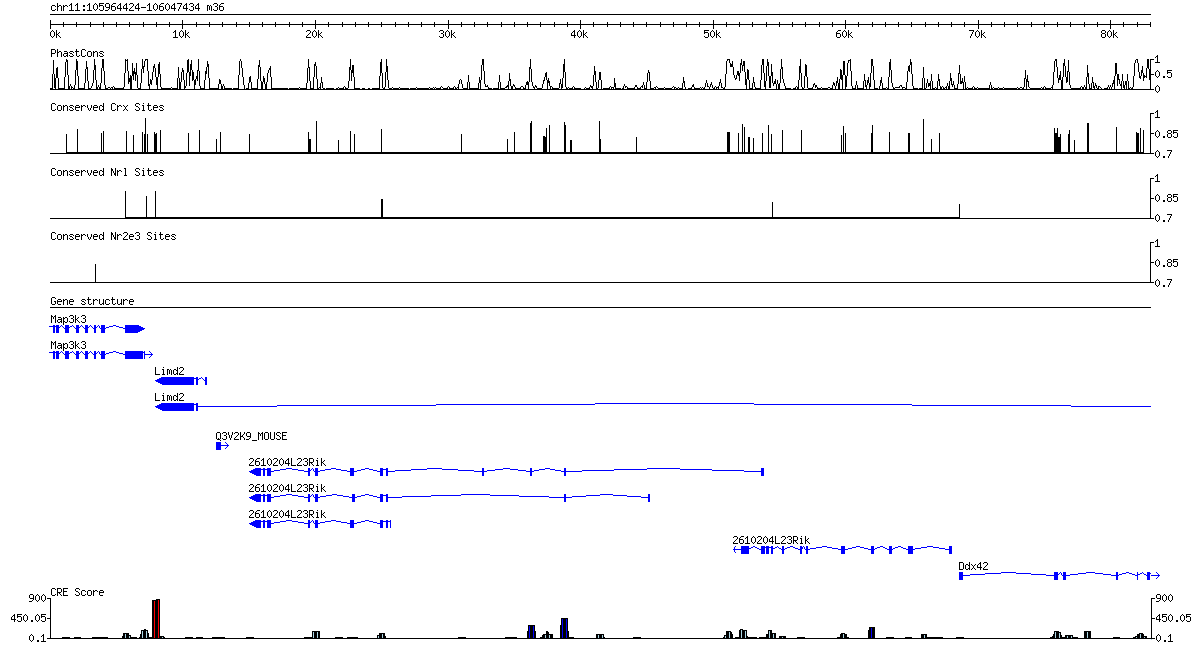

Supplement: Table S1 — Genes in the mouse photoreceptor transcription network. This table is a database which includes all genes dysregulated in Crx-/-, Nrl-/-, and/or Nr2e3-/- under high stringency criteria. Each row in the database represents a gene in the network and includes multiple links to additional types of information about that gene. ‘Large image gene +/− 15 Kb’ links to an image of our computational prediction of photoreceptor CREs in the genomic region 15 Kb upstream and downstream of the gene in question. ‘Closeup image TSS +/− 15 Kb’ links to an image of our computational prediction in the region 15 Kb on either side of the gene's TSS. Black bars within this image highlight the following regulatory peaks (if any are present): the peak closest to the TSS and the peak with the highest CRE score within this 30 Kb window. For those genes whose CRE predictions were tested experimentally, a red bar in this image indicates the location of the CRE that was tested. For Crx and Elovl4 the tested CRE is outside of this 30 Kb window and is therefore indicated in the ‘Large image’. ‘Links’ contains links to the UCSC genome browser, ENSEMBL, and NCBI database entries for the indicated gene, if available. ‘Max score (threshold of 200)’ contains the score of the highest predicted regulatory peak within the 30 Kb window around the gene's TSS. If this window does not contain any predicted peaks ≥ 200 (our cutoff threshold) no value is given (indicated by a dash). The next four columns of the table (‘Nr2e3-/-’ etc.) show the wild-type-to-mutant ratios of the averaged microarray scores for the given gene. For those genes to which more than one Affy tag correspond, the data for the first one listed under ‘Affy Mouse 430 2.0’ is given. Dark green = downregulated under high stringency (as described in METHODS); light green = downregulated under low stringency; red = upregulated under high stringency; orange = upregulated under low stringency. A dash indicates that the gene was not significantly a [file pone.0000643.s005.zip › Corbo_TableS1/images/2610204L23Rik.gif]

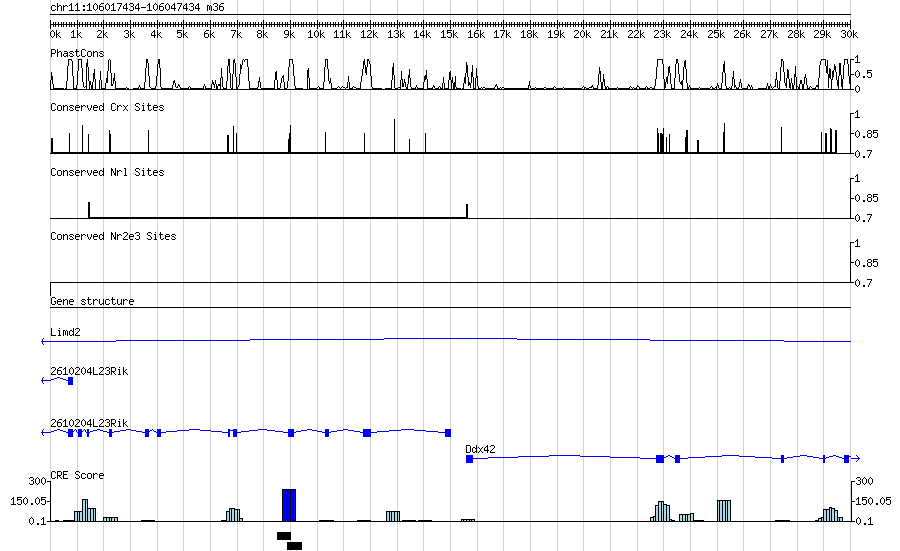

Supplement: Table S1 — Genes in the mouse photoreceptor transcription network. This table is a database which includes all genes dysregulated in Crx-/-, Nrl-/-, and/or Nr2e3-/- under high stringency criteria. Each row in the database represents a gene in the network and includes multiple links to additional types of information about that gene. ‘Large image gene +/− 15 Kb’ links to an image of our computational prediction of photoreceptor CREs in the genomic region 15 Kb upstream and downstream of the gene in question. ‘Closeup image TSS +/− 15 Kb’ links to an image of our computational prediction in the region 15 Kb on either side of the gene's TSS. Black bars within this image highlight the following regulatory peaks (if any are present): the peak closest to the TSS and the peak with the highest CRE score within this 30 Kb window. For those genes whose CRE predictions were tested experimentally, a red bar in this image indicates the location of the CRE that was tested. For Crx and Elovl4 the tested CRE is outside of this 30 Kb window and is therefore indicated in the ‘Large image’. ‘Links’ contains links to the UCSC genome browser, ENSEMBL, and NCBI database entries for the indicated gene, if available. ‘Max score (threshold of 200)’ contains the score of the highest predicted regulatory peak within the 30 Kb window around the gene's TSS. If this window does not contain any predicted peaks ≥ 200 (our cutoff threshold) no value is given (indicated by a dash). The next four columns of the table (‘Nr2e3-/-’ etc.) show the wild-type-to-mutant ratios of the averaged microarray scores for the given gene. For those genes to which more than one Affy tag correspond, the data for the first one listed under ‘Affy Mouse 430 2.0’ is given. Dark green = downregulated under high stringency (as described in METHODS); light green = downregulated under low stringency; red = upregulated under high stringency; orange = upregulated under low stringency. A dash indicates that the gene was not significantly a [file pone.0000643.s005.zip › Corbo_TableS1/images/2610204L23Rik_TSS.gif]

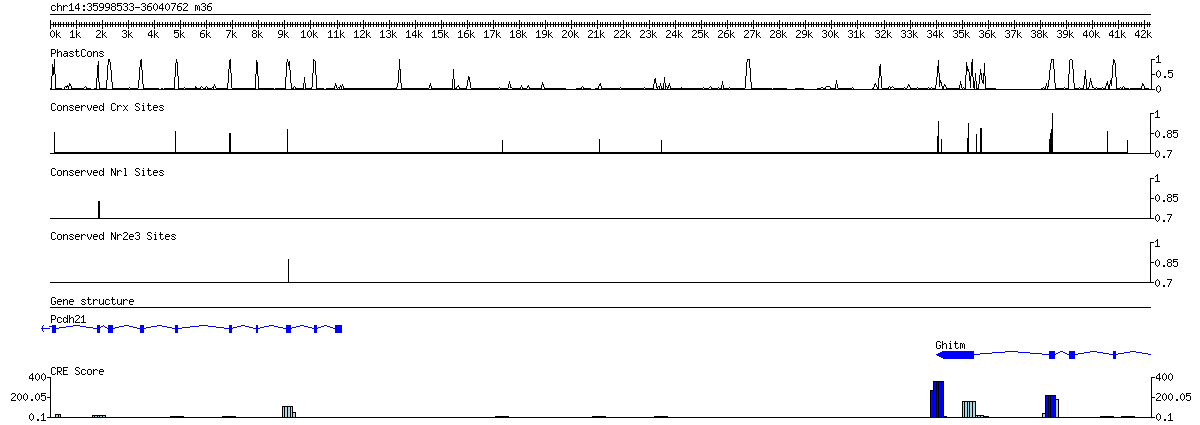

Supplement: Table S1 — Genes in the mouse photoreceptor transcription network. This table is a database which includes all genes dysregulated in Crx-/-, Nrl-/-, and/or Nr2e3-/- under high stringency criteria. Each row in the database represents a gene in the network and includes multiple links to additional types of information about that gene. ‘Large image gene +/− 15 Kb’ links to an image of our computational prediction of photoreceptor CREs in the genomic region 15 Kb upstream and downstream of the gene in question. ‘Closeup image TSS +/− 15 Kb’ links to an image of our computational prediction in the region 15 Kb on either side of the gene's TSS. Black bars within this image highlight the following regulatory peaks (if any are present): the peak closest to the TSS and the peak with the highest CRE score within this 30 Kb window. For those genes whose CRE predictions were tested experimentally, a red bar in this image indicates the location of the CRE that was tested. For Crx and Elovl4 the tested CRE is outside of this 30 Kb window and is therefore indicated in the ‘Large image’. ‘Links’ contains links to the UCSC genome browser, ENSEMBL, and NCBI database entries for the indicated gene, if available. ‘Max score (threshold of 200)’ contains the score of the highest predicted regulatory peak within the 30 Kb window around the gene's TSS. If this window does not contain any predicted peaks ≥ 200 (our cutoff threshold) no value is given (indicated by a dash). The next four columns of the table (‘Nr2e3-/-’ etc.) show the wild-type-to-mutant ratios of the averaged microarray scores for the given gene. For those genes to which more than one Affy tag correspond, the data for the first one listed under ‘Affy Mouse 430 2.0’ is given. Dark green = downregulated under high stringency (as described in METHODS); light green = downregulated under low stringency; red = upregulated under high stringency; orange = upregulated under low stringency. A dash indicates that the gene was not significantly a [file pone.0000643.s005.zip › Corbo_TableS1/images/2610528A11Rik.gif]

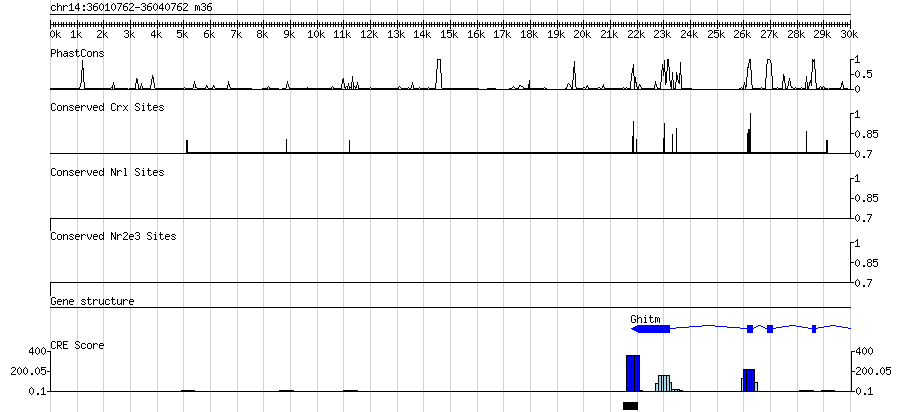

Supplement: Table S1 — Genes in the mouse photoreceptor transcription network. This table is a database which includes all genes dysregulated in Crx-/-, Nrl-/-, and/or Nr2e3-/- under high stringency criteria. Each row in the database represents a gene in the network and includes multiple links to additional types of information about that gene. ‘Large image gene +/− 15 Kb’ links to an image of our computational prediction of photoreceptor CREs in the genomic region 15 Kb upstream and downstream of the gene in question. ‘Closeup image TSS +/− 15 Kb’ links to an image of our computational prediction in the region 15 Kb on either side of the gene's TSS. Black bars within this image highlight the following regulatory peaks (if any are present): the peak closest to the TSS and the peak with the highest CRE score within this 30 Kb window. For those genes whose CRE predictions were tested experimentally, a red bar in this image indicates the location of the CRE that was tested. For Crx and Elovl4 the tested CRE is outside of this 30 Kb window and is therefore indicated in the ‘Large image’. ‘Links’ contains links to the UCSC genome browser, ENSEMBL, and NCBI database entries for the indicated gene, if available. ‘Max score (threshold of 200)’ contains the score of the highest predicted regulatory peak within the 30 Kb window around the gene's TSS. If this window does not contain any predicted peaks ≥ 200 (our cutoff threshold) no value is given (indicated by a dash). The next four columns of the table (‘Nr2e3-/-’ etc.) show the wild-type-to-mutant ratios of the averaged microarray scores for the given gene. For those genes to which more than one Affy tag correspond, the data for the first one listed under ‘Affy Mouse 430 2.0’ is given. Dark green = downregulated under high stringency (as described in METHODS); light green = downregulated under low stringency; red = upregulated under high stringency; orange = upregulated under low stringency. A dash indicates that the gene was not significantly a [file pone.0000643.s005.zip › Corbo_TableS1/images/2610528A11Rik_TSS.gif]

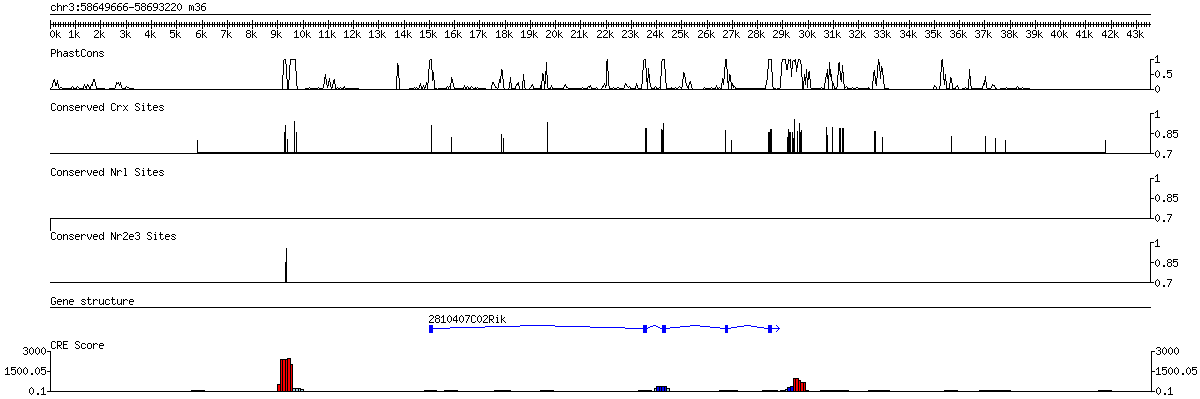

Supplement: Table S1 — Genes in the mouse photoreceptor transcription network. This table is a database which includes all genes dysregulated in Crx-/-, Nrl-/-, and/or Nr2e3-/- under high stringency criteria. Each row in the database represents a gene in the network and includes multiple links to additional types of information about that gene. ‘Large image gene +/− 15 Kb’ links to an image of our computational prediction of photoreceptor CREs in the genomic region 15 Kb upstream and downstream of the gene in question. ‘Closeup image TSS +/− 15 Kb’ links to an image of our computational prediction in the region 15 Kb on either side of the gene's TSS. Black bars within this image highlight the following regulatory peaks (if any are present): the peak closest to the TSS and the peak with the highest CRE score within this 30 Kb window. For those genes whose CRE predictions were tested experimentally, a red bar in this image indicates the location of the CRE that was tested. For Crx and Elovl4 the tested CRE is outside of this 30 Kb window and is therefore indicated in the ‘Large image’. ‘Links’ contains links to the UCSC genome browser, ENSEMBL, and NCBI database entries for the indicated gene, if available. ‘Max score (threshold of 200)’ contains the score of the highest predicted regulatory peak within the 30 Kb window around the gene's TSS. If this window does not contain any predicted peaks ≥ 200 (our cutoff threshold) no value is given (indicated by a dash). The next four columns of the table (‘Nr2e3-/-’ etc.) show the wild-type-to-mutant ratios of the averaged microarray scores for the given gene. For those genes to which more than one Affy tag correspond, the data for the first one listed under ‘Affy Mouse 430 2.0’ is given. Dark green = downregulated under high stringency (as described in METHODS); light green = downregulated under low stringency; red = upregulated under high stringency; orange = upregulated under low stringency. A dash indicates that the gene was not significantly a [file pone.0000643.s005.zip › Corbo_TableS1/images/2810407C02Rik.gif]

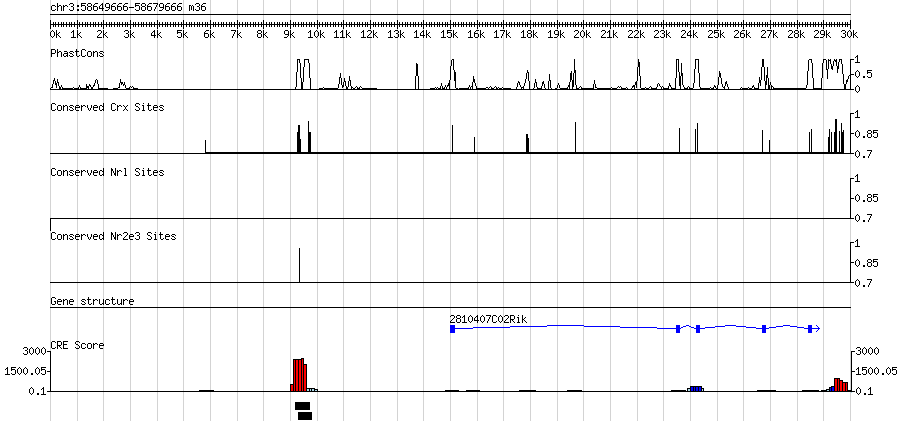

Supplement: Table S1 — Genes in the mouse photoreceptor transcription network. This table is a database which includes all genes dysregulated in Crx-/-, Nrl-/-, and/or Nr2e3-/- under high stringency criteria. Each row in the database represents a gene in the network and includes multiple links to additional types of information about that gene. ‘Large image gene +/− 15 Kb’ links to an image of our computational prediction of photoreceptor CREs in the genomic region 15 Kb upstream and downstream of the gene in question. ‘Closeup image TSS +/− 15 Kb’ links to an image of our computational prediction in the region 15 Kb on either side of the gene's TSS. Black bars within this image highlight the following regulatory peaks (if any are present): the peak closest to the TSS and the peak with the highest CRE score within this 30 Kb window. For those genes whose CRE predictions were tested experimentally, a red bar in this image indicates the location of the CRE that was tested. For Crx and Elovl4 the tested CRE is outside of this 30 Kb window and is therefore indicated in the ‘Large image’. ‘Links’ contains links to the UCSC genome browser, ENSEMBL, and NCBI database entries for the indicated gene, if available. ‘Max score (threshold of 200)’ contains the score of the highest predicted regulatory peak within the 30 Kb window around the gene's TSS. If this window does not contain any predicted peaks ≥ 200 (our cutoff threshold) no value is given (indicated by a dash). The next four columns of the table (‘Nr2e3-/-’ etc.) show the wild-type-to-mutant ratios of the averaged microarray scores for the given gene. For those genes to which more than one Affy tag correspond, the data for the first one listed under ‘Affy Mouse 430 2.0’ is given. Dark green = downregulated under high stringency (as described in METHODS); light green = downregulated under low stringency; red = upregulated under high stringency; orange = upregulated under low stringency. A dash indicates that the gene was not significantly a [file pone.0000643.s005.zip › Corbo_TableS1/images/2810407C02Rik_TSS.gif]

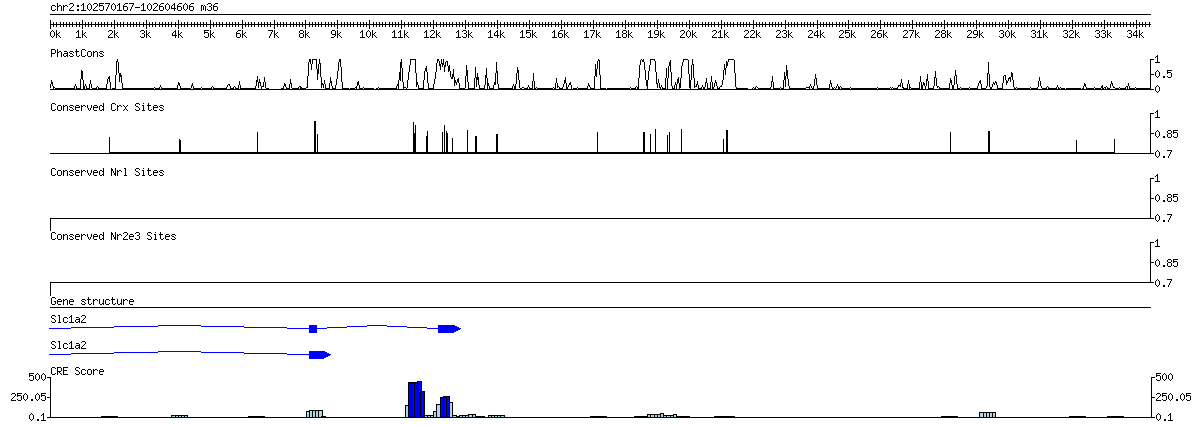

Supplement: Table S1 — Genes in the mouse photoreceptor transcription network. This table is a database which includes all genes dysregulated in Crx-/-, Nrl-/-, and/or Nr2e3-/- under high stringency criteria. Each row in the database represents a gene in the network and includes multiple links to additional types of information about that gene. ‘Large image gene +/− 15 Kb’ links to an image of our computational prediction of photoreceptor CREs in the genomic region 15 Kb upstream and downstream of the gene in question. ‘Closeup image TSS +/− 15 Kb’ links to an image of our computational prediction in the region 15 Kb on either side of the gene's TSS. Black bars within this image highlight the following regulatory peaks (if any are present): the peak closest to the TSS and the peak with the highest CRE score within this 30 Kb window. For those genes whose CRE predictions were tested experimentally, a red bar in this image indicates the location of the CRE that was tested. For Crx and Elovl4 the tested CRE is outside of this 30 Kb window and is therefore indicated in the ‘Large image’. ‘Links’ contains links to the UCSC genome browser, ENSEMBL, and NCBI database entries for the indicated gene, if available. ‘Max score (threshold of 200)’ contains the score of the highest predicted regulatory peak within the 30 Kb window around the gene's TSS. If this window does not contain any predicted peaks ≥ 200 (our cutoff threshold) no value is given (indicated by a dash). The next four columns of the table (‘Nr2e3-/-’ etc.) show the wild-type-to-mutant ratios of the averaged microarray scores for the given gene. For those genes to which more than one Affy tag correspond, the data for the first one listed under ‘Affy Mouse 430 2.0’ is given. Dark green = downregulated under high stringency (as described in METHODS); light green = downregulated under low stringency; red = upregulated under high stringency; orange = upregulated under low stringency. A dash indicates that the gene was not significantly a [file pone.0000643.s005.zip › Corbo_TableS1/images/2900019G14Rik.gif]

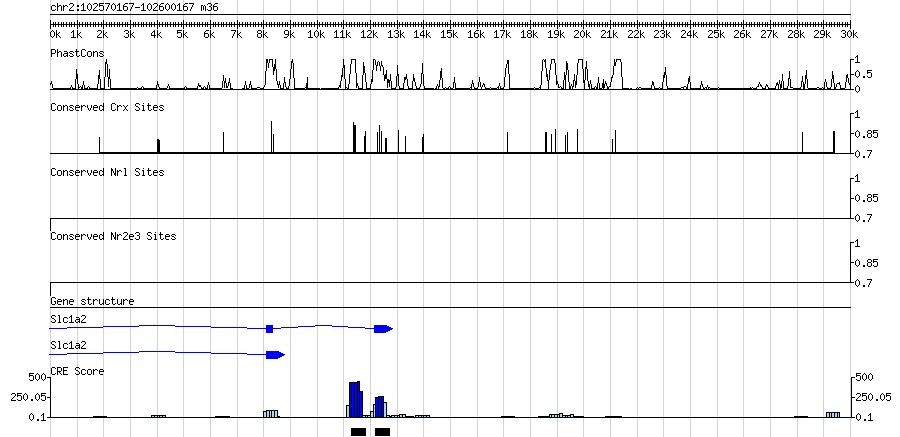

Supplement: Table S1 — Genes in the mouse photoreceptor transcription network. This table is a database which includes all genes dysregulated in Crx-/-, Nrl-/-, and/or Nr2e3-/- under high stringency criteria. Each row in the database represents a gene in the network and includes multiple links to additional types of information about that gene. ‘Large image gene +/− 15 Kb’ links to an image of our computational prediction of photoreceptor CREs in the genomic region 15 Kb upstream and downstream of the gene in question. ‘Closeup image TSS +/− 15 Kb’ links to an image of our computational prediction in the region 15 Kb on either side of the gene's TSS. Black bars within this image highlight the following regulatory peaks (if any are present): the peak closest to the TSS and the peak with the highest CRE score within this 30 Kb window. For those genes whose CRE predictions were tested experimentally, a red bar in this image indicates the location of the CRE that was tested. For Crx and Elovl4 the tested CRE is outside of this 30 Kb window and is therefore indicated in the ‘Large image’. ‘Links’ contains links to the UCSC genome browser, ENSEMBL, and NCBI database entries for the indicated gene, if available. ‘Max score (threshold of 200)’ contains the score of the highest predicted regulatory peak within the 30 Kb window around the gene's TSS. If this window does not contain any predicted peaks ≥ 200 (our cutoff threshold) no value is given (indicated by a dash). The next four columns of the table (‘Nr2e3-/-’ etc.) show the wild-type-to-mutant ratios of the averaged microarray scores for the given gene. For those genes to which more than one Affy tag correspond, the data for the first one listed under ‘Affy Mouse 430 2.0’ is given. Dark green = downregulated under high stringency (as described in METHODS); light green = downregulated under low stringency; red = upregulated under high stringency; orange = upregulated under low stringency. A dash indicates that the gene was not significantly a [file pone.0000643.s005.zip › Corbo_TableS1/images/2900019G14Rik_TSS.gif]

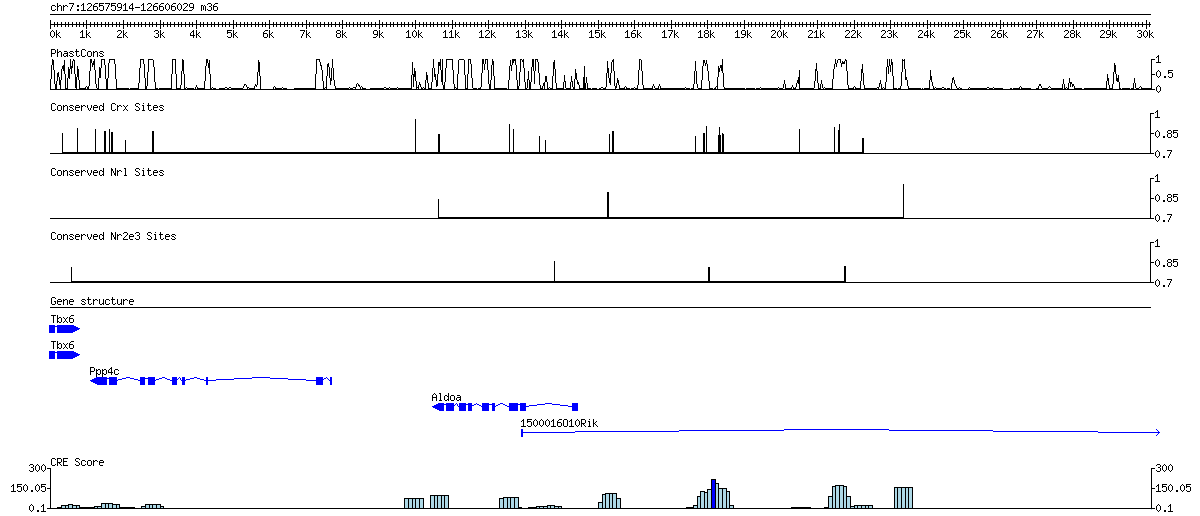

Supplement: Table S1 — Genes in the mouse photoreceptor transcription network. This table is a database which includes all genes dysregulated in Crx-/-, Nrl-/-, and/or Nr2e3-/- under high stringency criteria. Each row in the database represents a gene in the network and includes multiple links to additional types of information about that gene. ‘Large image gene +/− 15 Kb’ links to an image of our computational prediction of photoreceptor CREs in the genomic region 15 Kb upstream and downstream of the gene in question. ‘Closeup image TSS +/− 15 Kb’ links to an image of our computational prediction in the region 15 Kb on either side of the gene's TSS. Black bars within this image highlight the following regulatory peaks (if any are present): the peak closest to the TSS and the peak with the highest CRE score within this 30 Kb window. For those genes whose CRE predictions were tested experimentally, a red bar in this image indicates the location of the CRE that was tested. For Crx and Elovl4 the tested CRE is outside of this 30 Kb window and is therefore indicated in the ‘Large image’. ‘Links’ contains links to the UCSC genome browser, ENSEMBL, and NCBI database entries for the indicated gene, if available. ‘Max score (threshold of 200)’ contains the score of the highest predicted regulatory peak within the 30 Kb window around the gene's TSS. If this window does not contain any predicted peaks ≥ 200 (our cutoff threshold) no value is given (indicated by a dash). The next four columns of the table (‘Nr2e3-/-’ etc.) show the wild-type-to-mutant ratios of the averaged microarray scores for the given gene. For those genes to which more than one Affy tag correspond, the data for the first one listed under ‘Affy Mouse 430 2.0’ is given. Dark green = downregulated under high stringency (as described in METHODS); light green = downregulated under low stringency; red = upregulated under high stringency; orange = upregulated under low stringency. A dash indicates that the gene was not significantly a [file pone.0000643.s005.zip › Corbo_TableS1/images/2900046D03Rik.gif]

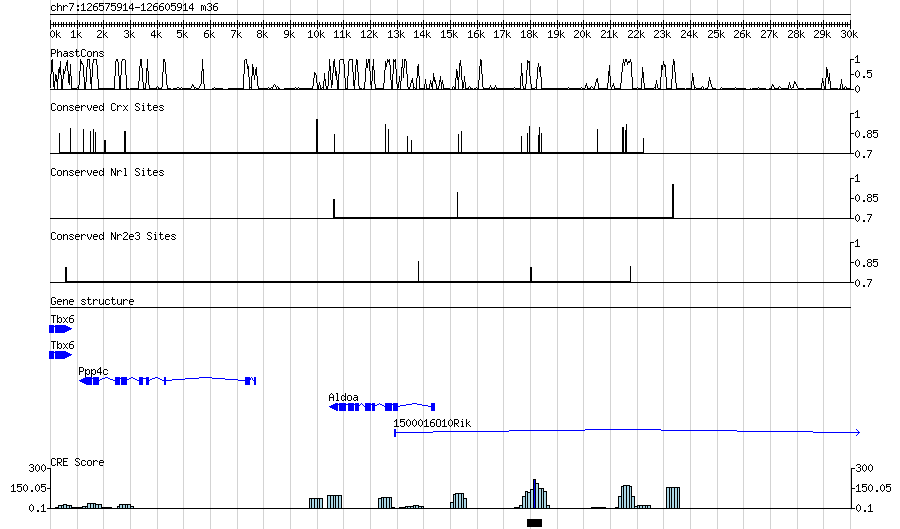

Supplement: Table S1 — Genes in the mouse photoreceptor transcription network. This table is a database which includes all genes dysregulated in Crx-/-, Nrl-/-, and/or Nr2e3-/- under high stringency criteria. Each row in the database represents a gene in the network and includes multiple links to additional types of information about that gene. ‘Large image gene +/− 15 Kb’ links to an image of our computational prediction of photoreceptor CREs in the genomic region 15 Kb upstream and downstream of the gene in question. ‘Closeup image TSS +/− 15 Kb’ links to an image of our computational prediction in the region 15 Kb on either side of the gene's TSS. Black bars within this image highlight the following regulatory peaks (if any are present): the peak closest to the TSS and the peak with the highest CRE score within this 30 Kb window. For those genes whose CRE predictions were tested experimentally, a red bar in this image indicates the location of the CRE that was tested. For Crx and Elovl4 the tested CRE is outside of this 30 Kb window and is therefore indicated in the ‘Large image’. ‘Links’ contains links to the UCSC genome browser, ENSEMBL, and NCBI database entries for the indicated gene, if available. ‘Max score (threshold of 200)’ contains the score of the highest predicted regulatory peak within the 30 Kb window around the gene's TSS. If this window does not contain any predicted peaks ≥ 200 (our cutoff threshold) no value is given (indicated by a dash). The next four columns of the table (‘Nr2e3-/-’ etc.) show the wild-type-to-mutant ratios of the averaged microarray scores for the given gene. For those genes to which more than one Affy tag correspond, the data for the first one listed under ‘Affy Mouse 430 2.0’ is given. Dark green = downregulated under high stringency (as described in METHODS); light green = downregulated under low stringency; red = upregulated under high stringency; orange = upregulated under low stringency. A dash indicates that the gene was not significantly a [file pone.0000643.s005.zip › Corbo_TableS1/images/2900046D03Rik_TSS.gif]

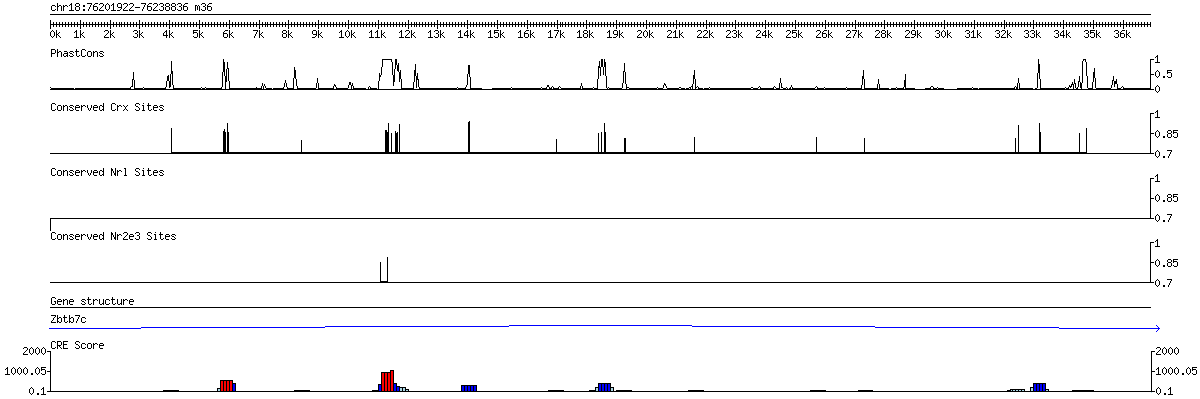

Supplement: Table S1 — Genes in the mouse photoreceptor transcription network. This table is a database which includes all genes dysregulated in Crx-/-, Nrl-/-, and/or Nr2e3-/- under high stringency criteria. Each row in the database represents a gene in the network and includes multiple links to additional types of information about that gene. ‘Large image gene +/− 15 Kb’ links to an image of our computational prediction of photoreceptor CREs in the genomic region 15 Kb upstream and downstream of the gene in question. ‘Closeup image TSS +/− 15 Kb’ links to an image of our computational prediction in the region 15 Kb on either side of the gene's TSS. Black bars within this image highlight the following regulatory peaks (if any are present): the peak closest to the TSS and the peak with the highest CRE score within this 30 Kb window. For those genes whose CRE predictions were tested experimentally, a red bar in this image indicates the location of the CRE that was tested. For Crx and Elovl4 the tested CRE is outside of this 30 Kb window and is therefore indicated in the ‘Large image’. ‘Links’ contains links to the UCSC genome browser, ENSEMBL, and NCBI database entries for the indicated gene, if available. ‘Max score (threshold of 200)’ contains the score of the highest predicted regulatory peak within the 30 Kb window around the gene's TSS. If this window does not contain any predicted peaks ≥ 200 (our cutoff threshold) no value is given (indicated by a dash). The next four columns of the table (‘Nr2e3-/-’ etc.) show the wild-type-to-mutant ratios of the averaged microarray scores for the given gene. For those genes to which more than one Affy tag correspond, the data for the first one listed under ‘Affy Mouse 430 2.0’ is given. Dark green = downregulated under high stringency (as described in METHODS); light green = downregulated under low stringency; red = upregulated under high stringency; orange = upregulated under low stringency. A dash indicates that the gene was not significantly a [file pone.0000643.s005.zip › Corbo_TableS1/images/2900057B20Rik.gif]

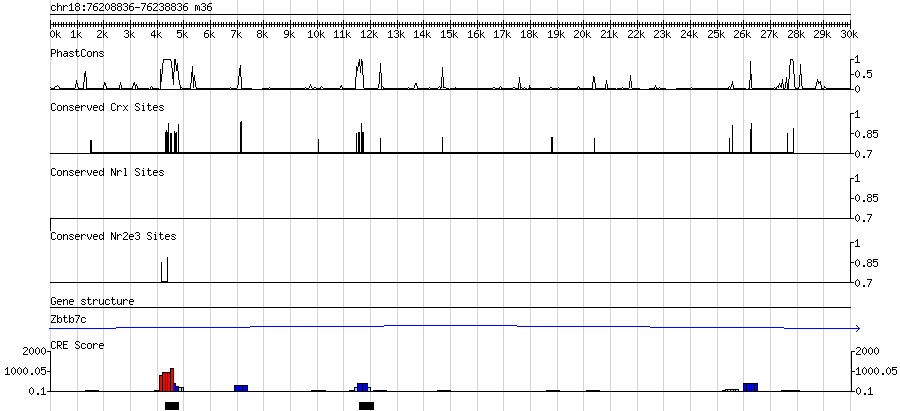

Supplement: Table S1 — Genes in the mouse photoreceptor transcription network. This table is a database which includes all genes dysregulated in Crx-/-, Nrl-/-, and/or Nr2e3-/- under high stringency criteria. Each row in the database represents a gene in the network and includes multiple links to additional types of information about that gene. ‘Large image gene +/− 15 Kb’ links to an image of our computational prediction of photoreceptor CREs in the genomic region 15 Kb upstream and downstream of the gene in question. ‘Closeup image TSS +/− 15 Kb’ links to an image of our computational prediction in the region 15 Kb on either side of the gene's TSS. Black bars within this image highlight the following regulatory peaks (if any are present): the peak closest to the TSS and the peak with the highest CRE score within this 30 Kb window. For those genes whose CRE predictions were tested experimentally, a red bar in this image indicates the location of the CRE that was tested. For Crx and Elovl4 the tested CRE is outside of this 30 Kb window and is therefore indicated in the ‘Large image’. ‘Links’ contains links to the UCSC genome browser, ENSEMBL, and NCBI database entries for the indicated gene, if available. ‘Max score (threshold of 200)’ contains the score of the highest predicted regulatory peak within the 30 Kb window around the gene's TSS. If this window does not contain any predicted peaks ≥ 200 (our cutoff threshold) no value is given (indicated by a dash). The next four columns of the table (‘Nr2e3-/-’ etc.) show the wild-type-to-mutant ratios of the averaged microarray scores for the given gene. For those genes to which more than one Affy tag correspond, the data for the first one listed under ‘Affy Mouse 430 2.0’ is given. Dark green = downregulated under high stringency (as described in METHODS); light green = downregulated under low stringency; red = upregulated under high stringency; orange = upregulated under low stringency. A dash indicates that the gene was not significantly a [file pone.0000643.s005.zip › Corbo_TableS1/images/2900057B20Rik_TSS.gif]

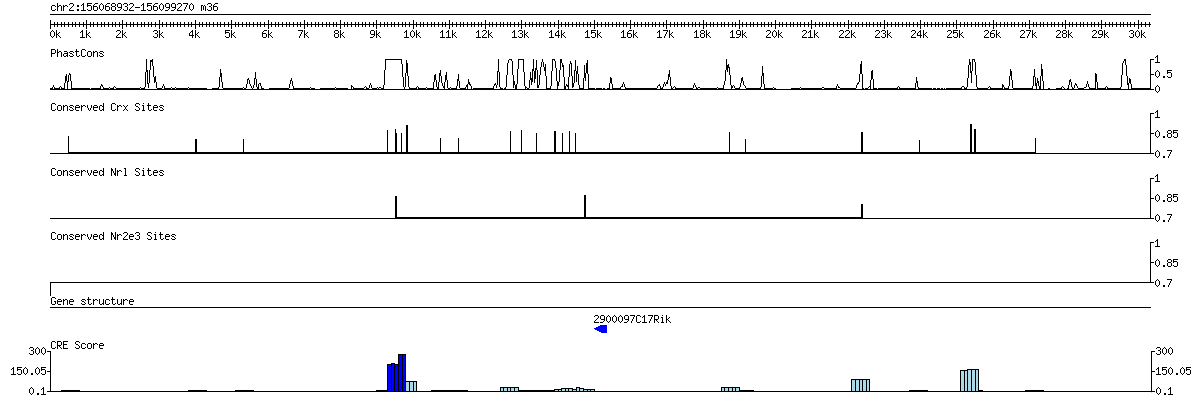

Supplement: Table S1 — Genes in the mouse photoreceptor transcription network. This table is a database which includes all genes dysregulated in Crx-/-, Nrl-/-, and/or Nr2e3-/- under high stringency criteria. Each row in the database represents a gene in the network and includes multiple links to additional types of information about that gene. ‘Large image gene +/− 15 Kb’ links to an image of our computational prediction of photoreceptor CREs in the genomic region 15 Kb upstream and downstream of the gene in question. ‘Closeup image TSS +/− 15 Kb’ links to an image of our computational prediction in the region 15 Kb on either side of the gene's TSS. Black bars within this image highlight the following regulatory peaks (if any are present): the peak closest to the TSS and the peak with the highest CRE score within this 30 Kb window. For those genes whose CRE predictions were tested experimentally, a red bar in this image indicates the location of the CRE that was tested. For Crx and Elovl4 the tested CRE is outside of this 30 Kb window and is therefore indicated in the ‘Large image’. ‘Links’ contains links to the UCSC genome browser, ENSEMBL, and NCBI database entries for the indicated gene, if available. ‘Max score (threshold of 200)’ contains the score of the highest predicted regulatory peak within the 30 Kb window around the gene's TSS. If this window does not contain any predicted peaks ≥ 200 (our cutoff threshold) no value is given (indicated by a dash). The next four columns of the table (‘Nr2e3-/-’ etc.) show the wild-type-to-mutant ratios of the averaged microarray scores for the given gene. For those genes to which more than one Affy tag correspond, the data for the first one listed under ‘Affy Mouse 430 2.0’ is given. Dark green = downregulated under high stringency (as described in METHODS); light green = downregulated under low stringency; red = upregulated under high stringency; orange = upregulated under low stringency. A dash indicates that the gene was not significantly a [file pone.0000643.s005.zip › Corbo_TableS1/images/2900097C17Rik.gif]

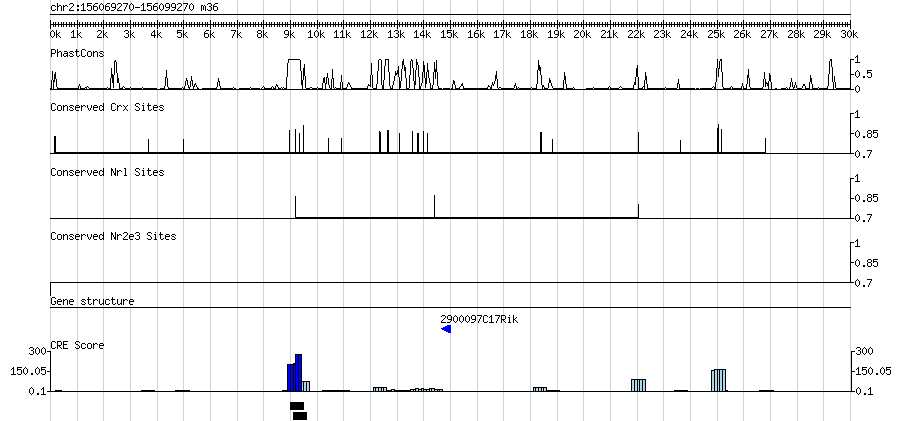

Supplement: Table S1 — Genes in the mouse photoreceptor transcription network. This table is a database which includes all genes dysregulated in Crx-/-, Nrl-/-, and/or Nr2e3-/- under high stringency criteria. Each row in the database represents a gene in the network and includes multiple links to additional types of information about that gene. ‘Large image gene +/− 15 Kb’ links to an image of our computational prediction of photoreceptor CREs in the genomic region 15 Kb upstream and downstream of the gene in question. ‘Closeup image TSS +/− 15 Kb’ links to an image of our computational prediction in the region 15 Kb on either side of the gene's TSS. Black bars within this image highlight the following regulatory peaks (if any are present): the peak closest to the TSS and the peak with the highest CRE score within this 30 Kb window. For those genes whose CRE predictions were tested experimentally, a red bar in this image indicates the location of the CRE that was tested. For Crx and Elovl4 the tested CRE is outside of this 30 Kb window and is therefore indicated in the ‘Large image’. ‘Links’ contains links to the UCSC genome browser, ENSEMBL, and NCBI database entries for the indicated gene, if available. ‘Max score (threshold of 200)’ contains the score of the highest predicted regulatory peak within the 30 Kb window around the gene's TSS. If this window does not contain any predicted peaks ≥ 200 (our cutoff threshold) no value is given (indicated by a dash). The next four columns of the table (‘Nr2e3-/-’ etc.) show the wild-type-to-mutant ratios of the averaged microarray scores for the given gene. For those genes to which more than one Affy tag correspond, the data for the first one listed under ‘Affy Mouse 430 2.0’ is given. Dark green = downregulated under high stringency (as described in METHODS); light green = downregulated under low stringency; red = upregulated under high stringency; orange = upregulated under low stringency. A dash indicates that the gene was not significantly a [file pone.0000643.s005.zip › Corbo_TableS1/images/2900097C17Rik_TSS.gif]

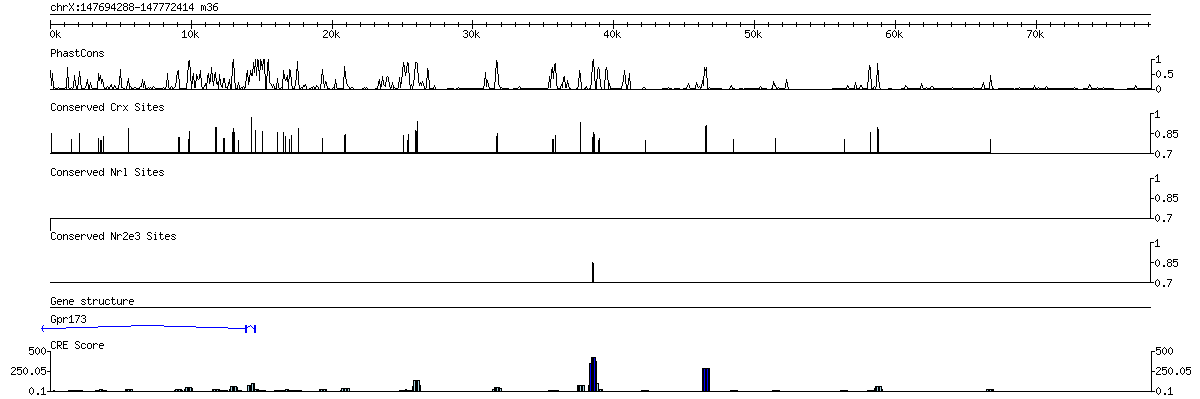

Supplement: Table S1 — Genes in the mouse photoreceptor transcription network. This table is a database which includes all genes dysregulated in Crx-/-, Nrl-/-, and/or Nr2e3-/- under high stringency criteria. Each row in the database represents a gene in the network and includes multiple links to additional types of information about that gene. ‘Large image gene +/− 15 Kb’ links to an image of our computational prediction of photoreceptor CREs in the genomic region 15 Kb upstream and downstream of the gene in question. ‘Closeup image TSS +/− 15 Kb’ links to an image of our computational prediction in the region 15 Kb on either side of the gene's TSS. Black bars within this image highlight the following regulatory peaks (if any are present): the peak closest to the TSS and the peak with the highest CRE score within this 30 Kb window. For those genes whose CRE predictions were tested experimentally, a red bar in this image indicates the location of the CRE that was tested. For Crx and Elovl4 the tested CRE is outside of this 30 Kb window and is therefore indicated in the ‘Large image’. ‘Links’ contains links to the UCSC genome browser, ENSEMBL, and NCBI database entries for the indicated gene, if available. ‘Max score (threshold of 200)’ contains the score of the highest predicted regulatory peak within the 30 Kb window around the gene's TSS. If this window does not contain any predicted peaks ≥ 200 (our cutoff threshold) no value is given (indicated by a dash). The next four columns of the table (‘Nr2e3-/-’ etc.) show the wild-type-to-mutant ratios of the averaged microarray scores for the given gene. For those genes to which more than one Affy tag correspond, the data for the first one listed under ‘Affy Mouse 430 2.0’ is given. Dark green = downregulated under high stringency (as described in METHODS); light green = downregulated under low stringency; red = upregulated under high stringency; orange = upregulated under low stringency. A dash indicates that the gene was not significantly a [file pone.0000643.s005.zip › Corbo_TableS1/images/3010001F23Rik.gif]

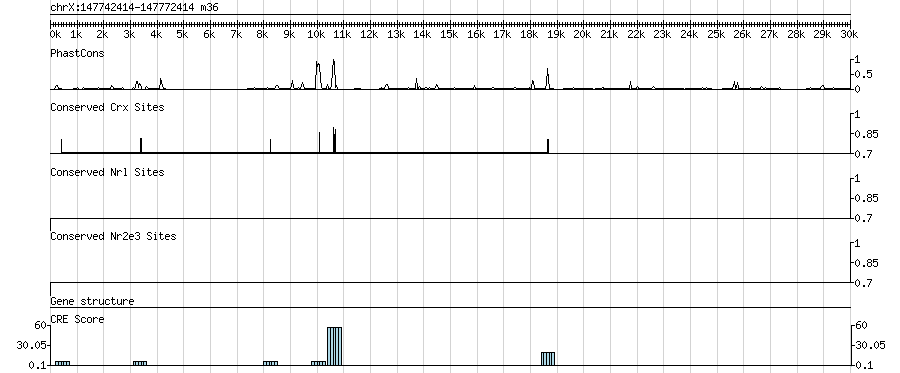

Supplement: Table S1 — Genes in the mouse photoreceptor transcription network. This table is a database which includes all genes dysregulated in Crx-/-, Nrl-/-, and/or Nr2e3-/- under high stringency criteria. Each row in the database represents a gene in the network and includes multiple links to additional types of information about that gene. ‘Large image gene +/− 15 Kb’ links to an image of our computational prediction of photoreceptor CREs in the genomic region 15 Kb upstream and downstream of the gene in question. ‘Closeup image TSS +/− 15 Kb’ links to an image of our computational prediction in the region 15 Kb on either side of the gene's TSS. Black bars within this image highlight the following regulatory peaks (if any are present): the peak closest to the TSS and the peak with the highest CRE score within this 30 Kb window. For those genes whose CRE predictions were tested experimentally, a red bar in this image indicates the location of the CRE that was tested. For Crx and Elovl4 the tested CRE is outside of this 30 Kb window and is therefore indicated in the ‘Large image’. ‘Links’ contains links to the UCSC genome browser, ENSEMBL, and NCBI database entries for the indicated gene, if available. ‘Max score (threshold of 200)’ contains the score of the highest predicted regulatory peak within the 30 Kb window around the gene's TSS. If this window does not contain any predicted peaks ≥ 200 (our cutoff threshold) no value is given (indicated by a dash). The next four columns of the table (‘Nr2e3-/-’ etc.) show the wild-type-to-mutant ratios of the averaged microarray scores for the given gene. For those genes to which more than one Affy tag correspond, the data for the first one listed under ‘Affy Mouse 430 2.0’ is given. Dark green = downregulated under high stringency (as described in METHODS); light green = downregulated under low stringency; red = upregulated under high stringency; orange = upregulated under low stringency. A dash indicates that the gene was not significantly a [file pone.0000643.s005.zip › Corbo_TableS1/images/3010001F23Rik_TSS.gif]

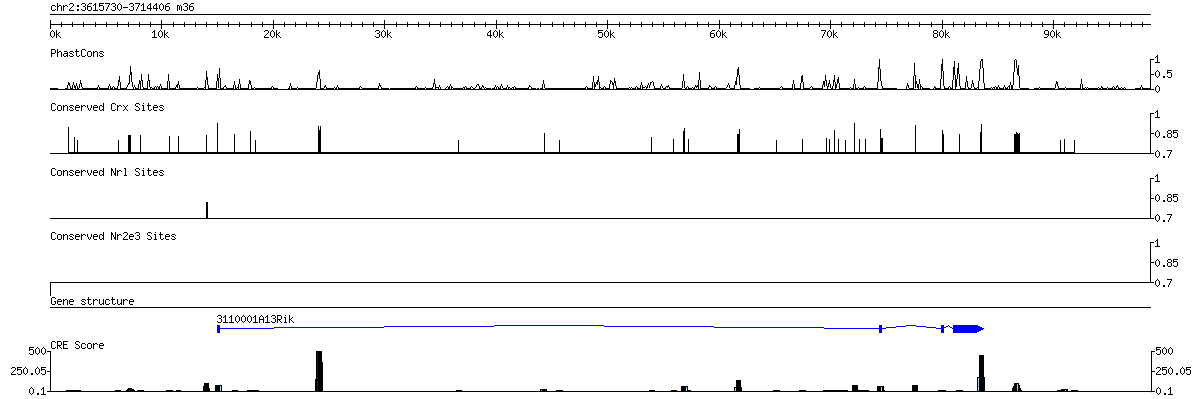

Supplement: Table S1 — Genes in the mouse photoreceptor transcription network. This table is a database which includes all genes dysregulated in Crx-/-, Nrl-/-, and/or Nr2e3-/- under high stringency criteria. Each row in the database represents a gene in the network and includes multiple links to additional types of information about that gene. ‘Large image gene +/− 15 Kb’ links to an image of our computational prediction of photoreceptor CREs in the genomic region 15 Kb upstream and downstream of the gene in question. ‘Closeup image TSS +/− 15 Kb’ links to an image of our computational prediction in the region 15 Kb on either side of the gene's TSS. Black bars within this image highlight the following regulatory peaks (if any are present): the peak closest to the TSS and the peak with the highest CRE score within this 30 Kb window. For those genes whose CRE predictions were tested experimentally, a red bar in this image indicates the location of the CRE that was tested. For Crx and Elovl4 the tested CRE is outside of this 30 Kb window and is therefore indicated in the ‘Large image’. ‘Links’ contains links to the UCSC genome browser, ENSEMBL, and NCBI database entries for the indicated gene, if available. ‘Max score (threshold of 200)’ contains the score of the highest predicted regulatory peak within the 30 Kb window around the gene's TSS. If this window does not contain any predicted peaks ≥ 200 (our cutoff threshold) no value is given (indicated by a dash). The next four columns of the table (‘Nr2e3-/-’ etc.) show the wild-type-to-mutant ratios of the averaged microarray scores for the given gene. For those genes to which more than one Affy tag correspond, the data for the first one listed under ‘Affy Mouse 430 2.0’ is given. Dark green = downregulated under high stringency (as described in METHODS); light green = downregulated under low stringency; red = upregulated under high stringency; orange = upregulated under low stringency. A dash indicates that the gene was not significantly a [file pone.0000643.s005.zip › Corbo_TableS1/images/3110001A13Rik.gif]

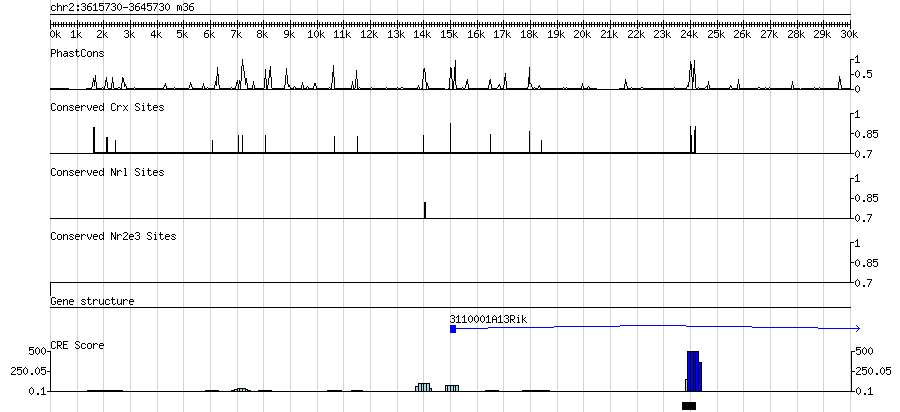

Supplement: Table S1 — Genes in the mouse photoreceptor transcription network. This table is a database which includes all genes dysregulated in Crx-/-, Nrl-/-, and/or Nr2e3-/- under high stringency criteria. Each row in the database represents a gene in the network and includes multiple links to additional types of information about that gene. ‘Large image gene +/− 15 Kb’ links to an image of our computational prediction of photoreceptor CREs in the genomic region 15 Kb upstream and downstream of the gene in question. ‘Closeup image TSS +/− 15 Kb’ links to an image of our computational prediction in the region 15 Kb on either side of the gene's TSS. Black bars within this image highlight the following regulatory peaks (if any are present): the peak closest to the TSS and the peak with the highest CRE score within this 30 Kb window. For those genes whose CRE predictions were tested experimentally, a red bar in this image indicates the location of the CRE that was tested. For Crx and Elovl4 the tested CRE is outside of this 30 Kb window and is therefore indicated in the ‘Large image’. ‘Links’ contains links to the UCSC genome browser, ENSEMBL, and NCBI database entries for the indicated gene, if available. ‘Max score (threshold of 200)’ contains the score of the highest predicted regulatory peak within the 30 Kb window around the gene's TSS. If this window does not contain any predicted peaks ≥ 200 (our cutoff threshold) no value is given (indicated by a dash). The next four columns of the table (‘Nr2e3-/-’ etc.) show the wild-type-to-mutant ratios of the averaged microarray scores for the given gene. For those genes to which more than one Affy tag correspond, the data for the first one listed under ‘Affy Mouse 430 2.0’ is given. Dark green = downregulated under high stringency (as described in METHODS); light green = downregulated under low stringency; red = upregulated under high stringency; orange = upregulated under low stringency. A dash indicates that the gene was not significantly a [file pone.0000643.s005.zip › Corbo_TableS1/images/3110001A13Rik_TSS.gif]

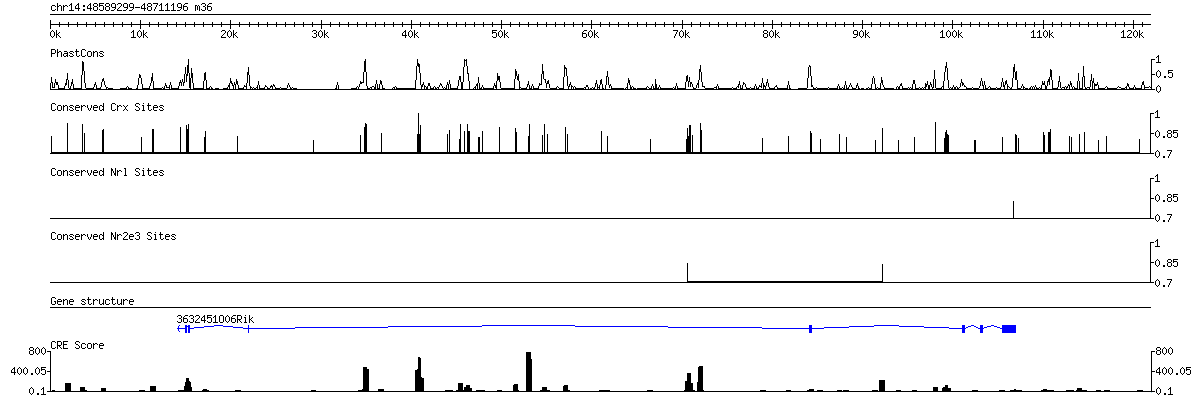

Supplement: Table S1 — Genes in the mouse photoreceptor transcription network. This table is a database which includes all genes dysregulated in Crx-/-, Nrl-/-, and/or Nr2e3-/- under high stringency criteria. Each row in the database represents a gene in the network and includes multiple links to additional types of information about that gene. ‘Large image gene +/− 15 Kb’ links to an image of our computational prediction of photoreceptor CREs in the genomic region 15 Kb upstream and downstream of the gene in question. ‘Closeup image TSS +/− 15 Kb’ links to an image of our computational prediction in the region 15 Kb on either side of the gene's TSS. Black bars within this image highlight the following regulatory peaks (if any are present): the peak closest to the TSS and the peak with the highest CRE score within this 30 Kb window. For those genes whose CRE predictions were tested experimentally, a red bar in this image indicates the location of the CRE that was tested. For Crx and Elovl4 the tested CRE is outside of this 30 Kb window and is therefore indicated in the ‘Large image’. ‘Links’ contains links to the UCSC genome browser, ENSEMBL, and NCBI database entries for the indicated gene, if available. ‘Max score (threshold of 200)’ contains the score of the highest predicted regulatory peak within the 30 Kb window around the gene's TSS. If this window does not contain any predicted peaks ≥ 200 (our cutoff threshold) no value is given (indicated by a dash). The next four columns of the table (‘Nr2e3-/-’ etc.) show the wild-type-to-mutant ratios of the averaged microarray scores for the given gene. For those genes to which more than one Affy tag correspond, the data for the first one listed under ‘Affy Mouse 430 2.0’ is given. Dark green = downregulated under high stringency (as described in METHODS); light green = downregulated under low stringency; red = upregulated under high stringency; orange = upregulated under low stringency. A dash indicates that the gene was not significantly a [file pone.0000643.s005.zip › Corbo_TableS1/images/3632451O06Rik.gif]

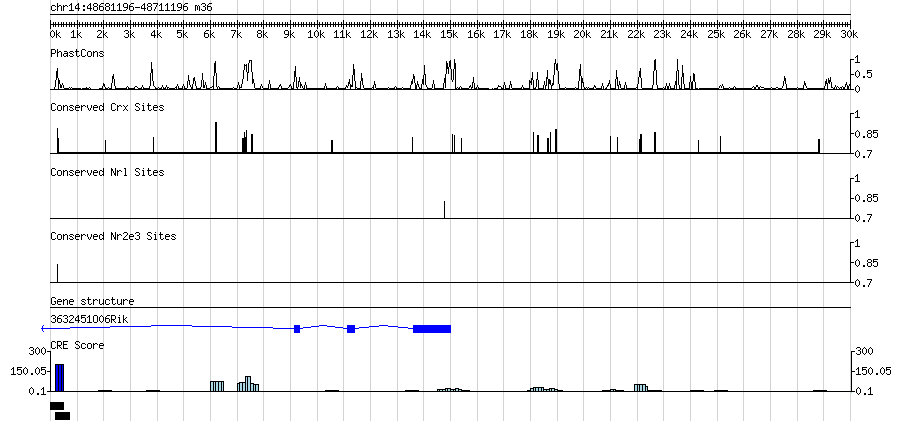

Supplement: Table S1 — Genes in the mouse photoreceptor transcription network. This table is a database which includes all genes dysregulated in Crx-/-, Nrl-/-, and/or Nr2e3-/- under high stringency criteria. Each row in the database represents a gene in the network and includes multiple links to additional types of information about that gene. ‘Large image gene +/− 15 Kb’ links to an image of our computational prediction of photoreceptor CREs in the genomic region 15 Kb upstream and downstream of the gene in question. ‘Closeup image TSS +/− 15 Kb’ links to an image of our computational prediction in the region 15 Kb on either side of the gene's TSS. Black bars within this image highlight the following regulatory peaks (if any are present): the peak closest to the TSS and the peak with the highest CRE score within this 30 Kb window. For those genes whose CRE predictions were tested experimentally, a red bar in this image indicates the location of the CRE that was tested. For Crx and Elovl4 the tested CRE is outside of this 30 Kb window and is therefore indicated in the ‘Large image’. ‘Links’ contains links to the UCSC genome browser, ENSEMBL, and NCBI database entries for the indicated gene, if available. ‘Max score (threshold of 200)’ contains the score of the highest predicted regulatory peak within the 30 Kb window around the gene's TSS. If this window does not contain any predicted peaks ≥ 200 (our cutoff threshold) no value is given (indicated by a dash). The next four columns of the table (‘Nr2e3-/-’ etc.) show the wild-type-to-mutant ratios of the averaged microarray scores for the given gene. For those genes to which more than one Affy tag correspond, the data for the first one listed under ‘Affy Mouse 430 2.0’ is given. Dark green = downregulated under high stringency (as described in METHODS); light green = downregulated under low stringency; red = upregulated under high stringency; orange = upregulated under low stringency. A dash indicates that the gene was not significantly a [file pone.0000643.s005.zip › Corbo_TableS1/images/3632451O06Rik_TSS.gif]

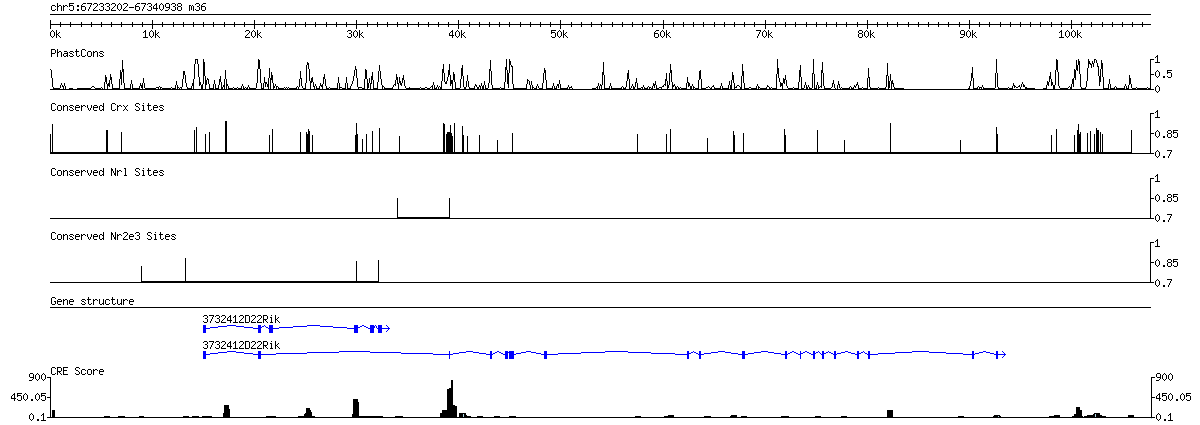

Supplement: Table S1 — Genes in the mouse photoreceptor transcription network. This table is a database which includes all genes dysregulated in Crx-/-, Nrl-/-, and/or Nr2e3-/- under high stringency criteria. Each row in the database represents a gene in the network and includes multiple links to additional types of information about that gene. ‘Large image gene +/− 15 Kb’ links to an image of our computational prediction of photoreceptor CREs in the genomic region 15 Kb upstream and downstream of the gene in question. ‘Closeup image TSS +/− 15 Kb’ links to an image of our computational prediction in the region 15 Kb on either side of the gene's TSS. Black bars within this image highlight the following regulatory peaks (if any are present): the peak closest to the TSS and the peak with the highest CRE score within this 30 Kb window. For those genes whose CRE predictions were tested experimentally, a red bar in this image indicates the location of the CRE that was tested. For Crx and Elovl4 the tested CRE is outside of this 30 Kb window and is therefore indicated in the ‘Large image’. ‘Links’ contains links to the UCSC genome browser, ENSEMBL, and NCBI database entries for the indicated gene, if available. ‘Max score (threshold of 200)’ contains the score of the highest predicted regulatory peak within the 30 Kb window around the gene's TSS. If this window does not contain any predicted peaks ≥ 200 (our cutoff threshold) no value is given (indicated by a dash). The next four columns of the table (‘Nr2e3-/-’ etc.) show the wild-type-to-mutant ratios of the averaged microarray scores for the given gene. For those genes to which more than one Affy tag correspond, the data for the first one listed under ‘Affy Mouse 430 2.0’ is given. Dark green = downregulated under high stringency (as described in METHODS); light green = downregulated under low stringency; red = upregulated under high stringency; orange = upregulated under low stringency. A dash indicates that the gene was not significantly a [file pone.0000643.s005.zip › Corbo_TableS1/images/3732412D22Rik.gif]

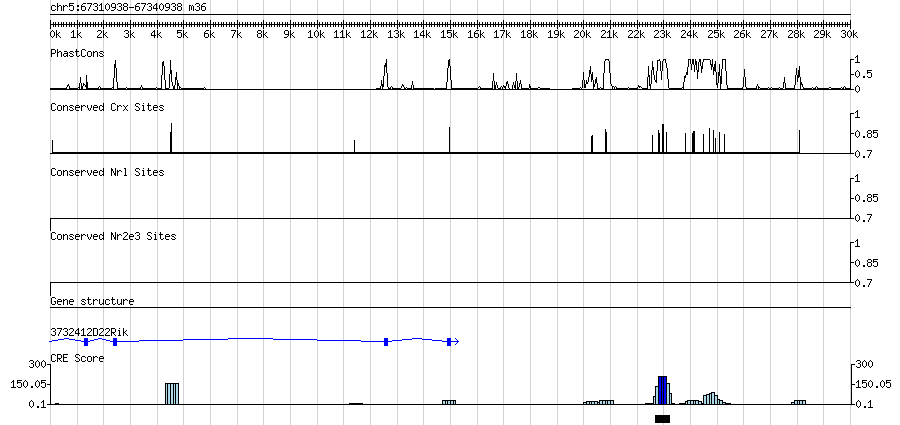

Supplement: Table S1 — Genes in the mouse photoreceptor transcription network. This table is a database which includes all genes dysregulated in Crx-/-, Nrl-/-, and/or Nr2e3-/- under high stringency criteria. Each row in the database represents a gene in the network and includes multiple links to additional types of information about that gene. ‘Large image gene +/− 15 Kb’ links to an image of our computational prediction of photoreceptor CREs in the genomic region 15 Kb upstream and downstream of the gene in question. ‘Closeup image TSS +/− 15 Kb’ links to an image of our computational prediction in the region 15 Kb on either side of the gene's TSS. Black bars within this image highlight the following regulatory peaks (if any are present): the peak closest to the TSS and the peak with the highest CRE score within this 30 Kb window. For those genes whose CRE predictions were tested experimentally, a red bar in this image indicates the location of the CRE that was tested. For Crx and Elovl4 the tested CRE is outside of this 30 Kb window and is therefore indicated in the ‘Large image’. ‘Links’ contains links to the UCSC genome browser, ENSEMBL, and NCBI database entries for the indicated gene, if available. ‘Max score (threshold of 200)’ contains the score of the highest predicted regulatory peak within the 30 Kb window around the gene's TSS. If this window does not contain any predicted peaks ≥ 200 (our cutoff threshold) no value is given (indicated by a dash). The next four columns of the table (‘Nr2e3-/-’ etc.) show the wild-type-to-mutant ratios of the averaged microarray scores for the given gene. For those genes to which more than one Affy tag correspond, the data for the first one listed under ‘Affy Mouse 430 2.0’ is given. Dark green = downregulated under high stringency (as described in METHODS); light green = downregulated under low stringency; red = upregulated under high stringency; orange = upregulated under low stringency. A dash indicates that the gene was not significantly a [file pone.0000643.s005.zip › Corbo_TableS1/images/3732412D22Rik_TSS.gif]

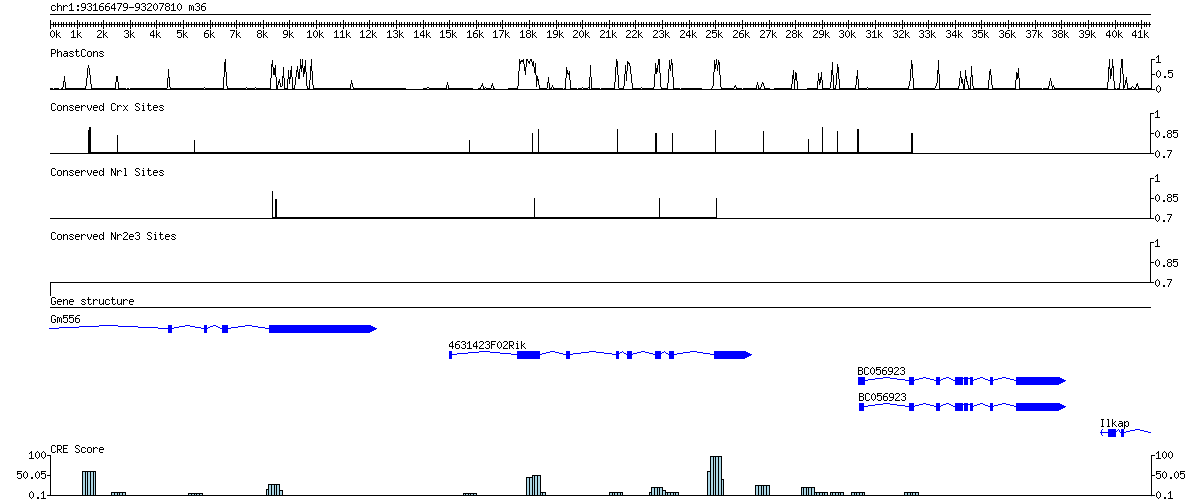

Supplement: Table S1 — Genes in the mouse photoreceptor transcription network. This table is a database which includes all genes dysregulated in Crx-/-, Nrl-/-, and/or Nr2e3-/- under high stringency criteria. Each row in the database represents a gene in the network and includes multiple links to additional types of information about that gene. ‘Large image gene +/− 15 Kb’ links to an image of our computational prediction of photoreceptor CREs in the genomic region 15 Kb upstream and downstream of the gene in question. ‘Closeup image TSS +/− 15 Kb’ links to an image of our computational prediction in the region 15 Kb on either side of the gene's TSS. Black bars within this image highlight the following regulatory peaks (if any are present): the peak closest to the TSS and the peak with the highest CRE score within this 30 Kb window. For those genes whose CRE predictions were tested experimentally, a red bar in this image indicates the location of the CRE that was tested. For Crx and Elovl4 the tested CRE is outside of this 30 Kb window and is therefore indicated in the ‘Large image’. ‘Links’ contains links to the UCSC genome browser, ENSEMBL, and NCBI database entries for the indicated gene, if available. ‘Max score (threshold of 200)’ contains the score of the highest predicted regulatory peak within the 30 Kb window around the gene's TSS. If this window does not contain any predicted peaks ≥ 200 (our cutoff threshold) no value is given (indicated by a dash). The next four columns of the table (‘Nr2e3-/-’ etc.) show the wild-type-to-mutant ratios of the averaged microarray scores for the given gene. For those genes to which more than one Affy tag correspond, the data for the first one listed under ‘Affy Mouse 430 2.0’ is given. Dark green = downregulated under high stringency (as described in METHODS); light green = downregulated under low stringency; red = upregulated under high stringency; orange = upregulated under low stringency. A dash indicates that the gene was not significantly a [file pone.0000643.s005.zip › Corbo_TableS1/images/4631423F02Rik.gif]
