# Supplementary material for: The Cis-regulatory Logic of the Mammalian Photoreceptor Transcriptional Network
Source: PLoS One. 2007 Jul 25;2(7):e643. doi: 10.1371/journal.pone.0000643 (PMC1916400; doi:10.1371/journal.pone.0000643)
Supplement: Table S1 — Genes in the mouse photoreceptor transcription network. This table is a database which includes all genes dysregulated in Crx-/-, Nrl-/-, and/or Nr2e3-/- under high stringency criteria. Each row in the database represents a gene in the network and includes multiple links to additional types of information about that gene. ‘Large image gene +/− 15 Kb’ links to an image of our computational prediction of photoreceptor CREs in the genomic region 15 Kb upstream and downstream of the gene in question. ‘Closeup image TSS +/− 15 Kb’ links to an image of our computational prediction in the region 15 Kb on either side of the gene's TSS. Black bars within this image highlight the following regulatory peaks (if any are present): the peak closest to the TSS and the peak with the highest CRE score within this 30 Kb window. For those genes whose CRE predictions were tested experimentally, a red bar in this image indicates the location of the CRE that was tested. For Crx and Elovl4 the tested CRE is outside of this 30 Kb window and is therefore indicated in the ‘Large image’. ‘Links’ contains links to the UCSC genome browser, ENSEMBL, and NCBI database entries for the indicated gene, if available. ‘Max score (threshold of 200)’ contains the score of the highest predicted regulatory peak within the 30 Kb window around the gene's TSS. If this window does not contain any predicted peaks ≥ 200 (our cutoff threshold) no value is given (indicated by a dash). The next four columns of the table (‘Nr2e3-/-’ etc.) show the wild-type-to-mutant ratios of the averaged microarray scores for the given gene. For those genes to which more than one Affy tag correspond, the data for the first one listed under ‘Affy Mouse 430 2.0’ is given. Dark green = downregulated under high stringency (as described in METHODS); light green = downregulated under low stringency; red = upregulated under high stringency; orange = upregulated under low stringency. A dash indicates that the gene was not significantly a [file pone.0000643.s005.zip › Corbo_TableS1/Corbo_TableS1.html]

SortedTable


|  |  |  |  |  |  |  |  |  |  |  |  |  |  |  |  |  |  |  |  |  |  |
| --- | --- | --- | --- | --- | --- | --- | --- | --- | --- | --- | --- | --- | --- | --- | --- | --- | --- | --- | --- | --- | --- |
| Gene Symbol | Large Image Gene +/- 15kb | Closeup Image TSS +/- 15kb | Links | Max Score (threshold of 200) | *Nr2e3 -/-* | *Crx -/-* | *Nrl -/-* | *Crx;Nrl -/-* | In situ  hybridization | Electroporation | Full Name | Averaged Affy Scores | | | | | | | | Locus | Affy Mouse 430 2 |
|  |  |  |  |  |  |  |  |  |  |  |  | B6 | *Nr2e3 -/-* | 129 | *Crx -/-* | B6 | *Nrl -/-* | B6;129 | *Crx;Nrl -/-* |  |  |
| 0610011I04Rik | View | View |  | - | - | 0.30 | 0.60 | 0.54 |  |  | RIKEN CDNA 0610011I04 GENE | 474 | 509 | 416 | 1377 | 474 | 789 | 445 | 826 | chr6:48771095-48776634 | 1423909\_at |
| 0610037M15Rik | View | View |  | - | - | 0.08 | 0.29 | 0.33 |  |  | RIKEN CDNA 0610037M15 GENE | 109 | 265 | 134 | 1597 | 109 | 371 | 122 | 368 | chr17:35033315-35038106 | 1431008\_at |
| 0610038B21Rik | View | View |  | - | - | 8.09 | - | - |  |  | RIKEN CDNA 0610038B21 GENE | 233 | 218 | 275 | 34 | 233 | 105 | 254 | 44 | chr8:80413854-80414650 | 1430930\_at |
| 1110002B05Rik | View | View |  | - | 0.41 | - | 0.45 | 0.37 |  |  | RIKEN CDNA 1110002B05 GENE | 3353 | 8107 | 3568 | 4274 | 3353 | 7436 | 3461 | 9405 | chr12:55567027-55578155 | 1448388\_a\_at |
| 1110003E01Rik | View | View |  | 749 | - | 0.46 | 0.53 | 0.55 |  |  | RIKEN CDNA 1110003E01 GENE | 2043 | 2194 | 2507 | 5490 | 2043 | 3827 | 2275 | 4119 | chr5:65727699-65772073 | 1416768\_at, 1444100\_at |
| 1110020G09Rik | View | View |  | 245 | 2.37 | 3.96 | 2.24 | 3.33 |  |  | RIKEN CDNA 1110020G09 GENE | 6341 | 2679 | 5332 | 1347 | 6341 | 2832 | 5837 | 1753 | chr15:8998142-9037020 | 1437287\_at, 1453422\_a\_at |
| 1110051B16Rik | View | View |  | - | 3.25 | 2.37 | 9.70 | 5.78 |  |  | RIKEN CDNA 1110051B16 GENE | 1708 | 526 | 246 | 104 | 1708 | 176 | 977 | 169 | chr14:24816189-24822578 | 1445710\_x\_at, 1429099\_at |
| 1300007C21Rik | - | - |  | - | - | - | 0.33 | 0.07 |  |  | RIKEN CDNA 1300007C21 GENE | 836 | 752 | 8 | 20 | 836 | 2519 | 422 | 5881 | chr18 | 1431213\_a\_at |
| 1300010M03Rik | View | View |  | 253 | - | - | 0.22 | - |  |  | RIKEN CDNA 1300010M03 GENE | 367 | 289 | 1509 | 1503 | 367 | 1639 | 938 | 1650 | chr11:100912412-100935933 | 1424159\_at |
| 1500001H12Rik | View | View |  | 298 | - | - | 0.14 | - |  |  | RIKEN CDNA 1500001H12 GENE | 174 | 151 | 1110 | 1028 | 174 | 1271 | 642 | 1222 | chr7:141277769-141280740 | 1421348\_a\_at, 1421349\_x\_at |
| 1500016O10Rik | View | View |  | 236 | - | 2.18 | 2.31 | 1.94 |  |  | RIKEN CDNA 1500016O10 GENE | 20372 | 17294 | 15926 | 7304 | 20372 | 8827 | 18149 | 9376 | chr7:126588816-126621362 | 1438641\_x\_at, 1452807\_s\_at, 1454209\_at, 1452806\_at |
| 1700027L20Rik | View | View |  | - | - | 19.21 | - | - |  |  | RIKEN CDNA 1700027L20 GENE | 99 | 123 | 269 | 14 | 99 | 72 | 184 | 55 | chr1:89017836-89019574 | 1429647\_at |
| 1700056N10Rik | View | View |  | 430 | - | - | 0.16 | - |  |  | RIKEN CDNA 1700056N10 GENE | 19 | 19 | 93 | 86 | 19 | 122 | 56 | 109 | chr16:16961083-16962674 | 1432356\_at |
| 1810009A15Rik | View | View |  | 783 | - | 2.35 | 1.89 | 2.64 |  |  | RIKEN CDNA 1810009A15 GENE | 13339 | 13771 | 11847 | 5038 | 13339 | 7056 | 12593 | 4767 | chr19:8955948-8957785 | 1417886\_at, 1457083\_at |
| 1810041L15Rik | View | View |  | 227 | - | 0.34 | 0.40 | 0.46 |  |  | RIKEN CDNA 1810041L15 GENE | 2009 | 2223 | 2129 | 6342 | 2009 | 5030 | 2069 | 4479 | chr15:84207813-84274863 | 1455515\_at, 1429440\_at |
| 2310026E23Rik | View | View |  | 481 | - | - | 0.17 | 0.15 |  |  | RIKEN CDNA 2310026E23 GENE | 102 | 222 | 113 | 159 | 102 | 584 | 108 | 702 | chr4:110912938-110957857 | 1435351\_at |
| 2310030G06Rik | View | View |  | 743 | - | - | 0.18 | - |  |  | RIKEN CDNA 2310030G06 GENE | 27 | 45 | 100 | 115 | 27 | 153 | 64 | 174 | chr9:50491921-50498751 | 1449357\_at |
| 2310068G24Rik | View | View |  | - | - | - | 0.26 | - |  |  | RIKEN CDNA 2310068G24 GENE | 302 | 396 | 264 | 206 | 302 | 1155 | 283 | 386 | chr12:82162308-82163919 | 1433023\_at |
| 2510049J12Rik | View | View |  | - | - | 5.81 | - | 8.24 |  |  | RIKEN CDNA 2510049J12 GENE | 380 | 219 | 180 | 31 | 380 | 224 | 280 | 34 | chr6:115549164-115558095 | 1440916\_at |
| 2610034M16Rik | View | View |  | 881 | 1.45 | 20.97 | 10.19 | 30.01 |  |  | RIKEN CDNA 2610034M16 GENE | 4167 | 2883 | 3754 | 179 | 4167 | 409 | 3961 | 132 | chr17:58564413-58660601 | 1425408\_a\_at |
| 2610204L23Rik | View | View |  | 238 | - | - | 0.15 | - |  |  | RIKEN CDNA 2610204L23 GENE | 205 | 238 | 957 | 1055 | 205 | 1323 | 581 | 1119 | chr11:105979424-106032434 | 1424636\_at |
| 2610528A11Rik | View | View |  | 356 | 2.22 | - | 5.61 | - |  |  | RIKEN CDNA 2610528A11 GENE | 275 | 124 | 55 | 50 | 275 | 49 | 165 | 52 | chr14:36013533-36025762 | 1435639\_at |
| 2810407C02Rik | View | View |  | 2435 | - | - | 0.49 | - |  |  | RIKEN CDNA 2810407C02 GENE | 2714 | 2628 | 4691 | 5062 | 2714 | 5533 | 3703 | 5160 | chr3:58664666-58678220 | 1453726\_s\_at |
| 2900019G14Rik | View | View |  | - | - | - | 0.20 | - |  |  | RIKEN CDNA 2900019G14 GENE | 33 | 49 | 86 | 151 | 33 | 169 | 60 | 181 | chr2:102585167-102589606 | 1459014\_at |
| 2900046D03Rik | View | View |  | - | - | 7.17 | 2.58 | 4.76 |  |  | RIKEN CDNA 2900046D03 GENE | 1906 | 1425 | 1333 | 186 | 1906 | 740 | 1620 | 340 | chr7:126590914-126591029 | 1444230\_at |
| 2900057B20Rik | View | View |  | 1113 | - | - | 6.16 | - |  |  | RIKEN CDNA 2900057B20 GENE | 277 | 600 | 91 | 51 | 277 | 45 | 184 | 46 | chr18:76216922-76223836 | 1431631\_at |
| 2900097C17Rik | View | View |  | 270 | - | - | 0.07 | 0.21 |  |  | RIKEN CDNA 2900097C17 GENE | 144 | 114 | 420 | 430 | 144 | 1960 | 282 | 1319 | chr2:156083932-156084270 | 1432646\_a\_at |
| 3010001F23Rik | View | View |  | - | 3.95 | 5.28 | 12.15 | 8.35 |  |  | RIKEN CDNA 3010001F23 GENE | 1106 | 280 | 897 | 170 | 1106 | 91 | 1002 | 120 | chrX:147709288-147757414 | 1457010\_at |
| 3110001A13Rik | View | View |  | 498 | - | 3.04 | 0.36 | - |  |  | RIKEN CDNA 3110001A13 GENE | 502 | 297 | 1094 | 360 | 502 | 1382 | 798 | 528 | chr2:3630730-3699406 | 1416893\_at |
| 3632451O06Rik | View | View |  | 201 | - | 3.98 | - | - |  |  | RIKEN CDNA 3632451O06 GENE | 227 | 270 | 1043 | 262 | 227 | 257 | 635 | 233 | chr14:48604299-48696196 | 1450770\_at |
| 3732412D22Rik | View | View |  | - | - | - | 0.15 | - |  |  | RIKEN CDNA 3732412D22 GENE | 22 | 82 | 70 | 87 | 22 | 149 | 46 | 169 | chr5:67248202-67325938 | 1440776\_at |
| 4631423F02Rik | View | View |  | - | - | 0.12 | - | - |  |  | RIKEN CDNA 4631423F02 GENE | 39 | 24 | 94 | 757 | 39 | 32 | 67 | 236 | chr1:93181479-93192810 | 1452707\_at |
| 4631427C17Rik | View | View |  | 355 | - | - | 0.16 | 0.35 |  |  | RIKEN CDNA 4631427C17 GENE | 158 | 194 | 424 | 587 | 158 | 961 | 291 | 836 | chr6:29718543-29862319 | 1419730\_at |
| 4632412N22Rik | View | View |  | - | - | 0.03 | - | - |  |  | RIKEN CDNA 4632412N22 GENE | 9 | 12 | 9 | 261 | 9 | 15 | 9 | 13 | chr17:71590022-71634526 | 1440898\_at |
| 4732415M23Rik | View | View |  | - | - | - | 0.10 | - |  |  | RIKEN CDNA 4732415M23 GENE | 12 | 20 | 96 | 43 | 12 | 115 | 54 | 90 | chr8:126098974-126108134 | 1435365\_at |
| 4833428M15Rik | View | View |  | 2270 | - | - | 0.08 | - |  |  | RIKEN CDNA 4833428M15 GENE | 8 | 17 | 81 | 86 | 8 | 100 | 45 | 66 | chr14:96967846-96969866 | 1431471\_at |
| 4921509F24Rik | View | View |  | - | - | 10.30 | 6.14 | 26.20 |  |  | RIKEN CDNA 4921509F24 GENE | 215 | 170 | 309 | 30 | 215 | 35 | 262 | 10 | chr9:26567476-26610101 | 1453143\_at |
| 4921511K06Rik | View | View |  | 280 | 0.55 | 0.45 | 0.16 | 0.21 | Link |  | RIKEN CDNA 4921511K06 GENE | 1966 | 3563 | 2044 | 4537 | 1966 | 12675 | 2005 | 9656 | chr6:29349153-29376962 | 1424699\_at |
| 4930418G15Rik | View | View |  | 305 | - | 7.54 | 2.13 | 4.17 |  |  | RIKEN CDNA 4930418G15 GENE | 459 | 297 | 490 | 65 | 459 | 215 | 475 | 114 | chr1:9953846-9994350 | 1430283\_s\_at, 1424977\_at |
| 4930430E16Rik | View | View |  | 1473 | - | 3.68 | - | 1.93 |  |  | RIKEN CDNA 4930430E16 GENE | 1829 | 1864 | 2037 | 553 | 1829 | 1785 | 1933 | 1000 | chr11:22907531-22930788 | 1443569\_at, 1451653\_a\_at |
| 4930519N16Rik | View | View |  | 359 | - | 5.61 | - | 11.43 |  |  | RIKEN CDNA 4930519N16 GENE | 6736 | 6244 | 6660 | 1188 | 6736 | 5389 | 6698 | 586 | chr13:51183519-51187684 | 1429133\_at |
| 4930535I16Rik | View | View |  | 537 | - | - | 0.18 | - |  |  | RIKEN CDNA 4930535I16 GENE | 24 | 33 | 46 | 53 | 24 | 137 | 35 | 42 | chr4:123419807-123420325 | 1430608\_at |
| 4930554H23Rik | View | View |  | - | 0.09 | - | 0.12 | 0.24 |  |  | RIKEN CDNA 4930554H23 GENE | 82 | 880 | 85 | 64 | 82 | 681 | 84 | 357 | chr7:63223642-63323476 | 1454020\_at |
| 4932417H02Rik | View | View |  | - | - | - | 0.20 | - |  |  | RIKEN CDNA 4932417H02 GENE | 25 | 33 | 120 | 91 | 25 | 124 | 73 | 116 | chr11:119419788-119715680 | 1458068\_at |
| 4933409K07Rik | View | View |  | - | 0.09 | - | - | - |  |  | RIKEN CDNA 4933409K07 GENE | 22 | 243 | 85 | 48 | 22 | 46 | 54 | 59 | chr4:42479920-42484753 | 1447937\_a\_at |
| 4933437N03Rik | View | View |  | - | - | 0.16 | - | 0.04 |  |  | RIKEN CDNA 4933437N03 GENE | 78 | 122 | 53 | 338 | 78 | 117 | 66 | 1670 | chr4:62867719-62880377 | 1430207\_at |
| 4933439C20Rik | View | View |  | - | 5.23 | - | - | - |  |  | RIKEN CDNA 4933439C20 GENE | 947 | 181 | 899 | 742 | 947 | 1286 | 923 | 751 | chr11:3024018-3031950 | 1429452\_x\_at, 1453144\_at, 1453145\_at |
| 5430405N12Rik | View | View |  | 516 | 0.30 | - | - | - |  |  | RIKEN CDNA 5430405N12 GENE | 411 | 1384 | 34 | 32 | 411 | 351 | 223 | 233 | chr14:11032440-11034160 | 1429691\_at |
| 5430420E18Rik | View | View |  | 2077 | - | 5.59 | - | - |  |  | RIKEN CDNA 5430420E18 GENE | 12 | 22 | 151 | 27 | 12 | 16 | 82 | 8 | chr14:60173225-60175199 | 1429823\_at |
| 5830408C22Rik | View | View |  | - | 3.40 | - | 17.47 | - |  |  | RIKEN CDNA 5830408C22 GENE | 262 | 77 | 138 | 143 | 262 | 15 | 200 | 9 | chr8:26572543-26575531 | 1430159\_at |
| Gene Symbol | Large Image Gene +/- 15kb | Closeup Image TSS +/- 15kb | Links | Max Score (threshold of 200) | *Nr2e3 -/-* | *Crx -/-* | *Nrl -/-* | *Crx;Nrl -/-* | In situ  hybridization | Electroporation | Full Name | Averaged Affy Scores | | | | | | | | Locus | Affy Mouse 430 2 |
|  |  |  |  |  |  |  |  |  |  |  |  | B6 | *Nr2e3 -/-* | 129 | *Crx -/-* | B6 | *Nrl -/-* | B6;129 | *Crx;Nrl -/-* |  |  |
| 6030439D06Rik | View | View |  | 440 | - | 0.09 | - | - |  |  | RIKEN CDNA 6030439D06 GENE | 59 | 34 | 16 | 177 | 59 | 11 | 38 | 58 | chr8:118573701-118578294 | 1443078\_at |
| 6330407D12Rik | View | View |  | 299 | - | 2.61 | 3.37 | 3.46 | Link |  | RIKEN CDNA 6330407D12 GENE | 9089 | 9261 | 9493 | 3642 | 9089 | 2698 | 9291 | 2689 | chr6:49248939-49271167 | 1434313\_at |
| 6330442E10Rik | View | View |  | 1048 | - | 4.25 | 1.54 | - |  |  | RIKEN CDNA 6330442E10 GENE | 1743 | 1483 | 3357 | 789 | 1743 | 1130 | 2550 | 1326 | chr12:79880637-79926177 | 1457254\_x\_at, 1454632\_at |
| 6330442E10Rik | View | View |  | 1048 | - | 4.03 | 1.95 | 2.05 |  |  | RIKEN CDNA 6330442E10 GENE | 2677 | 1995 | 4047 | 1004 | 2677 | 1373 | 3362 | 1640 | chr12:79880637-79926177 | 1454632\_at |
| 6430548M08Rik | View | View |  | - | 1.41 | 3.16 | 1.85 | 2.39 |  |  | RIKEN CDNA 6430548M08 GENE | 2036 | 1448 | 2256 | 715 | 2036 | 1101 | 2146 | 897 | chr8:123000143-123051275 | 1425637\_at |
| 6430562O15Rik | View | View |  | - | 2.90 | - | 5.55 | - |  |  | RIKEN CDNA 6430562O15 GENE | 322 | 111 | 53 | 58 | 322 | 58 | 188 | 37 | chr13:100508767-100513033 | 1456982\_at |
| 6620401M08Rik | View | View |  | 436 | 0.33 | 0.66 | 0.28 | 0.36 |  |  | RIKEN CDNA 6620401M08 GENE | 1424 | 4298 | 1506 | 2299 | 1424 | 5086 | 1465 | 4099 | chr7:51865572-51874036 | 1429146\_at, 1453049\_at |
| 7530404M11Rik | View | View |  | - | 0.62 | 0.42 | 0.18 | 0.24 | Link |  | RIKEN CDNA 7530404M11 GENE | 1703 | 2764 | 2043 | 4892 | 1703 | 9234 | 1873 | 7875 | chr3:104900227-104901988 | 1442160\_at |
| 9030218A15Rik | View | View |  | 1517 | - | - | 0.16 | - |  |  | RIKEN CDNA 9030218A15 GENE | 39 | 38 | 145 | 134 | 39 | 247 | 92 | 108 | chr3:86448414-86449506 | 1432790\_at |
| 9030407P20Rik | View | View |  | - | - | - | 0.07 | - |  |  | RIKEN CDNA 9030407P20 GENE | 12 | 23 | 105 | 151 | 12 | 162 | 59 | 182 | chr7:120464588-120468669 | 1454056\_at |
| 9130230N09Rik | View | View |  | 625 | - | 0.17 | - | - |  |  | RIKEN CDNA 9130230N09 GENE | 36 | 22 | 28 | 168 | 36 | 85 | 32 | 55 | chr11:22760361-22761256 | 1437980\_at |
| 9330168M11Rik | View | View |  | - | 2.27 | 167.32 | 96.90 | 325.09 |  |  | RIKEN CDNA 9330168M11 GENE | 3973 | 1751 | 3179 | 19 | 3973 | 41 | 3576 | 11 | chrX:145803307-145806370 | 1458418\_at |
| 9930021J17Rik | View | View |  | 782 | - | 5.26 | 12.52 | 10.20 |  |  | RIKEN CDNA 9930021J17 GENE | 526 | 241 | 473 | 90 | 526 | 42 | 500 | 49 | chr3:85745993-85816368 | 1437371\_at |
| A230097K15Rik | View | View |  | - | 0.23 | - | 0.13 | 0.14 |  |  | RIKEN CDNA A230097K15 GENE | 158 | 681 | 179 | 266 | 158 | 1227 | 169 | 1237 | chr5:101086598-101139403 | 1454799\_at |
| A2m | View | View |  | 2026 | 0.45 | 0.28 | 0.65 | 2.61 |  |  | ALPHA-2-MACROGLOBULIN | 478 | 1055 | 410 | 1475 | 478 | 737 | 444 | 170 | chr6:121601908-121644181 | 1434719\_at |
| A330094K24Rik | View | View |  | - | 1.55 | 9.28 | 3.61 | 9.01 |  |  | RIKEN CDNA A330094K24 GENE | 1266 | 819 | 1039 | 112 | 1266 | 351 | 1153 | 128 | chr18:77933759-77937546 | 1460479\_at |
| A430060F13Rik | View | View |  | - | - | - | 0.09 | - |  |  | RIKEN CDNA A430060F13 GENE | 14 | 30 | 42 | 67 | 14 | 153 | 28 | 76 | chr11:94573144-94577001 | 1437361\_at |
| A730062M13Rik | View | View |  | 667 | - | - | 0.19 | - |  |  | RIKEN CDNA A730062M13 GENE | 82 | 102 | 89 | 221 | 82 | 443 | 86 | 263 | chr9:72666784-72668693 | 1446810\_at |
| A830021M18 | - | - |  | - | - | - | 0.16 | 0.24 |  |  | HYPOTHETICAL PROTEIN A830021M18 | 160 | 304 | 47 | 141 | 160 | 1000 | 104 | 427 | chr15 | 1445721\_at |
| A930004D18Rik | - | - |  | - | 2.13 | 51.61 | 2.56 | 4.64 |  |  | RIKEN CDNA A930004D18 GENE | 1295 | 608 | 1445 | 28 | 1295 | 506 | 1370 | 295 | chr2 | 1429791\_at, 1440683\_at |
| A930009A15Rik | View | View |  | - | 0.11 | 0.07 | 0.07 | 0.06 |  |  | RIKEN CDNA A930009A15 GENE | 96 | 844 | 209 | 3124 | 96 | 1322 | 153 | 2408 | chr10:114974096-114986558 | 1453398\_at |
| A930015D22Rik | View | View |  | 1219 | - | 6.18 | - | 3.18 |  |  | RIKEN CDNA A930015D22 GENE | 1328 | 1482 | 1507 | 244 | 1328 | 1591 | 1418 | 446 | chr2:121142621-121148222 | 1426035\_at |
| A930018M24Rik | View | View |  | - | - | 0.30 | 0.05 | 0.09 | Link |  | HYPOTHETICAL PROTEIN 9330167E06 | 136 | 88 | 286 | 960 | 136 | 2495 | 211 | 2223 | chr14:49816905-49817486 | 1439143\_at |
| A930028C08Rik | View | View |  | - | 2.77 | - | 114.00 | - |  |  | RIKEN CDNA A930028C08 GENE | 1026 | 370 | 11 | 5 | 1026 | 9 | 519 | 8 | chr4:148191714-148203128 | 1430945\_at |
| A930036K24Rik | View | View |  | 449 | 1.65 | 70.03 | 20.28 | 51.09 |  |  | RIKEN CDNA A930036K24 GENE | 2657 | 1606 | 8684 | 124 | 2657 | 131 | 5671 | 111 | chr9:107538207-107539380 | 1431174\_at |
| Aadacl1 | View | View |  | 809 | - | - | 0.20 | - |  |  | ARYLACETAMIDE DEACETYLASE-LIKE 1 | 92 | 118 | 502 | 504 | 92 | 465 | 297 | 468 | chr3:27374073-27475525 | 1435135\_at |
| Abca13 | View | View |  | - | - | 10.82 | 9.47 | 12.27 |  |  | ATP-BINDING CASSETTE, SUB-FAMILY A (ABC1), MEMBER 13 | 625 | 650 | 552 | 51 | 625 | 66 | 589 | 48 | chr11:9091969-9583635 | 1446715\_at, 1440370\_at |
| Abca4 | View | View |  | 350 | - | 4.34 | 1.88 | - | Link |  | ATP-BINDING CASSETTE, SUB-FAMILY A (ABC1), MEMBER 4 | 10378 | 8800 | 4388 | 1011 | 10378 | 5527 | 7383 | 2445 | chr3:122036482-122172083 | 1449588\_at |
| Abi3 | View | View |  | 206 | - | 0.39 | 0.10 | 0.06 |  |  | ABI GENE FAMILY, MEMBER 3 | 38 | 185 | 85 | 219 | 38 | 391 | 62 | 1014 | chr11:95646162-95658250 | 1452928\_at |
| Acbd6 | View | View |  | 2732 | 0.54 | 0.35 | 0.21 | 0.31 |  |  | ACYL-COENZYME A BINDING DOMAIN CONTAINING 6 | 641 | 1187 | 742 | 2146 | 641 | 3089 | 692 | 2261 | chr1:157320337-157449450 | 1452601\_a\_at, 1453199\_at |
| Accn4 | View | View |  | 1792 | - | 0.32 | 0.06 | 0.15 |  |  | AMILORIDE-SENSITIVE CATION CHANNEL 4, PITUITARY | 125 | 113 | 566 | 1780 | 125 | 2058 | 346 | 2298 | chr1:75333847-75357488 | 1437091\_at |
| Acoxl | View | View |  | - | - | - | 13.55 | - |  |  | ACYL-COENZYME A OXIDASE-LIKE | 149 | 78 | 30 | 10 | 149 | 11 | 90 | 12 | chr2:127546069-127815314 | 1460470\_at |
| Actb | View | View |  | - | - | - | 0.29 | - |  |  | ACTIN, BETA, CYTOPLASMIC | 1387 | 1612 | 4491 | 4187 | 1387 | 4834 | 2939 | 4694 | chr5:143168256-143171864 | 1419734\_at |
| Acvr2b | View | View |  | - | - | - | 3.20 | - |  |  | ACTIVIN RECEPTOR IIB | 1131 | 912 | 528 | 353 | 1131 | 353 | 830 | 332 | chr9:119251215-119282213 | 1439856\_at |
| Adcy1 | View | View |  | 441 | 1.52 | 1.65 | 3.85 | 2.98 |  |  | ADENYLATE CYCLASE 1 | 7016 | 4628 | 3998 | 2428 | 7016 | 1820 | 5507 | 1848 | chr11:6963492-7078509 | 1456487\_at |
| Adipor1 | View | View |  | - | - | 3.46 | 2.62 | 3.01 |  |  | ADIPONECTIN RECEPTOR 1 | 10985 | 10322 | 10259 | 2969 | 10985 | 4198 | 10622 | 3533 | chr1:136231891-136248748 | 1439017\_x\_at, 1424312\_at, 1451311\_a\_at |
| Adrb1 | View | View |  | 1023 | 0.28 | 0.57 | 0.15 | 0.19 | Link |  | ADRENERGIC RECEPTOR, BETA 1 | 1385 | 5018 | 1561 | 2743 | 1385 | 9023 | 1473 | 7919 | chr19:56775622-56777022 | 1423420\_at |
| Adrb2 | View | View |  | 312 | - | - | 0.11 | 0.02 |  |  | ADRENERGIC RECEPTOR, BETA 2 | 31 | 22 | 5 | 19 | 31 | 270 | 18 | 783 | chr18:62303865-62305121 | 1437302\_at |
| Agpat3 | View | View |  | - | - | 2.51 | 1.65 | 1.92 |  |  | 1-ACYLGLYCEROL-3-PHOSPHATE O-ACYLTRANSFERASE 3 | 6827 | 6210 | 7234 | 2887 | 6827 | 4134 | 7031 | 3670 | chr10:77674692-77754829 | 1433819\_s\_at |
| Agr2 | View | View |  | - | - | 5.08 | - | - |  |  | ANTERIOR GRADIENT 2 (XENOPUS LAEVIS) | 36 | 90 | 132 | 26 | 36 | 20 | 84 | 26 | chr12:36503173-36514346 | 1419268\_at |
| AI847670 | View | View |  | 265 | 0.22 | - | - | - |  |  | EXPRESSED SEQUENCE AI847670 | 385 | 1756 | 411 | 411 | 385 | 498 | 398 | 423 | chr5:20936992-20993948 | 1434266\_at |
| AI852064 | - | - |  | - | - | 3.95 | 4.39 | 6.00 |  |  | EXPRESSED SEQUENCE AI852064 | 9552 | 9923 | 6366 | 1610 | 9552 | 2178 | 7959 | 1327 | chr2 | 1440838\_at |
| Aipl1 | View | View |  | - | - | - | 0.28 | - | Link |  | ARYL HYDROCARBON RECEPTOR-INTERACTING PROTEIN-LIKE 1 | 4572 | 3537 | 7361 | 8565 | 4572 | 16164 | 5967 | 7449 | chr11:71845360-71853653 | 1425590\_s\_at |
| Ak3l1 | View | View |  | 775 | - | - | 0.17 | - |  |  | ADENYLATE KINASE 3 ALPHA-LIKE 1 | 25 | 67 | 163 | 136 | 25 | 144 | 94 | 104 | chr2:72434775-72435446 | 1421829\_at |
| Akp2 | View | View |  | - | 1.24 | 5.90 | 3.94 | 14.82 |  |  | ALKALINE PHOSPHATASE 2, LIVER | 2794 | 2259 | 1741 | 295 | 2794 | 710 | 2268 | 153 | chr4:137013809-137068395 | 1423611\_at |
| Aldh2 | View | View |  | 249 | - | - | 0.20 | - |  |  | ALDEHYDE DEHYDROGENASE 2, MITOCHONDRIAL | 122 | 117 | 598 | 623 | 122 | 622 | 360 | 536 | chr5:121828319-121854203 | 1448143\_at |
| Amigo1 | View | View |  | 1765 | - | - | 0.09 | - |  |  | ADHESION MOLECULE WITH IG LIKE DOMAIN 1 | 20 | 116 | 124 | 168 | 20 | 220 | 72 | 239 | chr3:108314391-108319832 | 1425851\_a\_at |
| Ampd2 | View | View |  | 256 | 0.67 | 0.50 | 0.28 | 0.35 | Link |  | ADENOSINE MONOPHOSPHATE DEAMINASE 2 (ISOFORM L) | 865 | 1295 | 707 | 1428 | 865 | 3056 | 786 | 2276 | chr3:108202971-108209197 | 1426757\_at, 1438941\_x\_at |
| Ankrd11 | View | View |  | - | - | - | 0.16 | - |  |  | ANKYRIN REPEAT DOMAIN 11 | 165 | 242 | 762 | 748 | 165 | 1043 | 464 | 904 | chr8:125770244-125786243 | 1437633\_at |
| Ankrd33 | View | View |  | 448 | - | 14.41 | - | - |  |  | ANKYRIN REPEAT DOMAIN 33 | 5830 | 7952 | 4019 | 279 | 5830 | 8037 | 4925 | 2894 | chr15:100943789-100948062 | 1451636\_at |
| Gene Symbol | Large Image Gene +/- 15kb | Closeup Image TSS +/- 15kb | Links | Max Score (threshold of 200) | *Nr2e3 -/-* | *Crx -/-* | *Nrl -/-* | *Crx;Nrl -/-* | In situ  hybridization | Electroporation | Full Name | Averaged Affy Scores | | | | | | | | Locus | Affy Mouse 430 2 |
|  |  |  |  |  |  |  |  |  |  |  |  | B6 | *Nr2e3 -/-* | 129 | *Crx -/-* | B6 | *Nrl -/-* | B6;129 | *Crx;Nrl -/-* |  |  |
| Anxa2 | View | View |  | 287 | - | 0.33 | - | - |  |  | ANNEXIN A2 | 607 | 627 | 515 | 1582 | 607 | 595 | 561 | 462 | chr9:69252847-69290959 | 1419091\_a\_at |
| Aof1 | View | View |  | 247 | 0.12 | - | - | - |  |  | AMINE OXIDASE, FLAVIN CONTAINING 1 | 487 | 4131 | 568 | 528 | 487 | 589 | 528 | 459 | chr13:47054541-47095585 | 1433649\_at |
| Ap2a2 | View | View |  | - | 1.30 | 1.36 | 2.36 | 2.17 |  |  | ADAPTOR PROTEIN COMPLEX AP-2, ALPHA 2 SUBUNIT | 5871 | 4508 | 4568 | 3366 | 5871 | 2488 | 5220 | 2405 | chr7:141413558-141484333 | 1435869\_s\_at, 1455482\_at |
| Ap2b1 | View | View |  | 465 | - | - | 0.21 | - |  |  | ADAPTOR-RELATED PROTEIN COMPLEX 2, BETA 1 SUBUNIT | 468 | 591 | 1947 | 1530 | 468 | 2280 | 1208 | 1911 | chr11:82933040-83221234 | 1452292\_at |
| Aplp2 | View | View |  | 896 | - | - | 0.32 | - |  |  | AMYLOID BETA (A4) PRECURSOR-LIKE PROTEIN 2 | 2264 | 1811 | 6545 | 5295 | 2264 | 7164 | 4405 | 6008 | chr9:30899122-30961358 | 1421889\_a\_at |
| Apobec2 | View | View |  | - | 6.14 | 0.12 | 1.83 | - |  |  | APOLIPOPROTEIN B EDITING COMPLEX 2 | 706 | 115 | 161 | 1315 | 706 | 385 | 434 | 872 | chr17:47884798-47898295 | 1417889\_at |
| Aqp1 | View | View |  | 240 | 16.32 | 42.55 | 15.49 | 49.32 |  |  | AQUAPORIN 1 | 1828 | 112 | 2808 | 66 | 1828 | 118 | 2318 | 47 | chr6:55266010-55278133 | 1416203\_at |
| Arg2 | View | View |  | - | - | 23.81 | 2.59 | 10.32 |  |  | ARGINASE TYPE II | 733 | 623 | 857 | 36 | 733 | 283 | 795 | 77 | chr12:80049664-80075138 | 1418847\_at, 1438841\_s\_at |
| Arhgap10 | View | View |  | - | - | 5.12 | - | - |  |  | RHO GTPASE ACTIVATING PROTEIN 10 | 60 | 67 | 128 | 25 | 60 | 81 | 94 | 41 | chr8:80146991-80413860 | 1459609\_at, 1426027\_a\_at |
| Arhgdib | View | View |  | 291 | 0.12 | - | 0.20 | - |  |  | RHO, GDP DISSOCIATION INHIBITOR (GDI) BETA | 321 | 2763 | 128 | 182 | 321 | 1640 | 225 | 421 | chr6:136887905-136905913 | 1426454\_at |
| Arl13b | View | View |  | 287 | 1.62 | 2.45 | 5.41 | 4.27 |  |  | ADP-RIBOSYLATION FACTOR-LIKE 13B | 1142 | 703 | 908 | 370 | 1142 | 211 | 1025 | 240 | chr16:62735941-62789253 | 1437021\_at |
| Arl2bp | View | View |  | 351 | - | - | 0.11 | - |  |  | ADP-RIBOSYLATION FACTOR-LIKE 2 BINDING PROTEIN | 291 | 327 | 2086 | 2250 | 291 | 2674 | 1189 | 2332 | chr8:97555780-97563546 | 1429859\_a\_at |
| Arpc3 | View | View |  | 263 | - | - | 0.15 | - |  |  | ACTIN RELATED PROTEIN 2/3 COMPLEX, SUBUNIT 3 | 386 | 379 | 2066 | 2379 | 386 | 2624 | 1226 | 2631 | chr5:122657428-122666607 | 1448279\_at |
| Arr3 | View | View |  | 266 | 0.59 | 2.02 | 0.22 | - | Link |  | ARRESTIN 3, RETINAL | 4257 | 7245 | 4313 | 2140 | 4257 | 19723 | 4285 | 5975 | chrX:96808275-96821106 | 1450329\_a\_at, 1425232\_x\_at |
| Atp1a1 | View | View |  | 455 | - | - | 2.97 | - |  |  | ATPASE, NA+/K+ TRANSPORTING, ALPHA 1 POLYPEPTIDE | 5107 | 4775 | 3724 | 4313 | 5107 | 1717 | 4416 | 2566 | chr3:101705286-101733745 | 1423653\_at, 1451071\_a\_at |
| Atp1b2 | View | View |  | 286 | - | 0.67 | 0.46 | 0.56 |  |  | ATPASE, NA+/K+ TRANSPORTING, BETA 2 POLYPEPTIDE | 3952 | 3683 | 5863 | 8766 | 3952 | 8568 | 4908 | 8835 | chr11:69415937-69422137 | 1422009\_at |
| Atp6ap1 | View | View |  | - | - | - | 0.47 | - |  |  | ATPASE, H+ TRANSPORTING, LYSOSOMAL (VACUOLAR PROTON PUMP), SUBUNIT 1 | 3582 | 3325 | 6146 | 7165 | 3582 | 7599 | 4864 | 7478 | chrX:70549826-70557410 | 1449622\_s\_at |
| Atp6v0a1 | View | View |  | 640 | - | - | 0.17 | - |  |  | ATPASE, H+ TRANSPORTING, LYSOSOMAL V0 SUBUNIT A1 | 294 | 206 | 857 | 1087 | 294 | 1775 | 576 | 1168 | chr11:100825545-100879804 | 1425227\_a\_at |
| AW547186 | View | View |  | 861 | 0.13 | - | - | - |  |  | EXPRESSED SEQUENCE AW547186 | 216 | 1716 | 371 | 387 | 216 | 310 | 294 | 306 | chrX:137927784-138089716 | 1433854\_at, 1454741\_s\_at |
| Azin1 | View | View |  | 261 | - | - | 0.15 | - |  |  | ANTIZYME INHIBITOR 1 | 134 | 128 | 729 | 711 | 134 | 919 | 432 | 961 | chr15:38432019-38463855 | 1430984\_at |
| B230343A10Rik | View | View |  | - | - | 3.10 | - | - |  |  | RIKEN CDNA B230343A10 GENE | 311 | 295 | 1301 | 420 | 311 | 355 | 806 | 317 | chr11:67503316-67505125 | 1442019\_at |
| B3gnt2 | View | View |  | 625 | - | 0.25 | 0.42 | - |  |  | UDP-GLCNAC:BETAGAL BETA-1,3-N-ACETYLGLUCOSAMINYLTRANSFERASE 1 | 163 | 141 | 314 | 1256 | 163 | 389 | 239 | 327 | chr11:22734734-22760336 | 1420852\_a\_at, 1450026\_a\_at |
| B4galt2 | View | View |  | 471 | 1.97 | 13.75 | 2.25 | 10.76 |  |  | COILED-COIL DOMAIN CONTAINING 24 | 1730 | 880 | 2227 | 162 | 1730 | 769 | 1979 | 184 | chr4:117367196-117381401 | 1456905\_at |
| Bach2 | View | View |  | 2021 | 0.26 | 0.30 | 0.40 | 0.33 |  |  | BTB AND CNC HOMOLOGY 2 | 262 | 1010 | 246 | 821 | 262 | 653 | 254 | 778 | chr4:32830238-32909894 | 1437667\_a\_at, 1441657\_at |
| Bax | View | View |  | - | - | 0.70 | 0.32 | - |  |  | BCL2-ASSOCIATED X PROTEIN | 374 | 355 | 931 | 1337 | 374 | 1159 | 653 | 1150 | chr7:45329742-45334871 | 1416837\_at |
| Bbs2 | View | View |  | 742 | - | - | 1.91 | 1.88 |  | Link | BARDET-BIEDL SYNDROME 2 HOMOLOG (HUMAN) | 1797 | 1301 | 1391 | 927 | 1797 | 941 | 1594 | 850 | chr8:96957083-96987940 | 1424478\_at |
| Bbs5 | View | View |  | 644 | - | 2.90 | - | 2.20 |  | Link | BARDET-BIEDL SYNDROME 5 (HUMAN) | 2866 | 2168 | 3735 | 1290 | 2866 | 2265 | 3301 | 1502 | chr2:69448094-69468408 | 1429762\_a\_at |
| Bbs7 | View | View |  | 772 | 1.46 | - | 3.23 | - |  |  | BARDET-BIEDL SYNDROME 7 | 3702 | 2531 | 1643 | 2007 | 3702 | 1147 | 2673 | 1329 | chr3:36764695-36804897 | 1454684\_at |
| BC038479 | View | View |  | - | 1.49 | 1.80 | 8.61 | 14.30 |  |  | CDNA SEQUENCE BC038479 | 2212 | 1487 | 2794 | 1551 | 2212 | 257 | 2503 | 175 | chr9:26512490-26555867 | 1433728\_at, 1433727\_at |
| BC060957 | View | View |  | - | - | 7.62 | - | - |  |  | CDNA SEQUENCE BC060957 | 199 | 238 | 221 | 29 | 199 | 140 | 210 | 101 | chr8:98423229-98448017 | 1436285\_at |
| BC067067 | View | View |  | - | - | 0.03 | - | 0.13 |  |  | CDNA SEQUENCE BC067067 | 39 | 64 | 40 | 1479 | 39 | 63 | 40 | 310 | chr15:101621342-101630366 | 1436557\_at |
| Bcl2l2 | View | View |  | 290 | - | - | 0.17 | - |  |  | BCL2-LIKE 2 | 18 | 48 | 77 | 92 | 18 | 109 | 48 | 113 | chr14:53837600-53842435 | 1430454\_x\_at |
| Bcl3 | View | View |  | 1145 | - | 0.13 | - | - |  |  | B-CELL LEUKEMIA/LYMPHOMA 3 | 70 | 101 | 14 | 108 | 70 | 8 | 42 | 23 | chr7:18966984-18981237 | 1418133\_at |
| Birc4 | View | View |  | 246 | - | 2.25 | - | - |  |  | BACULOVIRAL IAP REPEAT-CONTAINING 4 | 10381 | 11821 | 10853 | 4831 | 10381 | 9482 | 10617 | 7392 | chrX:38315952-38350134 | 1437533\_at |
| Bpnt1 | View | View |  | - | - | - | 0.32 | 0.48 |  |  | BISPHOSPHATE 3'-NUCLEOTIDASE 1 | 476 | 455 | 712 | 870 | 476 | 1491 | 594 | 1227 | chr1:187032947-187058557 | 1418764\_a\_at |
| Brcc3 | View | View |  | 287 | - | - | 0.05 | - |  |  | BRCA1/BRCA2-CONTAINING COMPLEX, SUBUNIT 3 | 9 | 38 | 233 | 199 | 9 | 193 | 121 | 228 | chrX:71669439-71706720 | 1426605\_at |
| Bspry | View | View |  | 608 | - | - | 0.08 | 0.14 |  |  | B-BOX AND SPRY DOMAIN CONTAINING | 46 | 163 | 53 | 46 | 46 | 543 | 50 | 355 | chr4:61966428-61983659 | 1450131\_a\_at |
| Btg2 | View | View |  | - | - | 0.49 | 0.49 | 0.30 |  |  | B-CELL TRANSLOCATION GENE 2, ANTI-PROLIFERATIVE | 2896 | 3068 | 2854 | 5768 | 2896 | 5942 | 2875 | 9501 | chr1:135891276-135895566 | 1416250\_at |
| Bub1b | View | View |  | 590 | 0.17 | - | 0.50 | - |  |  | BUDDING UNINHIBITED BY BENZIMIDAZOLES 1 HOMOLOG, BETA (S. CEREVISIAE) | 819 | 4852 | 134 | 11 | 819 | 1634 | 477 | 53 | chr2:118289708-118333032 | 1416961\_at, 1447363\_s\_at |
| Bzw1 | View | View |  | - | - | - | 0.24 | - |  |  | BASIC LEUCINE ZIPPER AND W2 DOMAINS 1 | 441 | 374 | 1876 | 2383 | 441 | 1815 | 1159 | 2146 | chr1:58337673-58351098 | 1450846\_at |
| C130076O07Rik | View | View |  | 375 | - | - | 0.17 | 0.38 |  |  | RIKEN CDNA C030017F07 GENE | 74 | 73 | 123 | 212 | 74 | 446 | 99 | 260 | chr12:45199270-45469135 | 1442587\_at, 1434709\_at |
| C1ql3 | View | View |  | 1447 | - | 0.46 | 0.75 | 0.42 |  |  | C1Q-LIKE 3 | 2381 | 2223 | 2905 | 6334 | 2381 | 3171 | 2643 | 6234 | chr2:12921293-12929600 | 1451620\_at |
| C230014O12Rik | View | View |  | 1903 | - | - | 0.16 | - |  |  | RIKEN CDNA C230014O12 GENE | 22 | 42 | 51 | 10 | 22 | 137 | 37 | 66 | chr2:33840298-33929738 | 1446877\_at |
| C4b | View | View |  | - | - | 0.22 | - | - |  |  | COMPLEMENT COMPONENT 4B (CHILDO BLOOD GROUP) | 622 | 763 | 230 | 1052 | 622 | 619 | 426 | 212 | chr17:34336443-34351939 | 1418021\_at |
| C530024P05Rik | View | View |  | 245 | - | - | 0.32 | - |  |  | RIKEN CDNA 4933413A10 GENE | 646 | 707 | 651 | 698 | 646 | 2041 | 649 | 858 | chr5:113731055-113850662 | 1435796\_at |
| C79127 | View | View |  | 253 | - | 14.92 | - | 11.05 |  |  | EXPRESSED SEQUENCE C79127 | 1507 | 1572 | 746 | 50 | 1507 | 810 | 1127 | 102 | chr7:18435328-18438571 | 1442242\_at |
| C81521 | - | - |  | - | - | - | - | - |  |  | EXPRESSED SEQUENCE C81521 |  |  |  |  |  |  |  |  | - | 1441013\_at |
| Cabp4 | View | View |  | 218 | - | 7.75 | - | 13.00 |  | Link | CALCIUM BINDING PROTEIN 4 | 2606 | 2212 | 3216 | 415 | 2606 | 2168 | 2911 | 224 | chr19:4135423-4139609 | 1425878\_at, 1456936\_at |
| Cabp5 | View | View |  | 446 | - | - | 0.29 | - | Link |  | CALCIUM BINDING PROTEIN 5 | 3163 | 3297 | 5555 | 4428 | 3163 | 10768 | 4359 | 6306 | chr7:12298311-12309059 | 1451826\_at, 1421780\_a\_at |
| Cacna1f | View | View |  | 995 | - | 1.64 | - | 1.84 |  | Link | CALCIUM CHANNEL, VOLTAGE-DEPENDENT, ALPHA 1F SUBUNIT | 3307 | 3669 | 2219 | 1351 | 3307 | 2689 | 2763 | 1499 | chrX:6764079-6792152 | 1449955\_at |
| Gene Symbol | Large Image Gene +/- 15kb | Closeup Image TSS +/- 15kb | Links | Max Score (threshold of 200) | *Nr2e3 -/-* | *Crx -/-* | *Nrl -/-* | *Crx;Nrl -/-* | In situ  hybridization | Electroporation | Full Name | Averaged Affy Scores | | | | | | | | Locus | Affy Mouse 430 2 |
|  |  |  |  |  |  |  |  |  |  |  |  | B6 | *Nr2e3 -/-* | 129 | *Crx -/-* | B6 | *Nrl -/-* | B6;129 | *Crx;Nrl -/-* |  |  |
| Cacna2d4 | View | View |  | - | - | - | 0.47 | - |  |  | CALCIUM CHANNEL, VOLTAGE-DEPENDENT, ALPHA 2/DELTA SUBUNIT 4 | 767 | 687 | 1211 | 1656 | 767 | 1642 | 989 | 1377 | chr6:119202145-119316175 | 1442863\_at |
| Cadps2 | View | View |  | 596 | - | 0.14 | - | - |  |  | CEREBELLUM POSTNATAL DEVELOPMENT ASSOCIATED PROTEIN 2 | 294 | 323 | 329 | 2275 | 294 | 368 | 312 | 379 | chr6:23212799-23789139 | 1451499\_at |
| Calu | View | View |  | 1040 | - | 0.72 | 3.14 | 0.66 |  |  | CALUMENIN | 2113 | 2027 | 1830 | 2542 | 2113 | 674 | 1972 | 2977 | chr6:29298191-29326677 | 1415870\_at |
| Camk2b | View | View |  | 390 | - | - | 0.16 | 0.03 |  |  | CALCIUM/CALMODULIN-DEPENDENT PROTEIN KINASE II, BETA | 338 | 450 | 59 | 31 | 338 | 2128 | 199 | 7826 | chr11:5869675-5965751 | 1455869\_at, 1448676\_at |
| Camsap1l1 | View | View |  | 248 | - | - | 0.19 | - |  |  | CALMODULIN REGULATED SPECTRIN-ASSOCIATED PROTEIN 1-LIKE 1 | 30 | 34 | 111 | 179 | 30 | 162 | 71 | 121 | chr1:138084664-138162515 | 1459385\_at |
| Casp7 | View | View |  | - | - | 2.07 | 0.11 | 0.36 | Link |  | CASPASE 7 | 498 | 658 | 775 | 375 | 498 | 4684 | 637 | 1748 | chr19:56457666-56494345 | 1426062\_a\_at, 1448659\_at |
| Cbx7 | View | View |  | - | - | 0.13 | 0.56 | - |  |  | CHROMOBOX HOMOLOG 7 | 197 | 100 | 14 | 104 | 197 | 349 | 106 | 154 | chr15:79743065-79759894 | 1420039\_s\_at |
| Ccdc64 | View | View |  | 879 | - | - | 3.18 | 4.48 |  |  | COILED-COIL DOMAIN CONTAINING 64 | 2391 | 2411 | 1772 | 802 | 2391 | 751 | 2082 | 465 | chr5:115909177-115992551 | 1426816\_at |
| Cckbr | View | View |  | 486 | 0.15 | 0.19 | 0.03 | 0.06 |  |  | CHOLECYSTOKININ B RECEPTOR | 70 | 452 | 109 | 576 | 70 | 2375 | 90 | 1451 | chr7:105299638-105344719 | 1460663\_at, 1454770\_at |
| Ccl21c | View | View |  | - | 0.04 | - | - | - |  |  | CHEMOKINE (C-C MOTIF) LIGAND 21B | 9 | 204 | 7 | 6 | 9 | 8 | 8 | 6 | chr4:42015844-42016974 | 1419426\_s\_at |
| Ccndbp1 | View | View |  | 1279 | - | 2.03 | - | - |  |  | CYCLIN D-TYPE BINDING-PROTEIN 1 | 5235 | 5212 | 7248 | 3572 | 5235 | 5433 | 6242 | 4374 | chr2:120699913-120708332 | 1420745\_a\_at |
| Ccnt2 | View | View |  | 486 | 1.15 | - | 0.31 | - |  |  | CYCLIN T2 | 539 | 468 | 1542 | 1988 | 539 | 1753 | 1041 | 1786 | chr1:129601763-129631105 | 1453399\_at |
| Cd47 | View | View |  | 346 | - | 0.47 | - | - |  |  | CD47 ANTIGEN (RH-RELATED ANTIGEN, INTEGRIN-ASSOCIATED SIGNAL TRANSDUCER) | 2118 | 2408 | 2679 | 5675 | 2118 | 2285 | 2399 | 2841 | chr16:49775413-49826444 | 1449507\_a\_at |
| Cd8a | View | View |  | 238 | 0.15 | - | - | - |  |  | CD8 ANTIGEN, ALPHA CHAIN | 79 | 519 | 44 | 52 | 79 | 78 | 62 | 49 | chr6:71303062-71307116 | 1444078\_at, 1425335\_at |
| Cdh23 | View | View |  | 988 | - | - | - | - |  |  | CADHERIN 23 (OTOCADHERIN) | 27 | 40 | 9 | 29 | 27 | 21 | 18 | 7 | chr10:59698656-60052690 | 1452028\_a\_at |
| Cdr2 | View | View |  | 325 | - | 5.62 | 6.44 | 37.81 |  |  | CEREBELLAR DEGENERATION-RELATED 2 | 5429 | 4433 | 5837 | 1038 | 5429 | 843 | 5633 | 149 | chr7:120748185-120773433 | 1417430\_at |
| Cds1 | View | View |  | 273 | 1.53 | 4.02 | - | 2.03 |  |  | CDP-DIACYLGLYCEROL SYNTHASE 1 | 4334 | 2824 | 3685 | 916 | 4334 | 3462 | 4010 | 1975 | chr5:102005432-102064154 | 1428680\_at |
| Cebpd | View | View |  | 324 | - | 0.20 | - | - |  |  | CCAAT/ENHANCER BINDING PROTEIN (C/EBP), DELTA | 871 | 1049 | 432 | 2149 | 871 | 960 | 652 | 473 | chr16:15800903-15803123 | 1423233\_at |
| Cep290 | View | View |  | 502 | - | - | - | - |  |  | CENTROSOMAL PROTEIN 290 | 1467 | 1185 | 1397 | 1094 | 1467 | 1535 | 1432 | 1348 | chr10:99917995-100003343 | 1425642\_at |
| Chd4 | View | View |  | 975 | - | - | 0.17 | - |  |  | CHROMODOMAIN HELICASE DNA BINDING PROTEIN 4 | 27 | 52 | 126 | 147 | 27 | 155 | 77 | 173 | chr6:125061782-125096133 | 1438476\_a\_at |
| Chrnb4 | View | View |  | - | - | 0.17 | 0.20 | 0.04 |  |  | CHOLINERGIC RECEPTOR, NICOTINIC, BETA POLYPEPTIDE 4 | 106 | 201 | 117 | 702 | 106 | 531 | 112 | 2764 | chr9:54826293-54846727 | 1457008\_at, 1425849\_at |
| Clca3 | View | View |  | - | 0.06 | - | 0.06 | 0.33 | Link |  | CHLORIDE CHANNEL CALCIUM ACTIVATED 3 | 230 | 3926 | 133 | 76 | 230 | 3669 | 182 | 558 | chr3:144941923-144970158 | 1416306\_at |
| Cldn7 | View | View |  | 844 | - | 7.38 | 0.56 | 3.76 |  |  | CLAUDIN 7 | 170 | 205 | 236 | 32 | 170 | 305 | 203 | 54 | chr11:69781696-69784073 | 1448393\_at |
| Clic4 | View | View |  | 249 | 0.51 | - | 0.32 | 0.27 |  |  | CHLORIDE INTRACELLULAR CHANNEL 4 (MITOCHONDRIAL) | 663 | 1291 | 769 | 1143 | 663 | 2055 | 716 | 2654 | chr4:134486045-134544836 | 1438606\_a\_at |
| Clpb | View | View |  | 362 | - | - | 0.32 | 0.38 |  |  | CLPB CASEINOLYTIC PEPTIDASE B HOMOLOG (E. COLI) | 420 | 578 | 483 | 652 | 420 | 1328 | 452 | 1204 | chr7:101537589-101663989 | 1416541\_at |
| Cltb | View | View |  | - | - | 0.28 | 0.21 | 0.24 | Link |  | CLATHRIN, LIGHT POLYPEPTIDE (LCB) | 2026 | 2333 | 2683 | 9553 | 2026 | 9475 | 2355 | 9784 | chr13:54602561-54620896 | 1453063\_at, 1460740\_at, 1456797\_at |
| Cnga1 | View | View |  | 1573 | - | 3.33 | 3315.50 | 1600.88 |  | Link | CYCLIC NUCLEOTIDE GATED CHANNEL ALPHA 1 | 13262 | 10718 | 12352 | 3708 | 13262 | 4 | 12807 | 8 | chr5:72883250-72916981 | 1451763\_at |
| Cnga3 | View | View |  | 340 | NA | NA | NA | NA |  |  | CYCLIC NUCLEOTIDE GATED CHANNEL ALPHA 3 |  |  |  |  |  |  |  |  | chr1:37163807-37207932 |  |
| Cngb1b | View | View |  | 464 | - | 9.63 | 17.23 | 75.75 | Link | Link | CYCLIC NUCLEOTIDE GATED CHANNEL BETA 1B | 7513 | 7150 | 5364 | 557 | 7513 | 436 | 6439 | 85 | chr8:98128177-98195714 | 1451549\_at |
| Cngb3 | View | View |  | 1661 | 0.35 | 0.62 | 0.07 | 0.06 |  |  | CYCLIC NUCLEOTIDE GATED CHANNEL BETA 3 | 520 | 1480 | 503 | 817 | 520 | 7755 | 512 | 7983 | chr4:19208080-19432497 | 1450492\_at |
| Col19a1 | View | View |  | 633 | 2.35 | 14.68 | 9.78 | 8.81 |  |  | PROCOLLAGEN, TYPE XIX, ALPHA 1 | 479 | 204 | 279 | 19 | 479 | 49 | 379 | 43 | chr1:24218269-24541686 | 1421698\_a\_at |
| Col4a4 | View | View |  | 305 | - | - | 7.36 | - |  |  | PROCOLLAGEN, TYPE IV, ALPHA 4 | 331 | 107 | 95 | 52 | 331 | 45 | 213 | 42 | chr1:82331582-82465816 | 1440250\_at |
| Cplx2 | View | View |  | - | - | 0.59 | 0.22 | - |  |  | COMPLEXIN 2 | 395 | 470 | 1459 | 2461 | 395 | 1822 | 927 | 1815 | chr13:54380974-54389837 | 1421477\_at |
| Cpm | View | View |  | 237 | 4.20 | 8.25 | 31.50 | 25.80 |  |  | CARBOXYPEPTIDASE M | 252 | 60 | 264 | 32 | 252 | 8 | 258 | 10 | chr10:117032498-117088295 | 1453009\_at, 1429413\_at |
| Crb1 | View | View |  | 786 | 0.28 | 7.98 | 1.73 | 9.17 |  |  | CRUMBS HOMOLOG 1 (DROSOPHILA) | 2076 | 7535 | 7700 | 965 | 2076 | 1200 | 4888 | 533 | chr1:141014754-141193511 | 1441330\_at |
| Crot | View | View |  | - | - | 0.38 | 0.32 | 0.42 |  |  | CARNITINE O-OCTANOYLTRANSFERASE | 1565 | 1619 | 1621 | 4265 | 1565 | 4861 | 1593 | 3780 | chr5:8972045-9003167 | 1450966\_at |
| Crx | View | View |  | 253 | - | 1.87 | - | 2.05 | Link | Link | CONE-ROD HOMEOBOX CONTAINING GENE | 13926 | 15097 | 14279 | 7632 | 13926 | 12985 | 14103 | 6888 | chr7:15024471-15038416 | 1418705\_at |
| Crxos1 | View | View |  | - | 0.66 | 1.48 | 0.19 | 0.37 |  |  | CRX OPPOSITE STRAND TRANSCRIPT 1 | 606 | 922 | 621 | 420 | 606 | 3195 | 614 | 1650 | chr7:15054646-15062531 | 1460605\_at |
| Crym | View | View |  | - | - | 0.37 | - | - |  |  | CRYSTALLIN, MU | 3232 | 2718 | 2128 | 5721 | 3232 | 3535 | 2680 | 3324 | chr7:119977533-119993137 | 1416776\_at |
| Csda | View | View |  | 442 | - | 1.99 | 2.74 | 3.08 |  |  | COLD SHOCK DOMAIN PROTEIN A | 15518 | 14644 | 13721 | 6899 | 15518 | 5667 | 14620 | 4748 | chr6:131330551-131354143 | 1435800\_a\_at, 1451012\_a\_at |
| Ctdsp2 | View | View |  | 254 | - | - | 0.16 | - |  |  | CTD (CARBOXY-TERMINAL DOMAIN, RNA POLYMERASE II, POLYPEPTIDE A) SMALL PHOSPHATASE 2 | 43 | 59 | 182 | 235 | 43 | 269 | 113 | 160 | chr10:126398560-126402918 | 1423660\_at |
| Cth | View | View |  | 248 | - | - | 0.31 | - |  |  | CYSTATHIONASE (CYSTATHIONINE GAMMA-LYASE) | 329 | 257 | 518 | 410 | 329 | 1052 | 424 | 369 | chr3:157829541-157860356 | 1426243\_at |
| Ctnnd1 | View | View |  | 476 | - | - | 0.21 | - |  |  | CATENIN (CADHERIN ASSOCIATED PROTEIN), DELTA 1 | 22 | 89 | 22 | 105 | 22 | 106 | 22 | 76 | chr2:84401622-84451514 | 1445830\_at |
| Cxx1c | View | View |  | - | 0.67 | 0.34 | 0.25 | 0.37 | Link |  | CAAX BOX 1 HOMOLOG C (HUMAN) | 2274 | 3376 | 2747 | 8043 | 2274 | 9245 | 2511 | 6714 | chrX:49802659-49803053 | 1450080\_at |
| Cyr61 | View | View |  | 359 | - | - | 0.20 | - |  |  | CYSTEINE RICH PROTEIN 61 | 68 | 159 | 121 | 92 | 68 | 337 | 95 | 87 | chr3:145584362-145587367 | 1438133\_a\_at |
| D130004H04Rik | View | View |  | 710 | - | - | 0.17 | - |  |  | RIKEN CDNA D130004H04 GENE | 114 | 128 | 595 | 765 | 114 | 658 | 355 | 656 | chr4:82270712-82273600 | 1435104\_at |
| D13Wsu177e | View | View |  | - | - | - | 0.17 | 0.49 |  |  | DNA SEGMENT, CHR 13, WAYNE STATE UNIVERSITY 177, EXPRESSED | 171 | 207 | 507 | 624 | 171 | 992 | 339 | 685 | chr13:54593813-54599696 | 1424620\_at |
| D1Bwg0212e | View | View |  | - | - | - | 0.19 | - |  |  | DNA SEGMENT, CHR 1, BRIGHAM & WOMEN'S GENETICS 0212 EXPRESSED | 51 | 64 | 209 | 196 | 51 | 262 | 130 | 178 | chr1:39480348-39491437 | 1452824\_at |
| D1Ertd622e | View | View |  | - | 1.53 | 2.95 | 3.63 | 3.64 |  |  | DNA SEGMENT, CHR 1, ERATO DOI 622, EXPRESSED | 1309 | 854 | 1319 | 447 | 1309 | 361 | 1314 | 361 | chr1:99474307-99492382 | 1448721\_at |
| D230044M03Rik | View | View |  | 677 | - | 0.00 | - | - |  |  | RIKEN CDNA D230044M03 GENE | 16 | 31 | 3 | 1340 | 16 | 4 | 10 | 3 | chr10:14405824-14408412 | 1430110\_at |
| Gene Symbol | Large Image Gene +/- 15kb | Closeup Image TSS +/- 15kb | Links | Max Score (threshold of 200) | *Nr2e3 -/-* | *Crx -/-* | *Nrl -/-* | *Crx;Nrl -/-* | In situ  hybridization | Electroporation | Full Name | Averaged Affy Scores | | | | | | | | Locus | Affy Mouse 430 2 |
|  |  |  |  |  |  |  |  |  |  |  |  | B6 | *Nr2e3 -/-* | 129 | *Crx -/-* | B6 | *Nrl -/-* | B6;129 | *Crx;Nrl -/-* |  |  |
| D330010C22Rik | View | View |  | 1618 | - | 8.84 | - | - |  |  | RIKEN CDNA 330010C22 GENE | 258 | 91 | 672 | 76 | 258 | 91 | 465 | 46 | chr4:151150823-151162408 | 1444404\_at, 1436277\_at |
| D430039N05Rik | View | View |  | 298 | - | - | 0.29 | - |  |  | RIKEN CDNA D430039N05 GENE | 1227 | 1635 | 4359 | 4109 | 1227 | 4167 | 2793 | 3496 | chr2:83613567-83682197 | 1436350\_at |
| D6Wsu176e | View | View |  | - | - | 3.33 | - | 2.94 |  |  | DNA SEGMENT, CHR 6, WAYNE STATE UNIVERSITY 176, EXPRESSED | 2973 | 2740 | 4027 | 1210 | 2973 | 1705 | 3500 | 1189 | chr6:22258450-22289388 | 1448904\_at, 1417953\_at |
| D730039F16Rik | View | View |  | - | - | 13.00 | - | - |  |  | RIKEN CDNA D730039F16 GENE | 101 | 57 | 117 | 9 | 101 | 85 | 109 | 8 | chr2:34696405-34714142 | 1425746\_at |
| D9Ertd292e | - | - |  | - | - | - | 0.17 | - |  |  | DNA SEGMENT, CHR 9, ERATO DOI 292, EXPRESSED | 22 | 7 | 50 | 49 | 22 | 127 | 36 | 63 | chr9 | 1446102\_at |
| Dcamkl1 | View | View |  | 370 | - | - | 0.11 | - |  |  | DOUBLE CORTIN AND CALCIUM/CALMODULIN-DEPENDENT PROTEIN KINASE-LIKE 1 | 11 | 14 | 90 | 103 | 11 | 103 | 51 | 114 | chr3:55330557-55624607 | 1451917\_a\_at |
| Ddit3 | View | View |  | 529 | - | - | 0.19 | - |  |  | DNA-DAMAGE INDUCIBLE TRANSCRIPT 3 | 40 | 28 | 58 | 164 | 40 | 216 | 49 | 142 | chr10:126693757-126699237 | 1443897\_at |
| Dgkd | View | View |  | - | - | - | 0.07 | - | Link |  | DIACYLGLYCEROL KINASE, DELTA | 8 | 5 | 42 | 134 | 8 | 121 | 25 | 165 | chr1:89711545-89775660 | 1443781\_at, 1442254\_at |
| Dhrs3 | View | View |  | 262 | 1.72 | 1.81 | 4.78 | 8.83 |  |  | DEHYDROGENASE/REDUCTASE (SDR FAMILY) MEMBER 3 | 1224 | 711 | 1126 | 621 | 1224 | 256 | 1175 | 133 | chr4:144159764-144194896 | 1448390\_a\_at |
| Dleu7 | View | View |  | - | - | 6.60 | 2.84 | 4.28 |  |  | DELETED IN LYMPHOCYTIC LEUKEMIA, 7 | 179 | 235 | 376 | 57 | 179 | 63 | 278 | 65 | chr14:61230339-61247073 | 1442180\_at |
| Dnajc9 | View | View |  | - | - | - | 0.28 | - |  |  | DNAJ (HSP40) HOMOLOG, SUBFAMILY C, MEMBER 9 | 427 | 380 | 1516 | 1438 | 427 | 1520 | 972 | 1671 | chr14:19173190-19177432 | 1426473\_at |
| Dnm1l | View | View |  | - | - | - | 0.18 | - |  |  | DYNAMIN 1-LIKE | 108 | 80 | 690 | 656 | 108 | 588 | 399 | 659 | chr16:16225811-16272608 | 1452638\_s\_at |
| Dock6 | View | View |  | - | - | 0.20 | - | - |  |  | DEDICATOR OF CYTOKINESIS 6 | 52 | 37 | 25 | 122 | 52 | 128 | 39 | 70 | chr9:21550590-21603018 | 1427240\_at |
| Dock8 | View | View |  | 240 | - | 5.44 | - | - |  |  | DEDICATOR OF CYTOKINESIS 8 | 224 | 150 | 484 | 89 | 224 | 113 | 354 | 178 | chr19:25066626-25269529 | 1449419\_at |
| Dock9 | View | View |  | 226 | 0.06 | - | 0.02 | - |  |  | DEDICATOR OF CYTOKINESIS 9 | 16 | 273 | 621 | 552 | 16 | 794 | 319 | 464 | chr14:120677334-120873642 | 1439200\_x\_at |
| Dpf3 | View | View |  | - | 1.63 | 4.97 | 3.40 | 6.86 |  |  | D4, ZINC AND DOUBLE PHD FINGERS, FAMILY 3 | 747 | 457 | 1173 | 236 | 747 | 220 | 960 | 140 | chr12:84188281-84260507 | 1450162\_at, 1442686\_at |
| Drd4 | View | View |  | - | - | 2.22 | - | 19.94 |  |  | DOPAMINE RECEPTOR 4 | 7173 | 8399 | 7942 | 3571 | 7173 | 7084 | 7558 | 379 | chr7:141143350-141147783 | 1422830\_s\_at |
| Dynlt3 | View | View |  | 217 | - | - | 0.20 | - |  |  | DYNEIN LIGHT CHAIN TCTEX-TYPE 3 | 457 | 416 | 2169 | 2157 | 457 | 2331 | 1313 | 2065 | chrX:8811229-8819942 | 1449928\_at |
| Dyrk2 | View | View |  | 1035 | - | 1.66 | 3.66 | 2.64 |  |  | DUAL-SPECIFICITY TYROSINE-(Y)-PHOSPHORYLATION REGULATED KINASE 2 | 4463 | 3322 | 2271 | 1370 | 4463 | 1221 | 3367 | 1276 | chr10:118262338-118271896 | 1436918\_at, 1428637\_at |
| E130012K09 | View | View |  | 903 | - | - | 0.39 | - | Link |  | HYPOTHETICAL PROTEIN E130012K09 | 5206 | 6100 | 208 | 201 | 5206 | 13197 | 2707 | 4519 | chr10:93723464-93728502 | 1457737\_at |
| E130304F04Rik | - | - |  | - | - | 5.79 | 3.73 | 6.37 |  |  | RIKEN CDNA E130304F04 GENE | 1130 | 783 | 1187 | 205 | 1130 | 303 | 1159 | 182 | chrNT\_110857:66905-112693 | 1434297\_at |
| Ebpl | View | View |  | 686 | - | 0.57 | 8.15 | 3.49 |  |  | EMOPAMIL BINDING PROTEIN-LIKE | 611 | 911 | 513 | 903 | 611 | 75 | 562 | 161 | chr14:60294860-60314549 | 1458177\_at, 1417298\_at |
| Ece1 | View | View |  | 1397 | 0.47 | 0.63 | 0.29 | 0.32 |  |  | ENDOTHELIN CONVERTING ENZYME 1 | 709 | 1516 | 619 | 980 | 709 | 2435 | 664 | 2068 | chr4:137185658-137237301 | 1455741\_a\_at, 1434177\_at, 1441423\_at |
| Edn2 | View | View |  | 439 | - | - | 9.63 | - |  |  | ENDOTHELIN 2 | 770 | 796 | 102 | 121 | 770 | 80 | 436 | 34 | chr4:119658836-119664996 | 1449161\_at |
| Egf | View | View |  | 246 | - | 8.46 | 0.77 | 11.22 |  |  | EPIDERMAL GROWTH FACTOR | 553 | 677 | 457 | 54 | 553 | 715 | 505 | 45 | chr3:129669600-129747338 | 1443066\_at |
| Egr1 | View | View |  | 1608 | 0.42 | 0.21 | - | 0.15 |  |  | EARLY GROWTH RESPONSE 1 | 697 | 1671 | 1557 | 7420 | 697 | 1348 | 1127 | 7591 | chr18:34987181-34990930 | 1417065\_at |
| Eif2s3x | View | View |  | - | - | - | 0.33 | - |  |  | EUKARYOTIC TRANSLATION INITIATION FACTOR 2, SUBUNIT 3, STRUCTURAL GENE X-LINKED | 575 | 486 | 1851 | 1774 | 575 | 1755 | 1213 | 1802 | chrX:90441422-90465370 | 1421895\_at |
| Eif3s8 | View | View |  | - | - | - | 0.22 | - |  |  | EUKARYOTIC TRANSLATION INITIATION FACTOR 3, SUBUNIT 8 | 523 | 507 | 1871 | 2003 | 523 | 2330 | 1197 | 2187 | chr7:126338055-126357551 | 1415859\_at |
| Elovl2 | View | View |  | 432 | 0.20 | 0.71 | 0.24 | 0.21 |  |  | ELONGATION OF VERY LONG CHAIN FATTY ACIDS (FEN1/ELO2, SUR4/ELO3, YEAST)-LIKE 2 | 1155 | 5685 | 1054 | 1484 | 1155 | 4799 | 1105 | 5257 | chr13:41196268-41208172 | 1416444\_at |
| Elovl4 | View | View |  | 230 | - | - | 1.48 | 1.37 |  | Link | ELONGATION OF VERY LONG CHAIN FATTY ACIDS (FEN1/ELO2, SUR4/ELO3, YEAST)-LIKE 4 | 9352 | 9868 | 8148 | 6914 | 9352 | 6306 | 8750 | 6401 | chr9:83575418-83602883 | 1424306\_at |
| Elovl7 | View | View |  | - | - | - | 0.14 | 0.08 |  |  | ELOVL FAMILY MEMBER 7, ELONGATION OF LONG CHAIN FATTY ACIDS (YEAST) | 43 | 60 | 88 | 67 | 43 | 303 | 66 | 829 | chr13:109335180-109405803 | 1424098\_at, 1440312\_at, 1441891\_x\_at |
| En2 | View | View |  | 973 | - | - | 0.09 | - |  |  | ENGRAILED 2 | 40 | 59 | 59 | 35 | 40 | 466 | 50 | 93 | chr5:28497267-28502960 | 1418868\_at |
| Enpp5 | View | View |  | - | - | - | 0.35 | - | Link |  | ECTONUCLEOTIDE PYROPHOSPHATASE/PHOSPHODIESTERASE 5 | 1805 | 1716 | 4606 | 4076 | 1805 | 5164 | 3206 | 4912 | chr17:43541924-43549614 | 1425702\_a\_at |
| Epas1 | View | View |  | - | 2.14 | - | 2.09 | 2.11 |  |  | ENDOTHELIAL PAS DOMAIN PROTEIN 1 | 6613 | 3090 | 4819 | 5100 | 6613 | 3158 | 5716 | 2704 | chr17:86662228-86739907 | 1435436\_at |
| Epb4.1l1 | View | View |  | - | 0.33 | - | - | - |  |  | ERYTHROCYTE PROTEIN BAND 4.1-LIKE 1 | 1063 | 3257 | 1069 | 1113 | 1063 | 1422 | 1066 | 1221 | chr2:156112525-156228930 | 1460592\_at |
| Epha10 | View | View |  | - | 0.12 | - | 0.17 | 0.51 |  |  | EPH RECEPTOR A10 | 342 | 2748 | 266 | 135 | 342 | 1994 | 304 | 591 | chr4:124384128-124388812 | 1436093\_at |
| Erdr1 | - | - |  | - | 0.24 | - | 0.07 | - |  |  | ERYTHROID DIFFERENTIATION REGULATOR 1 | 282 | 1192 | 3068 | 2590 | 282 | 4323 | 1675 | 2566 | - | 1452406\_x\_at |
| Ermap | View | View |  | 363 | - | - | 0.12 | 0.01 |  |  | ERYTHROBLAST MEMBRANE-ASSOCIATED PROTEIN | 33 | 96 | 89 | 147 | 33 | 268 | 61 | 7786 | chr4:118673522-118687908 | 1418909\_at |
| Esrrb | View | View |  | 1503 | - | 5.20 | 6.67 | 14.13 | Link |  | ESTROGEN RECEPTOR RELATED 2 | 580 | 448 | 946 | 182 | 580 | 87 | 763 | 54 | chr12:87250220-87410725 | 1422986\_at, 1436926\_at, 1441921\_x\_at |
| Ewsr1 | View | View |  | 448 | - | 5.91 | 1.74 | - |  |  | EWING SARCOMA BREAKPOINT REGION 1 | 643 | 445 | 880 | 149 | 643 | 370 | 762 | 205 | chr11:4969701-4999070 | 1425919\_at |
| Ext1 | View | View |  | 260 | 2.14 | - | 3.71 | - |  |  | EXOSTOSES (MULTIPLE) 1 | 1801 | 841 | 870 | 1239 | 1801 | 485 | 1336 | 532 | chr15:52898747-53175446 | 1417730\_at |
| Eya1 | View | View |  | 1389 | - | - | 0.09 | 0.04 |  |  | EYES ABSENT 1 HOMOLOG (DROSOPHILA) | 17 | 27 | 24 | 72 | 17 | 182 | 21 | 594 | chr1:14154172-14295413 | 1443117\_at, 1457424\_at, 1421727\_at |
| Fabp5 | View | View |  | - | - | 0.42 | - | - |  |  | FATTY ACID BINDING PROTEIN 5, EPIDERMAL | 3568 | 3786 | 3441 | 8212 | 3568 | 3083 | 3505 | 4571 | chr3:9995121-9999139 | 1416021\_a\_at |
| Fabp7 | View | View |  | - | 0.03 | 0.04 | 0.02 | 0.02 |  |  | FATTY ACID BINDING PROTEIN 7, BRAIN | 118 | 3761 | 261 | 6229 | 118 | 5796 | 190 | 10899 | chr10:57473338-57476865 | 1450779\_at |
| Faim | View | View |  | 507 | - | 5.36 | 4.04 | 4.98 |  |  | FAS APOPTOTIC INHIBITORY MOLECULE | 3846 | 4244 | 5220 | 973 | 3846 | 951 | 4533 | 910 | chr9:98795725-98811370 | 1418029\_at |
| Fbxw11 | View | View |  | 259 | - | - | 0.17 | - |  |  | F-BOX AND WD-40 DOMAIN PROTEIN 11 | 20 | 28 | 158 | 138 | 20 | 116 | 89 | 142 | chr11:32542748-32646816 | 1438336\_at |
| Fgf11 | View | View |  | 757 | - | - | 0.17 | - |  |  | FIBROBLAST GROWTH FACTOR 11 | 24 | 42 | 120 | 115 | 24 | 140 | 72 | 124 | chr11:69612264-69617911 | 1421793\_at |
| Fin15 | View | View |  | - | - | 0.57 | 0.23 | 0.35 |  |  | FIBROBLAST GROWTH FACTOR INDUCIBLE 15 | 1007 | 1124 | 1597 | 2820 | 1007 | 4382 | 1302 | 3754 | chr6:50131321-50131584 | 1421534\_at, 1439263\_at |
| Fkbp5 | View | View |  | - | - | 0.33 | - | - |  |  | FK506 BINDING PROTEIN 5 | 681 | 655 | 562 | 1685 | 681 | 602 | 622 | 859 | chr17:28126686-28213738 | 1416125\_at |
| Fkbp9 | View | View |  | 627 | - | 0.61 | 0.30 | - |  |  | FK506 BINDING PROTEIN 9 | 843 | 849 | 990 | 1619 | 843 | 2854 | 917 | 1193 | chr6:56761637-56808935 | 1437687\_x\_at |
| Gene Symbol | Large Image Gene +/- 15kb | Closeup Image TSS +/- 15kb | Links | Max Score (threshold of 200) | *Nr2e3 -/-* | *Crx -/-* | *Nrl -/-* | *Crx;Nrl -/-* | In situ  hybridization | Electroporation | Full Name | Averaged Affy Scores | | | | | | | | Locus | Affy Mouse 430 2 |
|  |  |  |  |  |  |  |  |  |  |  |  | B6 | *Nr2e3 -/-* | 129 | *Crx -/-* | B6 | *Nrl -/-* | B6;129 | *Crx;Nrl -/-* |  |  |
| Fntb | View | View |  | - | - | 5.61 | - | - |  |  | FARNESYLTRANSFERASE, CAAX BOX, BETA | 65 | 37 | 393 | 70 | 65 | 26 | 229 | 39 | chr12:77756307-77840254 | 1459043\_at |
| Frmd6 | View | View |  | 984 | - | - | 0.17 | - |  |  | FERM DOMAIN CONTAINING 6 | 46 | 79 | 92 | 237 | 46 | 264 | 69 | 327 | chr12:71744119-71820839 | 1457184\_at |
| Frmpd1 | View | View |  | - | - | 7.41 | 3.96 | 7.69 |  |  | FERM AND PDZ DOMAIN CONTAINING 1 | 2708 | 3041 | 1749 | 236 | 2708 | 684 | 2229 | 290 | chr4:45265731-45307036 | 1435189\_at |
| Fscn2 | View | View |  | - | - | 111.17 | 10.04 | 14.32 | Link |  | FASCIN HOMOLOG 2, ACTIN-BUNDLING PROTEIN, RETINAL (STRONGYLOCENTROTUS PURPURATUS) | 1767 | 1976 | 667 | 6 | 1767 | 176 | 1217 | 85 | chr11:120177624-120184261 | 1440605\_at |
| Fus | View | View |  | 919 | - | - | 0.38 | - |  |  | FUSION, DERIVED FROM T(12;16) MALIGNANT LIPOSARCOMA (HUMAN) | 2281 | 2140 | 4382 | 4652 | 2281 | 5936 | 3332 | 5534 | chr7:95337213-95338638 | 1451285\_at |
| Gabra2 | View | View |  | 583 | - | - | 0.19 | - |  |  | GAMMA-AMINOBUTYRIC ACID (GABA-A) RECEPTOR, SUBUNIT ALPHA 2 | 81 | 67 | 608 | 486 | 81 | 418 | 345 | 406 | chr5:71240192-71374984 | 1421738\_at |
| Galnt10 | View | View |  | - | 0.69 | 1.90 | 3.65 | 2.47 |  |  | UDP-N-ACETYL-ALPHA-D-GALACTOSAMINE:POLYPEPTIDE N-ACETYLGALACTOSAMINYLTRANSFERASE 10 | 1127 | 1623 | 820 | 432 | 1127 | 309 | 974 | 394 | chr11:57461637-57603696 | 1418195\_at |
| Galnt13 | View | View |  | 314 | - | 0.43 | 0.17 | 0.27 |  |  | UDP-N-ACETYL-ALPHA-D-GALACTOSAMINE:POLYPEPTIDE N-ACETYLGALACTOSAMINYLTRANSFERASE 13 | 289 | 399 | 470 | 1090 | 289 | 1714 | 380 | 1404 | chr2:54331926-54928347 | 1457045\_at |
| Gas7 | View | View |  | - | 0.74 | 8.49 | 2.83 | 4.09 |  |  | GROWTH ARREST SPECIFIC 7 | 1009 | 1356 | 781 | 92 | 1009 | 357 | 895 | 219 | chr11:67363349-67501169 | 1417859\_at |
| Gckr | View | View |  | - | 0.30 | 3.77 | 0.52 | 2.73 |  |  | GLUCOKINASE REGULATORY PROTEIN | 869 | 2933 | 1249 | 331 | 869 | 1674 | 1059 | 388 | chr5:31574162-31603883 | 1426059\_at |
| Gfap | View | View |  | 780 | - | 0.13 | 1.93 | - |  |  | GLIAL FIBRILLARY ACIDIC PROTEIN | 680 | 875 | 155 | 1210 | 680 | 353 | 418 | 187 | chr11:102703437-102713221 | 1426509\_s\_at, 1440142\_s\_at, 1426508\_at |
| Gfra1 | View | View |  | 1193 | - | - | 0.19 | - |  |  | GLIAL CELL LINE DERIVED NEUROTROPHIC FACTOR FAMILY RECEPTOR ALPHA 1 | 30 | 17 | 121 | 124 | 30 | 154 | 76 | 97 | chr19:58288862-58508567 | 1421973\_at |
| Ggtl3 | View | View |  | 367 | - | - | 0.32 | - |  |  | GAMMA-GLUTAMYLTRANSFERASE-LIKE 3 | 334 | 368 | 898 | 1176 | 334 | 1030 | 616 | 1088 | chr2:155181828-155206284 | 1451094\_at |
| Gja9 | View | View |  | 467 | - | - | 0.31 | - |  |  | GAP JUNCTION MEMBRANE CHANNEL PROTEIN ALPHA 9 | 345 | 333 | 1048 | 793 | 345 | 1105 | 697 | 871 | chr2:113701037-113705060 | 1423019\_at |
| Glipr2 | View | View |  | 1020 | - | 0.17 | - | - |  |  | GLI PATHOGENESIS-RELATED 2 | 22 | 13 | 36 | 214 | 22 | 51 | 29 | 29 | chr4:43978802-44000218 | 1428492\_at |
| Glo1 | View | View |  | 534 | 0.63 | 0.78 | 0.36 | 0.53 |  |  | GLYOXALASE 1 | 5766 | 9219 | 11834 | 15228 | 5766 | 15812 | 8800 | 16667 | chr17:30319878-30339627 | 1424109\_a\_at, 1424108\_at, 1451240\_a\_at |
| Glra1 | View | View |  | 886 | - | - | 0.18 | - |  |  | GLYCINE RECEPTOR, ALPHA 1 SUBUNIT | 97 | 173 | 254 | 224 | 97 | 532 | 176 | 430 | chr11:55358236-55417834 | 1437139\_at |
| Gls | View | View |  | 319 | - | - | 0.19 | - |  |  | GLUTAMINASE | 160 | 161 | 512 | 495 | 160 | 848 | 336 | 692 | chr1:52129826-52177777 | 1435708\_at |
| Gm1582 | View | View |  | - | 1.68 | 22.18 | 3.31 | 25.33 |  |  | GENE MODEL 1582, (NCBI) | 4230 | 2520 | 3926 | 177 | 4230 | 1279 | 4078 | 161 | chr14:32394390-32404346 | 1458506\_at |
| Gm1683 | View | View |  | 645 | - | 0.16 | - | - |  |  | GENE MODEL 1683, (NCBI) | 9 | 25 | 39 | 245 | 9 | 4 | 24 | 38 | chr5:64872998-64889834 | 1449600\_at |
| Gnat1 | View | View |  | 449 | - | 32.25 | 1095.62 | 583.00 | Link | Link | GUANINE NUCLEOTIDE BINDING PROTEIN, ALPHA TRANSDUCING 1 | 37251 | 37675 | 31543 | 978 | 37251 | 34 | 34397 | 59 | chr9:107532576-107537716 | 1460212\_at |
| Gnat2 | View | View |  | 774 | 0.21 | - | 0.22 | 0.30 | Link |  | GUANINE NUCLEOTIDE BINDING PROTEIN, ALPHA TRANSDUCING 2 | 6558 | 31411 | 6786 | 8343 | 6558 | 29421 | 6672 | 22199 | chr3:108221124-108229485 | 1422907\_at |
| Gnb1 | View | View |  | 666 | - | 4.68 | 30.09 | 9.04 | Link |  | GUANINE NUCLEOTIDE BINDING PROTEIN, BETA 1 | 18627 | 16893 | 30106 | 6432 | 18627 | 619 | 24367 | 2695 | chr4:154335170-154401616 | 1417432\_a\_at, 1454696\_at |
| Gnb2 | View | View |  | 813 | - | 0.33 | 0.54 | 0.59 |  |  | GUANINE NUCLEOTIDE BINDING PROTEIN, BETA 2 | 2863 | 3324 | 2752 | 8402 | 2863 | 5291 | 2808 | 4734 | chr5:137757915-137763022 | 1450623\_at |
| Gnb3 | View | View |  | 640 | 0.52 | 0.48 | 0.40 | 0.51 | Link |  | GUANINE NUCLEOTIDE BINDING PROTEIN, BETA 3 | 8936 | 17104 | 6396 | 13273 | 8936 | 22620 | 7666 | 15134 | chr6:124799859-124805894 | 1449159\_at |
| Gng11 | View | View |  | 849 | - | 0.30 | - | - |  |  | GUANINE NUCLEOTIDE BINDING PROTEIN (G PROTEIN), GAMMA 11 | 478 | 477 | 375 | 1241 | 478 | 452 | 427 | 920 | chr6:3953987-3958445 | 1448942\_at |
| Gngt1 | View | View |  | 849 | - | 3.15 | 2.03 | 2.34 | Link |  | GUANINE NUCLEOTIDE BINDING PROTEIN (G PROTEIN), GAMMA TRANSDUCING ACTIVITY POLYPEPTIDE 1 | 13938 | 10711 | 12013 | 3811 | 13938 | 6870 | 12976 | 5539 | chr6:3944012-3947436 | 1425167\_a\_at, 1451633\_a\_at, 1425168\_at |
| Gngt2 | View | View |  | 206 | - | 0.31 | 0.18 | 0.13 | Link |  | GUANINE NUCLEOTIDE BINDING PROTEIN (G PROTEIN), GAMMA TRANSDUCING ACTIVITY POLYPEPTIDE 2 | 115 | 268 | 148 | 472 | 115 | 634 | 132 | 1002 | chr11:95658760-95661819 | 1428733\_at |
| Gosr1 | View | View |  | 372 | - | - | 0.10 | - |  |  | GOLGI SNAP RECEPTOR COMPLEX MEMBER 1 | 17 | 27 | 121 | 146 | 17 | 163 | 69 | 128 | chr11:76542797-76579750 | 1448256\_at |
| Gpr178 | View | View |  | 410 | - | - | 0.28 | - |  |  | G PROTEIN-COUPLED RECEPTOR 178 | 299 | 211 | 1161 | 1216 | 299 | 1065 | 730 | 713 | chr17:6213432-6408178 | 1440862\_at, 1452848\_at |
| Gpr98 | View | View |  | - | - | 1.75 | 1.45 | 2.31 |  |  | G PROTEIN-COUPLED RECEPTOR 98 | 931 | 967 | 995 | 570 | 931 | 640 | 963 | 416 | chr13:81558819-82072776 | 1425314\_at |
| Gpsm2 | View | View |  | 505 | 0.58 | 7.96 | 6.18 | 10.86 |  |  | G-PROTEIN SIGNALLING MODULATOR 2 (AGS3-LIKE, C. ELEGANS) | 4416 | 7616 | 4771 | 599 | 4416 | 714 | 4594 | 423 | chr3:108806694-108850199 | 1424895\_at |
| Greb1 | View | View |  | 2313 | - | - | 0.17 | - |  |  | GENE REGULATED BY ESTROGEN IN BREAST CANCER PROTEIN | 49 | 81 | 192 | 263 | 49 | 284 | 121 | 214 | chr12:16697094-16765652 | 1419593\_at |
| Grk1 | View | View |  | - | - | 2.62 | - | - | Link |  | G PROTEIN-COUPLED RECEPTOR KINASE 1 | 674 | 529 | 2195 | 837 | 674 | 626 | 1435 | 799 | chr8:13405058-13417557 | 1421361\_at |
| Grtp1 | View | View |  | - | - | 5.75 | 3.81 | 5.40 |  |  | GH REGULATED TBC PROTEIN 1 | 2210 | 2288 | 1339 | 233 | 2210 | 580 | 1775 | 329 | chr8:13176846-13200601 | 1439150\_x\_at, 1425891\_a\_at |
| Gtf2i | View | View |  | - | - | - | 0.12 | - |  |  | GENERAL TRANSCRIPTION FACTOR II I | 38 | 25 | 220 | 238 | 38 | 329 | 129 | 275 | chr5:134522470-134599357 | 1431675\_a\_at, 1431676\_x\_at |
| Guca1a | View | View |  | 927 | 0.61 | - | 0.78 | - |  |  | GUANYLATE CYCLASE ACTIVATOR 1A (RETINA) | 21635 | 35667 | 21160 | 18177 | 21635 | 27715 | 21398 | 22325 | chr17:46857749-46863775 | 1421061\_at |
| Guca1b | View | View |  | 226 | - | 23.30 | 5.56 | 12.43 | Link |  | GUANYLATE CYCLASE ACTIVATOR 1B | 22046 | 19720 | 9485 | 407 | 22046 | 3964 | 15766 | 1268 | chr17:46750145-46856158 | 1425138\_at, 1425441\_at |
| Gucy2e | View | View |  | 290 | - | 2.35 | - | - |  | Link | GUANYLATE CYCLASE 2E | 136 | 125 | 348 | 148 | 136 | 205 | 242 | 119 | chr11:69039230-69053217 | 1422193\_at |
| Gulo | View | View |  | 300 | 0.06 | 0.18 | 0.11 | 0.08 | Link |  | GULONOLACTONE (L-) OXIDASE | 522 | 8088 | 640 | 3535 | 522 | 4641 | 581 | 7237 | chr14:64941676-64962250 | 1451297\_at |
| Gzmm | View | View |  | - | 0.61 | 2.24 | 0.12 | 0.55 |  |  | GRANZYME M (LYMPHOCYTE MET-ASE 1) | 1168 | 1913 | 1598 | 714 | 1168 | 9803 | 1383 | 2522 | chr10:79092149-79098389 | 1449501\_a\_at |
| H2-Q6 | View | View |  | - | - | 0.07 | - | - |  |  | HISTOCOMPATIBILITY 2, Q REGION LOCUS 1 | 35 | 24 | 23 | 318 | 35 | 58 | 29 | 64 | chr17:35032930-35038102 | 1451644\_a\_at |
| Hcls1 | View | View |  | 357 | - | 39.78 | - | 9.86 |  |  | HEMATOPOIETIC CELL SPECIFIC LYN SUBSTRATE 1 | 213 | 115 | 358 | 9 | 213 | 113 | 286 | 29 | chr16:36856526-36881988 | 1418842\_at |
| Hcrtr1 | View | View |  | - | - | 0.20 | - | - |  |  | HYPOCRETIN (OREXIN) RECEPTOR 1 | 51 | 94 | 93 | 473 | 51 | 37 | 72 | 156 | chr4:129633145-129639932 | 1436295\_at |
| Herc3 | View | View |  | - | - | 3.29 | 2.46 | 2.66 |  |  | HECT DOMAIN AND RLD 3 | 2089 | 1729 | 2019 | 613 | 2089 | 850 | 2054 | 771 | chr6:58761467-58849978 | 1433590\_at |
| Hes5 | View | View |  | - | - | - | 0.15 | - |  |  | HAIRY AND ENHANCER OF SPLIT 5 (DROSOPHILA) | 35 | 87 | 127 | 111 | 35 | 230 | 81 | 214 | chr4:153804723-153806171 | 1423146\_at |
| Hist1h1c | View | View |  | 968 | - | 3.13 | - | - |  |  | HISTONE CLUSTER 1, H1C | 2235 | 1657 | 4158 | 1329 | 2235 | 1766 | 3197 | 1297 | chr13:23746272-23747831 | 1436994\_a\_at, 1416101\_a\_at |
| Hist3h2a | View | View |  | 357 | 0.59 | 0.64 | 0.37 | - |  |  | HISTONE CLUSTER 3, H2A | 3733 | 6280 | 2610 | 4057 | 3733 | 10082 | 3172 | 4618 | chr11:58770880-58789293 | 1435865\_at, 1435866\_s\_at, 1455712\_at |
| Hist3h2ba | View | View |  | 362 | 0.50 | 0.69 | 0.18 | 0.29 | Link |  | HISTONE CLUSTER 3, H2BA | 990 | 1964 | 3696 | 5386 | 990 | 5576 | 2343 | 8108 | chr11:58765106-58765727 | 1449482\_at |
| Hk2 | View | View |  | - | 0.34 | 2.56 | 0.41 | - |  |  | HEXOKINASE 2 | 2841 | 8290 | 7314 | 2853 | 2841 | 6934 | 5078 | 4991 | chr6:82690705-82740117 | 1422612\_at |
| Gene Symbol | Large Image Gene +/- 15kb | Closeup Image TSS +/- 15kb | Links | Max Score (threshold of 200) | *Nr2e3 -/-* | *Crx -/-* | *Nrl -/-* | *Crx;Nrl -/-* | In situ  hybridization | Electroporation | Full Name | Averaged Affy Scores | | | | | | | | Locus | Affy Mouse 430 2 |
|  |  |  |  |  |  |  |  |  |  |  |  | B6 | *Nr2e3 -/-* | 129 | *Crx -/-* | B6 | *Nrl -/-* | B6;129 | *Crx;Nrl -/-* |  |  |
| Hlrc1 | View | View |  | 303 | - | 6.41 | 1.63 | 5.01 |  |  | HEAT-LIKE (PBS LYASE) REPEAT CONTAINING 1 | 3738 | 3695 | 3710 | 579 | 3738 | 2300 | 3724 | 744 | chr10:80787592-80797094 | 1436402\_at |
| Hmg20a | View | View |  | - | - | - | 0.08 | - |  |  | HIGH MOBILITY GROUP 20A | 10 | 24 | 53 | 55 | 10 | 118 | 32 | 108 | chr9:56216781-56295021 | 1438043\_at |
| Hmgb2 | View | View |  | 365 | - | 3.43 | - | 2.65 |  |  | HIGH MOBILITY GROUP BOX 2 | 720 | 1052 | 1125 | 328 | 720 | 572 | 923 | 348 | chr8:60404049-60406636 | 1452534\_a\_at |
| Hnrpa1 | View | View |  | 537 | - | - | 0.19 | - |  |  | HETEROGENEOUS NUCLEAR RIBONUCLEOPROTEIN A1 | 67 | 81 | 226 | 217 | 67 | 354 | 147 | 189 | chr15:103068451-103072048 | 1455740\_at |
| Hrasls | View | View |  | 1596 | 0.25 | - | 0.14 | 0.19 |  |  | HRAS-LIKE SUPPRESSOR | 71 | 282 | 130 | 79 | 71 | 502 | 101 | 518 | chr16:29137359-29147981 | 1422919\_at |
| Hspa1b | View | View |  | - | 1.50 | 8.78 | 2.25 | 5.56 |  |  | HEAT SHOCK PROTEIN 1B | 2644 | 1764 | 1896 | 216 | 2644 | 1175 | 2270 | 408 | chr17:34565135-34567063 | 1427127\_x\_at, 1427126\_at, 1452318\_a\_at |
| Hspa9a | View | View |  | 655 | - | - | 0.11 | - |  |  | HEAT SHOCK PROTEIN 9A | 41 | 25 | 343 | 391 | 41 | 358 | 192 | 300 | chr18:35063391-35080287 | 1431274\_a\_at |
| Ibrdc2 | View | View |  | 216 | 0.49 | 9.95 | 4.07 | 8.56 |  |  | IBR DOMAIN CONTAINING 2 | 285 | 584 | 657 | 66 | 285 | 70 | 471 | 55 | chr13:47133692-47257448 | 1456834\_at |
| Icmt | View | View |  | 767 | 1.24 | 2.68 | 1.78 | 2.37 |  |  | ISOPRENYLCYSTEINE CARBOXYL METHYLTRANSFERASE | 7196 | 5826 | 6791 | 2536 | 7196 | 4053 | 6994 | 2948 | chr4:151141104-151150919 | 1426500\_at |
| Ier3 | View | View |  | - | - | 4.80 | - | 5.29 |  |  | IMMEDIATE EARLY RESPONSE 3 | 2174 | 1636 | 3431 | 715 | 2174 | 3298 | 2803 | 530 | chr17:35429737-35430969 | 1419647\_a\_at |
| Ier5 | View | View |  | 815 | - | - | 0.14 | - |  |  | IMMEDIATE EARLY RESPONSE 5 | 23 | 18 | 64 | 119 | 23 | 161 | 44 | 188 | chr1:156860722-156861648 | 1417613\_at |
| Igsf3 | View | View |  | 687 | - | - | 0.28 | 0.31 |  |  | IMMUNOGLOBULIN SUPERFAMILY, MEMBER 3 | 312 | 413 | 318 | 451 | 312 | 1125 | 315 | 1027 | chr3:101506186-101589911 | 1431322\_at |
| Igsf4a | View | View |  | 1218 | - | - | 2.12 | 1.89 |  |  | IMMUNOGLOBULIN SUPERFAMILY, MEMBER 4A | 9307 | 9951 | 6519 | 5151 | 9307 | 4392 | 7913 | 4193 | chr9:47281252-47602023 | 1417378\_at |
| Ilvbl | View | View |  | - | - | - | 0.14 | - |  |  | ILVB (BACTERIAL ACETOLACTATE SYNTHASE)-LIKE | 137 | 124 | 751 | 603 | 137 | 1014 | 444 | 620 | chr10:77977671-77987622 | 1454658\_at |
| Impdh1 | View | View |  | 372 | - | 2.00 | - | 2.11 |  | Link | INOSINE 5'-PHOSPHATE DEHYDROGENASE 1 | 7065 | 7939 | 4803 | 2397 | 7065 | 5558 | 5934 | 2818 | chr6:29150447-29166360 | 1423239\_at |
| Inhbb | View | View |  | 599 | - | 0.18 | - | 0.11 |  |  | INHIBIN BETA-B | 211 | 265 | 212 | 1197 | 211 | 190 | 212 | 1958 | chr1:121243011-121249794 | 1426858\_at |
| Insm2 | View | View |  | 729 | - | 0.07 | - | 0.12 |  |  | INSULINOMA-ASSOCIATED 2 | 38 | 99 | 15 | 222 | 38 | 65 | 27 | 223 | chr12:56517603-56519083 | 1422269\_at, 1437781\_at |
| Ipmk | View | View |  | - | 1.42 | 3.02 | - | 2.15 |  |  | INOSITOL POLYPHOSPHATE MULTIKINASE | 1915 | 1351 | 1722 | 570 | 1915 | 1525 | 1819 | 845 | chr10:70743093-70781185 | 1456200\_at, 1436215\_at, 1437856\_at |
| Ipo4 | View | View |  | 254 | 0.20 | 0.59 | 0.59 | - |  |  | IMPORTIN 4 | 1578 | 7777 | 1320 | 2226 | 1578 | 2658 | 1449 | 1674 | chr14:54579824-54590028 | 1436420\_a\_at, 1450814\_a\_at |
| Iqcb1 | View | View |  | - | - | - | - | - |  |  | IQ CALMODULIN-BINDING MOTIF CONTAINING 1 | 577 | 474 | 560 | 612 | 577 | 608 | 569 | 651 | chr16:36747647-36791952 | 1434770\_at |
| Iqsec3 | View | View |  | 597 | 0.44 | - | - | - |  |  | IQ MOTIF AND SEC7 DOMAIN 3 | 4126 | 9400 | 3893 | 5409 | 4126 | 3248 | 4010 | 3085 | chr6:121338552-121439297 | 1437912\_at |
| Itpr3 | View | View |  | 247 | 0.13 | - | - | - |  |  | INOSITOL 1,4,5-TRIPHOSPHATE RECEPTOR 3 | 93 | 726 | 135 | 99 | 93 | 199 | 114 | 63 | chr17:26785125-26849812 | 1417297\_at |
| Jam3 | View | View |  | 268 | - | 0.25 | 0.19 | 0.20 |  |  | JUNCTION ADHESION MOLECULE 3 | 442 | 593 | 598 | 2359 | 442 | 2311 | 520 | 2620 | chr9:26846831-26904839 | 1423503\_at, 1444895\_at |
| Kcnab1 | View | View |  | 234 | - | 0.42 | - | 0.33 |  |  | POTASSIUM VOLTAGE-GATED CHANNEL, SHAKER-RELATED SUBFAMILY, BETA MEMBER 1 | 1364 | 1638 | 2151 | 5066 | 1364 | 1841 | 1758 | 5292 | chr3:65197313-65466155 | 1448468\_a\_at |
| Kcnb1 | View | View |  | - | - | 4.01 | 5.10 | 6.59 | Link |  | POTASSIUM VOLTAGE GATED CHANNEL, SHAB-RELATED SUBFAMILY, MEMBER 1 | 6738 | 5714 | 7719 | 1925 | 6738 | 1321 | 7229 | 1097 | chr2:166794583-166880004 | 1423180\_at, 1423179\_at |
| Kcne2 | View | View |  | 275 | - | 7.45 | 0.20 | 5.75 |  |  | POTASSIUM VOLTAGE-GATED CHANNEL, ISK-RELATED SUBFAMILY, GENE 2 | 2914 | 3505 | 3181 | 427 | 2914 | 14364 | 3048 | 530 | chr16:92181248-92186988 | 1449421\_a\_at |
| Kcnj14 | View | View |  | 956 | 1.61 | 49.25 | 96.57 | 121.67 |  |  | POTASSIUM INWARDLY-RECTIFYING CHANNEL, SUBFAMILY J, MEMBER 14 | 7629 | 4725 | 9160 | 186 | 7629 | 79 | 8395 | 69 | chr7:45684503-45692798 | 1452243\_at |
| Kcnv2 | View | View |  | 224 | - | 2.86 | - | 2.05 | Link | Link | POTASSIUM CHANNEL, SUBFAMILY V, MEMBER 2 | 2127 | 1657 | 2650 | 925 | 2127 | 2086 | 2389 | 1165 | chr19:27389685-27404275 | 1440537\_at |
| Khsrp | View | View |  | 289 | - | - | 3.95 | - |  |  | KH-TYPE SPLICING REGULATORY PROTEIN | 1368 | 1619 | 426 | 350 | 1368 | 346 | 897 | 309 | chr17:56706180-56716603 | 1428174\_x\_at |
| Kif1b | View | View |  | 616 | 1.65 | 1.97 | 3.48 | 3.21 |  |  | KINESIN FAMILY MEMBER 1B | 1191 | 721 | 551 | 279 | 1191 | 342 | 871 | 271 | chr4:148020119-148151498 | 1425270\_at |
| Kif5c | View | View |  | 1040 | - | - | 0.20 | - |  |  | KINESIN FAMILY MEMBER 5C | 135 | 140 | 830 | 873 | 135 | 664 | 483 | 839 | chr2:49441323-49596787 | 1422945\_a\_at |
| Klf3 | View | View |  | 314 | 0.58 | 0.56 | 0.32 | 0.44 |  |  | KRUPPEL-LIKE FACTOR 3 (BASIC) | 451 | 784 | 658 | 1176 | 451 | 1409 | 555 | 1264 | chr5:65082658-65109264 | 1454666\_at |
| Klf9 | View | View |  | 248 | - | - | 2.60 | 2.08 |  |  | KRUPPEL-LIKE FACTOR 9 | 5099 | 5245 | 3548 | 4422 | 5099 | 1964 | 4324 | 2079 | chr19:23208323-23234008 | 1428289\_at |
| Klhl18 | View | View |  | - | - | 2.71 | 3.12 | 3.10 |  |  | KELCH-LIKE 18 (DROSOPHILA) | 3527 | 3607 | 2483 | 916 | 3527 | 1130 | 3005 | 969 | chr9:110271094-110321510 | 1455543\_at |
| Klhl4 | View | View |  | - | 0.48 | 0.44 | 0.20 | 0.15 |  |  | KELCH-LIKE 4 (DROSOPHILA) | 208 | 429 | 260 | 597 | 208 | 1035 | 234 | 1551 | chrX:110591464-110677642 | 1439078\_at |
| Kpna2 | View | View |  | - | 1.45 | 4.74 | 1.67 | 3.69 | Link |  | KARYOPHERIN (IMPORTIN) ALPHA 2 | 10686 | 7372 | 10834 | 2287 | 10686 | 6393 | 10760 | 2916 | chr11:106804719-106815615 | 1415860\_at |
| Krt1-18 | View | View |  | 1607 | 2.81 | 4.72 | 1.90 | 2.63 |  |  | KERATIN COMPLEX 1, ACIDIC, GENE 18 | 2676 | 953 | 3522 | 746 | 2676 | 1409 | 3099 | 1179 | chr15:101856259-101860055 | 1448169\_at |
| Laptm4a | View | View |  | - | - | - | 0.27 | - |  |  | LYSOSOMAL-ASSOCIATED PROTEIN TRANSMEMBRANE 4A | 2257 | 1927 | 7532 | 8859 | 2257 | 8323 | 4895 | 7449 | chr12:8947672-8964735 | 1423368\_at |
| Laptm4b | View | View |  | - | - | 1.97 | - | - |  |  | LYSOSOMAL-ASSOCIATED PROTEIN TRANSMEMBRANE 4B | 13420 | 14672 | 13040 | 6622 | 13420 | 14144 | 13230 | 11219 | chr15:34182615-34228884 | 1416148\_at |
| Leng9 | View | View |  | - | - | - | 0.16 | - |  |  | LEUKOCYTE RECEPTOR CLUSTER (LRC) MEMBER 9 | 22 | 67 | 78 | 100 | 22 | 134 | 50 | 84 | chr7:3751305-3752762 | 1440191\_s\_at |
| Lgr5 | View | View |  | 2078 | 0.10 | 0.06 | 0.06 | 0.05 |  |  | LEUCINE RICH REPEAT CONTAINING G PROTEIN COUPLED RECEPTOR 5 | 55 | 545 | 116 | 1956 | 55 | 937 | 86 | 1655 | chr10:114855736-114991603 | 1441809\_at |
| Lin7c | View | View |  | 612 | - | - | 0.32 | - |  |  | LIN-7 HOMOLOG C (C. ELEGANS) | 332 | 245 | 1177 | 1083 | 332 | 1037 | 755 | 1198 | chr2:109691707-109701815 | 1449262\_s\_at |
| Llgl2 | View | View |  | - | - | 3.23 | 2.52 | 2.77 |  |  | LETHAL GIANT LARVAE HOMOLOG 2 (DROSOPHILA) | 1633 | 1542 | 1092 | 338 | 1633 | 649 | 1363 | 492 | chr11:115650918-115671348 | 1423938\_at |
| Lmnb2 | View | View |  | - | - | - | 0.19 | - |  |  | LAMIN B2 | 27 | 19 | 82 | 89 | 27 | 142 | 55 | 73 | chr10:80304493-80321362 | 1451849\_a\_at |
| Lmo1 | View | View |  | 772 | - | 1.50 | 5.58 | 2.80 |  |  | LIM DOMAIN ONLY 1 | 1065 | 1397 | 1495 | 998 | 1065 | 191 | 1280 | 457 | chr7:108930048-108934847 | 1418478\_at |
| LOC236220 | - | - |  | - | - | 0.11 | - | - |  |  | HYPOTHETICAL LOC236220 | 11 | 48 | 21 | 192 | 11 | 35 | 16 | 30 | chrNT\_164885:814-1330 | 1425220\_x\_at |
| Lrfn2 | View | View |  | 399 | 0.24 | - | 0.21 | - |  |  | LEUCINE RICH REPEAT AND FIBRONECTIN TYPE III DOMAIN CONTAINING 2 | 361 | 1480 | 282 | 255 | 361 | 1717 | 322 | 370 | chr17:48397946-48563155 | 1453126\_at |
| Lrrc2 | View | View |  | - | 1.44 | - | 6.34 | 11.71 |  |  | LEUCINE RICH REPEAT CONTAINING 2 | 1134 | 786 | 317 | 252 | 1134 | 179 | 726 | 62 | chr9:110796361-110828878 | 1453628\_s\_at, 1427388\_at |
| Lrrc22 | View | View |  | 237 | - | 3.77 | 1.48 | 3.04 |  |  | LEUCINE RICH REPEAT CONTAINING 22 | 3099 | 3210 | 2883 | 764 | 3099 | 2088 | 2991 | 983 | chr14:35979290-35984976 | 1443940\_at |
| Lrrc4 | View | View |  | 1340 | - | 0.28 | - | 0.28 |  |  | LEUCINE RICH REPEAT CONTAINING 4 | 305 | 306 | 340 | 1199 | 305 | 402 | 323 | 1135 | chr6:28779666-28781624 | 1416097\_at |
| Gene Symbol | Large Image Gene +/- 15kb | Closeup Image TSS +/- 15kb | Links | Max Score (threshold of 200) | *Nr2e3 -/-* | *Crx -/-* | *Nrl -/-* | *Crx;Nrl -/-* | In situ  hybridization | Electroporation | Full Name | Averaged Affy Scores | | | | | | | | Locus | Affy Mouse 430 2 |
|  |  |  |  |  |  |  |  |  |  |  |  | B6 | *Nr2e3 -/-* | 129 | *Crx -/-* | B6 | *Nrl -/-* | B6;129 | *Crx;Nrl -/-* |  |  |
| Lrtm1 | View | View |  | 875 | - | - | 0.27 | 0.49 |  |  | LEUCINE-RICH REPEATS AND TRANSMEMBRANE DOMAINS 1 | 789 | 601 | 2764 | 2601 | 789 | 2959 | 1777 | 3599 | chr14:27845265-27854891 | 1443866\_at |
| Maf | View | View |  | 827 | - | 0.15 | - | 0.32 |  |  | AVIAN MUSCULOAPONEUROTIC FIBROSARCOMA (V-MAF) AS42 ONCOGENE HOMOLOG | 83 | 54 | 73 | 481 | 83 | 83 | 78 | 245 | chr8:118568919-118592849 | 1437473\_at, 1456060\_at, 1447849\_s\_at |
| Malat1 | - | - |  | - | - | - | 0.21 | - |  |  | METASTASIS ASSOCIATED LUNG ADENOCARCINOMA TRANSCRIPT 1 (NON-CODING RNA) | 383 | 375 | 1436 | 1781 | 383 | 1795 | 910 | 1728 | chr19 | 1427285\_s\_at |
| Mapk1 | View | View |  | 458 | - | - | 0.14 | - |  |  | MITOGEN ACTIVATED PROTEIN KINASE 1 | 110 | 154 | 808 | 654 | 110 | 789 | 459 | 678 | chr16:16896945-16961015 | 1419568\_at |
| Mapk4 | View | View |  | - | - | - | 0.27 | - |  |  | MITOGEN-ACTIVATED PROTEIN KINASE 4 | 391 | 459 | 415 | 374 | 391 | 1442 | 403 | 661 | chr18:74053858-74190298 | 1435367\_at |
| Marveld3 | View | View |  | - | - | - | 7.98 | 6.61 |  |  | MARVEL (MEMBRANE-ASSOCIATING) DOMAIN CONTAINING 3 | 407 | 203 | 200 | 119 | 407 | 51 | 304 | 46 | chr8:112837034-112851301 | 1453264\_at |
| Mast4 | View | View |  | 357 | - | 0.20 | - | - |  |  | MICROTUBULE ASSOCIATED SERINE/THREONINE KINASE FAMILY MEMBER 4 | 112 | 130 | 120 | 605 | 112 | 181 | 116 | 206 | chr13:103852873-104454843 | 1445866\_at |
| Mc1r | View | View |  | 331 | - | 19.14 | - | - |  |  | MELANOCORTIN 1 RECEPTOR | 105 | 86 | 134 | 7 | 105 | 11 | 120 | 11 | chr8:126293586-126294846 | 1422069\_at |
| Mcc | View | View |  | 264 | - | 0.21 | 0.32 | 0.19 |  |  | MUTATED IN COLORECTAL CANCERS | 315 | 384 | 400 | 1881 | 315 | 980 | 358 | 1890 | chr18:44555218-44937494 | 1438081\_at |
| Mdm1 | View | View |  | 290 | - | 3.75 | - | 2.69 |  |  | TRANSFORMED MOUSE 3T3 CELL DOUBLE MINUTE 1 | 2469 | 2087 | 3788 | 1011 | 2469 | 1984 | 3129 | 1162 | chr10:117532014-117571990 | 1451053\_a\_at |
| Mef2c | View | View |  | 1489 | - | 10.74 | 5.63 | 9.86 |  |  | MYOCYTE ENHANCER FACTOR 2C | 675 | 613 | 1138 | 106 | 675 | 120 | 907 | 92 | chr13:83981532-84141286 | 1421027\_a\_at, 1421028\_a\_at, 1424852\_at, 1439946\_at, 1451507\_at |
| Mfng | View | View |  | - | 0.43 | - | 0.18 | 0.22 |  |  | MANIC FRINGE HOMOLOG (DROSOPHILA) | 220 | 516 | 368 | 377 | 220 | 1204 | 294 | 1309 | chr15:78583137-78600713 | 1416992\_at |
| Mknk1 | View | View |  | - | 1.94 | 1.40 | 3.04 | 1.99 |  |  | MAP KINASE-INTERACTING SERINE/THREONINE KINASE 1 | 1298 | 668 | 873 | 624 | 1298 | 427 | 1086 | 546 | chr4:115337178-115377180 | 1417630\_at |
| Mlf1 | View | View |  | 1262 | - | - | 5.08 | 7.93 |  |  | MYELOID LEUKEMIA FACTOR 1 | 376 | 308 | 543 | 617 | 376 | 74 | 460 | 58 | chr3:67462026-67487932 | 1418589\_a\_at |
| Moxd1 | View | View |  | 615 | 0.50 | - | 0.18 | - |  |  | MONOOXYGENASE, DBH-LIKE 1 | 336 | 668 | 297 | 305 | 336 | 1818 | 317 | 342 | chr10:23912933-23992199 | 1422643\_at |
| Mpp4 | View | View |  | - | - | 2.34 | - | 3.64 |  |  | MEMBRANE PROTEIN, PALMITOYLATED 4 (MAGUK P55 SUBFAMILY MEMBER 4) | 15563 | 14536 | 14219 | 6073 | 15563 | 14817 | 14891 | 4087 | chr1:59065489-59181765 | 1460368\_at |
| Mpp6 | View | View |  | 1177 | - | - | 0.26 | - |  |  | MEMBRANE PROTEIN, PALMITOYLATED 6 (MAGUK P55 SUBFAMILY MEMBER 6) | 439 | 465 | 354 | 547 | 439 | 1669 | 397 | 687 | chr6:50039835-50128173 | 1441546\_at, 1440396\_at, 1449348\_at |
| Msl2l1 | View | View |  | 213 | - | - | 0.19 | - |  |  | MALE-SPECIFIC LETHAL 2-LIKE 1 (DROSOPHILA) | 218 | 236 | 1155 | 1258 | 218 | 1129 | 687 | 1069 | chr9:100887852-100958425 | 1429109\_at |
| Mt1 | View | View |  | 420 | 0.63 | 0.42 | - | 1.90 |  |  | METALLOTHIONEIN 1 | 3653 | 5757 | 2716 | 6398 | 3653 | 4704 | 3185 | 1677 | chr8:97068356-97069453 | 1422557\_s\_at |
| Mt2 | View | View |  | 237 | 0.56 | 0.31 | - | 2.09 |  |  | METALLOTHIONEIN 2 | 1834 | 3290 | 1354 | 4308 | 1834 | 2503 | 1594 | 764 | chr8:97061913-97062691 | 1428942\_at |
| Mtac2d1 | View | View |  | 850 | - | - | 0.04 | - |  |  | MEMBRANE TARGETING (TANDEM) C2 DOMAIN CONTAINING 1 | 12 | 49 | 12 | 20 | 12 | 278 | 12 | 11 | chr12:102050117-102110540 | 1443933\_at |
| Mtap2 | View | View |  | 312 | - | 1.46 | 0.31 | - |  |  | MICROTUBULE-ASSOCIATED PROTEIN 2 | 655 | 739 | 1723 | 1183 | 655 | 2123 | 1189 | 1441 | chr1:66184870-66375789 | 1421328\_at, 1421327\_at |
| Mtap6 | View | View |  | 563 | - | - | 0.30 | 0.40 |  |  | MICROTUBULE-ASSOCIATED PROTEIN 6 | 1178 | 1549 | 991 | 1939 | 1178 | 3935 | 1085 | 2714 | chr7:99141577-99211219 | 1422550\_a\_at |
| Mtpn | View | View |  | - | - | - | 0.17 | - |  |  | MYOTROPHIN | 141 | 98 | 571 | 661 | 141 | 821 | 356 | 753 | chr6:35439058-35469939 | 1420472\_at |
| Mxra7 | View | View |  | - | - | 0.33 | - | - |  |  | MATRIX-REMODELLING ASSOCIATED 7 | 1209 | 1682 | 622 | 1866 | 1209 | 1577 | 916 | 1089 | chr11:116619494-116644136 | 1447509\_at, 1440975\_at |
| Mylk | View | View |  | 620 | 0.44 | 3.46 | 0.50 | - |  |  | MYOSIN, LIGHT POLYPEPTIDE KINASE | 2346 | 5320 | 2732 | 789 | 2346 | 4719 | 2539 | 1247 | chr16:34704668-34920340 | 1425506\_at, 1425505\_at |
| Myo7a | View | View |  | 1014 | 1.86 | - | 1.54 | - |  | Link | MYOSIN VIIA | 1090 | 586 | 73 | 44 | 1090 | 708 | 582 | 258 | chr7:97926593-97984444 | 1421385\_a\_at |
| Myocd | View | View |  | 280 | - | 0.11 | 0.04 | 0.05 |  |  | MYOCARDIN | 11 | 22 | 21 | 191 | 11 | 275 | 16 | 350 | chr11:64992756-65021661 | 1425808\_a\_at, 1425978\_at |
| Nab2 | View | View |  | 277 | - | 0.23 | - | 0.37 |  |  | NGFI-A BINDING PROTEIN 2 | 275 | 390 | 292 | 1258 | 275 | 201 | 284 | 774 | chr10:127063868-127069627 | 1417930\_at |
| Napb | View | View |  | - | 0.70 | - | 0.30 | - |  |  | N-ETHYLMALEIMIDE SENSITIVE FUSION PROTEIN ATTACHMENT PROTEIN BETA | 1071 | 1525 | 4071 | 3227 | 1071 | 3531 | 2571 | 3174 | chr2:148386098-148423861 | 1452444\_at, 1427470\_s\_at |
| Nat1 | View | View |  | 249 | 0.03 | - | - | - |  |  | N-ACETYLTRANSFERASE 1 (ARYLAMINE N-ACETYLTRANSFERASE) | 49 | 1431 | 133 | 33 | 49 | 105 | 91 | 17 | chr8:70419747-70421093 | 1421758\_at |
| Ncam1 | View | View |  | 609 | 46.18 | - | 2.06 | 2.43 |  |  | NEURAL CELL ADHESION MOLECULE 1 | 508 | 11 | 351 | 233 | 508 | 247 | 430 | 177 | chr9:49257298-49322170 | 1442680\_at |
| Ndn | View | View |  | - | - | - | 0.44 | - |  |  | NECDIN | 3123 | 3326 | 6527 | 6749 | 3123 | 7132 | 4825 | 6379 | chr7:62227270-62228875 | 1438625\_s\_at |
| Ndrg1 | View | View |  | 294 | - | 1.57 | 2.71 | 1.72 |  |  | N-MYC DOWNSTREAM REGULATED GENE 1 | 5940 | 4450 | 5066 | 3225 | 5940 | 2190 | 5503 | 3195 | chr15:66758991-66799308 | 1456174\_x\_at, 1420760\_s\_at |
| Neurod1 | View | View |  | 1423 | - | - | 0.38 | 0.57 | Link |  | NEUROGENIC DIFFERENTIATION 1 | 8168 | 6999 | 14102 | 21777 | 8168 | 21398 | 11135 | 19677 | chr2:79253480-79257479 | 1426413\_at |
| Nfasc | View | View |  | 309 | - | - | 3.33 | 2.47 |  |  | NEUROFASCIN | 3395 | 2815 | 2878 | 2374 | 3395 | 1020 | 3137 | 1268 | chr1:134397537-134569303 | 1436205\_at, 1459357\_at |
| Nfkb1 | View | View |  | 350 | - | 0.33 | 0.18 | 0.39 |  |  | NUCLEAR FACTOR OF KAPPA LIGHT CHAIN GENE ENHANCER IN B-CELLS 1, P105 | 346 | 400 | 465 | 1402 | 346 | 1911 | 406 | 1047 | chr3:135522081-135628718 | 1427705\_a\_at |
| Nphp1 | View | View |  | - | - | - | - | - |  |  | NEPHRONOPHTHISIS 1 (JUVENILE) HOMOLOG (HUMAN) | 328 | 376 | 284 | 201 | 328 | 301 | 306 | 297 | chr2:127432173-127480295 | 1416919\_a\_at |
| Npm3 | View | View |  | 1951 | - | - | 0.13 | - |  |  | NUCLEOPLASMIN 3 | 17 | 44 | 101 | 122 | 17 | 132 | 59 | 133 | chr19:45801047-45802874 | 1423522\_at |
| Nr2e3 | View | View |  | 1882 | 0.27 | - | 183.64 | 17.23 | Link | Link | NUCLEAR RECEPTOR SUBFAMILY 2, GROUP E, MEMBER 3 | 4040 | 15144 | 6158 | 5646 | 4040 | 22 | 5099 | 296 | chr9:59740777-59748085 | 1423631\_at |
| Nr4a1 | View | View |  | - | - | - | 0.13 | 0.40 |  |  | NUCLEAR RECEPTOR SUBFAMILY 4, GROUP A, MEMBER 1 | 473 | 920 | 1399 | 1920 | 473 | 3576 | 936 | 2336 | chr15:101094887-101102826 | 1416505\_at |
| Nrl | View | View |  | 860 | - | 2.78 | 974.91 | 3083.70 | Link | Link | NEURAL RETINA LEUCINE ZIPPER GENE | 31197 | 29946 | 30476 | 10946 | 31197 | 32 | 30837 | 10 | chr14:54473589-54479052 | 1450946\_at |
| Nrxn3 | View | View |  | 456 | - | - | 0.12 | 0.32 |  |  | NEUREXIN III | 297 | 298 | 1146 | 1260 | 297 | 2383 | 722 | 2251 | chr12:89308664-90737434 | 1460101\_at |
| Nsg1 | View | View |  | 290 | - | - | 0.30 | - |  |  | NEURON SPECIFIC GENE FAMILY MEMBER 1 | 643 | 715 | 2134 | 2222 | 643 | 2131 | 1389 | 1818 | chr5:38425442-38447716 | 1423055\_at |
| Nsun3 | View | View |  | 455 | - | - | 0.19 | - |  |  | NOL1/NOP2/SUN DOMAIN FAMILY 3 | 30 | 62 | 132 | 159 | 30 | 159 | 81 | 198 | chr16:62674701-62745920 | 1444634\_at |
| Nt5e | View | View |  | - | - | 2.03 | 5.02 | 7.39 |  |  | 5' NUCLEOTIDASE, ECTO | 3711 | 3944 | 5212 | 2564 | 3711 | 739 | 4462 | 604 | chr9:88125533-88169982 | 1428547\_at |
| Nup62 | View | View |  | - | - | 2.17 | 2.18 | 2.69 |  |  | NUCLEOPORIN 62 | 6860 | 6158 | 8216 | 3786 | 6860 | 3152 | 7538 | 2805 | chr7:44684381-44698846 | 1447905\_x\_at |
| Nupr1 | View | View |  | - | 0.68 | 0.16 | - | - |  |  | NUCLEAR PROTEIN 1 | 204 | 299 | 144 | 885 | 204 | 271 | 174 | 103 | chr7:126414397-126416637 | 1419665\_a\_at |
| Obox6 | View | View |  | - | - | 0.06 | - | 0.07 |  |  | OOCYTE SPECIFIC HOMEOBOX 6 | 52 | 85 | 94 | 1472 | 52 | 90 | 73 | 1071 | chr7:14991997-14993469 | 1440257\_at |
| Olfm1 | View | View |  | 363 | 0.40 | 0.49 | 0.39 | 0.37 | Link |  | OLFACTOMEDIN 1 | 3769 | 9399 | 3443 | 7006 | 3769 | 9708 | 3606 | 9684 | chr2:28015102-28052745 | 1425784\_a\_at, 1455796\_x\_at |
| Gene Symbol | Large Image Gene +/- 15kb | Closeup Image TSS +/- 15kb | Links | Max Score (threshold of 200) | *Nr2e3 -/-* | *Crx -/-* | *Nrl -/-* | *Crx;Nrl -/-* | In situ  hybridization | Electroporation | Full Name | Averaged Affy Scores | | | | | | | | Locus | Affy Mouse 430 2 |
|  |  |  |  |  |  |  |  |  |  |  |  | B6 | *Nr2e3 -/-* | 129 | *Crx -/-* | B6 | *Nrl -/-* | B6;129 | *Crx;Nrl -/-* |  |  |
| Opn1mw | View | View |  | 1119 | - | 494.33 | 0.63 | 72.63 | Link |  | OPSIN 1 (CONE PIGMENTS), MEDIUM-WAVE-SENSITIVE (COLOR BLINDNESS, DEUTAN) | 6269 | 7775 | 5932 | 12 | 6269 | 9928 | 6101 | 84 | chrX:70380184-70403476 | 1419723\_at |
| Opn1sw | View | View |  | 542 | 0.41 | 24.13 | 0.26 | - | Link |  | OPSIN 1 (CONE PIGMENTS), SHORT-WAVE-SENSITIVE (COLOR BLINDNESS, TRITAN) | 8727 | 21454 | 13153 | 545 | 8727 | 33451 | 10940 | 12900 | chr6:29326667-29330490 | 1449132\_at, 1418552\_at |
| Osgep | View | View |  | - | - | 2.81 | 0.27 | - | Link |  | O-SIALOGLYCOPROTEIN ENDOPEPTIDASE | 1946 | 1788 | 2550 | 908 | 1946 | 7236 | 2248 | 1598 | chr14:49828429-49846785 | 1418529\_at |
| Otop3 | View | View |  | 536 | 0.07 | - | 0.07 | 0.20 |  |  | OTOPETRIN 3 | 230 | 3429 | 225 | 178 | 230 | 3428 | 228 | 1113 | chr11:115150869-115163016 | 1429036\_at |
| Otx2 | View | View |  | 2011 | 0.74 | 0.48 | - | 0.46 | Link |  | ORTHODENTICLE HOMOLOG 2 (DROSOPHILA) | 6193 | 8398 | 4535 | 9484 | 6193 | 6905 | 5364 | 11720 | chr14:47580137-47589457 | 1425926\_a\_at |
| Pacsin2 | View | View |  | - | - | 2.01 | 1.80 | 2.80 |  |  | PROTEIN KINASE C AND CASEIN KINASE SUBSTRATE IN NEURONS 2 | 7853 | 7089 | 7305 | 3635 | 7853 | 4362 | 7579 | 2709 | chr15:83203373-83292336 | 1417810\_a\_at |
| Parvb | View | View |  | - | 0.65 | - | 0.20 | - |  |  | PARVIN, BETA | 531 | 822 | 372 | 460 | 531 | 2683 | 452 | 676 | chr15:84059809-84140797 | 1438672\_at |
| Parvg | View | View |  | - | - | - | 0.15 | - |  |  | PARVIN, GAMMA | 23 | 89 | 49 | 21 | 23 | 157 | 36 | 29 | chr15:84152486-84170705 | 1416875\_at |
| Pbx3 | View | View |  | 2018 | 0.69 | 0.72 | 0.50 | 0.39 |  |  | PRE B-CELL LEUKEMIA TRANSCRIPTION FACTOR 3 | 2720 | 3949 | 2434 | 3368 | 2720 | 5444 | 2577 | 6560 | chr2:33993768-34193945 | 1447640\_s\_at |
| Pcdh15 | View | View |  | 372 | 0.53 | - | 0.18 | 0.23 | Link |  | PROTOCADHERIN 15 | 807 | 1528 | 1266 | 1933 | 807 | 4590 | 1037 | 4562 | chr10:73342356-74089930 | 1444317\_at |
| Pcolce | View | View |  | - | - | 0.20 | 3.75 | - |  |  | PROCOLLAGEN C-ENDOPEPTIDASE ENHANCER PROTEIN | 841 | 1011 | 188 | 951 | 841 | 224 | 515 | 270 | chr5:137834897-137841156 | 1437165\_a\_at |
| Pdap1 | View | View |  | 247 | - | - | 0.30 | - |  |  | PDGFA ASSOCIATED PROTEIN 1 | 366 | 355 | 1217 | 1275 | 366 | 1210 | 792 | 1473 | chr5:145383183-145390524 | 1434020\_at |
| Pde1c | View | View |  | 627 | - | - | 0.09 | - |  |  | PHOSPHODIESTERASE 1C | 12 | 41 | 41 | 52 | 12 | 134 | 27 | 92 | chr6:55999382-56291970 | 1429643\_a\_at |
| Pde6a | View | View |  | 429 | - | 3.39 | 5.48 | 42.90 | Link |  | PHOSPHODIESTERASE 6A, CGMP-SPECIFIC, ROD, ALPHA | 4287 | 4494 | 4034 | 1190 | 4287 | 783 | 4161 | 97 | chr18:61345868-61415119 | 1450415\_at |
| Pde6b | View | View |  | 929 | - | 1.90 | 44.67 | 2.93 |  |  | PHOSPHODIESTERASE 6B, CGMP, ROD RECEPTOR, BETA POLYPEPTIDE | 26492 | 27523 | 25086 | 13175 | 26492 | 593 | 25789 | 8796 | chr5:108628694-108672034 | 1419740\_at |
| Pde6c | View | View |  | 264 | 0.14 | - | 0.13 | 0.11 | Link |  | PHOSPHODIESTERASE 6C, CGMP SPECIFIC, CONE, ALPHA PRIME | 2386 | 17230 | 826 | 1176 | 2386 | 17939 | 1606 | 14126 | chr19:38198090-38248883 | 1450830\_a\_at |
| Pde6g | View | View |  | - | - | 5.03 | 1.75 | 3.34 | Link |  | PHOSPHODIESTERASE 6G, CGMP-SPECIFIC, ROD, GAMMA | 33458 | 34101 | 28141 | 5597 | 33458 | 19145 | 30800 | 9233 | chr11:120263700-120269573 | 1425100\_a\_at, 1450453\_a\_at |
| Pde6h | View | View |  | - | 0.30 | 21.40 | 0.28 | 0.37 | Link |  | PHOSPHODIESTERASE 6H, CGMP-SPECIFIC, CONE, GAMMA | 7915 | 26118 | 6185 | 289 | 7915 | 28151 | 7050 | 18811 | chr6:136918719-136933052 | 1450766\_at, 1450765\_a\_at |
| Pdha1 | View | View |  | - | 0.63 | - | 0.33 | - |  |  | PYRUVATE DEHYDROGENASE E1 ALPHA 1 | 582 | 928 | 1212 | 1250 | 582 | 1756 | 897 | 1270 | chrX:155466324-155482441 | 1449137\_at |
| Pdia3 | View | View |  | 441 | - | 0.78 | 0.06 | - |  |  | PROTEIN DISULFIDE ISOMERASE ASSOCIATED 3 | 276 | 311 | 3888 | 4985 | 276 | 4843 | 2082 | 4633 | chr2:121105386-121129419 | 1423423\_at |
| Pdia5 | View | View |  | 212 | 1.28 | 13.21 | 8.72 | 10.39 |  |  | PROTEIN DISULFIDE ISOMERASE ASSOCIATED 5 | 2293 | 1795 | 2048 | 155 | 2293 | 263 | 2171 | 209 | chr16:35317205-35386404 | 1424650\_at |
| Pex2 | View | View |  | 605 | - | 2.08 | 0.12 | - |  |  | PEROXIN 2 | 374 | 210 | 3989 | 1917 | 374 | 3038 | 2182 | 1887 | chr3:33142161-33274578 | 1438399\_at |
| Pfkfb2 | View | View |  | 552 | - | 5.20 | 3.23 | 4.34 |  |  | 6-PHOSPHOFRUCTO-2-KINASE/FRUCTOSE-2,6-BIPHOSPHATASE 2 | 252 | 155 | 260 | 50 | 252 | 78 | 256 | 59 | chr1:132516728-132543360 | 1431901\_a\_at, 1422090\_a\_at, 1429486\_at, 1422091\_at |
| Pfkl | View | View |  | - | 1.44 | 1.40 | 1.97 | 2.16 |  |  | PHOSPHOFRUCTOKINASE, LIVER, B-TYPE | 5916 | 4109 | 3836 | 2747 | 5916 | 3006 | 4876 | 2253 | chr10:77390400-77412878 | 1450269\_a\_at, 1439148\_a\_at |
| Pftk1 | View | View |  | 642 | - | - | 0.19 | - |  |  | PFTAIRE PROTEIN KINASE 1 | 56 | 30 | 388 | 414 | 56 | 301 | 222 | 390 | chr5:4809739-5386209 | 1453956\_a\_at |
| Phf20 | View | View |  | - | - | - | 0.32 | - |  |  | PHD FINGER PROTEIN 20 | 362 | 434 | 1355 | 1678 | 362 | 1145 | 859 | 1371 | chr2:155888172-155998877 | 1452259\_at |
| Phr1 | View | View |  | - | - | - | 0.18 | - |  |  | PAM, HIGHWIRE, RPM 1 | 101 | 100 | 472 | 527 | 101 | 548 | 287 | 540 | chr14:101999559-102232127 | 1456715\_at |
| Pik3ap1 | View | View |  | - | - | - | 0.11 | - |  |  | PHOSPHOINOSITIDE-3-KINASE ADAPTOR PROTEIN 1 | 21 | 144 | 13 | 19 | 21 | 199 | 17 | 97 | chr19:41327607-41429589 | 1421285\_at, 1429831\_at |
| Pik3r1 | View | View |  | 845 | - | - | 0.31 | - |  |  | PHOSPHATIDYLINOSITOL 3-KINASE, REGULATORY SUBUNIT, POLYPEPTIDE 1 (P85 ALPHA) | 401 | 501 | 1869 | 2307 | 401 | 1291 | 1135 | 1509 | chr13:102781018-102868441 | 1438682\_at |
| Pip5k1b | View | View |  | 228 | - | - | 0.15 | - |  |  | PHOSPHATIDYLINOSITOL-4-PHOSPHATE 5-KINASE, TYPE 1 BETA | 166 | 223 | 858 | 929 | 166 | 1081 | 512 | 965 | chr3:95145888-95191855 | 1455191\_x\_at, 1435039\_a\_at, 1426009\_a\_at |
| Pitpnc1 | View | View |  | - | - | - | 0.19 | - |  |  | PHOSPHATIDYLINOSITOL TRANSFER PROTEIN, CYTOPLASMIC 1 | 38 | 15 | 319 | 199 | 38 | 196 | 179 | 285 | chr11:107023982-107286810 | 1428879\_at |
| Pitpnm3 | View | View |  | - | - | 2.15 | 7.56 | 4.74 |  |  | PITPNM FAMILY MEMBER 3 | 3971 | 3784 | 3752 | 1744 | 3971 | 525 | 3862 | 814 | chr11:71863723-71951973 | 1455140\_at, 1458956\_at |
| Pkp4 | View | View |  | 681 | - | - | 0.34 | - |  |  | PLAKOPHILIN 4 | 372 | 355 | 864 | 1133 | 372 | 1110 | 618 | 1044 | chr2:59015522-59156047 | 1438677\_at |
| Plec1 | View | View |  | - | 0.70 | 0.28 | 0.36 | 0.25 |  |  | PLECTIN 1 | 575 | 817 | 555 | 1957 | 575 | 1577 | 565 | 2256 | chr15:75998231-76058633 | 1419835\_s\_at, 1434610\_at |
| Plekha2 | View | View |  | - | 2.47 | - | 5.52 | 4.04 |  |  | PLECKSTRIN HOMOLOGY DOMAIN-CONTAINING, FAMILY A (PHOSPHOINOSITIDE BINDING SPECIFIC) MEMBER 2 | 685 | 277 | 341 | 510 | 685 | 124 | 513 | 127 | chr8:26506385-26567739 | 1436177\_at, 1417288\_at |
| Plekha8 | View | View |  | 374 | 1.37 | 3.02 | - | 2.63 |  |  | PLECKSTRIN HOMOLOGY DOMAIN CONTAINING, FAMILY A (PHOSPHOINOSITIDE BINDING SPECIFIC) MEMBER 8 | 2495 | 1816 | 3326 | 1101 | 2495 | 2264 | 2911 | 1105 | chr6:54524689-54572670 | 1454819\_at |
| Plekhf2 | View | View |  | 471 | - | 5.33 | 2.34 | 5.00 |  |  | PLECKSTRIN HOMOLOGY DOMAIN CONTAINING, FAMILY F (WITH FYVE DOMAIN) MEMBER 2 | 636 | 528 | 1513 | 284 | 636 | 272 | 1075 | 215 | chr4:10915810-10934717 | 1423862\_at |
| Plekhh2 | View | View |  | 1135 | - | - | 0.10 | - |  |  | PLECKSTRIN HOMOLOGY DOMAIN CONTAINING, FAMILY H (WITH MYTH4 DOMAIN) MEMBER 2 | 14 | 20 | 34 | 22 | 14 | 135 | 24 | 45 | chr17:84420221-84530455 | 1427597\_at |
| Pnn | View | View |  | 1243 | - | - | 0.22 | - |  |  | PININ | 328 | 289 | 1238 | 1227 | 328 | 1474 | 783 | 1226 | chr12:59985004-59991917 | 1423325\_at |
| Pnp | View | View |  | 289 | - | - | 0.21 | 0.27 |  |  | PURINE-NUCLEOSIDE PHOSPHORYLASE | 996 | 1043 | 1497 | 2094 | 996 | 4669 | 1247 | 4640 | chr14:49777557-49875363 | 1416530\_a\_at, 1453299\_a\_at |
| Podxl | View | View |  | 475 | - | - | 0.20 | - |  |  | PODOCALYXIN-LIKE | 78 | 68 | 254 | 242 | 78 | 388 | 166 | 258 | chr6:31449656-31494074 | 1417396\_at |
| Polg2 | View | View |  | 557 | 2.28 | 4.23 | 8.16 | 7.30 |  |  | POLYMERASE (DNA DIRECTED), GAMMA 2, ACCESSORY SUBUNIT | 3379 | 1484 | 2231 | 527 | 3379 | 414 | 2805 | 384 | chr11:106584396-106595532 | 1450816\_at |
| Ppap2c | View | View |  | - | 1.87 | 5.65 | 14.29 | 7.55 | Link |  | PHOSPHATIDIC ACID PHOSPHATASE TYPE 2C | 3557 | 1906 | 4020 | 711 | 3557 | 249 | 3789 | 502 | chr10:78929564-78936892 | 1451210\_at |
| Ppargc1b | View | View |  | 351 | - | 1.82 | 5.07 | 3.69 |  |  | PEROXISOME PROLIFERATIVE ACTIVATED RECEPTOR, GAMMA, COACTIVATOR 1 BETA | 3952 | 3616 | 2350 | 1292 | 3952 | 780 | 3151 | 853 | chr18:61423505-61525800 | 1449945\_at |
| Ppm1j | View | View |  | 248 | - | 0.34 | 0.24 | 0.22 |  |  | PROTEIN PHOSPHATASE 1J | 522 | 607 | 481 | 1433 | 522 | 2218 | 502 | 2328 | chr3:104909112-104914004 | 1453127\_at |
| Ppp2r5e | View | View |  | 282 | 2.63 | - | 3.86 | - |  |  | PROTEIN PHOSPHATASE 2, REGULATORY SUBUNIT B (B56), EPSILON ISOFORM | 1105 | 420 | 390 | 270 | 1105 | 286 | 748 | 385 | chr12:76369721-76515040 | 1443533\_at |
| Prcd | View | View |  | 253 | - | 5.96 | 1.69 | 3.02 |  | Link | PROGRESSIVE ROD-CONE DEGENERATION | 9351 | 9679 | 8813 | 1479 | 9351 | 5543 | 9082 | 3004 | chr11:116473308-116477593 | 1436657\_at |
| Prdm1 | View | View |  | 3732 | 0.44 | 0.47 | 0.32 | 0.19 |  |  | PR DOMAIN CONTAINING 1, WITH ZNF DOMAIN | 501 | 1136 | 524 | 1123 | 501 | 1563 | 513 | 2748 | chr10:44125592-44147163 | 1420425\_at |
| Prmt1 | View | View |  | 420 | - | - | 0.21 | - |  |  | PROTEIN ARGININE N-METHYLTRANSFERASE 1 | 810 | 734 | 3794 | 3961 | 810 | 3827 | 2302 | 3836 | chr7:44844800-44854398 | 1452787\_a\_at |
| Prmt8 | View | View |  | - | 0.52 | 0.50 | 0.33 | 0.30 |  |  | PROTEIN ARGININE N-METHYLTRANSFERASE 8 | 944 | 1808 | 942 | 1888 | 944 | 2819 | 943 | 3104 | chr6:127654626-127734366 | 1435204\_at |
| Gene Symbol | Large Image Gene +/- 15kb | Closeup Image TSS +/- 15kb | Links | Max Score (threshold of 200) | *Nr2e3 -/-* | *Crx -/-* | *Nrl -/-* | *Crx;Nrl -/-* | In situ  hybridization | Electroporation | Full Name | Averaged Affy Scores | | | | | | | | Locus | Affy Mouse 430 2 |
|  |  |  |  |  |  |  |  |  |  |  |  | B6 | *Nr2e3 -/-* | 129 | *Crx -/-* | B6 | *Nrl -/-* | B6;129 | *Crx;Nrl -/-* |  |  |
| Prom1 | View | View |  | - | - | 1.26 | 1.40 | 1.69 | Link |  | PROMININ 1 | 23602 | 21904 | 20115 | 15936 | 23602 | 16881 | 21859 | 12965 | chr5:44281869-44389964 | 1419700\_a\_at |
| Prpf19 | View | View |  | - | - | - | 0.17 | - |  |  | PRP19/PSO4 PRE-MRNA PROCESSING FACTOR 19 HOMOLOG (S. CEREVISIAE) | 243 | 258 | 960 | 1363 | 243 | 1397 | 602 | 1339 | chr19:10955201-10972575 | 1460633\_at |
| Prph1 | View | View |  | 437 | - | 0.19 | - | - |  |  | PERIPHERIN 1 | 108 | 196 | 102 | 533 | 108 | 116 | 105 | 70 | chr15:98883215-98887000 | 1422530\_at |
| Prtg | View | View |  | 691 | 0.54 | 0.36 | 0.28 | 0.44 |  |  | PROTOGENIN HOMOLOG (GALLUS GALLUS) | 397 | 735 | 185 | 512 | 397 | 1414 | 291 | 661 | chr9:72606024-72710423 | 1456329\_at |
| Psap | View | View |  | - | - | - | 0.44 | - |  |  | PROSAPOSIN | 3369 | 3385 | 6099 | 6249 | 3369 | 7642 | 4734 | 6361 | chr10:59673071-59697954 | 1421813\_a\_at |
| Ptgds | View | View |  | - | 0.44 | 2.51 | - | - |  |  | PROSTAGLANDIN D2 SYNTHASE (BRAIN) | 4007 | 9182 | 7382 | 2936 | 4007 | 4234 | 5695 | 3914 | chr2:25288721-25291758 | 1423859\_a\_at |
| Ptp4a3 | View | View |  | 446 | - | 0.62 | 2.11 | - |  |  | PROTEIN TYROSINE PHOSPHATASE 4A3 | 8545 | 9382 | 5102 | 8225 | 8545 | 4049 | 6824 | 5468 | chr15:73552053-73584482 | 1418181\_at |
| Ptplad1 | View | View |  | - | - | - | 0.13 | - |  |  | PROTEIN TYROSINE PHOSPHATASE-LIKE A DOMAIN CONTAINING 1 | 310 | 324 | 1621 | 1593 | 310 | 2445 | 966 | 1549 | chr9:64784992-64819719 | 1452427\_s\_at |
| Pygm | View | View |  | 663 | 0.15 | - | 0.39 | 0.19 |  |  | MUSCLE GLYCOGEN PHOSPHORYLASE | 1055 | 7204 | 799 | 1470 | 1055 | 2717 | 927 | 4887 | chr19:6384429-6398459 | 1448602\_at |
| Q3URA8\_MOUSE | View | View |  | 300 | - | - | 0.18 | 0.33 |  |  | HYPOTHETICAL PROTEIN 6330405H19 | 213 | 358 | 252 | 337 | 213 | 1162 | 233 | 714 | chr5:108611116-108612694 | 1436265\_at |
| Q8CBC5\_MOUSE | View | View |  | - | - | - | 0.19 | - |  |  | HYPOTHETICAL GENE SUPPORTED BY AK036329 | 49 | 44 | 37 | 71 | 49 | 257 | 43 | 131 | chr18:74190471-74190953 | 1446217\_at |
| Qscn6 | View | View |  | - | - | - | 0.11 | 0.24 |  |  | QUIESCIN Q6 | 183 | 368 | 294 | 398 | 183 | 1709 | 239 | 985 | chr1:157540373-157575117 | 1420831\_at |
| Rab18 | View | View |  | - | - | - | 0.31 | - |  |  | RAB18, MEMBER RAS ONCOGENE FAMILY | 623 | 510 | 2002 | 2238 | 623 | 2025 | 1313 | 2402 | chr18:6765203-6790229 | 1420899\_at |
| Rabgef1 | View | View |  | - | - | 4.50 | 1.69 | 3.11 |  |  | RAB GUANINE NUCLEOTIDE EXCHANGE FACTOR (GEF) 1 | 4573 | 3800 | 5458 | 1213 | 4573 | 2707 | 5016 | 1613 | chr5:130471883-130499040 | 1419067\_a\_at, 1419069\_at, 1419068\_at |
| Rasal2 | View | View |  | 519 | - | - | 0.20 | - |  |  | RAS PROTEIN ACTIVATOR LIKE 2 | 24 | 33 | 104 | 86 | 24 | 122 | 64 | 94 | chr1:158977391-159248561 | 1444671\_at |
| Rasgrf2 | View | View |  | 456 | - | - | 0.04 | - |  |  | RAS PROTEIN-SPECIFIC GUANINE NUCLEOTIDE-RELEASING FACTOR 2 | 13 | 31 | 213 | 254 | 13 | 289 | 113 | 354 | chr13:92358674-92462287 | 1421621\_at |
| Rbp4 | View | View |  | 264 | - | 0.03 | - | 0.14 |  |  | RETINOL BINDING PROTEIN 4, PLASMA | 90 | 138 | 150 | 5259 | 90 | 155 | 120 | 833 | chr19:38181773-38190392 | 1426225\_at |
| Rcvrn | View | View |  | 2128 | - | 17.55 | 1.52 | 3.11 | Link |  | RECOVERIN | 24722 | 27261 | 22206 | 1265 | 24722 | 16247 | 23464 | 7542 | chr11:67511521-67519528 | 1450215\_at |
| Rdh12 | View | View |  | 492 | 1.39 | 12.42 | 2.78 | 8.40 | Link | Link | RETINOL DEHYDROGENASE 12 | 12548 | 9040 | 11861 | 955 | 12548 | 4517 | 12205 | 1453 | chr12:80127754-80141501 | 1424256\_at, 1431010\_a\_at |
| Rdh5 | View | View |  | 445 | 0.47 | 2.98 | - | - |  |  | RETINOL DEHYDROGENASE 5 | 541 | 1154 | 1191 | 400 | 541 | 659 | 866 | 399 | chr10:128316537-128322222 | 1418808\_at |
| Rds | View | View |  | 449 | - | 6.88 | 0.41 | - |  | Link | RETINAL DEGENERATION, SLOW (RETINITIS PIGMENTOSA 7) | 177 | 107 | 2086 | 303 | 177 | 427 | 1132 | 342 | chr17:46373676-46388117 | 1420511\_at |
| Reep6 | View | View |  | - | - | 34.01 | 16.93 | 40.04 | Link |  | RECEPTOR ACCESSORY PROTEIN 6 | 19573 | 14211 | 15100 | 444 | 19573 | 1156 | 17337 | 433 | chr10:79733360-79737930 | 1430128\_a\_at |
| Rgl3 | View | View |  | - | - | 0.25 | 0.13 | - |  |  | RAL GUANINE NUCLEOTIDE DISSOCIATION STIMULATOR-LIKE 3 | 219 | 356 | 281 | 1126 | 219 | 1738 | 250 | 1765 | chr9:21721929-21739855 | 1431829\_a\_at |
| Rgr | View | View |  | 237 | 0.33 | 3.91 | - | - |  |  | RETINAL G PROTEIN COUPLED RECEPTOR | 542 | 1666 | 2046 | 523 | 542 | 925 | 1294 | 501 | chr14:35948731-35960205 | 1422832\_at |
| Rgs20 | View | View |  | - | - | 3.32 | - | - |  |  | REGULATOR OF G-PROTEIN SIGNALING 20 | 1407 | 1031 | 1487 | 448 | 1407 | 1350 | 1447 | 1087 | chr1:4900326-5009507 | 1443694\_at |
| Rgs9 | View | View |  | 445 | - | - | - | - | Link | Link | REGULATOR OF G-PROTEIN SIGNALING 9 | 277 | 224 | 244 | 236 | 277 | 289 | 261 | 202 | chr11:109041445-109114219 | 1418691\_at |
| Rgs9bp | View | View |  | 622 | - | 4.09 | 1.75 | 2.99 | Link | Link | REGULATOR OF G-PROTEIN SIGNALLING 9 BINDING PROTEIN | 9348 | 8361 | 8748 | 2139 | 9348 | 5354 | 9048 | 3023 | chr7:35293267-35293980 | 1440256\_at |
| Rhbdf2 | View | View |  | - | - | 0.11 | - | - |  |  | RHOMBOID, VEINLET-LIKE 6 (DROSOPHILA) | 264 | 91 | 17 | 149 | 264 | 216 | 141 | 70 | chr11:116414259-116443109 | 1434774\_at |
| Rho | View | View |  | 1044 | - | 2.77 | 626.03 | 686.31 | Link | Link | RHODOPSIN | 40692 | 40160 | 38920 | 14064 | 40692 | 65 | 39806 | 58 | chr6:115897546-115904449 | 1425172\_at, 1451617\_at, 1425171\_at, 1451618\_at |
| Rian | View | View |  | 279 | - | - | 0.11 | - |  |  | RNA IMPRINTED AND ACCUMULATED IN NUCLEUS | 232 | 258 | 2776 | 2973 | 232 | 2198 | 1504 | 2168 | chr12:110093969-110100035 | 1452899\_at |
| Ric8b | View | View |  | 1694 | 2.15 | 3.84 | 7.31 | 6.72 |  |  | RESISTANCE TO INHIBITORS OF CHOLINESTERASE 8 HOMOLOG B (C. ELEGANS) | 5397 | 2512 | 4017 | 1047 | 5397 | 738 | 4707 | 700 | chr10:84347415-84446246 | 1435096\_at |
| Rims1 | View | View |  | 543 | - | - | 1.54 | - |  |  | REGULATING SYNAPTIC MEMBRANE EXOCYTOSIS 1 | 3224 | 3900 | 2863 | 2765 | 3224 | 2096 | 3044 | 2524 | chr1:22271402-22759999 | 1435667\_at |
| Ris2 | View | View |  | - | 0.20 | - | 0.32 | - |  |  | RETROVIRAL INTEGRATION SITE 2 | 133 | 673 | 116 | 200 | 133 | 414 | 125 | 237 | chr8:125454116-125459644 | 1424144\_at, 1424143\_a\_at |
| Rnf14 | View | View |  | 1299 | - | - | 0.30 | - |  |  | RING FINGER PROTEIN 14 | 499 | 393 | 1710 | 1837 | 499 | 1688 | 1105 | 1943 | chr18:38422658-38443821 | 1431030\_a\_at |
| Rnf149 | View | View |  | - | - | - | 0.33 | 0.52 |  |  | RING FINGER PROTEIN 149 | 414 | 697 | 1606 | 1848 | 414 | 1256 | 1010 | 1939 | chr1:39495847-39521951 | 1429321\_at |
| Rnf25 | View | View |  | 408 | - | - | 0.15 | - |  |  | RING FINGER PROTEIN 25 | 166 | 146 | 915 | 962 | 166 | 1085 | 541 | 972 | chr1:74526960-74534604 | 1448674\_at |
| Rom1 | View | View |  | 1095 | - | 1.96 | 3.19 | 2.21 |  | Link | ROD OUTER SEGMENT MEMBRANE PROTEIN 1 | 19815 | 17812 | 17650 | 8994 | 19815 | 6218 | 18733 | 8492 | chr19:8994443-8996401 | 1448996\_at |
| Rp1h | View | View |  | 830 | - | 2.63 | 2.97 | 3.41 |  | Link | RETINITIS PIGMENTOSA 1 HOMOLOG (HUMAN) | 12129 | 11175 | 11985 | 4556 | 12129 | 4081 | 12057 | 3532 | chr1:4334224-4350473 | 1424963\_at |
| Rpe65 | View | View |  | 485 | 0.28 | 5.01 | 0.47 | - |  |  | RETINAL PIGMENT EPITHELIUM 65 | 124 | 437 | 972 | 194 | 124 | 262 | 548 | 110 | chr3:159535602-159560092 | 1450197\_at |
| Rpgr | View | View |  | 298 | - | - | 0.61 | - |  |  | RETINITIS PIGMENTOSA GTPASE REGULATOR | 674 | 637 | 856 | 904 | 674 | 1099 | 765 | 1113 | chrX:9194936-9373697 | 1427467\_a\_at |
| Rpgrip1 | View | View |  | 596 | - | 5.12 | 0.39 | - | Link | Link | RETINITIS PIGMENTOSA GTPASE REGULATOR INTERACTING PROTEIN 1 | 1422 | 1551 | 2736 | 534 | 1422 | 3602 | 2079 | 1136 | chr14:51032873-51082881 | 1451785\_at, 1421144\_at, 1454231\_a\_at, 1431357\_a\_at |
| Rps6ka2 | View | View |  | - | - | - | 2.48 | 2.11 |  |  | RIBOSOMAL PROTEIN S6 KINASE, RELATED SEQUENCE 1 | 5835 | 6780 | 3892 | 3346 | 5835 | 2351 | 4864 | 2301 | chr17:7019773-7152975 | 1417542\_at |
| Rs1h | View | View |  | 287 | - | 16.42 | 0.26 | - | Link | Link | RETINOSCHISIS 1 HOMOLOG (HUMAN) | 579 | 451 | 3925 | 239 | 579 | 2214 | 2252 | 504 | chrX:156112118-156143768 | 1421085\_at, 1421084\_at |
| Rtbdn | View | View |  | - | - | 12.72 | - | 10.14 | Link |  | RETBINDIN | 15475 | 17630 | 11324 | 890 | 15475 | 14707 | 13400 | 1321 | chr8:87837096-87846708 | 1451603\_at |
| Rtn1 | View | View |  | 342 | - | - | 2.08 | - |  |  | RETICULON 1 | 6607 | 7294 | 3848 | 4526 | 6607 | 3169 | 5228 | 3672 | chr12:73130586-73327777 | 1429761\_at |
| Rtn4 | View | View |  | 521 | - | - | 0.23 | - | Link |  | RETICULON 4 | 3003 | 2253 | 12304 | 11194 | 3003 | 13133 | 7654 | 12897 | chr11:29592955-29644255 | 1421116\_a\_at |
| Rufy3 | View | View |  | 889 | - | - | 0.29 | - |  |  | RUN AND FYVE DOMAIN CONTAINING 3 | 1714 | 1695 | 5743 | 6415 | 1714 | 5870 | 3729 | 4819 | chr5:89639710-89724536 | 1424402\_at |
| Rwdd4a | View | View |  | 250 | - | 2.19 | - | - |  |  | RWD DOMAIN CONTAINING 4A | 4102 | 3959 | 8075 | 3688 | 4102 | 3507 | 6089 | 3046 | chr8:49032509-49049544 | 1457983\_s\_at |
| Rxrg | View | View |  | 405 | - | 0.15 | 0.18 | 0.23 |  |  | RETINOID X RECEPTOR GAMMA | 182 | 329 | 204 | 1325 | 182 | 988 | 193 | 844 | chr1:169435059-169476298 | 1418782\_at |
| Sag | View | View |  | 325 | - | - | - | 1.26 | Link |  | RETINAL S-ANTIGEN | 35796 | 38136 | 35681 | 28028 | 35796 | 32205 | 35739 | 28426 | chr1:89634851-89676194 | 1419025\_at |
| Gene Symbol | Large Image Gene +/- 15kb | Closeup Image TSS +/- 15kb | Links | Max Score (threshold of 200) | *Nr2e3 -/-* | *Crx -/-* | *Nrl -/-* | *Crx;Nrl -/-* | In situ  hybridization | Electroporation | Full Name | Averaged Affy Scores | | | | | | | | Locus | Affy Mouse 430 2 |
|  |  |  |  |  |  |  |  |  |  |  |  | B6 | *Nr2e3 -/-* | 129 | *Crx -/-* | B6 | *Nrl -/-* | B6;129 | *Crx;Nrl -/-* |  |  |
| Samd7 | View | View |  | 1013 | - | 0.33 | - | 15.73 |  |  | STERILE ALPHA MOTIF DOMAIN CONTAINING 7 | 748 | 833 | 1832 | 5494 | 748 | 730 | 1290 | 82 | chr3:30937131-30958004 | 1455782\_at |
| Scamp1 | View | View |  | 355 | - | - | 0.32 | - |  |  | RIKEN CDNA 4930505M11 GENE | 482 | 418 | 1579 | 1531 | 482 | 1527 | 1031 | 1697 | chr13:95302187-95385980 | 1453054\_at |
| Scg3 | View | View |  | - | 0.70 | 0.72 | 0.44 | 0.44 |  |  | SECRETOGRANIN III | 5147 | 7321 | 5127 | 7142 | 5147 | 11604 | 5137 | 11553 | chr9:75429283-75469926 | 1448628\_at |
| Sec14l2 | View | View |  | 681 | 1.57 | 2.35 | 2.99 | 3.50 |  |  | SEC14-LIKE 2 (S. CEREVISIAE) | 1134 | 724 | 586 | 249 | 1134 | 379 | 860 | 246 | chr11:3997034-4018732 | 1424530\_at |
| Sec22c | View | View |  | - | - | - | 3.10 | 3.23 |  |  | SEC22 VESICLE TRAFFICKING PROTEIN-LIKE 3 (S. CEREVISIAE) | 2300 | 1995 | 893 | 620 | 2300 | 743 | 1597 | 495 | chr9:121531768-121553727 | 1436102\_at |
| Sema6d | View | View |  | 1094 | - | 0.27 | - | - |  |  | SEMA6D | 624 | 557 | 562 | 2088 | 624 | 500 | 593 | 729 | chr2:124301737-124359213 | 1453055\_at |
| Sema7a | View | View |  | 870 | - | - | 0.05 | - |  |  | SEMA DOMAIN, IMMUNOGLOBULIN DOMAIN (IG), AND GPI MEMBRANE ANCHOR, (SEMAPHORIN) 7A | 6 | 17 | 182 | 163 | 6 | 133 | 94 | 139 | chr9:57738272-57761002 | 1422040\_at |
| Serinc1 | View | View |  | - | - | - | 0.43 | - |  |  | SERINE INCORPORATOR 1 | 3193 | 2826 | 8453 | 8597 | 3193 | 7453 | 5823 | 8372 | chr10:57204197-57220919 | 1437513\_a\_at |
| Serpina3n | View | View |  | - | - | 0.21 | - | - |  |  | SERINE (OR CYSTEINE) PEPTIDASE INHIBITOR, CLADE A, MEMBER 3N | 116 | 230 | 475 | 2215 | 116 | 84 | 296 | 372 | chr12:104807779-104815378 | 1419100\_at |
| Serping1 | View | View |  | - | - | 0.17 | - | 0.32 |  |  | SERINE (OR CYSTEINE) PEPTIDASE INHIBITOR, CLADE G, MEMBER 1 | 425 | 437 | 204 | 1180 | 425 | 502 | 315 | 986 | chr2:84566224-84576243 | 1416625\_at |
| Sfrs1 | View | View |  | 916 | - | - | 0.46 | - |  |  | SPLICING FACTOR, ARGININE/SERINE-RICH 1 (ASF/SF2) | 2704 | 2333 | 5665 | 5700 | 2704 | 5819 | 4185 | 5668 | chr11:87863880-87867803 | 1452430\_s\_at |
| Sgcg | View | View |  | 358 | - | 262.50 | - | - |  |  | SARCOGLYCAN, GAMMA (DYSTROPHIN-ASSOCIATED GLYCOPROTEIN) | 9 | 18 | 525 | 2 | 9 | 1 | 267 | 11 | chr14:60175169-60212543 | 1437523\_s\_at, 1421254\_a\_at |
| Sh2d1a | View | View |  | 325 | 6.63 | 140.33 | 33.13 | 171.50 |  |  | SH2 DOMAIN PROTEIN 1A | 265 | 40 | 421 | 3 | 265 | 8 | 343 | 2 | chrX:38747189-38766724 | 1449393\_at |
| Six6os1 | View | View |  | 1081 | 0.69 | 0.60 | 0.22 | 0.27 |  |  | SIX6 OPPOSITE STRAND TRANSCRIPT 1 | 977 | 1412 | 1477 | 2455 | 977 | 4344 | 1227 | 4498 | chr12:73799947-73836603 | 1431393\_at, 1431896\_at |
| Slc16a6 | View | View |  | 358 | - | 2.91 | 6.77 | - |  |  | SOLUTE CARRIER FAMILY 16 (MONOCARBOXYLIC ACID TRANSPORTERS), MEMBER 6 | 1306 | 1312 | 422 | 145 | 1306 | 193 | 864 | 142 | chr11:109266945-109289686 | 1417884\_at |
| Slc17a1 | View | View |  | - | - | 13.40 | - | - |  |  | SOLUTE CARRIER FAMILY 17 (SODIUM PHOSPHATE), MEMBER 1 | 513 | 598 | 536 | 40 | 513 | 679 | 525 | 312 | chr13:23877797-23903195 | 1417280\_at |
| Slc17a7 | View | View |  | 311 | - | 2.61 | 1.50 | 1.77 |  |  | SOLUTE CARRIER FAMILY 17 (SODIUM-DEPENDENT INORGANIC PHOSPHATE COTRANSPORTER), MEMBER 7 | 9150 | 7766 | 6765 | 2594 | 9150 | 6085 | 7958 | 4496 | chr7:45032065-45043086 | 1428986\_at |
| Slc1a1 | View | View |  | 276 | - | - | 0.18 | - |  |  | SOLUTE CARRIER FAMILY 1 (NEURONAL/EPITHELIAL HIGH AFFINITY GLUTAMATE TRANSPORTE , SYSTEM XAG), MEMBER 1 [+/-]Show full name | 87 | 105 | 609 | 615 | 87 | 484 | 348 | 381 | chr19:28901132-28979956 | 1425415\_a\_at, 1460386\_a\_at |
| Slc24a1 | View | View |  | 778 | 1.57 | 17.69 | 14.73 | 111.40 | Link |  | SOLUTE CARRIER FAMILY 24 (SODIUM/POTASSIUM/CALCIUM EXCHANGER), MEMBER 1 | 5670 | 3604 | 15495 | 876 | 5670 | 385 | 10583 | 95 | chr9:64720865-64749613 | 1451647\_at |
| Slc24a4 | View | View |  | - | 0.35 | - | 0.28 | 0.27 |  |  | SOLUTE CARRIER FAMILY 24 (SODIUM/POTASSIUM/CALCIUM EXCHANGER), MEMBER 4 | 308 | 876 | 453 | 465 | 308 | 1113 | 381 | 1421 | chr12:102530469-102667799 | 1435206\_at |
| Slc25a25 | View | View |  | 388 | - | 3.11 | 2.10 | 3.52 |  |  | SOLUTE CARRIER FAMILY 25 (MITOCHONDRIAL CARRIER, PHOSPHATE CARRIER), MEMBER 25 | 1674 | 1645 | 1578 | 507 | 1674 | 799 | 1626 | 462 | chr2:32236493-32273431 | 1424735\_at |
| Slc26a4 | View | View |  | 314 | - | 0.15 | - | 0.17 |  |  | SOLUTE CARRIER FAMILY 26, MEMBER 4 | 26 | 36 | 34 | 231 | 26 | 51 | 30 | 174 | chr12:32105579-32145728 | 1419725\_at |
| Slc2a4 | View | View |  | 316 | - | - | 0.13 | 0.16 |  |  | SOLUTE CARRIER FAMILY 2 (FACILITATED GLUCOSE TRANSPORTER), MEMBER 4 | 14 | 42 | 37 | 52 | 14 | 109 | 26 | 160 | chr11:69758738-69764344 | 1415959\_at |
| Slc38a1 | View | View |  | 1332 | 0.49 | 0.73 | 0.59 | 0.55 |  |  | SOLUTE CARRIER FAMILY 38, MEMBER 1 | 5279 | 10798 | 5603 | 7650 | 5279 | 8953 | 5441 | 9866 | chr15:96404032-96470947 | 1454764\_s\_at, 1415903\_at |
| Slc6a6 | View | View |  | - | - | 1.96 | 0.66 | - |  |  | SOLUTE CARRIER FAMILY 6 (NEUROTRANSMITTER TRANSPORTER, TAURINE), MEMBER 6 | 7260 | 9211 | 10896 | 5549 | 7260 | 11053 | 9078 | 9098 | chr6:91649599-91724570 | 1437149\_at, 1421346\_a\_at |
| Slc8a3 | View | View |  | 518 | - | - | 0.14 | 0.47 |  |  | SOLUTE CARRIER FAMILY 8 (SODIUM/CALCIUM EXCHANGER), MEMBER 3 | 173 | 311 | 381 | 296 | 173 | 1210 | 277 | 588 | chr12:82118342-82252028 | 1450311\_at, 1440962\_at |
| Slco4a1 | View | View |  | - | - | 3.14 | 3.67 | 7.91 | Link |  | SOLUTE CARRIER ORGANIC ANION TRANSPORTER FAMILY, MEMBER 4A1 | 2905 | 2407 | 1775 | 565 | 2905 | 791 | 2340 | 296 | chr2:180385742-180404260 | 1455803\_at, 1438160\_x\_at |
| Slit3 | View | View |  | 444 | 0.10 | - | - | - |  |  | SLIT HOMOLOG 3 (DROSOPHILA) | 31 | 325 | 26 | 21 | 31 | 52 | 29 | 11 | chr11:34964938-35551565 | 1452296\_at |
| Smad3 | View | View |  | 750 | - | - | 0.13 | - |  |  | MAD HOMOLOG 3 (DROSOPHILA) | 20 | 48 | 88 | 146 | 20 | 153 | 54 | 147 | chr9:63444773-63556000 | 1450471\_at |
| Smarcb1 | View | View |  | - | - | - | 0.27 | - |  |  | SWI/SNF RELATED, MATRIX ASSOCIATED, ACTIN DEPENDENT REGULATOR OF CHROMATIN, SUBFAMILY B, MEMBER 1 | 305 | 469 | 577 | 382 | 305 | 1131 | 441 | 977 | chr10:75340491-75365312 | 1435856\_x\_at |
| Smoc2 | View | View |  | 321 | - | 0.07 | - | - |  |  | SPARC RELATED MODULAR CALCIUM BINDING 2 | 79 | 80 | 88 | 1184 | 79 | 94 | 84 | 125 | chr17:14016132-14138248 | 1415935\_at, 1431362\_a\_at |
| Smpdl3a | View | View |  | - | 0.35 | 0.34 | 0.19 | 0.14 |  |  | SPHINGOMYELIN PHOSPHODIESTERASE, ACID-LIKE 3A | 180 | 516 | 218 | 639 | 180 | 955 | 199 | 1375 | chr10:57483015-57500236 | 1416635\_at |
| Smug1 | View | View |  | 654 | 0.25 | 2.80 | 0.43 | - |  |  | SINGLE-STRAND SELECTIVE MONOFUNCTIONAL URACIL DNA GLYCOSYLASE | 2173 | 8769 | 2502 | 895 | 2173 | 5061 | 2338 | 1727 | chr15:102981341-102985963 | 1428763\_at |
| Snapc4 | View | View |  | - | - | - | 0.15 | - |  |  | SMALL NUCLEAR RNA ACTIVATING COMPLEX, POLYPEPTIDE 4 | 16 | 28 | 65 | 76 | 16 | 110 | 41 | 48 | chr2:26184783-26202628 | 1441795\_at |
| Snrpn | View | View |  | 972 | - | - | 0.49 | - |  |  | SMALL NUCLEAR RIBONUCLEOPROTEIN N | 4709 | 4211 | 9825 | 9740 | 4709 | 9685 | 7267 | 9826 | chr7:59862190-59866513 | 1421063\_s\_at |
| Snta1 | View | View |  | 772 | - | 2.40 | 3.01 | 4.21 |  |  | SYNTROPHIN, ACIDIC 1 | 1314 | 1205 | 999 | 417 | 1314 | 437 | 1157 | 275 | chr2:154067769-154099522 | 1416825\_at |
| Sntg2 | View | View |  | - | - | 8.42 | 3.18 | 8.03 |  |  | SYNTROPHIN, GAMMA 2 | 506 | 345 | 522 | 62 | 506 | 159 | 514 | 64 | chr12:30761046-30959380 | 1418789\_at |
| Snx15 | View | View |  | - | - | - | 0.19 | - |  |  | SORTING NEXIN 15 | 21 | 51 | 88 | 96 | 21 | 113 | 55 | 81 | chr19:6119399-6128215 | 1456649\_at |
| Socs3 | View | View |  | - | - | 0.12 | 0.17 | - |  |  | SUPPRESSOR OF CYTOKINE SIGNALING 3 | 138 | 250 | 113 | 909 | 138 | 835 | 126 | 61 | chr11:117782179-117785276 | 1455899\_x\_at, 1456212\_x\_at, 1416576\_at |
| Spop | View | View |  | 1376 | - | - | 0.28 | - |  |  | SPECKLE-TYPE POZ PROTEIN | 735 | 695 | 2103 | 1338 | 735 | 2671 | 1419 | 2764 | chr11:95230204-95309103 | 1447827\_x\_at |
| Srp72 | View | View |  | 288 | - | - | 0.28 | 0.50 |  |  | SIGNAL RECOGNITION PARTICLE 72 | 977 | 1062 | 3014 | 3527 | 977 | 3531 | 1996 | 3963 | chr5:78049483-78074707 | 1428877\_at |
| Srpk2 | View | View |  | - | - | - | 0.33 | - |  |  | SERINE/ARGININE-RICH PROTEIN SPECIFIC KINASE 2 | 525 | 686 | 1604 | 1612 | 525 | 1607 | 1065 | 1391 | chr5:23019360-23077091 | 1417136\_s\_at |
| St3gal1 | View | View |  | 251 | - | 2.89 | 5.15 | - |  |  | ST3 BETA-GALACTOSIDE ALPHA-2,3-SIALYLTRANSFERASE 1 | 206 | 108 | 202 | 70 | 206 | 40 | 204 | 49 | chr15:66932544-66943661 | 1418946\_at |
| St3gal3 | View | View |  | - | - | 0.28 | 0.11 | 0.28 |  |  | ST3 BETA-GALACTOSIDE ALPHA-2,3-SIALYLTRANSFERASE 3 | 130 | 187 | 208 | 742 | 130 | 1161 | 169 | 603 | chr4:117430086-117632822 | 1421915\_a\_at, 1450406\_a\_at |
| St8sia1 | View | View |  | 365 | - | 6.30 | 5.47 | 5.58 |  |  | ST8 ALPHA-N-ACETYL-NEURAMINIDE ALPHA-2,8-SIALYLTRANSFERASE 1 | 235 | 173 | 189 | 30 | 235 | 43 | 212 | 38 | chr6:142778763-142921674 | 1419695\_at, 1455695\_at, 1419694\_at |
| Stard7 | View | View |  | 606 | - | 2.67 | 2.10 | 2.48 |  |  | START DOMAIN CONTAINING 7 | 8276 | 6524 | 8047 | 3009 | 8276 | 3941 | 8162 | 3291 | chr2:126961680-126990381 | 1449628\_s\_at |
| Steap4 | View | View |  | 413 | - | 0.13 | - | - |  |  | STEAP FAMILY MEMBER 4 | 20 | 19 | 24 | 182 | 20 | 42 | 22 | 19 | chr5:7966478-7988219 | 1460197\_a\_at |
| Stk17b | View | View |  | 464 | 4.45 | 5.68 | 7.44 | 13.39 |  |  | SERINE/THREONINE KINASE 17B (APOPTOSIS-INDUCING) | 409 | 92 | 341 | 60 | 409 | 55 | 375 | 28 | chr1:53700057-53729699 | 1450997\_at |
| Stk35 | View | View |  | 628 | - | 2.69 | 3.49 | 2.46 |  |  | SERINE/THREONINE KINASE 35 | 4620 | 4316 | 7181 | 2667 | 4620 | 1325 | 5901 | 2398 | chr2:129492002-129523728 | 1439802\_at |
| Stk36 | View | View |  | 408 | - | - | 0.13 | - |  |  | SERINE/THREONINE KINASE 36 (FUSED HOMOLOG, DROSOPHILA) | 15 | 10 | 11 | 9 | 15 | 114 | 13 | 37 | chr1:74534662-74570103 | 1453584\_at |
| Gene Symbol | Large Image Gene +/- 15kb | Closeup Image TSS +/- 15kb | Links | Max Score (threshold of 200) | *Nr2e3 -/-* | *Crx -/-* | *Nrl -/-* | *Crx;Nrl -/-* | In situ  hybridization | Electroporation | Full Name | Averaged Affy Scores | | | | | | | | Locus | Affy Mouse 430 2 |
|  |  |  |  |  |  |  |  |  |  |  |  | B6 | *Nr2e3 -/-* | 129 | *Crx -/-* | B6 | *Nrl -/-* | B6;129 | *Crx;Nrl -/-* |  |  |
| Stom | View | View |  | - | - | 1.55 | 0.29 | - |  |  | STOMATIN | 301 | 293 | 584 | 376 | 301 | 1049 | 443 | 432 | chr2:35135999-35159018 | 1419098\_at, 1449341\_a\_at, 1419099\_x\_at |
| Supt16h | View | View |  | 304 | - | - | 0.16 | - |  |  | SUPPRESSOR OF TY 16 HOMOLOG (S. CEREVISIAE) | 101 | 66 | 712 | 716 | 101 | 627 | 407 | 671 | chr14:51082365-51119152 | 1449578\_at |
| Susd3 | View | View |  | - | 1.51 | 45.63 | 21.62 | 93.97 |  |  | SUSHI DOMAIN CONTAINING 3 | 3589 | 2381 | 2236 | 49 | 3589 | 166 | 2913 | 31 | chr13:49242627-49260643 | 1428975\_at |
| Syt1 | View | View |  | 482 | - | - | 0.29 | - |  |  | SYNAPTOTAGMIN I | 1866 | 1706 | 5884 | 6683 | 1866 | 6437 | 3875 | 6921 | chr10:107901762-108415063 | 1421990\_at, 1431191\_a\_at |
| Taf4b | View | View |  | 781 | 4.44 | 1.78 | 5.97 | 4.53 |  |  | TAF4B RNA POLYMERASE II, TATA BOX BINDING PROTEIN (TBP)-ASSOCIATED FACTOR | 1599 | 360 | 838 | 472 | 1599 | 268 | 1219 | 269 | chr18:14984630-15043377 | 1435303\_at |
| Tagln2 | View | View |  | 1235 | - | 0.20 | - | - |  |  | TRANSGELIN 2 | 58 | 65 | 39 | 200 | 58 | 17 | 49 | 27 | chr1:174336722-174344047 | 1439407\_x\_at |
| Taok3 | View | View |  | - | - | - | 0.17 | - |  |  | TAO KINASE 3 | 23 | 10 | 85 | 60 | 23 | 133 | 54 | 83 | chr5:117381159-117534864 | 1436261\_at |
| Tbc1d2 | View | View |  | 1135 | - | - | 0.14 | - |  |  | TBC1 DOMAIN FAMILY, MEMBER 2 | 18 | 25 | 19 | 15 | 18 | 130 | 19 | 12 | chr4:46625490-46671299 | 1456792\_at |
| Tdrd7 | View | View |  | 476 | 1.52 | 2.94 | 3.46 | 4.99 |  |  | TUDOR DOMAIN CONTAINING 7 | 9477 | 6228 | 9100 | 3094 | 9477 | 2741 | 9289 | 1862 | chr4:46008560-46055861 | 1426716\_at |
| Tecta | View | View |  | 694 | - | 0.04 | - | - |  |  | TECTORIN ALPHA | 17 | 29 | 54 | 1329 | 17 | 38 | 36 | 37 | chr9:42080617-42150924 | 1419632\_at |
| Tef | View | View |  | - | - | - | 0.27 | - |  |  | THYROTROPH EMBRYONIC FACTOR | 355 | 291 | 795 | 896 | 355 | 1292 | 575 | 955 | chr15:81630072-81654118 | 1450184\_s\_at |
| Tgm3 | View | View |  | - | - | 7.30 | - | - |  |  | TRANSGLUTAMINASE 3, E POLYPEPTIDE | 58 | 53 | 416 | 57 | 58 | 46 | 237 | 22 | chr2:129703790-129740002 | 1440150\_at |
| Thoc3 | View | View |  | - | - | - | 0.09 | - |  |  | THO COMPLEX 3 | 44 | 38 | 423 | 495 | 44 | 468 | 234 | 483 | chr13:54469140-54478441 | 1423971\_at |
| Thoc5 | View | View |  | 434 | - | 3.14 | 4.41 | 4.52 |  |  | FMS INTERACTING PROTEIN | 3538 | 3352 | 1977 | 630 | 3538 | 802 | 2758 | 610 | chr11:4795346-4828868 | 1452672\_at |
| Thrap2 | View | View |  | 978 | 1.41 | 1.57 | 4.42 | 2.65 |  |  | THYROID HORMONE RECEPTOR ASSOCIATED PROTEIN 2 | 5362 | 3801 | 1769 | 1124 | 5362 | 1214 | 3566 | 1345 | chr5:118821773-119024010 | 1434603\_at |
| Timm44 | View | View |  | 634 | 1.95 | - | 3.21 | - |  |  | TRANSLOCASE OF INNER MITOCHONDRIAL MEMBRANE 44 | 4116 | 2108 | 1344 | 1134 | 4116 | 1281 | 2730 | 1211 | chr8:4259888-4275895 | 1439371\_x\_at |
| Tkt | View | View |  | - | - | - | 0.31 | - |  |  | TRANSKETOLASE | 1350 | 1257 | 4482 | 4751 | 1350 | 4376 | 2916 | 4533 | chr14:29378158-29403731 | 1451015\_at, 1439443\_x\_at |
| Tmed9 | View | View |  | 342 | - | - | 0.28 | - |  |  | TRANSMEMBRANE EMP24 PROTEIN TRANSPORT DOMAIN CONTAINING 9 | 648 | 543 | 2109 | 2127 | 648 | 2335 | 1379 | 2063 | chr13:55602765-55607310 | 1448268\_at |
| Tmem108 | View | View |  | - | - | 1.71 | 3.40 | 4.03 |  |  | TRANSMEMBRANE PROTEIN 108 | 1402 | 1116 | 864 | 505 | 1402 | 412 | 1133 | 281 | chr9:103342589-103619967 | 1454729\_at |
| Tmem136 | View | View |  | 297 | - | 5.96 | 1.58 | 4.41 |  |  | TRANSMEMBRANE PROTEIN 136 | 3877 | 2581 | 4293 | 720 | 3877 | 2459 | 4085 | 926 | chr9:42859648-42867565 | 1457701\_at |
| Tmem142a | View | View |  | 1539 | - | - | 0.27 | - |  |  | TRANSMEMBRANE PROTEIN 142A | 272 | 616 | 320 | 457 | 272 | 1023 | 296 | 540 | chr5:123275691-123290773 | 1424990\_at |
| Tmem20 | View | View |  | 493 | 0.40 | 0.44 | 0.18 | 0.20 |  |  | TRANSMEMBRANE PROTEIN 20 | 157 | 397 | 248 | 568 | 157 | 895 | 203 | 1015 | chr19:38461124-38470751 | 1435452\_at |
| Tmem26 | View | View |  | 1080 | 0.18 | - | - | - |  |  | TRANSMEMBRANE PROTEIN 26 | 517 | 2818 | 182 | 110 | 517 | 641 | 350 | 198 | chr10:68119103-68175968 | 1435409\_at |
| Tmlhe | - | - |  | - | - | 6.91 | - | 2.56 |  |  | TRIMETHYLLYSINE HYDROXYLASE, EPSILON | 404 | 279 | 608 | 88 | 404 | 483 | 506 | 198 | chrX | 1420726\_x\_at, 1420725\_at, 1420727\_a\_at |
| Tmod1 | View | View |  | - | - | 0.16 | - | - |  |  | TROPOMODULIN 1 | 132 | 118 | 80 | 508 | 132 | 117 | 106 | 245 | chr4:46082066-46135904 | 1422754\_at |
| Tnfaip3 | View | View |  | 475 | 0.68 | 11.30 | 4.77 | 10.49 |  |  | TUMOR NECROSIS FACTOR, ALPHA-INDUCED PROTEIN 3 | 1031 | 1507 | 1989 | 176 | 1031 | 216 | 1510 | 144 | chr10:18690335-18704826 | 1450829\_at, 1433699\_at |
| Tnfrsf12a | View | View |  | 319 | - | 0.04 | - | 0.03 |  |  | TUMOR NECROSIS FACTOR RECEPTOR SUPERFAMILY, MEMBER 12A | 26 | 99 | 48 | 1337 | 26 | 112 | 37 | 1448 | chr17:23403063-23405066 | 1418571\_at, 1418572\_x\_at |
| Tnfsf13 | View | View |  | 212 | - | 3.01 | - | 2.33 |  |  | TUMOR NECROSIS FACTOR (LIGAND) SUPERFAMILY, MEMBER 13 | 2295 | 2597 | 2287 | 759 | 2295 | 1951 | 2291 | 985 | chr11:69498772-69512293 | 1418345\_at |
| Tnnt2 | View | View |  | - | - | - | 5.38 | - |  |  | TROPONIN T2, CARDIAC | 129 | 127 | 14 | 32 | 129 | 24 | 72 | 8 | chr1:137657286-137668671 | 1418726\_a\_at, 1424967\_x\_at |
| Tnpo1 | View | View |  | 261 | - | - | 2.35 | 2.21 |  |  | TRANSPORTIN 1 | 6650 | 7278 | 4324 | 3514 | 6650 | 2827 | 5487 | 2483 | chr13:99942895-100026541 | 1433585\_at |
| Tob1 | View | View |  | 1326 | - | - | 0.43 | 0.55 |  |  | TRANSDUCER OF ERBB-2.1 | 3790 | 4684 | 4340 | 5200 | 3790 | 8912 | 4065 | 7429 | chr11:94027544-94031585 | 1423176\_at, 1440844\_at |
| Tom1l2 | View | View |  | 224 | - | - | 0.20 | - |  |  | TARGET OF MYB1-LIKE 2 (CHICKEN) | 105 | 108 | 222 | 256 | 105 | 526 | 164 | 283 | chr11:60046211-60169079 | 1451795\_at |
| Traf3ip3 | View | View |  | 247 | 2.50 | 13.61 | - | 5.34 |  |  | TRAF3 INTERACTING PROTEIN 3 | 513 | 205 | 449 | 33 | 513 | 226 | 481 | 90 | chr1:194876230-194902210 | 1434573\_at |
| Trak2 | View | View |  | - | - | - | 0.17 | 0.34 |  |  | TRAFFICKING PROTEIN, KINESIN BINDING 2 | 66 | 77 | 229 | 291 | 66 | 378 | 148 | 437 | chr1:58844996-58917976 | 1429607\_at |
| Trappc3 | View | View |  | 261 | - | - | 0.16 | - |  |  | TRAFFICKING PROTEIN PARTICLE COMPLEX 3 | 515 | 543 | 3113 | 2932 | 515 | 3246 | 1814 | 3331 | chr4:125764709-125778187 | 1416771\_at |
| Trim17 | View | View |  | 357 | - | - | 0.20 | - |  |  | TRIPARTITE MOTIF PROTEIN 17 | 44 | 95 | 51 | 58 | 44 | 218 | 48 | 63 | chr11:58770880-58789293 | 1427507\_at |
| Trim36 | View | View |  | - | - | - | 5.21 | 5.04 |  |  | TRIPARTITE MOTIF-CONTAINING 36 | 745 | 335 | 697 | 226 | 745 | 143 | 721 | 143 | chr18:46291176-46337856 | 1441969\_at |
| Trp53 | View | View |  | - | - | 0.44 | 0.19 | - |  |  | TRANSFORMATION RELATED PROTEIN 53 | 25 | 46 | 86 | 196 | 25 | 135 | 56 | 189 | chr11:69396600-69407992 | 1426538\_a\_at |
| Tspan33 | View | View |  | 454 | - | - | 0.17 | - |  |  | TETRASPANIN 33 | 34 | 63 | 118 | 158 | 34 | 200 | 76 | 224 | chr6:29644266-29668572 | 1451609\_at |
| Tspan7 | View | View |  | 326 | - | - | 0.46 | - |  |  | TETRASPANIN 7 | 4596 | 3871 | 8080 | 6346 | 4596 | 10093 | 6338 | 7943 | chrX:9642075-9753565 | 1417502\_at |
| Tspan9 | View | View |  | 1232 | - | 0.45 | 0.17 | 0.33 |  |  | TETRASPANIN 9 | 63 | 61 | 138 | 308 | 63 | 380 | 101 | 303 | chr6:127927019-128109197 | 1441968\_at |
| ttc21a | View | View |  | - | - | 18.45 | - | - |  |  | TETRATRICOPEPTIDE REPEAT DOMAIN 21A | 140 | 82 | 203 | 11 | 140 | 61 | 172 | 23 | chr9:119786304-119816491 | 1442472\_at, 1441218\_at |
| Ttc9c | View | View |  | 246 | - | - | 0.20 | - |  |  | TETRATRICOPEPTIDE REPEAT DOMAIN 9C | 182 | 245 | 305 | 354 | 182 | 898 | 244 | 416 | chr19:8878341-8886333 | 1432245\_s\_at, 1434001\_at, 1430569\_at |
| Ttr | View | View |  | 1097 | 0.47 | 2.48 | - | 3.38 |  |  | TRANSTHYRETIN | 7409 | 15626 | 13758 | 5546 | 7409 | 8176 | 10584 | 3131 | chr18:20808423-20817331 | 1454608\_x\_at, 1451580\_a\_at, 1459737\_s\_at, 1455913\_x\_at |
| Ttyh2 | View | View |  | - | - | - | 0.17 | 0.19 |  |  | TWEETY HOMOLOG 2 (DROSOPHILA) | 81 | 64 | 50 | 195 | 81 | 469 | 66 | 339 | chr11:114491558-114537074 | 1421179\_at |
| Tub | View | View |  | - | - | 1.65 | 2.27 | 2.65 |  |  | TUBBY CANDIDATE GENE | 5228 | 4758 | 3968 | 2401 | 5228 | 2305 | 4598 | 1732 | chr7:108741785-108825636 | 1420925\_at |
| Tubb6 | View | View |  | - | - | 0.08 | - | - |  |  | TUBULIN, BETA 6 | 54 | 91 | 32 | 390 | 54 | 65 | 43 | 149 | chr18:67516088-67528117 | 1416431\_at |
| Tulp1 | View | View |  | - | - | 1.81 | - | - | Link | Link | TUBBY LIKE PROTEIN 1 | 21316 | 21597 | 16140 | 8907 | 21316 | 16709 | 18728 | 14692 | chr17:28079105-28092737 | 1451582\_at |
| Txndc13 | View | View |  | - | - | - | 0.17 | - |  |  | THIOREDOXIN DOMAIN CONTAINING 13 | 76 | 86 | 504 | 526 | 76 | 449 | 290 | 453 | chr2:134285949-134335562 | 1453787\_at |
| Txnip | View | View |  | 550 | - | - | 0.08 | - |  |  | THIOREDOXIN INTERACTING PROTEIN | 19 | 16 | 159 | 264 | 19 | 245 | 89 | 109 | chr3:96643362-96655007 | 1415997\_at |
| Gene Symbol | Large Image Gene +/- 15kb | Closeup Image TSS +/- 15kb | Links | Max Score (threshold of 200) | *Nr2e3 -/-* | *Crx -/-* | *Nrl -/-* | *Crx;Nrl -/-* | In situ  hybridization | Electroporation | Full Name | Averaged Affy Scores | | | | | | | | Locus | Affy Mouse 430 2 |
|  |  |  |  |  |  |  |  |  |  |  |  | B6 | *Nr2e3 -/-* | 129 | *Crx -/-* | B6 | *Nrl -/-* | B6;129 | *Crx;Nrl -/-* |  |  |
| Txnl6 | View | View |  | 614 | - | 22.06 | 3.49 | 29.76 |  |  | THIOREDOXIN-LIKE 6 | 4172 | 3558 | 1125 | 51 | 4172 | 1197 | 2649 | 89 | chr8:74489544-74495590 | 1425696\_at |
| U2af1 | View | View |  | 304 | - | - | 0.27 | - |  |  | U2 SMALL NUCLEAR RIBONUCLEOPROTEIN AUXILIARY FACTOR (U2AF) 1 | 827 | 758 | 3263 | 3181 | 827 | 3023 | 2045 | 3073 | chr17:31375793-31387432 | 1422509\_at |
| Uckl1 | View | View |  | - | - | 3.46 | - | - |  |  | URIDINE-CYTIDINE KINASE 1-LIKE 1 | 6121 | 5366 | 5425 | 1569 | 6121 | 5307 | 5773 | 4641 | chr2:181498559-181511380 | 1424646\_at |
| Unc119 | View | View |  | 879 | - | 1.42 | 1.29 | 1.30 | Link | Link | UNC-119 HOMOLOG (C. ELEGANS) | 27076 | 28427 | 25073 | 17674 | 27076 | 20962 | 26075 | 19985 | chr11:78159717-78165351 | 1418123\_at |
| Ush1g | View | View |  | 264 | NA | NA | NA | NA |  |  | USHER SYNDROME 1G HOMOLOG (HUMAN) |  |  |  |  |  |  |  |  | chr11:115134071-115137946 |  |
| Ush2a | View | View |  | 1126 | - | - | - | - | Link |  | USHER SYNDROME 2A (AUTOSOMAL RECESSIVE, MILD) HOMOLOG (HUMAN) |  |  |  |  |  |  |  |  | chr1:189962811-190665716 |  |
| Ush3a | View | View |  | 779 | - | - | - | - |  | Link | USHER SYNDROME 3A HOMOLOG (HUMAN) | 59 | 70 | 149 | 184 | 59 | 154 | 104 | 172 | chr3:58933144-58973141 | 1438347\_at |
| Usp2 | View | View |  | 310 | 0.45 | 0.60 | 0.75 | - |  |  | UBIQUITIN SPECIFIC PEPTIDASE 2 | 2712 | 6053 | 2015 | 3333 | 2712 | 3621 | 2364 | 2865 | chr9:43818134-43845472 | 1417168\_a\_at |
| Vamp4 | View | View |  | - | - | 0.64 | 0.43 | 0.49 |  |  | VESICLE-ASSOCIATED MEMBRANE PROTEIN 4 | 2383 | 3065 | 3011 | 4698 | 2383 | 5565 | 2697 | 5531 | chr1:164407398-164435753 | 1422895\_at |
| Vax2os1 | View | View |  | 457 | 1.53 | 5.70 | 6.83 | 21.68 | Link |  | VAX2 OPPOSITE STRAND TRANSCRIPT 1 | 1229 | 805 | 1373 | 241 | 1229 | 180 | 1301 | 60 | chr6:83667784-83676590 | 1446754\_a\_at, 1443232\_at |
| Vps35 | View | View |  | 565 | - | - | 0.26 | - |  |  | VACUOLAR PROTEIN SORTING 35 | 326 | 357 | 1521 | 1547 | 326 | 1246 | 924 | 1592 | chr8:88151235-88185062 | 1415784\_at |
| Vtn | View | View |  | 661 | - | 22.51 | 1.52 | 6.90 | Link |  | VITRONECTIN | 17518 | 16702 | 13575 | 603 | 17518 | 11532 | 15547 | 2254 | chr11:78315315-78318518 | 1420484\_a\_at, 1455098\_a\_at |
| Wdfy1 | View | View |  | - | 5.77 | - | 0.55 | - |  |  | WD REPEAT AND FYVE DOMAIN CONTAINING 1 | 1229 | 213 | 2142 | 2264 | 1229 | 2219 | 1686 | 2470 | chr1:79584403-79601262 | 1437358\_at, 1435588\_at |
| Wdr17 | View | View |  | - | - | 1.96 | 2.00 | 2.25 |  |  | WD REPEAT DOMAIN 17 | 7037 | 6153 | 3810 | 1948 | 7037 | 3523 | 5424 | 2408 | chr8:56128319-56223065 | 1435392\_at |
| Wdr31 | View | View |  | 608 | 0.50 | 4.02 | 0.21 | - |  |  | WD REPEAT DOMAIN 31 | 639 | 1281 | 1607 | 400 | 639 | 2992 | 1123 | 1323 | chr4:61940095-61957238 | 1423636\_at, 1459664\_at |
| Wdr63 | View | View |  | 205 | - | 69.67 | - | - |  |  | WD REPEAT DOMAIN 63 | 36 | 38 | 209 | 3 | 36 | 18 | 123 | 11 | chr3:145977913-146045422 | 1436675\_at |
| Wdr66 | View | View |  | - | - | 25.12 | - | 27.39 |  |  | WD REPEAT DOMAIN 66 | 833 | 1114 | 427 | 17 | 833 | 600 | 630 | 23 | chr5:123532219-123587412 | 1430112\_at |
| Wisp1 | View | View |  | - | 1.69 | 28.03 | 59.17 | 25.93 |  |  | WNT1 INDUCIBLE SIGNALING PATHWAY PROTEIN 1 | 1361 | 804 | 869 | 31 | 1361 | 23 | 1115 | 43 | chr15:66721062-66752868 | 1448594\_at, 1448593\_at |
| Wwc1 | View | View |  | 285 | - | 0.24 | 0.16 | - |  |  | WW, C2 AND COILED-COIL DOMAIN CONTAINING 1 | 212 | 498 | 257 | 1069 | 212 | 1357 | 235 | 2217 | chr11:35681827-35823890 | 1420008\_s\_at, 1427261\_at |
| Ykt6 | View | View |  | - | - | - | 0.16 | 0.41 |  |  | YKT6 HOMOLOG (S. CEREVISIAE) | 184 | 186 | 667 | 724 | 184 | 1159 | 426 | 1032 | chr11:5855835-5867785 | 1460191\_at |
| Ypel2 | View | View |  | 602 | - | - | 5.78 | - |  |  | YIPPEE-LIKE 2 (DROSOPHILA) | 439 | 272 | 160 | 111 | 439 | 76 | 300 | 107 | chr11:86756305-86809902 | 1440560\_at |
| Ywhab | View | View |  | - | - | - | 0.28 | - |  |  | TYROSINE 3-MONOOXYGENASE/TRYPTOPHAN 5-MONOOXYGENASE ACTIVATION PROTEIN, BETA POLYPEPTIDE | 357 | 368 | 2508 | 2445 | 357 | 1269 | 1433 | 2312 | chr2:163686638-163710028 | 1420878\_a\_at |
| Ywhag | View | View |  | 516 | - | - | 0.09 | - |  |  | 3-MONOOXYGENASE/TRYPTOPHAN 5-MONOOXYGENASE ACTIVATION PROTEIN, GAMMA POLYPEPTIDE | 58 | 51 | 614 | 745 | 58 | 617 | 336 | 667 | chr5:136193038-136219180 | 1420816\_at |
| Zbtb7c | View | View |  | 554 | - | 0.09 | - | - |  |  | ZINC FINGER AND BTB DOMAIN CONTAINING 7C | 81 | 110 | 17 | 189 | 81 | 60 | 49 | 21 | chr18:75945543-76273929 | 1436365\_at |
| Zc3h11a | View | View |  | 736 | - | - | 0.31 | - |  |  | ZINC FINGER CCCH TYPE CONTAINING 11A | 334 | 328 | 833 | 772 | 334 | 1067 | 584 | 851 | chr1:135449041-135488902 | 1426361\_at |
| Zdhhc14 | View | View |  | 276 | 0.29 | 0.37 | 0.38 | 0.36 |  |  | ZINC FINGER, DHHC DOMAIN CONTAINING 14 | 1219 | 4169 | 403 | 1096 | 1219 | 3210 | 811 | 2244 | chr17:5449082-5710373 | 1437614\_x\_at, 1438619\_x\_at, 1438975\_x\_at, 1423668\_at |
| Zfp146 | View | View |  | - | - | - | 0.16 | - |  |  | ZINC FINGER PROTEIN 146 | 74 | 68 | 452 | 455 | 74 | 453 | 263 | 427 | chr7:29870034-29878487 | 1422135\_at |
| Zfp316 | View | View |  | - | - | - | 0.19 | - |  |  | ZINC FINGER PROTEIN 316 | 45 | 34 | 251 | 268 | 45 | 239 | 148 | 295 | chr5:143516867-143529793 | 1450151\_at |
| Zfp36l2 | View | View |  | - | - | 2.58 | 5.31 | 6.39 |  |  | ZINC FINGER PROTEIN 36, C3H TYPE-LIKE 2 | 3880 | 3168 | 3410 | 1323 | 3880 | 731 | 3645 | 570 | chr17:84092250-84096273 | 1437626\_at |
| Zfp664 | View | View |  | - | - | - | 0.12 | - |  |  | ZINC FINGER PROTEIN 664 | 23 | 47 | 204 | 201 | 23 | 198 | 114 | 160 | chr5:125151743-125191672 | 1437047\_at |
| Zfyve28 | View | View |  | 245 | 4.29 | 6.18 | 8.18 | 8.83 |  |  | ZINC FINGER, FYVE DOMAIN CONTAINING 28 | 695 | 162 | 346 | 56 | 695 | 85 | 521 | 59 | chr5:34511729-34605183 | 1443669\_at, 1434504\_at |
| 1425288\_at | - | - |  | - | - | 3.35 | 14.18 | 41.66 |  |  | STERILE ALPHA MOTIF DOMAIN CONTAINING 11 | 7855 | 6558 | 6475 | 1930 | 7855 | 554 | 7165 | 172 | - | 1425288\_at |
| 1435462\_at | - | - |  | - | - | 1.79 | 2.46 | - |  |  | HYPOTHETICAL LOC433022 | 6785 | 6066 | 2974 | 1658 | 6785 | 2763 | 4880 | 2134 | - | 1435462\_at |
| 1435819\_at | - | - |  | - | - | 0.28 | - | 0.38 |  |  | HYPOTHETICAL LOC230602 | 250 | 286 | 375 | 1323 | 250 | 271 | 313 | 826 | - | 1435819\_at |
| 1436287\_at | - | - |  | - | 1.85 | 10.97 | - | 8.76 |  |  | ,GB:BF466943 /DB\_XREF=GI:11536126 /DB\_XREF=UI-M-CG0P-BRB-G-11-0-UI.S1 /CLONE=UI M-CG0P-BRB-G-11-0-UI /FEA=EST /CNT=10 /TID=MM.58847.1 /TIER=STACK /STK=10 /UG=MM.58847 /UG\_TITLE=ESTS [+/-]Show full name | 277 | 150 | 318 | 29 | 277 | 223 | 298 | 34 | - | 1436287\_at |
| 1436633\_at | - | - |  | - | 0.50 | 3.17 | 0.13 | 0.24 |  |  | GENE MODEL 1567, (NCBI) | 159 | 316 | 279 | 88 | 159 | 1212 | 219 | 897 | - | 1436633\_at |
| 1437003\_at | - | - |  | - | 2.14 | 3.60 | 3.45 | 3.76 |  |  | ,GB:BB323930 /DB\_XREF=GI:16403486 /DB\_XREF=BB323930 /CLONE=B430101L16 /FEA=EST CNT=35 /TID=MM.17706.1 /TIER=STACK /STK=30 /UG=MM.17706 /UG\_TITLE=ESTS [+/-]Show full name | 1297 | 607 | 953 | 265 | 1297 | 376 | 1125 | 299 | - | 1437003\_at |
| 1438068\_at | View | View |  | 300 | - | - | 0.15 | 0.27 |  |  | ,GB:BB251859 /DB\_XREF=GI:8944605 /DB\_XREF=BB251859 /CLONE=A730048G08 /FEA=EST / NT=21 /TID=MM.100454.1 /TIER=STACK /STK=12 /UG=MM.100454 /UG\_TITLE=ESTS [+/-]Show full name | 192 | 284 | 348 | 455 | 192 | 1266 | 270 | 982 | chr5:108605857-108606670 | 1438068\_at |
| 1439911\_at | View | View |  | 205 | 0.38 | - | 0.18 | 0.14 |  |  | SIMILAR TO SYNDECAN 2 | 136 | 356 | 292 | 322 | 136 | 744 | 214 | 1546 | chr15:32736771-32743720 | 1439911\_at |
| 1439936\_at | View | View |  | 543 | 0.21 | 5.12 | 0.30 | - |  |  | ,GB:BE949265 /DB\_XREF=GI:10527024 /DB\_XREF=UI-M-BH3-AVF-A-04-0-UI.S1 /CLONE=UI- -BH3-AVF-A-04-0-UI /FEA=EST /CNT=7 /TID=MM.150542.1 /TIER=CONSEND /STK=6 /UG=MM.150542 /UG\_TITLE=ESTS [+/-]Show full name | 398 | 1863 | 471 | 92 | 398 | 1343 | 435 | 356 | chr17:45194709-45195799 | 1439936\_at |
| 1441518\_at | View | View |  | - | 2.46 | 11.76 | 3.31 | 10.78 |  |  | ,GB:BB277231 /DB\_XREF=GI:16400956 /DB\_XREF=BB277231 /CLONE=A930003C13 /FEA=EST CNT=4 /TID=MM.115892.1 /TIER=CONSEND /STK=4 /UG=MM.115892 /UG\_TITLE=ESTS [+/-]Show full name | 295 | 120 | 200 | 17 | 295 | 89 | 248 | 23 | chr11:118921222-118921885 | 1441518\_at |
| 1442190\_at | View | View |  | - | - | 275.00 | 50.20 | 326.00 |  |  | ,GB:BI736087 /DB\_XREF=GI:15713100 /DB\_XREF=603359238F1 /CLONE=IMAGE:5366529 /FE =EST /CNT=6 /TID=MM.35149.1 /TIER=CONSEND /STK=3 /UG=MM.35149 /UG\_TITLE=ESTS, WEAKLY SIMILAR TO POL2 MOUSE RETROVIRUS-RELATED POL POLYPROTEIN (M.MUSCULUS) [+/-]Show full name | 753 | 493 | 550 | 2 | 753 | 15 | 652 | 2 | chr13:24343734-24378868 | 1442190\_at |
| 1442249\_at | View | View |  | 367 | - | 8.91 | - | 3.37 |  |  | ,GB:BB283887 /DB\_XREF=GI:16401406 /DB\_XREF=BB283887 /CLONE=A930103L05 /FEA=EST CNT=5 /TID=MM.134465.1 /TIER=CONSEND /STK=3 /UG=MM.134465 /UG\_TITLE=ESTS [+/-]Show full name | 326 | 340 | 205 | 23 | 326 | 248 | 266 | 79 | chr4:148418770-148419475 | 1442249\_at |
| 1442304\_at | View | View |  | 822 | - | - | 6.55 | - |  |  | ,GB:BB520468 /DB\_XREF=GI:16443408 /DB\_XREF=BB520468 /CLONE=D830044D09 /FEA=EST CNT=5 /TID=MM.74650.1 /TIER=CONSEND /STK=3 /UG=MM.74650 /UG\_TITLE=ESTS [+/-]Show full name | 131 | 49 | 57 | 21 | 131 | 20 | 94 | 36 | chr19:27327829-27329420 | 1442304\_at |
| 1443238\_at | - | - |  | - | - | - | 0.13 | - |  |  | ,GB:BB370461 /DB\_XREF=GI:16406876 /DB\_XREF=BB370461 /CLONE=C130053G19 /FEA=EST CNT=3 /TID=MM.209048.1 /TIER=CONSEND /STK=3 /UG=MM.209048 /UG\_TITLE=ESTS [+/-]Show full name | 21 | 77 | 5 | 8 | 21 | 163 | 13 | 65 | - | 1443238\_at |
| 1443306\_at | View | View |  | 419 | - | - | 3.07 | 2.41 |  |  | ,GB:BB380163 /DB\_XREF=GI:16407890 /DB\_XREF=BB380163 /CLONE=C230004E12 /FEA=EST CNT=3 /TID=MM.80674.1 /TIER=CONSEND /STK=3 /UG=MM.80674 /UG\_TITLE=ESTS [+/-]Show full name | 1193 | 1206 | 712 | 483 | 1193 | 389 | 953 | 395 | chr12:84124017-84124650 | 1443306\_at |
| 1443552\_at | View | View |  | 350 | - | 15.85 | 12.52 | - |  |  | SIMILAR TO HYPOTHETICAL PROTEIN LOC57653 | 313 | 98 | 206 | 13 | 313 | 25 | 260 | 32 | chr4:45967300-45971875 | 1443552\_at |
| 1444095\_a\_at | View | View |  | 630 | - | 19.89 | - | - |  |  | ,GB:BE986579 /DB\_XREF=GI:10661053 /DB\_XREF=UI-M-CG0P-BEW-B-11-0-UI.S1 /CLONE=UI M-CG0P-BEW-B-11-0-UI /FEA=EST /CNT=6 /TID=MM.40852.1 /TIER=CONSEND /STK=2 /UG=MM.40852 /UG\_TITLE=ESTS [+/-]Show full name | 2298 | 2486 | 2924 | 147 | 2298 | 2260 | 2611 | 2728 | chr2:128869589-128872073 | 1444095\_a\_at |
| 1444552\_at | View | View |  | 350 | - | - | 0.14 | - |  |  | ,GB:BB730792 /DB\_XREF=GI:16114067 /DB\_XREF=BB730792 /CLONE=E860130C01 /FEA=EST CNT=4 /TID=MM.218148.1 /TIER=CONSEND /STK=2 /UG=MM.218148 /UG\_TITLE=ESTS, WEAKLY SIMILAR TO TYROSINE-PROTEIN KINASE JAK3 (M.MUSCULUS) [+/-]Show full name | 56 | 61 | 254 | 260 | 56 | 390 | 155 | 272 | chr17:46316950-46317484 | 1444552\_at |
| 1444603\_at | View | View |  | - | - | 11.71 | 2.66 | 7.12 |  |  | ,GB:AI648973 /DB\_XREF=GI:4729807 /DB\_XREF=UK33F01.X1 /CLONE=IMAGE:1970809 /FEA= ST /CNT=4 /TID=MM.74819.1 /TIER=CONSEND /STK=2 /UG=MM.74819 /UG\_TITLE=ESTS [+/-]Show full name | 287 | 236 | 410 | 35 | 287 | 108 | 349 | 49 | chr8:13201411-13201767 | 1444603\_at |
| Gene Symbol | Large Image Gene +/- 15kb | Closeup Image TSS +/- 15kb | Links | Max Score (threshold of 200) | *Nr2e3 -/-* | *Crx -/-* | *Nrl -/-* | *Crx;Nrl -/-* | In situ  hybridization | Electroporation | Full Name | Averaged Affy Scores | | | | | | | | Locus | Affy Mouse 430 2 |
|  |  |  |  |  |  |  |  |  |  |  |  | B6 | *Nr2e3 -/-* | 129 | *Crx -/-* | B6 | *Nrl -/-* | B6;129 | *Crx;Nrl -/-* |  |  |
| 1447191\_at | View | View |  | - | - | - | 0.09 | - |  |  | ,GB:AI875120 /DB\_XREF=GI:5549169 /DB\_XREF=UL29G01.X1 /CLONE=IMAGE:2099760 /FEA= ST /CNT=2 /TID=MM.139154.1 /TIER=CONSEND /STK=2 /UG=MM.139154 /UG\_TITLE=ESTS [+/-]Show full name | 9 | 10 | 22 | 17 | 9 | 101 | 16 | 20 | chr15:99970442-99970934 | 1447191\_at |
| 1449609\_at | - | - |  | - | 2.94 | 0.06 | 0.65 | 0.15 |  |  | HYPOTHETICAL LOC433755 | 238 | 81 | 25 | 402 | 238 | 364 | 132 | 875 | - | 1449609\_at |
| 1456101\_at | View | View |  | 503 | 0.01 | 0.23 | 0.05 | - |  |  | SIMILAR TO FERRITIN LIGHT CHAIN (FERRITIN L SUBUNIT) | 3 | 244 | 19 | 82 | 3 | 57 | 11 | 37 | chr12:73019876-73027758 | 1456101\_at |
| 1457284\_at | View | View |  | 794 | - | - | 0.15 | - |  |  | ,GB:BB766038 /DB\_XREF=GI:16208999 /DB\_XREF=BB766038 /CLONE=G370060B20 /FEA=EST CNT=12 /TID=MM.45553.1 /TIER=CONSEND /STK=4 /UG=MM.45553 /UG\_TITLE=ESTS [+/-]Show full name | 137 | 204 | 548 | 553 | 137 | 919 | 343 | 670 | chr17:56295029-56296073 | 1457284\_at |
| 1459721\_at | View | View |  | 1179 | - | 1.24 | 0.20 | - |  |  | ,GB:BF662619 /DB\_XREF=GI:11927753 /DB\_XREF=MAA80H06.X1 /CLONE=IMAGE:3823331 /FE =EST /CNT=2 /TID=MM.32079.1 /TIER=CONSEND /STK=2 /UG=MM.32079 /UG\_TITLE=ESTS [+/-]Show full name | 82 | 81 | 273 | 221 | 82 | 414 | 178 | 332 | chr19:60230891-60231255 | 1459721\_at |
