# Supplementary figures and images for: The Cis-regulatory Logic of the Mammalian Photoreceptor Transcriptional Network (part 2 of 3)
Source: PLoS One. 2007 Jul 25;2(7):e643. doi: 10.1371/journal.pone.0000643 (PMC1916400; doi:10.1371/journal.pone.0000643)

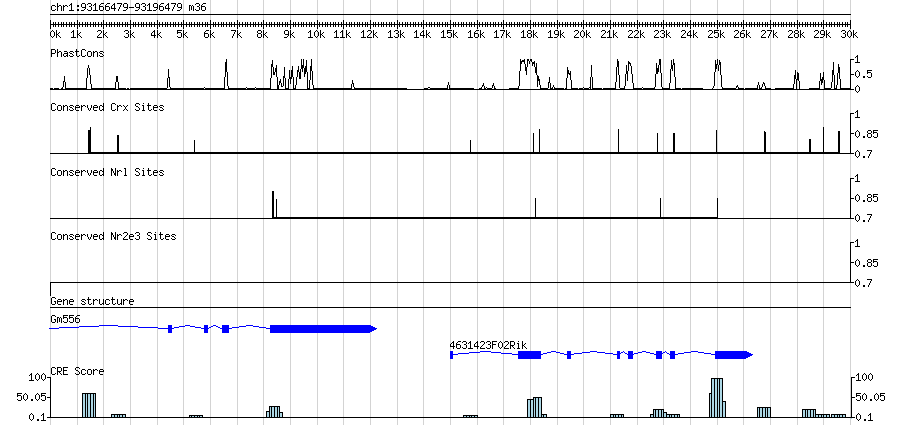

Supplement: Table S1 — Genes in the mouse photoreceptor transcription network. This table is a database which includes all genes dysregulated in Crx-/-, Nrl-/-, and/or Nr2e3-/- under high stringency criteria. Each row in the database represents a gene in the network and includes multiple links to additional types of information about that gene. ‘Large image gene +/− 15 Kb’ links to an image of our computational prediction of photoreceptor CREs in the genomic region 15 Kb upstream and downstream of the gene in question. ‘Closeup image TSS +/− 15 Kb’ links to an image of our computational prediction in the region 15 Kb on either side of the gene's TSS. Black bars within this image highlight the following regulatory peaks (if any are present): the peak closest to the TSS and the peak with the highest CRE score within this 30 Kb window. For those genes whose CRE predictions were tested experimentally, a red bar in this image indicates the location of the CRE that was tested. For Crx and Elovl4 the tested CRE is outside of this 30 Kb window and is therefore indicated in the ‘Large image’. ‘Links’ contains links to the UCSC genome browser, ENSEMBL, and NCBI database entries for the indicated gene, if available. ‘Max score (threshold of 200)’ contains the score of the highest predicted regulatory peak within the 30 Kb window around the gene's TSS. If this window does not contain any predicted peaks ≥ 200 (our cutoff threshold) no value is given (indicated by a dash). The next four columns of the table (‘Nr2e3-/-’ etc.) show the wild-type-to-mutant ratios of the averaged microarray scores for the given gene. For those genes to which more than one Affy tag correspond, the data for the first one listed under ‘Affy Mouse 430 2.0’ is given. Dark green = downregulated under high stringency (as described in METHODS); light green = downregulated under low stringency; red = upregulated under high stringency; orange = upregulated under low stringency. A dash indicates that the gene was not significantly a [file pone.0000643.s005.zip › Corbo_TableS1/images/4631423F02Rik_TSS.gif]

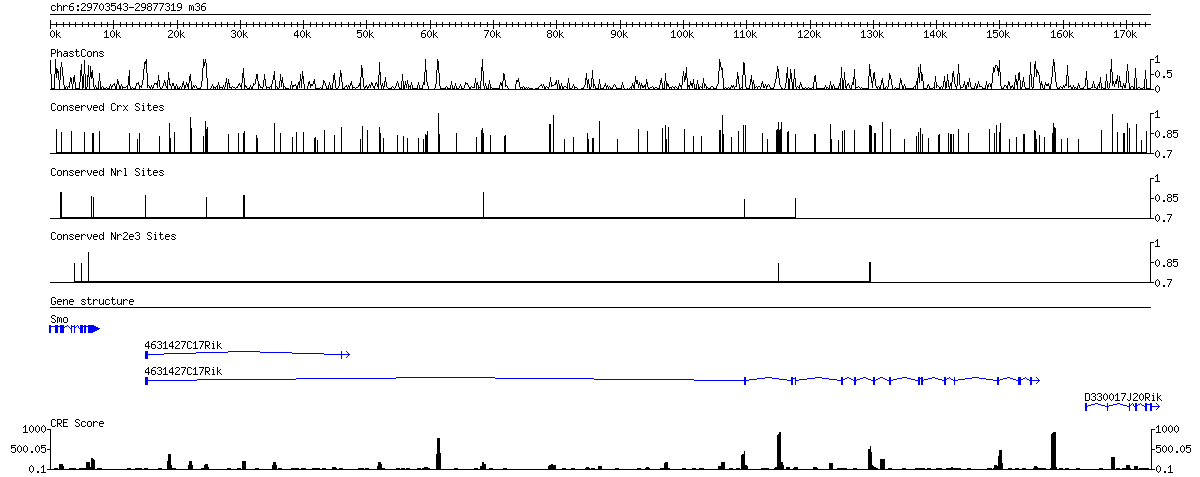

Supplement: Table S1 — Genes in the mouse photoreceptor transcription network. This table is a database which includes all genes dysregulated in Crx-/-, Nrl-/-, and/or Nr2e3-/- under high stringency criteria. Each row in the database represents a gene in the network and includes multiple links to additional types of information about that gene. ‘Large image gene +/− 15 Kb’ links to an image of our computational prediction of photoreceptor CREs in the genomic region 15 Kb upstream and downstream of the gene in question. ‘Closeup image TSS +/− 15 Kb’ links to an image of our computational prediction in the region 15 Kb on either side of the gene's TSS. Black bars within this image highlight the following regulatory peaks (if any are present): the peak closest to the TSS and the peak with the highest CRE score within this 30 Kb window. For those genes whose CRE predictions were tested experimentally, a red bar in this image indicates the location of the CRE that was tested. For Crx and Elovl4 the tested CRE is outside of this 30 Kb window and is therefore indicated in the ‘Large image’. ‘Links’ contains links to the UCSC genome browser, ENSEMBL, and NCBI database entries for the indicated gene, if available. ‘Max score (threshold of 200)’ contains the score of the highest predicted regulatory peak within the 30 Kb window around the gene's TSS. If this window does not contain any predicted peaks ≥ 200 (our cutoff threshold) no value is given (indicated by a dash). The next four columns of the table (‘Nr2e3-/-’ etc.) show the wild-type-to-mutant ratios of the averaged microarray scores for the given gene. For those genes to which more than one Affy tag correspond, the data for the first one listed under ‘Affy Mouse 430 2.0’ is given. Dark green = downregulated under high stringency (as described in METHODS); light green = downregulated under low stringency; red = upregulated under high stringency; orange = upregulated under low stringency. A dash indicates that the gene was not significantly a [file pone.0000643.s005.zip › Corbo_TableS1/images/4631427C17Rik.gif]

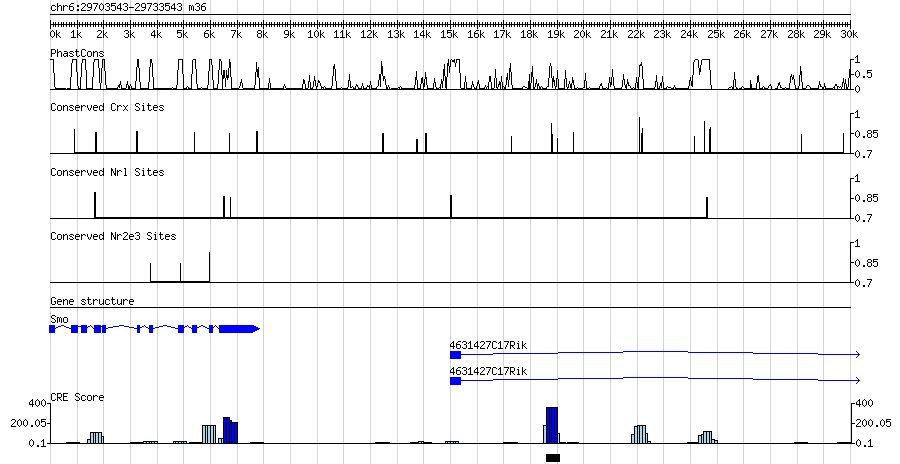

Supplement: Table S1 — Genes in the mouse photoreceptor transcription network. This table is a database which includes all genes dysregulated in Crx-/-, Nrl-/-, and/or Nr2e3-/- under high stringency criteria. Each row in the database represents a gene in the network and includes multiple links to additional types of information about that gene. ‘Large image gene +/− 15 Kb’ links to an image of our computational prediction of photoreceptor CREs in the genomic region 15 Kb upstream and downstream of the gene in question. ‘Closeup image TSS +/− 15 Kb’ links to an image of our computational prediction in the region 15 Kb on either side of the gene's TSS. Black bars within this image highlight the following regulatory peaks (if any are present): the peak closest to the TSS and the peak with the highest CRE score within this 30 Kb window. For those genes whose CRE predictions were tested experimentally, a red bar in this image indicates the location of the CRE that was tested. For Crx and Elovl4 the tested CRE is outside of this 30 Kb window and is therefore indicated in the ‘Large image’. ‘Links’ contains links to the UCSC genome browser, ENSEMBL, and NCBI database entries for the indicated gene, if available. ‘Max score (threshold of 200)’ contains the score of the highest predicted regulatory peak within the 30 Kb window around the gene's TSS. If this window does not contain any predicted peaks ≥ 200 (our cutoff threshold) no value is given (indicated by a dash). The next four columns of the table (‘Nr2e3-/-’ etc.) show the wild-type-to-mutant ratios of the averaged microarray scores for the given gene. For those genes to which more than one Affy tag correspond, the data for the first one listed under ‘Affy Mouse 430 2.0’ is given. Dark green = downregulated under high stringency (as described in METHODS); light green = downregulated under low stringency; red = upregulated under high stringency; orange = upregulated under low stringency. A dash indicates that the gene was not significantly a [file pone.0000643.s005.zip › Corbo_TableS1/images/4631427C17Rik_TSS.gif]

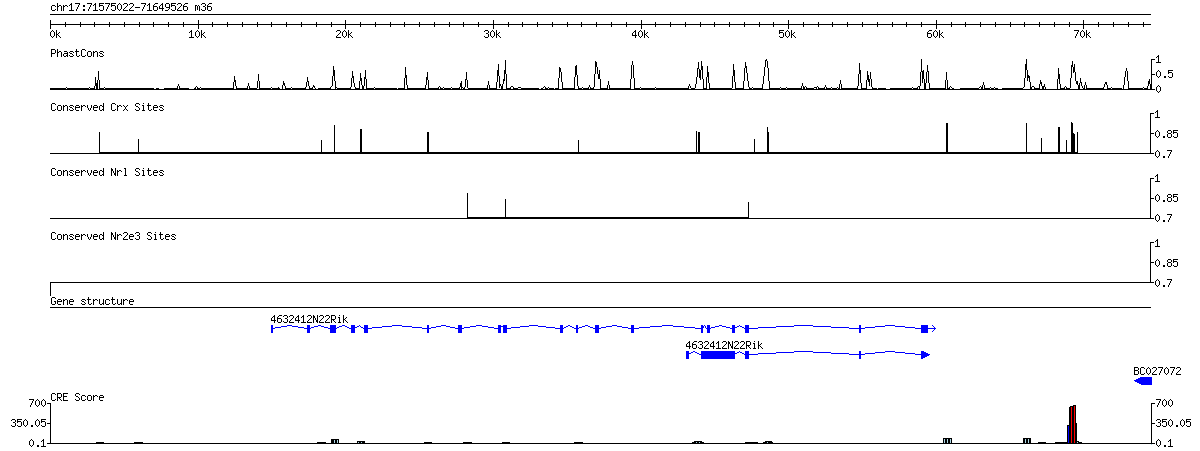

Supplement: Table S1 — Genes in the mouse photoreceptor transcription network. This table is a database which includes all genes dysregulated in Crx-/-, Nrl-/-, and/or Nr2e3-/- under high stringency criteria. Each row in the database represents a gene in the network and includes multiple links to additional types of information about that gene. ‘Large image gene +/− 15 Kb’ links to an image of our computational prediction of photoreceptor CREs in the genomic region 15 Kb upstream and downstream of the gene in question. ‘Closeup image TSS +/− 15 Kb’ links to an image of our computational prediction in the region 15 Kb on either side of the gene's TSS. Black bars within this image highlight the following regulatory peaks (if any are present): the peak closest to the TSS and the peak with the highest CRE score within this 30 Kb window. For those genes whose CRE predictions were tested experimentally, a red bar in this image indicates the location of the CRE that was tested. For Crx and Elovl4 the tested CRE is outside of this 30 Kb window and is therefore indicated in the ‘Large image’. ‘Links’ contains links to the UCSC genome browser, ENSEMBL, and NCBI database entries for the indicated gene, if available. ‘Max score (threshold of 200)’ contains the score of the highest predicted regulatory peak within the 30 Kb window around the gene's TSS. If this window does not contain any predicted peaks ≥ 200 (our cutoff threshold) no value is given (indicated by a dash). The next four columns of the table (‘Nr2e3-/-’ etc.) show the wild-type-to-mutant ratios of the averaged microarray scores for the given gene. For those genes to which more than one Affy tag correspond, the data for the first one listed under ‘Affy Mouse 430 2.0’ is given. Dark green = downregulated under high stringency (as described in METHODS); light green = downregulated under low stringency; red = upregulated under high stringency; orange = upregulated under low stringency. A dash indicates that the gene was not significantly a [file pone.0000643.s005.zip › Corbo_TableS1/images/4632412N22Rik.gif]

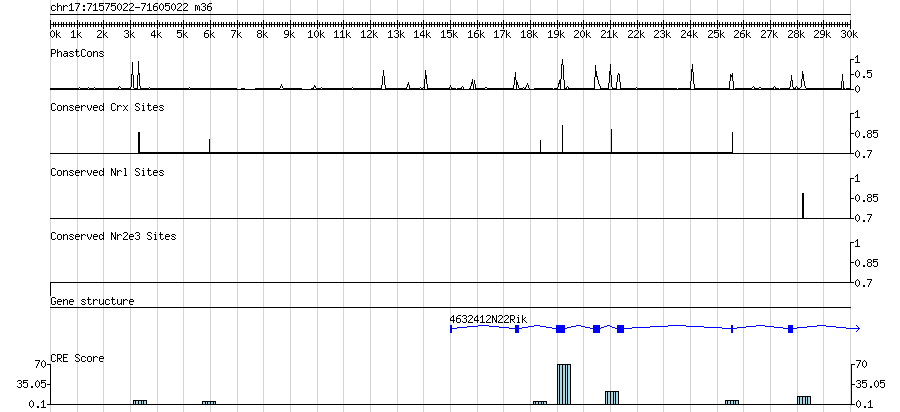

Supplement: Table S1 — Genes in the mouse photoreceptor transcription network. This table is a database which includes all genes dysregulated in Crx-/-, Nrl-/-, and/or Nr2e3-/- under high stringency criteria. Each row in the database represents a gene in the network and includes multiple links to additional types of information about that gene. ‘Large image gene +/− 15 Kb’ links to an image of our computational prediction of photoreceptor CREs in the genomic region 15 Kb upstream and downstream of the gene in question. ‘Closeup image TSS +/− 15 Kb’ links to an image of our computational prediction in the region 15 Kb on either side of the gene's TSS. Black bars within this image highlight the following regulatory peaks (if any are present): the peak closest to the TSS and the peak with the highest CRE score within this 30 Kb window. For those genes whose CRE predictions were tested experimentally, a red bar in this image indicates the location of the CRE that was tested. For Crx and Elovl4 the tested CRE is outside of this 30 Kb window and is therefore indicated in the ‘Large image’. ‘Links’ contains links to the UCSC genome browser, ENSEMBL, and NCBI database entries for the indicated gene, if available. ‘Max score (threshold of 200)’ contains the score of the highest predicted regulatory peak within the 30 Kb window around the gene's TSS. If this window does not contain any predicted peaks ≥ 200 (our cutoff threshold) no value is given (indicated by a dash). The next four columns of the table (‘Nr2e3-/-’ etc.) show the wild-type-to-mutant ratios of the averaged microarray scores for the given gene. For those genes to which more than one Affy tag correspond, the data for the first one listed under ‘Affy Mouse 430 2.0’ is given. Dark green = downregulated under high stringency (as described in METHODS); light green = downregulated under low stringency; red = upregulated under high stringency; orange = upregulated under low stringency. A dash indicates that the gene was not significantly a [file pone.0000643.s005.zip › Corbo_TableS1/images/4632412N22Rik_TSS.gif]

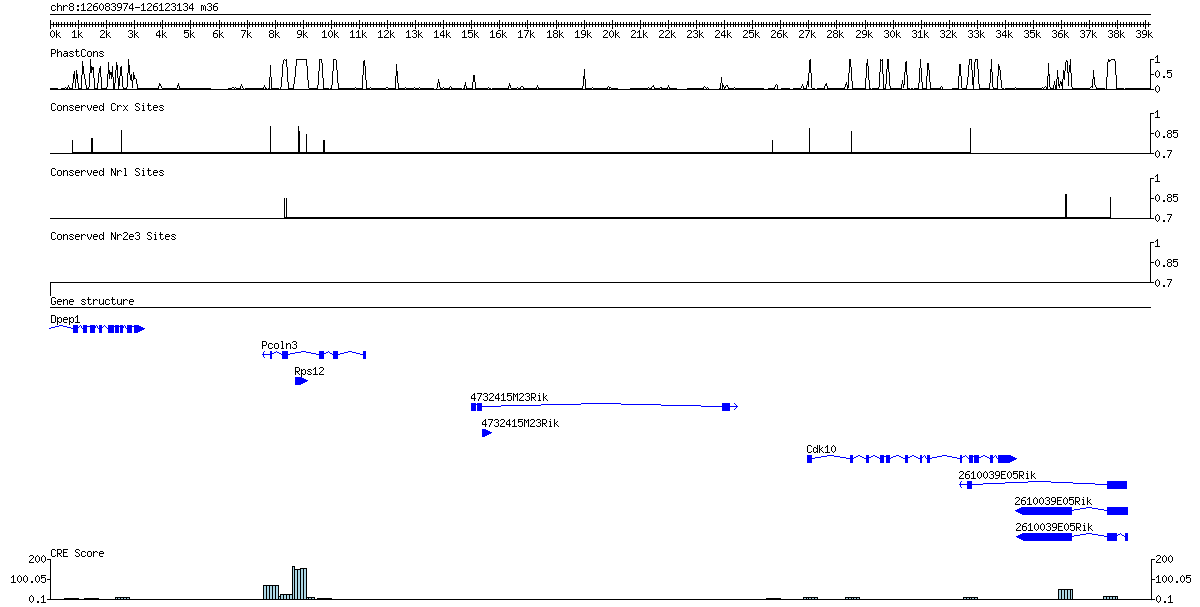

Supplement: Table S1 — Genes in the mouse photoreceptor transcription network. This table is a database which includes all genes dysregulated in Crx-/-, Nrl-/-, and/or Nr2e3-/- under high stringency criteria. Each row in the database represents a gene in the network and includes multiple links to additional types of information about that gene. ‘Large image gene +/− 15 Kb’ links to an image of our computational prediction of photoreceptor CREs in the genomic region 15 Kb upstream and downstream of the gene in question. ‘Closeup image TSS +/− 15 Kb’ links to an image of our computational prediction in the region 15 Kb on either side of the gene's TSS. Black bars within this image highlight the following regulatory peaks (if any are present): the peak closest to the TSS and the peak with the highest CRE score within this 30 Kb window. For those genes whose CRE predictions were tested experimentally, a red bar in this image indicates the location of the CRE that was tested. For Crx and Elovl4 the tested CRE is outside of this 30 Kb window and is therefore indicated in the ‘Large image’. ‘Links’ contains links to the UCSC genome browser, ENSEMBL, and NCBI database entries for the indicated gene, if available. ‘Max score (threshold of 200)’ contains the score of the highest predicted regulatory peak within the 30 Kb window around the gene's TSS. If this window does not contain any predicted peaks ≥ 200 (our cutoff threshold) no value is given (indicated by a dash). The next four columns of the table (‘Nr2e3-/-’ etc.) show the wild-type-to-mutant ratios of the averaged microarray scores for the given gene. For those genes to which more than one Affy tag correspond, the data for the first one listed under ‘Affy Mouse 430 2.0’ is given. Dark green = downregulated under high stringency (as described in METHODS); light green = downregulated under low stringency; red = upregulated under high stringency; orange = upregulated under low stringency. A dash indicates that the gene was not significantly a [file pone.0000643.s005.zip › Corbo_TableS1/images/4732415M23Rik.gif]

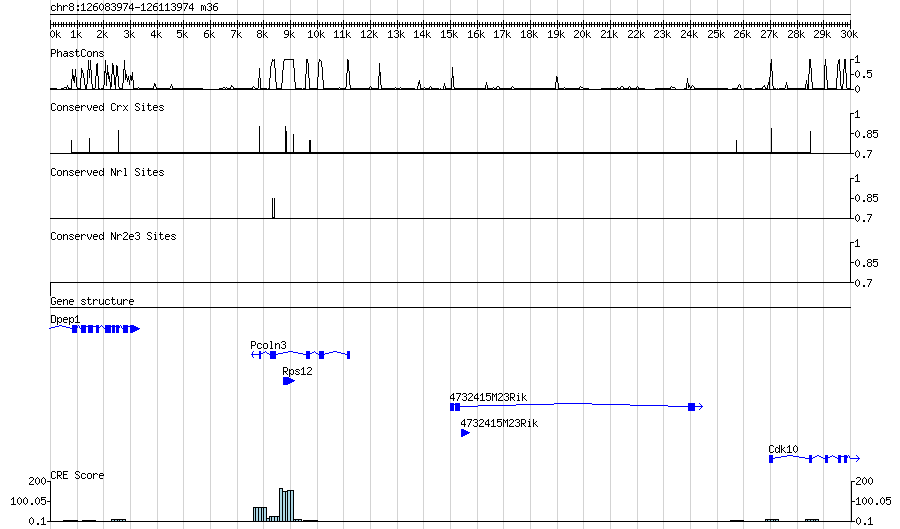

Supplement: Table S1 — Genes in the mouse photoreceptor transcription network. This table is a database which includes all genes dysregulated in Crx-/-, Nrl-/-, and/or Nr2e3-/- under high stringency criteria. Each row in the database represents a gene in the network and includes multiple links to additional types of information about that gene. ‘Large image gene +/− 15 Kb’ links to an image of our computational prediction of photoreceptor CREs in the genomic region 15 Kb upstream and downstream of the gene in question. ‘Closeup image TSS +/− 15 Kb’ links to an image of our computational prediction in the region 15 Kb on either side of the gene's TSS. Black bars within this image highlight the following regulatory peaks (if any are present): the peak closest to the TSS and the peak with the highest CRE score within this 30 Kb window. For those genes whose CRE predictions were tested experimentally, a red bar in this image indicates the location of the CRE that was tested. For Crx and Elovl4 the tested CRE is outside of this 30 Kb window and is therefore indicated in the ‘Large image’. ‘Links’ contains links to the UCSC genome browser, ENSEMBL, and NCBI database entries for the indicated gene, if available. ‘Max score (threshold of 200)’ contains the score of the highest predicted regulatory peak within the 30 Kb window around the gene's TSS. If this window does not contain any predicted peaks ≥ 200 (our cutoff threshold) no value is given (indicated by a dash). The next four columns of the table (‘Nr2e3-/-’ etc.) show the wild-type-to-mutant ratios of the averaged microarray scores for the given gene. For those genes to which more than one Affy tag correspond, the data for the first one listed under ‘Affy Mouse 430 2.0’ is given. Dark green = downregulated under high stringency (as described in METHODS); light green = downregulated under low stringency; red = upregulated under high stringency; orange = upregulated under low stringency. A dash indicates that the gene was not significantly a [file pone.0000643.s005.zip › Corbo_TableS1/images/4732415M23Rik_TSS.gif]

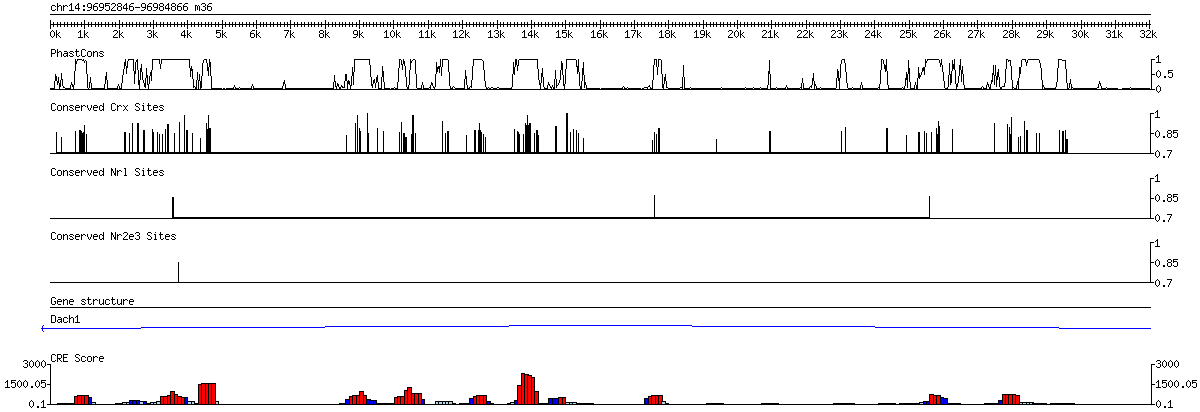

Supplement: Table S1 — Genes in the mouse photoreceptor transcription network. This table is a database which includes all genes dysregulated in Crx-/-, Nrl-/-, and/or Nr2e3-/- under high stringency criteria. Each row in the database represents a gene in the network and includes multiple links to additional types of information about that gene. ‘Large image gene +/− 15 Kb’ links to an image of our computational prediction of photoreceptor CREs in the genomic region 15 Kb upstream and downstream of the gene in question. ‘Closeup image TSS +/− 15 Kb’ links to an image of our computational prediction in the region 15 Kb on either side of the gene's TSS. Black bars within this image highlight the following regulatory peaks (if any are present): the peak closest to the TSS and the peak with the highest CRE score within this 30 Kb window. For those genes whose CRE predictions were tested experimentally, a red bar in this image indicates the location of the CRE that was tested. For Crx and Elovl4 the tested CRE is outside of this 30 Kb window and is therefore indicated in the ‘Large image’. ‘Links’ contains links to the UCSC genome browser, ENSEMBL, and NCBI database entries for the indicated gene, if available. ‘Max score (threshold of 200)’ contains the score of the highest predicted regulatory peak within the 30 Kb window around the gene's TSS. If this window does not contain any predicted peaks ≥ 200 (our cutoff threshold) no value is given (indicated by a dash). The next four columns of the table (‘Nr2e3-/-’ etc.) show the wild-type-to-mutant ratios of the averaged microarray scores for the given gene. For those genes to which more than one Affy tag correspond, the data for the first one listed under ‘Affy Mouse 430 2.0’ is given. Dark green = downregulated under high stringency (as described in METHODS); light green = downregulated under low stringency; red = upregulated under high stringency; orange = upregulated under low stringency. A dash indicates that the gene was not significantly a [file pone.0000643.s005.zip › Corbo_TableS1/images/4833428M15Rik.gif]

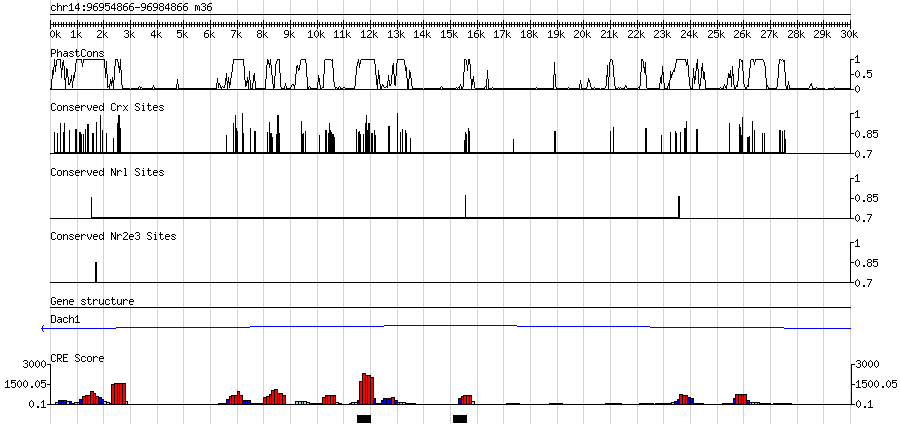

Supplement: Table S1 — Genes in the mouse photoreceptor transcription network. This table is a database which includes all genes dysregulated in Crx-/-, Nrl-/-, and/or Nr2e3-/- under high stringency criteria. Each row in the database represents a gene in the network and includes multiple links to additional types of information about that gene. ‘Large image gene +/− 15 Kb’ links to an image of our computational prediction of photoreceptor CREs in the genomic region 15 Kb upstream and downstream of the gene in question. ‘Closeup image TSS +/− 15 Kb’ links to an image of our computational prediction in the region 15 Kb on either side of the gene's TSS. Black bars within this image highlight the following regulatory peaks (if any are present): the peak closest to the TSS and the peak with the highest CRE score within this 30 Kb window. For those genes whose CRE predictions were tested experimentally, a red bar in this image indicates the location of the CRE that was tested. For Crx and Elovl4 the tested CRE is outside of this 30 Kb window and is therefore indicated in the ‘Large image’. ‘Links’ contains links to the UCSC genome browser, ENSEMBL, and NCBI database entries for the indicated gene, if available. ‘Max score (threshold of 200)’ contains the score of the highest predicted regulatory peak within the 30 Kb window around the gene's TSS. If this window does not contain any predicted peaks ≥ 200 (our cutoff threshold) no value is given (indicated by a dash). The next four columns of the table (‘Nr2e3-/-’ etc.) show the wild-type-to-mutant ratios of the averaged microarray scores for the given gene. For those genes to which more than one Affy tag correspond, the data for the first one listed under ‘Affy Mouse 430 2.0’ is given. Dark green = downregulated under high stringency (as described in METHODS); light green = downregulated under low stringency; red = upregulated under high stringency; orange = upregulated under low stringency. A dash indicates that the gene was not significantly a [file pone.0000643.s005.zip › Corbo_TableS1/images/4833428M15Rik_TSS.gif]

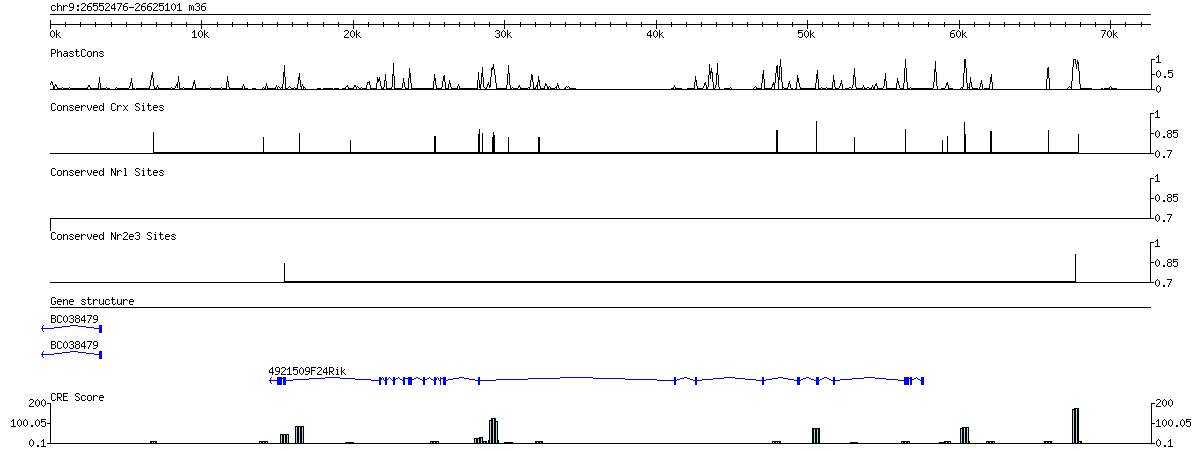

Supplement: Table S1 — Genes in the mouse photoreceptor transcription network. This table is a database which includes all genes dysregulated in Crx-/-, Nrl-/-, and/or Nr2e3-/- under high stringency criteria. Each row in the database represents a gene in the network and includes multiple links to additional types of information about that gene. ‘Large image gene +/− 15 Kb’ links to an image of our computational prediction of photoreceptor CREs in the genomic region 15 Kb upstream and downstream of the gene in question. ‘Closeup image TSS +/− 15 Kb’ links to an image of our computational prediction in the region 15 Kb on either side of the gene's TSS. Black bars within this image highlight the following regulatory peaks (if any are present): the peak closest to the TSS and the peak with the highest CRE score within this 30 Kb window. For those genes whose CRE predictions were tested experimentally, a red bar in this image indicates the location of the CRE that was tested. For Crx and Elovl4 the tested CRE is outside of this 30 Kb window and is therefore indicated in the ‘Large image’. ‘Links’ contains links to the UCSC genome browser, ENSEMBL, and NCBI database entries for the indicated gene, if available. ‘Max score (threshold of 200)’ contains the score of the highest predicted regulatory peak within the 30 Kb window around the gene's TSS. If this window does not contain any predicted peaks ≥ 200 (our cutoff threshold) no value is given (indicated by a dash). The next four columns of the table (‘Nr2e3-/-’ etc.) show the wild-type-to-mutant ratios of the averaged microarray scores for the given gene. For those genes to which more than one Affy tag correspond, the data for the first one listed under ‘Affy Mouse 430 2.0’ is given. Dark green = downregulated under high stringency (as described in METHODS); light green = downregulated under low stringency; red = upregulated under high stringency; orange = upregulated under low stringency. A dash indicates that the gene was not significantly a [file pone.0000643.s005.zip › Corbo_TableS1/images/4921509F24Rik.gif]

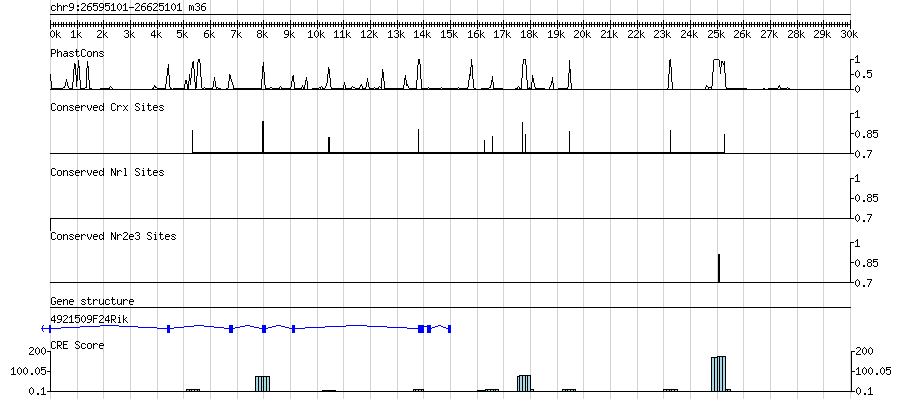

Supplement: Table S1 — Genes in the mouse photoreceptor transcription network. This table is a database which includes all genes dysregulated in Crx-/-, Nrl-/-, and/or Nr2e3-/- under high stringency criteria. Each row in the database represents a gene in the network and includes multiple links to additional types of information about that gene. ‘Large image gene +/− 15 Kb’ links to an image of our computational prediction of photoreceptor CREs in the genomic region 15 Kb upstream and downstream of the gene in question. ‘Closeup image TSS +/− 15 Kb’ links to an image of our computational prediction in the region 15 Kb on either side of the gene's TSS. Black bars within this image highlight the following regulatory peaks (if any are present): the peak closest to the TSS and the peak with the highest CRE score within this 30 Kb window. For those genes whose CRE predictions were tested experimentally, a red bar in this image indicates the location of the CRE that was tested. For Crx and Elovl4 the tested CRE is outside of this 30 Kb window and is therefore indicated in the ‘Large image’. ‘Links’ contains links to the UCSC genome browser, ENSEMBL, and NCBI database entries for the indicated gene, if available. ‘Max score (threshold of 200)’ contains the score of the highest predicted regulatory peak within the 30 Kb window around the gene's TSS. If this window does not contain any predicted peaks ≥ 200 (our cutoff threshold) no value is given (indicated by a dash). The next four columns of the table (‘Nr2e3-/-’ etc.) show the wild-type-to-mutant ratios of the averaged microarray scores for the given gene. For those genes to which more than one Affy tag correspond, the data for the first one listed under ‘Affy Mouse 430 2.0’ is given. Dark green = downregulated under high stringency (as described in METHODS); light green = downregulated under low stringency; red = upregulated under high stringency; orange = upregulated under low stringency. A dash indicates that the gene was not significantly a [file pone.0000643.s005.zip › Corbo_TableS1/images/4921509F24Rik_TSS.gif]

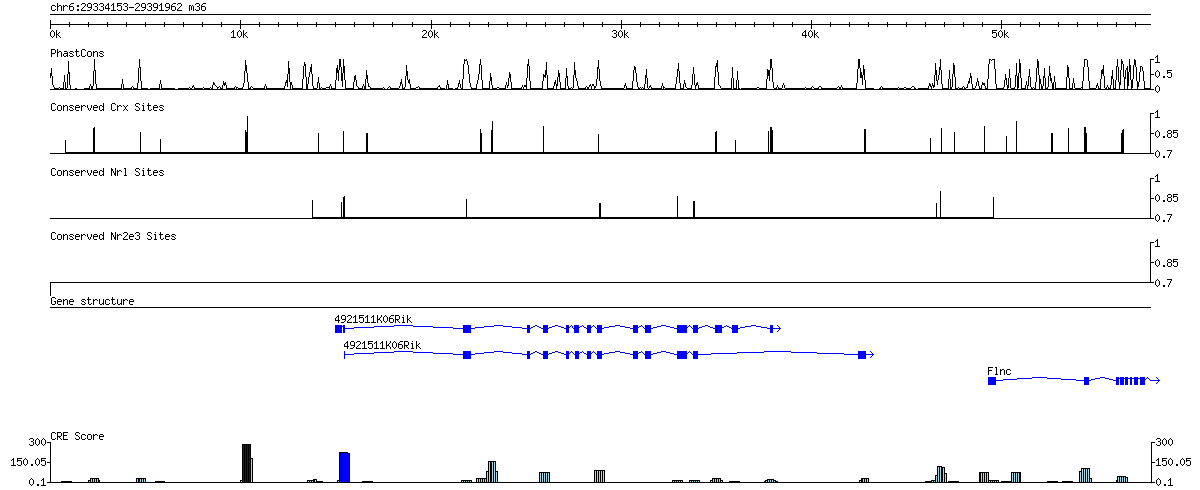

Supplement: Table S1 — Genes in the mouse photoreceptor transcription network. This table is a database which includes all genes dysregulated in Crx-/-, Nrl-/-, and/or Nr2e3-/- under high stringency criteria. Each row in the database represents a gene in the network and includes multiple links to additional types of information about that gene. ‘Large image gene +/− 15 Kb’ links to an image of our computational prediction of photoreceptor CREs in the genomic region 15 Kb upstream and downstream of the gene in question. ‘Closeup image TSS +/− 15 Kb’ links to an image of our computational prediction in the region 15 Kb on either side of the gene's TSS. Black bars within this image highlight the following regulatory peaks (if any are present): the peak closest to the TSS and the peak with the highest CRE score within this 30 Kb window. For those genes whose CRE predictions were tested experimentally, a red bar in this image indicates the location of the CRE that was tested. For Crx and Elovl4 the tested CRE is outside of this 30 Kb window and is therefore indicated in the ‘Large image’. ‘Links’ contains links to the UCSC genome browser, ENSEMBL, and NCBI database entries for the indicated gene, if available. ‘Max score (threshold of 200)’ contains the score of the highest predicted regulatory peak within the 30 Kb window around the gene's TSS. If this window does not contain any predicted peaks ≥ 200 (our cutoff threshold) no value is given (indicated by a dash). The next four columns of the table (‘Nr2e3-/-’ etc.) show the wild-type-to-mutant ratios of the averaged microarray scores for the given gene. For those genes to which more than one Affy tag correspond, the data for the first one listed under ‘Affy Mouse 430 2.0’ is given. Dark green = downregulated under high stringency (as described in METHODS); light green = downregulated under low stringency; red = upregulated under high stringency; orange = upregulated under low stringency. A dash indicates that the gene was not significantly a [file pone.0000643.s005.zip › Corbo_TableS1/images/4921511K06Rik.gif]

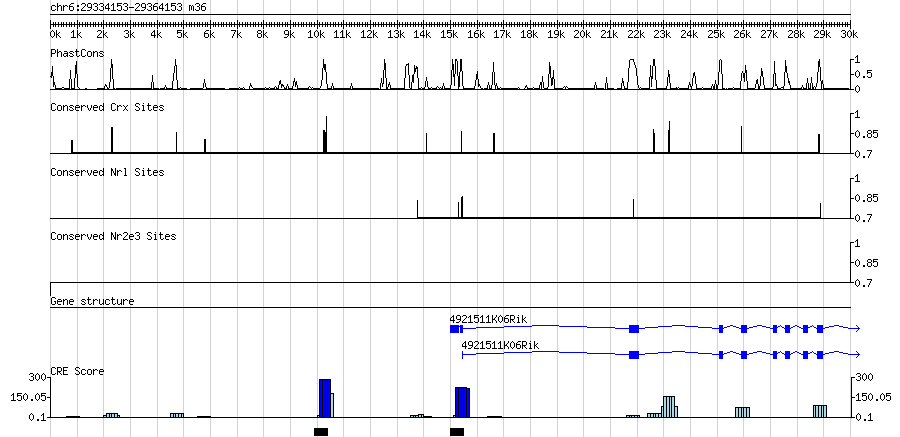

Supplement: Table S1 — Genes in the mouse photoreceptor transcription network. This table is a database which includes all genes dysregulated in Crx-/-, Nrl-/-, and/or Nr2e3-/- under high stringency criteria. Each row in the database represents a gene in the network and includes multiple links to additional types of information about that gene. ‘Large image gene +/− 15 Kb’ links to an image of our computational prediction of photoreceptor CREs in the genomic region 15 Kb upstream and downstream of the gene in question. ‘Closeup image TSS +/− 15 Kb’ links to an image of our computational prediction in the region 15 Kb on either side of the gene's TSS. Black bars within this image highlight the following regulatory peaks (if any are present): the peak closest to the TSS and the peak with the highest CRE score within this 30 Kb window. For those genes whose CRE predictions were tested experimentally, a red bar in this image indicates the location of the CRE that was tested. For Crx and Elovl4 the tested CRE is outside of this 30 Kb window and is therefore indicated in the ‘Large image’. ‘Links’ contains links to the UCSC genome browser, ENSEMBL, and NCBI database entries for the indicated gene, if available. ‘Max score (threshold of 200)’ contains the score of the highest predicted regulatory peak within the 30 Kb window around the gene's TSS. If this window does not contain any predicted peaks ≥ 200 (our cutoff threshold) no value is given (indicated by a dash). The next four columns of the table (‘Nr2e3-/-’ etc.) show the wild-type-to-mutant ratios of the averaged microarray scores for the given gene. For those genes to which more than one Affy tag correspond, the data for the first one listed under ‘Affy Mouse 430 2.0’ is given. Dark green = downregulated under high stringency (as described in METHODS); light green = downregulated under low stringency; red = upregulated under high stringency; orange = upregulated under low stringency. A dash indicates that the gene was not significantly a [file pone.0000643.s005.zip › Corbo_TableS1/images/4921511K06Rik_TSS.gif]

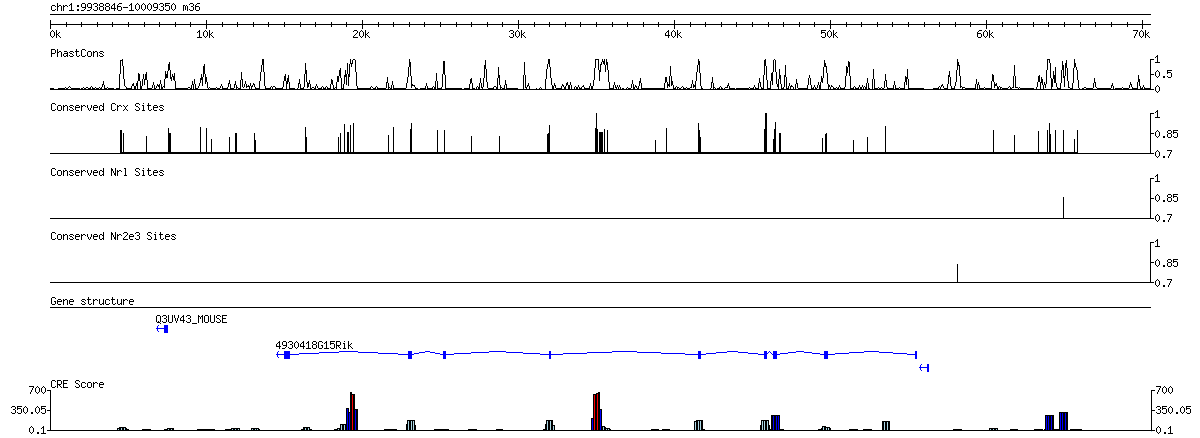

Supplement: Table S1 — Genes in the mouse photoreceptor transcription network. This table is a database which includes all genes dysregulated in Crx-/-, Nrl-/-, and/or Nr2e3-/- under high stringency criteria. Each row in the database represents a gene in the network and includes multiple links to additional types of information about that gene. ‘Large image gene +/− 15 Kb’ links to an image of our computational prediction of photoreceptor CREs in the genomic region 15 Kb upstream and downstream of the gene in question. ‘Closeup image TSS +/− 15 Kb’ links to an image of our computational prediction in the region 15 Kb on either side of the gene's TSS. Black bars within this image highlight the following regulatory peaks (if any are present): the peak closest to the TSS and the peak with the highest CRE score within this 30 Kb window. For those genes whose CRE predictions were tested experimentally, a red bar in this image indicates the location of the CRE that was tested. For Crx and Elovl4 the tested CRE is outside of this 30 Kb window and is therefore indicated in the ‘Large image’. ‘Links’ contains links to the UCSC genome browser, ENSEMBL, and NCBI database entries for the indicated gene, if available. ‘Max score (threshold of 200)’ contains the score of the highest predicted regulatory peak within the 30 Kb window around the gene's TSS. If this window does not contain any predicted peaks ≥ 200 (our cutoff threshold) no value is given (indicated by a dash). The next four columns of the table (‘Nr2e3-/-’ etc.) show the wild-type-to-mutant ratios of the averaged microarray scores for the given gene. For those genes to which more than one Affy tag correspond, the data for the first one listed under ‘Affy Mouse 430 2.0’ is given. Dark green = downregulated under high stringency (as described in METHODS); light green = downregulated under low stringency; red = upregulated under high stringency; orange = upregulated under low stringency. A dash indicates that the gene was not significantly a [file pone.0000643.s005.zip › Corbo_TableS1/images/4930418G15Rik.gif]

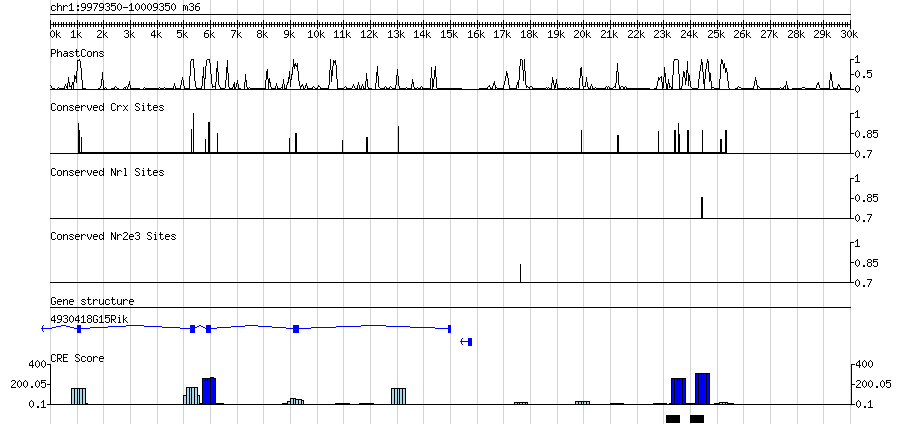

Supplement: Table S1 — Genes in the mouse photoreceptor transcription network. This table is a database which includes all genes dysregulated in Crx-/-, Nrl-/-, and/or Nr2e3-/- under high stringency criteria. Each row in the database represents a gene in the network and includes multiple links to additional types of information about that gene. ‘Large image gene +/− 15 Kb’ links to an image of our computational prediction of photoreceptor CREs in the genomic region 15 Kb upstream and downstream of the gene in question. ‘Closeup image TSS +/− 15 Kb’ links to an image of our computational prediction in the region 15 Kb on either side of the gene's TSS. Black bars within this image highlight the following regulatory peaks (if any are present): the peak closest to the TSS and the peak with the highest CRE score within this 30 Kb window. For those genes whose CRE predictions were tested experimentally, a red bar in this image indicates the location of the CRE that was tested. For Crx and Elovl4 the tested CRE is outside of this 30 Kb window and is therefore indicated in the ‘Large image’. ‘Links’ contains links to the UCSC genome browser, ENSEMBL, and NCBI database entries for the indicated gene, if available. ‘Max score (threshold of 200)’ contains the score of the highest predicted regulatory peak within the 30 Kb window around the gene's TSS. If this window does not contain any predicted peaks ≥ 200 (our cutoff threshold) no value is given (indicated by a dash). The next four columns of the table (‘Nr2e3-/-’ etc.) show the wild-type-to-mutant ratios of the averaged microarray scores for the given gene. For those genes to which more than one Affy tag correspond, the data for the first one listed under ‘Affy Mouse 430 2.0’ is given. Dark green = downregulated under high stringency (as described in METHODS); light green = downregulated under low stringency; red = upregulated under high stringency; orange = upregulated under low stringency. A dash indicates that the gene was not significantly a [file pone.0000643.s005.zip › Corbo_TableS1/images/4930418G15Rik_TSS.gif]

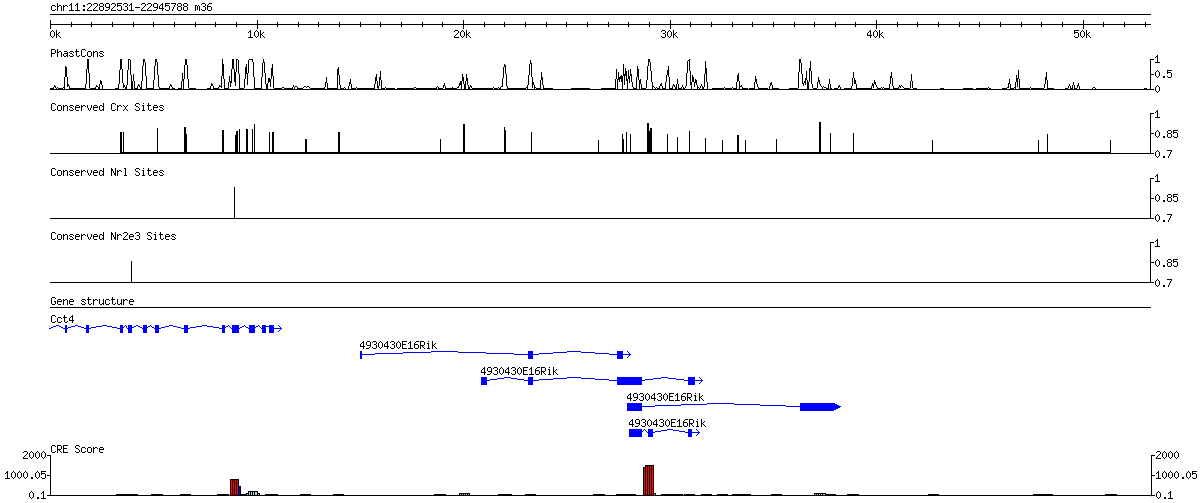

Supplement: Table S1 — Genes in the mouse photoreceptor transcription network. This table is a database which includes all genes dysregulated in Crx-/-, Nrl-/-, and/or Nr2e3-/- under high stringency criteria. Each row in the database represents a gene in the network and includes multiple links to additional types of information about that gene. ‘Large image gene +/− 15 Kb’ links to an image of our computational prediction of photoreceptor CREs in the genomic region 15 Kb upstream and downstream of the gene in question. ‘Closeup image TSS +/− 15 Kb’ links to an image of our computational prediction in the region 15 Kb on either side of the gene's TSS. Black bars within this image highlight the following regulatory peaks (if any are present): the peak closest to the TSS and the peak with the highest CRE score within this 30 Kb window. For those genes whose CRE predictions were tested experimentally, a red bar in this image indicates the location of the CRE that was tested. For Crx and Elovl4 the tested CRE is outside of this 30 Kb window and is therefore indicated in the ‘Large image’. ‘Links’ contains links to the UCSC genome browser, ENSEMBL, and NCBI database entries for the indicated gene, if available. ‘Max score (threshold of 200)’ contains the score of the highest predicted regulatory peak within the 30 Kb window around the gene's TSS. If this window does not contain any predicted peaks ≥ 200 (our cutoff threshold) no value is given (indicated by a dash). The next four columns of the table (‘Nr2e3-/-’ etc.) show the wild-type-to-mutant ratios of the averaged microarray scores for the given gene. For those genes to which more than one Affy tag correspond, the data for the first one listed under ‘Affy Mouse 430 2.0’ is given. Dark green = downregulated under high stringency (as described in METHODS); light green = downregulated under low stringency; red = upregulated under high stringency; orange = upregulated under low stringency. A dash indicates that the gene was not significantly a [file pone.0000643.s005.zip › Corbo_TableS1/images/4930430E16Rik.gif]

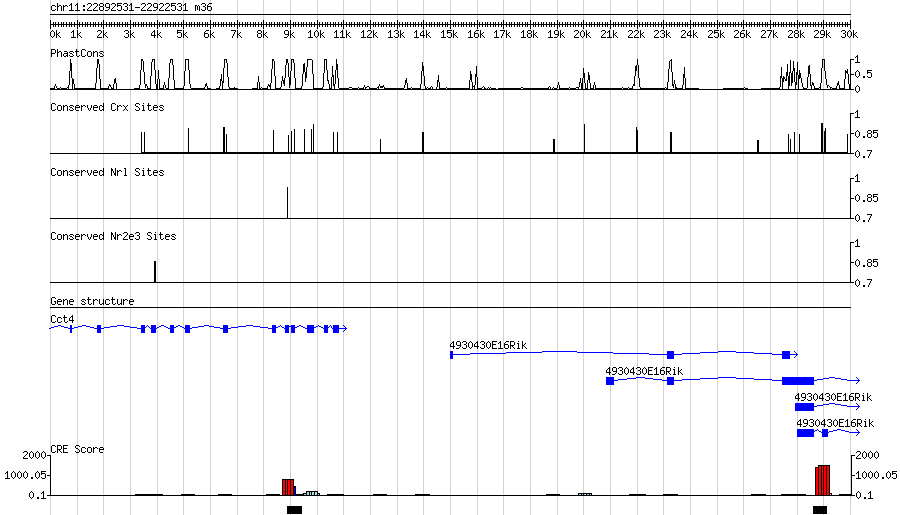

Supplement: Table S1 — Genes in the mouse photoreceptor transcription network. This table is a database which includes all genes dysregulated in Crx-/-, Nrl-/-, and/or Nr2e3-/- under high stringency criteria. Each row in the database represents a gene in the network and includes multiple links to additional types of information about that gene. ‘Large image gene +/− 15 Kb’ links to an image of our computational prediction of photoreceptor CREs in the genomic region 15 Kb upstream and downstream of the gene in question. ‘Closeup image TSS +/− 15 Kb’ links to an image of our computational prediction in the region 15 Kb on either side of the gene's TSS. Black bars within this image highlight the following regulatory peaks (if any are present): the peak closest to the TSS and the peak with the highest CRE score within this 30 Kb window. For those genes whose CRE predictions were tested experimentally, a red bar in this image indicates the location of the CRE that was tested. For Crx and Elovl4 the tested CRE is outside of this 30 Kb window and is therefore indicated in the ‘Large image’. ‘Links’ contains links to the UCSC genome browser, ENSEMBL, and NCBI database entries for the indicated gene, if available. ‘Max score (threshold of 200)’ contains the score of the highest predicted regulatory peak within the 30 Kb window around the gene's TSS. If this window does not contain any predicted peaks ≥ 200 (our cutoff threshold) no value is given (indicated by a dash). The next four columns of the table (‘Nr2e3-/-’ etc.) show the wild-type-to-mutant ratios of the averaged microarray scores for the given gene. For those genes to which more than one Affy tag correspond, the data for the first one listed under ‘Affy Mouse 430 2.0’ is given. Dark green = downregulated under high stringency (as described in METHODS); light green = downregulated under low stringency; red = upregulated under high stringency; orange = upregulated under low stringency. A dash indicates that the gene was not significantly a [file pone.0000643.s005.zip › Corbo_TableS1/images/4930430E16Rik_TSS.gif]

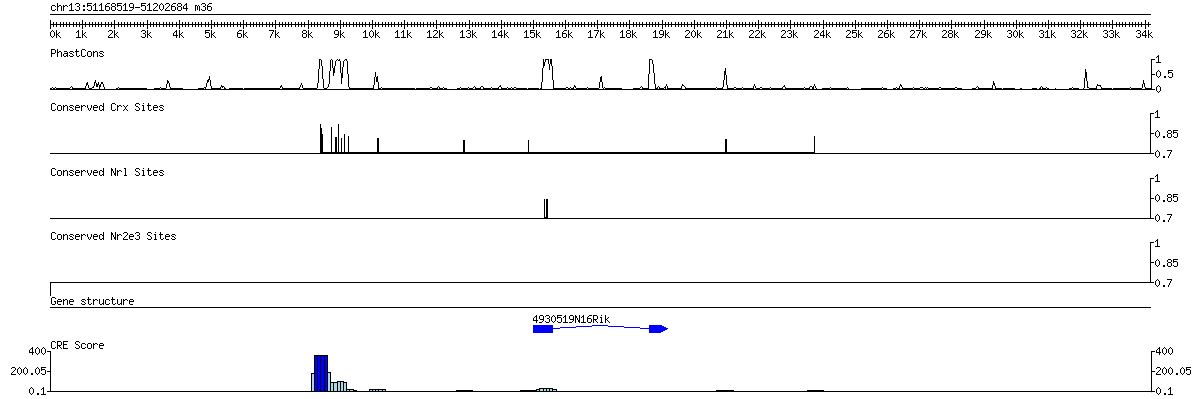

Supplement: Table S1 — Genes in the mouse photoreceptor transcription network. This table is a database which includes all genes dysregulated in Crx-/-, Nrl-/-, and/or Nr2e3-/- under high stringency criteria. Each row in the database represents a gene in the network and includes multiple links to additional types of information about that gene. ‘Large image gene +/− 15 Kb’ links to an image of our computational prediction of photoreceptor CREs in the genomic region 15 Kb upstream and downstream of the gene in question. ‘Closeup image TSS +/− 15 Kb’ links to an image of our computational prediction in the region 15 Kb on either side of the gene's TSS. Black bars within this image highlight the following regulatory peaks (if any are present): the peak closest to the TSS and the peak with the highest CRE score within this 30 Kb window. For those genes whose CRE predictions were tested experimentally, a red bar in this image indicates the location of the CRE that was tested. For Crx and Elovl4 the tested CRE is outside of this 30 Kb window and is therefore indicated in the ‘Large image’. ‘Links’ contains links to the UCSC genome browser, ENSEMBL, and NCBI database entries for the indicated gene, if available. ‘Max score (threshold of 200)’ contains the score of the highest predicted regulatory peak within the 30 Kb window around the gene's TSS. If this window does not contain any predicted peaks ≥ 200 (our cutoff threshold) no value is given (indicated by a dash). The next four columns of the table (‘Nr2e3-/-’ etc.) show the wild-type-to-mutant ratios of the averaged microarray scores for the given gene. For those genes to which more than one Affy tag correspond, the data for the first one listed under ‘Affy Mouse 430 2.0’ is given. Dark green = downregulated under high stringency (as described in METHODS); light green = downregulated under low stringency; red = upregulated under high stringency; orange = upregulated under low stringency. A dash indicates that the gene was not significantly a [file pone.0000643.s005.zip › Corbo_TableS1/images/4930519N16Rik.gif]

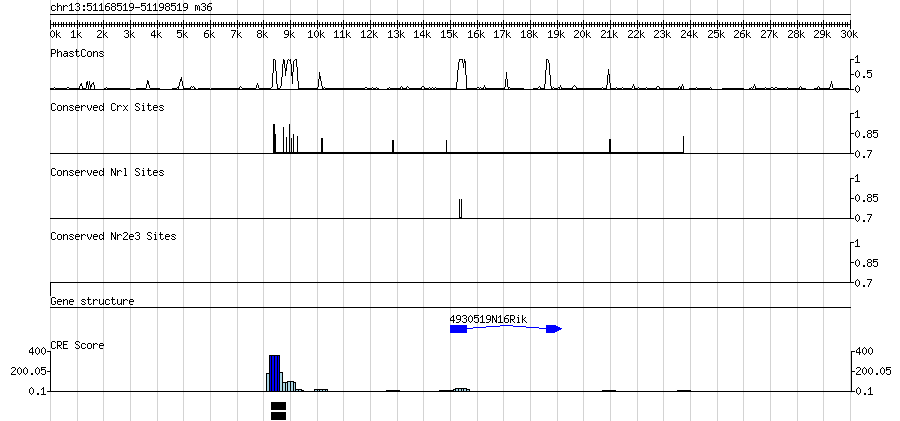

Supplement: Table S1 — Genes in the mouse photoreceptor transcription network. This table is a database which includes all genes dysregulated in Crx-/-, Nrl-/-, and/or Nr2e3-/- under high stringency criteria. Each row in the database represents a gene in the network and includes multiple links to additional types of information about that gene. ‘Large image gene +/− 15 Kb’ links to an image of our computational prediction of photoreceptor CREs in the genomic region 15 Kb upstream and downstream of the gene in question. ‘Closeup image TSS +/− 15 Kb’ links to an image of our computational prediction in the region 15 Kb on either side of the gene's TSS. Black bars within this image highlight the following regulatory peaks (if any are present): the peak closest to the TSS and the peak with the highest CRE score within this 30 Kb window. For those genes whose CRE predictions were tested experimentally, a red bar in this image indicates the location of the CRE that was tested. For Crx and Elovl4 the tested CRE is outside of this 30 Kb window and is therefore indicated in the ‘Large image’. ‘Links’ contains links to the UCSC genome browser, ENSEMBL, and NCBI database entries for the indicated gene, if available. ‘Max score (threshold of 200)’ contains the score of the highest predicted regulatory peak within the 30 Kb window around the gene's TSS. If this window does not contain any predicted peaks ≥ 200 (our cutoff threshold) no value is given (indicated by a dash). The next four columns of the table (‘Nr2e3-/-’ etc.) show the wild-type-to-mutant ratios of the averaged microarray scores for the given gene. For those genes to which more than one Affy tag correspond, the data for the first one listed under ‘Affy Mouse 430 2.0’ is given. Dark green = downregulated under high stringency (as described in METHODS); light green = downregulated under low stringency; red = upregulated under high stringency; orange = upregulated under low stringency. A dash indicates that the gene was not significantly a [file pone.0000643.s005.zip › Corbo_TableS1/images/4930519N16Rik_TSS.gif]

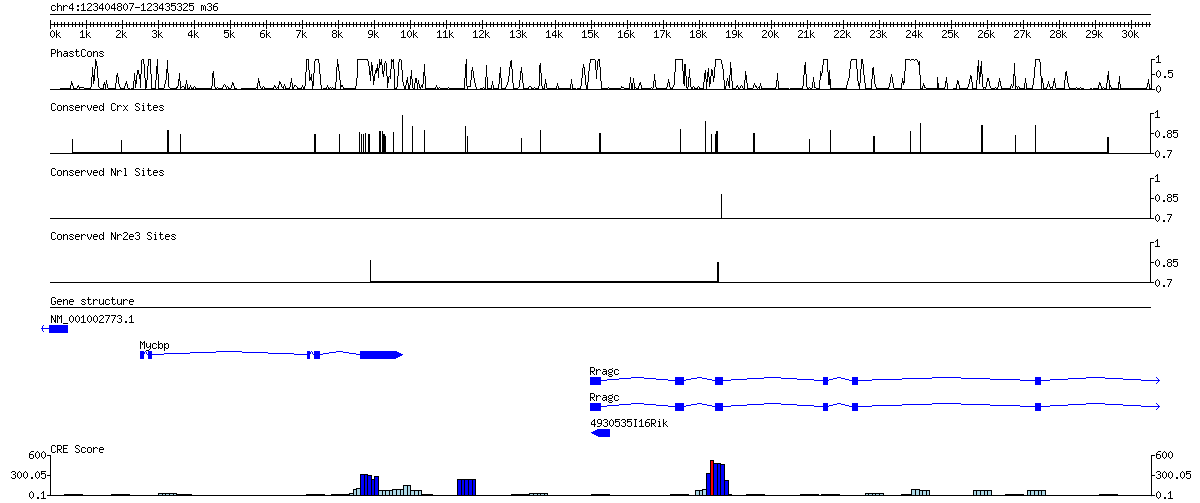

Supplement: Table S1 — Genes in the mouse photoreceptor transcription network. This table is a database which includes all genes dysregulated in Crx-/-, Nrl-/-, and/or Nr2e3-/- under high stringency criteria. Each row in the database represents a gene in the network and includes multiple links to additional types of information about that gene. ‘Large image gene +/− 15 Kb’ links to an image of our computational prediction of photoreceptor CREs in the genomic region 15 Kb upstream and downstream of the gene in question. ‘Closeup image TSS +/− 15 Kb’ links to an image of our computational prediction in the region 15 Kb on either side of the gene's TSS. Black bars within this image highlight the following regulatory peaks (if any are present): the peak closest to the TSS and the peak with the highest CRE score within this 30 Kb window. For those genes whose CRE predictions were tested experimentally, a red bar in this image indicates the location of the CRE that was tested. For Crx and Elovl4 the tested CRE is outside of this 30 Kb window and is therefore indicated in the ‘Large image’. ‘Links’ contains links to the UCSC genome browser, ENSEMBL, and NCBI database entries for the indicated gene, if available. ‘Max score (threshold of 200)’ contains the score of the highest predicted regulatory peak within the 30 Kb window around the gene's TSS. If this window does not contain any predicted peaks ≥ 200 (our cutoff threshold) no value is given (indicated by a dash). The next four columns of the table (‘Nr2e3-/-’ etc.) show the wild-type-to-mutant ratios of the averaged microarray scores for the given gene. For those genes to which more than one Affy tag correspond, the data for the first one listed under ‘Affy Mouse 430 2.0’ is given. Dark green = downregulated under high stringency (as described in METHODS); light green = downregulated under low stringency; red = upregulated under high stringency; orange = upregulated under low stringency. A dash indicates that the gene was not significantly a [file pone.0000643.s005.zip › Corbo_TableS1/images/4930535I16Rik.gif]

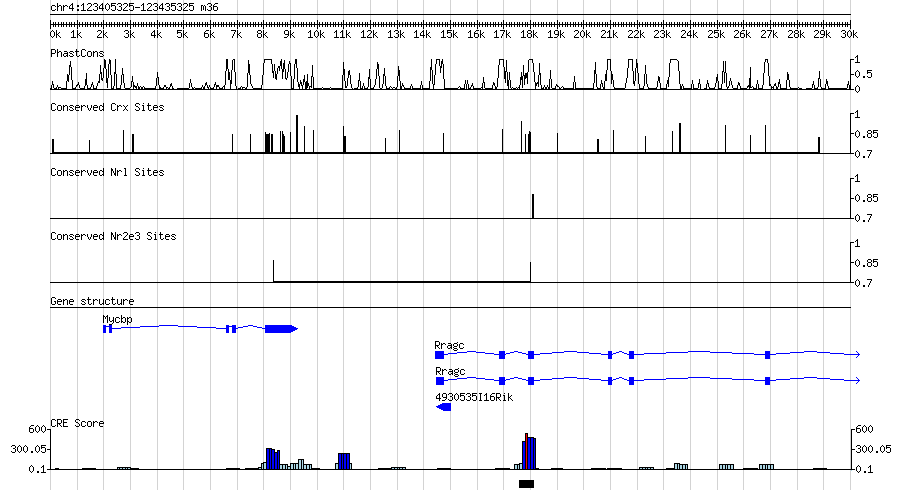

Supplement: Table S1 — Genes in the mouse photoreceptor transcription network. This table is a database which includes all genes dysregulated in Crx-/-, Nrl-/-, and/or Nr2e3-/- under high stringency criteria. Each row in the database represents a gene in the network and includes multiple links to additional types of information about that gene. ‘Large image gene +/− 15 Kb’ links to an image of our computational prediction of photoreceptor CREs in the genomic region 15 Kb upstream and downstream of the gene in question. ‘Closeup image TSS +/− 15 Kb’ links to an image of our computational prediction in the region 15 Kb on either side of the gene's TSS. Black bars within this image highlight the following regulatory peaks (if any are present): the peak closest to the TSS and the peak with the highest CRE score within this 30 Kb window. For those genes whose CRE predictions were tested experimentally, a red bar in this image indicates the location of the CRE that was tested. For Crx and Elovl4 the tested CRE is outside of this 30 Kb window and is therefore indicated in the ‘Large image’. ‘Links’ contains links to the UCSC genome browser, ENSEMBL, and NCBI database entries for the indicated gene, if available. ‘Max score (threshold of 200)’ contains the score of the highest predicted regulatory peak within the 30 Kb window around the gene's TSS. If this window does not contain any predicted peaks ≥ 200 (our cutoff threshold) no value is given (indicated by a dash). The next four columns of the table (‘Nr2e3-/-’ etc.) show the wild-type-to-mutant ratios of the averaged microarray scores for the given gene. For those genes to which more than one Affy tag correspond, the data for the first one listed under ‘Affy Mouse 430 2.0’ is given. Dark green = downregulated under high stringency (as described in METHODS); light green = downregulated under low stringency; red = upregulated under high stringency; orange = upregulated under low stringency. A dash indicates that the gene was not significantly a [file pone.0000643.s005.zip › Corbo_TableS1/images/4930535I16Rik_TSS.gif]

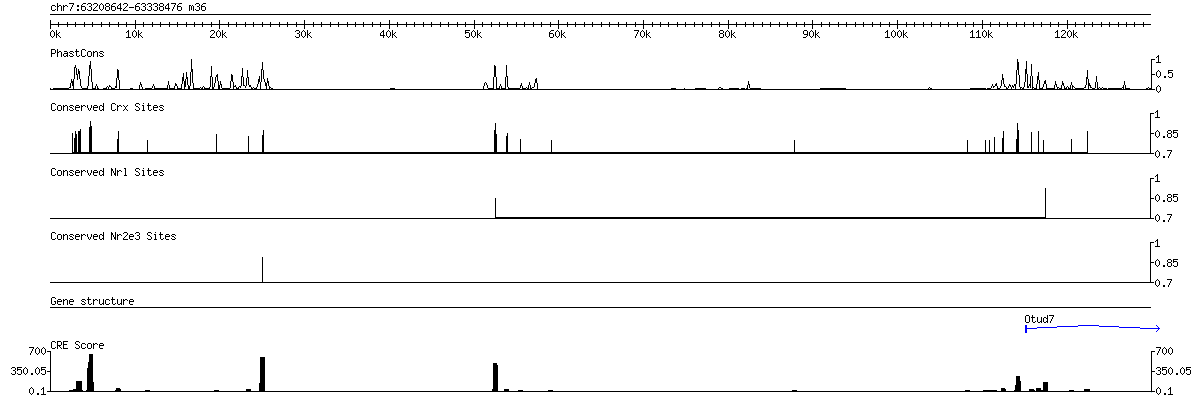

Supplement: Table S1 — Genes in the mouse photoreceptor transcription network. This table is a database which includes all genes dysregulated in Crx-/-, Nrl-/-, and/or Nr2e3-/- under high stringency criteria. Each row in the database represents a gene in the network and includes multiple links to additional types of information about that gene. ‘Large image gene +/− 15 Kb’ links to an image of our computational prediction of photoreceptor CREs in the genomic region 15 Kb upstream and downstream of the gene in question. ‘Closeup image TSS +/− 15 Kb’ links to an image of our computational prediction in the region 15 Kb on either side of the gene's TSS. Black bars within this image highlight the following regulatory peaks (if any are present): the peak closest to the TSS and the peak with the highest CRE score within this 30 Kb window. For those genes whose CRE predictions were tested experimentally, a red bar in this image indicates the location of the CRE that was tested. For Crx and Elovl4 the tested CRE is outside of this 30 Kb window and is therefore indicated in the ‘Large image’. ‘Links’ contains links to the UCSC genome browser, ENSEMBL, and NCBI database entries for the indicated gene, if available. ‘Max score (threshold of 200)’ contains the score of the highest predicted regulatory peak within the 30 Kb window around the gene's TSS. If this window does not contain any predicted peaks ≥ 200 (our cutoff threshold) no value is given (indicated by a dash). The next four columns of the table (‘Nr2e3-/-’ etc.) show the wild-type-to-mutant ratios of the averaged microarray scores for the given gene. For those genes to which more than one Affy tag correspond, the data for the first one listed under ‘Affy Mouse 430 2.0’ is given. Dark green = downregulated under high stringency (as described in METHODS); light green = downregulated under low stringency; red = upregulated under high stringency; orange = upregulated under low stringency. A dash indicates that the gene was not significantly a [file pone.0000643.s005.zip › Corbo_TableS1/images/4930554H23Rik.gif]

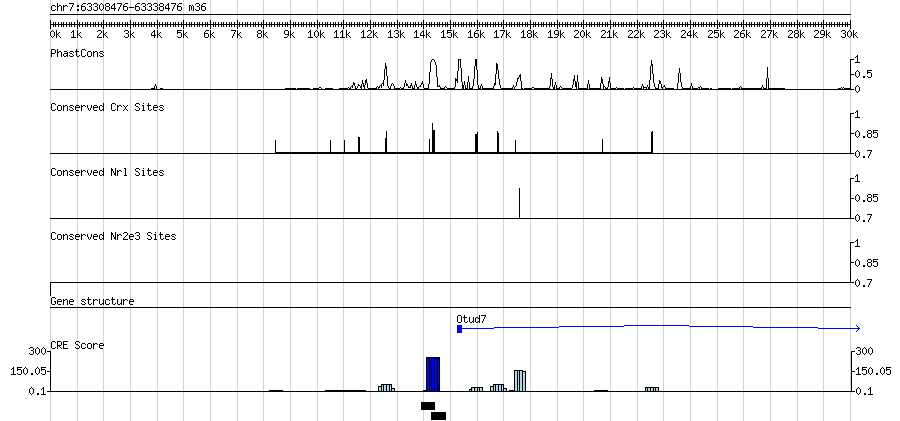

Supplement: Table S1 — Genes in the mouse photoreceptor transcription network. This table is a database which includes all genes dysregulated in Crx-/-, Nrl-/-, and/or Nr2e3-/- under high stringency criteria. Each row in the database represents a gene in the network and includes multiple links to additional types of information about that gene. ‘Large image gene +/− 15 Kb’ links to an image of our computational prediction of photoreceptor CREs in the genomic region 15 Kb upstream and downstream of the gene in question. ‘Closeup image TSS +/− 15 Kb’ links to an image of our computational prediction in the region 15 Kb on either side of the gene's TSS. Black bars within this image highlight the following regulatory peaks (if any are present): the peak closest to the TSS and the peak with the highest CRE score within this 30 Kb window. For those genes whose CRE predictions were tested experimentally, a red bar in this image indicates the location of the CRE that was tested. For Crx and Elovl4 the tested CRE is outside of this 30 Kb window and is therefore indicated in the ‘Large image’. ‘Links’ contains links to the UCSC genome browser, ENSEMBL, and NCBI database entries for the indicated gene, if available. ‘Max score (threshold of 200)’ contains the score of the highest predicted regulatory peak within the 30 Kb window around the gene's TSS. If this window does not contain any predicted peaks ≥ 200 (our cutoff threshold) no value is given (indicated by a dash). The next four columns of the table (‘Nr2e3-/-’ etc.) show the wild-type-to-mutant ratios of the averaged microarray scores for the given gene. For those genes to which more than one Affy tag correspond, the data for the first one listed under ‘Affy Mouse 430 2.0’ is given. Dark green = downregulated under high stringency (as described in METHODS); light green = downregulated under low stringency; red = upregulated under high stringency; orange = upregulated under low stringency. A dash indicates that the gene was not significantly a [file pone.0000643.s005.zip › Corbo_TableS1/images/4930554H23Rik_TSS.gif]

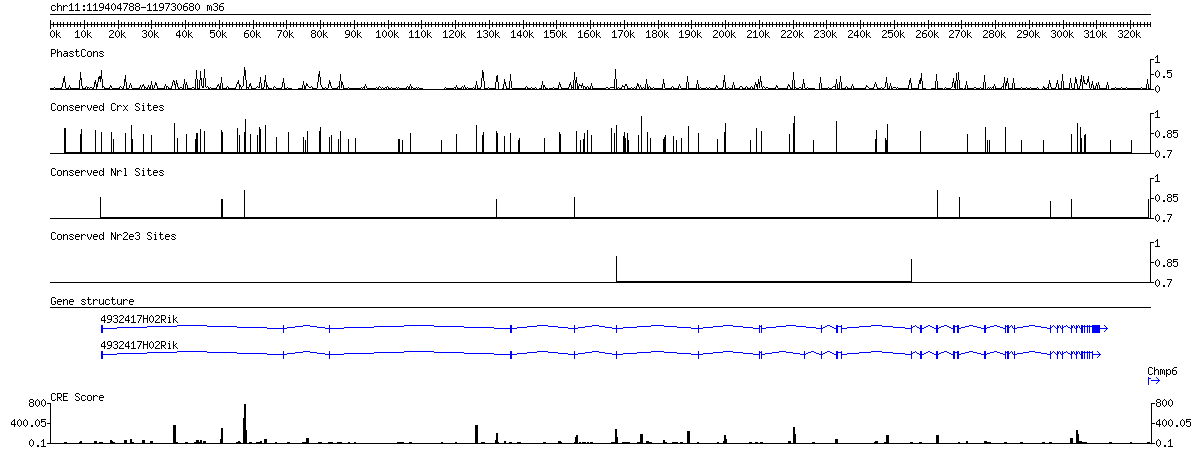

Supplement: Table S1 — Genes in the mouse photoreceptor transcription network. This table is a database which includes all genes dysregulated in Crx-/-, Nrl-/-, and/or Nr2e3-/- under high stringency criteria. Each row in the database represents a gene in the network and includes multiple links to additional types of information about that gene. ‘Large image gene +/− 15 Kb’ links to an image of our computational prediction of photoreceptor CREs in the genomic region 15 Kb upstream and downstream of the gene in question. ‘Closeup image TSS +/− 15 Kb’ links to an image of our computational prediction in the region 15 Kb on either side of the gene's TSS. Black bars within this image highlight the following regulatory peaks (if any are present): the peak closest to the TSS and the peak with the highest CRE score within this 30 Kb window. For those genes whose CRE predictions were tested experimentally, a red bar in this image indicates the location of the CRE that was tested. For Crx and Elovl4 the tested CRE is outside of this 30 Kb window and is therefore indicated in the ‘Large image’. ‘Links’ contains links to the UCSC genome browser, ENSEMBL, and NCBI database entries for the indicated gene, if available. ‘Max score (threshold of 200)’ contains the score of the highest predicted regulatory peak within the 30 Kb window around the gene's TSS. If this window does not contain any predicted peaks ≥ 200 (our cutoff threshold) no value is given (indicated by a dash). The next four columns of the table (‘Nr2e3-/-’ etc.) show the wild-type-to-mutant ratios of the averaged microarray scores for the given gene. For those genes to which more than one Affy tag correspond, the data for the first one listed under ‘Affy Mouse 430 2.0’ is given. Dark green = downregulated under high stringency (as described in METHODS); light green = downregulated under low stringency; red = upregulated under high stringency; orange = upregulated under low stringency. A dash indicates that the gene was not significantly a [file pone.0000643.s005.zip › Corbo_TableS1/images/4932417H02Rik.gif]

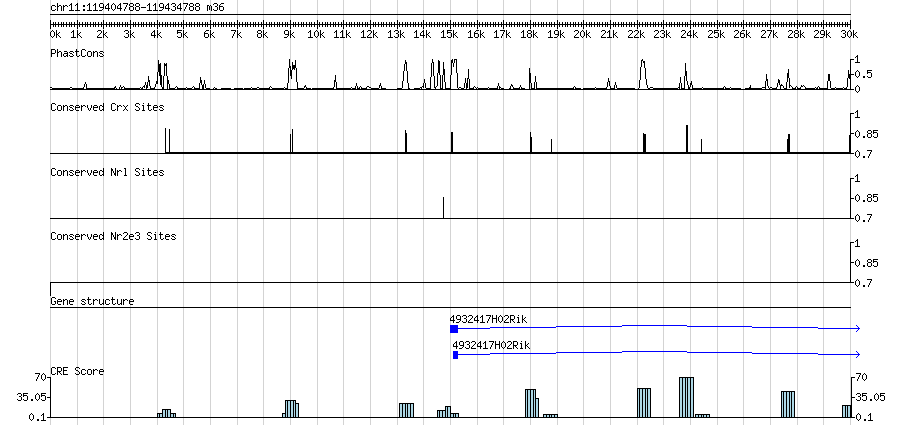

Supplement: Table S1 — Genes in the mouse photoreceptor transcription network. This table is a database which includes all genes dysregulated in Crx-/-, Nrl-/-, and/or Nr2e3-/- under high stringency criteria. Each row in the database represents a gene in the network and includes multiple links to additional types of information about that gene. ‘Large image gene +/− 15 Kb’ links to an image of our computational prediction of photoreceptor CREs in the genomic region 15 Kb upstream and downstream of the gene in question. ‘Closeup image TSS +/− 15 Kb’ links to an image of our computational prediction in the region 15 Kb on either side of the gene's TSS. Black bars within this image highlight the following regulatory peaks (if any are present): the peak closest to the TSS and the peak with the highest CRE score within this 30 Kb window. For those genes whose CRE predictions were tested experimentally, a red bar in this image indicates the location of the CRE that was tested. For Crx and Elovl4 the tested CRE is outside of this 30 Kb window and is therefore indicated in the ‘Large image’. ‘Links’ contains links to the UCSC genome browser, ENSEMBL, and NCBI database entries for the indicated gene, if available. ‘Max score (threshold of 200)’ contains the score of the highest predicted regulatory peak within the 30 Kb window around the gene's TSS. If this window does not contain any predicted peaks ≥ 200 (our cutoff threshold) no value is given (indicated by a dash). The next four columns of the table (‘Nr2e3-/-’ etc.) show the wild-type-to-mutant ratios of the averaged microarray scores for the given gene. For those genes to which more than one Affy tag correspond, the data for the first one listed under ‘Affy Mouse 430 2.0’ is given. Dark green = downregulated under high stringency (as described in METHODS); light green = downregulated under low stringency; red = upregulated under high stringency; orange = upregulated under low stringency. A dash indicates that the gene was not significantly a [file pone.0000643.s005.zip › Corbo_TableS1/images/4932417H02Rik_TSS.gif]

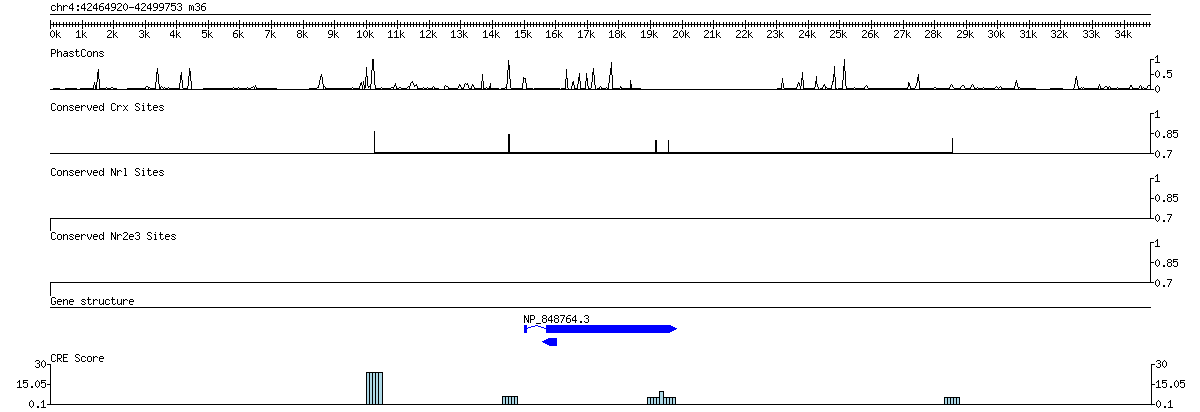

Supplement: Table S1 — Genes in the mouse photoreceptor transcription network. This table is a database which includes all genes dysregulated in Crx-/-, Nrl-/-, and/or Nr2e3-/- under high stringency criteria. Each row in the database represents a gene in the network and includes multiple links to additional types of information about that gene. ‘Large image gene +/− 15 Kb’ links to an image of our computational prediction of photoreceptor CREs in the genomic region 15 Kb upstream and downstream of the gene in question. ‘Closeup image TSS +/− 15 Kb’ links to an image of our computational prediction in the region 15 Kb on either side of the gene's TSS. Black bars within this image highlight the following regulatory peaks (if any are present): the peak closest to the TSS and the peak with the highest CRE score within this 30 Kb window. For those genes whose CRE predictions were tested experimentally, a red bar in this image indicates the location of the CRE that was tested. For Crx and Elovl4 the tested CRE is outside of this 30 Kb window and is therefore indicated in the ‘Large image’. ‘Links’ contains links to the UCSC genome browser, ENSEMBL, and NCBI database entries for the indicated gene, if available. ‘Max score (threshold of 200)’ contains the score of the highest predicted regulatory peak within the 30 Kb window around the gene's TSS. If this window does not contain any predicted peaks ≥ 200 (our cutoff threshold) no value is given (indicated by a dash). The next four columns of the table (‘Nr2e3-/-’ etc.) show the wild-type-to-mutant ratios of the averaged microarray scores for the given gene. For those genes to which more than one Affy tag correspond, the data for the first one listed under ‘Affy Mouse 430 2.0’ is given. Dark green = downregulated under high stringency (as described in METHODS); light green = downregulated under low stringency; red = upregulated under high stringency; orange = upregulated under low stringency. A dash indicates that the gene was not significantly a [file pone.0000643.s005.zip › Corbo_TableS1/images/4933409K07Rik.gif]

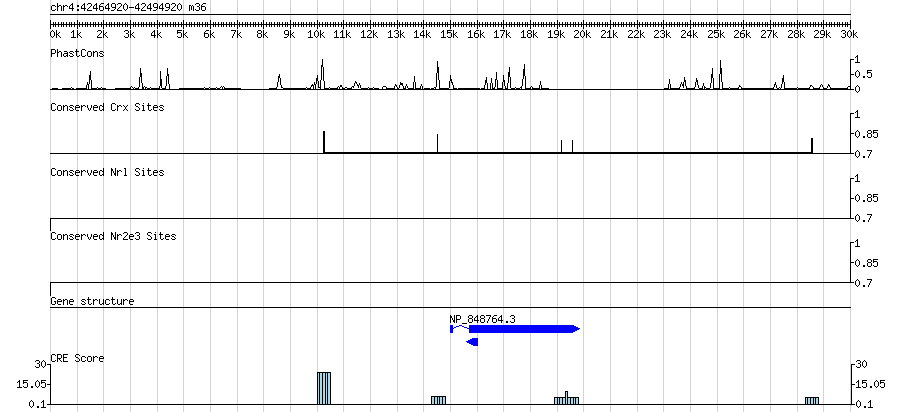

Supplement: Table S1 — Genes in the mouse photoreceptor transcription network. This table is a database which includes all genes dysregulated in Crx-/-, Nrl-/-, and/or Nr2e3-/- under high stringency criteria. Each row in the database represents a gene in the network and includes multiple links to additional types of information about that gene. ‘Large image gene +/− 15 Kb’ links to an image of our computational prediction of photoreceptor CREs in the genomic region 15 Kb upstream and downstream of the gene in question. ‘Closeup image TSS +/− 15 Kb’ links to an image of our computational prediction in the region 15 Kb on either side of the gene's TSS. Black bars within this image highlight the following regulatory peaks (if any are present): the peak closest to the TSS and the peak with the highest CRE score within this 30 Kb window. For those genes whose CRE predictions were tested experimentally, a red bar in this image indicates the location of the CRE that was tested. For Crx and Elovl4 the tested CRE is outside of this 30 Kb window and is therefore indicated in the ‘Large image’. ‘Links’ contains links to the UCSC genome browser, ENSEMBL, and NCBI database entries for the indicated gene, if available. ‘Max score (threshold of 200)’ contains the score of the highest predicted regulatory peak within the 30 Kb window around the gene's TSS. If this window does not contain any predicted peaks ≥ 200 (our cutoff threshold) no value is given (indicated by a dash). The next four columns of the table (‘Nr2e3-/-’ etc.) show the wild-type-to-mutant ratios of the averaged microarray scores for the given gene. For those genes to which more than one Affy tag correspond, the data for the first one listed under ‘Affy Mouse 430 2.0’ is given. Dark green = downregulated under high stringency (as described in METHODS); light green = downregulated under low stringency; red = upregulated under high stringency; orange = upregulated under low stringency. A dash indicates that the gene was not significantly a [file pone.0000643.s005.zip › Corbo_TableS1/images/4933409K07Rik_TSS.gif]

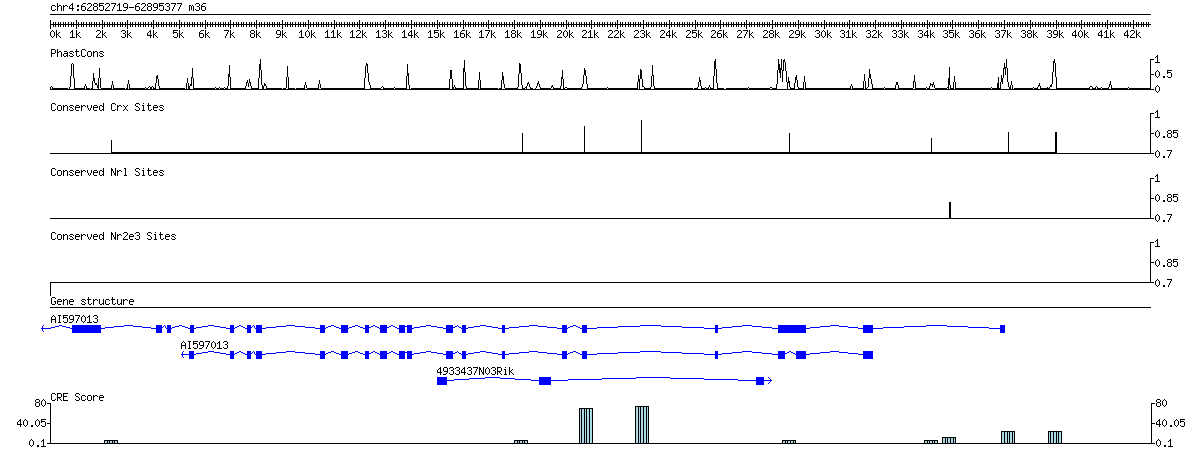

Supplement: Table S1 — Genes in the mouse photoreceptor transcription network. This table is a database which includes all genes dysregulated in Crx-/-, Nrl-/-, and/or Nr2e3-/- under high stringency criteria. Each row in the database represents a gene in the network and includes multiple links to additional types of information about that gene. ‘Large image gene +/− 15 Kb’ links to an image of our computational prediction of photoreceptor CREs in the genomic region 15 Kb upstream and downstream of the gene in question. ‘Closeup image TSS +/− 15 Kb’ links to an image of our computational prediction in the region 15 Kb on either side of the gene's TSS. Black bars within this image highlight the following regulatory peaks (if any are present): the peak closest to the TSS and the peak with the highest CRE score within this 30 Kb window. For those genes whose CRE predictions were tested experimentally, a red bar in this image indicates the location of the CRE that was tested. For Crx and Elovl4 the tested CRE is outside of this 30 Kb window and is therefore indicated in the ‘Large image’. ‘Links’ contains links to the UCSC genome browser, ENSEMBL, and NCBI database entries for the indicated gene, if available. ‘Max score (threshold of 200)’ contains the score of the highest predicted regulatory peak within the 30 Kb window around the gene's TSS. If this window does not contain any predicted peaks ≥ 200 (our cutoff threshold) no value is given (indicated by a dash). The next four columns of the table (‘Nr2e3-/-’ etc.) show the wild-type-to-mutant ratios of the averaged microarray scores for the given gene. For those genes to which more than one Affy tag correspond, the data for the first one listed under ‘Affy Mouse 430 2.0’ is given. Dark green = downregulated under high stringency (as described in METHODS); light green = downregulated under low stringency; red = upregulated under high stringency; orange = upregulated under low stringency. A dash indicates that the gene was not significantly a [file pone.0000643.s005.zip › Corbo_TableS1/images/4933437N03Rik.gif]

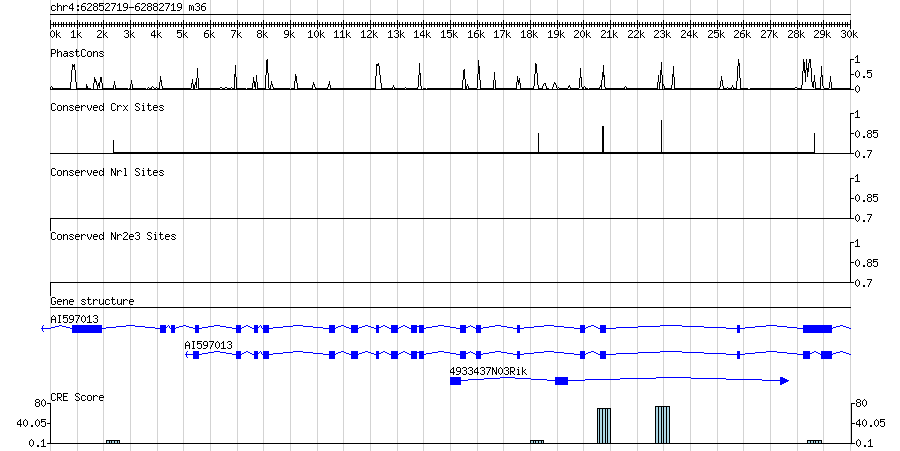

Supplement: Table S1 — Genes in the mouse photoreceptor transcription network. This table is a database which includes all genes dysregulated in Crx-/-, Nrl-/-, and/or Nr2e3-/- under high stringency criteria. Each row in the database represents a gene in the network and includes multiple links to additional types of information about that gene. ‘Large image gene +/− 15 Kb’ links to an image of our computational prediction of photoreceptor CREs in the genomic region 15 Kb upstream and downstream of the gene in question. ‘Closeup image TSS +/− 15 Kb’ links to an image of our computational prediction in the region 15 Kb on either side of the gene's TSS. Black bars within this image highlight the following regulatory peaks (if any are present): the peak closest to the TSS and the peak with the highest CRE score within this 30 Kb window. For those genes whose CRE predictions were tested experimentally, a red bar in this image indicates the location of the CRE that was tested. For Crx and Elovl4 the tested CRE is outside of this 30 Kb window and is therefore indicated in the ‘Large image’. ‘Links’ contains links to the UCSC genome browser, ENSEMBL, and NCBI database entries for the indicated gene, if available. ‘Max score (threshold of 200)’ contains the score of the highest predicted regulatory peak within the 30 Kb window around the gene's TSS. If this window does not contain any predicted peaks ≥ 200 (our cutoff threshold) no value is given (indicated by a dash). The next four columns of the table (‘Nr2e3-/-’ etc.) show the wild-type-to-mutant ratios of the averaged microarray scores for the given gene. For those genes to which more than one Affy tag correspond, the data for the first one listed under ‘Affy Mouse 430 2.0’ is given. Dark green = downregulated under high stringency (as described in METHODS); light green = downregulated under low stringency; red = upregulated under high stringency; orange = upregulated under low stringency. A dash indicates that the gene was not significantly a [file pone.0000643.s005.zip › Corbo_TableS1/images/4933437N03Rik_TSS.gif]

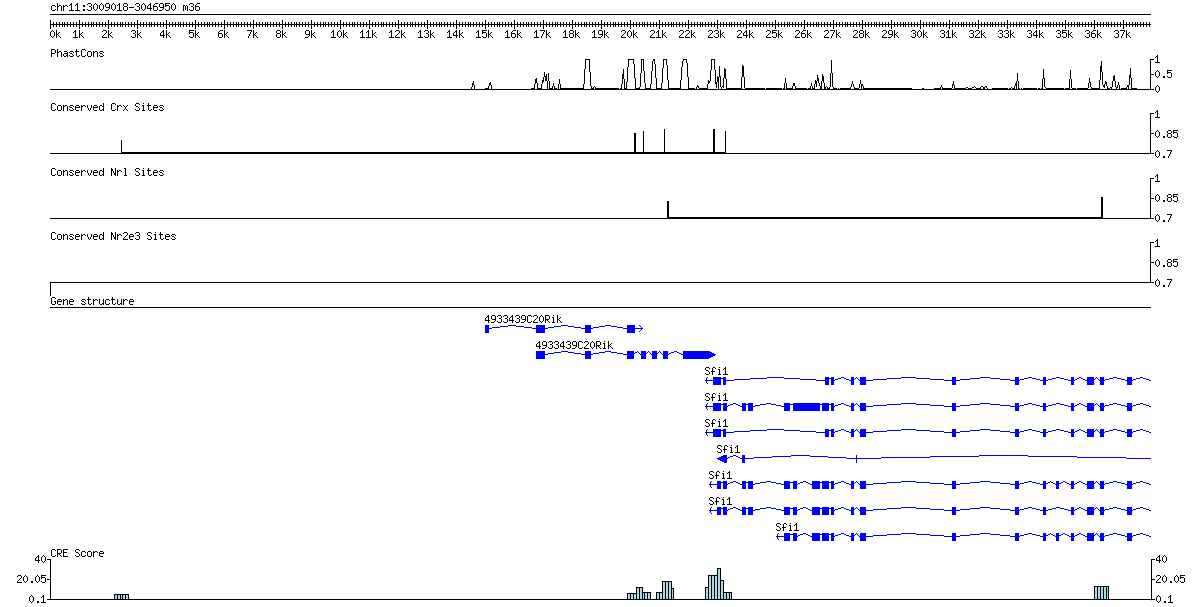

Supplement: Table S1 — Genes in the mouse photoreceptor transcription network. This table is a database which includes all genes dysregulated in Crx-/-, Nrl-/-, and/or Nr2e3-/- under high stringency criteria. Each row in the database represents a gene in the network and includes multiple links to additional types of information about that gene. ‘Large image gene +/− 15 Kb’ links to an image of our computational prediction of photoreceptor CREs in the genomic region 15 Kb upstream and downstream of the gene in question. ‘Closeup image TSS +/− 15 Kb’ links to an image of our computational prediction in the region 15 Kb on either side of the gene's TSS. Black bars within this image highlight the following regulatory peaks (if any are present): the peak closest to the TSS and the peak with the highest CRE score within this 30 Kb window. For those genes whose CRE predictions were tested experimentally, a red bar in this image indicates the location of the CRE that was tested. For Crx and Elovl4 the tested CRE is outside of this 30 Kb window and is therefore indicated in the ‘Large image’. ‘Links’ contains links to the UCSC genome browser, ENSEMBL, and NCBI database entries for the indicated gene, if available. ‘Max score (threshold of 200)’ contains the score of the highest predicted regulatory peak within the 30 Kb window around the gene's TSS. If this window does not contain any predicted peaks ≥ 200 (our cutoff threshold) no value is given (indicated by a dash). The next four columns of the table (‘Nr2e3-/-’ etc.) show the wild-type-to-mutant ratios of the averaged microarray scores for the given gene. For those genes to which more than one Affy tag correspond, the data for the first one listed under ‘Affy Mouse 430 2.0’ is given. Dark green = downregulated under high stringency (as described in METHODS); light green = downregulated under low stringency; red = upregulated under high stringency; orange = upregulated under low stringency. A dash indicates that the gene was not significantly a [file pone.0000643.s005.zip › Corbo_TableS1/images/4933439C20Rik.gif]

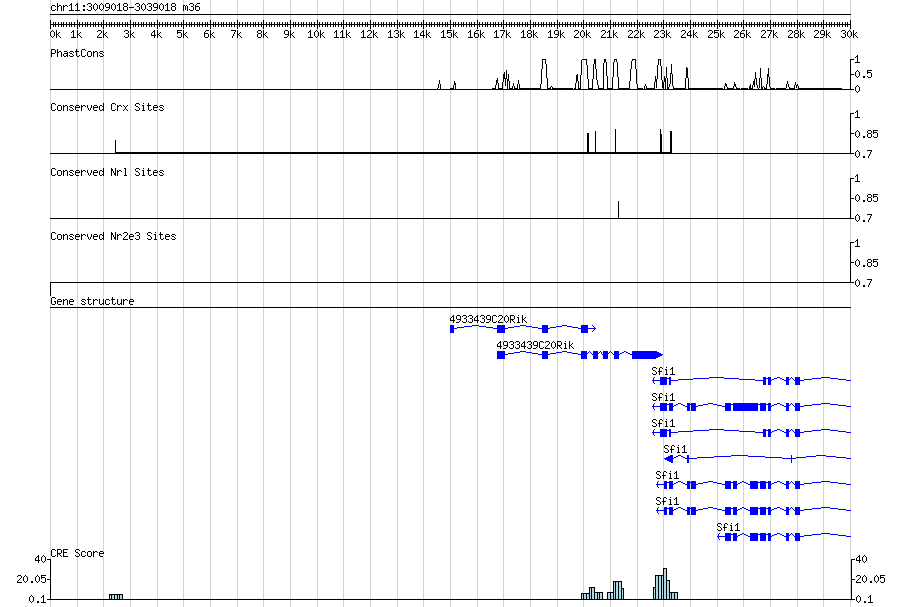

Supplement: Table S1 — Genes in the mouse photoreceptor transcription network. This table is a database which includes all genes dysregulated in Crx-/-, Nrl-/-, and/or Nr2e3-/- under high stringency criteria. Each row in the database represents a gene in the network and includes multiple links to additional types of information about that gene. ‘Large image gene +/− 15 Kb’ links to an image of our computational prediction of photoreceptor CREs in the genomic region 15 Kb upstream and downstream of the gene in question. ‘Closeup image TSS +/− 15 Kb’ links to an image of our computational prediction in the region 15 Kb on either side of the gene's TSS. Black bars within this image highlight the following regulatory peaks (if any are present): the peak closest to the TSS and the peak with the highest CRE score within this 30 Kb window. For those genes whose CRE predictions were tested experimentally, a red bar in this image indicates the location of the CRE that was tested. For Crx and Elovl4 the tested CRE is outside of this 30 Kb window and is therefore indicated in the ‘Large image’. ‘Links’ contains links to the UCSC genome browser, ENSEMBL, and NCBI database entries for the indicated gene, if available. ‘Max score (threshold of 200)’ contains the score of the highest predicted regulatory peak within the 30 Kb window around the gene's TSS. If this window does not contain any predicted peaks ≥ 200 (our cutoff threshold) no value is given (indicated by a dash). The next four columns of the table (‘Nr2e3-/-’ etc.) show the wild-type-to-mutant ratios of the averaged microarray scores for the given gene. For those genes to which more than one Affy tag correspond, the data for the first one listed under ‘Affy Mouse 430 2.0’ is given. Dark green = downregulated under high stringency (as described in METHODS); light green = downregulated under low stringency; red = upregulated under high stringency; orange = upregulated under low stringency. A dash indicates that the gene was not significantly a [file pone.0000643.s005.zip › Corbo_TableS1/images/4933439C20Rik_TSS.gif]

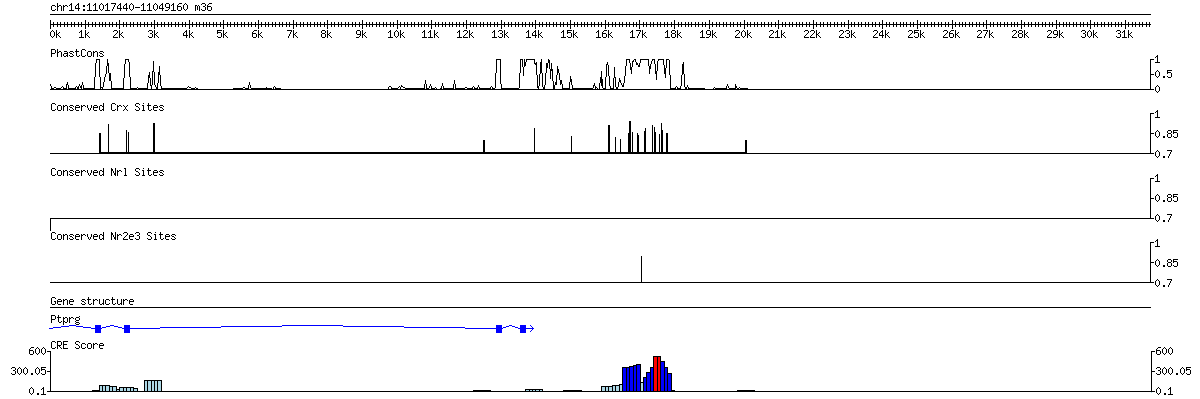

Supplement: Table S1 — Genes in the mouse photoreceptor transcription network. This table is a database which includes all genes dysregulated in Crx-/-, Nrl-/-, and/or Nr2e3-/- under high stringency criteria. Each row in the database represents a gene in the network and includes multiple links to additional types of information about that gene. ‘Large image gene +/− 15 Kb’ links to an image of our computational prediction of photoreceptor CREs in the genomic region 15 Kb upstream and downstream of the gene in question. ‘Closeup image TSS +/− 15 Kb’ links to an image of our computational prediction in the region 15 Kb on either side of the gene's TSS. Black bars within this image highlight the following regulatory peaks (if any are present): the peak closest to the TSS and the peak with the highest CRE score within this 30 Kb window. For those genes whose CRE predictions were tested experimentally, a red bar in this image indicates the location of the CRE that was tested. For Crx and Elovl4 the tested CRE is outside of this 30 Kb window and is therefore indicated in the ‘Large image’. ‘Links’ contains links to the UCSC genome browser, ENSEMBL, and NCBI database entries for the indicated gene, if available. ‘Max score (threshold of 200)’ contains the score of the highest predicted regulatory peak within the 30 Kb window around the gene's TSS. If this window does not contain any predicted peaks ≥ 200 (our cutoff threshold) no value is given (indicated by a dash). The next four columns of the table (‘Nr2e3-/-’ etc.) show the wild-type-to-mutant ratios of the averaged microarray scores for the given gene. For those genes to which more than one Affy tag correspond, the data for the first one listed under ‘Affy Mouse 430 2.0’ is given. Dark green = downregulated under high stringency (as described in METHODS); light green = downregulated under low stringency; red = upregulated under high stringency; orange = upregulated under low stringency. A dash indicates that the gene was not significantly a [file pone.0000643.s005.zip › Corbo_TableS1/images/5430405N12Rik.gif]

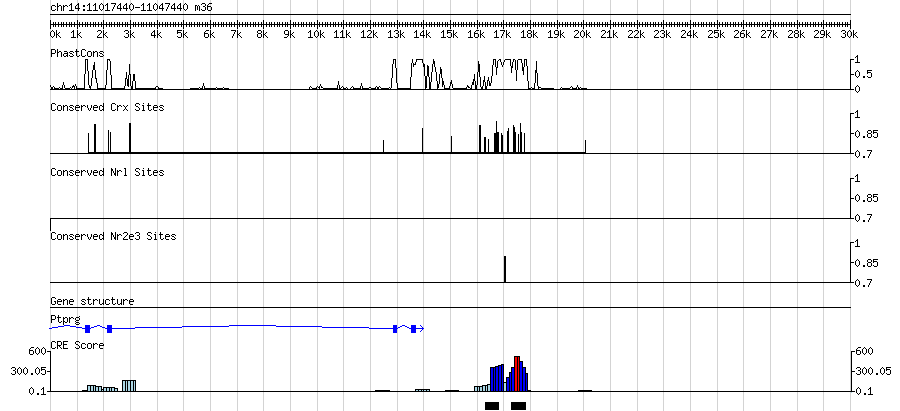

Supplement: Table S1 — Genes in the mouse photoreceptor transcription network. This table is a database which includes all genes dysregulated in Crx-/-, Nrl-/-, and/or Nr2e3-/- under high stringency criteria. Each row in the database represents a gene in the network and includes multiple links to additional types of information about that gene. ‘Large image gene +/− 15 Kb’ links to an image of our computational prediction of photoreceptor CREs in the genomic region 15 Kb upstream and downstream of the gene in question. ‘Closeup image TSS +/− 15 Kb’ links to an image of our computational prediction in the region 15 Kb on either side of the gene's TSS. Black bars within this image highlight the following regulatory peaks (if any are present): the peak closest to the TSS and the peak with the highest CRE score within this 30 Kb window. For those genes whose CRE predictions were tested experimentally, a red bar in this image indicates the location of the CRE that was tested. For Crx and Elovl4 the tested CRE is outside of this 30 Kb window and is therefore indicated in the ‘Large image’. ‘Links’ contains links to the UCSC genome browser, ENSEMBL, and NCBI database entries for the indicated gene, if available. ‘Max score (threshold of 200)’ contains the score of the highest predicted regulatory peak within the 30 Kb window around the gene's TSS. If this window does not contain any predicted peaks ≥ 200 (our cutoff threshold) no value is given (indicated by a dash). The next four columns of the table (‘Nr2e3-/-’ etc.) show the wild-type-to-mutant ratios of the averaged microarray scores for the given gene. For those genes to which more than one Affy tag correspond, the data for the first one listed under ‘Affy Mouse 430 2.0’ is given. Dark green = downregulated under high stringency (as described in METHODS); light green = downregulated under low stringency; red = upregulated under high stringency; orange = upregulated under low stringency. A dash indicates that the gene was not significantly a [file pone.0000643.s005.zip › Corbo_TableS1/images/5430405N12Rik_TSS.gif]

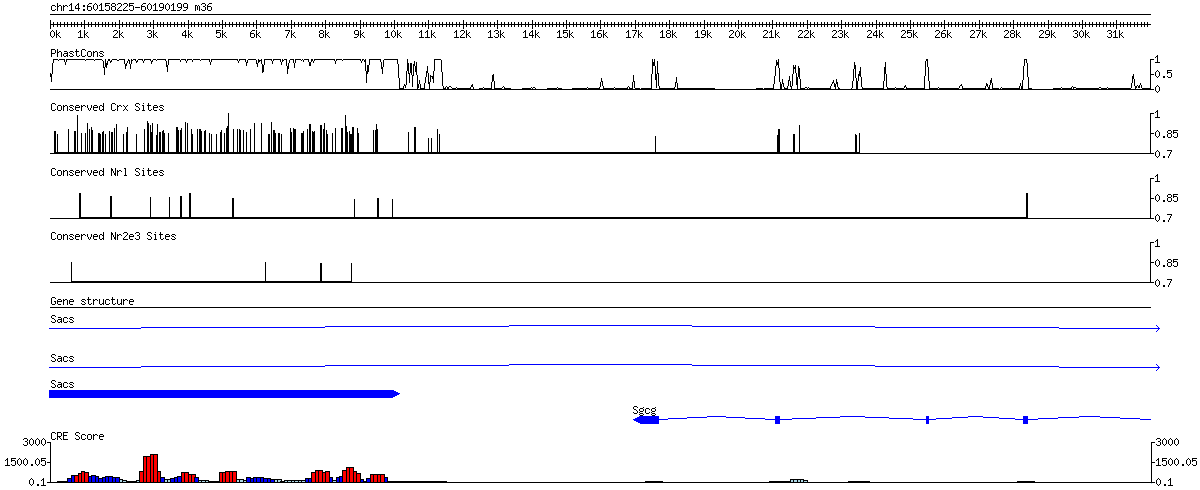

Supplement: Table S1 — Genes in the mouse photoreceptor transcription network. This table is a database which includes all genes dysregulated in Crx-/-, Nrl-/-, and/or Nr2e3-/- under high stringency criteria. Each row in the database represents a gene in the network and includes multiple links to additional types of information about that gene. ‘Large image gene +/− 15 Kb’ links to an image of our computational prediction of photoreceptor CREs in the genomic region 15 Kb upstream and downstream of the gene in question. ‘Closeup image TSS +/− 15 Kb’ links to an image of our computational prediction in the region 15 Kb on either side of the gene's TSS. Black bars within this image highlight the following regulatory peaks (if any are present): the peak closest to the TSS and the peak with the highest CRE score within this 30 Kb window. For those genes whose CRE predictions were tested experimentally, a red bar in this image indicates the location of the CRE that was tested. For Crx and Elovl4 the tested CRE is outside of this 30 Kb window and is therefore indicated in the ‘Large image’. ‘Links’ contains links to the UCSC genome browser, ENSEMBL, and NCBI database entries for the indicated gene, if available. ‘Max score (threshold of 200)’ contains the score of the highest predicted regulatory peak within the 30 Kb window around the gene's TSS. If this window does not contain any predicted peaks ≥ 200 (our cutoff threshold) no value is given (indicated by a dash). The next four columns of the table (‘Nr2e3-/-’ etc.) show the wild-type-to-mutant ratios of the averaged microarray scores for the given gene. For those genes to which more than one Affy tag correspond, the data for the first one listed under ‘Affy Mouse 430 2.0’ is given. Dark green = downregulated under high stringency (as described in METHODS); light green = downregulated under low stringency; red = upregulated under high stringency; orange = upregulated under low stringency. A dash indicates that the gene was not significantly a [file pone.0000643.s005.zip › Corbo_TableS1/images/5430420E18Rik.gif]

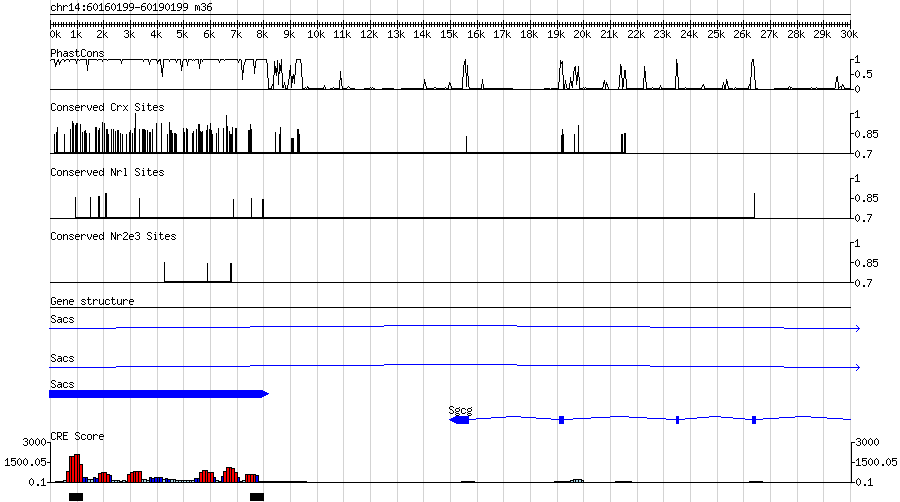

Supplement: Table S1 — Genes in the mouse photoreceptor transcription network. This table is a database which includes all genes dysregulated in Crx-/-, Nrl-/-, and/or Nr2e3-/- under high stringency criteria. Each row in the database represents a gene in the network and includes multiple links to additional types of information about that gene. ‘Large image gene +/− 15 Kb’ links to an image of our computational prediction of photoreceptor CREs in the genomic region 15 Kb upstream and downstream of the gene in question. ‘Closeup image TSS +/− 15 Kb’ links to an image of our computational prediction in the region 15 Kb on either side of the gene's TSS. Black bars within this image highlight the following regulatory peaks (if any are present): the peak closest to the TSS and the peak with the highest CRE score within this 30 Kb window. For those genes whose CRE predictions were tested experimentally, a red bar in this image indicates the location of the CRE that was tested. For Crx and Elovl4 the tested CRE is outside of this 30 Kb window and is therefore indicated in the ‘Large image’. ‘Links’ contains links to the UCSC genome browser, ENSEMBL, and NCBI database entries for the indicated gene, if available. ‘Max score (threshold of 200)’ contains the score of the highest predicted regulatory peak within the 30 Kb window around the gene's TSS. If this window does not contain any predicted peaks ≥ 200 (our cutoff threshold) no value is given (indicated by a dash). The next four columns of the table (‘Nr2e3-/-’ etc.) show the wild-type-to-mutant ratios of the averaged microarray scores for the given gene. For those genes to which more than one Affy tag correspond, the data for the first one listed under ‘Affy Mouse 430 2.0’ is given. Dark green = downregulated under high stringency (as described in METHODS); light green = downregulated under low stringency; red = upregulated under high stringency; orange = upregulated under low stringency. A dash indicates that the gene was not significantly a [file pone.0000643.s005.zip › Corbo_TableS1/images/5430420E18Rik_TSS.gif]

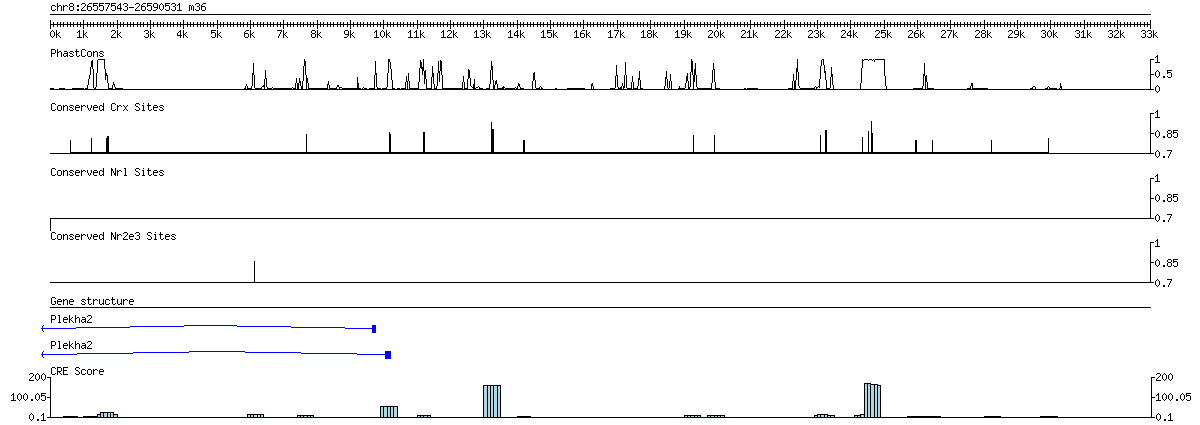

Supplement: Table S1 — Genes in the mouse photoreceptor transcription network. This table is a database which includes all genes dysregulated in Crx-/-, Nrl-/-, and/or Nr2e3-/- under high stringency criteria. Each row in the database represents a gene in the network and includes multiple links to additional types of information about that gene. ‘Large image gene +/− 15 Kb’ links to an image of our computational prediction of photoreceptor CREs in the genomic region 15 Kb upstream and downstream of the gene in question. ‘Closeup image TSS +/− 15 Kb’ links to an image of our computational prediction in the region 15 Kb on either side of the gene's TSS. Black bars within this image highlight the following regulatory peaks (if any are present): the peak closest to the TSS and the peak with the highest CRE score within this 30 Kb window. For those genes whose CRE predictions were tested experimentally, a red bar in this image indicates the location of the CRE that was tested. For Crx and Elovl4 the tested CRE is outside of this 30 Kb window and is therefore indicated in the ‘Large image’. ‘Links’ contains links to the UCSC genome browser, ENSEMBL, and NCBI database entries for the indicated gene, if available. ‘Max score (threshold of 200)’ contains the score of the highest predicted regulatory peak within the 30 Kb window around the gene's TSS. If this window does not contain any predicted peaks ≥ 200 (our cutoff threshold) no value is given (indicated by a dash). The next four columns of the table (‘Nr2e3-/-’ etc.) show the wild-type-to-mutant ratios of the averaged microarray scores for the given gene. For those genes to which more than one Affy tag correspond, the data for the first one listed under ‘Affy Mouse 430 2.0’ is given. Dark green = downregulated under high stringency (as described in METHODS); light green = downregulated under low stringency; red = upregulated under high stringency; orange = upregulated under low stringency. A dash indicates that the gene was not significantly a [file pone.0000643.s005.zip › Corbo_TableS1/images/5830408C22Rik.gif]

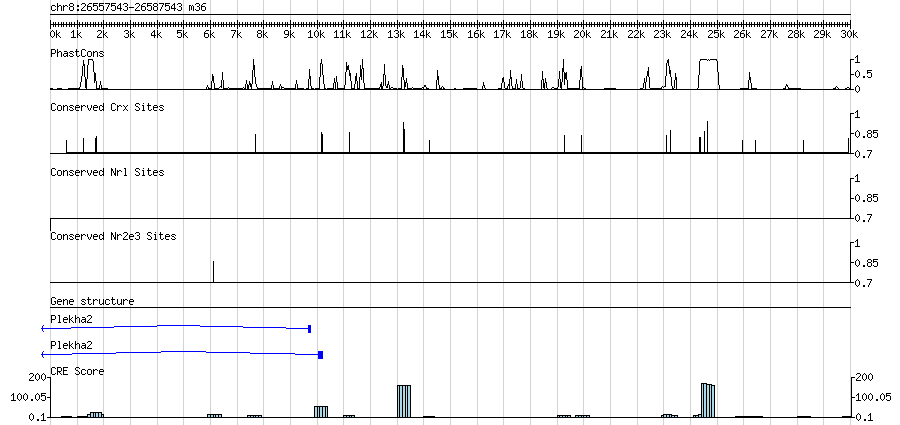

Supplement: Table S1 — Genes in the mouse photoreceptor transcription network. This table is a database which includes all genes dysregulated in Crx-/-, Nrl-/-, and/or Nr2e3-/- under high stringency criteria. Each row in the database represents a gene in the network and includes multiple links to additional types of information about that gene. ‘Large image gene +/− 15 Kb’ links to an image of our computational prediction of photoreceptor CREs in the genomic region 15 Kb upstream and downstream of the gene in question. ‘Closeup image TSS +/− 15 Kb’ links to an image of our computational prediction in the region 15 Kb on either side of the gene's TSS. Black bars within this image highlight the following regulatory peaks (if any are present): the peak closest to the TSS and the peak with the highest CRE score within this 30 Kb window. For those genes whose CRE predictions were tested experimentally, a red bar in this image indicates the location of the CRE that was tested. For Crx and Elovl4 the tested CRE is outside of this 30 Kb window and is therefore indicated in the ‘Large image’. ‘Links’ contains links to the UCSC genome browser, ENSEMBL, and NCBI database entries for the indicated gene, if available. ‘Max score (threshold of 200)’ contains the score of the highest predicted regulatory peak within the 30 Kb window around the gene's TSS. If this window does not contain any predicted peaks ≥ 200 (our cutoff threshold) no value is given (indicated by a dash). The next four columns of the table (‘Nr2e3-/-’ etc.) show the wild-type-to-mutant ratios of the averaged microarray scores for the given gene. For those genes to which more than one Affy tag correspond, the data for the first one listed under ‘Affy Mouse 430 2.0’ is given. Dark green = downregulated under high stringency (as described in METHODS); light green = downregulated under low stringency; red = upregulated under high stringency; orange = upregulated under low stringency. A dash indicates that the gene was not significantly a [file pone.0000643.s005.zip › Corbo_TableS1/images/5830408C22Rik_TSS.gif]

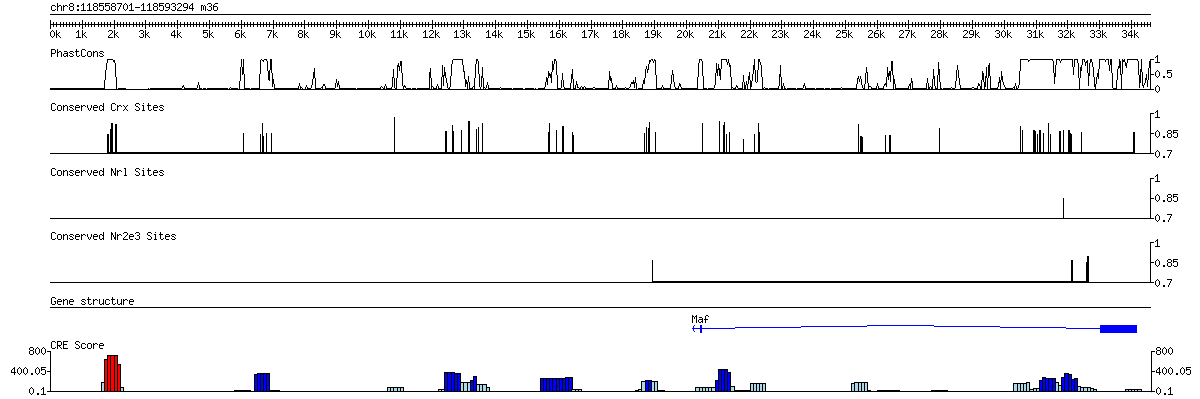

Supplement: Table S1 — Genes in the mouse photoreceptor transcription network. This table is a database which includes all genes dysregulated in Crx-/-, Nrl-/-, and/or Nr2e3-/- under high stringency criteria. Each row in the database represents a gene in the network and includes multiple links to additional types of information about that gene. ‘Large image gene +/− 15 Kb’ links to an image of our computational prediction of photoreceptor CREs in the genomic region 15 Kb upstream and downstream of the gene in question. ‘Closeup image TSS +/− 15 Kb’ links to an image of our computational prediction in the region 15 Kb on either side of the gene's TSS. Black bars within this image highlight the following regulatory peaks (if any are present): the peak closest to the TSS and the peak with the highest CRE score within this 30 Kb window. For those genes whose CRE predictions were tested experimentally, a red bar in this image indicates the location of the CRE that was tested. For Crx and Elovl4 the tested CRE is outside of this 30 Kb window and is therefore indicated in the ‘Large image’. ‘Links’ contains links to the UCSC genome browser, ENSEMBL, and NCBI database entries for the indicated gene, if available. ‘Max score (threshold of 200)’ contains the score of the highest predicted regulatory peak within the 30 Kb window around the gene's TSS. If this window does not contain any predicted peaks ≥ 200 (our cutoff threshold) no value is given (indicated by a dash). The next four columns of the table (‘Nr2e3-/-’ etc.) show the wild-type-to-mutant ratios of the averaged microarray scores for the given gene. For those genes to which more than one Affy tag correspond, the data for the first one listed under ‘Affy Mouse 430 2.0’ is given. Dark green = downregulated under high stringency (as described in METHODS); light green = downregulated under low stringency; red = upregulated under high stringency; orange = upregulated under low stringency. A dash indicates that the gene was not significantly a [file pone.0000643.s005.zip › Corbo_TableS1/images/6030439D06Rik.gif]

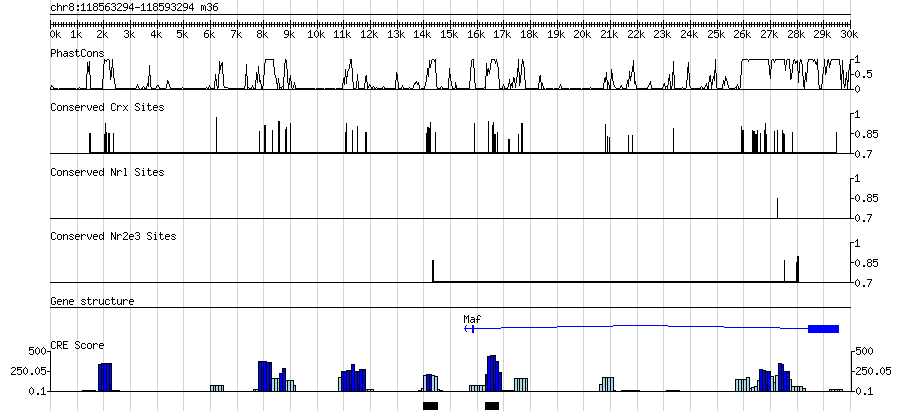

Supplement: Table S1 — Genes in the mouse photoreceptor transcription network. This table is a database which includes all genes dysregulated in Crx-/-, Nrl-/-, and/or Nr2e3-/- under high stringency criteria. Each row in the database represents a gene in the network and includes multiple links to additional types of information about that gene. ‘Large image gene +/− 15 Kb’ links to an image of our computational prediction of photoreceptor CREs in the genomic region 15 Kb upstream and downstream of the gene in question. ‘Closeup image TSS +/− 15 Kb’ links to an image of our computational prediction in the region 15 Kb on either side of the gene's TSS. Black bars within this image highlight the following regulatory peaks (if any are present): the peak closest to the TSS and the peak with the highest CRE score within this 30 Kb window. For those genes whose CRE predictions were tested experimentally, a red bar in this image indicates the location of the CRE that was tested. For Crx and Elovl4 the tested CRE is outside of this 30 Kb window and is therefore indicated in the ‘Large image’. ‘Links’ contains links to the UCSC genome browser, ENSEMBL, and NCBI database entries for the indicated gene, if available. ‘Max score (threshold of 200)’ contains the score of the highest predicted regulatory peak within the 30 Kb window around the gene's TSS. If this window does not contain any predicted peaks ≥ 200 (our cutoff threshold) no value is given (indicated by a dash). The next four columns of the table (‘Nr2e3-/-’ etc.) show the wild-type-to-mutant ratios of the averaged microarray scores for the given gene. For those genes to which more than one Affy tag correspond, the data for the first one listed under ‘Affy Mouse 430 2.0’ is given. Dark green = downregulated under high stringency (as described in METHODS); light green = downregulated under low stringency; red = upregulated under high stringency; orange = upregulated under low stringency. A dash indicates that the gene was not significantly a [file pone.0000643.s005.zip › Corbo_TableS1/images/6030439D06Rik_TSS.gif]

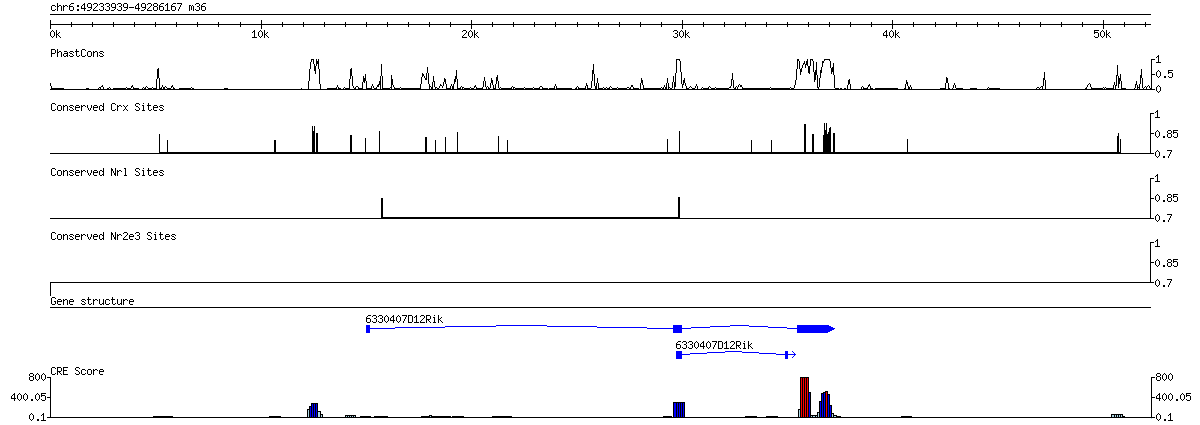

Supplement: Table S1 — Genes in the mouse photoreceptor transcription network. This table is a database which includes all genes dysregulated in Crx-/-, Nrl-/-, and/or Nr2e3-/- under high stringency criteria. Each row in the database represents a gene in the network and includes multiple links to additional types of information about that gene. ‘Large image gene +/− 15 Kb’ links to an image of our computational prediction of photoreceptor CREs in the genomic region 15 Kb upstream and downstream of the gene in question. ‘Closeup image TSS +/− 15 Kb’ links to an image of our computational prediction in the region 15 Kb on either side of the gene's TSS. Black bars within this image highlight the following regulatory peaks (if any are present): the peak closest to the TSS and the peak with the highest CRE score within this 30 Kb window. For those genes whose CRE predictions were tested experimentally, a red bar in this image indicates the location of the CRE that was tested. For Crx and Elovl4 the tested CRE is outside of this 30 Kb window and is therefore indicated in the ‘Large image’. ‘Links’ contains links to the UCSC genome browser, ENSEMBL, and NCBI database entries for the indicated gene, if available. ‘Max score (threshold of 200)’ contains the score of the highest predicted regulatory peak within the 30 Kb window around the gene's TSS. If this window does not contain any predicted peaks ≥ 200 (our cutoff threshold) no value is given (indicated by a dash). The next four columns of the table (‘Nr2e3-/-’ etc.) show the wild-type-to-mutant ratios of the averaged microarray scores for the given gene. For those genes to which more than one Affy tag correspond, the data for the first one listed under ‘Affy Mouse 430 2.0’ is given. Dark green = downregulated under high stringency (as described in METHODS); light green = downregulated under low stringency; red = upregulated under high stringency; orange = upregulated under low stringency. A dash indicates that the gene was not significantly a [file pone.0000643.s005.zip › Corbo_TableS1/images/6330407D12Rik.gif]

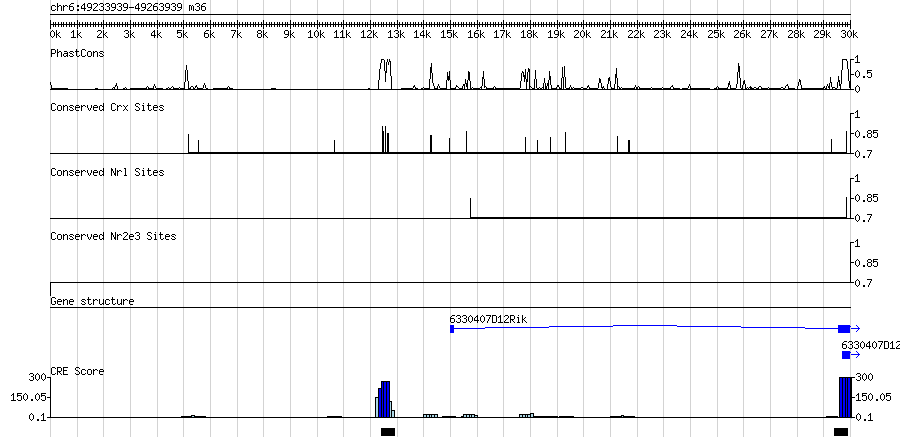

Supplement: Table S1 — Genes in the mouse photoreceptor transcription network. This table is a database which includes all genes dysregulated in Crx-/-, Nrl-/-, and/or Nr2e3-/- under high stringency criteria. Each row in the database represents a gene in the network and includes multiple links to additional types of information about that gene. ‘Large image gene +/− 15 Kb’ links to an image of our computational prediction of photoreceptor CREs in the genomic region 15 Kb upstream and downstream of the gene in question. ‘Closeup image TSS +/− 15 Kb’ links to an image of our computational prediction in the region 15 Kb on either side of the gene's TSS. Black bars within this image highlight the following regulatory peaks (if any are present): the peak closest to the TSS and the peak with the highest CRE score within this 30 Kb window. For those genes whose CRE predictions were tested experimentally, a red bar in this image indicates the location of the CRE that was tested. For Crx and Elovl4 the tested CRE is outside of this 30 Kb window and is therefore indicated in the ‘Large image’. ‘Links’ contains links to the UCSC genome browser, ENSEMBL, and NCBI database entries for the indicated gene, if available. ‘Max score (threshold of 200)’ contains the score of the highest predicted regulatory peak within the 30 Kb window around the gene's TSS. If this window does not contain any predicted peaks ≥ 200 (our cutoff threshold) no value is given (indicated by a dash). The next four columns of the table (‘Nr2e3-/-’ etc.) show the wild-type-to-mutant ratios of the averaged microarray scores for the given gene. For those genes to which more than one Affy tag correspond, the data for the first one listed under ‘Affy Mouse 430 2.0’ is given. Dark green = downregulated under high stringency (as described in METHODS); light green = downregulated under low stringency; red = upregulated under high stringency; orange = upregulated under low stringency. A dash indicates that the gene was not significantly a [file pone.0000643.s005.zip › Corbo_TableS1/images/6330407D12Rik_TSS.gif]

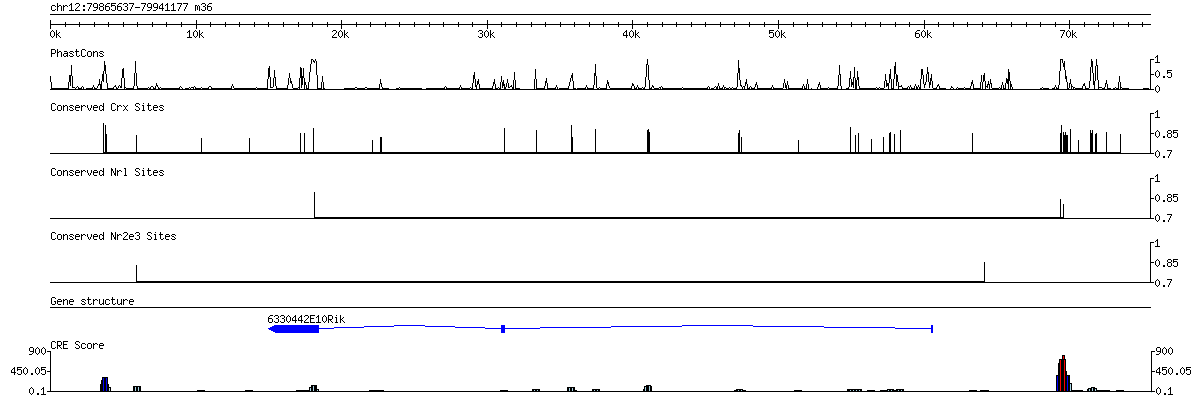

Supplement: Table S1 — Genes in the mouse photoreceptor transcription network. This table is a database which includes all genes dysregulated in Crx-/-, Nrl-/-, and/or Nr2e3-/- under high stringency criteria. Each row in the database represents a gene in the network and includes multiple links to additional types of information about that gene. ‘Large image gene +/− 15 Kb’ links to an image of our computational prediction of photoreceptor CREs in the genomic region 15 Kb upstream and downstream of the gene in question. ‘Closeup image TSS +/− 15 Kb’ links to an image of our computational prediction in the region 15 Kb on either side of the gene's TSS. Black bars within this image highlight the following regulatory peaks (if any are present): the peak closest to the TSS and the peak with the highest CRE score within this 30 Kb window. For those genes whose CRE predictions were tested experimentally, a red bar in this image indicates the location of the CRE that was tested. For Crx and Elovl4 the tested CRE is outside of this 30 Kb window and is therefore indicated in the ‘Large image’. ‘Links’ contains links to the UCSC genome browser, ENSEMBL, and NCBI database entries for the indicated gene, if available. ‘Max score (threshold of 200)’ contains the score of the highest predicted regulatory peak within the 30 Kb window around the gene's TSS. If this window does not contain any predicted peaks ≥ 200 (our cutoff threshold) no value is given (indicated by a dash). The next four columns of the table (‘Nr2e3-/-’ etc.) show the wild-type-to-mutant ratios of the averaged microarray scores for the given gene. For those genes to which more than one Affy tag correspond, the data for the first one listed under ‘Affy Mouse 430 2.0’ is given. Dark green = downregulated under high stringency (as described in METHODS); light green = downregulated under low stringency; red = upregulated under high stringency; orange = upregulated under low stringency. A dash indicates that the gene was not significantly a [file pone.0000643.s005.zip › Corbo_TableS1/images/6330442E10Rik.gif]

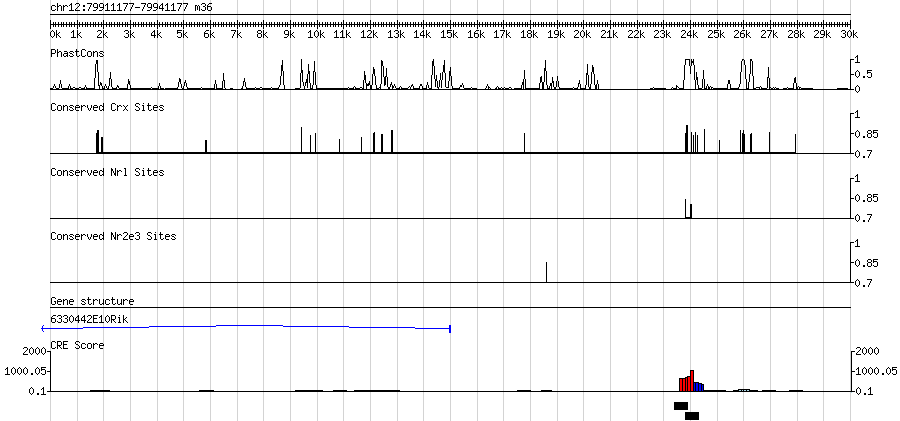

Supplement: Table S1 — Genes in the mouse photoreceptor transcription network. This table is a database which includes all genes dysregulated in Crx-/-, Nrl-/-, and/or Nr2e3-/- under high stringency criteria. Each row in the database represents a gene in the network and includes multiple links to additional types of information about that gene. ‘Large image gene +/− 15 Kb’ links to an image of our computational prediction of photoreceptor CREs in the genomic region 15 Kb upstream and downstream of the gene in question. ‘Closeup image TSS +/− 15 Kb’ links to an image of our computational prediction in the region 15 Kb on either side of the gene's TSS. Black bars within this image highlight the following regulatory peaks (if any are present): the peak closest to the TSS and the peak with the highest CRE score within this 30 Kb window. For those genes whose CRE predictions were tested experimentally, a red bar in this image indicates the location of the CRE that was tested. For Crx and Elovl4 the tested CRE is outside of this 30 Kb window and is therefore indicated in the ‘Large image’. ‘Links’ contains links to the UCSC genome browser, ENSEMBL, and NCBI database entries for the indicated gene, if available. ‘Max score (threshold of 200)’ contains the score of the highest predicted regulatory peak within the 30 Kb window around the gene's TSS. If this window does not contain any predicted peaks ≥ 200 (our cutoff threshold) no value is given (indicated by a dash). The next four columns of the table (‘Nr2e3-/-’ etc.) show the wild-type-to-mutant ratios of the averaged microarray scores for the given gene. For those genes to which more than one Affy tag correspond, the data for the first one listed under ‘Affy Mouse 430 2.0’ is given. Dark green = downregulated under high stringency (as described in METHODS); light green = downregulated under low stringency; red = upregulated under high stringency; orange = upregulated under low stringency. A dash indicates that the gene was not significantly a [file pone.0000643.s005.zip › Corbo_TableS1/images/6330442E10Rik_TSS.gif]

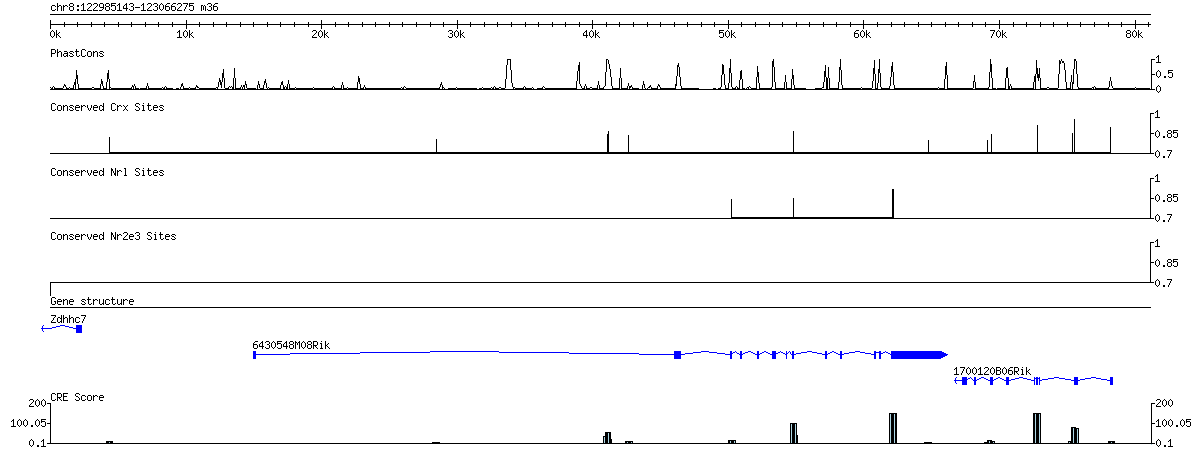

Supplement: Table S1 — Genes in the mouse photoreceptor transcription network. This table is a database which includes all genes dysregulated in Crx-/-, Nrl-/-, and/or Nr2e3-/- under high stringency criteria. Each row in the database represents a gene in the network and includes multiple links to additional types of information about that gene. ‘Large image gene +/− 15 Kb’ links to an image of our computational prediction of photoreceptor CREs in the genomic region 15 Kb upstream and downstream of the gene in question. ‘Closeup image TSS +/− 15 Kb’ links to an image of our computational prediction in the region 15 Kb on either side of the gene's TSS. Black bars within this image highlight the following regulatory peaks (if any are present): the peak closest to the TSS and the peak with the highest CRE score within this 30 Kb window. For those genes whose CRE predictions were tested experimentally, a red bar in this image indicates the location of the CRE that was tested. For Crx and Elovl4 the tested CRE is outside of this 30 Kb window and is therefore indicated in the ‘Large image’. ‘Links’ contains links to the UCSC genome browser, ENSEMBL, and NCBI database entries for the indicated gene, if available. ‘Max score (threshold of 200)’ contains the score of the highest predicted regulatory peak within the 30 Kb window around the gene's TSS. If this window does not contain any predicted peaks ≥ 200 (our cutoff threshold) no value is given (indicated by a dash). The next four columns of the table (‘Nr2e3-/-’ etc.) show the wild-type-to-mutant ratios of the averaged microarray scores for the given gene. For those genes to which more than one Affy tag correspond, the data for the first one listed under ‘Affy Mouse 430 2.0’ is given. Dark green = downregulated under high stringency (as described in METHODS); light green = downregulated under low stringency; red = upregulated under high stringency; orange = upregulated under low stringency. A dash indicates that the gene was not significantly a [file pone.0000643.s005.zip › Corbo_TableS1/images/6430548M08Rik.gif]

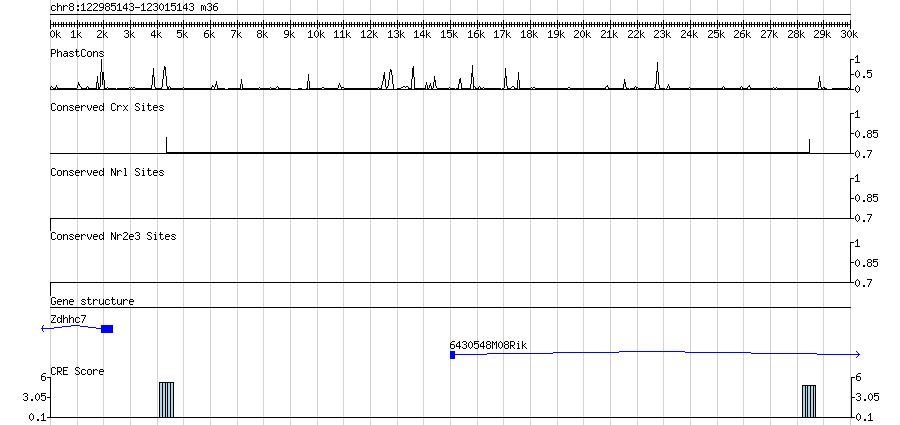

Supplement: Table S1 — Genes in the mouse photoreceptor transcription network. This table is a database which includes all genes dysregulated in Crx-/-, Nrl-/-, and/or Nr2e3-/- under high stringency criteria. Each row in the database represents a gene in the network and includes multiple links to additional types of information about that gene. ‘Large image gene +/− 15 Kb’ links to an image of our computational prediction of photoreceptor CREs in the genomic region 15 Kb upstream and downstream of the gene in question. ‘Closeup image TSS +/− 15 Kb’ links to an image of our computational prediction in the region 15 Kb on either side of the gene's TSS. Black bars within this image highlight the following regulatory peaks (if any are present): the peak closest to the TSS and the peak with the highest CRE score within this 30 Kb window. For those genes whose CRE predictions were tested experimentally, a red bar in this image indicates the location of the CRE that was tested. For Crx and Elovl4 the tested CRE is outside of this 30 Kb window and is therefore indicated in the ‘Large image’. ‘Links’ contains links to the UCSC genome browser, ENSEMBL, and NCBI database entries for the indicated gene, if available. ‘Max score (threshold of 200)’ contains the score of the highest predicted regulatory peak within the 30 Kb window around the gene's TSS. If this window does not contain any predicted peaks ≥ 200 (our cutoff threshold) no value is given (indicated by a dash). The next four columns of the table (‘Nr2e3-/-’ etc.) show the wild-type-to-mutant ratios of the averaged microarray scores for the given gene. For those genes to which more than one Affy tag correspond, the data for the first one listed under ‘Affy Mouse 430 2.0’ is given. Dark green = downregulated under high stringency (as described in METHODS); light green = downregulated under low stringency; red = upregulated under high stringency; orange = upregulated under low stringency. A dash indicates that the gene was not significantly a [file pone.0000643.s005.zip › Corbo_TableS1/images/6430548M08Rik_TSS.gif]

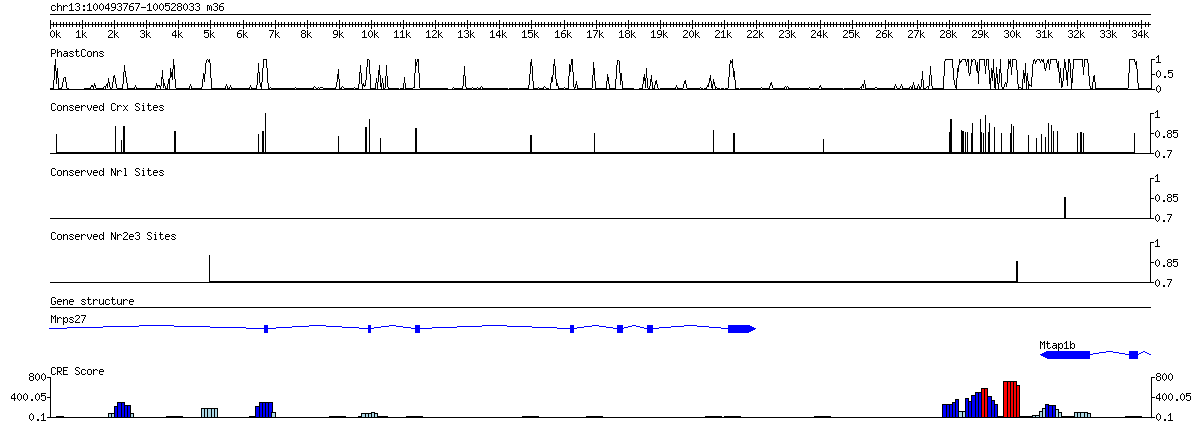

Supplement: Table S1 — Genes in the mouse photoreceptor transcription network. This table is a database which includes all genes dysregulated in Crx-/-, Nrl-/-, and/or Nr2e3-/- under high stringency criteria. Each row in the database represents a gene in the network and includes multiple links to additional types of information about that gene. ‘Large image gene +/− 15 Kb’ links to an image of our computational prediction of photoreceptor CREs in the genomic region 15 Kb upstream and downstream of the gene in question. ‘Closeup image TSS +/− 15 Kb’ links to an image of our computational prediction in the region 15 Kb on either side of the gene's TSS. Black bars within this image highlight the following regulatory peaks (if any are present): the peak closest to the TSS and the peak with the highest CRE score within this 30 Kb window. For those genes whose CRE predictions were tested experimentally, a red bar in this image indicates the location of the CRE that was tested. For Crx and Elovl4 the tested CRE is outside of this 30 Kb window and is therefore indicated in the ‘Large image’. ‘Links’ contains links to the UCSC genome browser, ENSEMBL, and NCBI database entries for the indicated gene, if available. ‘Max score (threshold of 200)’ contains the score of the highest predicted regulatory peak within the 30 Kb window around the gene's TSS. If this window does not contain any predicted peaks ≥ 200 (our cutoff threshold) no value is given (indicated by a dash). The next four columns of the table (‘Nr2e3-/-’ etc.) show the wild-type-to-mutant ratios of the averaged microarray scores for the given gene. For those genes to which more than one Affy tag correspond, the data for the first one listed under ‘Affy Mouse 430 2.0’ is given. Dark green = downregulated under high stringency (as described in METHODS); light green = downregulated under low stringency; red = upregulated under high stringency; orange = upregulated under low stringency. A dash indicates that the gene was not significantly a [file pone.0000643.s005.zip › Corbo_TableS1/images/6430562O15Rik.gif]

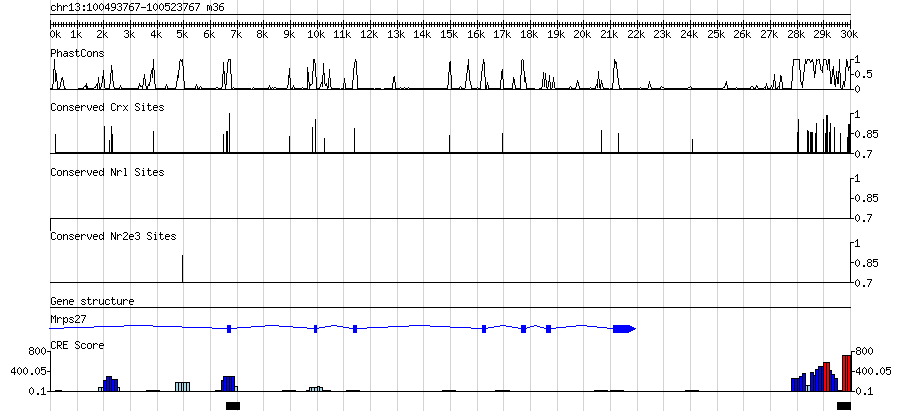

Supplement: Table S1 — Genes in the mouse photoreceptor transcription network. This table is a database which includes all genes dysregulated in Crx-/-, Nrl-/-, and/or Nr2e3-/- under high stringency criteria. Each row in the database represents a gene in the network and includes multiple links to additional types of information about that gene. ‘Large image gene +/− 15 Kb’ links to an image of our computational prediction of photoreceptor CREs in the genomic region 15 Kb upstream and downstream of the gene in question. ‘Closeup image TSS +/− 15 Kb’ links to an image of our computational prediction in the region 15 Kb on either side of the gene's TSS. Black bars within this image highlight the following regulatory peaks (if any are present): the peak closest to the TSS and the peak with the highest CRE score within this 30 Kb window. For those genes whose CRE predictions were tested experimentally, a red bar in this image indicates the location of the CRE that was tested. For Crx and Elovl4 the tested CRE is outside of this 30 Kb window and is therefore indicated in the ‘Large image’. ‘Links’ contains links to the UCSC genome browser, ENSEMBL, and NCBI database entries for the indicated gene, if available. ‘Max score (threshold of 200)’ contains the score of the highest predicted regulatory peak within the 30 Kb window around the gene's TSS. If this window does not contain any predicted peaks ≥ 200 (our cutoff threshold) no value is given (indicated by a dash). The next four columns of the table (‘Nr2e3-/-’ etc.) show the wild-type-to-mutant ratios of the averaged microarray scores for the given gene. For those genes to which more than one Affy tag correspond, the data for the first one listed under ‘Affy Mouse 430 2.0’ is given. Dark green = downregulated under high stringency (as described in METHODS); light green = downregulated under low stringency; red = upregulated under high stringency; orange = upregulated under low stringency. A dash indicates that the gene was not significantly a [file pone.0000643.s005.zip › Corbo_TableS1/images/6430562O15Rik_TSS.gif]

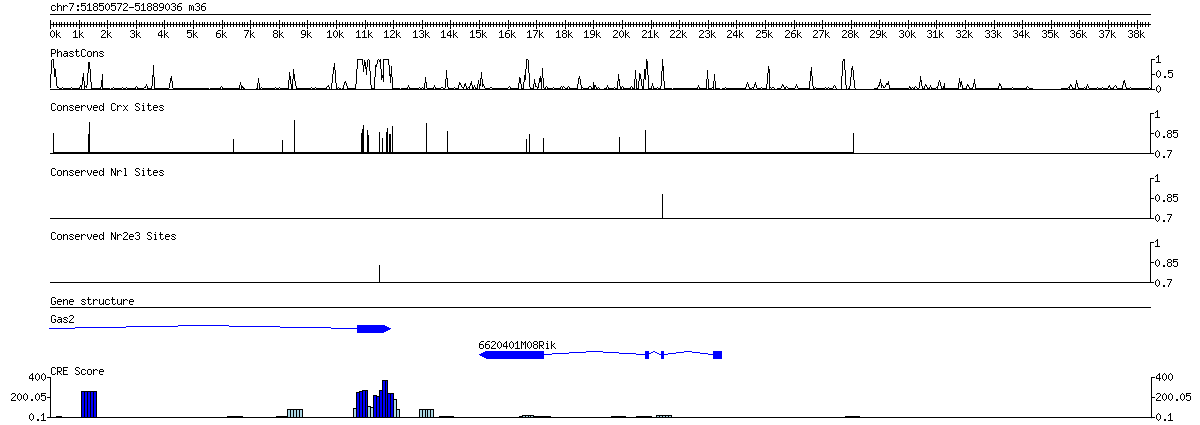

Supplement: Table S1 — Genes in the mouse photoreceptor transcription network. This table is a database which includes all genes dysregulated in Crx-/-, Nrl-/-, and/or Nr2e3-/- under high stringency criteria. Each row in the database represents a gene in the network and includes multiple links to additional types of information about that gene. ‘Large image gene +/− 15 Kb’ links to an image of our computational prediction of photoreceptor CREs in the genomic region 15 Kb upstream and downstream of the gene in question. ‘Closeup image TSS +/− 15 Kb’ links to an image of our computational prediction in the region 15 Kb on either side of the gene's TSS. Black bars within this image highlight the following regulatory peaks (if any are present): the peak closest to the TSS and the peak with the highest CRE score within this 30 Kb window. For those genes whose CRE predictions were tested experimentally, a red bar in this image indicates the location of the CRE that was tested. For Crx and Elovl4 the tested CRE is outside of this 30 Kb window and is therefore indicated in the ‘Large image’. ‘Links’ contains links to the UCSC genome browser, ENSEMBL, and NCBI database entries for the indicated gene, if available. ‘Max score (threshold of 200)’ contains the score of the highest predicted regulatory peak within the 30 Kb window around the gene's TSS. If this window does not contain any predicted peaks ≥ 200 (our cutoff threshold) no value is given (indicated by a dash). The next four columns of the table (‘Nr2e3-/-’ etc.) show the wild-type-to-mutant ratios of the averaged microarray scores for the given gene. For those genes to which more than one Affy tag correspond, the data for the first one listed under ‘Affy Mouse 430 2.0’ is given. Dark green = downregulated under high stringency (as described in METHODS); light green = downregulated under low stringency; red = upregulated under high stringency; orange = upregulated under low stringency. A dash indicates that the gene was not significantly a [file pone.0000643.s005.zip › Corbo_TableS1/images/6620401M08Rik.gif]

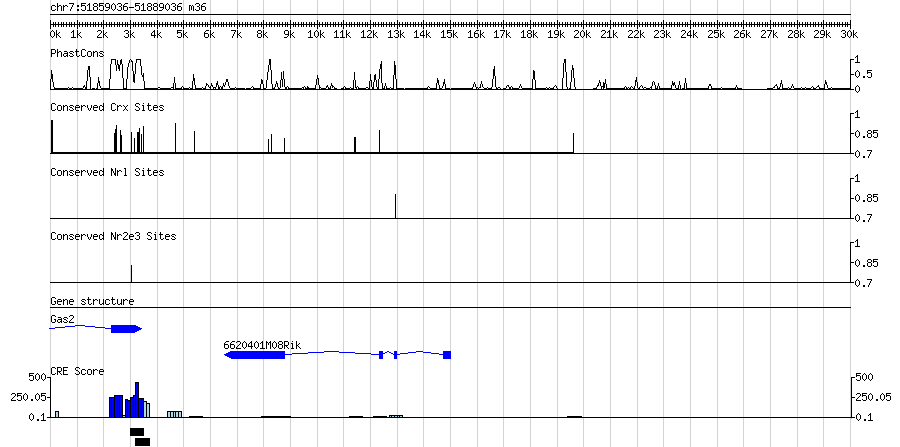

Supplement: Table S1 — Genes in the mouse photoreceptor transcription network. This table is a database which includes all genes dysregulated in Crx-/-, Nrl-/-, and/or Nr2e3-/- under high stringency criteria. Each row in the database represents a gene in the network and includes multiple links to additional types of information about that gene. ‘Large image gene +/− 15 Kb’ links to an image of our computational prediction of photoreceptor CREs in the genomic region 15 Kb upstream and downstream of the gene in question. ‘Closeup image TSS +/− 15 Kb’ links to an image of our computational prediction in the region 15 Kb on either side of the gene's TSS. Black bars within this image highlight the following regulatory peaks (if any are present): the peak closest to the TSS and the peak with the highest CRE score within this 30 Kb window. For those genes whose CRE predictions were tested experimentally, a red bar in this image indicates the location of the CRE that was tested. For Crx and Elovl4 the tested CRE is outside of this 30 Kb window and is therefore indicated in the ‘Large image’. ‘Links’ contains links to the UCSC genome browser, ENSEMBL, and NCBI database entries for the indicated gene, if available. ‘Max score (threshold of 200)’ contains the score of the highest predicted regulatory peak within the 30 Kb window around the gene's TSS. If this window does not contain any predicted peaks ≥ 200 (our cutoff threshold) no value is given (indicated by a dash). The next four columns of the table (‘Nr2e3-/-’ etc.) show the wild-type-to-mutant ratios of the averaged microarray scores for the given gene. For those genes to which more than one Affy tag correspond, the data for the first one listed under ‘Affy Mouse 430 2.0’ is given. Dark green = downregulated under high stringency (as described in METHODS); light green = downregulated under low stringency; red = upregulated under high stringency; orange = upregulated under low stringency. A dash indicates that the gene was not significantly a [file pone.0000643.s005.zip › Corbo_TableS1/images/6620401M08Rik_TSS.gif]

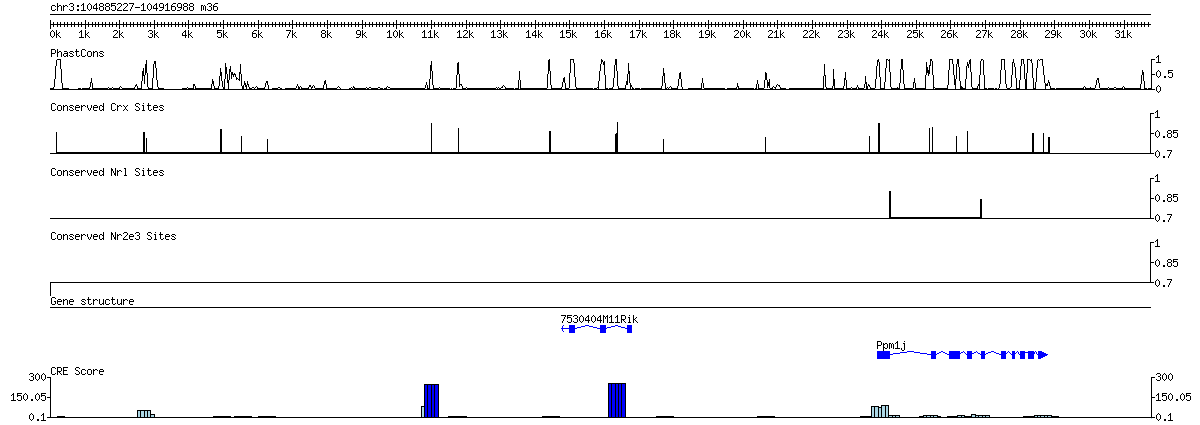

Supplement: Table S1 — Genes in the mouse photoreceptor transcription network. This table is a database which includes all genes dysregulated in Crx-/-, Nrl-/-, and/or Nr2e3-/- under high stringency criteria. Each row in the database represents a gene in the network and includes multiple links to additional types of information about that gene. ‘Large image gene +/− 15 Kb’ links to an image of our computational prediction of photoreceptor CREs in the genomic region 15 Kb upstream and downstream of the gene in question. ‘Closeup image TSS +/− 15 Kb’ links to an image of our computational prediction in the region 15 Kb on either side of the gene's TSS. Black bars within this image highlight the following regulatory peaks (if any are present): the peak closest to the TSS and the peak with the highest CRE score within this 30 Kb window. For those genes whose CRE predictions were tested experimentally, a red bar in this image indicates the location of the CRE that was tested. For Crx and Elovl4 the tested CRE is outside of this 30 Kb window and is therefore indicated in the ‘Large image’. ‘Links’ contains links to the UCSC genome browser, ENSEMBL, and NCBI database entries for the indicated gene, if available. ‘Max score (threshold of 200)’ contains the score of the highest predicted regulatory peak within the 30 Kb window around the gene's TSS. If this window does not contain any predicted peaks ≥ 200 (our cutoff threshold) no value is given (indicated by a dash). The next four columns of the table (‘Nr2e3-/-’ etc.) show the wild-type-to-mutant ratios of the averaged microarray scores for the given gene. For those genes to which more than one Affy tag correspond, the data for the first one listed under ‘Affy Mouse 430 2.0’ is given. Dark green = downregulated under high stringency (as described in METHODS); light green = downregulated under low stringency; red = upregulated under high stringency; orange = upregulated under low stringency. A dash indicates that the gene was not significantly a [file pone.0000643.s005.zip › Corbo_TableS1/images/7530404M11Rik.gif]

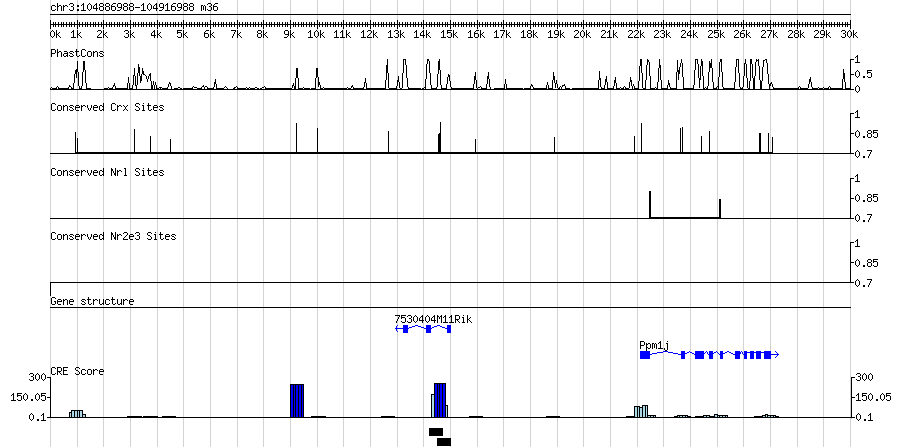

Supplement: Table S1 — Genes in the mouse photoreceptor transcription network. This table is a database which includes all genes dysregulated in Crx-/-, Nrl-/-, and/or Nr2e3-/- under high stringency criteria. Each row in the database represents a gene in the network and includes multiple links to additional types of information about that gene. ‘Large image gene +/− 15 Kb’ links to an image of our computational prediction of photoreceptor CREs in the genomic region 15 Kb upstream and downstream of the gene in question. ‘Closeup image TSS +/− 15 Kb’ links to an image of our computational prediction in the region 15 Kb on either side of the gene's TSS. Black bars within this image highlight the following regulatory peaks (if any are present): the peak closest to the TSS and the peak with the highest CRE score within this 30 Kb window. For those genes whose CRE predictions were tested experimentally, a red bar in this image indicates the location of the CRE that was tested. For Crx and Elovl4 the tested CRE is outside of this 30 Kb window and is therefore indicated in the ‘Large image’. ‘Links’ contains links to the UCSC genome browser, ENSEMBL, and NCBI database entries for the indicated gene, if available. ‘Max score (threshold of 200)’ contains the score of the highest predicted regulatory peak within the 30 Kb window around the gene's TSS. If this window does not contain any predicted peaks ≥ 200 (our cutoff threshold) no value is given (indicated by a dash). The next four columns of the table (‘Nr2e3-/-’ etc.) show the wild-type-to-mutant ratios of the averaged microarray scores for the given gene. For those genes to which more than one Affy tag correspond, the data for the first one listed under ‘Affy Mouse 430 2.0’ is given. Dark green = downregulated under high stringency (as described in METHODS); light green = downregulated under low stringency; red = upregulated under high stringency; orange = upregulated under low stringency. A dash indicates that the gene was not significantly a [file pone.0000643.s005.zip › Corbo_TableS1/images/7530404M11Rik_TSS.gif]

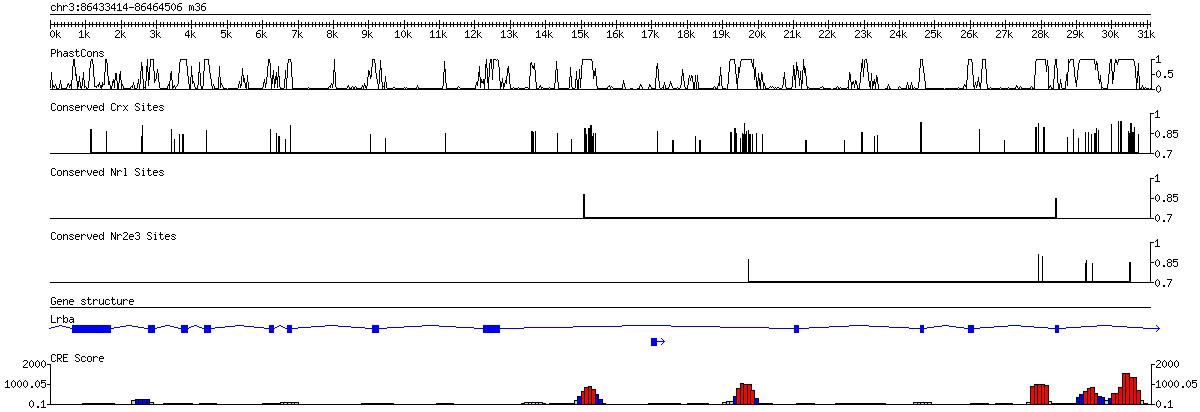

Supplement: Table S1 — Genes in the mouse photoreceptor transcription network. This table is a database which includes all genes dysregulated in Crx-/-, Nrl-/-, and/or Nr2e3-/- under high stringency criteria. Each row in the database represents a gene in the network and includes multiple links to additional types of information about that gene. ‘Large image gene +/− 15 Kb’ links to an image of our computational prediction of photoreceptor CREs in the genomic region 15 Kb upstream and downstream of the gene in question. ‘Closeup image TSS +/− 15 Kb’ links to an image of our computational prediction in the region 15 Kb on either side of the gene's TSS. Black bars within this image highlight the following regulatory peaks (if any are present): the peak closest to the TSS and the peak with the highest CRE score within this 30 Kb window. For those genes whose CRE predictions were tested experimentally, a red bar in this image indicates the location of the CRE that was tested. For Crx and Elovl4 the tested CRE is outside of this 30 Kb window and is therefore indicated in the ‘Large image’. ‘Links’ contains links to the UCSC genome browser, ENSEMBL, and NCBI database entries for the indicated gene, if available. ‘Max score (threshold of 200)’ contains the score of the highest predicted regulatory peak within the 30 Kb window around the gene's TSS. If this window does not contain any predicted peaks ≥ 200 (our cutoff threshold) no value is given (indicated by a dash). The next four columns of the table (‘Nr2e3-/-’ etc.) show the wild-type-to-mutant ratios of the averaged microarray scores for the given gene. For those genes to which more than one Affy tag correspond, the data for the first one listed under ‘Affy Mouse 430 2.0’ is given. Dark green = downregulated under high stringency (as described in METHODS); light green = downregulated under low stringency; red = upregulated under high stringency; orange = upregulated under low stringency. A dash indicates that the gene was not significantly a [file pone.0000643.s005.zip › Corbo_TableS1/images/9030218A15Rik.gif]

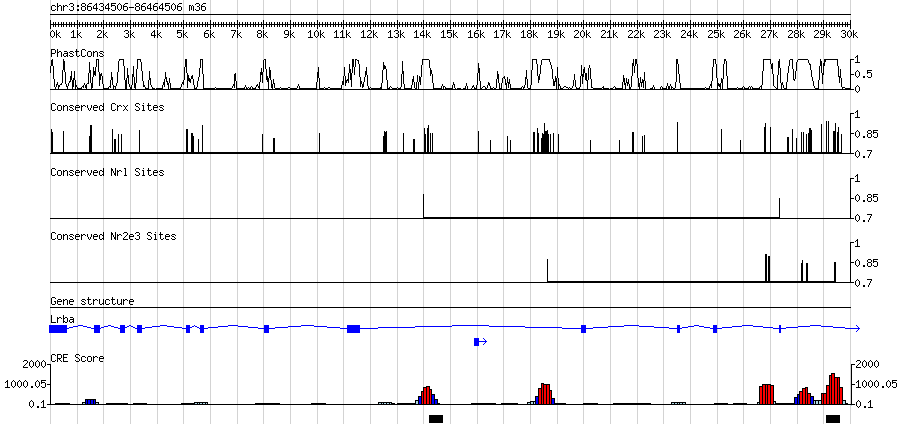

Supplement: Table S1 — Genes in the mouse photoreceptor transcription network. This table is a database which includes all genes dysregulated in Crx-/-, Nrl-/-, and/or Nr2e3-/- under high stringency criteria. Each row in the database represents a gene in the network and includes multiple links to additional types of information about that gene. ‘Large image gene +/− 15 Kb’ links to an image of our computational prediction of photoreceptor CREs in the genomic region 15 Kb upstream and downstream of the gene in question. ‘Closeup image TSS +/− 15 Kb’ links to an image of our computational prediction in the region 15 Kb on either side of the gene's TSS. Black bars within this image highlight the following regulatory peaks (if any are present): the peak closest to the TSS and the peak with the highest CRE score within this 30 Kb window. For those genes whose CRE predictions were tested experimentally, a red bar in this image indicates the location of the CRE that was tested. For Crx and Elovl4 the tested CRE is outside of this 30 Kb window and is therefore indicated in the ‘Large image’. ‘Links’ contains links to the UCSC genome browser, ENSEMBL, and NCBI database entries for the indicated gene, if available. ‘Max score (threshold of 200)’ contains the score of the highest predicted regulatory peak within the 30 Kb window around the gene's TSS. If this window does not contain any predicted peaks ≥ 200 (our cutoff threshold) no value is given (indicated by a dash). The next four columns of the table (‘Nr2e3-/-’ etc.) show the wild-type-to-mutant ratios of the averaged microarray scores for the given gene. For those genes to which more than one Affy tag correspond, the data for the first one listed under ‘Affy Mouse 430 2.0’ is given. Dark green = downregulated under high stringency (as described in METHODS); light green = downregulated under low stringency; red = upregulated under high stringency; orange = upregulated under low stringency. A dash indicates that the gene was not significantly a [file pone.0000643.s005.zip › Corbo_TableS1/images/9030218A15Rik_TSS.gif]

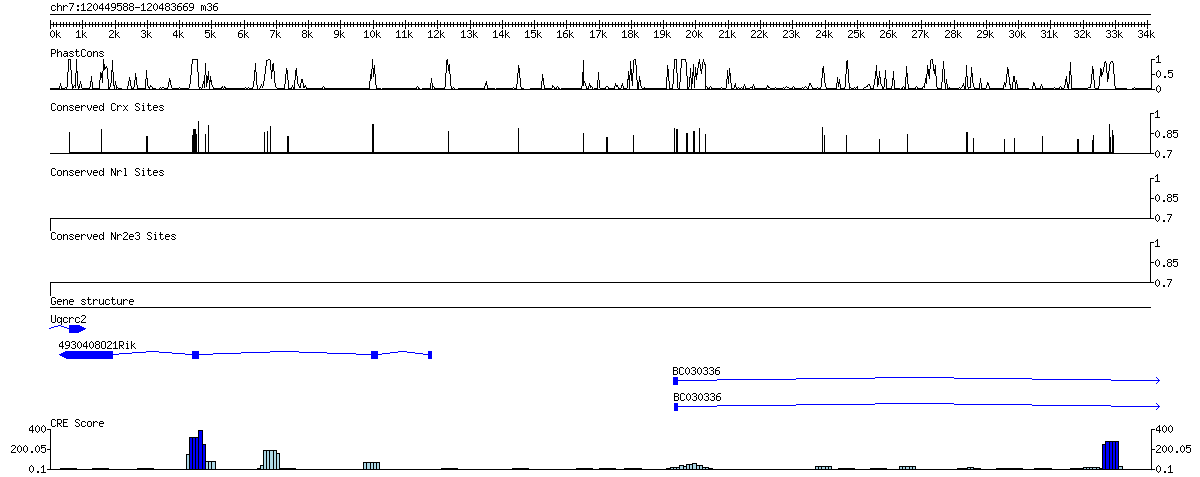

Supplement: Table S1 — Genes in the mouse photoreceptor transcription network. This table is a database which includes all genes dysregulated in Crx-/-, Nrl-/-, and/or Nr2e3-/- under high stringency criteria. Each row in the database represents a gene in the network and includes multiple links to additional types of information about that gene. ‘Large image gene +/− 15 Kb’ links to an image of our computational prediction of photoreceptor CREs in the genomic region 15 Kb upstream and downstream of the gene in question. ‘Closeup image TSS +/− 15 Kb’ links to an image of our computational prediction in the region 15 Kb on either side of the gene's TSS. Black bars within this image highlight the following regulatory peaks (if any are present): the peak closest to the TSS and the peak with the highest CRE score within this 30 Kb window. For those genes whose CRE predictions were tested experimentally, a red bar in this image indicates the location of the CRE that was tested. For Crx and Elovl4 the tested CRE is outside of this 30 Kb window and is therefore indicated in the ‘Large image’. ‘Links’ contains links to the UCSC genome browser, ENSEMBL, and NCBI database entries for the indicated gene, if available. ‘Max score (threshold of 200)’ contains the score of the highest predicted regulatory peak within the 30 Kb window around the gene's TSS. If this window does not contain any predicted peaks ≥ 200 (our cutoff threshold) no value is given (indicated by a dash). The next four columns of the table (‘Nr2e3-/-’ etc.) show the wild-type-to-mutant ratios of the averaged microarray scores for the given gene. For those genes to which more than one Affy tag correspond, the data for the first one listed under ‘Affy Mouse 430 2.0’ is given. Dark green = downregulated under high stringency (as described in METHODS); light green = downregulated under low stringency; red = upregulated under high stringency; orange = upregulated under low stringency. A dash indicates that the gene was not significantly a [file pone.0000643.s005.zip › Corbo_TableS1/images/9030407P20Rik.gif]

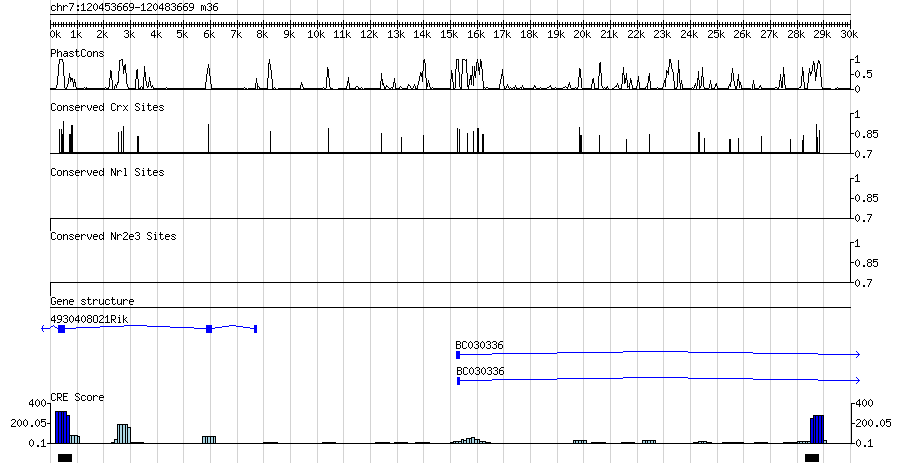

Supplement: Table S1 — Genes in the mouse photoreceptor transcription network. This table is a database which includes all genes dysregulated in Crx-/-, Nrl-/-, and/or Nr2e3-/- under high stringency criteria. Each row in the database represents a gene in the network and includes multiple links to additional types of information about that gene. ‘Large image gene +/− 15 Kb’ links to an image of our computational prediction of photoreceptor CREs in the genomic region 15 Kb upstream and downstream of the gene in question. ‘Closeup image TSS +/− 15 Kb’ links to an image of our computational prediction in the region 15 Kb on either side of the gene's TSS. Black bars within this image highlight the following regulatory peaks (if any are present): the peak closest to the TSS and the peak with the highest CRE score within this 30 Kb window. For those genes whose CRE predictions were tested experimentally, a red bar in this image indicates the location of the CRE that was tested. For Crx and Elovl4 the tested CRE is outside of this 30 Kb window and is therefore indicated in the ‘Large image’. ‘Links’ contains links to the UCSC genome browser, ENSEMBL, and NCBI database entries for the indicated gene, if available. ‘Max score (threshold of 200)’ contains the score of the highest predicted regulatory peak within the 30 Kb window around the gene's TSS. If this window does not contain any predicted peaks ≥ 200 (our cutoff threshold) no value is given (indicated by a dash). The next four columns of the table (‘Nr2e3-/-’ etc.) show the wild-type-to-mutant ratios of the averaged microarray scores for the given gene. For those genes to which more than one Affy tag correspond, the data for the first one listed under ‘Affy Mouse 430 2.0’ is given. Dark green = downregulated under high stringency (as described in METHODS); light green = downregulated under low stringency; red = upregulated under high stringency; orange = upregulated under low stringency. A dash indicates that the gene was not significantly a [file pone.0000643.s005.zip › Corbo_TableS1/images/9030407P20Rik_TSS.gif]

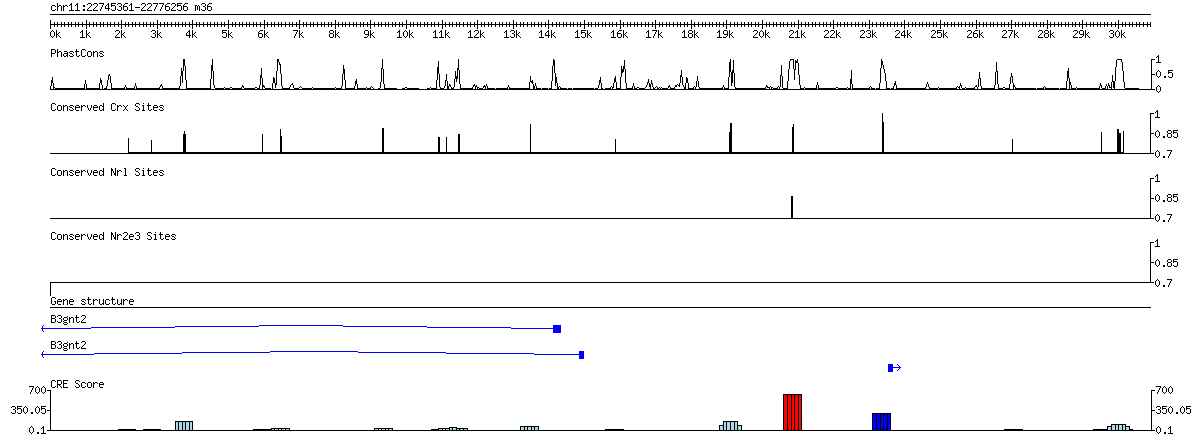

Supplement: Table S1 — Genes in the mouse photoreceptor transcription network. This table is a database which includes all genes dysregulated in Crx-/-, Nrl-/-, and/or Nr2e3-/- under high stringency criteria. Each row in the database represents a gene in the network and includes multiple links to additional types of information about that gene. ‘Large image gene +/− 15 Kb’ links to an image of our computational prediction of photoreceptor CREs in the genomic region 15 Kb upstream and downstream of the gene in question. ‘Closeup image TSS +/− 15 Kb’ links to an image of our computational prediction in the region 15 Kb on either side of the gene's TSS. Black bars within this image highlight the following regulatory peaks (if any are present): the peak closest to the TSS and the peak with the highest CRE score within this 30 Kb window. For those genes whose CRE predictions were tested experimentally, a red bar in this image indicates the location of the CRE that was tested. For Crx and Elovl4 the tested CRE is outside of this 30 Kb window and is therefore indicated in the ‘Large image’. ‘Links’ contains links to the UCSC genome browser, ENSEMBL, and NCBI database entries for the indicated gene, if available. ‘Max score (threshold of 200)’ contains the score of the highest predicted regulatory peak within the 30 Kb window around the gene's TSS. If this window does not contain any predicted peaks ≥ 200 (our cutoff threshold) no value is given (indicated by a dash). The next four columns of the table (‘Nr2e3-/-’ etc.) show the wild-type-to-mutant ratios of the averaged microarray scores for the given gene. For those genes to which more than one Affy tag correspond, the data for the first one listed under ‘Affy Mouse 430 2.0’ is given. Dark green = downregulated under high stringency (as described in METHODS); light green = downregulated under low stringency; red = upregulated under high stringency; orange = upregulated under low stringency. A dash indicates that the gene was not significantly a [file pone.0000643.s005.zip › Corbo_TableS1/images/9130230N09Rik.gif]

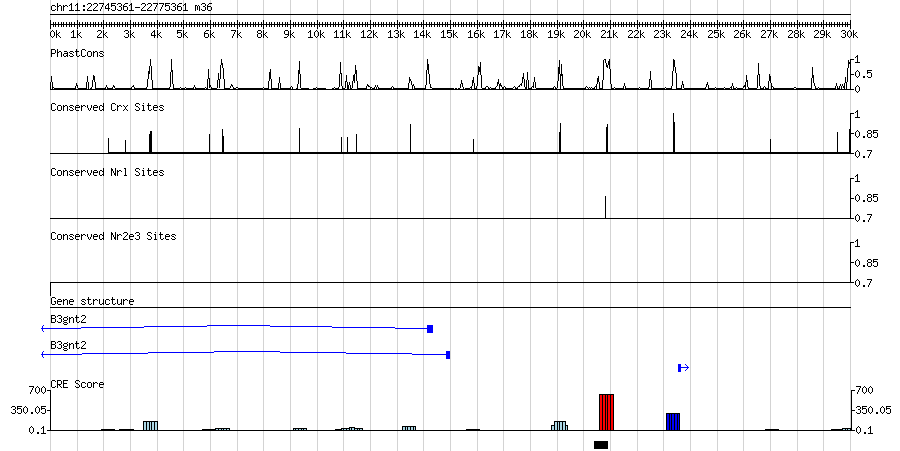

Supplement: Table S1 — Genes in the mouse photoreceptor transcription network. This table is a database which includes all genes dysregulated in Crx-/-, Nrl-/-, and/or Nr2e3-/- under high stringency criteria. Each row in the database represents a gene in the network and includes multiple links to additional types of information about that gene. ‘Large image gene +/− 15 Kb’ links to an image of our computational prediction of photoreceptor CREs in the genomic region 15 Kb upstream and downstream of the gene in question. ‘Closeup image TSS +/− 15 Kb’ links to an image of our computational prediction in the region 15 Kb on either side of the gene's TSS. Black bars within this image highlight the following regulatory peaks (if any are present): the peak closest to the TSS and the peak with the highest CRE score within this 30 Kb window. For those genes whose CRE predictions were tested experimentally, a red bar in this image indicates the location of the CRE that was tested. For Crx and Elovl4 the tested CRE is outside of this 30 Kb window and is therefore indicated in the ‘Large image’. ‘Links’ contains links to the UCSC genome browser, ENSEMBL, and NCBI database entries for the indicated gene, if available. ‘Max score (threshold of 200)’ contains the score of the highest predicted regulatory peak within the 30 Kb window around the gene's TSS. If this window does not contain any predicted peaks ≥ 200 (our cutoff threshold) no value is given (indicated by a dash). The next four columns of the table (‘Nr2e3-/-’ etc.) show the wild-type-to-mutant ratios of the averaged microarray scores for the given gene. For those genes to which more than one Affy tag correspond, the data for the first one listed under ‘Affy Mouse 430 2.0’ is given. Dark green = downregulated under high stringency (as described in METHODS); light green = downregulated under low stringency; red = upregulated under high stringency; orange = upregulated under low stringency. A dash indicates that the gene was not significantly a [file pone.0000643.s005.zip › Corbo_TableS1/images/9130230N09Rik_TSS.gif]

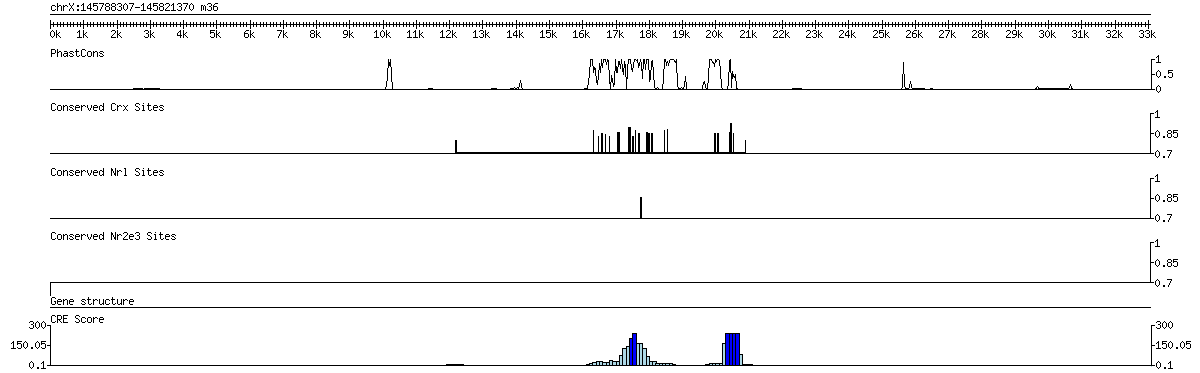

Supplement: Table S1 — Genes in the mouse photoreceptor transcription network. This table is a database which includes all genes dysregulated in Crx-/-, Nrl-/-, and/or Nr2e3-/- under high stringency criteria. Each row in the database represents a gene in the network and includes multiple links to additional types of information about that gene. ‘Large image gene +/− 15 Kb’ links to an image of our computational prediction of photoreceptor CREs in the genomic region 15 Kb upstream and downstream of the gene in question. ‘Closeup image TSS +/− 15 Kb’ links to an image of our computational prediction in the region 15 Kb on either side of the gene's TSS. Black bars within this image highlight the following regulatory peaks (if any are present): the peak closest to the TSS and the peak with the highest CRE score within this 30 Kb window. For those genes whose CRE predictions were tested experimentally, a red bar in this image indicates the location of the CRE that was tested. For Crx and Elovl4 the tested CRE is outside of this 30 Kb window and is therefore indicated in the ‘Large image’. ‘Links’ contains links to the UCSC genome browser, ENSEMBL, and NCBI database entries for the indicated gene, if available. ‘Max score (threshold of 200)’ contains the score of the highest predicted regulatory peak within the 30 Kb window around the gene's TSS. If this window does not contain any predicted peaks ≥ 200 (our cutoff threshold) no value is given (indicated by a dash). The next four columns of the table (‘Nr2e3-/-’ etc.) show the wild-type-to-mutant ratios of the averaged microarray scores for the given gene. For those genes to which more than one Affy tag correspond, the data for the first one listed under ‘Affy Mouse 430 2.0’ is given. Dark green = downregulated under high stringency (as described in METHODS); light green = downregulated under low stringency; red = upregulated under high stringency; orange = upregulated under low stringency. A dash indicates that the gene was not significantly a [file pone.0000643.s005.zip › Corbo_TableS1/images/9330168M11Rik.gif]

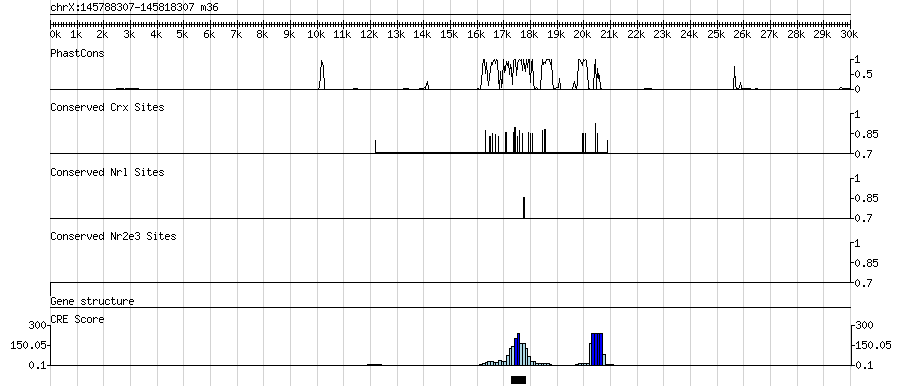

Supplement: Table S1 — Genes in the mouse photoreceptor transcription network. This table is a database which includes all genes dysregulated in Crx-/-, Nrl-/-, and/or Nr2e3-/- under high stringency criteria. Each row in the database represents a gene in the network and includes multiple links to additional types of information about that gene. ‘Large image gene +/− 15 Kb’ links to an image of our computational prediction of photoreceptor CREs in the genomic region 15 Kb upstream and downstream of the gene in question. ‘Closeup image TSS +/− 15 Kb’ links to an image of our computational prediction in the region 15 Kb on either side of the gene's TSS. Black bars within this image highlight the following regulatory peaks (if any are present): the peak closest to the TSS and the peak with the highest CRE score within this 30 Kb window. For those genes whose CRE predictions were tested experimentally, a red bar in this image indicates the location of the CRE that was tested. For Crx and Elovl4 the tested CRE is outside of this 30 Kb window and is therefore indicated in the ‘Large image’. ‘Links’ contains links to the UCSC genome browser, ENSEMBL, and NCBI database entries for the indicated gene, if available. ‘Max score (threshold of 200)’ contains the score of the highest predicted regulatory peak within the 30 Kb window around the gene's TSS. If this window does not contain any predicted peaks ≥ 200 (our cutoff threshold) no value is given (indicated by a dash). The next four columns of the table (‘Nr2e3-/-’ etc.) show the wild-type-to-mutant ratios of the averaged microarray scores for the given gene. For those genes to which more than one Affy tag correspond, the data for the first one listed under ‘Affy Mouse 430 2.0’ is given. Dark green = downregulated under high stringency (as described in METHODS); light green = downregulated under low stringency; red = upregulated under high stringency; orange = upregulated under low stringency. A dash indicates that the gene was not significantly a [file pone.0000643.s005.zip › Corbo_TableS1/images/9330168M11Rik_TSS.gif]

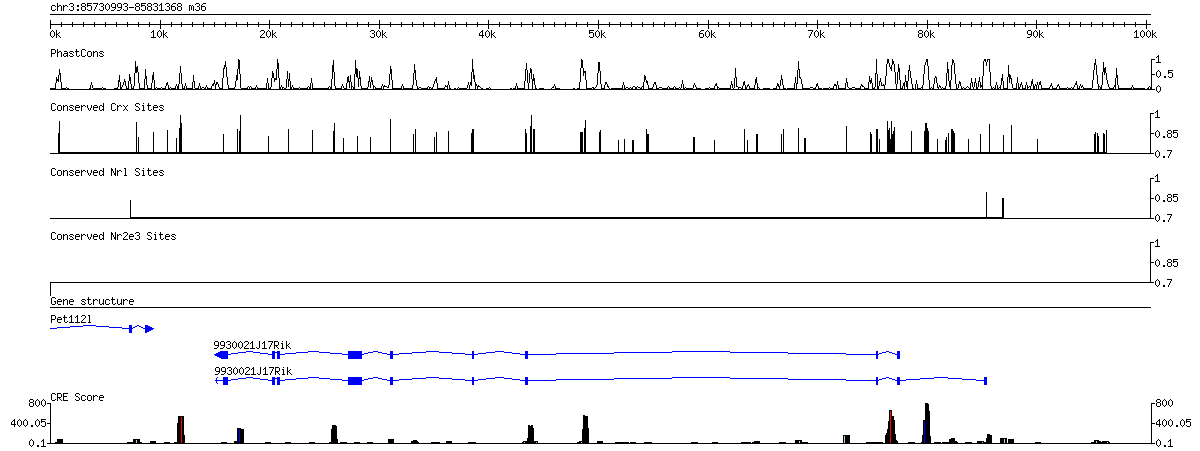

Supplement: Table S1 — Genes in the mouse photoreceptor transcription network. This table is a database which includes all genes dysregulated in Crx-/-, Nrl-/-, and/or Nr2e3-/- under high stringency criteria. Each row in the database represents a gene in the network and includes multiple links to additional types of information about that gene. ‘Large image gene +/− 15 Kb’ links to an image of our computational prediction of photoreceptor CREs in the genomic region 15 Kb upstream and downstream of the gene in question. ‘Closeup image TSS +/− 15 Kb’ links to an image of our computational prediction in the region 15 Kb on either side of the gene's TSS. Black bars within this image highlight the following regulatory peaks (if any are present): the peak closest to the TSS and the peak with the highest CRE score within this 30 Kb window. For those genes whose CRE predictions were tested experimentally, a red bar in this image indicates the location of the CRE that was tested. For Crx and Elovl4 the tested CRE is outside of this 30 Kb window and is therefore indicated in the ‘Large image’. ‘Links’ contains links to the UCSC genome browser, ENSEMBL, and NCBI database entries for the indicated gene, if available. ‘Max score (threshold of 200)’ contains the score of the highest predicted regulatory peak within the 30 Kb window around the gene's TSS. If this window does not contain any predicted peaks ≥ 200 (our cutoff threshold) no value is given (indicated by a dash). The next four columns of the table (‘Nr2e3-/-’ etc.) show the wild-type-to-mutant ratios of the averaged microarray scores for the given gene. For those genes to which more than one Affy tag correspond, the data for the first one listed under ‘Affy Mouse 430 2.0’ is given. Dark green = downregulated under high stringency (as described in METHODS); light green = downregulated under low stringency; red = upregulated under high stringency; orange = upregulated under low stringency. A dash indicates that the gene was not significantly a [file pone.0000643.s005.zip › Corbo_TableS1/images/9930021J17Rik.gif]

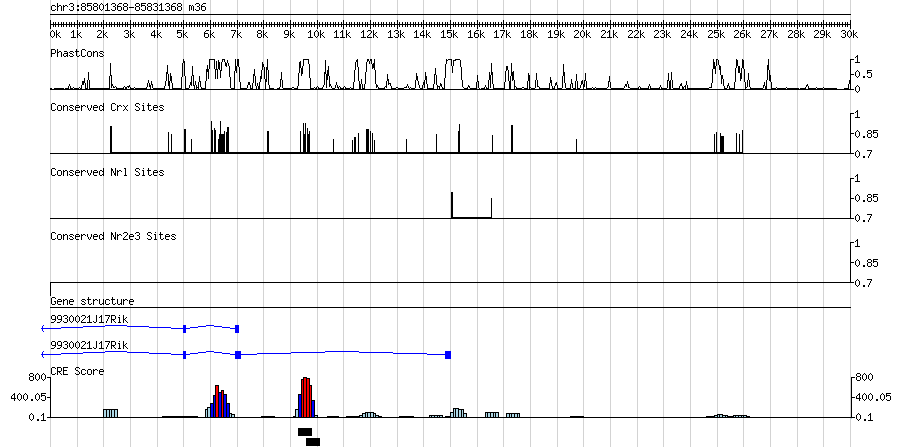

Supplement: Table S1 — Genes in the mouse photoreceptor transcription network. This table is a database which includes all genes dysregulated in Crx-/-, Nrl-/-, and/or Nr2e3-/- under high stringency criteria. Each row in the database represents a gene in the network and includes multiple links to additional types of information about that gene. ‘Large image gene +/− 15 Kb’ links to an image of our computational prediction of photoreceptor CREs in the genomic region 15 Kb upstream and downstream of the gene in question. ‘Closeup image TSS +/− 15 Kb’ links to an image of our computational prediction in the region 15 Kb on either side of the gene's TSS. Black bars within this image highlight the following regulatory peaks (if any are present): the peak closest to the TSS and the peak with the highest CRE score within this 30 Kb window. For those genes whose CRE predictions were tested experimentally, a red bar in this image indicates the location of the CRE that was tested. For Crx and Elovl4 the tested CRE is outside of this 30 Kb window and is therefore indicated in the ‘Large image’. ‘Links’ contains links to the UCSC genome browser, ENSEMBL, and NCBI database entries for the indicated gene, if available. ‘Max score (threshold of 200)’ contains the score of the highest predicted regulatory peak within the 30 Kb window around the gene's TSS. If this window does not contain any predicted peaks ≥ 200 (our cutoff threshold) no value is given (indicated by a dash). The next four columns of the table (‘Nr2e3-/-’ etc.) show the wild-type-to-mutant ratios of the averaged microarray scores for the given gene. For those genes to which more than one Affy tag correspond, the data for the first one listed under ‘Affy Mouse 430 2.0’ is given. Dark green = downregulated under high stringency (as described in METHODS); light green = downregulated under low stringency; red = upregulated under high stringency; orange = upregulated under low stringency. A dash indicates that the gene was not significantly a [file pone.0000643.s005.zip › Corbo_TableS1/images/9930021J17Rik_TSS.gif]

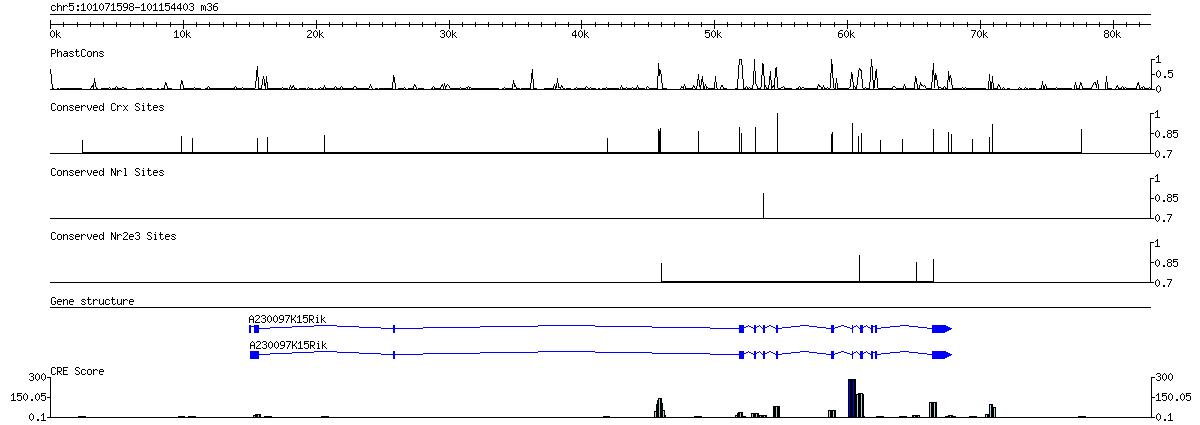

Supplement: Table S1 — Genes in the mouse photoreceptor transcription network. This table is a database which includes all genes dysregulated in Crx-/-, Nrl-/-, and/or Nr2e3-/- under high stringency criteria. Each row in the database represents a gene in the network and includes multiple links to additional types of information about that gene. ‘Large image gene +/− 15 Kb’ links to an image of our computational prediction of photoreceptor CREs in the genomic region 15 Kb upstream and downstream of the gene in question. ‘Closeup image TSS +/− 15 Kb’ links to an image of our computational prediction in the region 15 Kb on either side of the gene's TSS. Black bars within this image highlight the following regulatory peaks (if any are present): the peak closest to the TSS and the peak with the highest CRE score within this 30 Kb window. For those genes whose CRE predictions were tested experimentally, a red bar in this image indicates the location of the CRE that was tested. For Crx and Elovl4 the tested CRE is outside of this 30 Kb window and is therefore indicated in the ‘Large image’. ‘Links’ contains links to the UCSC genome browser, ENSEMBL, and NCBI database entries for the indicated gene, if available. ‘Max score (threshold of 200)’ contains the score of the highest predicted regulatory peak within the 30 Kb window around the gene's TSS. If this window does not contain any predicted peaks ≥ 200 (our cutoff threshold) no value is given (indicated by a dash). The next four columns of the table (‘Nr2e3-/-’ etc.) show the wild-type-to-mutant ratios of the averaged microarray scores for the given gene. For those genes to which more than one Affy tag correspond, the data for the first one listed under ‘Affy Mouse 430 2.0’ is given. Dark green = downregulated under high stringency (as described in METHODS); light green = downregulated under low stringency; red = upregulated under high stringency; orange = upregulated under low stringency. A dash indicates that the gene was not significantly a [file pone.0000643.s005.zip › Corbo_TableS1/images/A230097K15Rik.gif]

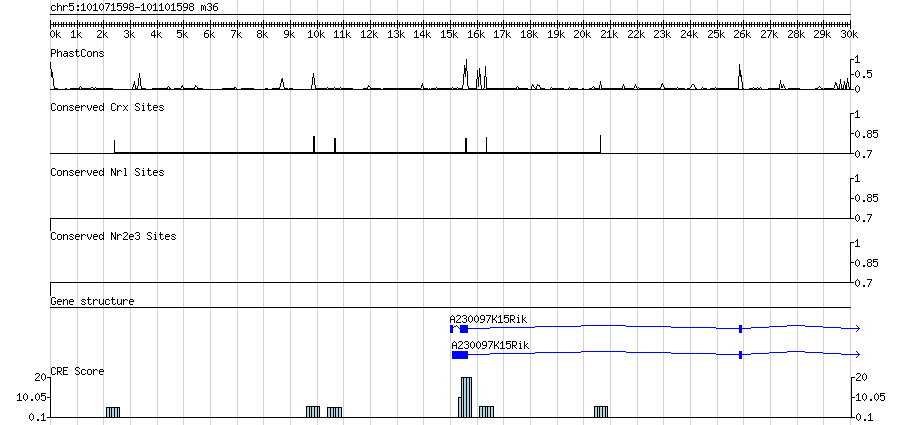

Supplement: Table S1 — Genes in the mouse photoreceptor transcription network. This table is a database which includes all genes dysregulated in Crx-/-, Nrl-/-, and/or Nr2e3-/- under high stringency criteria. Each row in the database represents a gene in the network and includes multiple links to additional types of information about that gene. ‘Large image gene +/− 15 Kb’ links to an image of our computational prediction of photoreceptor CREs in the genomic region 15 Kb upstream and downstream of the gene in question. ‘Closeup image TSS +/− 15 Kb’ links to an image of our computational prediction in the region 15 Kb on either side of the gene's TSS. Black bars within this image highlight the following regulatory peaks (if any are present): the peak closest to the TSS and the peak with the highest CRE score within this 30 Kb window. For those genes whose CRE predictions were tested experimentally, a red bar in this image indicates the location of the CRE that was tested. For Crx and Elovl4 the tested CRE is outside of this 30 Kb window and is therefore indicated in the ‘Large image’. ‘Links’ contains links to the UCSC genome browser, ENSEMBL, and NCBI database entries for the indicated gene, if available. ‘Max score (threshold of 200)’ contains the score of the highest predicted regulatory peak within the 30 Kb window around the gene's TSS. If this window does not contain any predicted peaks ≥ 200 (our cutoff threshold) no value is given (indicated by a dash). The next four columns of the table (‘Nr2e3-/-’ etc.) show the wild-type-to-mutant ratios of the averaged microarray scores for the given gene. For those genes to which more than one Affy tag correspond, the data for the first one listed under ‘Affy Mouse 430 2.0’ is given. Dark green = downregulated under high stringency (as described in METHODS); light green = downregulated under low stringency; red = upregulated under high stringency; orange = upregulated under low stringency. A dash indicates that the gene was not significantly a [file pone.0000643.s005.zip › Corbo_TableS1/images/A230097K15Rik_TSS.gif]

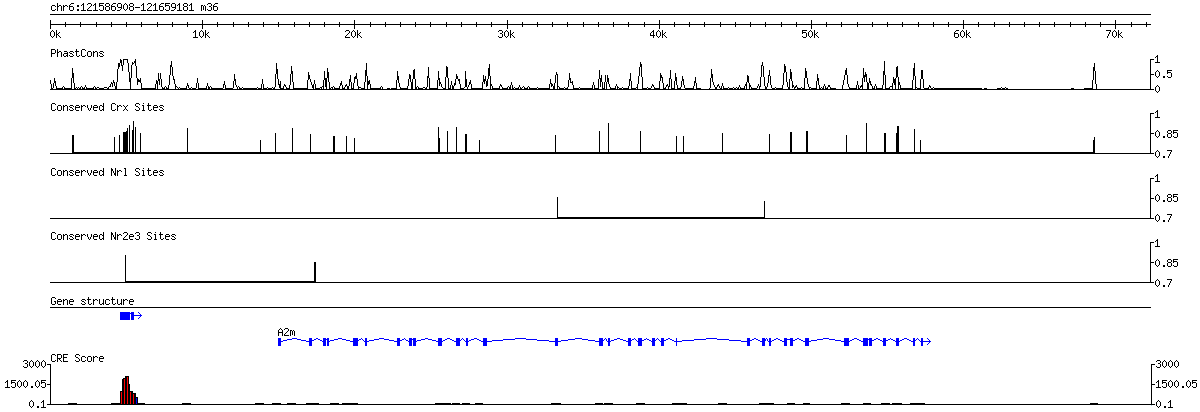

Supplement: Table S1 — Genes in the mouse photoreceptor transcription network. This table is a database which includes all genes dysregulated in Crx-/-, Nrl-/-, and/or Nr2e3-/- under high stringency criteria. Each row in the database represents a gene in the network and includes multiple links to additional types of information about that gene. ‘Large image gene +/− 15 Kb’ links to an image of our computational prediction of photoreceptor CREs in the genomic region 15 Kb upstream and downstream of the gene in question. ‘Closeup image TSS +/− 15 Kb’ links to an image of our computational prediction in the region 15 Kb on either side of the gene's TSS. Black bars within this image highlight the following regulatory peaks (if any are present): the peak closest to the TSS and the peak with the highest CRE score within this 30 Kb window. For those genes whose CRE predictions were tested experimentally, a red bar in this image indicates the location of the CRE that was tested. For Crx and Elovl4 the tested CRE is outside of this 30 Kb window and is therefore indicated in the ‘Large image’. ‘Links’ contains links to the UCSC genome browser, ENSEMBL, and NCBI database entries for the indicated gene, if available. ‘Max score (threshold of 200)’ contains the score of the highest predicted regulatory peak within the 30 Kb window around the gene's TSS. If this window does not contain any predicted peaks ≥ 200 (our cutoff threshold) no value is given (indicated by a dash). The next four columns of the table (‘Nr2e3-/-’ etc.) show the wild-type-to-mutant ratios of the averaged microarray scores for the given gene. For those genes to which more than one Affy tag correspond, the data for the first one listed under ‘Affy Mouse 430 2.0’ is given. Dark green = downregulated under high stringency (as described in METHODS); light green = downregulated under low stringency; red = upregulated under high stringency; orange = upregulated under low stringency. A dash indicates that the gene was not significantly a [file pone.0000643.s005.zip › Corbo_TableS1/images/A2m.gif]

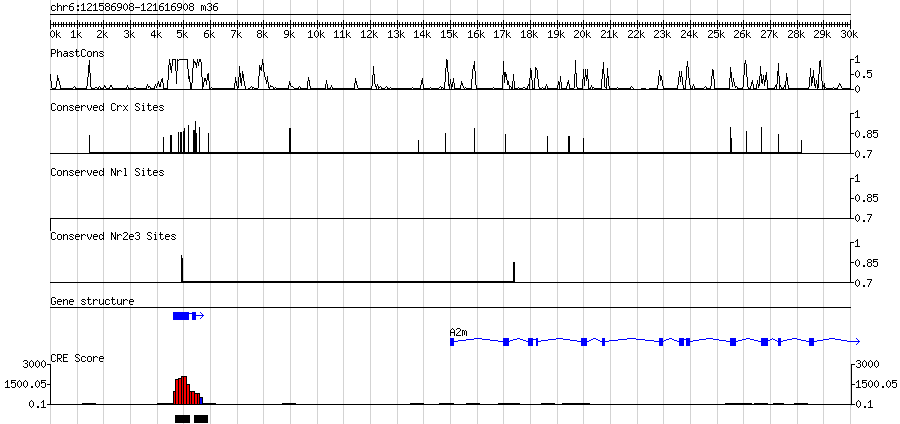

Supplement: Table S1 — Genes in the mouse photoreceptor transcription network. This table is a database which includes all genes dysregulated in Crx-/-, Nrl-/-, and/or Nr2e3-/- under high stringency criteria. Each row in the database represents a gene in the network and includes multiple links to additional types of information about that gene. ‘Large image gene +/− 15 Kb’ links to an image of our computational prediction of photoreceptor CREs in the genomic region 15 Kb upstream and downstream of the gene in question. ‘Closeup image TSS +/− 15 Kb’ links to an image of our computational prediction in the region 15 Kb on either side of the gene's TSS. Black bars within this image highlight the following regulatory peaks (if any are present): the peak closest to the TSS and the peak with the highest CRE score within this 30 Kb window. For those genes whose CRE predictions were tested experimentally, a red bar in this image indicates the location of the CRE that was tested. For Crx and Elovl4 the tested CRE is outside of this 30 Kb window and is therefore indicated in the ‘Large image’. ‘Links’ contains links to the UCSC genome browser, ENSEMBL, and NCBI database entries for the indicated gene, if available. ‘Max score (threshold of 200)’ contains the score of the highest predicted regulatory peak within the 30 Kb window around the gene's TSS. If this window does not contain any predicted peaks ≥ 200 (our cutoff threshold) no value is given (indicated by a dash). The next four columns of the table (‘Nr2e3-/-’ etc.) show the wild-type-to-mutant ratios of the averaged microarray scores for the given gene. For those genes to which more than one Affy tag correspond, the data for the first one listed under ‘Affy Mouse 430 2.0’ is given. Dark green = downregulated under high stringency (as described in METHODS); light green = downregulated under low stringency; red = upregulated under high stringency; orange = upregulated under low stringency. A dash indicates that the gene was not significantly a [file pone.0000643.s005.zip › Corbo_TableS1/images/A2m_TSS.gif]

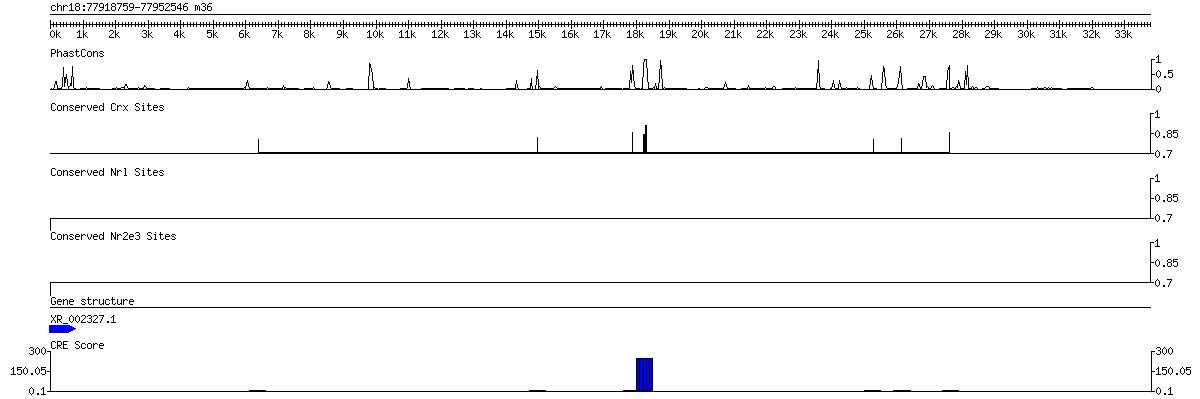

Supplement: Table S1 — Genes in the mouse photoreceptor transcription network. This table is a database which includes all genes dysregulated in Crx-/-, Nrl-/-, and/or Nr2e3-/- under high stringency criteria. Each row in the database represents a gene in the network and includes multiple links to additional types of information about that gene. ‘Large image gene +/− 15 Kb’ links to an image of our computational prediction of photoreceptor CREs in the genomic region 15 Kb upstream and downstream of the gene in question. ‘Closeup image TSS +/− 15 Kb’ links to an image of our computational prediction in the region 15 Kb on either side of the gene's TSS. Black bars within this image highlight the following regulatory peaks (if any are present): the peak closest to the TSS and the peak with the highest CRE score within this 30 Kb window. For those genes whose CRE predictions were tested experimentally, a red bar in this image indicates the location of the CRE that was tested. For Crx and Elovl4 the tested CRE is outside of this 30 Kb window and is therefore indicated in the ‘Large image’. ‘Links’ contains links to the UCSC genome browser, ENSEMBL, and NCBI database entries for the indicated gene, if available. ‘Max score (threshold of 200)’ contains the score of the highest predicted regulatory peak within the 30 Kb window around the gene's TSS. If this window does not contain any predicted peaks ≥ 200 (our cutoff threshold) no value is given (indicated by a dash). The next four columns of the table (‘Nr2e3-/-’ etc.) show the wild-type-to-mutant ratios of the averaged microarray scores for the given gene. For those genes to which more than one Affy tag correspond, the data for the first one listed under ‘Affy Mouse 430 2.0’ is given. Dark green = downregulated under high stringency (as described in METHODS); light green = downregulated under low stringency; red = upregulated under high stringency; orange = upregulated under low stringency. A dash indicates that the gene was not significantly a [file pone.0000643.s005.zip › Corbo_TableS1/images/A330094K24Rik.gif]

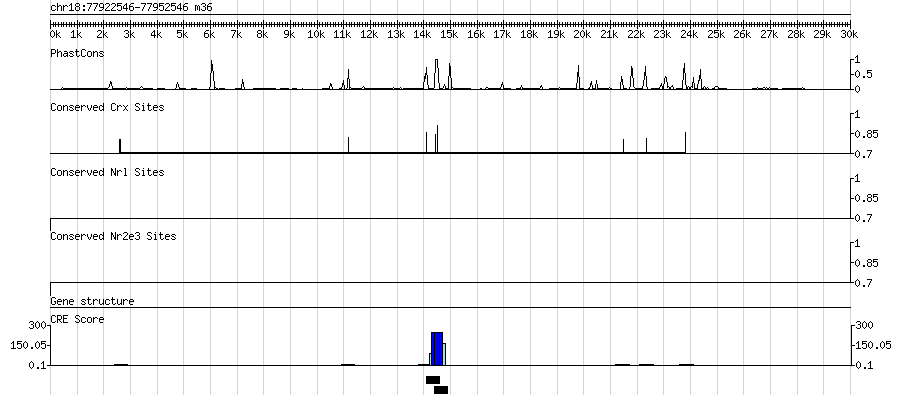

Supplement: Table S1 — Genes in the mouse photoreceptor transcription network. This table is a database which includes all genes dysregulated in Crx-/-, Nrl-/-, and/or Nr2e3-/- under high stringency criteria. Each row in the database represents a gene in the network and includes multiple links to additional types of information about that gene. ‘Large image gene +/− 15 Kb’ links to an image of our computational prediction of photoreceptor CREs in the genomic region 15 Kb upstream and downstream of the gene in question. ‘Closeup image TSS +/− 15 Kb’ links to an image of our computational prediction in the region 15 Kb on either side of the gene's TSS. Black bars within this image highlight the following regulatory peaks (if any are present): the peak closest to the TSS and the peak with the highest CRE score within this 30 Kb window. For those genes whose CRE predictions were tested experimentally, a red bar in this image indicates the location of the CRE that was tested. For Crx and Elovl4 the tested CRE is outside of this 30 Kb window and is therefore indicated in the ‘Large image’. ‘Links’ contains links to the UCSC genome browser, ENSEMBL, and NCBI database entries for the indicated gene, if available. ‘Max score (threshold of 200)’ contains the score of the highest predicted regulatory peak within the 30 Kb window around the gene's TSS. If this window does not contain any predicted peaks ≥ 200 (our cutoff threshold) no value is given (indicated by a dash). The next four columns of the table (‘Nr2e3-/-’ etc.) show the wild-type-to-mutant ratios of the averaged microarray scores for the given gene. For those genes to which more than one Affy tag correspond, the data for the first one listed under ‘Affy Mouse 430 2.0’ is given. Dark green = downregulated under high stringency (as described in METHODS); light green = downregulated under low stringency; red = upregulated under high stringency; orange = upregulated under low stringency. A dash indicates that the gene was not significantly a [file pone.0000643.s005.zip › Corbo_TableS1/images/A330094K24Rik_TSS.gif]

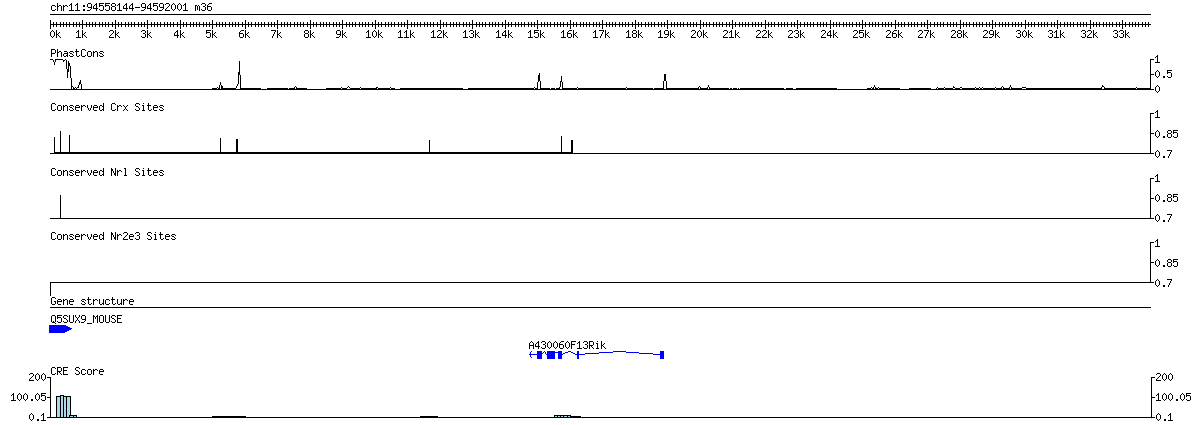

Supplement: Table S1 — Genes in the mouse photoreceptor transcription network. This table is a database which includes all genes dysregulated in Crx-/-, Nrl-/-, and/or Nr2e3-/- under high stringency criteria. Each row in the database represents a gene in the network and includes multiple links to additional types of information about that gene. ‘Large image gene +/− 15 Kb’ links to an image of our computational prediction of photoreceptor CREs in the genomic region 15 Kb upstream and downstream of the gene in question. ‘Closeup image TSS +/− 15 Kb’ links to an image of our computational prediction in the region 15 Kb on either side of the gene's TSS. Black bars within this image highlight the following regulatory peaks (if any are present): the peak closest to the TSS and the peak with the highest CRE score within this 30 Kb window. For those genes whose CRE predictions were tested experimentally, a red bar in this image indicates the location of the CRE that was tested. For Crx and Elovl4 the tested CRE is outside of this 30 Kb window and is therefore indicated in the ‘Large image’. ‘Links’ contains links to the UCSC genome browser, ENSEMBL, and NCBI database entries for the indicated gene, if available. ‘Max score (threshold of 200)’ contains the score of the highest predicted regulatory peak within the 30 Kb window around the gene's TSS. If this window does not contain any predicted peaks ≥ 200 (our cutoff threshold) no value is given (indicated by a dash). The next four columns of the table (‘Nr2e3-/-’ etc.) show the wild-type-to-mutant ratios of the averaged microarray scores for the given gene. For those genes to which more than one Affy tag correspond, the data for the first one listed under ‘Affy Mouse 430 2.0’ is given. Dark green = downregulated under high stringency (as described in METHODS); light green = downregulated under low stringency; red = upregulated under high stringency; orange = upregulated under low stringency. A dash indicates that the gene was not significantly a [file pone.0000643.s005.zip › Corbo_TableS1/images/A430060F13Rik.gif]

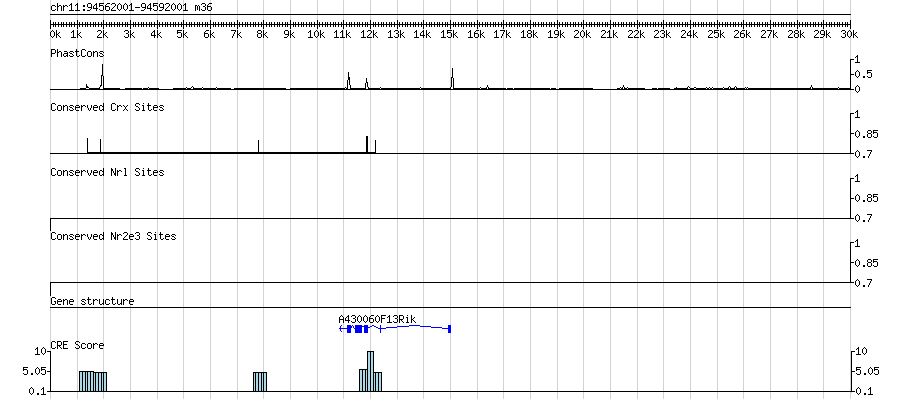

Supplement: Table S1 — Genes in the mouse photoreceptor transcription network. This table is a database which includes all genes dysregulated in Crx-/-, Nrl-/-, and/or Nr2e3-/- under high stringency criteria. Each row in the database represents a gene in the network and includes multiple links to additional types of information about that gene. ‘Large image gene +/− 15 Kb’ links to an image of our computational prediction of photoreceptor CREs in the genomic region 15 Kb upstream and downstream of the gene in question. ‘Closeup image TSS +/− 15 Kb’ links to an image of our computational prediction in the region 15 Kb on either side of the gene's TSS. Black bars within this image highlight the following regulatory peaks (if any are present): the peak closest to the TSS and the peak with the highest CRE score within this 30 Kb window. For those genes whose CRE predictions were tested experimentally, a red bar in this image indicates the location of the CRE that was tested. For Crx and Elovl4 the tested CRE is outside of this 30 Kb window and is therefore indicated in the ‘Large image’. ‘Links’ contains links to the UCSC genome browser, ENSEMBL, and NCBI database entries for the indicated gene, if available. ‘Max score (threshold of 200)’ contains the score of the highest predicted regulatory peak within the 30 Kb window around the gene's TSS. If this window does not contain any predicted peaks ≥ 200 (our cutoff threshold) no value is given (indicated by a dash). The next four columns of the table (‘Nr2e3-/-’ etc.) show the wild-type-to-mutant ratios of the averaged microarray scores for the given gene. For those genes to which more than one Affy tag correspond, the data for the first one listed under ‘Affy Mouse 430 2.0’ is given. Dark green = downregulated under high stringency (as described in METHODS); light green = downregulated under low stringency; red = upregulated under high stringency; orange = upregulated under low stringency. A dash indicates that the gene was not significantly a [file pone.0000643.s005.zip › Corbo_TableS1/images/A430060F13Rik_TSS.gif]

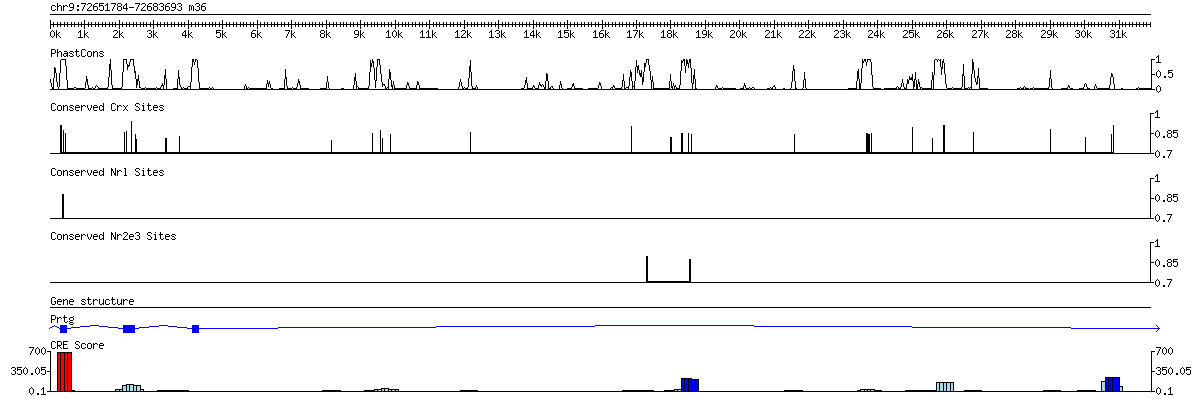

Supplement: Table S1 — Genes in the mouse photoreceptor transcription network. This table is a database which includes all genes dysregulated in Crx-/-, Nrl-/-, and/or Nr2e3-/- under high stringency criteria. Each row in the database represents a gene in the network and includes multiple links to additional types of information about that gene. ‘Large image gene +/− 15 Kb’ links to an image of our computational prediction of photoreceptor CREs in the genomic region 15 Kb upstream and downstream of the gene in question. ‘Closeup image TSS +/− 15 Kb’ links to an image of our computational prediction in the region 15 Kb on either side of the gene's TSS. Black bars within this image highlight the following regulatory peaks (if any are present): the peak closest to the TSS and the peak with the highest CRE score within this 30 Kb window. For those genes whose CRE predictions were tested experimentally, a red bar in this image indicates the location of the CRE that was tested. For Crx and Elovl4 the tested CRE is outside of this 30 Kb window and is therefore indicated in the ‘Large image’. ‘Links’ contains links to the UCSC genome browser, ENSEMBL, and NCBI database entries for the indicated gene, if available. ‘Max score (threshold of 200)’ contains the score of the highest predicted regulatory peak within the 30 Kb window around the gene's TSS. If this window does not contain any predicted peaks ≥ 200 (our cutoff threshold) no value is given (indicated by a dash). The next four columns of the table (‘Nr2e3-/-’ etc.) show the wild-type-to-mutant ratios of the averaged microarray scores for the given gene. For those genes to which more than one Affy tag correspond, the data for the first one listed under ‘Affy Mouse 430 2.0’ is given. Dark green = downregulated under high stringency (as described in METHODS); light green = downregulated under low stringency; red = upregulated under high stringency; orange = upregulated under low stringency. A dash indicates that the gene was not significantly a [file pone.0000643.s005.zip › Corbo_TableS1/images/A730062M13Rik.gif]

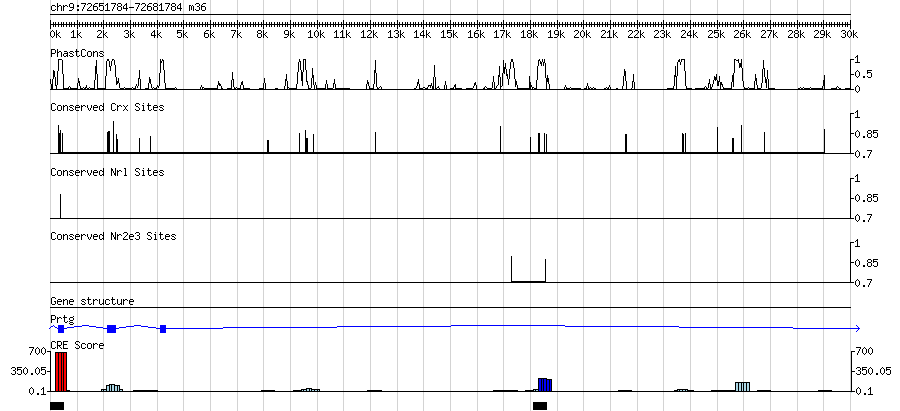

Supplement: Table S1 — Genes in the mouse photoreceptor transcription network. This table is a database which includes all genes dysregulated in Crx-/-, Nrl-/-, and/or Nr2e3-/- under high stringency criteria. Each row in the database represents a gene in the network and includes multiple links to additional types of information about that gene. ‘Large image gene +/− 15 Kb’ links to an image of our computational prediction of photoreceptor CREs in the genomic region 15 Kb upstream and downstream of the gene in question. ‘Closeup image TSS +/− 15 Kb’ links to an image of our computational prediction in the region 15 Kb on either side of the gene's TSS. Black bars within this image highlight the following regulatory peaks (if any are present): the peak closest to the TSS and the peak with the highest CRE score within this 30 Kb window. For those genes whose CRE predictions were tested experimentally, a red bar in this image indicates the location of the CRE that was tested. For Crx and Elovl4 the tested CRE is outside of this 30 Kb window and is therefore indicated in the ‘Large image’. ‘Links’ contains links to the UCSC genome browser, ENSEMBL, and NCBI database entries for the indicated gene, if available. ‘Max score (threshold of 200)’ contains the score of the highest predicted regulatory peak within the 30 Kb window around the gene's TSS. If this window does not contain any predicted peaks ≥ 200 (our cutoff threshold) no value is given (indicated by a dash). The next four columns of the table (‘Nr2e3-/-’ etc.) show the wild-type-to-mutant ratios of the averaged microarray scores for the given gene. For those genes to which more than one Affy tag correspond, the data for the first one listed under ‘Affy Mouse 430 2.0’ is given. Dark green = downregulated under high stringency (as described in METHODS); light green = downregulated under low stringency; red = upregulated under high stringency; orange = upregulated under low stringency. A dash indicates that the gene was not significantly a [file pone.0000643.s005.zip › Corbo_TableS1/images/A730062M13Rik_TSS.gif]

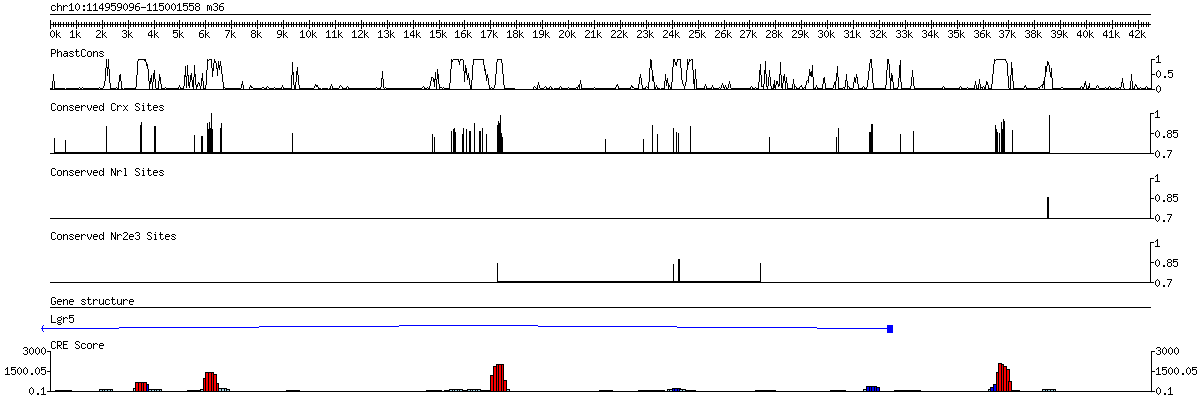

Supplement: Table S1 — Genes in the mouse photoreceptor transcription network. This table is a database which includes all genes dysregulated in Crx-/-, Nrl-/-, and/or Nr2e3-/- under high stringency criteria. Each row in the database represents a gene in the network and includes multiple links to additional types of information about that gene. ‘Large image gene +/− 15 Kb’ links to an image of our computational prediction of photoreceptor CREs in the genomic region 15 Kb upstream and downstream of the gene in question. ‘Closeup image TSS +/− 15 Kb’ links to an image of our computational prediction in the region 15 Kb on either side of the gene's TSS. Black bars within this image highlight the following regulatory peaks (if any are present): the peak closest to the TSS and the peak with the highest CRE score within this 30 Kb window. For those genes whose CRE predictions were tested experimentally, a red bar in this image indicates the location of the CRE that was tested. For Crx and Elovl4 the tested CRE is outside of this 30 Kb window and is therefore indicated in the ‘Large image’. ‘Links’ contains links to the UCSC genome browser, ENSEMBL, and NCBI database entries for the indicated gene, if available. ‘Max score (threshold of 200)’ contains the score of the highest predicted regulatory peak within the 30 Kb window around the gene's TSS. If this window does not contain any predicted peaks ≥ 200 (our cutoff threshold) no value is given (indicated by a dash). The next four columns of the table (‘Nr2e3-/-’ etc.) show the wild-type-to-mutant ratios of the averaged microarray scores for the given gene. For those genes to which more than one Affy tag correspond, the data for the first one listed under ‘Affy Mouse 430 2.0’ is given. Dark green = downregulated under high stringency (as described in METHODS); light green = downregulated under low stringency; red = upregulated under high stringency; orange = upregulated under low stringency. A dash indicates that the gene was not significantly a [file pone.0000643.s005.zip › Corbo_TableS1/images/A930009A15Rik.gif]

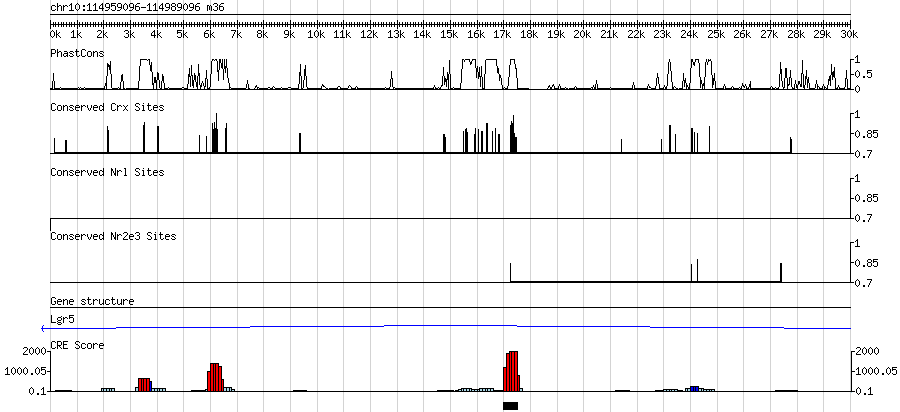

Supplement: Table S1 — Genes in the mouse photoreceptor transcription network. This table is a database which includes all genes dysregulated in Crx-/-, Nrl-/-, and/or Nr2e3-/- under high stringency criteria. Each row in the database represents a gene in the network and includes multiple links to additional types of information about that gene. ‘Large image gene +/− 15 Kb’ links to an image of our computational prediction of photoreceptor CREs in the genomic region 15 Kb upstream and downstream of the gene in question. ‘Closeup image TSS +/− 15 Kb’ links to an image of our computational prediction in the region 15 Kb on either side of the gene's TSS. Black bars within this image highlight the following regulatory peaks (if any are present): the peak closest to the TSS and the peak with the highest CRE score within this 30 Kb window. For those genes whose CRE predictions were tested experimentally, a red bar in this image indicates the location of the CRE that was tested. For Crx and Elovl4 the tested CRE is outside of this 30 Kb window and is therefore indicated in the ‘Large image’. ‘Links’ contains links to the UCSC genome browser, ENSEMBL, and NCBI database entries for the indicated gene, if available. ‘Max score (threshold of 200)’ contains the score of the highest predicted regulatory peak within the 30 Kb window around the gene's TSS. If this window does not contain any predicted peaks ≥ 200 (our cutoff threshold) no value is given (indicated by a dash). The next four columns of the table (‘Nr2e3-/-’ etc.) show the wild-type-to-mutant ratios of the averaged microarray scores for the given gene. For those genes to which more than one Affy tag correspond, the data for the first one listed under ‘Affy Mouse 430 2.0’ is given. Dark green = downregulated under high stringency (as described in METHODS); light green = downregulated under low stringency; red = upregulated under high stringency; orange = upregulated under low stringency. A dash indicates that the gene was not significantly a [file pone.0000643.s005.zip › Corbo_TableS1/images/A930009A15Rik_TSS.gif]

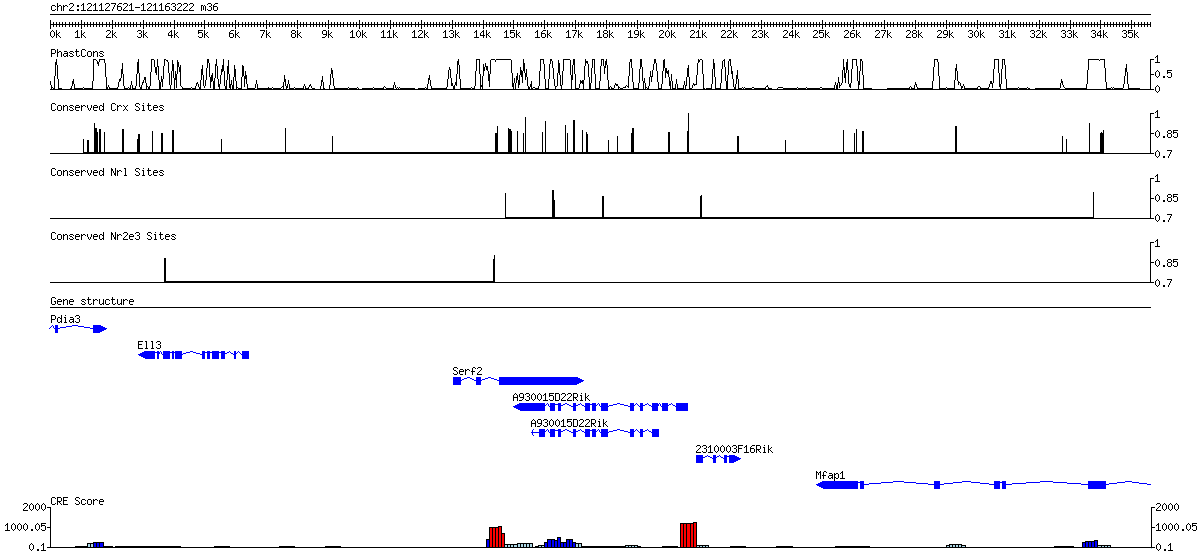

Supplement: Table S1 — Genes in the mouse photoreceptor transcription network. This table is a database which includes all genes dysregulated in Crx-/-, Nrl-/-, and/or Nr2e3-/- under high stringency criteria. Each row in the database represents a gene in the network and includes multiple links to additional types of information about that gene. ‘Large image gene +/− 15 Kb’ links to an image of our computational prediction of photoreceptor CREs in the genomic region 15 Kb upstream and downstream of the gene in question. ‘Closeup image TSS +/− 15 Kb’ links to an image of our computational prediction in the region 15 Kb on either side of the gene's TSS. Black bars within this image highlight the following regulatory peaks (if any are present): the peak closest to the TSS and the peak with the highest CRE score within this 30 Kb window. For those genes whose CRE predictions were tested experimentally, a red bar in this image indicates the location of the CRE that was tested. For Crx and Elovl4 the tested CRE is outside of this 30 Kb window and is therefore indicated in the ‘Large image’. ‘Links’ contains links to the UCSC genome browser, ENSEMBL, and NCBI database entries for the indicated gene, if available. ‘Max score (threshold of 200)’ contains the score of the highest predicted regulatory peak within the 30 Kb window around the gene's TSS. If this window does not contain any predicted peaks ≥ 200 (our cutoff threshold) no value is given (indicated by a dash). The next four columns of the table (‘Nr2e3-/-’ etc.) show the wild-type-to-mutant ratios of the averaged microarray scores for the given gene. For those genes to which more than one Affy tag correspond, the data for the first one listed under ‘Affy Mouse 430 2.0’ is given. Dark green = downregulated under high stringency (as described in METHODS); light green = downregulated under low stringency; red = upregulated under high stringency; orange = upregulated under low stringency. A dash indicates that the gene was not significantly a [file pone.0000643.s005.zip › Corbo_TableS1/images/A930015D22Rik.gif]

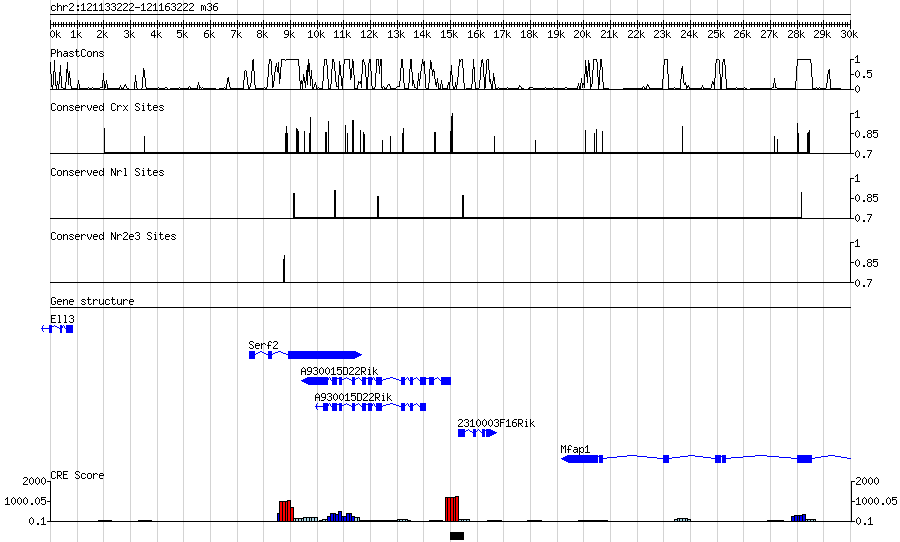

Supplement: Table S1 — Genes in the mouse photoreceptor transcription network. This table is a database which includes all genes dysregulated in Crx-/-, Nrl-/-, and/or Nr2e3-/- under high stringency criteria. Each row in the database represents a gene in the network and includes multiple links to additional types of information about that gene. ‘Large image gene +/− 15 Kb’ links to an image of our computational prediction of photoreceptor CREs in the genomic region 15 Kb upstream and downstream of the gene in question. ‘Closeup image TSS +/− 15 Kb’ links to an image of our computational prediction in the region 15 Kb on either side of the gene's TSS. Black bars within this image highlight the following regulatory peaks (if any are present): the peak closest to the TSS and the peak with the highest CRE score within this 30 Kb window. For those genes whose CRE predictions were tested experimentally, a red bar in this image indicates the location of the CRE that was tested. For Crx and Elovl4 the tested CRE is outside of this 30 Kb window and is therefore indicated in the ‘Large image’. ‘Links’ contains links to the UCSC genome browser, ENSEMBL, and NCBI database entries for the indicated gene, if available. ‘Max score (threshold of 200)’ contains the score of the highest predicted regulatory peak within the 30 Kb window around the gene's TSS. If this window does not contain any predicted peaks ≥ 200 (our cutoff threshold) no value is given (indicated by a dash). The next four columns of the table (‘Nr2e3-/-’ etc.) show the wild-type-to-mutant ratios of the averaged microarray scores for the given gene. For those genes to which more than one Affy tag correspond, the data for the first one listed under ‘Affy Mouse 430 2.0’ is given. Dark green = downregulated under high stringency (as described in METHODS); light green = downregulated under low stringency; red = upregulated under high stringency; orange = upregulated under low stringency. A dash indicates that the gene was not significantly a [file pone.0000643.s005.zip › Corbo_TableS1/images/A930015D22Rik_TSS.gif]

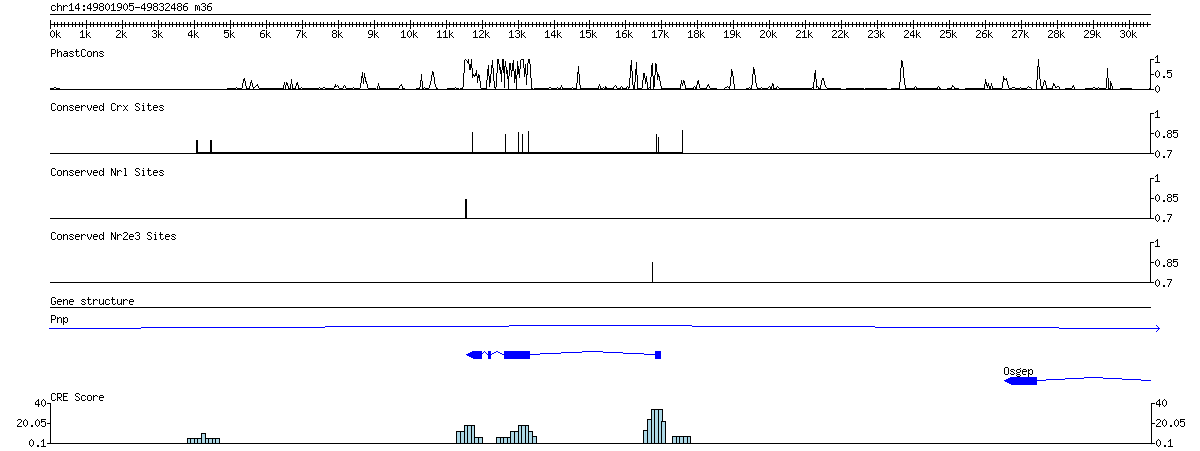

Supplement: Table S1 — Genes in the mouse photoreceptor transcription network. This table is a database which includes all genes dysregulated in Crx-/-, Nrl-/-, and/or Nr2e3-/- under high stringency criteria. Each row in the database represents a gene in the network and includes multiple links to additional types of information about that gene. ‘Large image gene +/− 15 Kb’ links to an image of our computational prediction of photoreceptor CREs in the genomic region 15 Kb upstream and downstream of the gene in question. ‘Closeup image TSS +/− 15 Kb’ links to an image of our computational prediction in the region 15 Kb on either side of the gene's TSS. Black bars within this image highlight the following regulatory peaks (if any are present): the peak closest to the TSS and the peak with the highest CRE score within this 30 Kb window. For those genes whose CRE predictions were tested experimentally, a red bar in this image indicates the location of the CRE that was tested. For Crx and Elovl4 the tested CRE is outside of this 30 Kb window and is therefore indicated in the ‘Large image’. ‘Links’ contains links to the UCSC genome browser, ENSEMBL, and NCBI database entries for the indicated gene, if available. ‘Max score (threshold of 200)’ contains the score of the highest predicted regulatory peak within the 30 Kb window around the gene's TSS. If this window does not contain any predicted peaks ≥ 200 (our cutoff threshold) no value is given (indicated by a dash). The next four columns of the table (‘Nr2e3-/-’ etc.) show the wild-type-to-mutant ratios of the averaged microarray scores for the given gene. For those genes to which more than one Affy tag correspond, the data for the first one listed under ‘Affy Mouse 430 2.0’ is given. Dark green = downregulated under high stringency (as described in METHODS); light green = downregulated under low stringency; red = upregulated under high stringency; orange = upregulated under low stringency. A dash indicates that the gene was not significantly a [file pone.0000643.s005.zip › Corbo_TableS1/images/A930018M24Rik.gif]

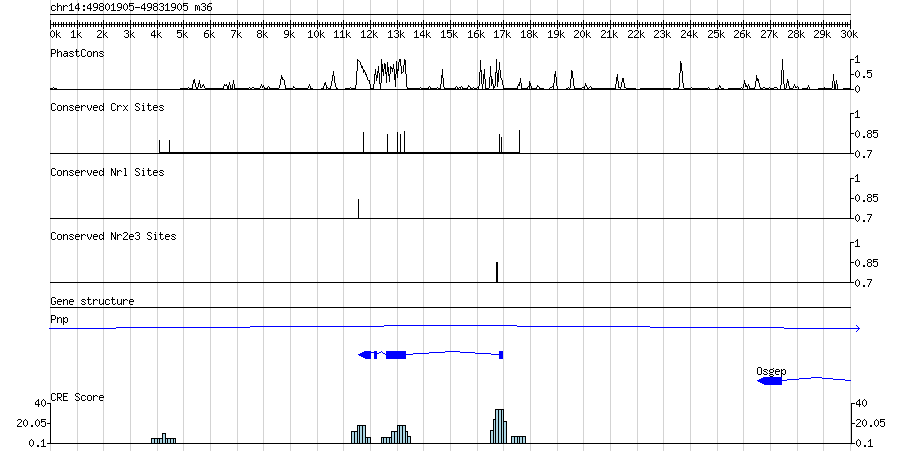

Supplement: Table S1 — Genes in the mouse photoreceptor transcription network. This table is a database which includes all genes dysregulated in Crx-/-, Nrl-/-, and/or Nr2e3-/- under high stringency criteria. Each row in the database represents a gene in the network and includes multiple links to additional types of information about that gene. ‘Large image gene +/− 15 Kb’ links to an image of our computational prediction of photoreceptor CREs in the genomic region 15 Kb upstream and downstream of the gene in question. ‘Closeup image TSS +/− 15 Kb’ links to an image of our computational prediction in the region 15 Kb on either side of the gene's TSS. Black bars within this image highlight the following regulatory peaks (if any are present): the peak closest to the TSS and the peak with the highest CRE score within this 30 Kb window. For those genes whose CRE predictions were tested experimentally, a red bar in this image indicates the location of the CRE that was tested. For Crx and Elovl4 the tested CRE is outside of this 30 Kb window and is therefore indicated in the ‘Large image’. ‘Links’ contains links to the UCSC genome browser, ENSEMBL, and NCBI database entries for the indicated gene, if available. ‘Max score (threshold of 200)’ contains the score of the highest predicted regulatory peak within the 30 Kb window around the gene's TSS. If this window does not contain any predicted peaks ≥ 200 (our cutoff threshold) no value is given (indicated by a dash). The next four columns of the table (‘Nr2e3-/-’ etc.) show the wild-type-to-mutant ratios of the averaged microarray scores for the given gene. For those genes to which more than one Affy tag correspond, the data for the first one listed under ‘Affy Mouse 430 2.0’ is given. Dark green = downregulated under high stringency (as described in METHODS); light green = downregulated under low stringency; red = upregulated under high stringency; orange = upregulated under low stringency. A dash indicates that the gene was not significantly a [file pone.0000643.s005.zip › Corbo_TableS1/images/A930018M24Rik_TSS.gif]

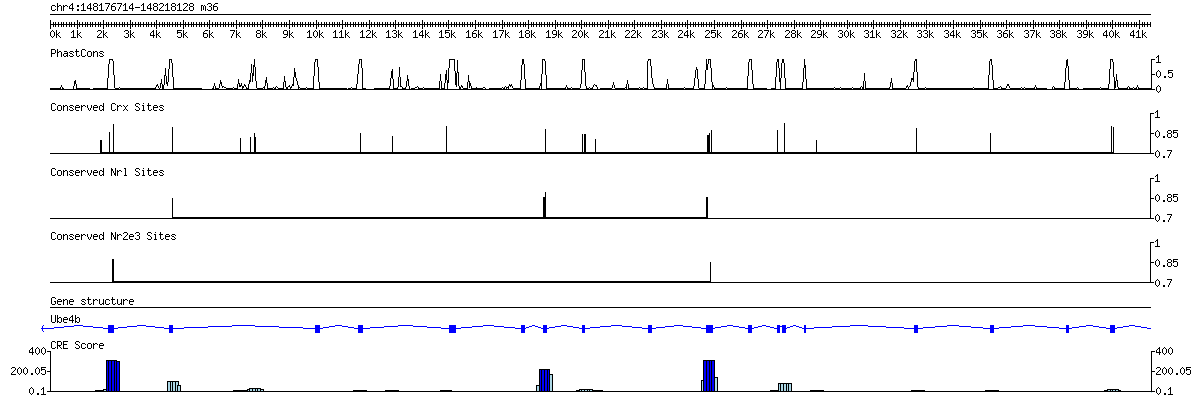

Supplement: Table S1 — Genes in the mouse photoreceptor transcription network. This table is a database which includes all genes dysregulated in Crx-/-, Nrl-/-, and/or Nr2e3-/- under high stringency criteria. Each row in the database represents a gene in the network and includes multiple links to additional types of information about that gene. ‘Large image gene +/− 15 Kb’ links to an image of our computational prediction of photoreceptor CREs in the genomic region 15 Kb upstream and downstream of the gene in question. ‘Closeup image TSS +/− 15 Kb’ links to an image of our computational prediction in the region 15 Kb on either side of the gene's TSS. Black bars within this image highlight the following regulatory peaks (if any are present): the peak closest to the TSS and the peak with the highest CRE score within this 30 Kb window. For those genes whose CRE predictions were tested experimentally, a red bar in this image indicates the location of the CRE that was tested. For Crx and Elovl4 the tested CRE is outside of this 30 Kb window and is therefore indicated in the ‘Large image’. ‘Links’ contains links to the UCSC genome browser, ENSEMBL, and NCBI database entries for the indicated gene, if available. ‘Max score (threshold of 200)’ contains the score of the highest predicted regulatory peak within the 30 Kb window around the gene's TSS. If this window does not contain any predicted peaks ≥ 200 (our cutoff threshold) no value is given (indicated by a dash). The next four columns of the table (‘Nr2e3-/-’ etc.) show the wild-type-to-mutant ratios of the averaged microarray scores for the given gene. For those genes to which more than one Affy tag correspond, the data for the first one listed under ‘Affy Mouse 430 2.0’ is given. Dark green = downregulated under high stringency (as described in METHODS); light green = downregulated under low stringency; red = upregulated under high stringency; orange = upregulated under low stringency. A dash indicates that the gene was not significantly a [file pone.0000643.s005.zip › Corbo_TableS1/images/A930028C08Rik.gif]

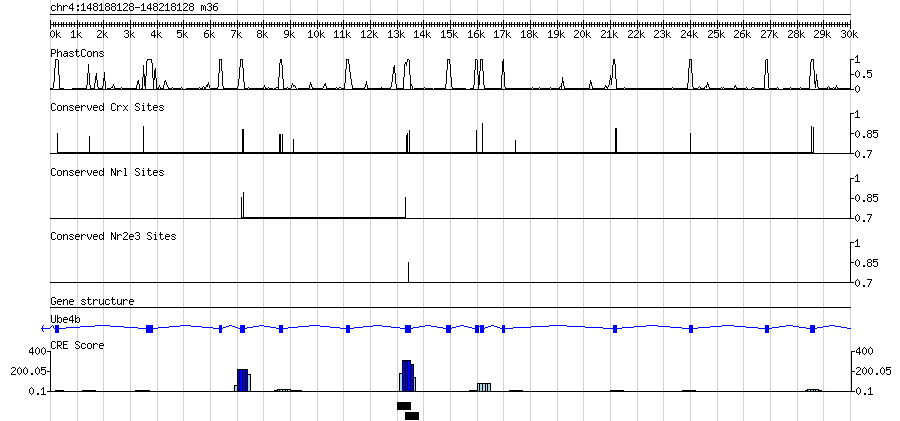

Supplement: Table S1 — Genes in the mouse photoreceptor transcription network. This table is a database which includes all genes dysregulated in Crx-/-, Nrl-/-, and/or Nr2e3-/- under high stringency criteria. Each row in the database represents a gene in the network and includes multiple links to additional types of information about that gene. ‘Large image gene +/− 15 Kb’ links to an image of our computational prediction of photoreceptor CREs in the genomic region 15 Kb upstream and downstream of the gene in question. ‘Closeup image TSS +/− 15 Kb’ links to an image of our computational prediction in the region 15 Kb on either side of the gene's TSS. Black bars within this image highlight the following regulatory peaks (if any are present): the peak closest to the TSS and the peak with the highest CRE score within this 30 Kb window. For those genes whose CRE predictions were tested experimentally, a red bar in this image indicates the location of the CRE that was tested. For Crx and Elovl4 the tested CRE is outside of this 30 Kb window and is therefore indicated in the ‘Large image’. ‘Links’ contains links to the UCSC genome browser, ENSEMBL, and NCBI database entries for the indicated gene, if available. ‘Max score (threshold of 200)’ contains the score of the highest predicted regulatory peak within the 30 Kb window around the gene's TSS. If this window does not contain any predicted peaks ≥ 200 (our cutoff threshold) no value is given (indicated by a dash). The next four columns of the table (‘Nr2e3-/-’ etc.) show the wild-type-to-mutant ratios of the averaged microarray scores for the given gene. For those genes to which more than one Affy tag correspond, the data for the first one listed under ‘Affy Mouse 430 2.0’ is given. Dark green = downregulated under high stringency (as described in METHODS); light green = downregulated under low stringency; red = upregulated under high stringency; orange = upregulated under low stringency. A dash indicates that the gene was not significantly a [file pone.0000643.s005.zip › Corbo_TableS1/images/A930028C08Rik_TSS.gif]

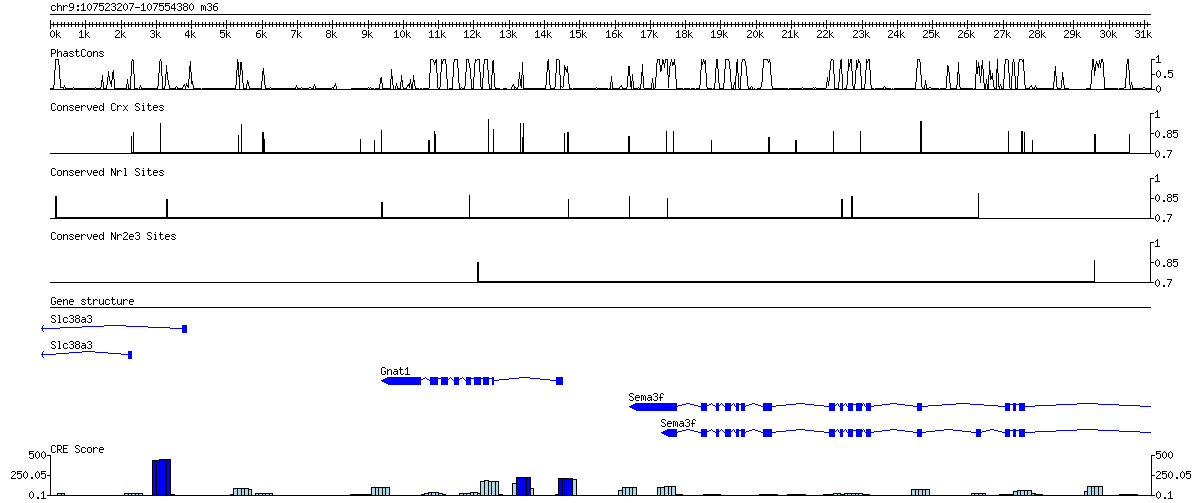

Supplement: Table S1 — Genes in the mouse photoreceptor transcription network. This table is a database which includes all genes dysregulated in Crx-/-, Nrl-/-, and/or Nr2e3-/- under high stringency criteria. Each row in the database represents a gene in the network and includes multiple links to additional types of information about that gene. ‘Large image gene +/− 15 Kb’ links to an image of our computational prediction of photoreceptor CREs in the genomic region 15 Kb upstream and downstream of the gene in question. ‘Closeup image TSS +/− 15 Kb’ links to an image of our computational prediction in the region 15 Kb on either side of the gene's TSS. Black bars within this image highlight the following regulatory peaks (if any are present): the peak closest to the TSS and the peak with the highest CRE score within this 30 Kb window. For those genes whose CRE predictions were tested experimentally, a red bar in this image indicates the location of the CRE that was tested. For Crx and Elovl4 the tested CRE is outside of this 30 Kb window and is therefore indicated in the ‘Large image’. ‘Links’ contains links to the UCSC genome browser, ENSEMBL, and NCBI database entries for the indicated gene, if available. ‘Max score (threshold of 200)’ contains the score of the highest predicted regulatory peak within the 30 Kb window around the gene's TSS. If this window does not contain any predicted peaks ≥ 200 (our cutoff threshold) no value is given (indicated by a dash). The next four columns of the table (‘Nr2e3-/-’ etc.) show the wild-type-to-mutant ratios of the averaged microarray scores for the given gene. For those genes to which more than one Affy tag correspond, the data for the first one listed under ‘Affy Mouse 430 2.0’ is given. Dark green = downregulated under high stringency (as described in METHODS); light green = downregulated under low stringency; red = upregulated under high stringency; orange = upregulated under low stringency. A dash indicates that the gene was not significantly a [file pone.0000643.s005.zip › Corbo_TableS1/images/A930036K24Rik.gif]

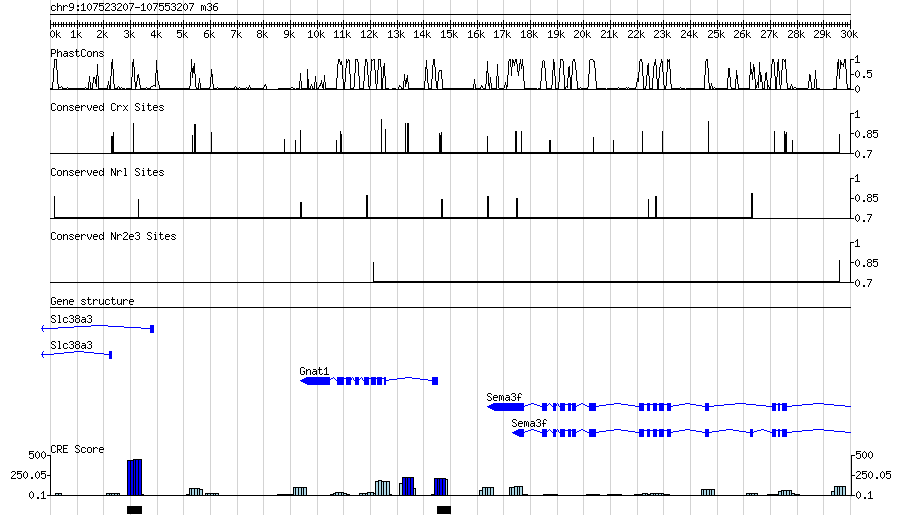

Supplement: Table S1 — Genes in the mouse photoreceptor transcription network. This table is a database which includes all genes dysregulated in Crx-/-, Nrl-/-, and/or Nr2e3-/- under high stringency criteria. Each row in the database represents a gene in the network and includes multiple links to additional types of information about that gene. ‘Large image gene +/− 15 Kb’ links to an image of our computational prediction of photoreceptor CREs in the genomic region 15 Kb upstream and downstream of the gene in question. ‘Closeup image TSS +/− 15 Kb’ links to an image of our computational prediction in the region 15 Kb on either side of the gene's TSS. Black bars within this image highlight the following regulatory peaks (if any are present): the peak closest to the TSS and the peak with the highest CRE score within this 30 Kb window. For those genes whose CRE predictions were tested experimentally, a red bar in this image indicates the location of the CRE that was tested. For Crx and Elovl4 the tested CRE is outside of this 30 Kb window and is therefore indicated in the ‘Large image’. ‘Links’ contains links to the UCSC genome browser, ENSEMBL, and NCBI database entries for the indicated gene, if available. ‘Max score (threshold of 200)’ contains the score of the highest predicted regulatory peak within the 30 Kb window around the gene's TSS. If this window does not contain any predicted peaks ≥ 200 (our cutoff threshold) no value is given (indicated by a dash). The next four columns of the table (‘Nr2e3-/-’ etc.) show the wild-type-to-mutant ratios of the averaged microarray scores for the given gene. For those genes to which more than one Affy tag correspond, the data for the first one listed under ‘Affy Mouse 430 2.0’ is given. Dark green = downregulated under high stringency (as described in METHODS); light green = downregulated under low stringency; red = upregulated under high stringency; orange = upregulated under low stringency. A dash indicates that the gene was not significantly a [file pone.0000643.s005.zip › Corbo_TableS1/images/A930036K24Rik_TSS.gif]

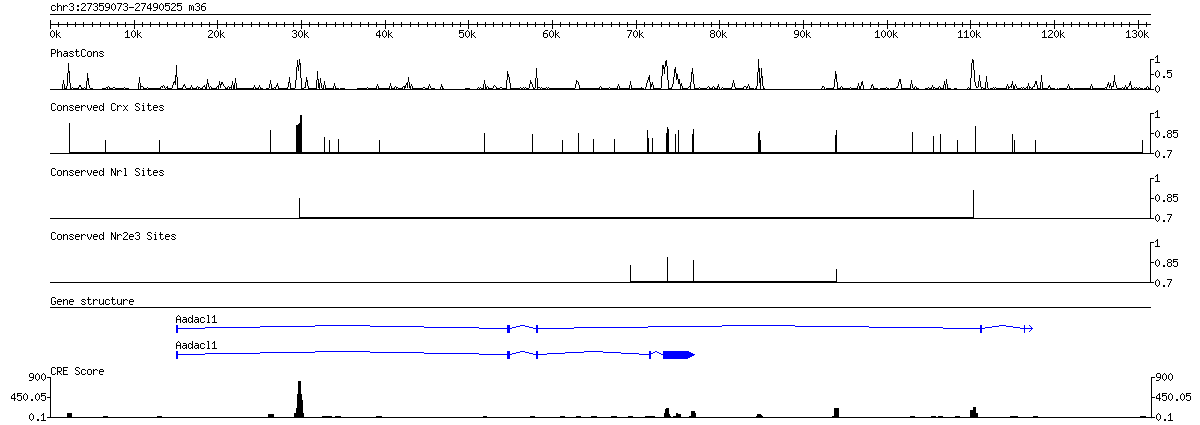

Supplement: Table S1 — Genes in the mouse photoreceptor transcription network. This table is a database which includes all genes dysregulated in Crx-/-, Nrl-/-, and/or Nr2e3-/- under high stringency criteria. Each row in the database represents a gene in the network and includes multiple links to additional types of information about that gene. ‘Large image gene +/− 15 Kb’ links to an image of our computational prediction of photoreceptor CREs in the genomic region 15 Kb upstream and downstream of the gene in question. ‘Closeup image TSS +/− 15 Kb’ links to an image of our computational prediction in the region 15 Kb on either side of the gene's TSS. Black bars within this image highlight the following regulatory peaks (if any are present): the peak closest to the TSS and the peak with the highest CRE score within this 30 Kb window. For those genes whose CRE predictions were tested experimentally, a red bar in this image indicates the location of the CRE that was tested. For Crx and Elovl4 the tested CRE is outside of this 30 Kb window and is therefore indicated in the ‘Large image’. ‘Links’ contains links to the UCSC genome browser, ENSEMBL, and NCBI database entries for the indicated gene, if available. ‘Max score (threshold of 200)’ contains the score of the highest predicted regulatory peak within the 30 Kb window around the gene's TSS. If this window does not contain any predicted peaks ≥ 200 (our cutoff threshold) no value is given (indicated by a dash). The next four columns of the table (‘Nr2e3-/-’ etc.) show the wild-type-to-mutant ratios of the averaged microarray scores for the given gene. For those genes to which more than one Affy tag correspond, the data for the first one listed under ‘Affy Mouse 430 2.0’ is given. Dark green = downregulated under high stringency (as described in METHODS); light green = downregulated under low stringency; red = upregulated under high stringency; orange = upregulated under low stringency. A dash indicates that the gene was not significantly a [file pone.0000643.s005.zip › Corbo_TableS1/images/Aadacl1.gif]

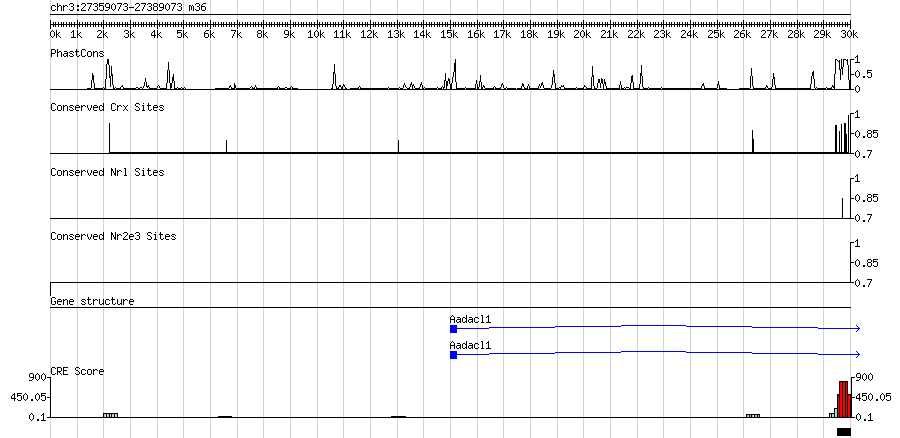

Supplement: Table S1 — Genes in the mouse photoreceptor transcription network. This table is a database which includes all genes dysregulated in Crx-/-, Nrl-/-, and/or Nr2e3-/- under high stringency criteria. Each row in the database represents a gene in the network and includes multiple links to additional types of information about that gene. ‘Large image gene +/− 15 Kb’ links to an image of our computational prediction of photoreceptor CREs in the genomic region 15 Kb upstream and downstream of the gene in question. ‘Closeup image TSS +/− 15 Kb’ links to an image of our computational prediction in the region 15 Kb on either side of the gene's TSS. Black bars within this image highlight the following regulatory peaks (if any are present): the peak closest to the TSS and the peak with the highest CRE score within this 30 Kb window. For those genes whose CRE predictions were tested experimentally, a red bar in this image indicates the location of the CRE that was tested. For Crx and Elovl4 the tested CRE is outside of this 30 Kb window and is therefore indicated in the ‘Large image’. ‘Links’ contains links to the UCSC genome browser, ENSEMBL, and NCBI database entries for the indicated gene, if available. ‘Max score (threshold of 200)’ contains the score of the highest predicted regulatory peak within the 30 Kb window around the gene's TSS. If this window does not contain any predicted peaks ≥ 200 (our cutoff threshold) no value is given (indicated by a dash). The next four columns of the table (‘Nr2e3-/-’ etc.) show the wild-type-to-mutant ratios of the averaged microarray scores for the given gene. For those genes to which more than one Affy tag correspond, the data for the first one listed under ‘Affy Mouse 430 2.0’ is given. Dark green = downregulated under high stringency (as described in METHODS); light green = downregulated under low stringency; red = upregulated under high stringency; orange = upregulated under low stringency. A dash indicates that the gene was not significantly a [file pone.0000643.s005.zip › Corbo_TableS1/images/Aadacl1_TSS.gif]

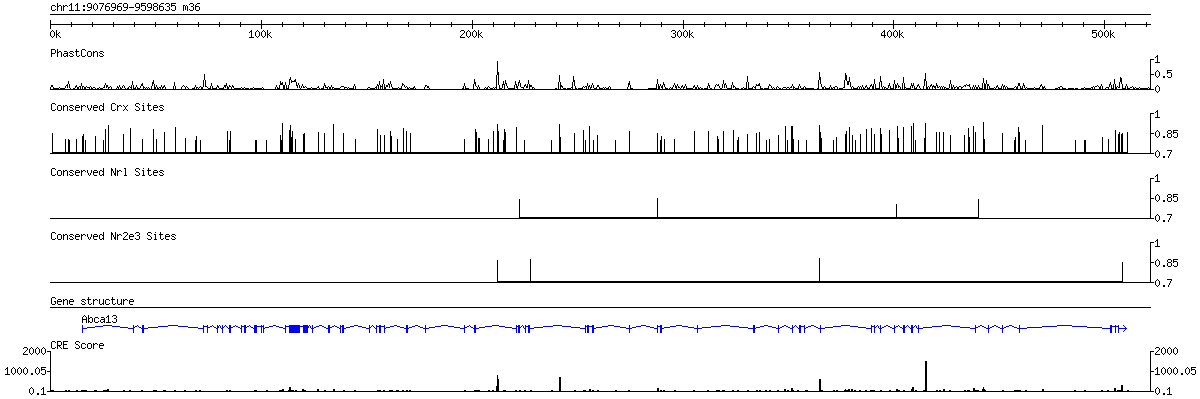

Supplement: Table S1 — Genes in the mouse photoreceptor transcription network. This table is a database which includes all genes dysregulated in Crx-/-, Nrl-/-, and/or Nr2e3-/- under high stringency criteria. Each row in the database represents a gene in the network and includes multiple links to additional types of information about that gene. ‘Large image gene +/− 15 Kb’ links to an image of our computational prediction of photoreceptor CREs in the genomic region 15 Kb upstream and downstream of the gene in question. ‘Closeup image TSS +/− 15 Kb’ links to an image of our computational prediction in the region 15 Kb on either side of the gene's TSS. Black bars within this image highlight the following regulatory peaks (if any are present): the peak closest to the TSS and the peak with the highest CRE score within this 30 Kb window. For those genes whose CRE predictions were tested experimentally, a red bar in this image indicates the location of the CRE that was tested. For Crx and Elovl4 the tested CRE is outside of this 30 Kb window and is therefore indicated in the ‘Large image’. ‘Links’ contains links to the UCSC genome browser, ENSEMBL, and NCBI database entries for the indicated gene, if available. ‘Max score (threshold of 200)’ contains the score of the highest predicted regulatory peak within the 30 Kb window around the gene's TSS. If this window does not contain any predicted peaks ≥ 200 (our cutoff threshold) no value is given (indicated by a dash). The next four columns of the table (‘Nr2e3-/-’ etc.) show the wild-type-to-mutant ratios of the averaged microarray scores for the given gene. For those genes to which more than one Affy tag correspond, the data for the first one listed under ‘Affy Mouse 430 2.0’ is given. Dark green = downregulated under high stringency (as described in METHODS); light green = downregulated under low stringency; red = upregulated under high stringency; orange = upregulated under low stringency. A dash indicates that the gene was not significantly a [file pone.0000643.s005.zip › Corbo_TableS1/images/Abca13.gif]

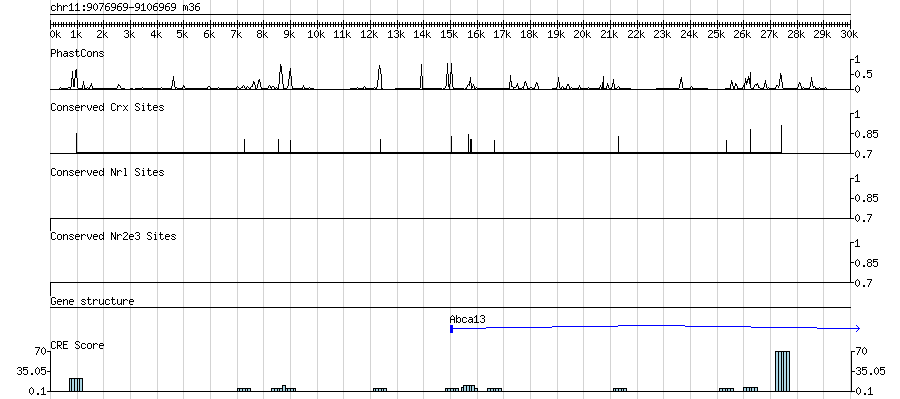

Supplement: Table S1 — Genes in the mouse photoreceptor transcription network. This table is a database which includes all genes dysregulated in Crx-/-, Nrl-/-, and/or Nr2e3-/- under high stringency criteria. Each row in the database represents a gene in the network and includes multiple links to additional types of information about that gene. ‘Large image gene +/− 15 Kb’ links to an image of our computational prediction of photoreceptor CREs in the genomic region 15 Kb upstream and downstream of the gene in question. ‘Closeup image TSS +/− 15 Kb’ links to an image of our computational prediction in the region 15 Kb on either side of the gene's TSS. Black bars within this image highlight the following regulatory peaks (if any are present): the peak closest to the TSS and the peak with the highest CRE score within this 30 Kb window. For those genes whose CRE predictions were tested experimentally, a red bar in this image indicates the location of the CRE that was tested. For Crx and Elovl4 the tested CRE is outside of this 30 Kb window and is therefore indicated in the ‘Large image’. ‘Links’ contains links to the UCSC genome browser, ENSEMBL, and NCBI database entries for the indicated gene, if available. ‘Max score (threshold of 200)’ contains the score of the highest predicted regulatory peak within the 30 Kb window around the gene's TSS. If this window does not contain any predicted peaks ≥ 200 (our cutoff threshold) no value is given (indicated by a dash). The next four columns of the table (‘Nr2e3-/-’ etc.) show the wild-type-to-mutant ratios of the averaged microarray scores for the given gene. For those genes to which more than one Affy tag correspond, the data for the first one listed under ‘Affy Mouse 430 2.0’ is given. Dark green = downregulated under high stringency (as described in METHODS); light green = downregulated under low stringency; red = upregulated under high stringency; orange = upregulated under low stringency. A dash indicates that the gene was not significantly a [file pone.0000643.s005.zip › Corbo_TableS1/images/Abca13_TSS.gif]

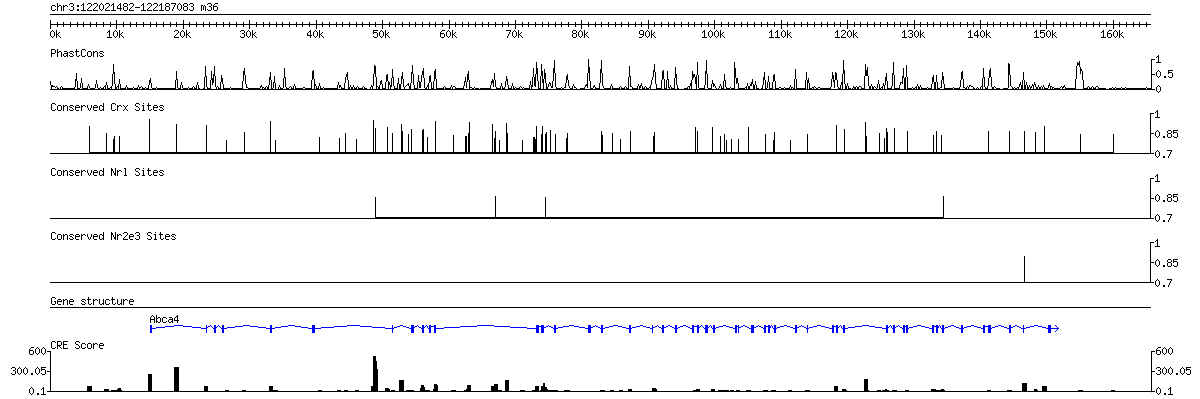

Supplement: Table S1 — Genes in the mouse photoreceptor transcription network. This table is a database which includes all genes dysregulated in Crx-/-, Nrl-/-, and/or Nr2e3-/- under high stringency criteria. Each row in the database represents a gene in the network and includes multiple links to additional types of information about that gene. ‘Large image gene +/− 15 Kb’ links to an image of our computational prediction of photoreceptor CREs in the genomic region 15 Kb upstream and downstream of the gene in question. ‘Closeup image TSS +/− 15 Kb’ links to an image of our computational prediction in the region 15 Kb on either side of the gene's TSS. Black bars within this image highlight the following regulatory peaks (if any are present): the peak closest to the TSS and the peak with the highest CRE score within this 30 Kb window. For those genes whose CRE predictions were tested experimentally, a red bar in this image indicates the location of the CRE that was tested. For Crx and Elovl4 the tested CRE is outside of this 30 Kb window and is therefore indicated in the ‘Large image’. ‘Links’ contains links to the UCSC genome browser, ENSEMBL, and NCBI database entries for the indicated gene, if available. ‘Max score (threshold of 200)’ contains the score of the highest predicted regulatory peak within the 30 Kb window around the gene's TSS. If this window does not contain any predicted peaks ≥ 200 (our cutoff threshold) no value is given (indicated by a dash). The next four columns of the table (‘Nr2e3-/-’ etc.) show the wild-type-to-mutant ratios of the averaged microarray scores for the given gene. For those genes to which more than one Affy tag correspond, the data for the first one listed under ‘Affy Mouse 430 2.0’ is given. Dark green = downregulated under high stringency (as described in METHODS); light green = downregulated under low stringency; red = upregulated under high stringency; orange = upregulated under low stringency. A dash indicates that the gene was not significantly a [file pone.0000643.s005.zip › Corbo_TableS1/images/Abca4.gif]

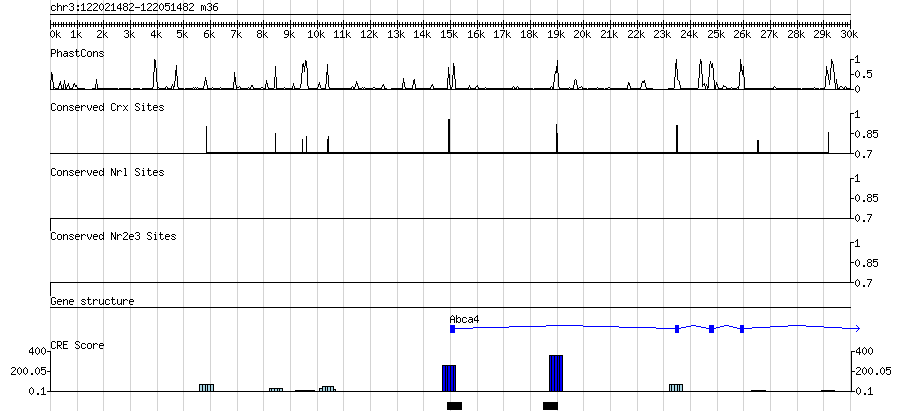

Supplement: Table S1 — Genes in the mouse photoreceptor transcription network. This table is a database which includes all genes dysregulated in Crx-/-, Nrl-/-, and/or Nr2e3-/- under high stringency criteria. Each row in the database represents a gene in the network and includes multiple links to additional types of information about that gene. ‘Large image gene +/− 15 Kb’ links to an image of our computational prediction of photoreceptor CREs in the genomic region 15 Kb upstream and downstream of the gene in question. ‘Closeup image TSS +/− 15 Kb’ links to an image of our computational prediction in the region 15 Kb on either side of the gene's TSS. Black bars within this image highlight the following regulatory peaks (if any are present): the peak closest to the TSS and the peak with the highest CRE score within this 30 Kb window. For those genes whose CRE predictions were tested experimentally, a red bar in this image indicates the location of the CRE that was tested. For Crx and Elovl4 the tested CRE is outside of this 30 Kb window and is therefore indicated in the ‘Large image’. ‘Links’ contains links to the UCSC genome browser, ENSEMBL, and NCBI database entries for the indicated gene, if available. ‘Max score (threshold of 200)’ contains the score of the highest predicted regulatory peak within the 30 Kb window around the gene's TSS. If this window does not contain any predicted peaks ≥ 200 (our cutoff threshold) no value is given (indicated by a dash). The next four columns of the table (‘Nr2e3-/-’ etc.) show the wild-type-to-mutant ratios of the averaged microarray scores for the given gene. For those genes to which more than one Affy tag correspond, the data for the first one listed under ‘Affy Mouse 430 2.0’ is given. Dark green = downregulated under high stringency (as described in METHODS); light green = downregulated under low stringency; red = upregulated under high stringency; orange = upregulated under low stringency. A dash indicates that the gene was not significantly a [file pone.0000643.s005.zip › Corbo_TableS1/images/Abca4_TSS.gif]

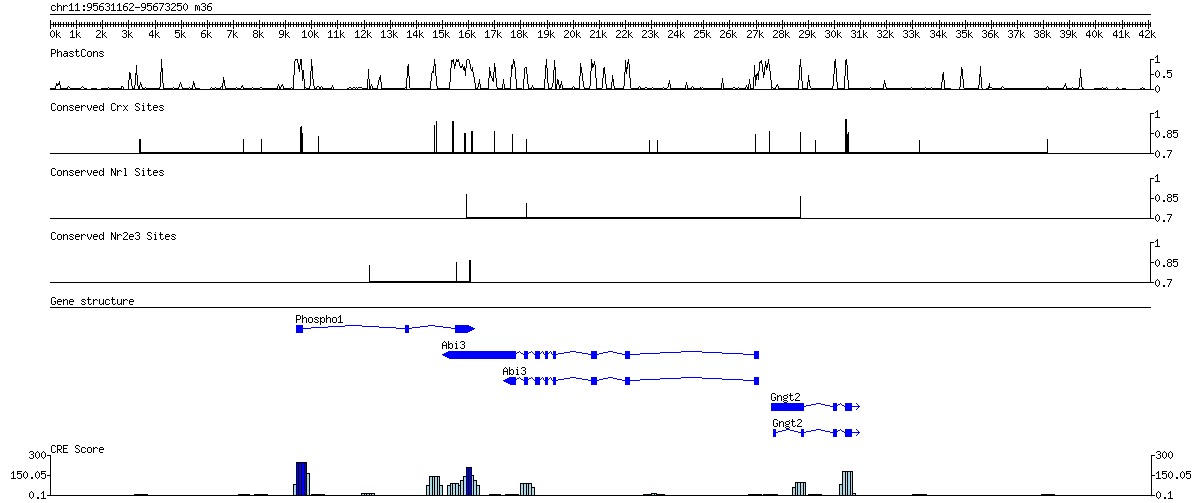

Supplement: Table S1 — Genes in the mouse photoreceptor transcription network. This table is a database which includes all genes dysregulated in Crx-/-, Nrl-/-, and/or Nr2e3-/- under high stringency criteria. Each row in the database represents a gene in the network and includes multiple links to additional types of information about that gene. ‘Large image gene +/− 15 Kb’ links to an image of our computational prediction of photoreceptor CREs in the genomic region 15 Kb upstream and downstream of the gene in question. ‘Closeup image TSS +/− 15 Kb’ links to an image of our computational prediction in the region 15 Kb on either side of the gene's TSS. Black bars within this image highlight the following regulatory peaks (if any are present): the peak closest to the TSS and the peak with the highest CRE score within this 30 Kb window. For those genes whose CRE predictions were tested experimentally, a red bar in this image indicates the location of the CRE that was tested. For Crx and Elovl4 the tested CRE is outside of this 30 Kb window and is therefore indicated in the ‘Large image’. ‘Links’ contains links to the UCSC genome browser, ENSEMBL, and NCBI database entries for the indicated gene, if available. ‘Max score (threshold of 200)’ contains the score of the highest predicted regulatory peak within the 30 Kb window around the gene's TSS. If this window does not contain any predicted peaks ≥ 200 (our cutoff threshold) no value is given (indicated by a dash). The next four columns of the table (‘Nr2e3-/-’ etc.) show the wild-type-to-mutant ratios of the averaged microarray scores for the given gene. For those genes to which more than one Affy tag correspond, the data for the first one listed under ‘Affy Mouse 430 2.0’ is given. Dark green = downregulated under high stringency (as described in METHODS); light green = downregulated under low stringency; red = upregulated under high stringency; orange = upregulated under low stringency. A dash indicates that the gene was not significantly a [file pone.0000643.s005.zip › Corbo_TableS1/images/Abi3.gif]

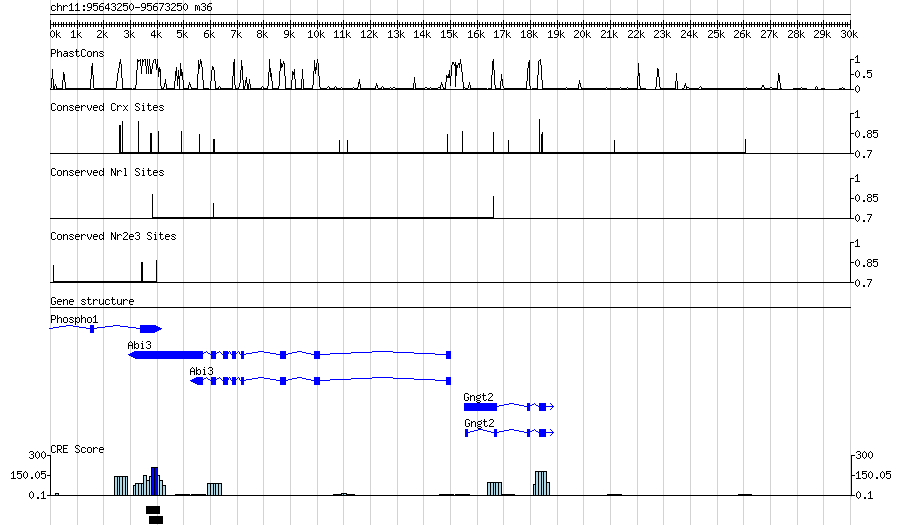

Supplement: Table S1 — Genes in the mouse photoreceptor transcription network. This table is a database which includes all genes dysregulated in Crx-/-, Nrl-/-, and/or Nr2e3-/- under high stringency criteria. Each row in the database represents a gene in the network and includes multiple links to additional types of information about that gene. ‘Large image gene +/− 15 Kb’ links to an image of our computational prediction of photoreceptor CREs in the genomic region 15 Kb upstream and downstream of the gene in question. ‘Closeup image TSS +/− 15 Kb’ links to an image of our computational prediction in the region 15 Kb on either side of the gene's TSS. Black bars within this image highlight the following regulatory peaks (if any are present): the peak closest to the TSS and the peak with the highest CRE score within this 30 Kb window. For those genes whose CRE predictions were tested experimentally, a red bar in this image indicates the location of the CRE that was tested. For Crx and Elovl4 the tested CRE is outside of this 30 Kb window and is therefore indicated in the ‘Large image’. ‘Links’ contains links to the UCSC genome browser, ENSEMBL, and NCBI database entries for the indicated gene, if available. ‘Max score (threshold of 200)’ contains the score of the highest predicted regulatory peak within the 30 Kb window around the gene's TSS. If this window does not contain any predicted peaks ≥ 200 (our cutoff threshold) no value is given (indicated by a dash). The next four columns of the table (‘Nr2e3-/-’ etc.) show the wild-type-to-mutant ratios of the averaged microarray scores for the given gene. For those genes to which more than one Affy tag correspond, the data for the first one listed under ‘Affy Mouse 430 2.0’ is given. Dark green = downregulated under high stringency (as described in METHODS); light green = downregulated under low stringency; red = upregulated under high stringency; orange = upregulated under low stringency. A dash indicates that the gene was not significantly a [file pone.0000643.s005.zip › Corbo_TableS1/images/Abi3_TSS.gif]

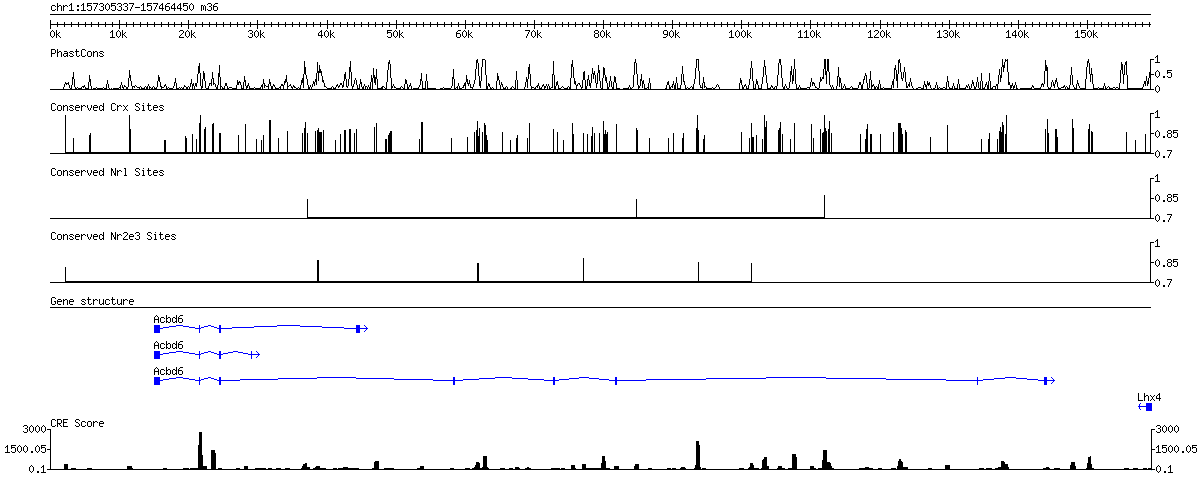

Supplement: Table S1 — Genes in the mouse photoreceptor transcription network. This table is a database which includes all genes dysregulated in Crx-/-, Nrl-/-, and/or Nr2e3-/- under high stringency criteria. Each row in the database represents a gene in the network and includes multiple links to additional types of information about that gene. ‘Large image gene +/− 15 Kb’ links to an image of our computational prediction of photoreceptor CREs in the genomic region 15 Kb upstream and downstream of the gene in question. ‘Closeup image TSS +/− 15 Kb’ links to an image of our computational prediction in the region 15 Kb on either side of the gene's TSS. Black bars within this image highlight the following regulatory peaks (if any are present): the peak closest to the TSS and the peak with the highest CRE score within this 30 Kb window. For those genes whose CRE predictions were tested experimentally, a red bar in this image indicates the location of the CRE that was tested. For Crx and Elovl4 the tested CRE is outside of this 30 Kb window and is therefore indicated in the ‘Large image’. ‘Links’ contains links to the UCSC genome browser, ENSEMBL, and NCBI database entries for the indicated gene, if available. ‘Max score (threshold of 200)’ contains the score of the highest predicted regulatory peak within the 30 Kb window around the gene's TSS. If this window does not contain any predicted peaks ≥ 200 (our cutoff threshold) no value is given (indicated by a dash). The next four columns of the table (‘Nr2e3-/-’ etc.) show the wild-type-to-mutant ratios of the averaged microarray scores for the given gene. For those genes to which more than one Affy tag correspond, the data for the first one listed under ‘Affy Mouse 430 2.0’ is given. Dark green = downregulated under high stringency (as described in METHODS); light green = downregulated under low stringency; red = upregulated under high stringency; orange = upregulated under low stringency. A dash indicates that the gene was not significantly a [file pone.0000643.s005.zip › Corbo_TableS1/images/Acbd6.gif]

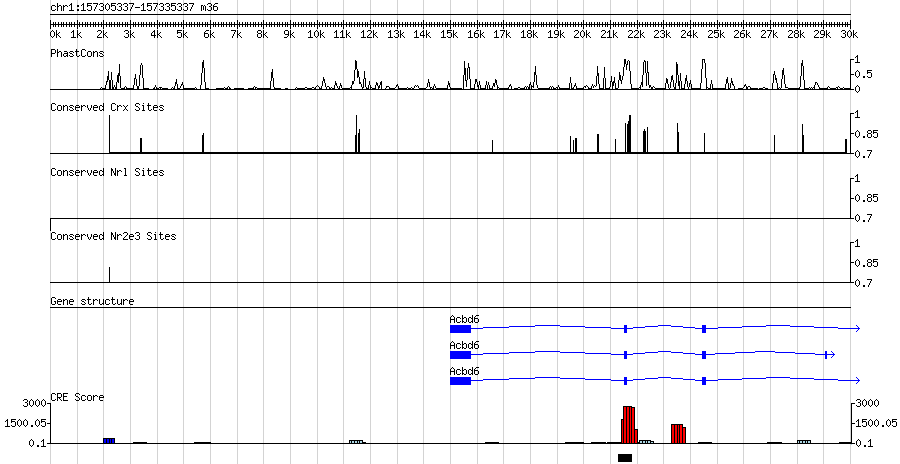

Supplement: Table S1 — Genes in the mouse photoreceptor transcription network. This table is a database which includes all genes dysregulated in Crx-/-, Nrl-/-, and/or Nr2e3-/- under high stringency criteria. Each row in the database represents a gene in the network and includes multiple links to additional types of information about that gene. ‘Large image gene +/− 15 Kb’ links to an image of our computational prediction of photoreceptor CREs in the genomic region 15 Kb upstream and downstream of the gene in question. ‘Closeup image TSS +/− 15 Kb’ links to an image of our computational prediction in the region 15 Kb on either side of the gene's TSS. Black bars within this image highlight the following regulatory peaks (if any are present): the peak closest to the TSS and the peak with the highest CRE score within this 30 Kb window. For those genes whose CRE predictions were tested experimentally, a red bar in this image indicates the location of the CRE that was tested. For Crx and Elovl4 the tested CRE is outside of this 30 Kb window and is therefore indicated in the ‘Large image’. ‘Links’ contains links to the UCSC genome browser, ENSEMBL, and NCBI database entries for the indicated gene, if available. ‘Max score (threshold of 200)’ contains the score of the highest predicted regulatory peak within the 30 Kb window around the gene's TSS. If this window does not contain any predicted peaks ≥ 200 (our cutoff threshold) no value is given (indicated by a dash). The next four columns of the table (‘Nr2e3-/-’ etc.) show the wild-type-to-mutant ratios of the averaged microarray scores for the given gene. For those genes to which more than one Affy tag correspond, the data for the first one listed under ‘Affy Mouse 430 2.0’ is given. Dark green = downregulated under high stringency (as described in METHODS); light green = downregulated under low stringency; red = upregulated under high stringency; orange = upregulated under low stringency. A dash indicates that the gene was not significantly a [file pone.0000643.s005.zip › Corbo_TableS1/images/Acbd6_TSS.gif]

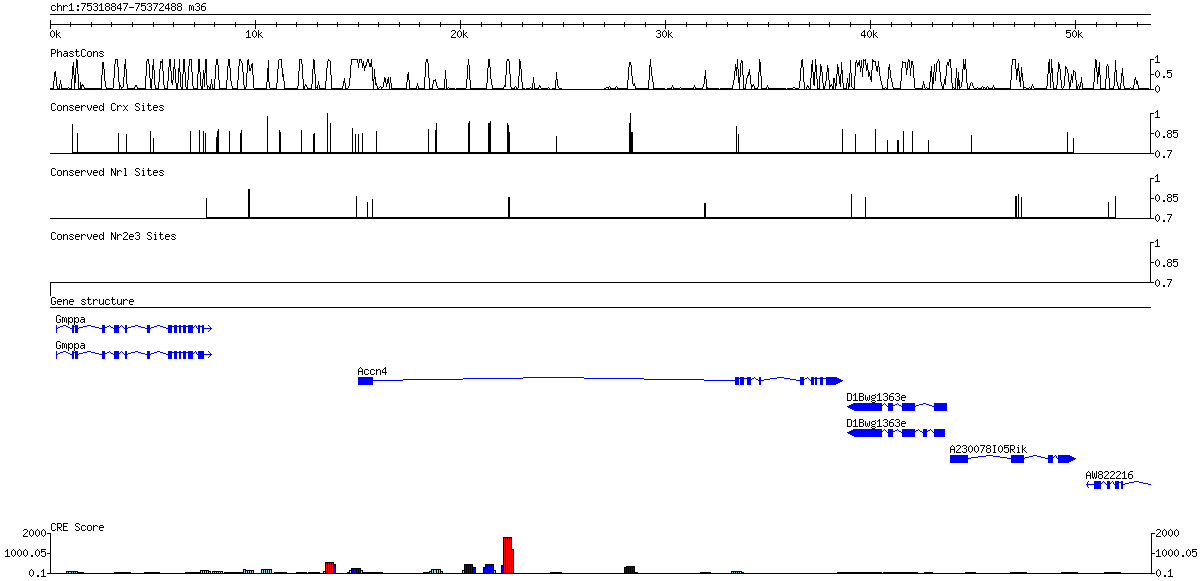

Supplement: Table S1 — Genes in the mouse photoreceptor transcription network. This table is a database which includes all genes dysregulated in Crx-/-, Nrl-/-, and/or Nr2e3-/- under high stringency criteria. Each row in the database represents a gene in the network and includes multiple links to additional types of information about that gene. ‘Large image gene +/− 15 Kb’ links to an image of our computational prediction of photoreceptor CREs in the genomic region 15 Kb upstream and downstream of the gene in question. ‘Closeup image TSS +/− 15 Kb’ links to an image of our computational prediction in the region 15 Kb on either side of the gene's TSS. Black bars within this image highlight the following regulatory peaks (if any are present): the peak closest to the TSS and the peak with the highest CRE score within this 30 Kb window. For those genes whose CRE predictions were tested experimentally, a red bar in this image indicates the location of the CRE that was tested. For Crx and Elovl4 the tested CRE is outside of this 30 Kb window and is therefore indicated in the ‘Large image’. ‘Links’ contains links to the UCSC genome browser, ENSEMBL, and NCBI database entries for the indicated gene, if available. ‘Max score (threshold of 200)’ contains the score of the highest predicted regulatory peak within the 30 Kb window around the gene's TSS. If this window does not contain any predicted peaks ≥ 200 (our cutoff threshold) no value is given (indicated by a dash). The next four columns of the table (‘Nr2e3-/-’ etc.) show the wild-type-to-mutant ratios of the averaged microarray scores for the given gene. For those genes to which more than one Affy tag correspond, the data for the first one listed under ‘Affy Mouse 430 2.0’ is given. Dark green = downregulated under high stringency (as described in METHODS); light green = downregulated under low stringency; red = upregulated under high stringency; orange = upregulated under low stringency. A dash indicates that the gene was not significantly a [file pone.0000643.s005.zip › Corbo_TableS1/images/Accn4.gif]

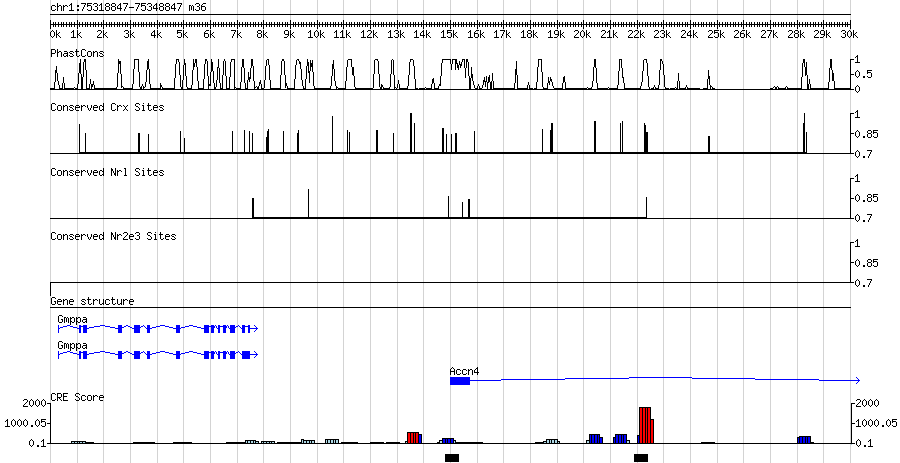

Supplement: Table S1 — Genes in the mouse photoreceptor transcription network. This table is a database which includes all genes dysregulated in Crx-/-, Nrl-/-, and/or Nr2e3-/- under high stringency criteria. Each row in the database represents a gene in the network and includes multiple links to additional types of information about that gene. ‘Large image gene +/− 15 Kb’ links to an image of our computational prediction of photoreceptor CREs in the genomic region 15 Kb upstream and downstream of the gene in question. ‘Closeup image TSS +/− 15 Kb’ links to an image of our computational prediction in the region 15 Kb on either side of the gene's TSS. Black bars within this image highlight the following regulatory peaks (if any are present): the peak closest to the TSS and the peak with the highest CRE score within this 30 Kb window. For those genes whose CRE predictions were tested experimentally, a red bar in this image indicates the location of the CRE that was tested. For Crx and Elovl4 the tested CRE is outside of this 30 Kb window and is therefore indicated in the ‘Large image’. ‘Links’ contains links to the UCSC genome browser, ENSEMBL, and NCBI database entries for the indicated gene, if available. ‘Max score (threshold of 200)’ contains the score of the highest predicted regulatory peak within the 30 Kb window around the gene's TSS. If this window does not contain any predicted peaks ≥ 200 (our cutoff threshold) no value is given (indicated by a dash). The next four columns of the table (‘Nr2e3-/-’ etc.) show the wild-type-to-mutant ratios of the averaged microarray scores for the given gene. For those genes to which more than one Affy tag correspond, the data for the first one listed under ‘Affy Mouse 430 2.0’ is given. Dark green = downregulated under high stringency (as described in METHODS); light green = downregulated under low stringency; red = upregulated under high stringency; orange = upregulated under low stringency. A dash indicates that the gene was not significantly a [file pone.0000643.s005.zip › Corbo_TableS1/images/Accn4_TSS.gif]

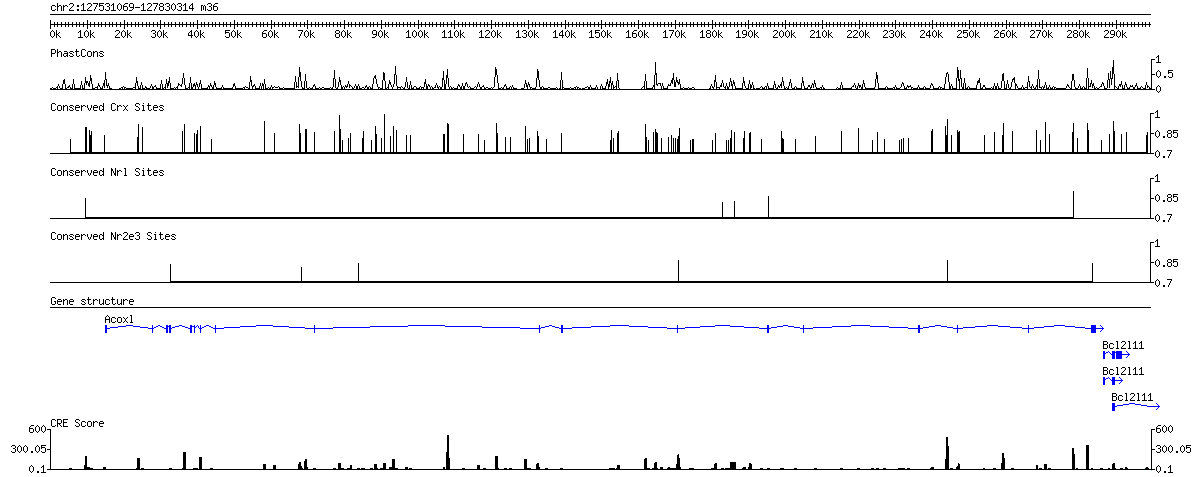

Supplement: Table S1 — Genes in the mouse photoreceptor transcription network. This table is a database which includes all genes dysregulated in Crx-/-, Nrl-/-, and/or Nr2e3-/- under high stringency criteria. Each row in the database represents a gene in the network and includes multiple links to additional types of information about that gene. ‘Large image gene +/− 15 Kb’ links to an image of our computational prediction of photoreceptor CREs in the genomic region 15 Kb upstream and downstream of the gene in question. ‘Closeup image TSS +/− 15 Kb’ links to an image of our computational prediction in the region 15 Kb on either side of the gene's TSS. Black bars within this image highlight the following regulatory peaks (if any are present): the peak closest to the TSS and the peak with the highest CRE score within this 30 Kb window. For those genes whose CRE predictions were tested experimentally, a red bar in this image indicates the location of the CRE that was tested. For Crx and Elovl4 the tested CRE is outside of this 30 Kb window and is therefore indicated in the ‘Large image’. ‘Links’ contains links to the UCSC genome browser, ENSEMBL, and NCBI database entries for the indicated gene, if available. ‘Max score (threshold of 200)’ contains the score of the highest predicted regulatory peak within the 30 Kb window around the gene's TSS. If this window does not contain any predicted peaks ≥ 200 (our cutoff threshold) no value is given (indicated by a dash). The next four columns of the table (‘Nr2e3-/-’ etc.) show the wild-type-to-mutant ratios of the averaged microarray scores for the given gene. For those genes to which more than one Affy tag correspond, the data for the first one listed under ‘Affy Mouse 430 2.0’ is given. Dark green = downregulated under high stringency (as described in METHODS); light green = downregulated under low stringency; red = upregulated under high stringency; orange = upregulated under low stringency. A dash indicates that the gene was not significantly a [file pone.0000643.s005.zip › Corbo_TableS1/images/Acoxl.gif]

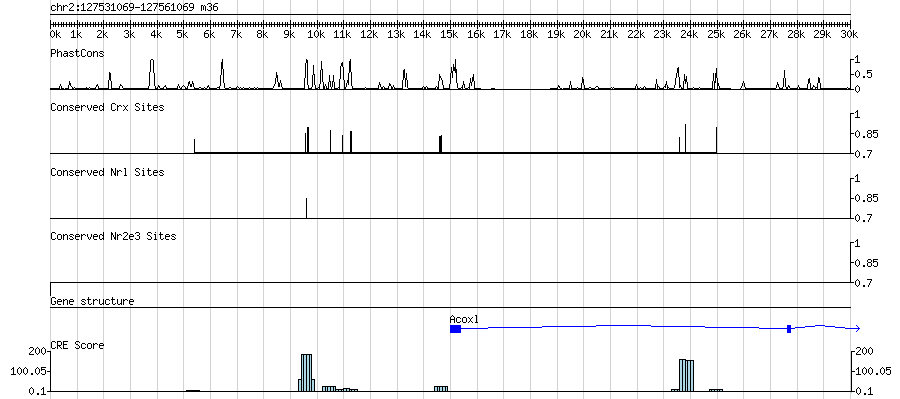

Supplement: Table S1 — Genes in the mouse photoreceptor transcription network. This table is a database which includes all genes dysregulated in Crx-/-, Nrl-/-, and/or Nr2e3-/- under high stringency criteria. Each row in the database represents a gene in the network and includes multiple links to additional types of information about that gene. ‘Large image gene +/− 15 Kb’ links to an image of our computational prediction of photoreceptor CREs in the genomic region 15 Kb upstream and downstream of the gene in question. ‘Closeup image TSS +/− 15 Kb’ links to an image of our computational prediction in the region 15 Kb on either side of the gene's TSS. Black bars within this image highlight the following regulatory peaks (if any are present): the peak closest to the TSS and the peak with the highest CRE score within this 30 Kb window. For those genes whose CRE predictions were tested experimentally, a red bar in this image indicates the location of the CRE that was tested. For Crx and Elovl4 the tested CRE is outside of this 30 Kb window and is therefore indicated in the ‘Large image’. ‘Links’ contains links to the UCSC genome browser, ENSEMBL, and NCBI database entries for the indicated gene, if available. ‘Max score (threshold of 200)’ contains the score of the highest predicted regulatory peak within the 30 Kb window around the gene's TSS. If this window does not contain any predicted peaks ≥ 200 (our cutoff threshold) no value is given (indicated by a dash). The next four columns of the table (‘Nr2e3-/-’ etc.) show the wild-type-to-mutant ratios of the averaged microarray scores for the given gene. For those genes to which more than one Affy tag correspond, the data for the first one listed under ‘Affy Mouse 430 2.0’ is given. Dark green = downregulated under high stringency (as described in METHODS); light green = downregulated under low stringency; red = upregulated under high stringency; orange = upregulated under low stringency. A dash indicates that the gene was not significantly a [file pone.0000643.s005.zip › Corbo_TableS1/images/Acoxl_TSS.gif]

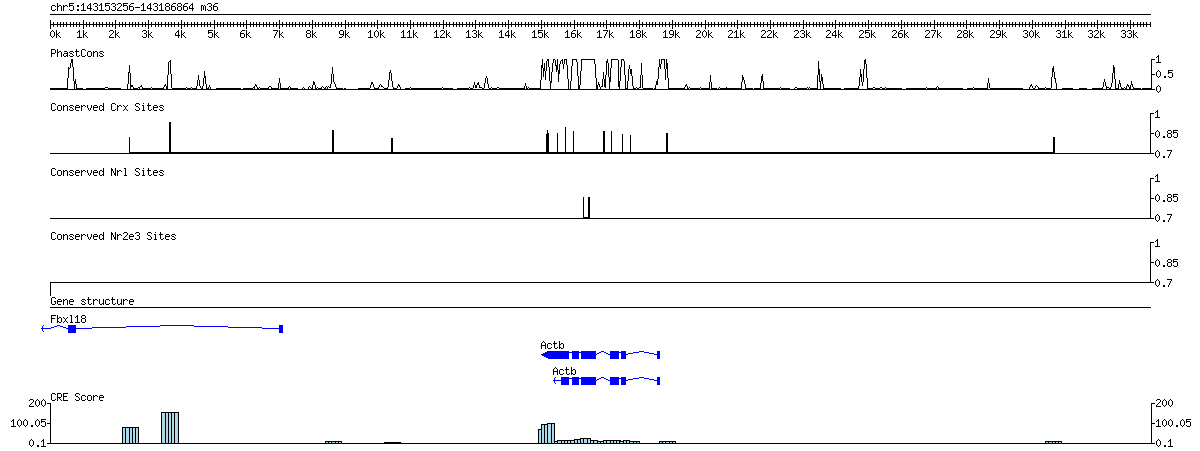

Supplement: Table S1 — Genes in the mouse photoreceptor transcription network. This table is a database which includes all genes dysregulated in Crx-/-, Nrl-/-, and/or Nr2e3-/- under high stringency criteria. Each row in the database represents a gene in the network and includes multiple links to additional types of information about that gene. ‘Large image gene +/− 15 Kb’ links to an image of our computational prediction of photoreceptor CREs in the genomic region 15 Kb upstream and downstream of the gene in question. ‘Closeup image TSS +/− 15 Kb’ links to an image of our computational prediction in the region 15 Kb on either side of the gene's TSS. Black bars within this image highlight the following regulatory peaks (if any are present): the peak closest to the TSS and the peak with the highest CRE score within this 30 Kb window. For those genes whose CRE predictions were tested experimentally, a red bar in this image indicates the location of the CRE that was tested. For Crx and Elovl4 the tested CRE is outside of this 30 Kb window and is therefore indicated in the ‘Large image’. ‘Links’ contains links to the UCSC genome browser, ENSEMBL, and NCBI database entries for the indicated gene, if available. ‘Max score (threshold of 200)’ contains the score of the highest predicted regulatory peak within the 30 Kb window around the gene's TSS. If this window does not contain any predicted peaks ≥ 200 (our cutoff threshold) no value is given (indicated by a dash). The next four columns of the table (‘Nr2e3-/-’ etc.) show the wild-type-to-mutant ratios of the averaged microarray scores for the given gene. For those genes to which more than one Affy tag correspond, the data for the first one listed under ‘Affy Mouse 430 2.0’ is given. Dark green = downregulated under high stringency (as described in METHODS); light green = downregulated under low stringency; red = upregulated under high stringency; orange = upregulated under low stringency. A dash indicates that the gene was not significantly a [file pone.0000643.s005.zip › Corbo_TableS1/images/Actb.gif]

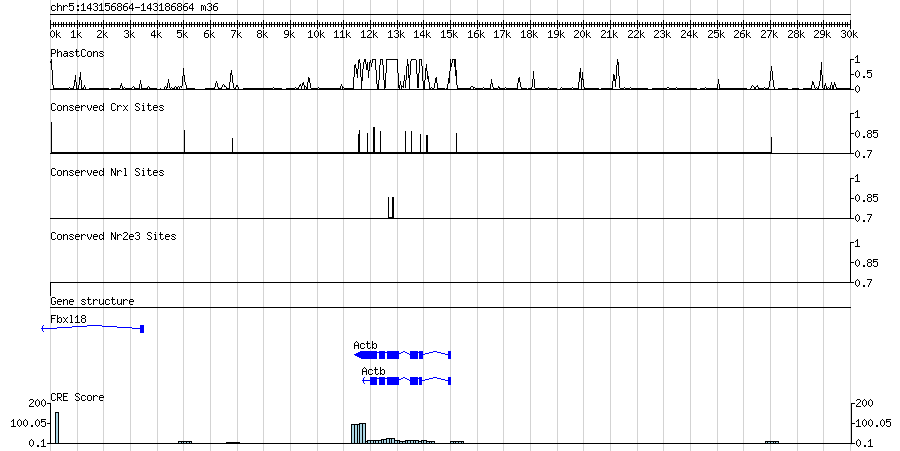

Supplement: Table S1 — Genes in the mouse photoreceptor transcription network. This table is a database which includes all genes dysregulated in Crx-/-, Nrl-/-, and/or Nr2e3-/- under high stringency criteria. Each row in the database represents a gene in the network and includes multiple links to additional types of information about that gene. ‘Large image gene +/− 15 Kb’ links to an image of our computational prediction of photoreceptor CREs in the genomic region 15 Kb upstream and downstream of the gene in question. ‘Closeup image TSS +/− 15 Kb’ links to an image of our computational prediction in the region 15 Kb on either side of the gene's TSS. Black bars within this image highlight the following regulatory peaks (if any are present): the peak closest to the TSS and the peak with the highest CRE score within this 30 Kb window. For those genes whose CRE predictions were tested experimentally, a red bar in this image indicates the location of the CRE that was tested. For Crx and Elovl4 the tested CRE is outside of this 30 Kb window and is therefore indicated in the ‘Large image’. ‘Links’ contains links to the UCSC genome browser, ENSEMBL, and NCBI database entries for the indicated gene, if available. ‘Max score (threshold of 200)’ contains the score of the highest predicted regulatory peak within the 30 Kb window around the gene's TSS. If this window does not contain any predicted peaks ≥ 200 (our cutoff threshold) no value is given (indicated by a dash). The next four columns of the table (‘Nr2e3-/-’ etc.) show the wild-type-to-mutant ratios of the averaged microarray scores for the given gene. For those genes to which more than one Affy tag correspond, the data for the first one listed under ‘Affy Mouse 430 2.0’ is given. Dark green = downregulated under high stringency (as described in METHODS); light green = downregulated under low stringency; red = upregulated under high stringency; orange = upregulated under low stringency. A dash indicates that the gene was not significantly a [file pone.0000643.s005.zip › Corbo_TableS1/images/Actb_TSS.gif]

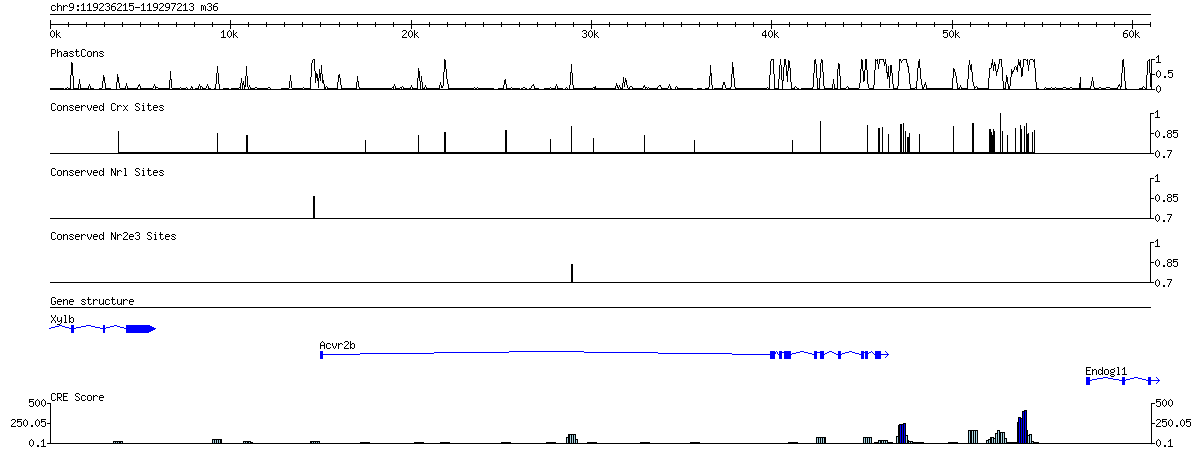

Supplement: Table S1 — Genes in the mouse photoreceptor transcription network. This table is a database which includes all genes dysregulated in Crx-/-, Nrl-/-, and/or Nr2e3-/- under high stringency criteria. Each row in the database represents a gene in the network and includes multiple links to additional types of information about that gene. ‘Large image gene +/− 15 Kb’ links to an image of our computational prediction of photoreceptor CREs in the genomic region 15 Kb upstream and downstream of the gene in question. ‘Closeup image TSS +/− 15 Kb’ links to an image of our computational prediction in the region 15 Kb on either side of the gene's TSS. Black bars within this image highlight the following regulatory peaks (if any are present): the peak closest to the TSS and the peak with the highest CRE score within this 30 Kb window. For those genes whose CRE predictions were tested experimentally, a red bar in this image indicates the location of the CRE that was tested. For Crx and Elovl4 the tested CRE is outside of this 30 Kb window and is therefore indicated in the ‘Large image’. ‘Links’ contains links to the UCSC genome browser, ENSEMBL, and NCBI database entries for the indicated gene, if available. ‘Max score (threshold of 200)’ contains the score of the highest predicted regulatory peak within the 30 Kb window around the gene's TSS. If this window does not contain any predicted peaks ≥ 200 (our cutoff threshold) no value is given (indicated by a dash). The next four columns of the table (‘Nr2e3-/-’ etc.) show the wild-type-to-mutant ratios of the averaged microarray scores for the given gene. For those genes to which more than one Affy tag correspond, the data for the first one listed under ‘Affy Mouse 430 2.0’ is given. Dark green = downregulated under high stringency (as described in METHODS); light green = downregulated under low stringency; red = upregulated under high stringency; orange = upregulated under low stringency. A dash indicates that the gene was not significantly a [file pone.0000643.s005.zip › Corbo_TableS1/images/Acvr2b.gif]

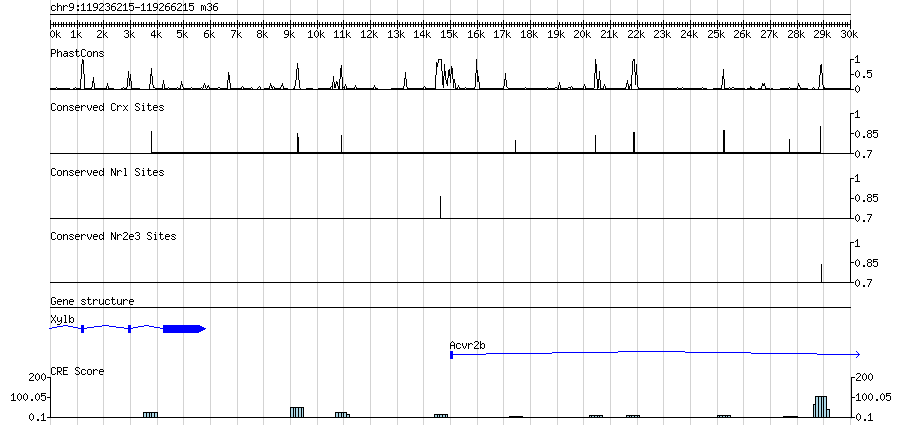

Supplement: Table S1 — Genes in the mouse photoreceptor transcription network. This table is a database which includes all genes dysregulated in Crx-/-, Nrl-/-, and/or Nr2e3-/- under high stringency criteria. Each row in the database represents a gene in the network and includes multiple links to additional types of information about that gene. ‘Large image gene +/− 15 Kb’ links to an image of our computational prediction of photoreceptor CREs in the genomic region 15 Kb upstream and downstream of the gene in question. ‘Closeup image TSS +/− 15 Kb’ links to an image of our computational prediction in the region 15 Kb on either side of the gene's TSS. Black bars within this image highlight the following regulatory peaks (if any are present): the peak closest to the TSS and the peak with the highest CRE score within this 30 Kb window. For those genes whose CRE predictions were tested experimentally, a red bar in this image indicates the location of the CRE that was tested. For Crx and Elovl4 the tested CRE is outside of this 30 Kb window and is therefore indicated in the ‘Large image’. ‘Links’ contains links to the UCSC genome browser, ENSEMBL, and NCBI database entries for the indicated gene, if available. ‘Max score (threshold of 200)’ contains the score of the highest predicted regulatory peak within the 30 Kb window around the gene's TSS. If this window does not contain any predicted peaks ≥ 200 (our cutoff threshold) no value is given (indicated by a dash). The next four columns of the table (‘Nr2e3-/-’ etc.) show the wild-type-to-mutant ratios of the averaged microarray scores for the given gene. For those genes to which more than one Affy tag correspond, the data for the first one listed under ‘Affy Mouse 430 2.0’ is given. Dark green = downregulated under high stringency (as described in METHODS); light green = downregulated under low stringency; red = upregulated under high stringency; orange = upregulated under low stringency. A dash indicates that the gene was not significantly a [file pone.0000643.s005.zip › Corbo_TableS1/images/Acvr2b_TSS.gif]

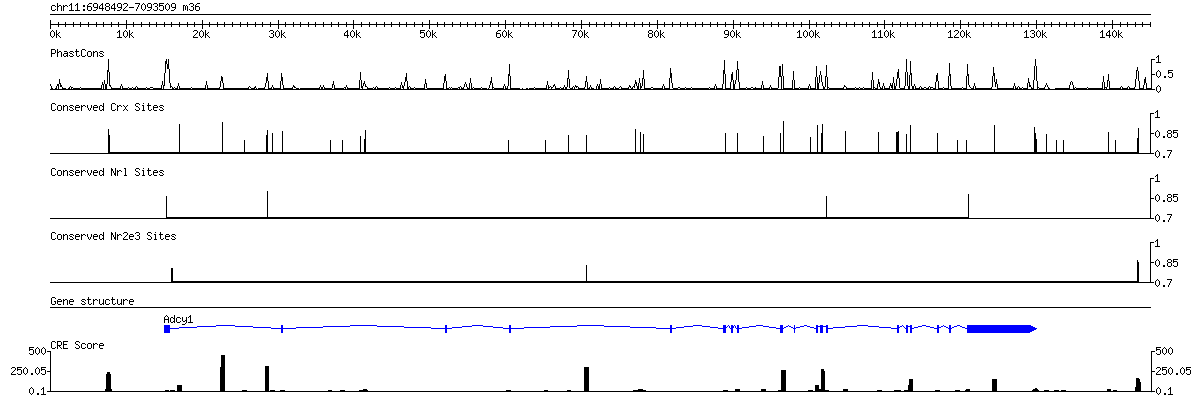

Supplement: Table S1 — Genes in the mouse photoreceptor transcription network. This table is a database which includes all genes dysregulated in Crx-/-, Nrl-/-, and/or Nr2e3-/- under high stringency criteria. Each row in the database represents a gene in the network and includes multiple links to additional types of information about that gene. ‘Large image gene +/− 15 Kb’ links to an image of our computational prediction of photoreceptor CREs in the genomic region 15 Kb upstream and downstream of the gene in question. ‘Closeup image TSS +/− 15 Kb’ links to an image of our computational prediction in the region 15 Kb on either side of the gene's TSS. Black bars within this image highlight the following regulatory peaks (if any are present): the peak closest to the TSS and the peak with the highest CRE score within this 30 Kb window. For those genes whose CRE predictions were tested experimentally, a red bar in this image indicates the location of the CRE that was tested. For Crx and Elovl4 the tested CRE is outside of this 30 Kb window and is therefore indicated in the ‘Large image’. ‘Links’ contains links to the UCSC genome browser, ENSEMBL, and NCBI database entries for the indicated gene, if available. ‘Max score (threshold of 200)’ contains the score of the highest predicted regulatory peak within the 30 Kb window around the gene's TSS. If this window does not contain any predicted peaks ≥ 200 (our cutoff threshold) no value is given (indicated by a dash). The next four columns of the table (‘Nr2e3-/-’ etc.) show the wild-type-to-mutant ratios of the averaged microarray scores for the given gene. For those genes to which more than one Affy tag correspond, the data for the first one listed under ‘Affy Mouse 430 2.0’ is given. Dark green = downregulated under high stringency (as described in METHODS); light green = downregulated under low stringency; red = upregulated under high stringency; orange = upregulated under low stringency. A dash indicates that the gene was not significantly a [file pone.0000643.s005.zip › Corbo_TableS1/images/Adcy1.gif]
